# Supplementary material for: Light-driven radical copper-catalyzed allylic amination via allylic copper intermediates
Source: Nat Commun. 2025 Nov 26;16:10425. doi: 10.1038/s41467-025-64951-5 (PMC12657895; doi:10.1038/s41467-025-64951-5)
Supplement: Supplementary file 1 — Supplementary Information [file 41467_2025_64951_MOESM1_ESM.pdf]

Supplementary Information

Light-Driven Radical Copper-Catalyzed Allylic Amination via Allylic Copper Intermediates

Table of Contents

Supplementary Information S1

Supplementary Methods S3

1. General Information S3

1.1 Materials and methods S3

2. Reaction Optimization of C(sp3)-N Couplings S4

3. Synthetic Process and Characterization Data of Substrates and Products S7

3.1 Starting Material Synthesis S7

3.2 General procedure 5 for C(sp3)-N couplings (GP5) S20

3.3 General procedure 6 for C(sp3)-N couplings (GP6) S21

3.4 Scale up reaction S21

3.5 Characterization data of product S22

4. Mechanistic Studies S60

4.1 UV-vis absorption of in-situ generated copper complex S60

4.2 Transient absorption experiments S60

4.3 Lifetime quenching experiments S60

4.4 Alkyl radical intermediate trapping experiments S61

4.5 Kinetic studies S62

4.6 Electronchemical of in-situ generated copper complex S65

4.7 Light on/off experiments S67

5. Computational Details S67

6. Copies of NMR Spectra S72

Supplementary Reference S202

Supplementary Methods

1. General Information

1.1 Materials and methods

Unless otherwise noted, all the materials were commercially available and used without further purification. All solvents were dried before use according to the standard methods. All reactions were performed in an N_2-filled glovebox using standard Schlenk techniques unless otherwise noted. All reactions were monitored by thin-layer chromatography (TLC), visualized by UV, Ninhydrin and $KMnO_4$ staining. Chromatographic purification of products was accomplished by silica gel chromatography. ^1H NMR, ^{13}C NMR and ^{19}F NMR, ^{31}P NMR spectra were recorded on a Bruker Avance II 400. NMR data is reported relative to internal $CHCl_3$ (^1H, δ = 7.26), $CDCl_3$ (^{13}C, δ = 77.0), CH_2Cl_2 (^1H, δ = 5.30), CD_2Cl_2 (^{13}C, δ = 53.0), DMSO (^1H, δ = 2.50), DMSO-*d6* (^{13}C, δ = 39.5), CH_3CN (^1H, δ = 1.94), CH_3CN-*d3* (^{13}C, δ = 1.3, 118.3). Data for ^1H NMR spectra are reported as follows: chemical shift (δ) in ppm; multiplicities are indicated s (singlet), brs (broad singlet), d (doublet), t (triplet), m (multiplet); coupling constants (J) are in Hertz (Hz) ^{13}C NMR spectra were reported as chemical shifts in ppm. The HRMS were obtained by using a Q Exactive high resolution liquid chromatography mass spectrometer (Q Exactive Plus) in ESI^+ mode or ESI^- mode. UV/Vis spectra were recorded using Shanghai Yoke T-UV756 instrument for samples in solution. The eight-position parallel light reaction system (RLH-18) and the large volume light reaction system (RLH-054) with 410 nm LEDs were purchased from Beijing Roger Technologies.

2. Reaction Optimization of C(sp3)-N Couplings

Procedure for optimization : To an oven-dried 10 mL reaction vial were added copper salt , ligand and 1 mL solvent in a nitrogen-filled glove box. The resulting mixture was stirred for 10 min, followed by adding base, *p*-toluidine and 1-(iodomethyl)-3-methylenecyclobutane in sequence, and sealed with a screwed cap. The sealed vial was placed on a photo-reactor under irradiation of 6W LEDs. The mixture was stirred at 25 °C for 15 h, quenched with H_2O, and extracted with ethyl acetate. The combined organic layers were dried over anhydrous Na_2SO_4, concentrated in vacuo. The crude product was analyzed by ^1H NMR with 1,3,5-trimethoxybenzene as the internal standard.

Supplementary Table 1. Preliminary screening

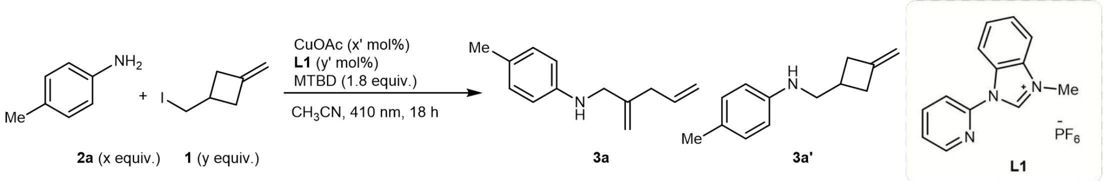

Entry[a]	x/y	Cu/L [mol%]	T	solvent	**3a**[b] [%]	**3a′**[b] [%]
1	1.5/1.0	5/5	-5°C	CH_3CN	<1	10
2	1.0/1.2	5/5	-5°C	CH_3CN	<1	17
3	1.0/1.5	5/5	-5°C	CH_3CN (0.05M)	<1	7
4	1.0/1.5	10/10	-5°C	CH_3CN	<1	20
5	1.0/1.5	10/10	r.t.	CH_3CN	<1	<5
6	1.0/1.5	10/10	r.t.	CH_3CN (0.05M)	<1	<5
7	1.0/1.5	20/10	r.t.	CH_3CN	<1	14
8	1.0/1.5	20/5	r.t.	CH_3CN	<1	19
9	1.0/1.2	10/10	r.t.	CH_3CN	<1	<5
**10**[c]	**1.0/1.5**	**10/10**	**r.t.**	**CH_3CN**	**14**	**<1**

[a]Reaction was carried out with **1** (x mmol), **2** (y mmol), CuOAc (x' mmol), **L1** (y' mmol) and MTBD (0.18 mmol) in CH_3CN (1 mL) at 410 nm irradiation for 18 hours. [b]Yields based on ^1H NMR analysis using 1,3,5-trimethoxybenzene as an external standard. [c]NaI (0.1 mmol) was added.

Supplementary Table 2. Base screening

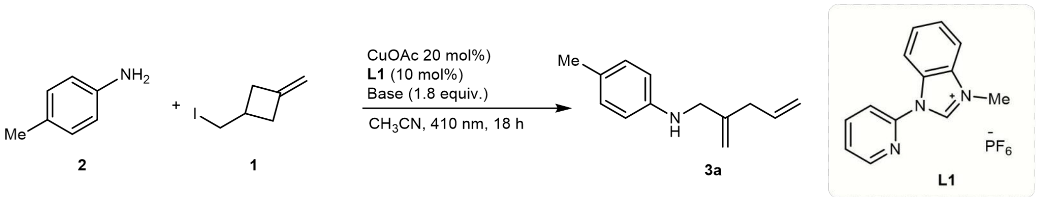

Entry[a]	Base	**3a**[b] [%]
1	K_2CO_3	25
2	K_3PO_4	21

3	Cs_2CO_3	14
4	Na_2CO_3	<1
5	Na_3PO_4	20
6	$KHCO_3$	11
7	K_2HPO_4	12
8	Et_3N	17
**9**	**TMG**	**34**
10	Pyridine	<5
11	DBU	29
12	BTMG	20
13	LiO*t*Bu	<5
14	DIPEA	22
15	DBN	<5
16	DBACO	n.r.
17	TBD	n.r.

[a]Reaction was carried out with **1** (0.15 mmol), **2** (0.1 mmol), CuOAc (0.02 mmol), **L1** (0.01 mmol) and base (0.18 mmol) in CH_3CN (1 mL) at 410 nm irradiation for 18 hours. [b]Yields based on ^1H NMR analysis using 1,3,5-trimethoxybenzene as an external standard.

Supplementary Table 3. Solvent screening

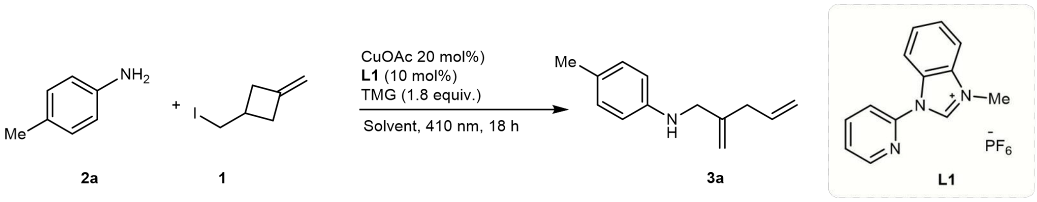

entry	Solvent	**3a** [%]
1	THF	46
2[a]	Dioxane	33
3	Toluene	29
**4**	**DMA**	**63**
5	CH_2Cl_2	12
6	DMSO	10
7	DMF	29

[a]Reaction was carried out with **1** (0.15 mmol), **2** (0.1 mmol), CuOAc (0.02 mmol), **L1** (0.01 mmol) and TMG (0.18 mmol) in **solvent** (1 mL) at 410 nm irradiation for 18 hours. [b]Yields based on ^1H NMR analysis using 1,3,5-trimethoxybenzene as an external standard.

Supplementary Table 4. Copper salt screening

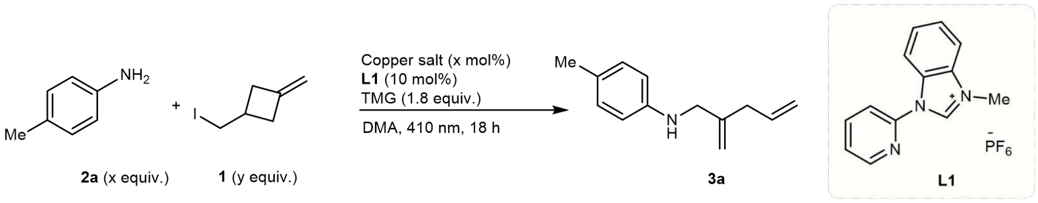

Entry[a]	x/y	Cu salt	Solvent	**3a**[b] [%]
**1**	**1.0/1.5**	**CuI**	**DMA**	**72**
2	1.0/1.5	$Cu(MeCN)_4PF_6$	DMA	71
3	1.0/1.5	$Cu(OAc)_2$	DMA	30
4	1.0/1.5	$Cu(OTf)_2$	DMA	57
5	1.0/1.5	CuOAc	DMA (0.2 M)	66
6	1.0/2.5	CuOAc	DMA	63

[a]Reaction was carried out with **1** (0.15 mmol), **2** (0.1 mmol), Copper salt (0.02 mmol), **L1** (0.01 mmol) and TMG (0.18 mmol) in DMA (1 mL) at 410 nm irradiation for 18 hours. [b]Yields based on ^1H NMR analysis using 1,3,5-trimethoxybenzene as an external standard.

Supplementary Table 5. Additives screening.

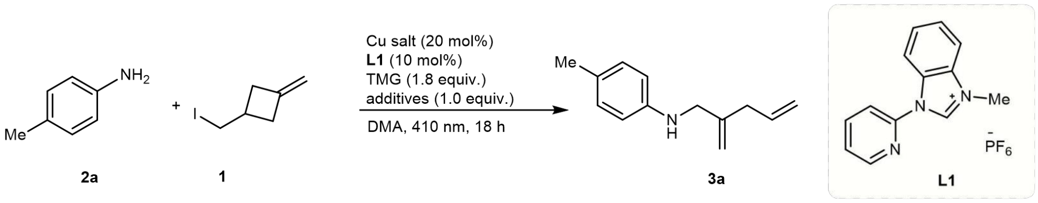

Entry[a]	Copper salt (x mol%)	additives	Temp.	**3a**[b] [%]
1	CuI (20 mol%)	KI	r.t.	69
2	CuI (20 mol%)	KBr	r.t.	49
3	CuI (20 mol%)	KF	r.t.	40
4	CuI (20 mol%)	TBAI	r.t.	68
5	CuI (1.0 equiv.)	-	50°C	82
6	CuI (1.0 equiv.)	-	r.t.	83
**7**	**CuI (50 mol%)**	**-**	**r.t.**	**86 (85)**
8	CuI (20 mol%)	-	r.t.	65

[a]Reaction was carried out with **1** (0.15 mmol), **2** (0.1 mmol), CuI (x mmol), **L1** (0.01 mmol), TMG (0.18 mmol) and additives (x mmol) in DMA (1 mL) at 410 nm irradiation for 18 hours. [b]Yields based on ^1H NMR analysis using 1,3,5-trimethoxybenzene as an external standard and isolated yields are given in parentheses.

Supplementary Table 6. Control experiments

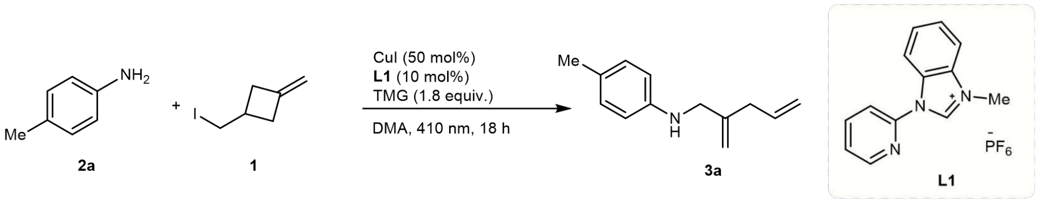

Entry[a]	variants from standard conditions	Yield[b] [%]
1	w/o light	n.d.
2	w/o CuI	n.d.
3	w/o **L1**	n.d.
4	open in air	n.d.

[a]Reaction was carried out with **1** (0.15 mmol), **2** (0.1 mmol), Copper salt (0.05 mmol), **L1** (0.01 mmol) and TMG (0.18 mmol) in DMA (1 mL) at 410 nm irradiation for 18 hours. [b]Yields based on ^{1}H NMR analysis using 1,3,5-trimethoxybenzene as an external standard.

Supplementary Table 7. Alkyl amines as the nucleophile

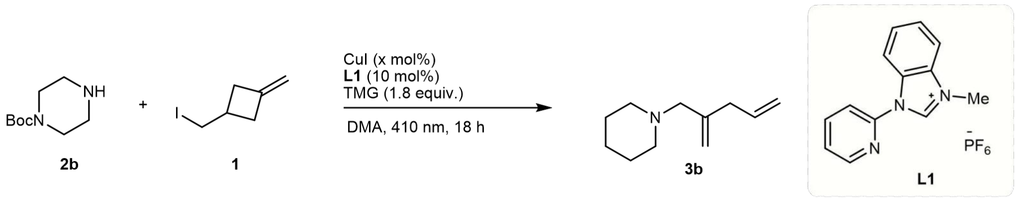

Entry[a]	Copper salt (x mol%)	**3b** [%]
1	CuI (50 mol%)	95
2	CuI (10 mol%)	98 (93)

Isolated yields are given in parentheses.

3. Synthetic Process and Characterization Data of Substrates and Products

3.1 Starting Material Synthesis

General procedure 1 (GP1)

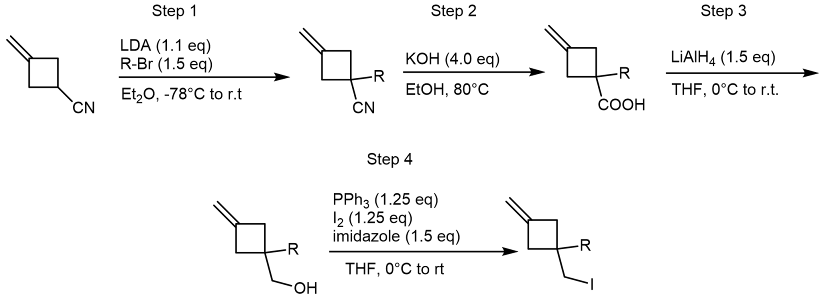

[Step 1]: To a solution of diisopropylamine (1.1 equiv.) in Et_2O at 0°C was added *n*-BuLi (1.1 equiv.) dropwise. The resulting mixture was stirred for 30 min, before 3-methylenecyclobutane-1-carbonitrile (1.0 equiv.) was added at -78°C. Then activated alkyl bromides (1.5 equiv.) was added dropwise at -78°C for 6 hours. The progress of the reactions was monitored by TLC. After the reaction was completed, the mixture was quenched by saturated NH_4Cl and extracted with ethyl acetate, dried over Na_2SO_4, filtered and concentrated under reduced pressure. The resulting alkylation product was purified by flash chromatography.

[Step 2]: To a solution of KOH (4.0 equiv.) in EtOH was added cyanide compound. The reaction mixture was stirred at 80°C for 12 hours. After cooling to room

temperature, the solvent was removed in vacuo. The residue was added water and washed by Et_2O. The aqueous layer was added 1M HCl to adjust pH to 2 and extracted with DCM. Then combined organic layer was concentrated to afford carboxylic acid product.

[Step 3]: To a solution of carboxylic acid compounds in THF was added another solution of $LiAlH_4$ in THF dropwise at 0°C. The resulting mixture was stirred at room temperature for 6 hours. After the reaction was completed, the mixture was diluted with Et_2O and quenched by water and 15%wt NaOH (aq). Then dried over $MgSO_4$, filtered, concentrated to give alcohol product.

[Step 4]: To a solution of the PPh_3 (1.25 equiv.), Imidazole (1.5 equiv.) and I_2 (1.25 equiv.) in dry DCM (0.3 M) was added alcohol (1.0 equiv.) portionwise at 0°C. The reaction was stirred for additional 12 hours at room temperature. The progress of the reactions was monitored by TLC. After the reaction was completed, the mixture was washed with a solution of $Na_2S_2O_3$, H_2O, brine, dried over Na_2SO_4, filtered and concentrated under reduced pressure. The resulting alkyl iodide was purified by flash chromatography.

General procedure 2 (GP2)

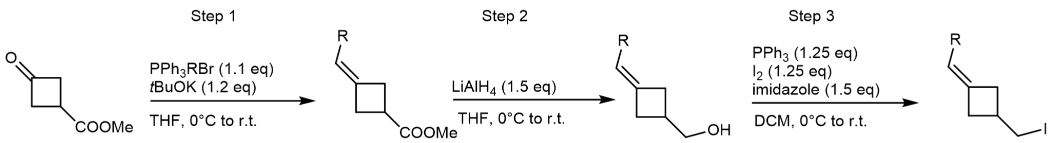

[Step 1]: A mixture of the Wittig reagent (1.1 equiv.) and *t*BuOK (1.2 equiv.) in THF at 0°C for 2 hours. Then ketone (1.0 equiv.) was added, the resulting mixture was stirred at room temperature for 24 hours. The progress of the reactions was monitored by TLC. After the reaction was completed, the mixture was quenched by water and extracted with ethyl acetate, dried over Na_2SO_4, filtered and concentrated under reduced pressure. The resulting alkene product was purified by flash chromatography.

[Step 2]: To a solution of ester compounds in THF was added another solution of $LiAlH_4$ in THF dropwise at 0°C. The resulting mixture was stirred at room temperature for 6 hours. After the reaction was completed, the mixture was diluted with Et_2O and quenched by water and 15wt% NaOH (aq). Then dried over $MgSO_4$, filtered, concentrated to give alcohol product.

[Step 3]: To a solution of the PPh_3 (1.25 equiv.), Imidazole (1.5 equiv.) and I_2 (1.25 equiv.) in dry DCM (0.3 M) was added alcohol (1.0 equiv.) portionwise at 0°C. The reaction was stirred for additional 12 hours at room temperature. The progress of the reactions was monitored by TLC. After the reaction was completed, the mixture was washed with a solution of $Na_2S_2O_3$, H_2O, brine, dried over Na_2SO_4, filtered and

concentrated under reduced pressure. The resulting alkyl iodide was purified by flash chromatography.

General procedure 3 (GP3)

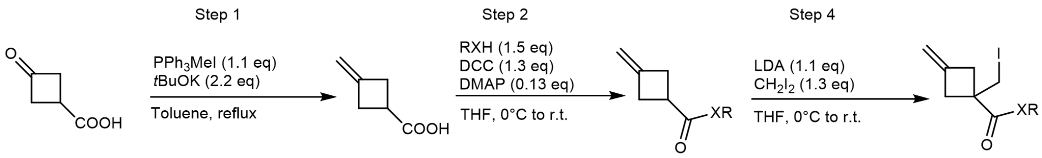

[Step 1]: A mixture of the PPh_3MeI (1.1 equiv.) and *t*BuOK (2.2 equiv.) in THF at 0°C for 2 hours, Then 3-oxocyclobutane-1-carboxylic acid (1.0 equiv.) was added to the mixture. The resulting mixture was stirred at reflux for 48 hours. After cooling to room temperature, the solvent was removed in vacuo. The residue was added water and washed by DCM. The aqueous layer was added 1M HCl to adjust pH to 2 and extracted with DCM. Then combined organic layer was concentrated to afford carboxylic acid product.

[Step 2]: To a solution of *N* or *O*-nucleophiles (1.0 equiv.) in DCM was treated with 1,3-dicyclohexylcarbodiimide (1.3 equiv.), 4-dimethylaminopyridine (0.13 equiv.), 3-methylenecyclobutane-1-carboxylic acid (1.3 equiv.) at 0°C. Then resulting mixture was stirred at room temperature for 12 hours. After the reaction was completed, the mixture was filtered and the filtrate was concentrated. The residue was purified by flash chromatography to afford the ester or amide compound.

[Step 3]: To a solution of diisopropylamine (1.1 equiv.) in THF was added *n*-BuLi (1.1 equiv.) dropwise at 0°C. The resulting mixture was stirred for 30 min, before ester or amide compounds (1.0 equiv.) was added to the mixture. The resulting mixture was stirred for 2 hours, followed by adding CH_2I_2 (1.3 equiv.). The mixture was stirred at room temperature for additional 6 hours. After the reaction was completed, the mixture was quenched by saturated NH_4Cl (aq) and extracted with ethyl acetate. Then combined organic layer dried over Na_2SO_4, filtered and concentrated. The residue was purified by flash chromatography to afford alkyl iodides.

General procedure 4 (GP4)

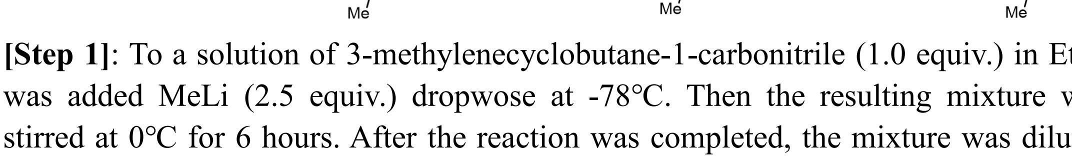

[Step 1]: To a solution of 3-methylenecyclobutane-1-carbonitrile (1.0 equiv.) in Et_2O was added MeLi (2.5 equiv.) dropwose at -78°C. Then the resulting mixture was stirred at 0°C for 6 hours. After the reaction was completed, the mixture was diluted with Et_2O, quenched by saturated NH_4Cl (aq) and extracted with Et_2O. The combined organic layer dried over Na_2SO_4, filtered and concentrated. The residue was purified by flash chromatography to afford the ketone compound.

[Step 2]: To a solution of ketone compound in EtOH was added $NaBH_4$ (1.2 equiv.) at 0°C. Then the resulting mixture was stirred at room temperature for 12 hours. After the reation was completed, The mixture was quenched by 1M HCl and extracted with ethyl acetate. The combined organic layer dried over Na_2SO_4, filtered and concentrated to give the alcohol product.

[Step 3]: To a solution of the PPh_3 (1.25 equiv.), Imidazole (1.5 equiv.) and I_2 (1.25 equiv.) in dry DCM (0.3 M) was added alcohol (1.0 equiv.) portionwise at 0°C. The reaction was stirred for additional 12 hours at room temperature. The progress of the reactions was monitored by TLC. After the reaction was completed, the mixture was washed with a solution of $Na_2S_2O_3$, H_2O, brine, dried over Na_2SO_4, filtered and concentrated under reduced pressure. The resulting alkyl iodide was purified by flash chromatography.

1-(Iodomethyl)-3-methylenecyclobutane (S1)

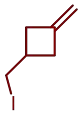

Synthesized according to **GP1**, colorless oil.

^{1}H NMR (400 MHz, $CDCl_3$): δ 4.87 – 4.80 (m, 2H), 3.31 (d, J = 7.7 Hz, 2H), 2.86 – 2.77 (m, 2H), 2.71 – 2.62 (m, 1H), 2.39 – 2.29 (m, 2H).
^{13}C NMR (101 MHz, $CDCl_3$): δ 143.4, 106.8, 38.5, 33.3, 13.4.
HRMS (ESI): Calcd for $C_6H_9I^+$ $[M]^+$:207.9749, found 207.9742.

((1-(Iodomethyl)-3-methylenecyclobutyl)methyl)benzene (S2)

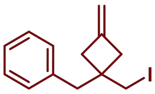

Synthesized according to **GP1**, colorless oil.

^{1}H NMR (400 MHz, $CDCl_3$): δ 7.35 – 7.23 (m, 5H), 4.97 – 4.88 (m, 2H), 3.31 (s, 2H), 2.92 (s, 2H), 2.78 – 2.67 (m, 2H), 2.54 – 2.43 (m, 2H).
^{13}C NMR (101 MHz, $CDCl_3$): δ 141.7, 138.1, 129.4, 128.3, 126.5, 108.2, 43.3, 41.5, 39.5, 21.0.
HRMS (ESI): Calcd for $C_{13}H_{15}^+$ $[M-I]^+$:171.1168, found 171.1175.

1-((1-(Iodomethyl)-3-methylenecyclobutyl)methyl)-2-methylbenzene (S3)

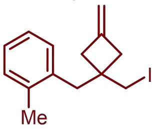

Synthesized according to **GP1**, colorless oil.

^{1}H NMR (400 MHz, CDCl$_3$): δ 7.22 – 7.14 (m, 4H), 4.90 – 4.83 (m, 2H), 3.56 (s, 2H), 2.93 (s, 2H), 2.72 – 2.63 (m, 2H), 2.53 – 2.44 (m, 2H), 2.36 (s, 3H).
^{13}C NMR (101 MHz, CDCl$_3$): δ 142.7, 137.1, 136.6, 130.6, 129.8, 126.5, 125.7, 107.5, 40.8, 39.8, 39.4, 22.3, 20.4.
HRMS (ESI): Calcd for $C_{14}H_{17}^{+}$ [M-I]$^{+}$: 185.1325, found 185.1320.

1-((1-(Iodomethyl)-3-methylenecyclobutyl)methyl)-2-(trifluoromethyl)benzene (S4)

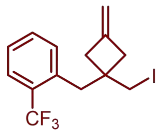

Synthesized according to **GP1**, colorless oil.

^{1}H NMR (400 MHz, CDCl$_3$): δ 7.71 – 7.65 (m, 1H), 7.55 – 7.48 (m, 1H), 7.40 – 7.30 (m, 2H), 4.88 – 4.82 (m, 2H), 3.58 (s, 2H), 3.13 (s, 2H), 2.75 – 2.62 (m, 2H), 2.57 – 2.46 (m, 2H).
^{13}C NMR (101 MHz, CDCl$_3$): δ 142.5, 137.2, 131.5, 131.2, 129.7 (q, J = 29.2 Hz), 126.6, 126.6 (q, J = 10.1 Hz), 124.5 (q, J = 274.3 Hz), 107.8, 41.0, 39.6, 39.1, 26.9, 21.8.
^{19}F NMR (376 MHz, CDCl$_3$): δ -58.3 (3F).
HRMS (ESI): Calcd for $C_{14}H_{14}F_3^{+}$ [M-I]$^{+}$: 239.1042, found: 239.1040.

1-Fluoro-3-((1-(iodomethyl)-3-methylenecyclobutyl)methyl)benzene (S5)

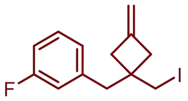

Synthesized according to **GP1**, colorless oil.

^{1}H NMR (400 MHz, CDCl$_3$): δ 7.32 – 7.22 (m, 1H), 7.10 – 7.05 (m, 1H), 7.03 – 6.92 (m, 2H), 4.96 – 4.90 (m, 2H), 3.29 (s, 2H), 2.91 (s, 2H), 2.74 – 2.66 (m, 2H), 2.53 – 2.44 (m, 2H).
^{13}C NMR (101 MHz, CDCl$_3$): δ 162.7 (d, J = 245.6 Hz), 141.3, 140.6 (d, J = 7.2 Hz), 129.7 (d, J = 8.5 Hz), 125.1 (d, J = 2.9 Hz), 116.2 (d, J = 20.5 Hz), 113.5 (d, J = 20.9 Hz), 108.4, 43.1, 41.5, 39.4, 20.5.
^{19}F NMR (376 MHz, CDCl$_3$): δ -113.3 (1F).
HRMS (ESI): Calcd for $C_{13}H_{14}F^{+}$ [M-I]$^{+}$: 189.1074, found: 189.1071..

1-((1-(Iodomethyl)-3-methylenecyclobutyl)methyl)-3-(trifluoromethyl)benzene (S6)

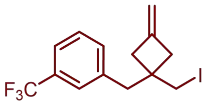

Synthesized according to **GP1**, colorless oil.

^{1}H NMR (400 MHz, CDCl$_3$): δ 7.56 – 7.48 (m, 3H), 7.46 – 7.40 (m, 1H), 4.99 – 4.92 (m, 2H), 3.26 (s, 2H), 2.98 (s, 2H), 2.77 – 2.66 (m, 2H), 2.53 – 2.45 (m, 2H).
^{13}C NMR (101 MHz, CDCl$_3$): δ 141.0, 139.0, 132.8, 130.7 (q, J = 32.0 Hz), 128.8, 126.0 (q, J = 3.8 Hz), 124.1 (q, J = 272.2 Hz), 123.5 (q, J = 3.9 Hz), 108.6, 43.2, 41.4, 39.4, 20.2.
^{19}F NMR (376 MHz, CDCl$_3$): δ -62.5 (3F).
HRMS (ESI): Calcd for $C_{14}H_{14}F_3^+$ [M-I]$^+$: 239.1042, found: 239.1040.

1-(*tert*-Butyl)-4-((1-(iodomethyl)-3-methylenecyclobutyl)methyl)benzene (S7)

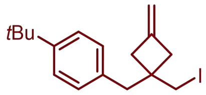

Synthesized according to **GP1**, colorless oil.

^{1}H NMR (400 MHz, CDCl$_3$): δ 7.35 (d, J = 8.3 Hz, 2H), 7.25 (d, J = 8.3 Hz, 2H), 4.99 – 4.90 (m, 2H), 3.34 (s, 2H), 2.91 (s, 2H), 2.80 – 2.68 (m, 2H), 2.57 – 2.45 (m, 2H), 1.35 (s, 9H).
^{13}C NMR (101 MHz, CDCl$_3$): δ 149.3, 141.8, 135.0, 129.1, 125.2, 108.1, 42.8, 41.5, 39.5, 34.4, 31.4, 26.9, 21.2.
HRMS (ESI): Calcd for $C_{17}H_{23}^+$ [M-I]$^+$: 227.1794, found 227.1797.

1-((1-(Iodomethyl)-3-methylenecyclobutyl)methyl)-4-(trifluoromethyl)benzene (S8)

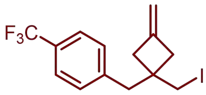

Synthesized according to **GP1**, colorless oil.

^{1}H NMR (400 MHz, CDCl$_3$): δ 7.56 (d, J = 8.0 Hz, 2H), 7.41 (d, J = 8.0 Hz, 2H), 4.99 – 4.89 (m, 2H), 3.26 (s, 2H), 2.98 (s, 2H), 2.75 – 2.66 (m, 2H), 2.54 – 2.44 (m, 2H).
^{13}C NMR (101 MHz, CDCl$_3$): δ 142.2, 141.0, 129.7, 128.9 (q, J = 32.2 Hz), 125.8 (q, J = 272.7 Hz), 125.3 (q, J = 3.7 Hz), 108.6, 43.2, 41.5, 39.4, 20.2.
^{19}F NMR (376 MHz, CDCl$_3$): δ -62.4 (3F).
HRMS (ESI): Calcd for $C_{14}H_{14}F_3^+$ [M-I]$^+$: 239.1042, found: 239.1040.

1-Fluoro-4-((1-(iodomethyl)-3-methylenecyclobutyl)methyl)benzene (S9)

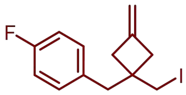

Synthesized according to **GP1**, colorless oil.

^{1}H NMR (400 MHz, CDCl$_3$): δ 7.30 – 7.23 (m, 2H), 7.03 – 6.97 (m, 2H), 5.00 – 4.88

(m, 2H), 3.28 (s, 2H), 2.89 (s, 2H), 2.75 – 2.63 (m, 2H), 2.53 – 2.41 (m, 2H).
^{13}C NMR (101 MHz, CDCl$_3$): δ 161.7 (d, J = 244.8 Hz), 141.3, 133.7 (d, J = 3.0 Hz), 130.8 (d, J = 7.3 Hz), 115.1 (d, J = 21.0 Hz), 108.4, 42.5, 41.3, 39.4, 20.7.
^{19}F NMR (376 MHz, CDCl$_3$): δ -116.3 (1F).
HRMS (ESI): Calcd for $C_{13}H_{14}F^+$ [M-I]$^+$: 189.1074, found: 189.1071.

1,2-Dichloro-4-((1-(iodomethyl)-3-methylenecyclobutyl)methyl)benzene (S10)

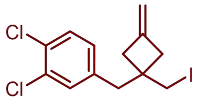

Synthesized according to **GP1**, colorless oil.

^{1}H NMR (400 MHz, CDCl$_3$): δ 7.41 – 7.34 (m, 2H), 7.17 – 7.11 (m, 1H), 4.96 – 4.89 (m, 2H), 3.26 (s, 2H), 2.87 (s, 2H), 2.74 – 2.62 (m, 2H), 2.53 – 2.42 (m, 2H).
^{13}C NMR (101 MHz, CDCl$_3$): δ 140.9, 138.3, 132.3, 131.2, 130.7, 130.3, 128.8, 108.7, 42.5, 41.4, 39.3, 20.1.
HRMS (ESI): Calcd for $C_{13}H_{13}Cl_2^+$ [M-I]$^+$: 239.0389, found 239.0380.

(1-(1-(Iodomethyl)-3-methylenecyclobutyl)ethyl)benzene (S11)

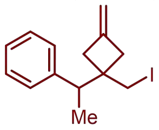

Synthesized according to **GP1**, colorless oil.

^{1}H NMR (400 MHz, CDCl$_3$): δ 7.40 – 7.16 (m, 5H), 4.94 – 4.81 (m, 2H), 3.52 (d, J = 9.8 Hz, 1H), 3.24 – 3.20 (m, 1H), 3.15 (q, J = 7.2 Hz, 1H), 3.01 – 2.92 (m, 1H), 2.90 – 2.82 (m, 1H), 2.48 – 2.40 (m, 1H), 2.36 – 2.28 (m, 1H), 1.34 (d, J = 7.2 Hz, 3H).
^{13}C NMR (101 MHz, CDCl$_3$): δ 143.7, 142.5, 128.2, 128.0, 126.5, 107.9, 43.0, 41.9, 39.3, 37.2, 23.0, 16.3.
HRMS (ESI): Calcd for $C_{14}H_{17}^+$ [M-I]$^+$: 185.1325, found 185.1328.

1-(Iodomethyl)-1-(2-methoxyethyl)-3-methylenecyclobutane (S12)

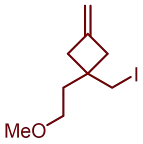

Synthesized according to **GP1**, colorless oil.

^{1}H NMR (400 MHz, CDCl$_3$): δ 4.90 – 4.84 (m, 2H), 3.42 (s, 2H), 3.39 (t, J = 6.6 Hz, 2H), 3.32 (s, 3H), 2.65 – 2.44 (m, 4H), 1.90 (t, J = 6.6 Hz, 2H).
^{13}C NMR (101 MHz, CDCl$_3$): δ 142.1, 108.1, 69.4, 58.8, 41.9, 37.5, 37.3, 20.6.
HRMS (ESI): Calcd for $C_9H_{15}O^+$ [M-I]$^+$: 139.1117, found 139.1120.

1-Allyl-1-(iodomethyl)-3-methylenecyclobutane (S13)

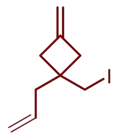

Synthesized according to **GP1**, colorless oil.

^{1}H NMR (400 MHz, $CDCl_3$): δ 5.75 – 5.61 (m, 1H), 5.23 – 5.09 (m, 2H), 4.92 – 4.88 (m, 2H), 3.38 (s, 2H), 2.62 – 2.44 (m, 4H), 2.36 (d, J = 7.3 Hz, 2H).
^{13}C NMR (101 MHz, $CDCl_3$): δ 141.6, 133.5, 118.3, 108.3, 42.5, 41.1, 37.8, 31.6, 20.6.
HRMS (ESI): Calcd for $C_9H_{13}^+$ [M-I]$^+$: 121.1012, found 121.1016.

1-(But-2-yn-1-yl)-1-(iodomethyl)-3-methylenecyclobutane (S14)

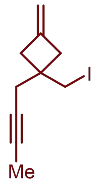

Synthesized according to **GP1**, colorless oil.

^{1}H NMR (400 MHz, $CDCl_3$): δ 4.92 – 4.88 (m, 2H), 3.53 (s, 2H), 2.63 – 2.48 (m, 4H), 2.44 (q, J = 2.5 Hz, 2H), 1.78 (t, J = 2.5 Hz, 3H).
^{13}C NMR (101 MHz, $CDCl_3$): δ 141.0, 108.4, 77.4, 75.4, 40.9, 38.3, 31.6, 29.1, 22.6, 20.0.
HRMS (ESI): Calcd for $C_{10}H_{13}^+$ [M-I]$^+$: 133.1012, found 133.1018.

1-(1-(Iodomethyl)-3-methylenecyclobutyl)-4-methylbenzene (S15)

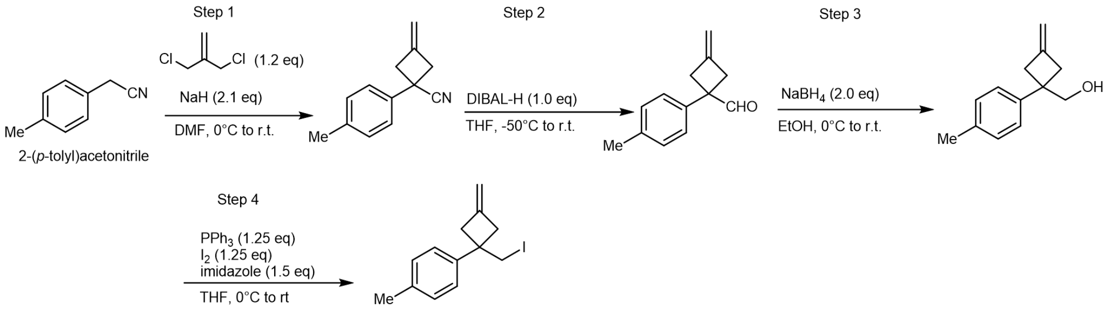

[Step 1]: To a solution of 3-5 dichloro-2-butene (1.2 equiv.) in dry DMF was added a 60% dispersion of NaH in mineral oil (2.1 equiv.) at 0°C. And then to the above suspension under vigorous stirring was added 2-(*p*-tolyl)acetonitrile (1.0 equiv.) dropwise in DMF over 30 minutes. After 2 hours, the reaction was quenched with saturated NH_4Cl and extracted with Et_2O, The organic layer was washed with water, brine, dried over Na_2SO_4, filtered and concentrated under reduced pressure. The resulting product was purified by flash chromatography.

[Step 2]: A flame-dried flask containing a 1.0 M solution of DIBAL-H in hexane (2.0 equiv) was diluted with anhydrous THF and cooled to -50°C. To this solution was added via cannula a solution of 3-methylene-1-(*p*-tolyl)cyclobutane-1-carbonitrile (1.0 equiv) in dry THF. After 3 h at -50°C, the reaction was quenched with saturated NH_4CI and was allowed to stir at room temperature for 30 minutes. The organic layers were separated and the aqueous layer was further extracted with EtOAc. The organic layers were combined and concentrated in vacuo. The resulting aldehyde product was purified by flash chromatography.

[Step 2]: A solution of aldehyde compounds (1.0 equiv) in ary EtOH was cooled to 0°C and treated with $NaBH_4$ (2.0 equiv). The reaction mixture was quenched with MeOH after stirring for one hour, then concentrated in vacuo to dryness. The residue was taken up in ether and washed with saturated Na_2CO_3 and brine. The organic layers were combined, dried over Na_2SO_4, filtered, and concentrated in vacuo. The resulting alcohol product was purified by flash chromatography.

[Step 4]: To a solution of the PPh_3 (1.25 equiv.), Imidazole (1.5 equiv.) and I_2 (1.25 equiv.) in dry DCM (0.3 M) was added alcohol (1.0 equiv.) portionwise at 0°C. The reaction was stirred for additional 12 hours at room temperature. The progress of the reactions was monitored by TLC. After the reaction was completed, the mixture was washed with a solution of $Na_2S_2O_3$, H_2O, brine, dried over Na_2SO_4, filtered and concentrated under reduced pressure. The resulting alkyl iodide was purified by flash chromatography.

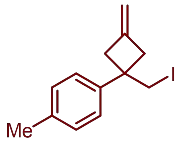

Synthesized according to **GP1**, colorless oil.

^{1}H NMR (400 MHz, $CDCl_3$): δ 7.18 – 7.08 (m, 4H), 4.97 – 4.90 (m, 2H), 3.64 (s, 2H), 3.21 – 3.11 (m, 2H), 2.94 – 2.86 (m, 2H), 2.35 (s, 3H).
^{13}C NMR (101 MHz, $CDCl_3$): δ 143.0, 142.0, 136.0, 128.7, 126.2, 108.0, 43.1, 42.9, 24.0, 21.1
HRMS (ESI): Calcd for $C_{13}H_{15}^+$ $[M-I]^+$: 171.1168, found 171.1174.

1-(Iodomethyl)-3-methylenecyclobutane-1-carbonitrile (S16)

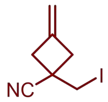

Synthesized according to **GP3**, colorless oil.

^{1}H NMR (400 MHz, $CDCl_3$): δ 5.02 (s, 1H), 3.45 (s, 1H), 3.29 (d, *J* = 16.5 Hz, 1H), 2.81 (d, *J* = 16.0 Hz, 1H).

^{13}C NMR (101 MHz, $CDCl_3$): δ 137.1, 122.3, 110.9, 51.5, 43.2, 33.8.
HRMS (ESI): Calcd for $C_7H_8N^+$ $[M-I]^+$: 106.0651, found: 106.0658. .

(1-(Iodomethyl)-3-methylenecyclobutyl)(morpholino)methanone (S17)

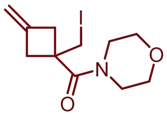

Synthesized according to **GP3**, colorless oil.

^{1}H NMR (400 MHz, $CDCl_3$): δ 4.93 – 4.89 (m, 2H), 3.71 – 3.63 (m, 6H), 3.50 (s, 2H), 3.45 – 3.33 (m, 2H), 3.31 – 3.22 (m, 2H), 2.70 – 2.59 (m, 2H).
^{13}C NMR (101 MHz, $CDCl_3$): δ 171.3, 140.3, 108.8, 66.9, 46.7, 44.0, 42.8, 41.9, 12.3.
HRMS (ESI): Calcd for $C_{11}H_{17}INO_2^+$ $[M+H]^+$: 322.0299, found 322.0304.

1-**(Iodomethyl)-3-(3-methylbutylidene)cyclobutane (S18)**

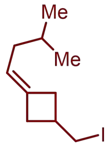

Synthesized according to **GP2** , colorless oil.

^{1}H NMR (400 MHz, $CDCl_3$): δ 5.26 – 5.14 (m, 1H), 3.30 (d, J = 7.7 Hz, 2H), 2.83 – 2.73 (m, 2H), 2.71 – 2.61 (m, 1H), 2.35 – 2.19 (m, 2H), 1.75 (t, J = 7.1 Hz, 2H), 1.63 – 1.51 (m, 1H), 0.87 (d, J = 6.6 Hz, 6H).
^{13}C NMR (101 MHz, $CDCl_3$): δ 133.1, 121.3, 37.5, 37.3, 36.1, 33.8, 28.6, 22.3, 22.3, 14.0.
HRMS (ESI): Calcd for $C_{10}H_{17}^+$ $[M-I]^+$: 137.1325, found 137.1328..

((3-(Iodomethyl)cyclobutylidene)methyl)benzene (S19)

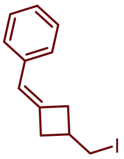

Synthesized according to **GP2** , colorless oil.

^{1}H NMR (400 MHz, $CDCl_3$): δ 7.34 – 7.27 (m, 2H), 7.23 – 7.13 (m, 3H), 6.24 – 6.17 (m, 1H), 3.40 – 3.34 (m, 2H), 3.24 – 3.13 (m, 1H), 3.07 – 2.95 (m, 1H), 2.90 – 2.78 (m, 1H), 2.75 – 2.66 (m, 1H), 2.58 – 2.49 (m, 1H).
^{13}C NMR (101 MHz, $CDCl_3$): δ 137.5, 137.2, 128.4, 127.1, 126.1, 122.6, 39.3, 39.1, 34.9, 13.3.
HRMS (ESI): Calcd for $C_{12}H_{13}^+$ $[M-I]^+$: 157.1012, found 157.1011.

1-(1-Iodoethyl)-3-methylenecyclobutane (S20)

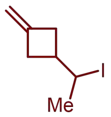

Synthesized according to **GP4**, colorless oil.

^{1}H NMR (400 MHz, CDCl$_3$): δ 4.86 – 4.77 (m, 2H), 4.22 – 4.10 (m, 1H), 2.84 – 2.67 (m, 2H), 2.63 – 2.52 (m, 1H), 2.47 – 2.27 (m, 2H), 1.84 (d, J = 6.7 Hz, 3H).
^{13}C NMR (101 MHz, CDCl$_3$): δ 142.7, 106.4, 41.3, 39.9, 36.2, 36.0, 25.9.
HRMS (ESI): Calcd for $C_7H_{11}I^+$ [M]$^+$: 221.9905, found 221.9915.

10,14-Dimethyl-15-(6-methylheptan-2-yl)-2,3,4,5,8,9,10,11,12,13,14,15,16,17-tetradecahydro-1H-cyclopenta[a]phenanthren-3-yl 1-(iodomethyl)-3-methylenecyclobutane-1-carboxylate (S21)

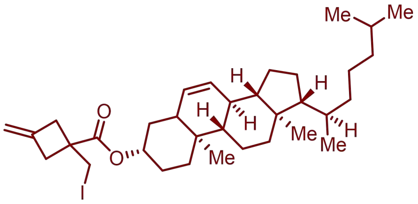

Synthesized according to **GP3**, colorless oil.

^{1}H NMR (400 MHz, CDCl$_3$): δ 5.43 – 5.33 (m, 1H), 4.96 – 4.90 (m, 2H), 4.73 – 4.60 (m, 1H), 3.56 (s, 2H), 3.24 – 3.14 (m, 2H), 2.65 – 2.52 (m, 2H), 2.40 – 2.30 (m, 2H), 2.05 – 1.93 (m, 3H), 1.91 – 1.79 (m, 3H), 1.60 – 1.44 (m, 7H), 1.37 – 1.30 (m, 4H), 1.27 – 1.23 (m, 2H), 1.17 – 1.06 (m, 8H), 1.02 (s, 3H), 0.91 (d, J = 6.5 Hz, 3H), 0.88 – 0.84 (m, 6H), 0.67 (s, 3H).
^{13}C NMR (101 MHz, CDCl$_3$): δ 172.6, 140.0, 139.3, 122.7, 108.9, 74.8, 56.6, 56.1, 49.9, 44.7, 42.2, 40.9, 39.6, 39.4, 37.9, 36.9, 36.5, 36.1, 35.7, 31.8, 31.8, 28.2, 27.9, 27.6, 24.2, 23.8, 22.8, 22.5, 21.0, 19.3, 18.7, 12.5, 11.8.
HRMS (ESI): Calcd for $C_{34}H_{54}IO_2^+$ [M+H]$^+$: 621.3163, found 621.3172.

Synthetic Procedure for 88

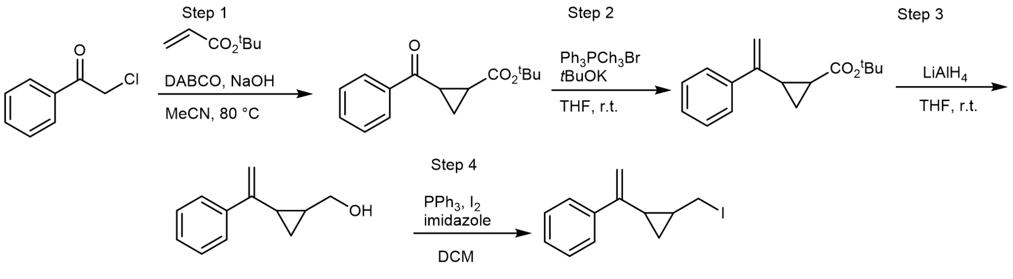

87

[Step 1]: To a solution of 2-chloro-1-phenylethan-1-one (1.0 equiv., 10 mmol) in MeCN was add Triethylenediamine (DABCO) (1.0 equiv.). The mixture was stirred for 30 min at room temperature, then added with NaOH (1.5 equiv.) and *tert*-butyl acrylate (1.0 equiv.). The reaction was stirred overnight at 80 °C, quenched by a

saturated solution of ammonium chloride, extracted with EA for 3 times, dried by Na_2SO_4, concentrated in vacuo and chromatographed (PE:EA = 10:1) to give *tert*-butyl 2-benzoylcyclop ropane-1-carboxylate (8.2 mmol, 82%) as an colorless oil.

[Step 2]: A mixture of Ph_3PCH_3Br (2.0 equiv.) and ^tBuOK (2.0 equiv.) was stirred in THF (0.5 M) at 0°C for 0.5 hours, Then *tert*-butyl 2-benzoylcyclopropane-1-carboxyl ate (1.0 equiv, 8.2 mmol) was added to the mixture. The resulting mixture was stirred at room temperature for 15 hours, then quenched with water, extracted with EA, dried by Na_2SO_4, concentrated in vacuo, and chromatographed (PE:EA = 10:1) to give *tert*-butyl 2-(1-phenylvinyl)cyclopro pane-1-carboxylate (7.3 mmol, 89%) as an colorless oil.

[Step 3]: To a solution of *tert*-butyl 2-(1-phenylvinyl)cyclopropane-1-carboxylate (1.0 equiv., 7.3 mmol) in THF was added $LiAlH_4$ (2.0 equiv.) portionwise at 0°C. The resulting mixture was stirred at room temperature for 6 hours. After the reaction was completed, the mixture was quenched by water and 15wt% NaOH (aq), filtered through a layer of silicone, dried over $MgSO_4$, concentrated and chromatographed (PE:EA = 2:1) to give (2-(1-phenylvinyl)cyclopropyl)methanol (4.9 mmol, 67%) as an colorless oil.

[Step 4]: To a N_2 protected solution of the PPh_3 (1.2 equiv.), imidazole (1.2 equiv.) and (2-(1-phenylvinyl)cyclopropyl)methanol (1.0 equiv., 4.9 mmol) in dry DCM (0.3 M) was added I_2 (1.5 equiv.) portionwise at 0°C. The reaction was stirred for 12 hours at room temperature. After the reaction was completed, the mixture was washed with a solution of $Na_2S_2O_3$, brine, dried over Na_2SO_4, filtered and concentrated under reduced pressure. (1-(2-(iodomethyl)cyclopropyl)vinyl)benzene **87** (2.2 mmol, 44%) was purified by flash chromatography (PE) as an colorless oil.

(1-(2-(iodomethyl)cyclopropyl)vinyl)benzene (87)

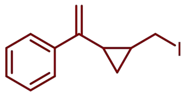

^{1}H NMR (500 MHz, $CDCl_3$): δ 7.62 – 7.52 (m, 2H), 7.41 – 7.37 (m, 2H), 7.35 – 7.31 (m, 1H), 5.34 (s, 1H), 5.03 – 4.98 (m, 1H), 3.50 – 3.42 (m, 1H), 3.23 – 3.17 (m, 1H), 1.79 – 1.70 (m, 1H), 1.61 – 1.53 (m, 1H), 1.21 – 1.14 (m, 1H), 0.90 – 0.82 (m, 1H).
^{13}C NMR (126 MHz, $CDCl_3$): δ 147.6, 140.9, 128.2, 127.6, 126.1, 110.1, 29.3, 25.2, 18.4, 11.9.
HRMS (ESI): Calcd for $C_{12}H_{14}I^+$ $[M+H]^+$: 285.0135, found 285.0143.

4-(iodomethyl)oxetan-2-one (88)

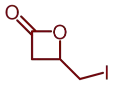

Synthesized according to reported literature[1], colorless oil.

^{1}H NMR (500 MHz, CDCl$_3$): δ 4.70 – 4.59 (m, 1H), 3.67 – 3.56 (m, 2H), 3.42 – 3.32 (m, 1H), 3.30 – 3.24 (m, 1H).

Synthetic Procedure for 89

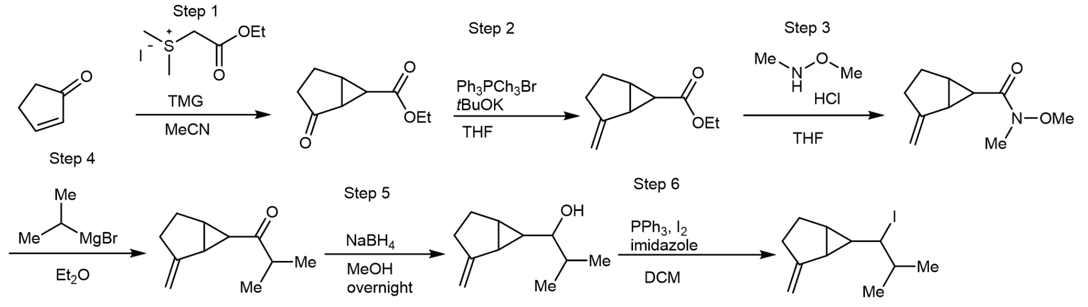

[Step 1]: To a rt suspension of (ethoxycarbonylmethyl)dimethylsulfonium bromide (1.2 equiv.) in MeCN (0.5 M) was added 1,1,3,3-tetramethylguanidine (TMG) (1.2 equiv.). The reaction mixture was stirred for 10 min at rt, cyclopent-2-enone (1.0 equiv., 10 mmol) was then added. The reaction mixture was stirred at room temperature overnight, then quenched with brine, dried by Na_2SO_4, concentrated in vacuo, and chromatographed (PE:EA = 10:1) to give ethyl 2-oxobicyclo[3.1.0] hexane-6-carboxylate (8.7 mmol, 87%) as an colorless oil.

[Step 2]: A mixture of Ph_3PCH_3Br (2.0 equiv.) and ^{t}BuOK (2.0 equiv.) was stirred in THF (0.5 M) at 0°C for 0.5 hours, Then ethyl 2-oxobicyclo[3.1.0] hexane-6-carboxylate (1.0 equiv, 8.7 mmol) was added to the mixture. The resulting mixture was stirred at room temperature for 15 hours, then quenched with water, extracted with EA, dried by Na_2SO_4, concentrated in vacuo, and chromatographed (PE:EA = 10:1) to give ethyl 2-methylenebicyclo[3.1.0]hexane -6-carboxylate (8.4 mmol, 96%) as an colorless oil.

[Step 3]: Stir a suspension of the ethyl 2-methylenebicyclo[3.1.0]hexane -6-carboxy late (1.0 equiv., 8.4 mmol) and N,O-dimethyl hydroxylamine hydrochloride (1.5 equiv.) in THF (1.0 M) at room temperature. Cool the mixture to 0°C, add isopropylmagnesium chloride (1.0 M in THF) (1.5 equiv.) to the mixture and stir for 30 min, then quenched by a saturated solution of ammonium chloride, extracted with EA for 3 times, dried by Na_2SO_4, concentrated in vacuo to give the *N*-methoxy-*N*-methyl-2-methylenebicyclo [3.1.0]hexane-6-carboxamide (6.5 mmol, 77%) as a yellow oil.

[Step 4]: To a N_2 protected suspension of *N*-methoxy-*N*-methyl-2-methylenebicyclo [3.1.0] hexane-6-carboxamide (1.0 equiv., 6.5 mmol) in Et_2O (1.0 M) was added isopropyl magnesium bromide (1.0 M in THF, 2.0 equiv.) slowly in 0 °C. The reaction mixture was stirred for 12 h at room temperature, and then quenched by a saturated solution of ammonium chloride, extracted with EA for 3 times, dried by Na2SO4, concentrated in vacuo and chromatographed (PE:EA = 10:1) to give

2-methyl-1-(2-methylenebicyclo
[3.1.0] hexan-6-yl)propan-1-one (6.0 mmol, 92%) as an colorless oil.

[Step 5]: To a solution of 2-methyl-1-(2-methylenebicyclo[3.1.0]hexan-6-yl)propan-1-one (1.0 equiv., 6.0 mmol) in MeOH was added $NaBH_4$ (2.0 equiv.) at rt. Then the resulting mixture was stirred at room temperature for 12 hours. After the reation was completed, The mixture was quenched by 1M HCl and extracted with ethyl acetate. The combined organic layer dried over Na_2SO_4, filtered and concentrated to give 2-methyl-1-(2-methylenebicyclo [3.1.0] hexan-6-yl)propan-1-ol (5.0 mmol, 83%) as an colorless oil.

[Step 6]: To a N_2 protected solution of the PPh_3 (1.2 equiv.), imidazole (1.2 equiv.) and 2-methyl-1-(2-methylenebicyclo[3.1.0] hexan-6-yl)propan-1-ol(1.0 equiv., 5.0 mmol) in dry DCM (0.3 M) was added I_2 (1.5 equiv.) portionwise at 0°C. The reaction was stirred for 12 hours at room temperature. After the reaction was completed, the mixture was washed with a solution of $Na_2S_2O_3$, brine, dried over Na_2SO_4, filtered and concentrated under reduced pressure. 6-(1-iodo-2-methylpropyl)-2-methylene bicyclo[3.1.0]hexane **89** (2.8 mmol,56%) was purified by flash chromatography (PE) as an colorless oil.

6-(1-iodo-2-methylpropyl)-2-methylenebicyclo[3.1.0]hexane (89)

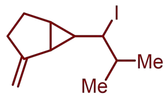

^{1}H NMR (400 MHz, CDCl$_3$): δ 5.73 – 5.66 (m, 1H), 5.42 – 5.34 (m, 1H), 5.30 – 5.23 (m, 1H), 4.00 (s, 2H), 3.25 – 3.13 (m, 1H), 2.48 – 2.41 (m, 2H), 2.25 – 2.16 (m, 2H), 1.67 – 1.59 (m, 1H), 0.97 (s, 3H), 0.95 (s, 3H).
^{13}C NMR (101 MHz, CDCl$_3$): δ 141.5, 136.8, 133.0, 129.5, 49.0, 33.6, 31.4, 30.8, 22.6, 5.0.
HRMS (ESI): Calcd for $C_{11}H_{18}I^+$ $[M+H]^+$: 277.0448, found 277.0459.

3.2 General procedure 5 for C(sp3)-N couplings (GP5)

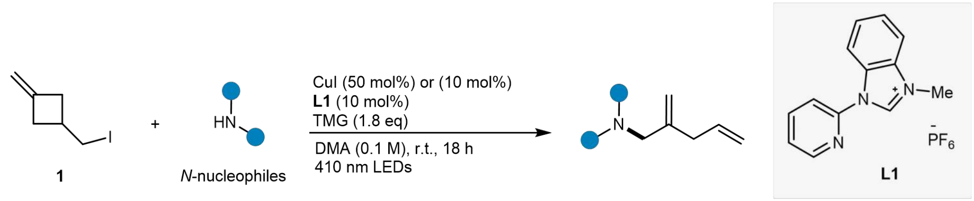

To an oven-dried 10 mL reaction vial were added CuI (9.5 mg, 0.05 mmol or 1.9 mg, 0.01 mmol), **L1** (3.6 mg, 0.01 mmol, 0.1 equiv.), 1,1,3,3-tetramethylguanidine (TMG) (23 μL, 0.18 mmol, 1.8 equiv.) and DMA (1 mL) in a nitrogen-filled glove box. The resulting mixture was stirred for 10 min, followed by adding *N*-nucleophiles (0.1 mmol, 1.0 equiv.) and alkyl iodides (0.15 mmol, 1.5 equiv.) in sequence, and sealed with a screwed cap. The sealed vial was placed on a photo-reactor under irradiation of LEDs (410 nm, 6 W). The mixture was stirred at room temperature for 18 h, quenched

with H_2O, and extracted with ethyl acetate. The combined organic layers were dried over anhydrous Na_2SO_4, concentrated in vacuo. The crude product was purified by silica gel column chromatography to afford the coupling product.

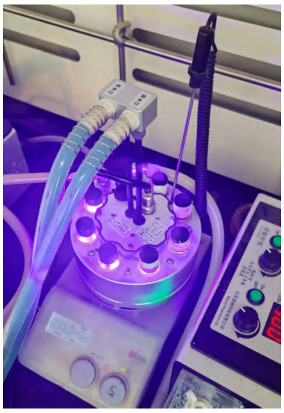

Supplementary Figure 1. The eight-position parallel light reaction system (RLH-18)

3.3 General procedure 6 for C(sp3)-N couplings (GP6)

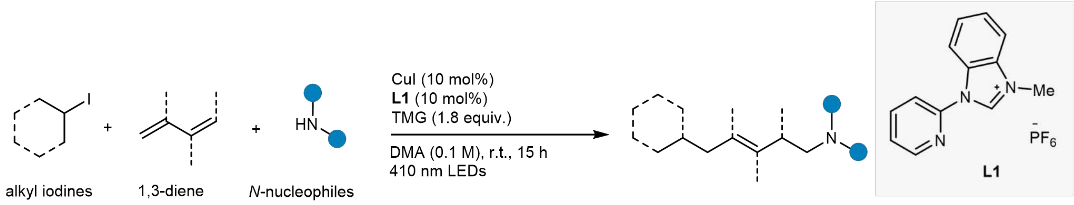

To an oven-dried 10 mL reaction vial were added CuI (1.9 mg, 0.01 mmol, 0.1 equiv.), **L1** (3.6 mg, 0.01 mmol, 0.1 equiv.), 1,1,3,3-tetramethylguanidine (TMG) (23 µL, 0.18 mmol, 1.8 equiv.) and DMA (1 mL) in a nitrogen-filled glove box. The resulting mixture was stirred for 10 min, followed by adding *N*-nucleophiles (0.1 mmol, 1.0 equiv.), alkyl iodides (0.15 mmol, 1.5 equiv.) and conjugated diene (0.3 mmol, 3.0 equiv.) in sequence, and sealed with a screwed cap. The sealed vial was placed on a photo-reactor under irradiation of LEDs (410 nm, 6 W). The mixture was stirred at room temperature for 18 h, quenched with H_2O, and extracted with ethyl acetate. The combined organic layers were dried over anhydrous Na_2SO_4, concentrated in vacuo. The crude product was purified by silica gel column chromatography to afford the product.

3.4 Scale up reaction

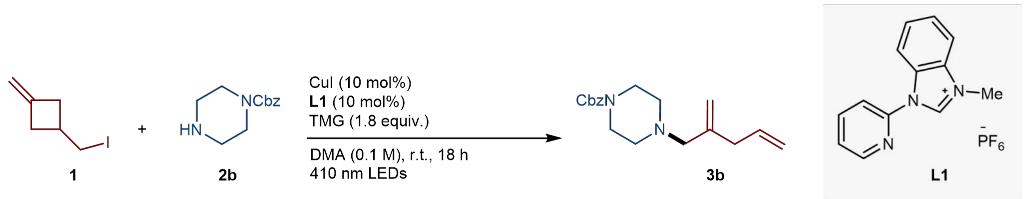

5 mmol scale for synthesis of 2b:

To an oven-dried 250 mL round-bottomed flask were added CuI (95 mg, 0.5 mmol, 0.1 equiv.), **L1** (180 mg, 0.5 mmol, 0.1 equiv.), 1,1,3,3-tetramethylguanidine (TMG)

(1.15 mL, 0.18 mmol, 1.8 equiv.) and DMA (50 mL) in a nitrogen-filled glove. The resulting mixture was stirred for 10 min, followed by adding benzyl piperazine-1-carboxylate (1.1 g, 5 mmol, 1.0 equiv.) and 1-(iodomethyl)-3-methylenecyclobutane (2.3 g, 0.15 mmol, 1.5 equiv.) in sequence, and sealed with a screwed cap. The sealed flask was placed on a large volume photo-reactor under irradiation of LEDs (410 nm, 50 W × 2). The mixture was stirred at 25 °C for 18 h, quenched with H_2O, and extracted with ethyl acetate. The combined organic layers were dried over anhydrous $MgSO_4$, concentrated in vacuo. The crude product was purified by silica gel column chromatography to afford the coupling product **2b** (1.38 g, 92% yield).

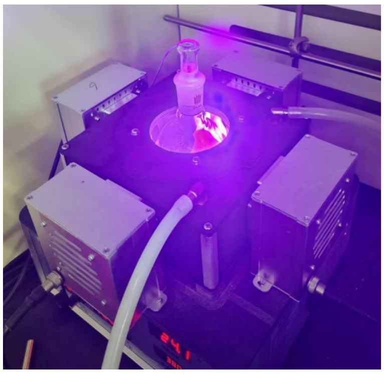

Supplementary Figure 2. The large volume light reaction system (RLH-054)

3.5 Characterization data of product

4-Methyl-*N*-(2-methylenepent-4-en-1-yl)aniline (3a)

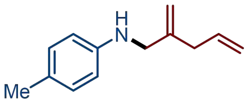

Prepared according to the **GP5** from *p*-toluidine (10.7 mg, 0.1 mmol) and 1-(iodomethyl)-3-methylenecyclobutane (31.2 mg, 0.15 mmol), The crude residue was purified by column chromatography to yield **3a** (15.9 mg, 85% yield) as a colorless oil.

^{1}H NMR (500 MHz, $CDCl_3$): δ 6.99 (d, *J* = 7.7 Hz, 2H), 6.55 (d, *J* = 7.8 Hz, 2H), 5.95 – 5.78 (m, 1H), 5.17 – 4.89 (m, 4H), 3.92 – 3.54 (m, 3H), 2.85 (d, *J* = 6.2 Hz, 2H), 2.25 (s, 3H).

^{13}C NMR (126 MHz, $CDCl_3$): δ 145.9, 145.2, 135.9, 129.6, 126.5, 116.5, 112.9, 111.2, 48.8, 38.8, 20.3.

HRMS (ESI): Calcd for $C_{13}H_{18}N^+$ $[M+H]^+$: 188.1434, found 188.1430.

Benzyl 4-(2-methylenepent-4-en-1-yl)piperazine-1-carboxylate (3b)

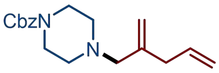

Prepared according to the **GP5** from benzyl piperazine-1-carboxylate (22.0 mg, 0.1 mmol) and 1-(iodomethyl)-3-methylenecyclobutane (31.2 mg, 0.15 mmol), The crude

residue was purified by column chromatography to yield **3b** (27.9 mg, 93% yield) as a colorless oil.

^{1}H NMR (400 MHz, CDCl$_3$): δ 7.55 – 7.29 (m, 5H), 6.02 – 5.71 (m, 1H), 5.14 (s, 2H), 5.11 – 4.81 (m, 4H), 3.62 – 3.44 (m, 4H), 2.89 (s, 2H), 2.81 (d, *J* = 6.8 Hz, 2H), 2.47 – 2.30 (m, 4H).
^{13}C NMR (101 MHz, CDCl$_3$): δ 155.2, 144.6, 136.8, 136.1, 128.4, 127.9, 127.8, 116.2, 113.3, 67.0, 63.7, 52.7, 43.9, 38.5.
HRMS (ESI): Calcd for $C_{18}H_{25}N_2O_2^+$ [M+H]$^+$: 301.1911, found 301.1915.

Enzyl (*E*)-4-(4-(oxetan-3-yl)but-2-en-1-yl)piperazine-1-carboxylate (6)

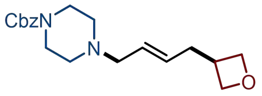

Prepared according to the **GP6** from benzyl piperazine-1-carboxylate (22.0 mg, 0.1 mmol) and 3-iodooxetane (27.6 mg, 0.15 mmol), buta-1,3-diene (150 µL, 0.3 mmol), The crude residue was purified by column chromatography to yield **6** (32.4 mg, 98% yield, 14:1 *E/Z,* > 20:1 rr) as a colorless oil.

^{1}H NMR (400 MHz, CDCl$_3$): δ 7.39 – 7.28 (m, 5H), 5.57 – 5.40 (m, 2H), 5.12 (s, 2H), 4.82 – 4.72 (m, 2H), 4.41 – 4.31 (m, 2H), 3.56 – 3.47 (m, 4H), 3.09 – 2.98 (m, 1H), 2.93 (d, *J* = 6.0 Hz, 2H), 2.48 – 2.34 (m, 6H).
^{13}C NMR (101 MHz, CDCl$_3$): δ 155.2, 136.7, 131.0, 128.4, 128.0, 127.8, 127.6, 76.9, 67.1, 60.7, 52.6, 43.7, 36.1, 34.1.
HRMS (ESI): Calcd for $C_{19}H_{27}N_2O_3^+$ [M+H]$^+$: 331.2016, found 331.2008.

4-Methoxy-*N*-(2-methylenepent-4-en-1-yl)aniline (7)

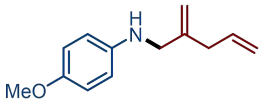

Prepared according to the **GP5** from 4-methoxyaniline (12.3 mg, 0.1 mmol) and 1-(iodomethyl)-3-methylenecyclobutane (31.2 mg, 0.15 mmol), The crude residue was purified by column chromatography to yield **7** (17.5 mg, 86% yield) as a colorless oil.

^{1}H NMR (400 MHz, CDCl$_3$): δ 6.77 (d, *J* = 8.9 Hz, 2H), 6.57 (d, *J* = 8.9 Hz, 2H), 5.93 – 5.79 (m, 1H), 5.15 – 4.91 (m, 4H), 3.74 (s, 3H), 3.67 (s, 2H), 2.84 (d, *J* = 6.8 Hz, 2H).
^{13}C NMR (101 MHz, CDCl$_3$): δ 152.0, 145.4, 142.4, 135.9, 116.6, 114.8, 114.0, 111.3, 55.8, 49.4, 38.8.
HRMS (ESI): Calcd for $C_{13}H_{18}NO^+$ [M+H]$^+$: 204.1383, found 204.1384.

4-Fluoro-*N*-(2-methylenepent-4-en-1-yl)aniline (8)

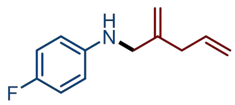

Prepared according to the **GP5** from 4-fluoroaniline (11.1 mg, 0.1 mmol) and 1-(iodomethyl)-3-methylenecyclobutane (31.2 mg, 0.15 mmol), The crude residue was purified by column chromatography to yield **8** (14.7 mg, 76% yield) as a colorless oil.

^{1}H NMR (400 MHz, CDCl$_3$): δ 6.87 (t, *J* = 8.7 Hz, 1H), 6.53 (dd, *J* = 8.9, 4.4 Hz, 1H), 5.99 – 5.74 (m, 1H), 5.21 – 4.90 (m, 3H), 3.87 – 3.60 (m, 2H), 2.83 (d, *J* = 6.8 Hz, 2H).
^{13}C NMR (101 MHz, CDCl$_3$): δ 155.7 (d, *J* = 234.7 Hz), 144.9, 144.5 (d, *J* = 1.3 Hz), 135.8, 116.7, 115.6 (d, *J* = 22.4 Hz), 113.6 (d, *J* = 7.3 Hz), 111.4, 49.1, 38.7.
^{19}F NMR (376 MHz, CDCl$_3$): δ -128.3 (1F).
HRMS (ESI): Calcd for $C_{12}H_{15}FN^{+}$ [M+H]$^{+}$: 192.1183, found 192.1179.

2-(4-((2-Methylenepent-4-en-1-yl)amino)phenyl)ethan-1-ol (9)

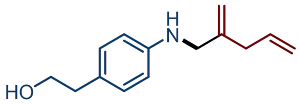

Prepared according to the **GP5** from 2-(4-aminophenyl)ethan-1-ol (13.7 mg, 0.1 mmol) and 1-(iodomethyl)-3-methylenecyclobutane (31.2 mg, 0.15 mmol), The crude residue was purified by column chromatography to yield **9** (16.5 mg, 76% yield) as a colorless oil.

^{1}H NMR (500 MHz, CDCl$_3$): δ 7.03 (d, *J* = 8.2 Hz, 2H), 6.57 (d, *J* = 8.2 Hz, 2H), 5.91 – 5.79 (m, 1H), 5.15 – 4.89 (m, 4H), 3.78 (t, *J* = 6.6 Hz, 2H), 3.70 (s, 2H), 2.84 (d, *J* = 6.8 Hz, 2H), 2.75 (t, *J* = 6.6 Hz, 2H).
^{13}C NMR (126 MHz, CDCl$_3$): δ 146.7, 145.0, 135.8, 129.7, 126.8, 116.6, 113.0, 111.3, 63.8, 48.6, 38.7, 38.2.
HRMS (ESI): Calcd for $C_{14}H_{20}NO^{+}$ [M+H]$^{+}$: 218.1539, found 218.1537.

3-((2-Methylenepent-4-en-1-yl)amino)phenol (10)

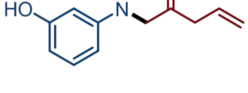

Prepared according to the **GP5** from 3-aminophenol (10.9 mg, 0.1 mmol) and 1-(iodomethyl)-3-methylenecyclobutane (31.2 mg, 0.15 mmol), The crude residue was purified by column chromatography to yield **10** (14.6 mg, 77% yield) as a colorless oil.

^{1}H NMR (400 MHz, CDCl$_3$): δ 7.04 – 6.96 (m, 1H), 6.24 – 6.14 (m, 2H), 6.11 – 6.03 (m, 1H), 5.94 – 5.76 (m, 1H), 5.20 – 4.89 (m, 4H), 4.62 (brs, 1H), 3.90 (brs, 1H), 3.69 (s, 2H), 2.83 (d, *J* = 6.9 Hz, 2H).

^{13}C NMR (101 MHz, CDCl$_3$): δ 156.6, 149.8, 144.8, 135.8, 130.1, 116.7, 111.4, 106.0, 104.2, 99.5, 48.4, 38.7.
HRMS (ESI): Calcd for $C_{12}H_{16}NO^+$ [M+H]$^+$: 190.1226, found 190.1222.

4-Fluoro-2-methoxy-*N*-(2-methylenepent-4-en-1-yl)aniline (11)

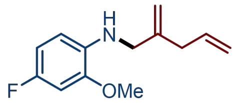

Prepared according to the **GP5** from 4-fluoro-2-methoxyaniline (14.1 mg, 0.1 mmol) and 1-(iodomethyl)-3-methylenecyclobutane (31.2 mg, 0.15 mmol), The crude residue was purified by column chromatography to yield **11** (19.9 mg, 90% yield) as a colorless oil.

^{1}H NMR (400 MHz, CDCl$_3$): δ 6.61 – 6.40 (m, 3H), 5.91 – 5.79 (m, 1H), 5.16 – 4.91 (m, 4H), 4.25 (s, 1H), 3.84 (s, 3H), 3.71 (s, 2H), 2.83 (d, *J* = 6.8 Hz, 2H).
^{13}C NMR (101 MHz, CDCl$_3$): δ 155.2 (d, *J* = 234.1 Hz), 147.1 (d, *J* = 9.4 Hz), 145.0, 135.8, 134.3 (d, *J* = 2.6 Hz), 116.6, 111.2, 109.7 (d, *J* = 8.8 Hz), 106.1 (d, *J* = 21.1 Hz), 98.4 (d, *J* = 27.5 Hz), 55.6, 48.7, 38.7.
^{19}F NMR (376 MHz, CDCl$_3$): δ -126.6 (1F).
HRMS (ESI): Calcd for $C_{13}H_{17}FNO^+$ [M+H]$^+$: 222.1289, found 222.1284.

***N*-Methyl-*N*-(2-methylenepent-4-en-1-yl)aniline (12)**

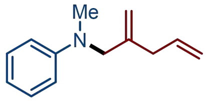

Prepared according to the **GP5** from *N*-methylaniline (10.7 mg, 0.1 mmol) and 1-(iodomethyl)-3-methylenecyclobutane (31.2 mg, 0.15 mmol), The crude residue was purified by column chromatography to yield **12** (12.2 mg, 65% yield) as a colorless oil.

^{1}H NMR (400 MHz, CDCl$_3$): δ 7.25 – 7.18 (m, 2H), 6.72 – 6.65 (m, 3H), 5.95 – 5.79 (m, 1H), 5.16 – 5.05 (m, 2H), 4.94 – 4.86 (m, 2H), 3.83 (s, 2H), 2.95 (s, 3H), 2.78 (d, *J* = 6.7 Hz, 2H).
^{13}C NMR (101 MHz, CDCl$_3$): δ 149.6, 143.6, 135.8, 129.0, 116.6, 116.1, 112.0, 111.0, 57.5, 38.2, 38.2.
HRMS (ESI): Calcd for $C_{13}H_{18}N^+$ [M+H]$^+$: 188.1434, found 188.1430.

4-(2-Methylenepent-4-en-1-yl)morpholine (13)

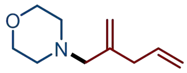

Prepared according to the **GP5** from morpholine (8.7 mg, 0.1 mmol) and 1-(iodomethyl)-3-methylenecyclobutane (31.2 mg, 0.15 mmol), The crude residue was purified by column chromatography to yield **13** (12.2 mg, 73% yield) as a colorless oil.

^{1}H NMR (400 MHz, CDCl$_3$): δ 5.97 – 5.66 (m, 1H), 5.18 – 4.83 (m, 4H), 3.79 – 3.60 (m, 4H), 2.87 (s, 2H), 2.81 (d, *J* = 6.9 Hz, 2H), 2.42 – 2.34 (m, 4H).
^{13}C NMR (101 MHz, CDCl$_3$): δ 144.6, 136.3, 116.2, 113.3, 67.1, 64.2, 53.6, 38.6.
HRMS (ESI): Calcd for $C_{10}H_{18}NO^+$ [M+H]$^+$: 168.1383, found 168.1381.

2-(2ethylenepent-4-en-1-yl)-1,2,3,4-tetrahydroisoquinoline (14)

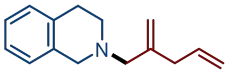

Prepared according to the **GP5** from 1,2,3,4-tetrahydroisoquinoline (13.3 mg, 0.1 mmol) and 1-(iodomethyl)-3-methylenecyclobutane (31.2 mg, 0.15 mmol), The crude residue was purified by column chromatography to yield **14** (20.5 mg, 96% yield) as a colorless oil.

^{1}H NMR (400 MHz, CDCl$_3$): δ 7.20 – 6.98 (m, 4H), 5.97 – 5.80 (m, 1H), 5.18 – 4.92 (m, 4H), 3.59 (s, 2H), 3.08 (s, 2H), 2.97 – 2.84 (m, 4H), 2.75 – 2.65 (m, 2H).
^{13}C NMR (101 MHz, CDCl$_3$): δ 145.2, 136.3, 135.2, 134.6, 128.6, 126.5, 126.0, 125.5, 116.1, 113.0, 63.6, 56.2, 50.5, 38.5, 29.2.
HRMS (ESI): Calcd for $C_{15}H_{20}N^+$ [M+H]$^+$: 214.1590, found 214.1586.

Ethyl (2-methylenepent-4-en-1-yl)prolinate (15)

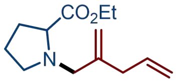

Prepared according to the **GP5** from ethyl prolinate (14.1 mg, 0.1 mmol) and 1-(iodomethyl)-3-methylenecyclobutane (31.2 mg, 0.15 mmol), The crude residue was purified by column chromatography to yield **15** (14.3 mg, 82% yield) as a colorless oil.

^{1}H NMR (400 MHz, CDCl$_3$): δ 5.94 – 5.73 (m, 1H), 5.12 – 4.78 (m, 4H), 4.15 (q, *J* = 7.1 Hz, 2H), 3.27 (d, *J* = 12.7 Hz, 1H), 3.17 – 3.12 (m, 1H), 3.08 – 3.02 (m, 1H), 2.95 (d, *J* = 12.8 Hz, 1H), 2.85 (d, *J* = 6.8 Hz, 2H), 2.36 – 2.27 (m, 1H), 2.17 – 2.02 (m, 1H), 1.97 – 1.73 (m, 3H), 1.26 (t, *J* = 7.1 Hz, 3H).
^{13}C NMR (101 MHz, CDCl$_3$): δ 174.3, 146.1, 136.4, 116.0, 112.6, 65.8, 60.4, 60.1, 53.5, 38.5, 29.4, 23.1, 14.2.
HRMS (ESI): Calcd for $C_{13}H_{22}NO_2^+$ [M+H]$^+$: 224.1645, found 224.1646.

***N*-Benzyl-*N*-methyl-2-methylenepent-4-en-1-amine (16)**

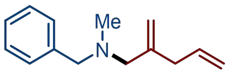

Prepared according to the **GP5** from *N*-methyl-1-phenylmethanamine (12.1 mg, 0.1 mmol) and 1-(iodomethyl)-3-methylenecyclobutane (31.2 mg, 0.15 mmol), The crude residue was purified by column chromatography to yield **16** (19.3 mg, 96% yield) as a colorless oil.

^{1}H NMR (400 MHz, CDCl$_3$): δ 7.46 – 7.18 (m, 5H), 5.90 – 5.78 (m, 1H), 5.10 – 4.88 (m, 4H), 3.46 (s, 2H), 2.93 (s, 2H), 2.87 (d, *J* = 6.9 Hz, 2H), 2.14 (s, 3H).
^{13}C NMR (101 MHz, CDCl$_3$): δ 146.1, 139.5, 136.4, 128.8, 128.1, 126.8, 116.1, 112.8, 63.1, 61.9, 42.2, 38.5.
HRMS (ESI): Calcd for $C_{14}H_{20}N^+$ $[M+H]^+$: 202.1590, found 202.1586.

Ethyl (2-methylenepent-4-en-1-yl)glycinate (17)

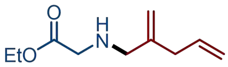

Prepared according to the **GP5** from ethyl glycinate (10.3 mg, 0.1 mmol) and 1-(iodomethyl)-3-methylenecyclobutane (31.2 mg, 0.15 mmol), The crude residue was purified by column chromatography to yield **17** (9.5 mg, 52% yield) as a colorless oil.

^{1}H NMR (400 MHz, CDCl$_3$): δ 5.92 – 5.72 (m, 1H), 5.16 – 4.84 (m, 4H), 4.19 (q, *J* = 7.1 Hz, 2H), 3.37 (s, 2H), 3.20 (s, 2H), 2.80 (d, *J* = 6.7 Hz, 2H), 1.27 (t, *J* = 7.2 Hz, 3H).
^{13}C NMR (101 MHz, CDCl$_3$): δ 172.5, 145.6, 136.0, 116.3, 111.7, 60.7, 53.6, 50.0, 38.8, 14.2.
HRMS (ESI): Calcd for $C_{10}H_{18}NO_2^+$ $[M+H]^+$: 184.1332, found 184.1328.

***N*-(2-Methylenepent-4-en-1-yl)adamantan-1-amine (18)**

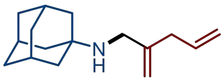

Prepared according to the **GP5** from adamantan-1-amine (15.1 mg, 0.1 mmol) and 1-(iodomethyl)-3-methylenecyclobutane (31.2 mg, 0.15 mmol), The crude residue was purified by column chromatography to yield **18** (22.2 mg, 96% yield) as a colorless oil.

^{1}H NMR (400 MHz, CDCl$_3$): δ 5.89 – 5.75 (m, 1H), 5.10 – 4.80 (m, 4H), 3.15 (s, 2H), 2.83 (d, *J* = 6.8 Hz, 2H), 2.06 (s, 3H), 1.70 – 1.56 (m, 12H).
^{13}C NMR (101 MHz, CDCl$_3$): δ 147.6, 136.3, 116.0, 110.1, 50.4, 45.0, 42.8, 39.5, 36.7, 29.6.
HRMS (ESI): Calcd for $C_{16}H_{26}N^+$ $[M+H]^+$: 232.2060, found 232.2066.

Ethyl 1-(4-methoxyphenyl)-6-(4-((2-methylenepent-4-en-1-yl)amino)phenyl) -7-oxo- 4,5,6,7-tetrahydro-1H-pyrazolo[3,4-c]pyridine-3-carboxylate (19)

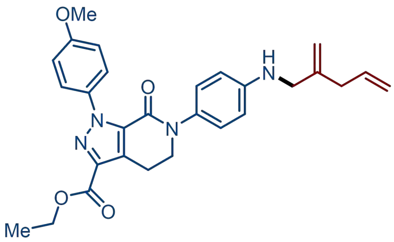

Prepared according to the **GP5** from ethyl 6-(4-aminophenyl)-1-(4-methoxyphenyl)

-7-oxo-4,5,6,7-tetrahydro-1H-pyrazolo[3,4-c]pyridine-3-carboxylate (40.6 mg, 0.1 mmol) and 1-(iodomethyl)-3-methylenecyclobutane (31.2 mg, 0.15 mmol), The crude residue was purified by column chromatography to yield **19** (42.7 mg, 88% yield,) as a colorless oil.

^{1}H NMR (400 MHz, CDCl$_3$): δ 7.48 (d, *J* = 8.7 Hz, 2H), 7.07 (d, *J* = 8.7 Hz, 2H), 6.89 (d, *J* = 8.6 Hz, 2H), 6.55 (d, *J* = 8.6 Hz, 2H), 5.90 – 5.75 (m, 1H), 5.14 – 5.04 (m, 2H), 5.01 (s, 1H), 4.91 (s, 1H), 4.45 (q, *J* = 7.1 Hz, 2H), 4.00 (t, *J* = 6.7 Hz, 2H), 3.79 (s, 3H), 3.68 (s, 2H), 3.27 (t, *J* = 6.7 Hz, 2H), 2.80 (d, *J* = 6.8 Hz, 2H), 1.42 (t, *J* = 7.1 Hz, 3H).
^{13}C NMR (101 MHz, CDCl$_3$): δ 162.2, 159.7, 157.3, 146.8, 144.6, 138.8, 135.7, 133.4, 132.6, 131.5, 127.0, 126.6, 126.4, 116.6, 113.5, 112.7, 111.4, 61.1, 55.4, 51.4, 48.4, 38.6, 21.5, 14.4.
HRMS (ESI): Calcd for $C_{28}H_{31}N_4O_4^+$ [M+H]$^+$: 487.2339 , found 487.2318.
8-(2-Methylenepent-4-en-1-yl)-8-azabicyclo[3.2.1]octan-3-ol (20)

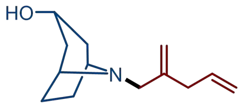

Prepared according to the **GP5** from 8-azabicyclo[3.2.1]octan-3-ol (12.7 mg, 0.1 mmol) and 1-(iodomethyl)-3-methylenecyclobutane (31.2 mg, 0.15 mmol), The crude residue was purified by column chromatography to yield **20** (16.6 mg, 80% yield) as a colorless oil.

^{1}H NMR (400 MHz, CDCl$_3$): δ 4.67 – 4.48 (m, 1H), 3.87 – 3.51 (m, 4H), 2.77 (t, *J* = 5.0 Hz, 1H), 1.84 (s, 2H), 1.62 (s, 2H), 1.57 (d, *J* = 6.8 Hz, 2H), 0.89 – 0.75 (m, 4H), 0.69 – 0.60 (m, 2H), 0.38 (d, *J* = 14.2 Hz, 2H).
^{13}C NMR (101 MHz, CDCl$_3$): δ 146.5, 136.6, 115.9, 111.4, 65.3, 58.1, 57.8, 40.0, 38.7, 26.1.
HRMS (ESI): Calcd for $C_{13}H_{22}NO^+$ [M+H]$^+$: 208.1696, found 208.1699.

8-Chloro-11-(1-(2-methylenepent-4-en-1-yl)piperidin-4-ylidene)-6,11-dihydro-5H-benzo[5,6]cyclohepta[1,2-b]pyridine (21)

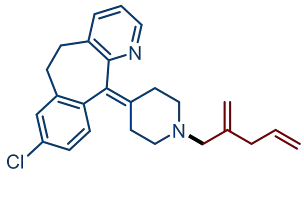

Prepared according to the **GP5** from 8-chloro-11-(piperidin-4-ylidene)-6,11-dihydro-5H-benzo[5,6]cyclohepta[1,2-b]pyridine (31.1 mg, 0.1 mmol) and 1-(iodomethyl)-3-methylenecyclobutane (31.2 mg, 0.15 mmol), The crude residue was purified by column chromatography to yield **21** (35.2 mg, 90% yield) as a colorless oil.

^{1}H NMR (400 MHz, CDCl$_3$): δ 8.39 (d, *J* = 3.8 Hz, 1H), 7.41 (d, *J* = 7.4 Hz, 1H), 7.16 – 6.98 (m, 4H), 5.95 – 5.74 (m, 1H), 5.13 – 4.82 (m, 4H), 3.49 – 3.26 (m, 2H), 2.85 (s, 2H), 2.83 – 2.62 (m, 6H), 2.54 – 2.23 (m, 4H), 2.11 – 1.96 (m, 2H).
^{13}C NMR (101 MHz, CDCl$_3$): δ 157.7, 146.6, 145.3, 139.5, 139.4, 137.9, 137.1, 136.3, 133.3, 132.5, 132.3, 130.9, 128.9, 125.9, 122.0, 116.0, 112.6, 63.6, 54.8, 54.8, 38.6, 31.8, 31.4, 31.0, 30.8.
HRMS (ESI): Calcd for $C_{25}H_{28}ClN_2^+$ [M+H]$^+$: 391.1936, found 391.1937.

11-(4-(2-Methylenepent-4-en-1-yl)piperazin-1-yl)dibenzo[b,f][1,4]thiazepine (22)

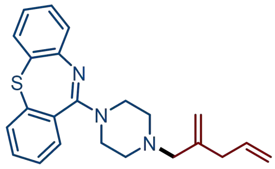

Prepared according to the **GP5** from 4-fluoro-2-methoxyaniline (29.5 mg, 0.1 mmol) and 1-(iodomethyl)-3-methylenecyclobutane (31.2 mg, 0.15 mmol), The crude residue was purified by column chromatography to yield **22** (33.0 mg, 88% yield) as a colorless oil.

^{1}H NMR (400 MHz, CDCl$_3$): δ 7.51 (d, *J* = 7.4 Hz, 1H), 7.39 (d, *J* = 7.7 Hz, 1H), 7.34 – 7.27 (m, 3H), 7.21 – 7.14 (m, 1H), 7.08 (d, *J* = 7.9 Hz, 1H), 6.94 – 6.84 (m, 1H), 5.93 – 5.76 (m, 1H), 5.19 – 4.77 (m, 4H), 3.58 – 3.43 (m, 4H), 2.94 (s, 2H), 2.83 (d, *J* = 6.9 Hz, 2H), 2.61 – 2.34 (m, 4H).
^{13}C NMR (101 MHz, CDCl$_3$): δ 160.9, 149.0, 144.7, 139.9, 136.2, 134.2, 132.1, 132.1, 130.7, 129.1, 129.0, 128.2, 128.0, 125.3, 122.7, 116.1, 113.3, 63.8, 52.9, 47.0, 38.6.
HRMS (ESI): Calcd for $C_{23}H_{26}N_3S^+$ [M+H]$^+$: 376.1842, found 376.1834.

Furan-2-yl(4-(2-methylenepent-4-en-1-yl)piperazin-1-yl)methanone (23)

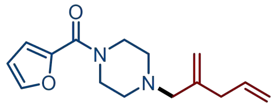

Prepared according to the **GP5** from furan-2-yl(piperazin-1-yl)methanone (18.0 mg, 0.1 mmol) and 1-(iodomethyl)-3-methylenecyclobutane (31.2 mg, 0.15 mmol), The crude residue was purified by column chromatography to yield **23** (17.6 mg, 85% yield) as a colorless oil.

^{1}H NMR (400 MHz, CDCl$_3$): δ 7.46 (s, 1H), 6.96 (d, *J* = 3.4 Hz, 1H), 6.46 (dd, *J* = 3.2, 1.6 Hz, 2H), 5.96 – 5.71 (m, 2H), 5.19 – 4.86 (m, 4H), 3.78 (s, 4H), 2.90 (s, 2H), 2.82 (d, *J* = 6.9 Hz, 2H), 2.50 – 2.31 (m, 4H).
^{13}C NMR (101 MHz, CDCl$_3$): δ 158.7, 147.6, 144.1, 143.2, 135.8, 115.9, 115.8, 113.1, 110.8, 63.2, 52.8, 44.5, 38.2.
HRMS (ESI): Calcd for $C_{15}H_{21}N_2O_2^+$ [M+H]$^+$: 261.1598, found 261.1692.

***N*-(2-(1H-indol-3-yl)ethyl)-2-methylenepent-4-en-1-amine (24)**

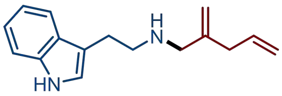

Prepared according to the modified **GP5** from 2-(1H-indol-3-yl)ethan-1-amine (32.0 mg, 0.2 mmol) and 1-(iodomethyl)-3-methylenecyclobutane (31.2 mg, 0.10 mmol), The crude residue was purified by column chromatography to yield **24** (9.6 mg, 40% yield) as a colorless oil.

^{1}H NMR (500 MHz, CDCl$_3$): δ 8.14 (s, 1H), 7.62 (d, *J* = 7.6 Hz, 1H), 7.37 (d, *J* = 7.9 Hz, 1H), 7.23 – 7.17 (m, 2H), 7.13 (d, *J* = 7.0 Hz, 2H), 5.68 – 5.44 (m, 1H), 5.06 – 4.66 (m, 4H), 3.26 (s, 2H), 3.07 – 2.91 (m, 4H), 2.72 (d, *J* = 6.2 Hz, 3H), 2.37 (s, 1H).
^{13}C NMR (126 MHz, CDCl$_3$): δ 145.0, 136.4, 136.4, 127.3, 122.4, 122.1, 119.3, 118.8, 113.4, 112.3, 111.2, 111.2, 54.1, 49.1, 39.2, 25.4.
HRMS (ESI): Calcd for $C_{16}H_{21}N_2^+$ $[M+H]^+$: 241.1699, found 241.1700.

Benzyl 4-(4-benzyl-2-methylenepent-4-en-1-yl)piperazine-1-carboxylate (25)

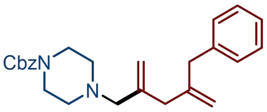

Prepared according to the **GP5** from benzyl piperazine-1-carboxylate (22.0 mg, 0.1 mmol) and ((1-(iodomethyl)-3-methylenecyclobutyl)methyl)benzene (44.7 mg, 0.15 mmol), The crude residue was purified by column chromatography to yield **25** (38.2 mg, 98% yield) as a colorless oil.

^{1}H NMR (400 MHz, CDCl$_3$): δ 7.41 – 7.26 (m, 7H), 7.24 – 7.16 (m, 3H), 5.15 (s, 2H), 5.04 (s, 1H), 4.94 (s, 1H), 4.91 (s, 1H), 4.85 (s, 1H), 3.47 (t, *J* = 5.1 Hz, 4H), 3.33 (s, 2H), 2.85 (s, 2H), 2.76 (s, 2H), 2.36 – 2.29 (m, 4H).
^{13}C NMR (101 MHz, CDCl$_3$): δ 155.2, 146.3, 143.5, 139.6, 136.7, 129.0, 128.4, 128.2, 127.9, 127.8, 126.0, 114.5, 113.6, 66.6, 62.8, 52.7, 43.9, 42.2, 40.4.
HRMS (ESI): Calcd for $C_{25}H_{31}N_2O_2^+$ $[M+H]^+$: 391.2380, found 391.2385.

Benzyl
4-(4-(2-methylbenzyl)-2-methylenepent-4-en-1-yl)piperazine-1-carboxylate (26)

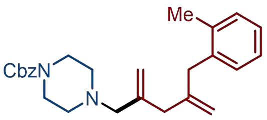

Prepared according to the **GP5** from benzyl piperazine-1-carboxylate (22.0 mg, 0.1 mmol) and 1-((1-(iodomethyl)-3-methylenecyclobutyl)methyl)-2-methylbenzene (46.8 mg, 0.15 mmol), The crude residue was purified by column chromatography to yield **26** (37.6 mg, 93% yield) as a colorless oil.

^{1}H NMR (400 MHz, CDCl$_3$): δ 7.41 – 7.30 (m, 5H), 7.18 – 7.08 (m, 4H), 5.15 (s, 2H), 5.06 (s, 1H), 4.98 (s, 1H), 4.90 (s, 1H), 4.60 (s, 1H), 3.53 – 3.46 (m, 4H), 3.30 (s, 2H), 2.88 (s, 2H), 2.83 (s, 2H), 2.35 (s, 4H), 2.26 (s, 3H).

^{13}C NMR (101 MHz, CDCl$_3$): δ 155.2, 145.3, 143.6, 137.6, 136.8, 136.7, 130.1, 130.0, 128.4, 127.9, 127.8, 126.3, 125.8, 114.4, 113.2, 66.3, 62.9, 52.7, 43.9, 41.3, 39.5, 19.3.
HRMS (ESI): Calcd for $C_{26}H_{33}N_2O_2^+$ [M+H]$^+$: 405.2537, found 405.2539.

Benzyl 4-(2-methylene-4-(2-(trifluoromethyl)benzyl)pent-4-en-1-yl)piperazine-1-carboxylate (27)

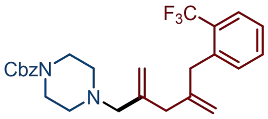

Prepared according to the **GP5** from benzyl piperazine-1-carboxylate (22.0 mg, 0.1 mmol) and 1-((1-(iodomethyl)-3-methylenecyclobutyl)methyl)-2-(trifluoromethyl)benzene (54.9 mg, 0.15 mmol), The crude residue was purified by column chromatography to yield **27** (44.9 mg, 98% yield) as a colorless oil.

^{1}H NMR (400 MHz, CDCl$_3$): δ 7.63 (d, J = 7.8 Hz, 1H), 7.52 – 7.44 (m, 1H), 7.40 – 7.28 (m, 7H), 5.13 (s, 2H), 5.04 (s, 1H), 4.96 (d, J = 8.6 Hz, 2H), 4.59 (s, 1H), 3.53 – 3.43 (m, 6H), 2.88 – 2.81 (m, 4H), 2.36 – 2.30 (m, 4H).
^{13}C NMR (101 MHz, CDCl$_3$): δ 155.2, 145.4, 143.3, 138.0, 136.8, 131.6, 131.5, 129.0 (q, J = 30.3 Hz), 128.4, 127.9, 127.8, 126.2, 125.8 (q, J = 5.6 Hz), 124.5 (q, J = 272.7 Hz), 114.7, 114.4, 67.0, 62.8, 52.7, 43.9, 41.3, 38.3.
^{19}F NMR (376 MHz, CDCl$_3$): δ -59.8 (3F).
HRMS (ESI): Calcd for $C_{26}H_{30}F_3N_2O_2^+$ [M+H]$^+$: 459.2254, found 459.2250.

Benzyl 4-(4-(3-fluorobenzyl)-2-methylenepent-4-en-1-yl)piperazine-1-carboxylate (28)

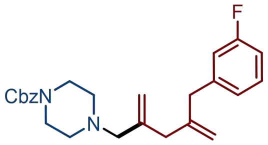

Prepared according to the **GP5** from benzyl piperazine-1-carboxylate (22.0 mg, 0.1 mmol) and 1-fluoro-3-((1-(iodomethyl)-3-methylenecyclobutyl)methyl)benzene (47.3 mg, 0.15 mmol), The crude residue was purified by column chromatography to yield **28** (40.0 mg, 98% yield) as a colorless oil.

^{1}H NMR (400 MHz, CDCl$_3$): δ 7.42 – 7.18 (m, 6H), 6.98 – 6.86 (m, 3H), 5.14 (s, 2H), 5.04 (s, 1H), 4.92 (s, 2H), 4.86 (s, 1H), 3.49 – 3.45 (m, 4H), 3.31 (s, 2H), 2.84 (s, 2H), 2.74 (s, 2H), 2.35 – 2.28 (m, 4H).
^{13}C NMR (101 MHz, CDCl$_3$): δ 162.8 (d, J = 245.0 Hz), 155.2, 145.6, 143.4, 142.2 (d, J = 7.2 Hz), 136.7, 129.6 (d, J = 8.6 Hz), 128.4, 127.9, 127.8, 124.7 (d, J = 2.9 Hz), 115.8 (d, J = 21.0 Hz), 114.4 (d, J = 53.1 Hz), 112.9 (d, J = 21.0 Hz), 67.0, 62.8, 52.7, 43.9, 41.8, 40.3.
^{19}F NMR (376 MHz, CDCl$_3$): δ -113.8 (1F).

HRMS (ESI): Calcd for $C_{25}H_{30}FN_2O_2^+$ $[M+H]^+$: 409.2286, found 409.2289.

Benzyl 4-(2-methylene-4-(3-(trifluoromethyl)benzyl)pent-4-en-1-yl)piperazine-1-carboxylate (29)

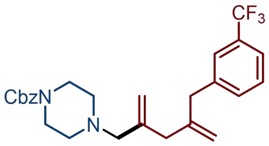

Prepared according to the **GP5** from benzyl piperazine-1-carboxylate (22.0 mg, 0.1 mmol) and 1-((1-(iodomethyl)-3-methylenecyclobutyl)methyl)-3-(trifluoromethyl)benzene (36.6 mg, 0.15 mmol), The crude residue was purified by column chromatography to yield **29** (44.9 mg, 98% yield) as a colorless oil.

^{1}H NMR (400 MHz, CDCl$_3$): δ 7.54 – 7.29 (m, 9H), 5.14 (s, 2H), 5.04 (s, 1H), 4.94 (s, 1H), 4.91 (s, 1H), 4.85 (s, 1H), 3.49 – 3.44 (m, 4H), 3.37 (s, 2H), 2.83 (s, 2H), 2.74 (s, 2H), 2.37 – 2.26 (m, 4H).
^{13}C NMR (101 MHz, CDCl$_3$): δ 155.2, 145.4, 143.3, 140.5, 136.8, 132.5, 130.5 (q, J = 32.0 Hz), 128.6, 128.5, 127.9, 127.8, 125.7 (q, J = 3.7 Hz), 124.5 (q, J = 272.7 Hz), 123.0 (q, J = 4.0 Hz), 114.8, 114.4, 67.0, 62.8, 52.7, 43.9, 41.9, 40.4.
^{19}F NMR (376 MHz, CDCl$_3$): δ -62.5 (3F).
HRMS (ESI): Calcd for $C_{26}H_{30}F_3N_2O_2^+$ $[M+H]^+$: 459.2254, found 459.2256.

Benzyl 4-(4-(4-(*tert*-butyl)benzyl)-2-methylenepent-4-en-1-yl)piperazine-1-carboxylate (30)

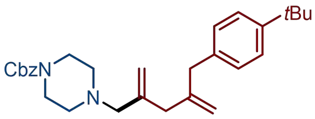

Prepared according to the **GP5** from benzyl piperazine-1-carboxylate (22.0 mg, 0.1 mmol) and 1-(*tert*-butyl)-4-((1-(iodomethyl)-3-methylenecyclobutyl)methyl)benzene (35.4 mg, 0.15 mmol), The crude residue was purified by column chromatography to yield **30** (42.4 mg, 95% yield) as a colorless oil.

^{1}H NMR (400 MHz, CDCl$_3$): δ 7.42 – 7.29 (m, 7H), 7.12 (d, J = 8.1 Hz, 2H), 5.15 (s, 2H), 5.04 (s, 1H), 4.95 (s, 1H), 4.90 – 4.85 (m, 2H), 3.51 – 3.44 (m, 4H), 3.30 (s, 2H), 2.85 (s, 2H), 2.76 (s, 2H), 2.36 – 2.28 (m, 4H), 1.32 (s, 9H).
^{13}C NMR (101 MHz, CDCl$_3$): δ 155.2, 148.8, 146.5, 143.6, 136.8, 136.5, 128.7, 128.4, 127.9, 127.8, 125.1, 114.4, 113.4, 67.0, 62.9, 52.7, 43.9, 41.6, 40.4, 34.3, 31.4.
HRMS (ESI): Calcd for $C_{29}H_{39}N_2O_2^+$ $[M+H]^+$: 447.3006, found 447.3011.

Benzyl 4-(2-methylene-4-(4-(trifluoromethyl)benzyl)pent-4-en-1-yl)piperazine-1-carboxylate (31)

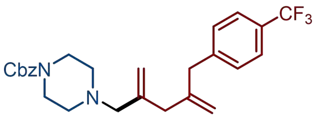

Prepared according to the **GP5** from benzyl piperazine-1-carboxylate (22.0 mg, 0.1 mmol) and 1-((1-(iodomethyl)-3-methylenecyclobutyl)methyl)-4-(trifluoromethyl)benzene (54.9 mg, 0.15 mmol), The crude residue was purified by column chromatography to yield **31** (40.8 mg, 89% yield) as a colorless oil.

^{1}H NMR (400 MHz, $CDCl_3$): δ 7.54 (d, *J* = 8.0 Hz, 2H), 7.39 – 7.25 (m, 7H), 5.13 (s, 2H), 5.04 (s, 1H), 4.95 (s, 1H), 4.92 (s, 1H), 4.83 (s, 1H), 3.51 – 3.44 (m, 4H), 3.36 (s, 2H), 2.83 (s, 2H), 2.74 (s, 2H), 2.32 (s, 4H).
^{13}C NMR (101 MHz, $CDCl_3$): δ 155.2, 145.4, 143.7, 143.3, 136.7, 129.4, 128.5, 128.4 (q, *J* = 30.3 Hz), 128.0, 127.8, 125.2 (q, *J* = 3.9 Hz), 124.2 (q, *J* = 272.7 Hz) 114.8, 114.4, 67.0, 62.8, 52.7, 43.9, 41.9, 40.5.
^{19}F NMR (376 MHz, $CDCl_3$): δ -62.3 (3F).
HRMS (ESI): Calcd for $C_{26}H_{30}F_3N_2O_2^+$ $[M+H]^+$: 459.2254, found 459.2256.

Benzyl 4-(4-(4-fluorobenzyl)-2-methylenepent-4-en-1-yl)piperazine-1-carboxylate (32)

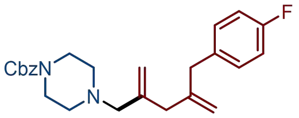

Prepared according to the **GP5** from benzyl piperazine-1-carboxylate (22.0 mg, 0.1 mmol) and 1-fluoro-4-((1-(iodomethyl)-3-methylenecyclobutyl)methyl)benzene (47.4 mg, 0.15 mmol), The crude residue was purified by column chromatography to yield **32** (40.0 mg, 98% yield) as a colorless oil.

^{1}H NMR (400 MHz, $CDCl_3$): δ 7.39 – 7.29 (m, 5H), 7.15 – 7.09 (m, 2H), 7.01 – 6.93 (m, 2H), 5.13 (s, 2H), 5.03 (s, 1H), 4.93 – 4.88 (m, 2H), 4.82 (s, 1H), 3.49 – 3.45 (m, 4H), 3.28 (s, 2H), 2.83 (s, 2H), 2.73 (s, 2H), 2.36 – 2.27 (m, 4H).
^{13}C NMR (101 MHz, $CDCl_3$): δ 161.5 (d, *J* = 244.0 Hz), 155.3, 146.3, 143.5, 136.8, 135.2 (d, *J* = 3.3 Hz), 130.5 (d, *J* = 7.8 Hz), 128.5, 128.0, 127.9, 115.0 (d, *J* = 21.1 Hz), 114.6, 113.8, 67.1, 62.9, 52.8, 43.9, 41.3, 40.5.
^{19}F NMR (376 MHz, $CDCl_3$): δ -117.3 (1F).
HRMS (ESI): Calcd for $C_{25}H_{30}FN_2O_2^+$ $[M+H]^+$: 409.2286, found 409.2288.

Benzyl 4-(4-(3,4-dichlorobenzyl)-2-methylenepent-4-en-1-yl)piperazine-1-carboxylate (33)

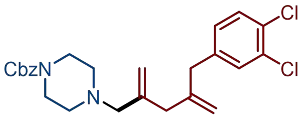

Prepared according to the **GP5** from benzyl piperazine-1-carboxylate (22.0 mg, 0.1 mmol) and 1,2-dichloro-4-((1-(iodomethyl)-3-methylenecyclobutyl)methyl)benzene

(55.1 mg, 0.15 mmol), The crude residue was purified by column chromatography to yield **33** (39.9 mg, 87% yield) as a colorless oil.

^{1}H NMR (400 MHz, CDCl$_3$): δ 7.40 – 7.30 (m, 6H), 7.26 (s, 1H), 7.01 (dd, *J* = 8.2, 2.1 Hz, 1H), 5.14 (s, 2H), 5.04 (s, 1H), 4.95 – 4.90 (m, 2H), 4.85 (s, 1H), 3.51 – 3.45 (m, 4H), 3.26 (s, 2H), 2.83 (s, 2H), 2.72 (s, 2H), 2.38 – 2.27 (m, 4H).
^{13}C NMR (101 MHz, CDCl$_3$): δ 155.2, 145.2, 143.2, 139.9, 136.7, 132.1, 130.9, 130.1, 130.0, 128.5, 128.4, 127.9, 127.8, 114.8, 114.5, 67.0, 62.9, 52.7, 43.9, 41.2, 40.3.
HRMS (ESI): Calcd for $C_{25}H_{29}Cl_2N_2O_2^+$ [M+H]$^+$: 459.1601, found 459.1602.

Benzyl 4-(2,4-dimethylene-5-phenylhexyl)piperazine-1-carboxylate (34)

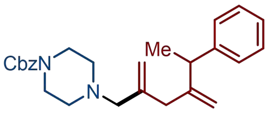

Prepared according to the **GP5** from benzyl piperazine-1-carboxylate (22.0 mg, 0.1 mmol) and (1-(1-(iodomethyl)-3-methylenecyclobutyl)ethyl)benzene (46.8 mg, 0.15 mmol), The crude residue was purified by column chromatography to yield **34** (31.6 mg, 78% yield) as a colorless oil.

^{1}H NMR (400 MHz, CDCl$_3$): δ 7.41 – 7.25 (m, 7H), 7.23 – 7.15 (m, 3H), 5.13 (s, 2H), 5.07 – 4.82 (m, 4H), 3.49 – 3.39 (m, 5H), 2.86 – 2.80 (m, 1H), 2.77 – 2.70 (m, 2H), 2.63 – 2.56 (m, 1H), 2.35 – 2.23 (m, 4H), 1.36 (d, *J* = 7.1 Hz, 3H).
^{13}C NMR (101 MHz, CDCl$_3$): δ 155.2, 150.3, 145.2, 143.9, 136.8, 128.4, 128.3, 127.9, 127.8, 127.6, 126.1, 114.5, 111.0, 67.0, 62.7, 52.6, 44.4, 43.9, 40.3, 20.7.
HRMS (ESI): Calcd for $C_{26}H_{33}N_2O_2^+$ [M+H]$^+$: 405.2537, found 405.2537.

Benzyl 4-(6-methoxy-2,4-dimethylenehexyl)piperazine-1-carboxylate (35)

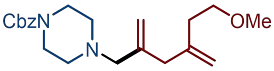

Prepared according to the **GP5** from benzyl piperazine-1-carboxylate (22.0 mg, 0.1 mmol) and 1-(iodomethyl)-1-(2-methoxyethyl)-3-methylenecyclobutane (39.9 mg, 0.15 mmol), The crude residue was purified by column chromatography to yield **35** (35.1 mg, 98% yield) as a colorless oil.

^{1}H NMR (400 MHz, CDCl$_3$): δ 7.40 – 7.27 (m, 5H), 5.13 (s, 2H), 5.00 (s, 1H), 4.92 (s, 1H), 4.87 (s, 2H), 3.53 – 3.46 (m, 6H), 3.33 (s, 3H), 2.85 – 2.80 (m, 4H), 2.39 – 2.24 (m, 6H).
^{13}C NMR (101 MHz, CDCl$_3$): δ 155.2, 143.8, 143.5, 136.7, 128.4, 127.9, 127.8, 114.4, 112.9, 71.1, 67.0, 62.8, 58.5, 52.7, 43.9, 41.4, 35.3.
HRMS (ESI): Calcd for $C_{21}H_{31}N_2O_3^+$ [M+H]$^+$: 359.2329, found 259.2319.

Benzyl 4-(2,4-dimethylenehept-6-en-1-yl)piperazine-1-carboxylate (36)

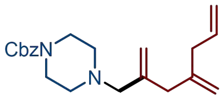

Prepared according to the **GP5** from benzyl piperazine-1-carboxylate (22.0 mg, 0.1 mmol) and 1-allyl-1-(iodomethyl)-3-methylenecyclobutane (37.2 mg, 0.15 mmol), The crude residue was purified by column chromatography to yield **36** (24.9 mg, 73% yield) as a colorless oil.

^{1}H NMR (400 MHz, CDCl$_3$): δ 7.40 – 7.29 (m, 5H), 5.88 – 5.73 (m, 1H), 5.13 (s, 2H), 5.07 – 5.00 (m, 3H), 4.92 (s, 1H), 4.87 – 4.82 (m, 2H), 3.53 – 3.47 (m, 4H), 2.85 – 2.79 (m, 4H), 2.73 (d, J = 6.9 Hz, 2H), 2.39 – 2.29 (m, 4H). ^{13}C NMR (101 MHz, CDCl$_3$) δ 155.3, 145.5, 143.6, 136.8, 136.3, 128.5, 127.9, 127.8, 116.2, 114.3, 112.5, 67.0, 62.9, 52.7, 43.9, 41.1, 40.0.
^{13}C NMR (101 MHz, CDCl$_3$): δ 155.3, 145.5, 143.6, 136.8, 136.3, 128.5, 127.9, 127.8, 116.2, 114.3, 112.5, 67.0, 62.9, 52.7, 43.9, 41.1, 40.0.
HRMS (ESI): Calcd for $C_{21}H_{29}N_2O_2^+$ [M+H]$^+$: 341.2224, found 341.2222.

Benzyl 4-(2,4-dimethyleneoct-6-yn-1-yl)piperazine-1-carboxylate (37)

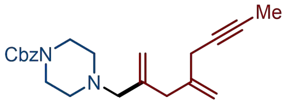

Prepared according to the **GP5** from benzyl piperazine-1-carboxylate (22.0 mg, 0.1 mmol) and 1-(iodomethyl)-3-methylene-1-(prop-2-yn-1-yl)cyclobutane (36.9 mg, 0.15 mmol), The crude residue was purified by column chromatography to yield **37** (14.6 mg, 45% yield) as a colorless oil.

^{1}H NMR (500 MHz, CDCl$_3$): δ 7.39 – 7.28 (m, 5H), 5.17 (s, 1H), 5.13 (s, 2H), 5.01 (s, 1H), 4.95 – 4.78 (m, 2H), 3.56 – 3.44 (m, 4H), 2.89 – 2.80 (m, 5H), 2.49 – 2.26 (m, 5H), 1.82 (s, 3H).
^{13}C NMR (126 MHz, CDCl$_3$): δ 155.2, 143.3, 142.5, 136.8, 128.5, 128.0, 127.8, 114.6, 112.9, 78.2, 76.1, 67.0, 62.8, 52.7, 43.9, 40.9, 25.4, 3.5.
HRMS (ESI): Calcd for $C_{22}H_{29}N_2O_2^+$ [M+H]$^+$: 353.2224, found 353.2219.

Benzyl 4-(2-methylene-4-(*p*-tolyl)pent-4-en-1-yl)piperazine-1-carboxylate (38)

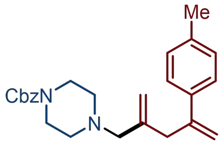

Prepared according to the **GP5** from benzyl piperazine-1-carboxylate (22.0 mg, 0.1 mmol) and (1-(iodomethyl)-3-methylenecyclobutyl)benzene (42.6 mg, 0.15 mmol), The crude residue was purified by column chromatography to yield **38** (25.0 mg, 64% yield) as a colorless oil.

^{1}H NMR (400 MHz, $CDCl_3$): δ 7.40 – 7.30 (m, 7H), 7.11 (d, *J* = 8.0 Hz, 2H), 5.42 (d, *J* = 1.6 Hz, 1H), 5.14 (s, 2H), 5.08 (s, 1H), 4.96 (s, 1H), 4.91 (s, 1H), 3.55 – 3.47 (m, 4H), 3.28 (s, 2H), 2.86 (s, 2H), 2.37 – 2.29 (m, 7H).
^{13}C NMR (101 MHz, $CDCl_3$): δ 155.3, 145.4, 143.9, 138.0, 137.1, 136.8, 128.8, 128.5, 128.0, 127.8, 126.0, 114.8, 113.8, 67.0, 63.5, 52.7, 43.9, 40.0, 21.1.
HRMS (ESI): Calcd for $C_{25}H_{31}N_2O_2^+$ [M+H]$^+$: 391.2380, found 391.2385.

Benzyl 4-(4-cyano-2-methylenepent-4-en-1-yl)piperazine-1-carboxylate (39)

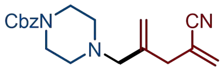

Prepared according to the **GP5** from benzyl piperazine-1-carboxylate (22.0 mg, 0.1 mmol) and 1-(iodomethyl)-3-methylenecyclobutane-1-carbonitrile (35.0 mg, 0.15 mmol), The crude residue was purified by column chromatography to yield **39** (16.9 mg, 52% yield) as a colorless oil.

^{1}H NMR (400 MHz, $CDCl_3$): δ 7.40 – 7.29 (m, 5H), 5.93 (s, 1H), 5.78 (s, 1H), 5.14 – 5.12 (m, 3H), 5.06 (s, 1H), 3.54 – 3.46 (m, 4H), 3.03 (s, 2H), 2.90 (s, 2H), 2.35 – 2.32 (m, 4H).
^{13}C NMR (101 MHz, $CDCl_3$): δ 155.2, 140.8, 136.7, 131.8, 128.5, 128.0, 128.0, 127.8, 121.2, 116.8, 67.0, 62.9, 52.5, 43.8, 39.0.
HRMS (ESI): Calcd for $C_{19}H_{24}N_3O_2^+$ [M+H]$^+$: 326.1863, found 326.1856.

Benzyl 4-(2-methylene-4-(morpholine-4-carbonyl)pent-4-en-1-yl)piperazine-1-carboxylate (40)

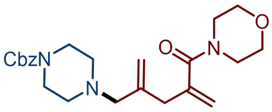

Prepared according to the **GP5** from benzyl piperazine-1-carboxylate (22.0 mg, 0.1 mmol) and (1-(iodomethyl)-3-methylenecyclobutyl)(morpholino)methanone (48.2 mg, 0.15 mmol), The crude residue was purified by column chromatography to yield **40** (19.8 mg, 48% yield) as a colorless oil.

^{1}H NMR (500 MHz, $CDCl_3$): δ 7.37 – 7.29 (m, 5H), 5.26 (s, 1H), 5.13 (s, 1H), 5.12 (s, 2H), 5.06 (s, 1H), 4.96 (s, 1H), 3.69 – 3.55 (m, 9H), 3.51 – 3.46 (m, 4H), 3.07 (s, 2H), 2.90 (s, 2H), 2.40 – 2.28 (m, 4H).
^{13}C NMR (126 MHz, $CDCl_3$): δ 170.3, 155.2, 142.1, 141.8, 136.7, 128.4, 127.9, 127.8, 116.5, 115.8, 67.0, 66.9, 62.9, 52.6, 47.5, 43.8, 41.9, 38.4.
HRMS (ESI): Calcd for $C_{23}H_{32}N_3O_4^+$ [M+H]$^+$: 414.2387, found 414.2380.

Benzyl-4-(2-allyl-5-methylhexa-2,4-dien-1-yl)piperazine-1-carboxylate (41)

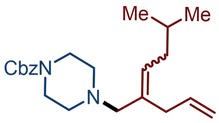

Prepared according to the **GP5** from benzyl piperazine-1-carboxylate (22.0 mg, 0.1 mmol) and 1-(iodomethyl)-3-(3-methylbutylidene)cyclobutane (39.6 mg, 0.15 mmol), The crude residue was purified by column chromatography to yield **41** (32.0 mg, 90% yield, 1.4:1 *E/Z*) as a colorless oil.

^{1}H NMR (500 MHz, CDCl$_3$, *E/Z* mixture): δ 7.40 – 7.29 (m, 5H), 5.89 – 5.70 (m, 1H), 5.44 – 5.33 (m, 1H), 5.13 (s, 2H), 5.07 – 4.93 (m, 2H), 3.52 – 3.46 (m, 4H), 2.91 (s, 1H), 2.85 – 2.79 (m, 3H), 2.35 – 2.27 (m, 4H), 1.94 (t, *J* = 7.0 Hz, 2H), 1.64 – 1.55 (m, 1H), 0.93 – 0.84 (m, 6H).
^{13}C NMR (126 MHz, CDCl$_3$, *E/Z* mixture): δ 155.2, 137.3, 136.8, 136.2, 134.7, 134.1, 129.1, 128.8, 128.4, 127.9, 127.8, 115.5, 114.9, 67.0, 64.9, 56.8, 52.7, 52.6, 44.0, 40.2, 36.7, 36.7, 33.2, 28.9, 28.7, 22.5, 22.4.
HRMS (ESI): Calcd for $C_{22}H_{33}N_2O_2^+$ [M+H]$^+$: 357.2537, found 357.2536.

Benzyl 4-(2-benzylidenepent-4-en-1-yl)piperazine-1-carboxylate (42)

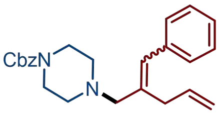

Prepared according to the **GP5** from benzyl piperazine-1-carboxylate (22.0 mg, 0.1 mmol) and ((3-(iodomethyl)cyclobutylidene)methyl)benzene (42.6 mg, 0.15 mmol), The crude residue was purified by column chromatography to yield **42** (24.5 mg, 83% yield, 1.8:1 *E/Z*) as a colorless oil.

^{1}H NMR (400 MHz, CDCl$_3$, *E/Z* mixture): δ 7.41 – 7.26 (m, 8H), 7.24 – 7.13 (m, 2H), 6.58 – 6.51 (m, 1H), 6.05 – 5.85 (m, 1H), 5.21 – 5.04 (m, 4H), 3.57 – 3.43 (m, 4H), 3.16 – 2.98 (m, 4H), 2.56 – 2.21 (m, 4H).
^{13}C NMR (101 MHz, CDCl$_3$, *E/Z* mixture): δ 155.3, 155.2, 138.2, 137.5, 137.3, 136.8, 136.7, 136.7, 136.6, 135.9, 129.6, 129.0, 128.9, 128.5, 128.5, 128.4, 128.1, 128.0, 127.9, 127.8, 127.8, 126.6, 126.4, 116.3, 115.9, 67.0, 67.0, 64.6, 57.0, 52.7, 52.5, 43.9, 40.0, 33.7.
HRMS (ESI): Calcd for $C_{24}H_{29}N_2O_2^+$ [M+H]$^+$: 377.2224, found 377.2226.

Benzyl-4-(2-methylenehex-4-en-1-yl)piperazine-1-carboxylate (43)

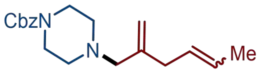

Prepared according to the **GP5** from benzyl piperazine-1-carboxylate (22.0 mg, 0.1 mmol) and 1-(1-iodoethyl)-3-methylenecyclobutane (33.3 mg, 0.15 mmol), The crude residue was purified by column chromatography to yield **43** (31.1 mg, 99% yield, 3:1 *E/Z*) as a colorless oil.

^{1}H NMR (400 MHz, CDCl$_3$, *E/Z* mixture): δ 7.43 – 7.27 (m, 5H), 5.64 – 5.36 (m, 2H), 5.13 (s, 2H), 5.01 – 4.84 (m, 2H), 3.51 (d, *J* = 4.8 Hz, 4H), 2.87 (s, 2H), 2.77 (dd, *J* = 33.8, 6.4 Hz, 2H), 2.38 – 2.29 (m, 4H), 1.70 – 1.61 (m, 3H).

^{13}C NMR (101 MHz, CDCl$_3$, *E/Z* mixture): δ 155.2, 145.4, 136.8, 128.5, 128.4, 127.9, 127.8, 127.6, 126.7, 125.4, 112.7, 67.0, 63.7, 52.7, 52.7, 43.9, 37.4, 31.7, 17.9, 12.7.
HRMS (ESI): Calcd for $C_{19}H_{27}N_2O_2^+$ [M+H]$^+$: 315.2067, found 315.2068.

Benzyl 4-(4-(((10,14-dimethyl-15-(6-methylheptan-2-yl)-2,3,4,7,8,9,10,11,12,13,14,15,16,17-tetradecahydro-1H-cyclopenta[a]phenanthren-3-yl)oxy)carbonyl)-2-methylenepent-4-en-1-yl)piperazine-1-carboxylate(44)

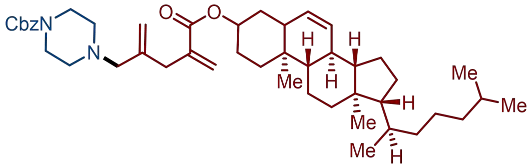

Prepared according to the **GP5** from benzyl piperazine-1-carboxylate (22.0 mg, 0.1 mmol) and (8S,9S,10S,13R,14S,17R)-10,13-dimethyl-17-((R)-6-methylheptan-2-yl)-2,3,4,5,8,9,10,11,12,13,14,15,16,17-tetradecahydro-1H-cyclopenta[a]phenanthren-3-yl 1-(iodomethyl)-3-methylenecyclobutane-1-carboxylate (93.0 mg, 0.15 mmol), The crude residue was purified by column chromatography to yield **44** (37.0 mg, 52% yield) as a colorless oil.

^{1}H NMR (500 MHz, CDCl$_3$): δ 7.37 – 7.29 (m, 5H), 6.20 (s, 1H), 5.54 (s, 1H), 5.37 (s, 1H), 5.13 (s, 2H), 4.99 (s, 1H), 4.88 (s, 1H), 4.71 – 4.60 (m, 1H), 3.56 – 3.44 (m, 4H), 3.07 (s, 2H), 2.89 (s, 2H), 2.40 – 2.29 (m, 6H), 2.06 – 1.92 (m, 3H), 1.91 – 1.80 (m, 4H), 1.65 – 1.42 (m, 9H), 1.39 – 1.24 (m, 7H), 1.19 – 1.06 (m, 9H), 1.05 – 0.99 (m, 6H), 0.94 – 0.90 (m, 4H), 0.89 – 0.84 (m, 8H), 0.70 – 0.66 (m, 4H).
^{13}C NMR (126 MHz, CDCl$_3$): δ 166.4, 155.3, 143.6, 139.5, 138.9, 136.8, 128.5, 127.9, 127.8, 125.9, 122.7, 114.4, 74.3, 67.0, 63.6, 56.7, 56.1, 52.6, 50.0, 43.9, 42.3, 39.7, 39.5, 38.1, 36.9, 36.6, 36.4, 36.2, 35.8, 31.9, 31.8, 28.2, 28.0, 27.7, 24.3, 23.8, 22.8, 22.5, 21.0, 19.3, 18.7, 11.8.
HRMS (ESI): Calcd for $C_{46}H_{69}N_2O_4^+$ [M+H]$^+$: 713.5252, found 713.5255.

Benzyl (*S,E*)-4-(4-(1-tosylpyrrolidin-3-yl)but-2-en-1-yl)piperazine-1-carboxylate (45)

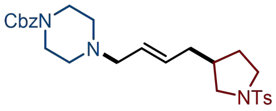

Prepared according to the **GP6** from benzyl piperazine-1-carboxylate (22.0 mg, 0.1 mmol) and 3-iodo-1-tosylpyrrolidine (52.7 mg, 0.15 mmol), buta-1,3-diene (150 μL, 0.3 mmol), The crude residue was purified by column chromatography to yield **45** (44.8 mg, 90% yield, 19:1 *E/Z,* > 20:1 rr) as a colorless oil.

^{1}H NMR (400 MHz, CDCl$_3$): δ 7.68 (d, *J* = 8.1 Hz, 2H), 7.37 – 7.28 (m, 7H), 5.50 – 5.35 (m, 2H), 5.11 (s, 2H), 3.55 – 3.46 (m, 4H), 3.40 – 3.27 (m, 2H), 3.24 – 3.11 (m,

1H), 2.95 – 2.87 (m, 2H), 2.84 – 2.78 (m, 1H), 2.41 (s, 3H), 2.37 – 2.32 (m, 4H), 2.14 – 2.03 (m, 2H), 2.00 – 1.94 (m, 2H), 1.92 – 1.83 (m, 1H), 1.46 – 1.34 (m, 1H).
^{13}C NMR (101 MHz, CDCl$_3$): δ 155.1, 143.3, 136.6, 133.7, 131.7, 129.5, 128.4, 128.0, 127.9, 127.8, 127.4, 67.0, 60.5, 52.7, 52.5, 47.3, 43.6, 38.3, 35.7, 30.8, 21.4.
HRMS (ESI): Calcd for $C_{27}H_{36}N_3O_4S^+$ [M+H]$^+$: 498.2421, found 498.2410.

Benzyl (*E*)-4-(4-(tetrahydro-2H-thiopyran-4-yl)but-2-en-1-yl)piperazine-1-carboxylate (46)

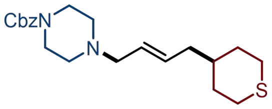

Prepared according to the **GP6** from benzyl piperazine-1-carboxylate (22.0 mg, 0.1 mmol) and 4-iodotetrahydro-2H-thiopyran (34.2 mg, 0.15 mmol), buta-1,3-diene (150 µL, 0.3 mmol), The crude residue was purified by column chromatography to yield **46** (30.0 mg, 80% yield, 14:1 *E/Z,* > 20:1 rr) as a colorless oil.

^{1}H NMR (400 MHz, CDCl$_3$): δ 7.39 – 7.28 (m, 5H), 5.58 – 5.39 (m, 2H), 5.12 (s, 2H), 3.57 – 3.46 (m, 4H), 2.94 (d, J = 6.2 Hz, 2H), 2.68 – 2.51 (m, 4H), 2.42 – 2.34 (m, 4H), 2.01 – 1.92 (m, 4H), 1.41 – 1.28 (m, 2H).
^{13}C NMR (101 MHz, CDCl$_3$): δ 155.2, 136.7, 132.3, 128.4, 128.0, 127.8, 127.8, 67.1, 60.8, 52.6, 43.7, 40.2, 37.2, 33.8, 28.7.
HRMS (ESI): Calcd for $C_{21}H_{31}N_2O_2S^+$ [M+H]$^+$: 375.2101, found 375.2094.

Benzyl (*E*)-4-(4-(1-(*tert*-butoxycarbonyl)piperidin-4-yl)but-2-en-1-yl)piperazine -1-carboxylate (47)

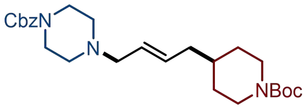

Prepared according to the **GP6** from benzyl piperazine-1-carboxylate (22.0 mg, 0.1 mmol) and *tert*-butyl 4-iodopiperidine-1-carboxylate (31.2 mg, 0.15 mmol), buta-1,3-diene (150 µL, 0.3 mmol), The crude residue was purified by column chromatography to yield **47** (35.7 mg, 78% yield, 15:1 *E/Z,* > 20:1 rr) as a colorless oil.

^{1}H NMR (500 MHz, CDCl$_3$): δ 7.39 – 7.28 (m, 5H), 5.61 – 5.40 (m, 2H), 5.12 (s, 2H), 4.21 – 3.96 (m, 2H), 3.57 – 3.48 (m, 4H), 3.05 – 2.93 (m, 2H), 2.70 – 2.60 (m, 2H), 2.45 – 2.33 (m, 4H), 2.04 – 1.94 (m, 2H), 1.69 – 1.56 (m, 2H), 1.44 (s, 9H), 1.13 – 1.02 (m, 2H).
^{13}C NMR (126 MHz, CDCl$_3$): δ 155.2, 154.8, 136.7, 132.5, 128.4, 128.0, 127.9, 127.6, 79.2, 67.1, 60.8, 52.6, 43.7, 43.7, 39.4, 36.0, 31.9, 28.4.
HRMS (ESI): Calcd for $C_{26}H_{40}N_3O_4^+$ [M+H]$^+$: 458.3013, found 458.3003.

Benzyl (*E*)-4-(4-(4,4-difluorocyclohexyl)but-2-en-1-yl)piperazine-1-carboxylate (48)

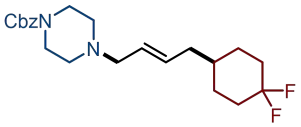

Prepared according to the **GP6** from benzyl piperazine-1-carboxylate (22.0 mg, 0.1 mmol) and 1,1-difluoro-4-iodocyclohexane (36.9 mg, 0.15 mmol), buta-1,3-diene (150 μL, 0.3 mmol), The crude residue was purified by column chromatography to yield **48** (20.4 mg, 52% yield, 14:1 *E/Z,* > 20:1 rr) as a colorless oil.

^{1}H NMR (400 MHz, CDCl$_3$): δ 7.39 – 7.28 (m, 5H), 5.62 – 5.43 (m, 2H), 5.13 (s, 2H), 3.56 – 3.49 (m, 4H), 2.95 (d, J = 6.3 Hz, 2H), 2.43 – 2.35 (m, 4H), 2.12 – 1.96 (m, 4H), 1.81 – 1.71 (m, 2H), 1.66 – 1.58 (m, 1H), 1.32 – 1.19 (m, 4H).
^{13}C NMR (101 MHz, CDCl$_3$): δ 155.2, 136.7, 132.6, 128.5, 128.0, 127.9, 127.7, 67.1, 60.8, 52.7, 43.8, 38.6 (d, J = 2.3 Hz), 35.8, 33.4 (dd, J = 25.4, 22.4 Hz), 28.7, 28.6.
^{19}F NMR (376 MHz, CDCl$_3$): δ -91.3, -91.9, -101.5, -102.2. (2F)
HRMS (ESI): Calcd for $C_{22}H_{31}F_2N_2O_2^+$ [M+H]$^+$: 393.2348, found 393.2339.

Benzyl (*E*)-4-(4-(4-oxocyclohexyl)but-2-en-1-yl)piperazine-1-carboxylate (49)

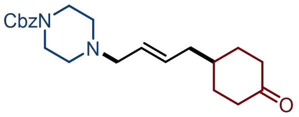

Prepared according to the **GP6** from benzyl piperazine-1-carboxylate (22.0 mg, 0.1 mmol) and 4-iodocyclohexan-1-one (43.6 mg, 0.15 mmol), buta-1,3-diene (150 μL, 0.3 mmol), The crude residue was purified by column chromatography to yield **49** (15.2 mg, 41% yield, 15:1 *E/Z,* > 20:1 rr) as a colorless oil.

^{1}H NMR (400 MHz, CDCl$_3$): δ 7.41 – 7.27 (m, 5H), 5.68 – 5.43 (m, 2H), 5.12 (s, 2H), 3.58 – 3.47 (m, 4H), 3.04 – 2.90 (m, 2H), 2.47 – 2.24 (m, 8H), 2.12 – 1.99 (m, 4H), 1.96 – 1.88 (m, 1H), 1.84 – 1.72 (m, 1H), 1.46 – 1.32 (m, 2H).
^{13}C NMR (101 MHz, CDCl$_3$): δ 212.0, 155.2, 136.7, 132.4, 128.4, 128.0, 127.9, 127.9, 67.1, 60.7, 52.6, 43.7, 40.7, 38.4, 36.0, 32.3.
HRMS (ESI): Calcd for $C_{22}H_{31}N_2O^{3+}$ [M+H]$^+$: 371.2329, found 371.2322.

Benzyl 4-((*E*)-4-((6S)-2-oxohexahydro-2H-3,5-methanocyclopenta[b]furan-6-yl) but-2-en-1-yl)piperazine-1-carboxylate (50)

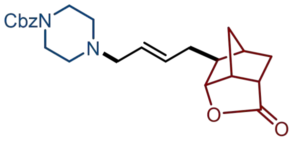

Prepared according to the **GP6** from benzyl piperazine-1-carboxylate (22.0 mg, 0.1 mmol) and 6-iodohexahydro-2H-3,5-methanocyclopenta[b]furan-2-one (39.6 mg, 0.15 mmol), buta-1,3-diene (150 μL, 0.3 mmol), The crude residue was purified by column chromatography to yield **50** (27.5 mg, 67% yield, 19:1 *E/Z,* > 20:1 rr) as a colorless oil.

^{1}H NMR (400 MHz, CDCl$_3$): δ 7.38 – 7.28 (m, 5H), 5.57 – 5.49 (m, 2H), 5.12 (s, 2H), 4.33 (d, J = 4.8 Hz, 1H), 3.59 – 3.47 (m, 4H), 3.16 – 3.10 (m, 1H), 3.02 – 2.91 (m, 2H), 2.57 – 2.49 (m, 1H), 2.43 – 2.35 (m, 4H), 2.22 (s, 1H), 2.05 – 1.94 (m, 3H), 1.87 – 1.76 (m, 3H), 1.73 – 1.61 (m, 2H), 1.51 (d, J = 9.9 Hz, 1H).
^{13}C NMR (101 MHz, CDCl$_3$): δ 181.0, 155.2, 136.7, 131.2, 128.5, 128.4, 128.0, 127.9, 85.1, 67.1, 60.6, 52.6, 48.8, 46.2, 43.7, 40.6, 38.8, 35.1, 34.7, 34.6.
HRMS (ESI): Calcd for $C_{24}H_{31}N_2O_4^+$ [M+H]$^+$: 411.2278, found 411.2268.

Enzyl (*E*)-4-(4-(1-methylcyclohexyl)but-2-en-1-yl)piperazine-1-carboxylate (51)

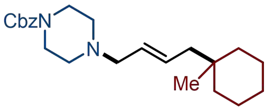

Prepared according to the **GP6** from benzyl piperazine-1-carboxylate (22.0 mg, 0.1 mmol) and 1-iodo-1-methylcyclohexane (33.6 mg, 0.15 mmol), buta-1,3-diene (150 µL, 0.3 mmol), The crude residue was purified by column chromatography to yield **51** (14.8 mg, 40% yield, 19:1 *E/Z,* > 20:1 rr) as a colorless oil.

^{1}H NMR (400 MHz, CDCl$_3$): δ 7.38 – 7.29 (m, 5H), 5.69 – 5.39 (m, 2H), 5.13 (s, 2H), 3.56 – 3.49 (m, 4H), 3.02 – 2.94 (m, 2H), 2.43 – 2.36 (m, 4H), 1.95 (d, J = 7.3 Hz, 2H), 1.70 (s, 2H), 1.45 – 1.40 (m, 4H), 1.28 – 1.19 (m, 4H), 0.83 (s, 3H).
^{13}C NMR (101 MHz, CDCl$_3$): δ 155.2, 136.7, 131.7, 128.5, 128.0, 128.0, 127.9, 67.1, 61.1, 52.6, 43.8, 37.7, 33.2, 29.7, 26.4, 25.2, 22.0.
HRMS (ESI): Calcd for $C_{23}H_{35}N_2O_2^+$ [M+H]$^+$: 371.2693, found 371.2685.

Benzyl 4-((*E*)-4-((3r,5r,7r)-adamantan-1-yl)but-2-en-1-yl)piperazine-1-carboxylate (52)

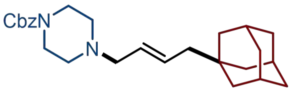

Prepared according to the **GP6** from benzyl piperazine-1-carboxylate (22.0 mg, 0.1 mmol) and 1-iodoadamantane (39.3 mg, 0.15 mmol), buta-1,3-diene (150 µL, 0.3 mmol), The crude residue was purified by column chromatography to yield **52** (40.0 mg, 98% yield, 19:1 *E/Z,* > 20:1 rr) as a colorless oil.

^{1}H NMR (400 MHz, CDCl$_3$): δ 7.38 – 7.28 (m, 5H), 5.72 – 5.52 (m, 1H), 5.49 – 5.34 (m, 1H), 5.12 (s, 2H), 3.56 – 3.48 (m, 4H), 2.96 (d, J = 6.7 Hz, 2H), 2.45 – 2.34 (m, 4H), 1.96 – 1.90 (m, 4H), 1.81 – 1.76 (m, 2H), 1.72 – 1.66 (m,4H), 1.61 – 1.56 (m, 4H), 1.48 – 1.43 (m, 6H).
^{13}C NMR (101 MHz, CDCl$_3$): δ 155.2, 136.7, 130.9, 128.4, 128.0, 127.9, 127.8, 67.0, 61.0, 52.6, 47.4, 45.3, 43.8, 42.4, 42.4, 42.3, 37.0, 37.0, 36.0, 32.8, 30.7, 28.6.
HRMS (ESI): Calcd for $C_{26}H_{37}N_2O_2^+$ [M+H]$^+$: 409.2850, found 409.2839.

Benzyl (*R,E*)-4-(5-(tetrahydro-2H-pyran-4-yl)hex-2-en-1-yl)piperazine-1-carboxylate (53)

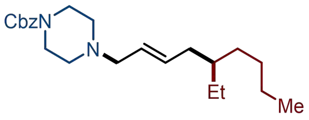

Prepared according to the **GP6** from benzyl piperazine-1-carboxylate (22.0 mg, 0.1 mmol) and 3-iodoheptane (33.9 mg, 0.15 mmol), buta-1,3-diene (150 µL, 0.3 mmol), The crude residue was purified by column chromatography to yield **53** (26.1 mg, 70% yield, 12:1 *E/Z,* > 20:1 rr) as a colorless oil.

^{1}H NMR (400 MHz, CDCl$_3$): δ 7.42 – 7.26 (m, 5H), 5.63 – 5.36 (m, 2H), 5.13 (s, 2H), 3.57 – 3.46 (m, 4H), 2.95 (d, J = 6.5 Hz, 2H), 2.44 – 2.36 (m, 4H), 2.06 – 1.91 (m, 2H), 1.33 – 1.17 (m, 9H), 0.93 – 0.78 (m, 6H).
^{13}C NMR (101 MHz, CDCl$_3$): δ 155.2, 136.7, 133.7, 128.4, 127.9, 127.8, 127.0, 67.0, 61.0, 52.6, 43.8, 39.0, 36.0, 32.6, 28.9, 25.8, 23.0, 14.1, 10.9.
HRMS (ESI): Calcd for $C_{23}H_{37}N_2O_2^+$ [M+H]$^+$: 373.2850, found 373.2841.

Benzyl (*R,E*)-4-(5-ethylnon-2-en-1-yl)piperazine-1-carboxylate (54)

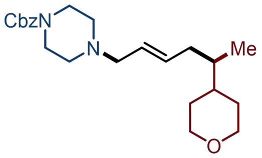

Prepared according to the **GP6** from benzyl piperazine-1-carboxylate (22.0 mg, 0.1 mmol) and 4-(1-iodoethyl)tetrahydro-2H-pyran (36.0 mg, 0.15 mmol), buta-1,3-diene (150 µL, 0.3 mmol), The crude residue was purified by column chromatography to yield **54** (27.1 mg, 70% yield, 14:1 *E/Z,* > 20:1 rr) as a colorless oil.

^{1}H NMR (400 MHz, CDCl$_3$): δ 7.46 – 7.26 (m, 5H), 5.65 – 5.34 (m, 2H), 5.12 (s, 2H), 4.06 – 3.88 (m, 2H), 3.60 – 3.47 (m, 4H), 3.39 – 3.24 (m, 2H), 3.02 – 2.87 (m, 2H), 2.47 – 2.30 (m, 4H), 2.19 – 2.09 (m, 1H), 1.99 (s, 1H), 1.91 – 1.81 (m, 1H), 1.56 – 1.47 (m, 2H), 1.44 – 1.32 (m, 3H), 0.83 (d, J = 6.4 Hz, 3H).
^{13}C NMR (101 MHz, CDCl$_3$): δ 155.2, 136.7, 133.6, 128.4, 128.0, 127.8, 127.3, 68.4, 68.3, 67.1, 60.9, 52.6, 43.7, 39.6, 37.6, 36.6, 30.6, 29.2, 15.9.
HRMS (ESI): Calcd for $C_{23}H_{35}N_2O_3^+$ [M+H]$^+$: 387.2642, found 387.2648.

Benzyl 4-((*E*)-4-((1R)-2-fluorocyclohexyl)but-2-en-1-yl)piperazine-1-carboxylate (55)

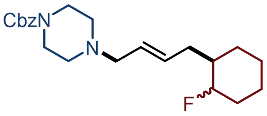

Prepared according to the **GP6** from benzyl piperazine-1-carboxylate (22.0 mg, 0.1 mmol) and 1-fluoro-2-iodocyclohexane (34.2 mg, 0.15 mmol), buta-1,3-diene (150 µL, 0.3 mmol), The crude residue was purified by column chromatography to yield **55** (28.0 mg, 75% yield, 14:1 *E/Z*, > 20:1 rr, 2.4:1 dr) as a colorless oil.

^{1}H NMR (400 MHz, CDCl$_3$, diastereomers): δ 7.41 – 7.27 (m, 5H), 5.64 – 5.43 (m, 2H), 5.12 (s, 2H), 4.79 – 4.54 (m, 0.3H), 4.25 – 3.98 (m, 0.8H), 3.57 – 3.46 (m, 4H), 2.95 (d, J = 6.3 Hz, 2H), δ 2.48 – 2.33 (m, 4H), 2.11 – 1.89 (m, 3H), 1.80 – 1.70 (m, 2H), 1.61 – 1.35 (m, 3H), 1.31 – 1.08 (m, 3H), 0.99 – 0.85 (m, 3H).
^{13}C NMR (101 MHz, CDCl$_3$, diastereomers): δ 155.2, 136.7, 132.7, 132.3, 128.4, 127.9, 127.8, 127.7, 96.4, 94.7, 91.9, 90.2, 43.7, 43.1, 43.0, 40.9, 40.7, 34.9, 32.3, 32.2, 31.1, 30.8, 29.6, 29.6, 26.6, 25.0, 24.8, 24.1, 24.0, 20.2.
^{19}F NMR (376 MHz, CDCl$_3$, diastereomers): δ -174.0, -174.1.
HRMS (ESI): Calcd for $C_{22}H_{32}FN_2O_2^+$ [M+H]$^+$: 375.2442, found 375.2432.

Benzyl 4-((*E*)-4-((1R)-2-ethoxycyclohexyl)but-2-en-1-yl)piperazine-1-carboxylate (56)

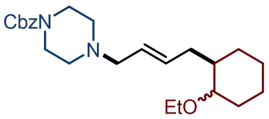

Prepared according to the **GP6** from benzyl piperazine-1-carboxylate (22.0 mg, 0.1 mmol) and 1-ethoxy-2-iodocyclohexane (38.1 mg, 0.15 mmol), buta-1,3-diene (150 µL, 0.3 mmol), The crude residue was purified by column chromatography to yield **56** (20.8 mg, 52% yield, 14:1 *E/Z*, > 20:1 rr, 2.1:1) as a colorless oil.

^{1}H NMR (400 MHz, CDCl$_3$, diastereomers): δ 7.43 – 7.26 (m, 5H), 5.62 – 5.37 (m, 2H), 5.12 (s, 2H), 3.71 – 3.60 (m, 1H), 3.55 – 3.49 (m, 4H), 3.41 – 3.28 (m, 1H), 3.04 – 2.92 (m, 2H), 2.85 – 2.78 (m, 1H), 2.54 – 2.46 (m, 1H), 2.43 – 2.35 (m, 4H), 2.22 – 1.71 (m, 5H), 1.63 – 1.48 (m, 2H), 1.40 – 1.24 (m, 2H), 1.21 – 1.11 (m, 4H).
^{13}C NMR (101 MHz, CDCl$_3$, diastereomers): δ 155.2, 136.7, 134.2, 133.5, 128.4, 128.0, 127.8, 127.1, 126.7, 81.5, 67.1, 63.9, 63.5, 61.0, 52.6, 43.8, 43.1, 40.6, 35.3, 31.4, 30.5, 28.7, 27.1, 25.5, 25.4, 24.8, 24.5, 21.2, 15.7.
HRMS (ESI): Calcd for $C_{24}H_{37}N_2O_3^+$ [M+H]$^+$: 401.2799, found 401.2791.

Benzyl 4-((*E*)-4-((3S,10R,13S)-16-acetyl-10,13-dimethyl-2,3,4,5,8,9,10,11,12,13, 14,15,16,17-tetradecahydro-1H-cyclopenta[a]phenanthren-3-yl)but-2-en-1-yl)pip erazine-1-carboxylate (57)

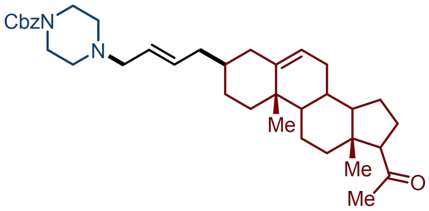

Prepared according to the **GP6** from benzyl piperazine-1-carboxylate (22.0 mg, 0.1 mmol) and *tert*-butyl 4-iodopiperidine-1-carboxylate (31.2 mg, 0.15 mmol), buta-1,3-diene (150 µL, 0.3 mmol), The crude residue was purified by column chromatography to yield **57** (48.7 mg, 85% yield, 12:1 *E/Z*, > 20:1 rr, 1.7:1 dr) as a colorless oil.

^{1}**H NMR (400 MHz, CDCl$_3$, diastereomers):** δ 7.40 – 7.27 (m, 5H), 5.64 – 5.39 (m, 2H), 5.28 – 5.20 (m, 1H), 5.13 (s, 2H), 3.56 – 3.46 (m, 4H), 3.04 – 2.93 (m, 2H), 2.48 – 2.31 (m, 4H), 2.05 – 1.87 (m, 5H), 1.86 – 1.77 (m, 2H), 1.75 (s, 1H), 1.49 – 1.38 (m, 3H), 1.34 – 1.23 (m, 3H), 1.17 – 1.05 (m, 3H), 1.00 (s, 3H), 0.94 – 0.90 (m, 3H), 0.88 – 0.83 (m, 6H), 0.67 (s, 3H).
^{13}**C NMR (101 MHz, CDCl$_3$, diastereomers):** δ 155.2, 143.1, 140.2, 136.7, 134.3, 133.4, 128.4, 127.9, 127.8, 126.9, 126.7, 121.3, 119.4, 67.0, 61.0, 60.9, 56.8, 56.1, 52.7, 52.6, 50.5, 50.4, 43.7, 42.2, 40.1, 39.8, 39.5, 39.4, 39.3, 37.3, 37.1, 36.4, 36.1, 35.8, 34.2, 33.9, 33.8, 31.9, 31.8, 28.8, 28.2, 28.0, 25.9, 24.2, 23.8, 23.8, 22.8, 22.5, 20.9, 20.7, 19.4, 19.4, 18.7, 11.8.
HRMS (ESI): Calcd for $C_{37}H_{53}N_2O_3^+$ [M+H]$^+$: 573.4051, found 573.4058.

Benzyl (*E*)-4-(5-(2,2,7,7-tetramethyltetrahydro-5H-bis([1,3]dioxolo)[4,5-b:4',5'-d] pyran-5-yl)pent-2-en-1-yl)piperazine-1-carboxylate (58)

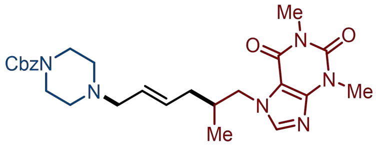

Prepared according to the **GP6** from benzyl piperazine-1-carboxylate (22.0 mg, 0.1 mmol) and *tert*-butyl 4-iodopiperidine-1-carboxylate (31.2 mg, 0.15 mmol), buta-1,3-diene (150 µL, 0.3 mmol), The crude residue was purified by column chromatography to yield **58** (39.6 mg, 80% yield, 19:1 *E/Z*, > 20:1 rr) as a colorless oil.
^{1}**H NMR (400 MHz, CDCl$_3$):** δ 7.48 (s, 1H), 7.35 – 7.24 (m, 5H), 5.64 – 5.45 (m, 2H), 5.10 (s, 2H), 4.27 – 4.15 (m, 1H), 4.03 – 3.93 (m, 1H), 3.56 (s, 3H), 3.54 – 3.50 (m, 4H), 3.37 (s, 3H), 2.98 (d, J = 5.9 Hz, 2H), 2.45 – 2.38 (m, 4H), 2.23 – 1.90 (m, 3H), 0.86 (d, J = 6.7 Hz, 3H).
^{13}**C NMR (101 MHz, CDCl$_3$):** δ 155.1, 155.1, 151.6, 148.9, 141.2, 136.6, 131.7, 128.4, 128.2, 127.9, 127.8, 107.0, 67.1, 60.5, 52.6, 52.5, 43.5, 36.8, 34.1, 29.7, 29.6, 27.9, 16.8.
HRMS (ESI): Calcd for $C_{26}H_{35}N_6O_4^+$ [M+H]$^+$: 495.2714, found 497.2722.

Benzyl 4-((5*S*,*E*)-6-(1,3-dimethyl-2,6-dioxo-1,2,3,4,5,6-hexahydro-7H-purin-7-yl)-5-methylhex-2-en-1-yl)piperazine-1-carboxylate (59)

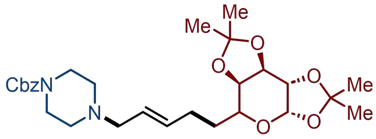

Prepared according to the **GP6** from benzyl piperazine-1-carboxylate (22.0 mg, 0.1 mmol) and *tert*-butyl 4-iodopiperidine-1-carboxylate (31.2 mg, 0.15 mmol), buta-1,3-diene (150 µL, 0.3 mmol), The crude residue was purified by column chromatography to yield **59** (47.0 mg, 91% yield, 19:1 *E/Z,* > 20:1 rr) as a colorless oil.

^{1}H NMR (400 MHz, CDCl$_3$): δ 7.37 – 7.26 (m, 5H), 5.67 – 5.43 (m, 3H), 5.11 (s, 2H), 4.59 – 4.52 (m, 1H), 4.31 – 4.24 (m, 1H), 4.14 – 4.06 (m, 1H), 3.76 – 3.67 (m, 1H), 3.54 – 3.46 (m, 4H), 3.04 – 2.87 (m, 1H), 2.42 – 2.33 (m, 4H), 2.26 – 2.00 (m, 3H), 1.86 – 1.70 (m, 1H), 1.67 – 1.53 (m, 1H), 1.48 (s, 3H), 1.44 (s, 3H), 1.32 (s, 3H), 1.30 (s, 3H).
^{13}C NMR (101 MHz, CDCl$_3$): δ 155.2, 136.7, 134.2, 128.4, 127.9, 127.8, 126.4, 108.9, 108.2, 96.5, 72.7, 70.8, 70.5, 67.0, 66.7, 60.8, 52.6, 43.7, 29.6, 28.3, 26.0, 25.9, 24.8, 24.3.
HRMS (ESI): Calcd for $C_{28}H_{41}N_2O_7^+$ [M+H]$^+$: 517.2908, found 517.2911.

Benzyl (*R*,*E*)-4-(5-(oxetan-3-yl)-1-phenylpent-3-en-2-yl)piperazine-1-carboxylate (60)

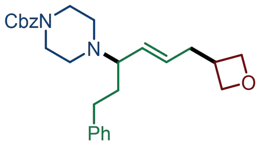

Prepared according to the **GP6** from benzyl piperazine-1-carboxylate (22.0 mg, 0.1 mmol) and 3-iodooxetane (27.6 mg, 0.15 mmol), (*E*)-penta-2,4-dien-1-ylbenzene (43.6 mg, 0.3 mmol), The crude residue was purified by column chromatography to yield **60** (27.8 mg, 66% yield, 19:1 *E/Z,* > 20:1 rr) as a colorless oil.

^{1}H NMR (400 MHz, CDCl$_3$): δ 7.37 – 7.24 (m, 7H), 7.22 – 7.13 (m, 3H), 5.49 – 5.29 (m, 2H), 5.13 (s, 2H), 4.82 – 4.76 (m, 2H), 4.41 – 4.35 (m, 2H), 3.56 – 3.41 (m, 4H), 3.13 – 2.97 (m, 1H), 2.82 – 2.74 (m, 1H), 2.67 – 2.49 (m, 4H), 2.48 – 2.42 (m, 2H), 2.40 – 2.31 (m, 2H), 1.99 – 1.88 (m, 1H), 1.76 – 1.64 (m, 1H).
^{13}C NMR (101 MHz, CDCl$_3$): δ 155.1, 142.0, 136.7, 130.7, 130.0, 128.4, 128.3, 128.3, 127.9, 127.8, 125.8, 76.9, 76.8, 67.0, 66.5, 49.0, 44.1, 36.2, 34.3, 33.8, 32.5.
HRMS (ESI): Calcd for $C_{28}H_{41}N_2O_7^+$ [M+H]$^+$: 517.2908, found 517.2912.

Benzyl (*R*,*E*)-4-(1-cyclohexyl-4-(oxetan-3-yl)but-2-en-1-yl)piperazine-1-carboxylate (61)

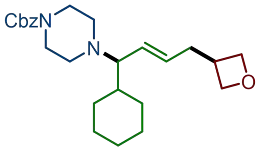

Prepared according to the **GP6** from benzyl piperazine-1-carboxylate (22.0 mg, 0.1 mmol) and 3-iodooxetane (27.6 mg, 0.15 mmol), (*E*)-penta-2,4-dien-1-ylcyclohexane (45.1 mg, 0.3 mmol), The crude residue was purified by column chromatography to yield **61** (16.5 mg, 40% yield, 12:1 *E/Z,* > 20:1 rr) as a colorless oil.

^{1}H NMR (500 MHz, CDCl$_3$): δ 7.38 – 7.27 (m, 5H), 5.47 – 5.32 (m, 1H), 5.21 – 5.06 (m, 3H), 4.80 – 4.62 (m, 2H), 4.47 – 4.30 (m, 2H), 3.47 (s, 4H), 3.20 – 2.84 (m, 2H), 2.62 – 2.21 (m, 5H), 2.14 – 1.56 (m, 7H), 1.36 – 0.93 (m, 5H).
^{13}C NMR (126 MHz, CDCl$_3$): δ 155.1, 140.7, 136.7, 128.4, 127.9, 127.8, 124.8, 78.0,

77.6, 67.0, 66.6, 49.3, 44.0, 40.5, 36.3, 33.3, 33.0, 32.8, 26.0, 25.9.
HRMS (ESI): Calcd for $C_{25}H_{37}N_2O_3^+$ [M+H]$^+$: 413.2799, found 413.2791.

Benzyl (*S,E*)-4-(4-(oxetan-3-yl)-1-phenylbut-2-en-1-yl)piperazine-1-carboxylate (62)

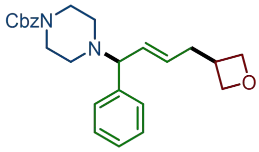

Prepared according to the **GP6** from benzyl piperazine-1-carboxylate (22.0 mg, 0.1 mmol) and 3-iodooxetane (27.6 mg, 0.15 mmol), (*E*)-penta-2,4-dien-1-ylbenzene (43.3 mg, 0.3 mmol), The crude residue was purified by column chromatography to yield **62** (20.7 mg, 51% yield, 19:1 *E/Z,* > 20:1 rr) as a colorless oil.

^{1}H NMR (400 MHz, CDCl$_3$): δ 7.37 – 7.25 (m, 10H), 5.58 – 5.50 (m, 2H), 5.12 (s, 2H), 4.79 – 4.71 (m, 2H), 4.37 – 4.28 (m, 2H), 3.67 – 3.61 (m, 1H), 3.53 – 3.46 (m, 4H), 3.08 – 2.95 (m, 1H), 2.48 – 2.37 (m, 4H), 2.34 – 2.22 (m, 2H).
^{13}C NMR (101 MHz, CDCl$_3$): δ 155.2, 141.8, 136.7, 133.0, 129.2, 128.6, 128.4, 127.9, 127.8, 127.7, 127.2, 76.9, 73.6, 67.0, 50.8, 43.9, 36.1, 34.2.
HRMS (ESI): Calcd for $C_{25}H_{31}N_2O_3^+$ [M+H]$^+$: 407.2329, found 407.2330.

Benzyl (*E*)-4-(3-methyl-4-(oxetan-3-yl)but-2-en-1-yl)piperazine-1-carboxylate (63)

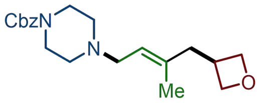

Prepared according to the **GP6** from benzyl piperazine-1-carboxylate (22.0 mg, 0.1 mmol) and 3-iodooxetane (27.6 mg, 0.15 mmol), isoprene (20.4 mg, 0.3 mmol), The crude residue was purified by column chromatography to yield **63** (31.0 mg, 90% yield, 10:1 *E/Z,* > 20:1 rr) as a colorless oil.

^{1}H NMR (500 MHz, CDCl$_3$): δ 7.39 – 7.27 (m, 5H), 5.24 – 5.09 (m, 3H), 4.82 – 4.71 (m, 2H), 4.42 – 4.31 (m, 2H), 3.56 – 3.46 (m, 4H), 3.19 – 2.90 (m, 2H), 2.85 – 2.77 (m, 1H), 2.49 – 2.21 (m, 6H), 1.82 – 1.57 (m, 3H).
^{13}C NMR (126 MHz, CDCl$_3$): δ 155.2, 155.1, 136.7, 136.7, 134.2, 134.1, 128.4, 127.9, 127.9, 127.8, 127.8, 125.4, 124.2, 121.0, 77.1, 67.1, 67.0, 67.0, 58.6, 55.8, 52.7, 52.6, 52.6, 43.8, 43.4, 35.1, 34.9, 33.1, 31.6, 22.9, 16.7, 15.2.
HRMS (ESI): Calcd for $C_{20}H_{29}N_2O_3^+$ [M+H]$^+$: 345.2173, found 345.2165.

Benzyl (*E*)-4-(7-methyl-3-(oxetan-3-ylmethyl)octa-2,6-dien-1-yl)piperazine-1-carboxylate (64)

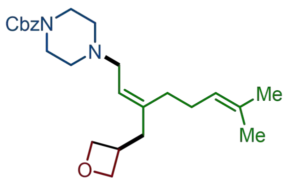

Prepared according to the **GP6** from benzyl piperazine-1-carboxylate (22.0 mg, 0.1 mmol) and 3-iodooxetane (27.6 mg, 0.15 mmol), 7-methyl-3-methyleneocta-1,6-diene (40.9 mg, 0.3 mmol), The crude residue was purified by column chromatography to yield **64** (19.4 mg, 47% yield, 6:1 *E/Z,* > 20:1 rr) as a colorless oil.

^{1}H NMR (500 MHz, CDCl$_3$): δ 7.39 – 7.28 (m, 5H), 5.12 (s, 2H), 5.08 – 5.01 (m, 2H), 4.82 – 4.71 (m, 2H), 4.39 – 4.31 (m, 2H), 3.60 – 3.44 (m, 4H), 3.19 – 3.10 (m, 1H), 3.06 – 2.92 (m, 2H), 2.47 – 2.31 (m, 6H), 2.08 – 1.89 (m, 4H), 1.67 (s, 3H), 1.59 (s, 3H).
^{13}C NMR (126 MHz, CDCl$_3$): δ 155.1, 140.5, 136.6, 132.1, 128.4, 127.9, 127.8, 123.5, 121.4, 77.3, 77.3, 67.0, 55.7, 52.8, 43.7, 40.7, 36.5, 33.1, 31.0, 26.8, 26.4, 25.6, 17.6.
HRMS (ESI): Calcd for $C_{26}H_{37}N_2O_3^+$ [M+H]$^+$: 413.2799, found 413.2791.

benzyl 4-((1*R*,4*R*)-4-(oxetan-3-yl)cyclohex-2-en-1-yl)piperazine-1-carboxylate (65)

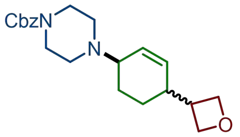

Prepared according to the **GP6** from benzyl piperazine-1-carboxylate (22.0 mg, 0.1 mmol) and 3-iodooxetane (27.6 mg, 0.15 mmol), cyclohexa-1,3-diene (24.0 mg, 0.3 mmol), The crude residue was purified by column chromatography to yield **65** (29.9 mg, 84% yield, 2:1 dr) as a colorless oil.

^{1}H NMR (500 MHz, CDCl$_3$, diastereomers): δ 7.38 – 7.23 (m, 5H), 5.73 – 5.57 (m, 2H), 5.10 (s, 2H), 4.79 – 4.67 (m, 2H), 4.53 – 4.36 (m, 2H), 3.56 – 3.41 (m, 4H), 3.27 – 3.08 (m, 1H), 2.89 – 2.70 (m, 1H), 2.58 – 2.36 (m, 4H), 2.14 – 1.74 (m, 2H), 1.64 – 0.92 (m, 3H).
^{13}C NMR (126 MHz, CDCl$_3$, diastereomers): δ 155.0, 136.6, 130.8, 130.4, 130.4, 130.2, 128.3, 127.8, 127.7, 76.2, 75.5, 75.3, 75.3, 66.9, 60.6, 59.1, 48.6, 48.1, 44.2, 39.7, 39.0, 38.9, 37.9, 25.7, 23.5, 21.9, 19.8.
HRMS (ESI): Calcd for $C_{21}H_{29}N_2O_3^+$ [M+H]$^+$: 357.2173, found 357.2164.

Benzyl (*E*)-4-(2,3-dimethyl-4-(oxetan-3-yl)but-2-en-1-yl)piperazine-1-carboxylate (66)

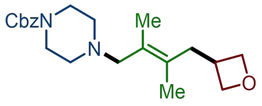

Prepared according to the **GP6** from benzyl piperazine-1-carboxylate (22.0 mg, 0.1 mmol) and 3-iodooxetane (27.6 mg, 0.15 mmol), 2,3-dimethylbuta-1,3-diene (24.6 mg, 0.3 mmol), The crude residue was purified by column chromatography to yield **66** (25.1 mg, 70% yield, 1.6:1 *E/Z,* > 20:1 rr) as a colorless oil.

^{1}H NMR (500 MHz, $CDCl_3$, *E/Z* mixture): δ 7.44 – 7.28 (m, 5H), 5.12 (d, *J* = 3.4 Hz, 2H), 4.79 – 4.68 (m, 2H), 4.46 – 4.31 (m, 2H), 3.52 – 3.41 (m, 4H), 3.20 – 3.06 (m, 1H), 2.96 (s, 1H), 2.88 (s, 1H), 2.52 – 2.44 (m, 2H), 2.36 – 2.24 (m, 4H), 1.75 (s, 2H), 1.67 (s, 1H), 1.63 – 1.57 (m, 3H).
^{13}C NMR (126 MHz, $CDCl_3$, *E/Z* mixture): δ 155.2, 136.7, 130.2, 130.0, 128.4, 127.9, 127.8, 126.9, 126.7, 77.5, 77.4, 66.9, 60.8, 60.5, 52.5, 43.9, 38.3, 37.4, 34.4, 34.2, 18.6, 18.0, 17.7, 17.7.
HRMS (ESI): Calcd for $C_{21}H_{31}N_2O_3^+$ $[M+H]^+$: 359.2329, found 359.2324.

(*E*)-4-Chloro-1-(4-(oxetan-3-yl)but-2-en-1-yl)piperidine (67)

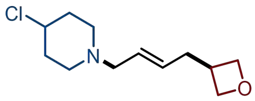

Prepared according to the **GP6** from 4-chloropiperidine (12.0 mg, 0.1 mmol) and 3-iodooxetane (27.6 mg, 0.15 mmol), buta-1,3-diene (150 μL, 0.3 mmol), The crude residue was purified by column chromatography to yield **67** (17.2 mg, 75% yield, 15:1 *E/Z,* > 20:1 rr) as a colorless oil.

^{1}H NMR (400 MHz, $CDCl_3$): δ 5.57 – 5.42 (m, 2H), 4.83 – 4.73 (m, 2H), 4.41 – 4.30 (m, 2H), 4.03 (s, 1H), 3.10 – 2.99 (m, 1H), 2.92 (d, *J* = 5.4 Hz, 2H), 2.76 – 2.65 (m, 2H), 2.45 – 2.38 (m, 2H), 2.27 – 2.02 (m, 4H), 1.96 – 1.84 (m, 2H).
^{13}C NMR (101 MHz, $CDCl_3$): δ 130.6, 128.1, 77.0, 60.6, 57.2, 51.1, 36.1, 35.4, 34.2.
HRMS (ESI): Calcd for $C_{12}H_{21}ClNO^+$ $[M+H]^+$: 230.1306, found 230.1302.

(*E*)-2-(4-(Oxetan-3-yl)but-2-en-1-yl)-1,2,3,4-tetrahydroisoquinoline (68)

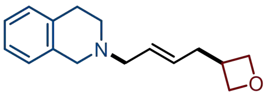

Prepared according to the **GP6** from 1,2,3,4-tetrahydroisoquinoline (13.3 mg, 0.1 mmol) and 3-iodooxetane (27.6 mg, 0.15 mmol), buta-1,3-diene (150 μL, 0.3 mmol), The crude residue was purified by column chromatography to yield **68** (18.0 mg, 74% yield, 19:1 *E/Z,* > 20:1 rr) as a colorless oil.

^{1}H NMR (400 MHz, $CDCl_3$): δ 7.14 – 6.98 (m, 4H), 5.67 – 5.52 (m, 2H), 4.84 – 4.76 (m, 2H), 4.43 – 4.36 (m, 2H), 3.59 (s, 2H), 3.15 – 3.05 (m, 3H), 2.94 – 2.87 (m,2H), 2.75 – 2.67 (m, 2H), 2.51 – 2.42 (m, 2H).
^{13}C NMR (101 MHz, $CDCl_3$): δ 134.6, 134.2, 130.4, 128.6, 128.4, 126.5, 126.1, 125.6, 77.1, 60.4, 55.9, 50.5, 36.2, 34.2, 29.0.

HRMS (ESI): Calcd for $C_{16}H_{22}NO^+$ $[M+H]^+$: 244.1696, found 244.1691.

Methyl (*E*)-(4-(oxetan-3-yl)but-2-en-1-yl)prolinate (69)

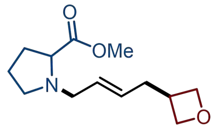

Prepared according to the **GP6** from methyl prolinate (12.9 mg, 0.1 mmol) and 3-iodooxetane (27.6 mg, 0.15 mmol), buta-1,3-diene (150 µL, 0.3 mmol), The crude residue was purified by column chromatography to yield **69** (19.4 mg, 81% yield, 15:1 *E/Z,* > 20:1 rr) as a colorless oil.

^{1}H NMR (500 MHz, $CDCl_3$): δ 5.61 – 5.42 (m, 2H), 4.80 – 4.72 (m, 2H), 4.37 – 4.28 (m, 2H), 3.70 (s, 3H), 3.28 – 2.96 (m, 5H), 2.48 – 2.28 (m, 3H), 2.16 – 2.06 (m, 1H), 1.96 – 1.73 (m, 3H).
^{13}C NMR (126 MHz, $CDCl_3$): δ 174.5, 130.2, 128.3, 77.0, 65.2, 56.8, 53.5, 51.8, 36.1, 34.1, 29.5, 23.0.
HRMS (ESI): Calcd for $C_{13}H_{22}NO_3^+$ $[M+H]^+$: 240.1594, found 240.1589.

(*E*)-*N*-Methyl-*N*-(naphthalen-1-ylmethyl)-4-(oxetan-3-yl)but-2-en-1-amine (70)

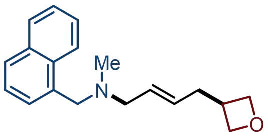

Prepared according to the **GP6** from *N*-methyl-1-(naphthalen-1-yl)methanamine (17.1 mg, 0.1 mmol) and 3-iodooxetane (27.6 mg, 0.15 mmol), buta-1,3-diene (150 µL, 0.3 mmol), The crude residue was purified by column chromatography to yield **70** (23.9 mg, 85% yield, 19:1 *E/Z,* > 20:1 rr) as a colorless oil.

^{1}H NMR (400 MHz, $CDCl_3$): δ 8.28 – 8.22 (m, 1H), 7.88 – 7.82 (m, 1H), 7.80 – 7.74 (m, 1H), 7.56 – 7.44 (m, 2H), 7.43 – 7.38 (m, 2H), 5.67 – 5.52 (m, 2H), 4.83 – 4.77 (m, 2H), 4.42 – 4.36 (m, 2H), 3.86 (s, 2H), 3.11 – 3.02 (m, 3H), 2.48 – 2.41 (m, 2H), 2.19 (s, 3H).
^{13}C NMR (101 MHz, $CDCl_3$): δ 134.8, 133.8, 132.4, 130.2, 129.1, 128.4, 127.9, 127.4, 125.8, 125.5, 125.1, 124.5, 77.0, 60.2, 59.8, 42.3, 36.2, 34.3.
HRMS (ESI): Calcd for $C_{19}H_{24}NO^+$ $[M+H]^+$: 282.1852, found 282.1846.

(*E*)-1-((4-Chlorophenyl)(phenyl)methyl)-4-(4-(oxetan-3-yl)but-2-en-1-yl)piperazine (71)

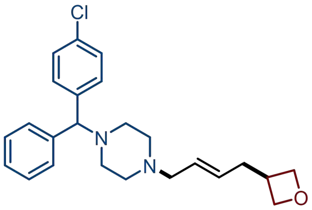

Prepared according to the **GP6** from 1-((4-chlorophenyl)(phenyl)methyl)piperazine hydrochloride (22.0 mg, 0.1 mmol) and 3-iodooxetane (32.3 mg, 0.15 mmol), buta-1,3-diene (150 μL, 0.3 mmol), The crude residue was purified by column chromatography to yield **71** (30.2 mg, 76% yield, 12:1 *E/Z,* > 20:1 rr) as a colorless oil.

^{1}H NMR (400 MHz, CDCl$_3$): δ 7.38 – 7.33 (m, 4H), 7.29 – 7.16 (m, 5H), 5.56 – 5.42 (m, 2H), 4.79 – 4.72 (m, 2H), 4.39 – 4.30 (m,2H), 4.20 (s, 1H), 3.08 – 2.97 (m, 1H), 2.93 (d, *J* = 5.3 Hz, 2H), 2.58 – 2.09 (m, 10H).
^{13}C NMR (101 MHz, CDCl$_3$): δ 142.1, 141.3, 132.4, 130.6, 129.1, 128.6, 128.5, 128.0, 127.8, 127.1, 76.9, 75.4, 60.7, 53.2, 51.7, 36.1, 34.1.
HRMS (ESI): Calcd for $C_{24}H_{30}ClN_2O^+$ [M+H]$^+$: 397.2041, found 397.2033.

(*E*)-*N*-Benzyl-4-(oxetan-3-yl)but-2-en-1-amine (72)

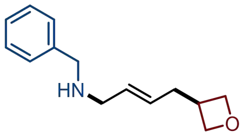

Prepared according to the **GP6** from phenylmethanamine (10.7 mg, 0.1 mmol) and 3-iodooxetane (27.6 mg, 0.15 mmol), buta-1,3-diene (150 μL, 0.3 mmol), The crude residue was purified by column chromatography to yield **72** (13.5 mg, 62% yield, 19:1 *E/Z,* > 20:1 rr) as a colorless oil.

^{1}H NMR (500 MHz, CDCl$_3$): δ 7.46 – 7.41 (m, 2H), 7.38 – 7.23 (m, 3H), 5.72 – 5.59 (m, 2H), 5.33 (brs, 1H), 4.83 – 4.75 (m, 2H), 4.39 – 4.31 (m, 2H), 3.90 (s, 2H), 3.33 (d, *J* = 5.0 Hz, 2H), 3.09 – 3.00 (m, 1H), 2.46 – 2.38 (m, 2H).
^{13}C NMR (126 MHz, CDCl$_3$): δ 135.0, 133.3, 129.3, 128.8, 128.3, 125.3, 76.8, 51.4, 49.2, 36.0, 33.9. **HRMS (ESI):** Calcd for $C_{14}H_{20}NO^+$ [M+H]$^+$: 218.1539, found 218.1537.

(*E*)-4-Methyl-N-(5-(oxetan-3-yl)pent-3-en-1-yl)aniline (73)

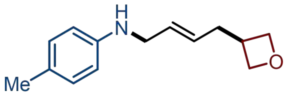

Prepared according to the **GP6** from *p*-toluidine (10.7 mg, 0.1 mmol) and 3-iodooxetane (27.6 mg, 0.15 mmol), buta-1,3-diene (150 μL, 0.3 mmol), The crude residue was purified by column chromatography to yield **73** (10.9 mg, 50% yield, 19:1 *E/Z,* > 20:1 rr) as a colorless oil.

^{1}H NMR (400 MHz, CDCl$_3$): δ 6.98 (d, *J* = 8.0 Hz, 2H), 6.54 (d, *J* = 8.1 Hz, 2H), 5.64 – 5.54 (m, 2H), 4.82 – 4.74 (m, 2H), 4.43 – 4.33 (m, 2H), 3.68 (d, *J* = 3.4 Hz, 2H), 3.10 – 2.98 (m, 1H), 2.50 – 2.38 (m, 2H), 2.24 (s, 3H).
^{13}C NMR (101 MHz, CDCl$_3$): δ 145.7, 129.6, 128.9, 128.9, 126.7, 113.2, 77.0, 46.2, 36.1, 34.2, 20.3.

HRMS (ESI): Calcd for $C_{14}H_{20}NO$ $[M+H]^+$: 218.1539, found 218.1540.

(1*R*)-3-((*E*)-4-(Oxetan-3-yl)but-2-en-1-yl)-1,2,3,4,5,6-hexahydro-8H-1,5-methano pyrido[1,2-a][1,5]diazocin-8-one (74)

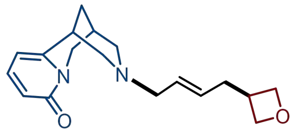

Prepared according to the **GP6** from 1,2,3,4,5,6-hexahydro-8H-1,5-methan opyrido[1,2-a][1,5]diazocin-8-one (19.0 mg, 0.1 mmol) and 3-iodooxetane (27.6 mg, 0.15 mmol), buta-1,3-diene (150 µL, 0.3 mmol), The crude residue was purified by column chromatography to yield **74** (23.1 mg, 77% yield, 19:1 *E/Z,* > 20:1 rr) as a colorless oil.

^{1}H NMR (400 MHz, CDCl$_3$): δ 7.31 – 7.20 (m, 1H), 6.40 (d, *J* = 9.0 Hz, 1H), 5.93 (d, *J* = 6.8 Hz, 1H), 5.40 – 5.14 (m, 2H), 4.78 – 4.63 (m, 2H), 4.32 – 4.22 (m, 2H), 4.05 – 3.94 (m, 1H), 3.90 – 3.79 (m, 1H), 3.12 – 2.72 (m, 5H), 2.40 (s, 1H), 2.33 – 2.27 (m, 2H), 2.25 – 2.18 (m, 2H), 1.88 – 1.69 (m, 2H), 1.42 – 1.35 (m, 1H).

^{13}C NMR (101 MHz, CDCl$_3$): δ 163.6, 151.5, 138.6, 129.6, 128.1, 116.5, 104.5, 76.8, 59.8, 49.9, 45.7, 35.9, 35.4, 34.1, 27.9, 25.8, 8.5.

HRMS (ESI): Calcd for $C_{18}H_{25}N_2O_2^+$ $[M+H]^+$: 301.1911, found 301.1905.

(3R,4S)-3-((benzo[d][1,3]dioxol-5-yloxy)methyl)-4-(4-fluorophenyl)-1-((E)-4-(oxet an-3-yl)but-2-en-1-yl)piperidine (75)

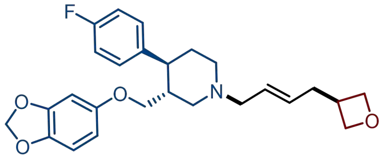

Prepared according to the **GP6** from (3R,4S)-3-((benzo[d][1,3]dioxol-5-yloxy) methyl)-4-(4-fluorophenyl)-1-((E)-4-(oxetan-3-yl)but-2-en-1-yl)piperidine (43.9 mg, 0.1 mmol) and 3-iodooxetane (27.6 mg, 0.15 mmol), buta-1,3-diene (150 µL, 0.3 mmol), The crude residue was purified by column chromatography to yield **75** (39.5 mg, 90% yield, 10:1 *E/Z,* > 20:1 rr) as a colorless oil.

^{1}H NMR (400 MHz, CDCl$_3$): δ 7.20 – 7.11 (m, 2H), 7.01 – 6.91 (m, 2H), 6.61 (d, *J* = 8.4 Hz, 1H), 6.32 (d, *J* = 2.5 Hz, 1H), 6.17 – 6.06 (m, 1H), 5.86 (s, 2H), 5.67 – 5.44 (m, 2H), 4.83 – 4.71 (m, 2H), 4.44 – 4.33 (m, 2H), 3.62 – 3.50 (m, 1H), 3.46 – 3.37 (m, 1H), 3.28 – 3.18 (m, 1H), 3.16 – 2.92 (m, 4H), 2.53 – 2.37 (m, 3H), 2.26 – 2.12 (m, 1H), 2.07 – 1.94 (m, 2H), 1.92 – 1.74 (m, 2H).

^{13}C NMR (101 MHz, CDCl$_3$): δ 161.4 (d, *J* = 244.3 Hz), 154.3, 148.1, 141.5, 139.5, 130.7, 128.7 (d, *J* = 7.7 Hz), 128.1, 115.3 (d, *J* = 21.1 Hz), 107.8, 105.4, 101.0, 97.8, 77.0, 69.4, 61.0, 57.3, 53.9, 44.0, 42.0, 36.2, 34.2, 34.2.

^{19}F NMR (376 MHz, CDCl$_3$) δ -116.5 (1F)

HRMS (ESI): Calcd for $C_{26}H_{31}FNO_4^+$ $[M+H]^+$: 440.2231 , found 440.2215.

(R,E)-*N*-(1-(naphthalen-1-yl)ethyl)-4-(oxetan-3-yl)-N-(3-(4-(trifluoromethyl)phen yl) propyl)but-2-en-1-amine (76)

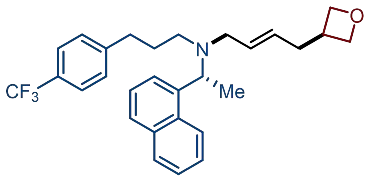

Prepared according to the **GP6** from 3-((benzo[d][1,3]dioxol-5-yloxy)methyl)-4-(4-(trifluoromethyl)phenyl)piperidine (37.9 mg, 0.1 mmol) and 3-iodooxetane (27.6 mg, 0.15 mmol), buta-1,3-diene (150 μL, 0.3 mmol), The crude residue was purified by column chromatography to yield **76** (44.0 mg, 92% yield, >20:1 *E/Z,* > 20:1 rr) as a colorless oil.

^{1}H NMR (400 MHz, CDCl$_3$): δ 8.42 (d, *J* = 8.2 Hz, 1H), 7.90 – 7.82 (m, 1H), 7.75 (d, *J* = 8.1 Hz, 1H), 7.58 – 7.45 (m, 3H), 7.45 – 7.35 (m, 2H), 7.31 – 7.23 (m, 2H), 7.08 (d, *J* = 7.7 Hz, 1H), 5.61 – 5.49 (m, 1H), 5.49 – 5.39 (m, 1H), 4.81 – 4.70 (m, 2H), 4.69 – 4.53 (m, 1H), 4.41 – 4.28 (m, 2H), 3.32 – 3.11 (m, 2H), 3.07 – 2.92 (m, 1H), 2.67 – 2.46 (m, 2H), 2.46 – 2.29 (m, 4H), 1.70 – 1.61 (m, 2H), 1.45 (d, *J* = 6.6 Hz, 3H).
^{13}C NMR (101 MHz, CDCl$_3$): δ 143.4, 140.4, 134.0, 132.0, 131.6, 130.4 (q, *J* = 32.2 Hz), 129.6, 129.2, 128.6, 128.5, 127.4, 125.6 (q, *J* = 272.1 Hz), 125.3, 125.1, 124.8 (q, *J* = 3.7 Hz), 124.5, 122.4 (q, *J* = 1.0 Hz), 77.2, 56.5, 52.9, 48.9, 36.2, 34.3, 33.2, 28.9, 15.7.
^{19}F NMR (376 MHz, CDCl$_3$) δ -62.5 (3F).
HRMS (ESI): Calcd for $C_{29}H_{33}F_3NO^+$ $[M+H]^+$: 468.2508 , found 468.2513.

(S,E)-*N*-Methyl-*N*-(3-(naphthalen-1-yloxy)-3-(thiophen-2-yl)propyl)-4-(oxetan-3-yl)but-2-en-1-amine (77)

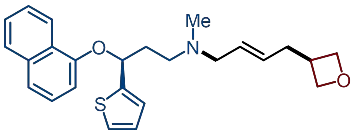

Prepared according to the **GP6** from N-methyl-3-(naphthalen-1-yloxy)-3-(thiophen -2-yl)propan-1-amine hydrochloride (33.3 mg, 0.1 mmol) and 3-iodooxetane (27.6 mg, 0.15 mmol), buta-1,3-diene (150 μL, 0.3 mmol), The crude residue was purified by column chromatography to yield **77** (32.1 mg, 79% yield, 7:1 *E/Z,* > 20:1 rr) as a colorless oil.

^{1}H NMR (400 MHz, CDCl$_3$, *E/Z* mixture): δ 8.34 – 8.27 (m, 1H), 7.78 – 7.71 (m, 1H), 7.50 – 7.42 (m, 2H), 7.36 (d, 1H), 7.26 – 7.20 (m, 1H), 7.17 (d, 1H), 7.03 (d, 1H), 6.94 – 6.88 (m, 1H), 6.83 (d, *J* = 7.7 Hz, 1H), 5.79 – 5.70 (m, 1H), 5.45 – 5.27 (m, 2H), 4.73 – 4.59 (m, 2H), 4.29 – 4.19 (m, 2H), 2.98 – 2.86 (m, 2H), 2.84 – 2.75 (m, 1H), 2.66 – 2.29 (m, 4H), 2.22 – 2.15 (m, 5H).

^{13}C NMR (101 MHz, CDCl$_3$, *E/Z* mixture): δ 153.4, 145.3, 134.5, 130.2, 128.4, 127.4, 126.5, 126.3, 126.1, 125.7, 125.1, 124.6, 124.5, 122.1, 120.5, 106.8, 77.0, 74.3, 60.0, 52.7, 42.1, 36.7, 36.0, 34.1.
HRMS (ESI): Calcd for $C_{25}H_{30}NO_2S^+$ [M+H]$^+$: 408.1991, found 408.1986.

(*E*)-*N*-(((2S,4aR,10aR)-7-isopropyl-2,4a-dimethyl-1,2,3,4,4a,9,10,10a-octahydrophenanthren-2-yl)methyl)-4-(oxetan-3-yl)but-2-en-1-amine (78)

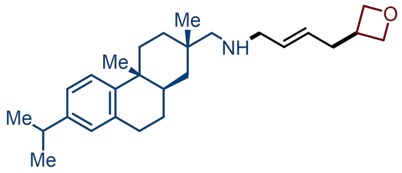

Prepared according to the **GP6** from benzyl piperazine-1-carboxylate (22.0 mg, 0.1 mmol) and 3-iodooxetane (27.6 mg, 0.15 mmol), buta-1,3-diene (150 µL, 0.3 mmol), The crude residue was purified by column chromatography to yield **78** (15.8 mg, 40% yield, 19:1 *E/Z,* > 20:1 rr) as a colorless oil.

^{1}H NMR (500 MHz, CDCl$_3$): δ 7.18 (d, *J* = 7.9 Hz, 1H), 6.99 (d, *J* = 7.4 Hz, 1H), 6.89 (s, 1H), 5.57 – 5.43 (m, 2H), 4.82 – 4.75 (m, 2H), 4.45 – 4.32 (m, 2H), 3.17 (s, 2H), 3.09 – 2.99 (m, 1H), 2.95 – 2.88 (m, 1H), 2.88 – 2.80 (m, 2H), 2.50 – 2.45 (m, 1H), 2.43 – 2.39 (m, 2H), 2.31 – 2.25 (m, 2H), 1.80 – 1.72 (m, 3H), 1.68 – 1.62 (m, 1H), 1.61 – 1.56 (m, 1H), 1.44 – 1.38 (m, 3H), 1.26 – 1.22 (m, 10H), 0.96 – 0.91 (m, 3H).
^{13}C NMR (126 MHz, CDCl$_3$): δ 147.5, 145.4, 134.7, 130.5, 128.3, 126.7, 124.2, 123.7, 77.0, 60.9, 52.4, 45.4, 38.4, 37.4, 36.9, 36.2, 34.3, 33.4, 30.2, 25.3, 23.9, 19.2, 18.8, 18.7.
HRMS (ESI): Calcd for $C_{27}H_{42}NO^+$ [M+H]$^+$: 396.3261, found 396.3263.

(*E*)-4-(Oxetan-3-yl)but-2-en-1-yl benzoate (79)

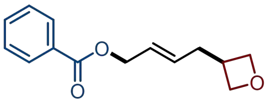

Prepared according to the **GP6** from benzoic acid (12.2 mg, 0.1 mmol) and 3-iodooxetane (27.6 mg, 0.15 mmol), buta-1,3-diene (150 µL, 0.3 mmol), The crude residue was purified by column chromatography to yield **79** (14.6 mg, 63% yield, 10:1 *E/Z,* > 20:1 rr) as a colorless oil.

^{1}H NMR (500 MHz, CDCl$_3$): δ 8.08 – 8.01 (m, 2H), 7.61 – 7.53 (m, 1H), 7.49 – 7.40 (m, 2H), 5.83 – 5.64 (m, 2H), 4.86 – 4.69 (m, 4H), 4.43 – 4.34 (m, 2H), 3.17 – 3.02 (m, 1H), 2.54 – 2.42 (m, 2H).
^{13}C NMR (126 MHz, CDCl$_3$): δ 166.3, 133.0, 132.3, 129.6, 128.3, 125.6, 77.0, 65.3, 36.1, 34.0.
HRMS (ESI): Calcd for $C_{14}H_{17}O_3^+$ [M+H]$^+$: 233.1172, found 233.1169.

(*E*)-4-(Oxetan-3-yl)but-2-en-1-yl 4-methoxybenzoate (80)

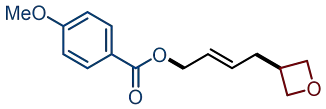

Prepared according to the **GP6** from 4-methoxybenzoic acid (15.2 mg, 0.1 mmol) and 3-iodooxetane (27.6 mg, 0.15 mmol), buta-1,3-diene (150 µL, 0.3 mmol), The crude residue was purified by column chromatography to yield **80** (19.1 mg, 73% yield, 12:1 *E/Z,* > 20:1 rr) as a colorless oil.

^{1}H NMR (400 MHz, $CDCl_3$): δ 7.99 (d, *J* = 8.9 Hz, 2H), 6.91 (d, *J* = 8.9 Hz, 2H), 5.83 – 5.58 (m, 2H), 4.84 – 4.78 (m, 2H), 4.72 (d, *J* = 5.9 Hz, 2H), 4.42 – 4.35 (m, 2H), 3.86 (s, 3H), 3.13 – 3.03 (m, 1H), 2.51 – 2.42 (m, 2H).
^{13}C NMR (101 MHz, $CDCl_3$): δ 166.1, 163.3, 131.9, 131.6, 125.8, 122.6, 113.6, 77.0, 65.0, 55.4, 36.1, 34.0.
HRMS (ESI): Calcd for $C_{15}H_{19}O_4^+$ $[M+H]^+$: 263.1278, found 263.1274.

(*E*)-4-(Oxetan-3-yl)but-2-en-1-yl 4-chlorobenzoate (81)

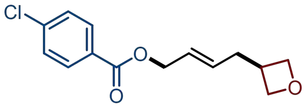

Prepared according to the **GP6** from 4-chlorobenzoic acid (15.7 mg, 0.1 mmol) and 3-iodooxetane (27.6 mg, 0.15 mmol), buta-1,3-diene (150 µL, 0.3 mmol), The crude residue was purified by column chromatography to yield **81** (13.9 mg, 52% yield, 10:1 *E/Z,* > 20:1 rr) as a colorless oil.

^{1}H NMR (500 MHz, $CDCl_3$): δ 7.97 (d, *J* = 8.4 Hz, 2H), 7.40 (d, *J* = 8.4 Hz, 2H), 5.83 – 5.61 (m, 2H), 4.83 – 4.72 (m, 4H), 4.40 – 4.35 (m, 2H), 3.13 – 3.02 (m, 1H), 2.52 – 2.42 (m, 2H).
^{13}C NMR (126 MHz, $CDCl_3$): δ 165.4, 139.4, 132.6, 131.0, 128.7, 128.6, 125.4, 76.9, 65.5, 36.1, 34.0.
HRMS (ESI): Calcd for $C_{14}H_{16}ClO_3^+$ $[M+H]^+$: 267.0782, found 267.0788.

(*E*)-4-(Oxetan-3-yl)but-2-en-1-yl 3-methoxy-5-(trifluoromethyl)benzoate (82)

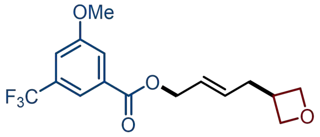

Prepared according to the **GP6** from 3-methoxy-5-(trifluoromethyl)benzoic acid (22.0 mg, 0.1 mmol) and 3-iodooxetane (27.6 mg, 0.15 mmol), buta-1,3-diene (150 µL, 0.3 mmol), The crude residue was purified by column chromatography to yield **82** (19.5 mg, 59% yield, 8:1 *E/Z,* > 20:1 rr) as a colorless oil.

^{1}H NMR (400 MHz, $CDCl_3$): δ 7.85 (s, 1H), 7.70 (s, 1H), 7.29 (s, 1H), 5.86 – 5.59 (m, 2H), 4.84 – 4.72 (m, 4H), 4.43 – 4.32 (m, 2H), 3.87 (s, 3H), 3.16 – 3.01 (m, 1H),

2.63 – 2.44 (m, 2H).
^{13}C NMR (101 MHz, CDCl$_3$): δ 164.9, 159.8, 132.9, 132.4, 132.03 (q, *J* = 32.9 Hz), 125.1, 123.40 (q, *J* = 272.7 Hz), 118.5 (q, *J* = 3.8 Hz), 117.7, 115.63 (q, *J* = 3.6 Hz), 76.8, 65.8, 55.7, 36.0, 33.9.
^{19}F NMR (376 MHz, CDCl$_3$): δ -62.8 (3F).
HRMS (ESI): Calcd for $C_{16}H_{18}F_3O_4^+$ [M+H]$^+$: 331.1152, found 331.1143.

(*E*)-4-(Oxetan-3-yl)but-2-en-1-yl 3-methoxypropanoate (83)

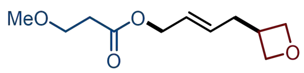

Prepared according to the **GP6** from 3-methoxypropanoic acid (10.4 mg, 0.1 mmol) and 3-iodooxetane (27.6 mg, 0.15 mmol), buta-1,3-diene (150 µL, 0.3 mmol), The crude residue was purified by column chromatography to yield **83** (16.3 mg, 76% yield, 9:1 *E/Z,* > 20:1 rr) as a colorless oil.
^{1}H NMR (500 MHz, CDCl$_3$): δ 5.76 – 5.49 (m, 2H), 4.79 (t, *J* = 6.7 Hz, 2H), 4.53 (d, *J* = 5.9 Hz, 2H), 4.36 (t, *J* = 5.9 Hz, 2H), 3.65 (t, *J* = 6.1 Hz, 2H), 3.35 (s, 3H), 3.09 – 3.01 (m, 1H), 2.58 (t, *J* = 6.1 Hz, 2H), 2.44 (t, *J* = 6.6 Hz, 2H).
^{13}C NMR (126 MHz, CDCl$_3$): δ 171.3, 132.2, 125.5, 76.9, 67.9, 64.9, 58.8, 36.1, 35.0, 34.0.
HRMS (ESI): Calcd for $C_{11}H_{19}O_4^+$ [M+H]$^+$: 215.1278, found 215.1279.

(*E*)-4-(Oxetan-3-yl)but-2-en-1-yl 3-(methylsulfonyl)benzoate (84)

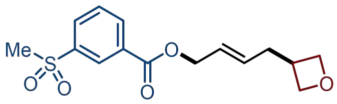

Prepared according to the **GP6** from 3-(methylsulfonyl)benzoic acid (20.0 mg, 0.1 mmol) and 3-iodooxetane (27.6 mg, 0.15 mmol), buta-1,3-diene (150 µL, 0.3 mmol), The crude residue was purified by column chromatography to yield **84** (15.5 mg, 50% yield, 10:1 *E/Z,* > 20:1 rr) as a colorless oil.

^{1}H NMR (500 MHz, CDCl$_3$): δ 8.57 (s, 1H), 8.30 (d, *J* = 7.6 Hz, 1H), 8.12 (d, *J* = 7.5 Hz, 1H), 7.76 – 7.59 (m, 1H), 5.82 – 5.59 (m, 2H), 4.85 – 4.73 (m, 4H), 4.40 – 4.31 (m, 2H), 3.11 – 3.03 (m, 4H), 2.51 – 2.41 (m, 2H).
^{13}C NMR (126 MHz, CDCl$_3$): δ 164.4, 141.1, 134.5, 133.3, 131.7, 131.3, 129.6, 128.5, 124.9, 76.8, 66.0, 44.3, 36.0, 33.8.
HRMS (ESI): Calcd for $C_{15}H_{19}O_5S^+$ [M+H]$^+$: 311.0948, found 311.0942.

(*E*)-4-(Oxetan-3-yl)but-2-en-1-yl 2-(4-isobutylphenyl)propanoate (85)

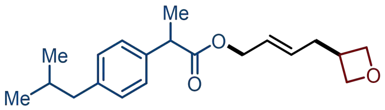

Prepared according to the **GP6** from 2-(4-isobutylphenyl)propanoic acid (20.6 mg, 0.1 mmol) and 3-iodooxetane (27.6 mg, 0.15 mmol), buta-1,3-diene (150 µL, 0.3

mmol), The crude residue was purified by column chromatography to yield **85** (23.7 mg, 75% yield, 10:1 *E/Z,* > 20:1 rr) as a colorless oil.

^{1}H NMR (500 MHz, CDCl$_3$): δ 7.19 (d, *J* = 7.4 Hz, 2H), 7.09 (d, *J* = 7.5 Hz, 2H), 5.61 – 5.41 (m, 2H), 4.75 (t, *J* = 6.7 Hz, 2H), 4.50 (d, *J* = 5.4 Hz, 2H), 4.32 (t, *J* = 5.9 Hz, 2H), 3.73 – 3.63 (m, 1H), 3.06 – 2.93 (m, 1H), 2.50 – 2.34 (m, 4H), 1.89 – 1.78 (m, 1H), 1.48 (d, *J* = 7.0 Hz, 3H), 0.89 (d, *J* = 6.4 Hz, 6H).
^{13}C NMR (126 MHz, CDCl$_3$): δ 174.4, 140.5, 137.6, 131.7, 129.3, 127.1, 125.5, 76.9, 64.8, 45.1, 45.0, 36.0, 33.9, 30.2, 22.3, 18.5.
HRMS (ESI): Calcd for $C_{20}H_{29}O_3^+$ [M+H]$^+$: 317.2111, found 317.2107.

(*E*)-4-(Oxetan-3-yl)but-2-en-1-yl 3-(4,5-diphenyloxazol-2-yl)propanoate (86)

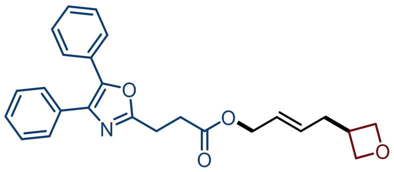

Prepared according to the **GP6** from 3-(4,5-diphenyloxazol-2-yl)propanoic acid (29.3 mg, 0.1 mmol) and 3-iodooxetane (27.6 mg, 0.15 mmol), buta-1,3-diene (150 µL, 0.3 mmol), The crude residue was purified by column chromatography to yield **86** (21.8 mg, 54% yield, 9:1 *E/Z,* > 20:1 rr) as a colorless oil.

^{1}H NMR (500 MHz, CDCl$_3$): δ 7.66 – 7.61 (m, 2H), 7.59 – 7.55 (m, 2H), 7.39 – 7.29 (m, 6H), 5.83 – 5.43 (m, 2H), 4.76 (t, *J* = 6.8 Hz, 2H), 4.56 (d, *J* = 5.8 Hz, 2H), 4.33 (t, *J* = 5.9 Hz, 2H), 3.18 (t, *J* = 7.2 Hz, 2H), 3.03 – 2.96 (m, 1H), 2.92 (t, *J* = 7.3 Hz, 2H), 2.39 (t, *J* = 6.6 Hz, 2H).
^{13}C NMR (126 MHz, CDCl$_3$): δ 171.6, 161.6, 145.3, 135.0, 132.3, 132.2, 128.9, 128.5, 128.4, 128.4, 128.0, 127.8, 126.3, 125.3, 76.8, 65.0, 36.0, 33.9, 31.0, 23.4.
HRMS (ESI): Calcd for $C_{25}H_{26}NO_4^+$[M+H]$^+$: 404.1856, found 404.1858.

4-Methyl-N-(2-phenylhexa-2,5-dien-1-yl)aniline (90)

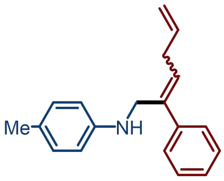

Prepared according to the **GP5** from *p*-toluidine (10.7 mg, 0.1 mmol) and (1-(2-(iodomethyl)cyclopropyl)vinyl)benzene (42.6 mg, 0.15 mmol), The crude residue was purified by column chromatography to yield **90** (24.7 mg, 94% yield, 1:1 E/Z) as a colorless oil.

^{1}H NMR (400 MHz, CDCl$_3$, *E/Z* mixture): δ 7.48 (d, *J* = 7.2 Hz, 1H), 7.44 – 7.29 (m, 3H), 7.29 – 7.20 (m, 1H), 7.09 – 6.98 (m, 2H), 6.63 – 6.54 (m, 2H), 6.09 – 5.88 (m, 1H), 5.91 – 5.74 (m, 1H), 5.21 – 5.08 (m, 1H), 5.07 – 4.96 (m, 1H), 4.14 (s, 1H),

4.02 (s, 1H), 3.88 – 3.38 (m, 1H), 3.14 – 3.05 (m, 1H), 2.83 – 2.72 (m, 1H), 2.29 (d, *J* = 5.2 Hz, 3H).
^{13}C NMR (101 MHz, $CDCl_3$, *E/Z* mixture) δ 145.9, 145.7, 141.1, 139.0, 139.0, 137.9, 136.9, 136.3, 129.7, 129.6, 129.8, 128.4, 128.3, 128.3, 127.2, 127.1, 126.7, 126.5, 126.1, 125.7, 51.3, 43.4, 32.9, 32.8, 20.4.
HRMS (ESI): Calcd for $C_{19}H_{22}N^+$ $[M+H]^+$: 264.1746 , found 264.1738.

Benzyl 4-allylpiperazine-1-carboxylate (91)

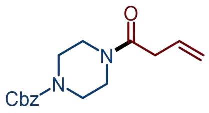

Prepared according to the **GP5** from benzyl piperazine-1-carboxylate (22.0 mg, 0.1 mmol) and 4-(iodomethyl)oxetan-2-one (31.8 mg, 0.15 mmol), The crude residue was purified by column chromatography to yield **91** (5.5 mg, 19% yield) as a colorless oil.

^{1}H NMR (500 MHz, $CDCl_3$) δ 7.40 – 7.30 (m, 5H), 5.99 – 5.87 (m, 1H), 5.21 – 5.10 (m, 4H), 3.64 – 3.56 (m, 2H), 3.54 – 3.48 (m, 4H), 3.47 – 3.39 (m, 2H), 3.18 – 3.13 (m, 2H).
^{13}C NMR (126 MHz, $CDCl_3$) δ 169.5, 155.1, 136.3, 131.1, 128.5, 128.2, 128.0, 118.1, 67.4, 45.5,43.6, 41.3, 38.7.
HRMS (ESI): Calcd for $C_{16}H_{21}N_2O_3^+$ $[M+H]^+$: 289.1547 , found 289.1533.

Benzyl (Z)-4-((3-(3-methylbut-1-en-1-yl)cyclopent-1-en-1-yl)methyl)piperazine -1-car boxylate (92)

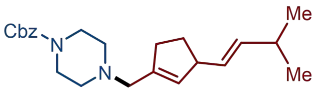

Prepared according to the **GP5** from benzyl piperazine-1-carboxylate (22.0 mg, 0.1 mmol) and 6-(iodomethyl)-2-methylenebicyclo[3.1.0]hexane (35.1 mg, 0.15 mmol), The crude residue was purified by column chromatography to yield **92** (31.6 mg, 85% yield, >20:1 *E/Z*) as a colorless oil.

^{1}H NMR (400 MHz, $CDCl_3$): δ 7.38 (d, *J* = 4.4 Hz, 4H), 7.38 – 7.30 (m, 1H), 5.48 – 5.42 (m, 1H), 5.43 – 5.25 (m, 2H), 5.16 (s, 2H), 3.58 – 3.50 (m, 4H), 3.26 (d, *J* = 7.7 Hz, 1H), 3.03 (s, 2H), 2.38 (s, 5H), 2.30 – 2.21 (m, 2H), 2.19 – 2.11 (m, 1H), 1.64 – 1.55 (m, 1H), 0.98 (d, *J* = 6.7 Hz, 6H).
^{13}C NMR (101 MHz, $CDCl_3$): δ 155.3, 141.2, 136.8, 136.3, 132.0, 130.8, 128.5, 128.0, 127.9, 67.1, 59.1, 53.0, 48.3, 43.9, 33.7, 31.5, 30.8, 22.7.
HRMS (ESI): Calcd for $C_{23}H_{33}N_2O_2^+$ $[M+H]^+$: 369.2536 , found 369.2523.

Benzyl 4-(5-hydroxy-2-methylenepentyl)piperazine-1-carboxylate (93)

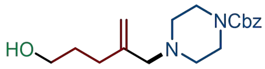

Prepared according to a known procedure[2]. In a nitrogen-filled glovebox, 10 mL dried vial was charged with stir bar, 9-BBN-H (0.9 mL, 0.5 M in THF, 1.1 equiv.), 1,4-dioxane (0.8 mL, 0.5 M) and compound **5** (120.2 mg, 0.4 mmol, 1.0 equiv.). The reaction was stirred overnight at 60°C. The solvent was removed under reduced pressure. To the resulting reaction mixture was added a solution of $NaBO_3{\cdot}H_2O$ (1.3 mmol, 3.3 equiv.) in THF:H_2O (0.8 mL, 1:1, 0.5 M). The reaction mixture was stirred at room temperature for 12 hours. The reaction mixture was dilute with water and extracted with diethyl ether. The combined organic phase was dried with Na_2SO_4 and concentrated under reduced pressure. The crude product was purified by column chromatography to yield **93** (66.2 mg, 52%) as a colorless oil.

^{1}H NMR (500 MHz, $CDCl_3$) δ 7.41 – 7.28 (m, 1H), 5.12 (s, 1H), 4.96 – 4.91 (m, 1H), 3.62 (t, *J* = 6.3 Hz, 1H), 3.54 – 3.46 (m, 2H), 2.91 (s, 1H), 2.37 (s, 1H), 2.17 (t, *J* = 7.3 Hz, 1H), 1.72 (t, *J* = 7.0 Hz, 1H).
^{13}C NMR (126 MHz, $CDCl_3$) δ 155.2, 145.1, 136.6, 128.4, 127.9, 127.8, 113.7, 67.0, 64.4, 61.9, 52.8, 43.6, 30.6, 30.0.
HRMS (ESI): Calcd for $C_{18}H_{27}N_2O_3^+$ $[M+H]^+$: 319.2016, found 319.2010.

Benzyl 4-(5-(4-(ethoxycarbonyl)phenyl)-2-methylenepentyl)piperazine-1-carboxylate (94)

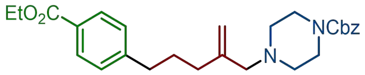

Prepared according to a known procedure[2]. In a nitrogen-filled glovebox, 10 mL dried reaction tube was charged with stir bar, 9-BBN-H (0.9 mL, 0.5 M in THF, 1.1 equiv.), 1,4-dioxane (0.8 mL, 0.5 M) and compound **5** (120.2 mg, 0.4 mmol, 1.0 equiv.). The reaction was stirred overnight at 60°C. To the reaction mixture as added $Pd(OAc)_2$ (0.9 mg, 0.004 mmol, 0.01 equiv.), tricyclohexylphosphine (2.2 mg, 0.008 mmol, 0.02 equiv.), Cs_2CO_3 (391.0 mg, 1.2 mmol, 3.0 equiv.) and ethyl 4-bromobenzoate (100.8 mg, 0.44 mmol, 1.1 equiv.) respectively. The reaction mixture was stirred at 100°C for 24 hours. The reaction mixture was dilute with ethyl acetate and filltered through a plug of silica. The solvent was removed under reduced pressure and the reaction mixture was stirred at room temperature for 12 hours. The reaction mixture was dilute with water and extracted with diethyl ether. The combined organic phase was dried with Na_2SO_4 and concentrated under reduced pressure. The crude product was purified by column chromatography to yield **94** (129.8 mg, 72%) as a colorless oil.

^{1}H NMR (500 MHz, $CDCl_3$) δ 7.87 (d, *J* = 8.0 Hz, 2H), 7.29 – 7.20 (m, 5H), 7.15 (d, *J* = 8.0 Hz, 2H), 5.04 (s, 2H), 4.87 – 4.76 (m, 2H), 4.27 (q, *J* = 7.1 Hz, 2H), – 3.36 (m, 4H), 2.78 (s, 2H), 2.58 (t, *J* = 7.7 Hz, 2H), 2.30 – 2.17 (m, 4H), 2.00 (t, *J* = 7.5 Hz, 2H), 1.76 – 1.63 (m, 2H), 1.29 (t, *J* = 7.1 Hz, 3H).
^{13}C NMR (126 MHz, $CDCl_3$) δ 166.6, 155.2, 147.8, 145.4, 136.7, 129.6, 128.4, 128.3, 128.0, 127.9, 127.8, 112.8, 67.0, 63.9, 60.7, 52.8, 43.8, 35.5, 33.6, 28.9, 14.3.
HRMS (ESI): Calcd for $C_{27}H_{35}N_2O_4^+$ $[M+H]^+$: 451.2591, found 451.2582.

Benzyl 4-(2-(2-methylenepent-4-en-1-yl)phenyl)piperazine-1-carboxylate (95)

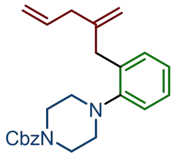

Prepared according to a known procedure[3]. 10 mL dired vial was charged stir bar, *o*-trimethylsilylphenyl triflate (59.7 mg, 0.2 mmo, 1.0 equiv.), CsF (91.1 mg, 0.6 mmol, 3.0 equiv.) Toluene/MeCN (3/1, 1 mL) and compound **5** (90.2 mg, 0.3 mmol, 1.5 equiv.). The reaction was stirred at 110°C for 48 hours. The reaction mixture was then diluted with ethyl acetate (3 mL). Then the combined organic layers washed with brine, dried with Na_2SO_4, and concentrated. The resulting residue was purified by silica gel chromatography to yield **95** (48.9 mg, 68% yield) as colorless oil.

^{1}H NMR (400 MHz, $CDCl_3$) δ 7.43 – 7.31 (m, 5H), 7.25 – 7.19 (m, 2H), 7.12 – 7.05 (m, 2H), 5.92 – 5.78 (m, 1H), 5.18 (s, 2H), 5.09 (s, 1H), 5.05 (d, *J* = 5.7 Hz, 1H), 4.88 (s, 1H), 4.68 (s, 1H), 3.67 – 3.61 (m, 4H), 3.46 (s, 2H), 2.90 – 2.81 (m, 4H), 2.76 (d, *J* = 6.8 Hz, 2H).
^{13}C NMR (101 MHz, $CDCl_3$) δ 155.3, 151.4, 147.8, 136.7, 136.3, 134.5, 131.0, 128.5, 128.0, 127.9, 127.1, 124.1, 120.2, 116.3, 111.7, 67.1, 52.4, 44.4, 40.8, 36.9.
HRMS (ESI): Calcd for $C_{24}H_{29}N_2O_2^+$ $[M+H]^+$: 377.2224, found 377.2215.

Benzyl 4-(2-methyl-4-methylenehept-6-enoyl)piperazine-1-carboxylate (96)

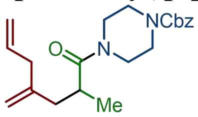

Prepared according to a known procedure[4]. 10 mL dired vial was charged stir bar, compound **5** (120.2 mg, 0.4 mmol, 1.0 equiv.), $Sc(OTf)_3$ (393.7 mg, 0.8 mmol, 2.0 equiv.), *N*,*N*-diisopropylethylamine (174 μL, 1.0 mmol, 2.5 equiv.), propionyl chloride (55.5 mg, 0.6 mmol, 1.5 equiv.) and DCM (2 mL). The reaction was stired at room temperature for 24 hours. The reaction mixture was then diluted with ethyl acetate (10 mL) and washed with aqueous 1N NaOH (5 mL). The aqueous layer was then extracted with ethyl acetate, and the combined organic layers washed with brine, dried with Na_2SO_4, and concentrated. The resulting residue was purified by silica gel chromatography to yield **96** (78.4 mg, 55% yield) as colorless oil.

^{1}H NMR (400 MHz, $CDCl_3$) δ 7.43 – 7.31 (m, 5H), 5.88 – 5.72 (m, 1H), 5.14 (s, 2H), 5.08 – 5.01 (m, 2H), 4.82 (s, 1H), 4.75 (s, 1H), 3.65 (s, 1H), 3.58 – 3.43 (m, 8H), 2.88 (d, *J* = 6.7 Hz, 1H), 2.78 – 2.72 (m, 2H), 2.48 – 2.38 (m, 1H), 2.14 – 2.04 (m, 1H).
^{13}C NMR (101 MHz, $CDCl_3$) δ 174.8, 155.1, 145.3, 136.3, 136.1, 128.5, 128.2, 128.0, 116.5, 112.1, 67.4, 45.2, 44.0, 43.7, 41.4, 41.0, 39.8, 33.4, 17.4.
HRMS (ESI): Calcd for $C_{21}H_{29}N_2O_3^+$ $[M+H]^+$: 357.2173, found 357.2166.

4. Mechanistic Studies

4.1 UV-vis absorption of in-situ generated copper complex

The UV-vis spectra of single component (1.25×10^{-4} M) was measured directly in DMA. A mixture of components (the amount of component is equal to each other) was stirred for 30 min before measurement in 10 mm pathlength quartz cuvette.

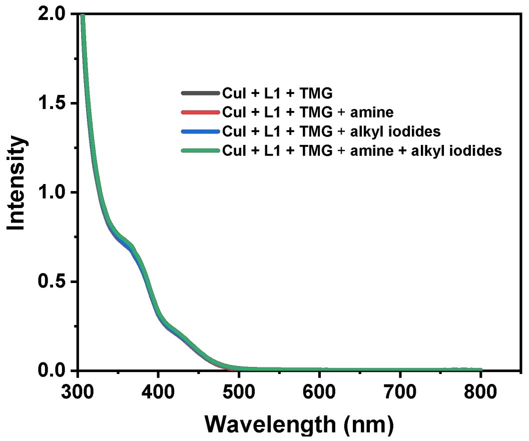

Supplementary Figure 3. UV-vis absorption spectra of reaction complexes in DMA

4.2 Transient absorption experiments

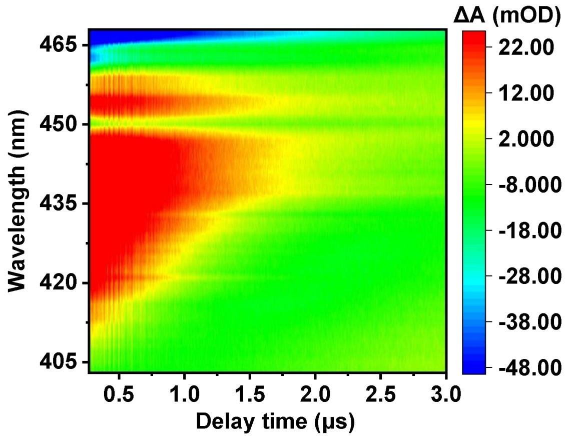

Supplementary Figure 4. Two-dimensional pseudo-colour TA spectra of in-situ generated copper complex (λ_{pump} = 400 nm) measured by ultrafast transient absorption (TA)

4.3 Lifetime quenching experiments

Experimental Procedure: Rigorously degassed solutions of each component were prepared under atmosphere prior to each set of experiments. In a typical experiments, a 0.5 mM solution of in situ generated copper complex: CuI (1.0 equiv.), L1 (1.0 equiv.) and TMG (1.5 equiv.) in DMA was added in increasing amounts (0 mM to 19.2 mM) of quencher (1-(iodomethyl)-3-methylenecyclobutane) in a 1 mm quartz

cuvette. The lifetime of non-emissive excited state of copper complex complex as function of electrophile concentration was measured at room temperature by transient absorption spectroscopy.

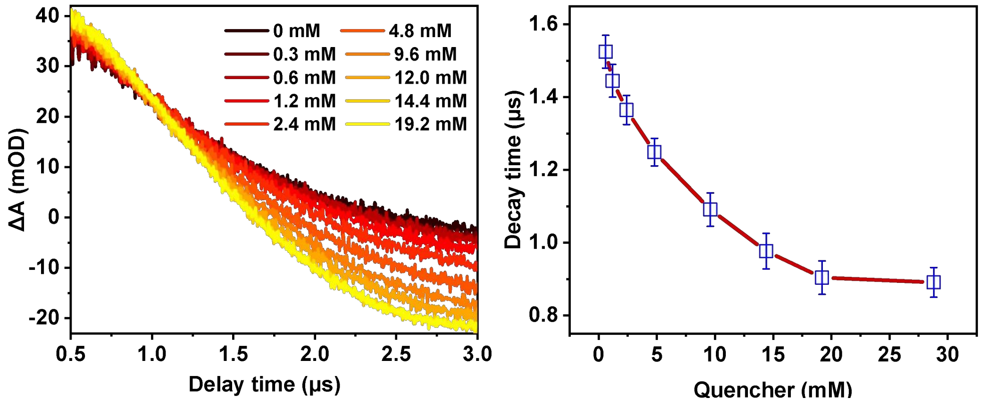

Supplementary Figure 5. LFP lifetime of of in-situ generated cooper complex (^{3}MLCT) in the presence of iodocylohexane (quencher) (λ_{pump} = 355 nm, λ_{probe} = 440 nm). Bars in left figure represent mean ± SD (n = 20; individual data points are plotted).

4.4 Alkyl radical intermediate trapping experiments

TEMPO trapping experiments

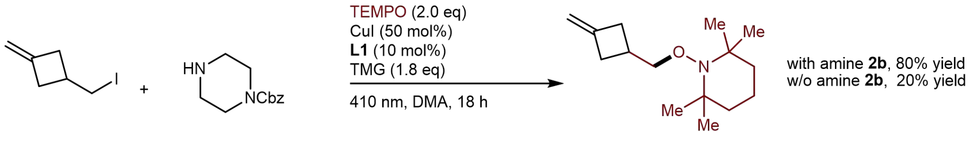

Experimental Procedure: To an oven-dried 10 mL vial were added CuI (9.5 mg, 0.05 mmol), **L1** (3.55 mg, 0.01 mmol), 1,1,3,3-Tetramethylguanidine (TMG) (23 μL, 0.18 mmol) and 1 mL DMA in a nitrogen-filled glove box. The resulting mixture was stirred for 10 min, followed by adding 1-(iodomethyl)-3-methylenecyclobutane (31.2 mg, 0.15 mmol), TEMPO (31.3 mg, 0.2 mmol) with/without benzyl piperazine-1-carboxylate (22.0 mg, 0.1 mmol) in sequence, and then sealed with a screwed cap. The sealed vial was placed on a photo-reactor under irradiation of LEDs (410 nm, 6 W). The mixture was stirred at 25 °C for 15 h, quenched with H_2O, and extracted with ethyl acetate. The combined organic layers were dried over anhydrous Na_2SO_4, concentrated in vacuo. The crude product was analyzed by ^{1}H NMR with 1,3,5-trimethoxybenzene as an internal standard.

Radical intermediates trapping experiments

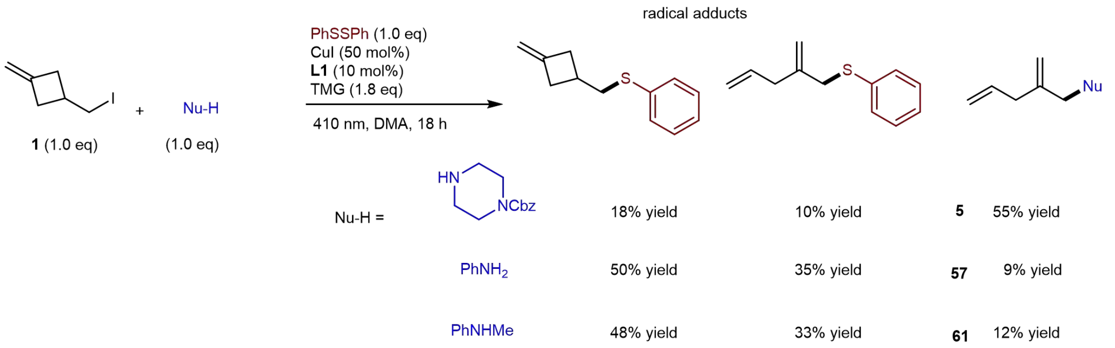

Experimental Procedure: To an oven-dried 10 mL reaction vial were added CuI (9.5 mg, 0.05 mmol), **L1** (3.6 mg, 0.01 mmol), 1,1,3,3-Tetramethylguanidine (TMG) (23 μL, 0.18 mmol) and 1 mL DMA in a nitrogen-filled glove box. The resulting mixture was stirred for 10 min, followed by adding 1-(iodomethyl)-3-methylenecyclobutane (31.2 mg, 0.15 mmol), diphenyl disulfide (21.8 mg, 0.1 mmol), *N*-nucleophiles (0.1 mmol) in sequence, and sealed with a screwed cap. The sealed vial was placed on a photo-reactor under irradiation of LEDs (410 nm, 6 W). The mixture was stirred at 25 °C for 15 h, quenched with H_2O, and extracted with ethyl acetate. The combined organic layers were dried over anhydrous Na_2SO_4, concentrated in vacuo. The crude product was analyzed by ^{1}H NMR with 1,3,5-trimethoxybenzene as an internal standard.

4.5 Kinetic studies

Hammett studies

To oven-dried 10 mL vial in a glove box were added CuI (3.8 mg, 0.02 mmol), **L1** (3.6 mg, 0.01 mmol), TMG (0.18 mmol) and DMA (1 mL). The reaction mixture was stirred for 10 min, followed by adding *p*-substituted *N*-methylaniline (0.1 mmol) and 1-(iodomethyl)-3-methylenecyclobutane (31.2 mg, 0.15 mmol). The sealed vial was placed on a photo-reactor under irradiation of 6W 410 nm LEDs and stirred at 25 °C, After completion of the reaction, The mixture was filtered through a celite pad and concentrated in vacuo. The reside was analyzed by ^{1}H NMR spectroscopy with 1,3,5-trimethoxybenzene as the internal standard.

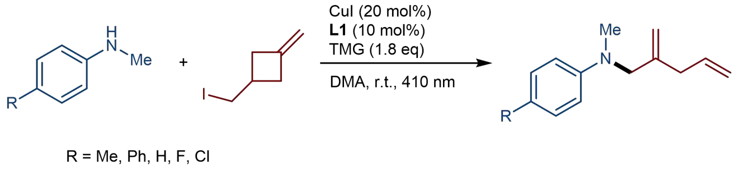

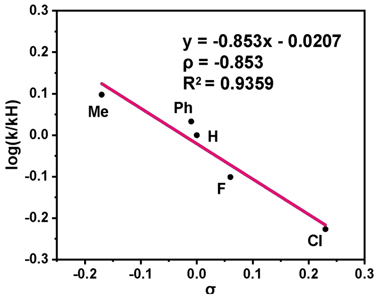

Supplementary Figure 6. Hammett plots of 1 with *p*-substituted *N*-methylaniline

Kinetic order of *N*-nucleophilic: To oven-dried 10 mL vial were added CuI (3.8 mg, 0.02 mmol), L1 (3.5 mg, 0.01 mmol), TMG (23 μL, 0.18 mmol) and DMA (1.0 mL) in a nitrogen-filled glove box. The resulting mixture was stirred for 10 min, followed by adding the appropriate amount of *N*-Methylaniline **98** or benzyl piperazine-1-carboxylate **2b** and 1-(iodomethyl)-3-methylenecyclobutane (0.1 mmol,) in sequence, and sealed with a screwed cap. The sealed vial was placed on a photo-reactor under irradiation of LEDs (410 nm, 6 W). The mixture was stirred at room temperature.The yield was analyzed by ^{1}H NMR along time. The experimental results were shown in the **Supplementary Figure 7** below.

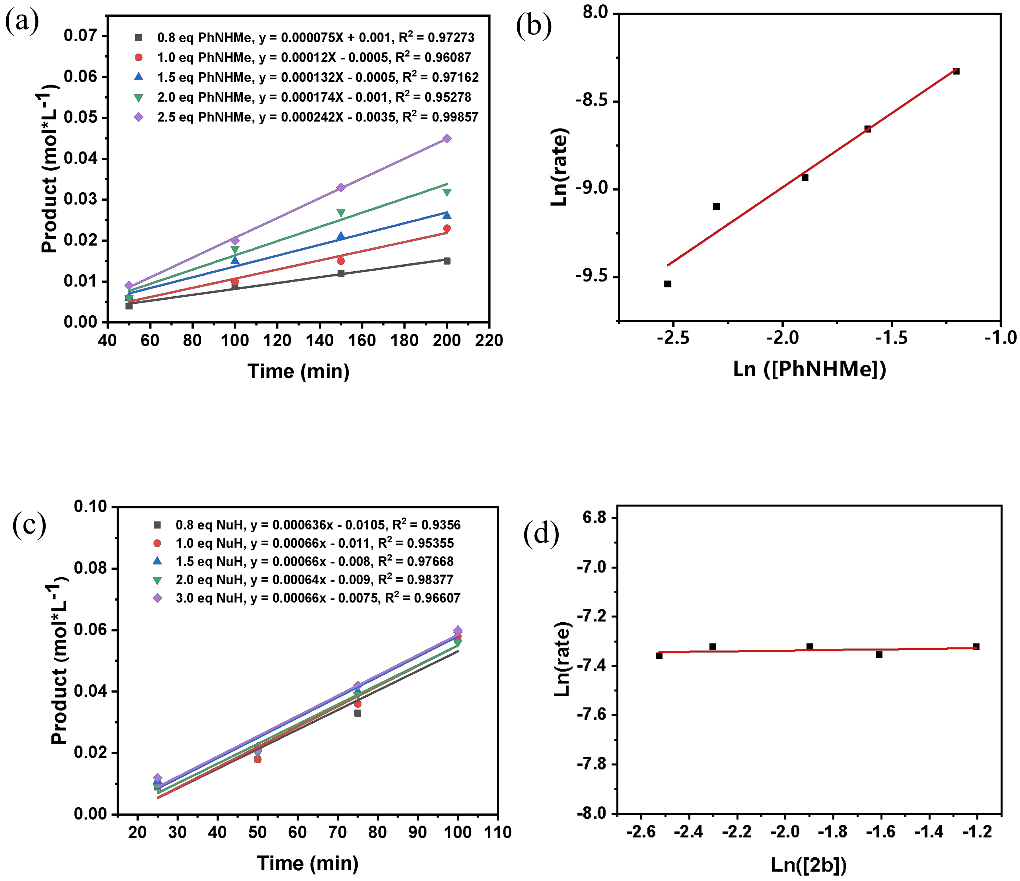

Supplementary Figure 7. a) The plot of concentration of **12** over time for reactions performed with varying concentration of PhNHMe. b) The plot of ln(rate) vs. ln(**PhNHMe**) for reaction. c) The plot of concentration of **12** over time for reactions performed with varying concentration of benzyl piperazine-1-carboxylate **2b**. d) The plot of ln(rate) vs. ln(**2b**)

Kinetic order of catalyst: To oven-dried 10 mL vial in a glove box were added was charged with the appropriate amount of insitu generated catalyst (CuI, **L1** and TMG in DMA). The reaction mixture was stirred for 10 minutes, followed by adding *N*-Methylaniline (0.10 mmol) or benzyl piperazine-1-carboxylate **2b** (0.10 mmol), 1-(iodomethyl)-3-methylenecyclobutane (0.10 mmol) in sequence, and sealed with a screwed cap. The sealed vial was placed on a photo-reactor under irradiation of LEDs

(410 nm, 6 W). The mixture was stirred at room temperature.The yield was analyzed by ^{1}H NMR along time. The experimental results were shown in the **Supplementary Figure 8** below.

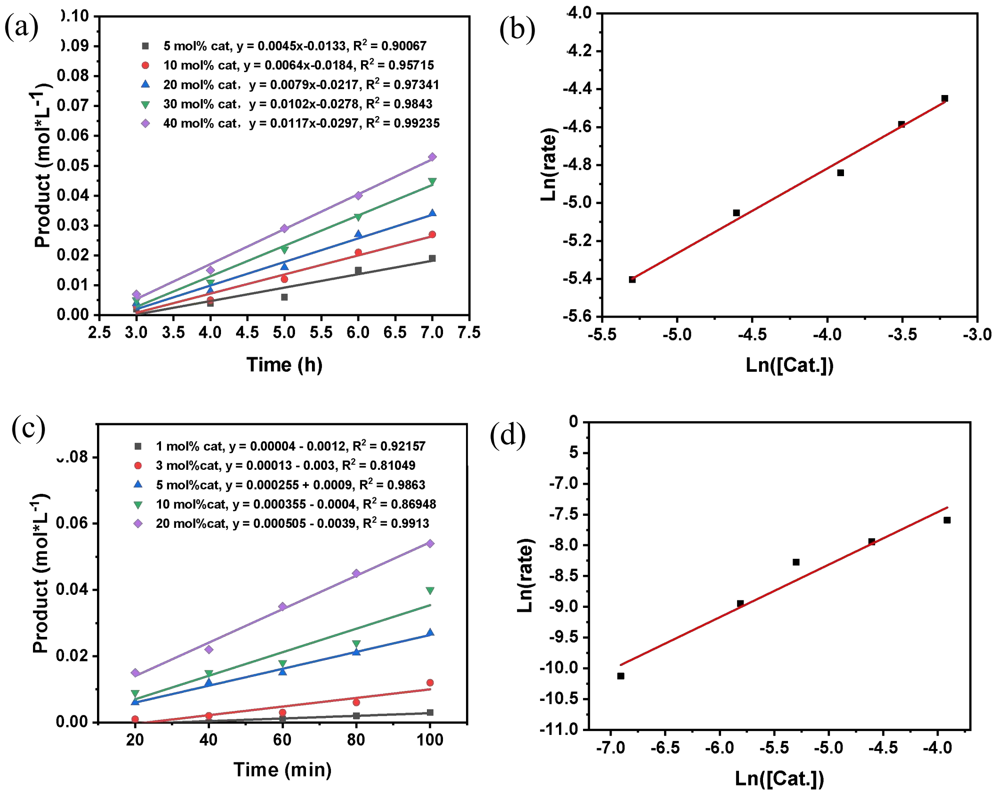

Supplementary Figure 8. PhNHMe as nucleophilies: a) The plot of concentration of **12** over time for reactions performed with varying concentration of catalyst. b) The plot of ln(rate) vs. ln(**cat.**) for reaction. benzyl piperazine-1-carboxylate **2b** as nucleophilies: c) The plot of concentration of **12** over time for reactions performed with varying concentration of catalyst. d) The plot of ln(rate) vs. ln(**cat.**) for reaction.

Kinetic order of alkyl iodides: To oven-dried 10 mL vial were added CuI (0.02 mmol), L1 (0.01 mmol), TMG (0.18 mmol) and DMA (1.0 mL) in a nitrogen-filled glove box. The resulting mixture was stirred for 10 min, followed by adding *N*-Methylaniline or benzyl piperazine-1-carboxylate **2b** and the appropriate amount of 1-(iodomethyl)-3-methylenecyclobutane in sequence, and sealed with a screwed cap. The sealed vial was placed on a photo-reactor under irradiation of LEDs (410 nm, 6 W). The mixture was stirred at room temperature. The yield was analyzed by ^{1}H NMR along time. The experimental results were shown in the **Supplementary Figure 9** below.

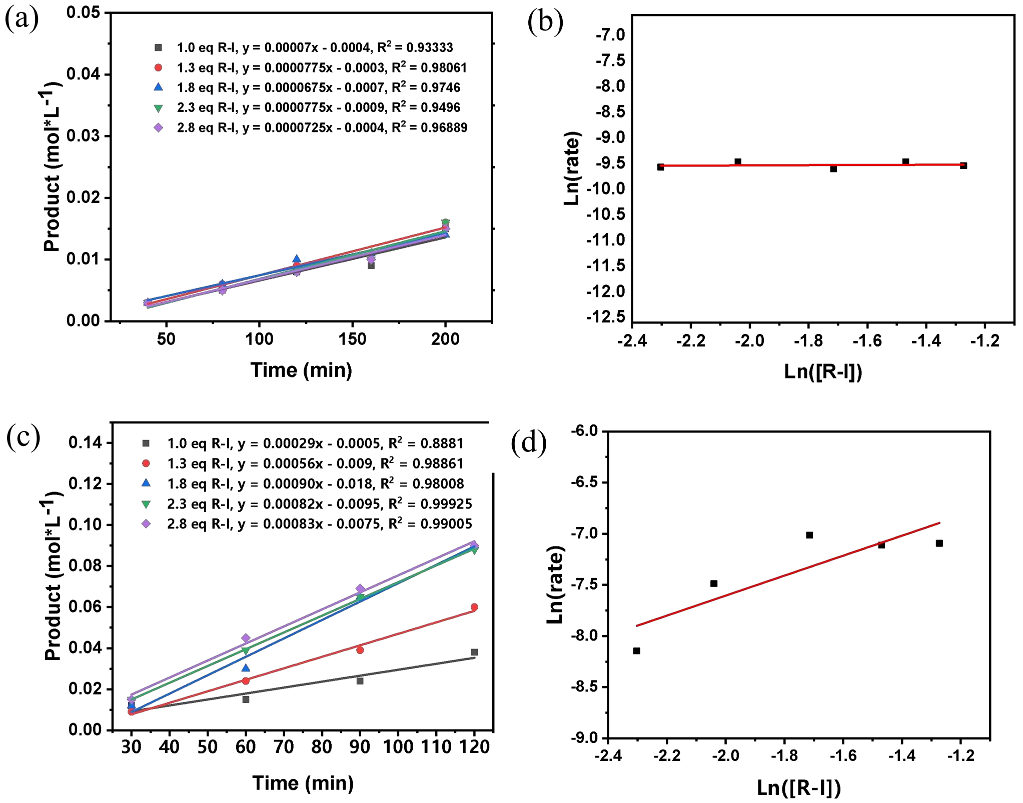

Supplementary Figure 9. PhNHMe as nucleophilies: a) The plot of concentration of **12** over time for reactions performed with varying concentration of alkyl iodides **1**. b) The plot of ln(rate) vs. ln(**1**) for reaction. benzyl piperazine-1-carboxylate **2b** as nucleophilies: c) The plot of concentration of **12** over time for reactions performed with varying concentration of alkyl iodides **1**. d) The plot of ln(rate) vs. ln(**1**) for reaction.

4.6 Electronchemical of in-situ generated copper complex

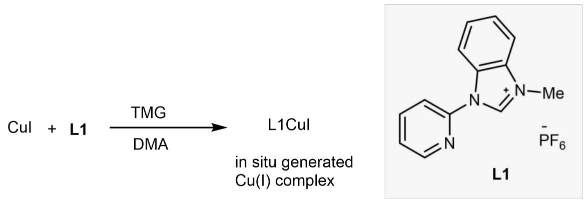

Experimental procedure: in situ gernerated copper(I) complex was prepared: a solution of CuI (1.0 equiv.), L1 (1.0 equiv.) and TMG (1.0 equiv.) in DMA. Cyclic voltammograms were recorded using a CH Instruments 656E potentiostat, a glassy carbon working electrode, a Ag/AgCl reference electrode, and a Pt counter electrode. The voltammograms were recorded at room temperature under nitrogen atmosphere in 0.1 M Bu_4NPF_6 in DMA containing in situ generated copper(I) complex (1 mM). Scan rate = 0.1 V/s, All potentials are reported in V vs Ag/AgCl.

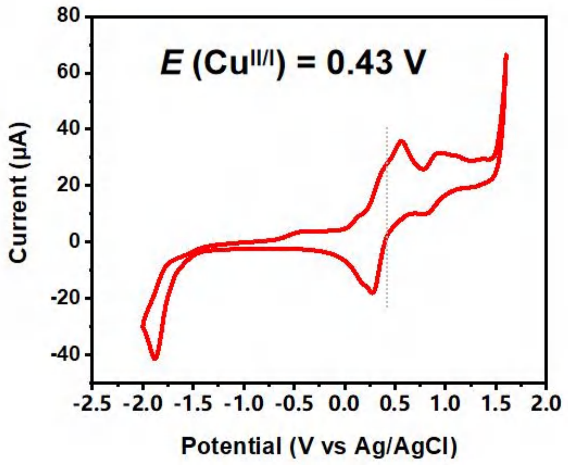

Supplementary Figure 10. Cyclic voltammogram of in-situ generated copper(I) complex

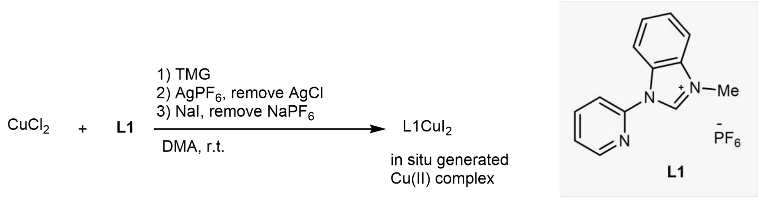

Experimental procedure: in situ gernerated copper(II) complex was prepared: a solution of $CuCl_2$ (1.0 equiv.), L1 (1.0 equiv.) and TMG (1.0 equiv.) in DMA, followed by the addition of $AgPF_6$ (2.0 equiv.) and NaI (2.0 equiv.) in sequence. Cyclic voltammograms were recorded using a CH Instruments 656E potentiostat, a glassy carbon working electrode, a Ag/AgCl reference electrode, and a Pt counter electrode. The voltammograms were recorded at room temperature under nitrogen atmosphere in 0.1 M Bu_4NPF_6 in DMA containing in situ generated copper(II) complex (1 mM). Scan rate = 0.1 V/s, All potentials are reported in V vs Ag/AgCl.

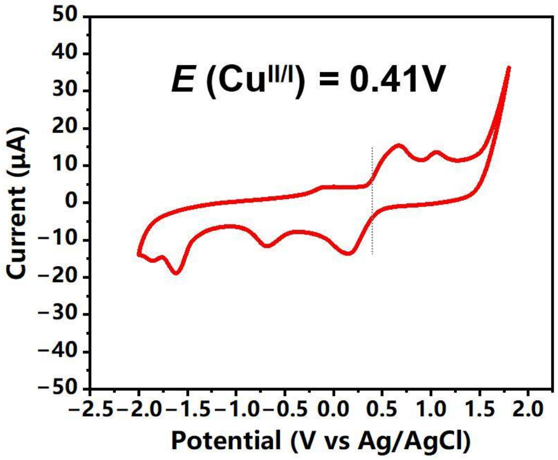

Supplementary Figure 11. Cyclic voltammogram of in-situ generated copper(II)

4.7 Light on/off experiments

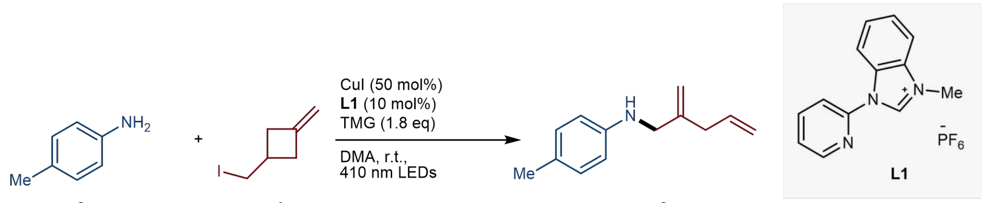

Experimental procedure: To oven-dried 10 mL vial were added CuI (0.05 mmol), L1 (0.01 mmol, 1.0 equiv.), TMG (0.18 mmol, 1.8 equiv.) and DMA (1.0 mL) in a nitrogen-filled glove box. The resulting mixture was stirred for 10 min, followed by adding *p*-toluidine (0.1 mmol, 1.0 equiv.), 1-(iodomethyl)-3-methylenecyclobutane (0.15 mmol, 1.5 equiv). The resulting mixture was subjected to alternating intervals of irradiation with 410 nm light and dark. The reaction profile was shown in **Supplementary Figure 12** and the yield of product **3a** as a function of time was determined by ^{1}H-NMR using 1,3,5-trimethoxybenzene as an internal standard. These results indicated that continuous irradiation with light was essential for the progress of this reaction.

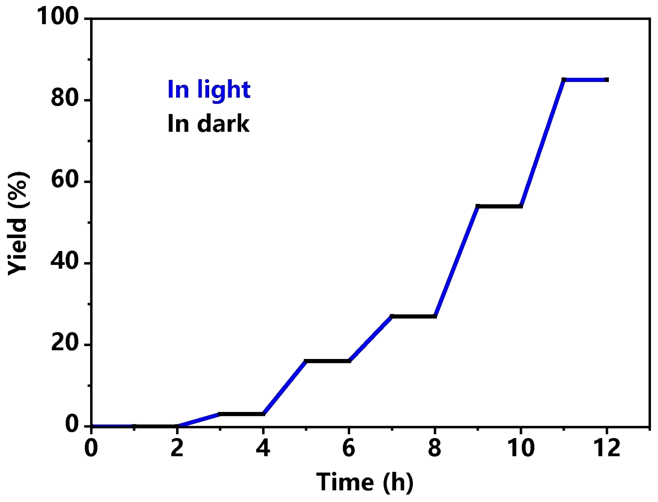

Supplementary Figure 12. Light on/off experiments

5. Computational Details

All computations in the present study were performed by Density Functional Theory implemented in Gaussian16c01 program.[5] The optimizer implemented in develop version of global reaction route mapping (GRRM) program[6] were used for all the optimizations of intermediates, transition state (TS) and the solvent effects of by the PCM model in *N*,*N*-dimethylacetamide was used for geometry optimization of all chemical structures. B3LYP functional with the D3 empirical dispersion correction (B3LYP-D3)[7] was selected as the exchange-correlation functional and Def2TZVP basis set was used for all the atoms. The transition states were verified by frequency calculations and IRC calculations. Gibbs free energy was calculated at 298.15 K. The

redox potential was calculated by the following equation:

$$E_{0/1} = -\left(\frac{E_{(reduced)} - E_{(oxidized)}}{n_e F}\right) - E_{ABS}(REF)$$

Where $E_{(reduced)}$ is the energy of neutral system, $E_{(oxidized)}$ is energy of oxidized system, n_e is the number of electrons transferred, F is Faraday constant (96485.33289 C/mol), $E_{ABS}(REF)$ is the reference potential of Ag/AgCl, 4.639 (4.44V + 0.199V) at 25 °C.

$E(Cu^{II})$ = -2902.85829945183 Hatree = -7621454465 J/mol
$E(Cu^{I})$ = -2903.01123718847 Hatree = -7621856003 J/mol
Therefore, $E_{cal}(Cu^{II/I})$ = 0.48 vs Ag/AgCl

E_{rad} = -234.0727463 Hatree = -614557995.4 J/mol
$E_{cat.}$= -233.8676269 Hatree = -614019454.4 J/mol
Therefore, $E(_{rad}/_{cat.})$ = 0.94 vs Ag/AgCl

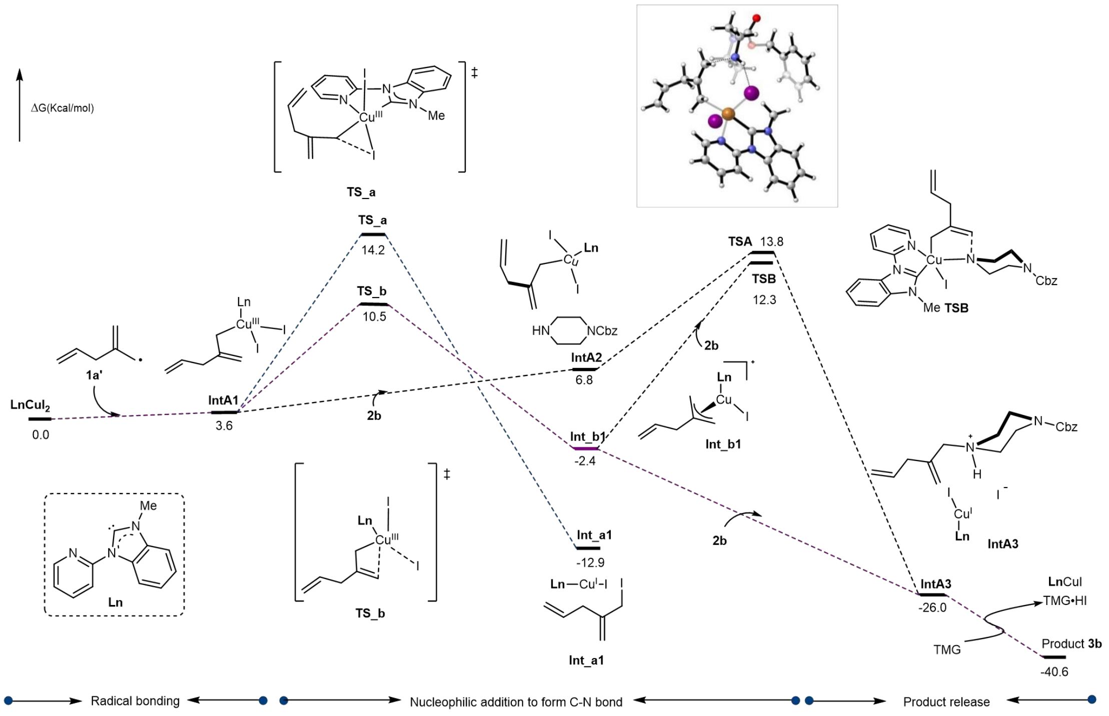

Supplementary Figure 13. DFT calculation for reaction pathways. Gibbs free energy was calculated by DFT at the level of B3LYP-D3/Def2TZVP at 298.15 K. The solvent effects of *N*,*N*-dimethylacetamide by the PCM model was considered. The energies are given in kcal/mol.

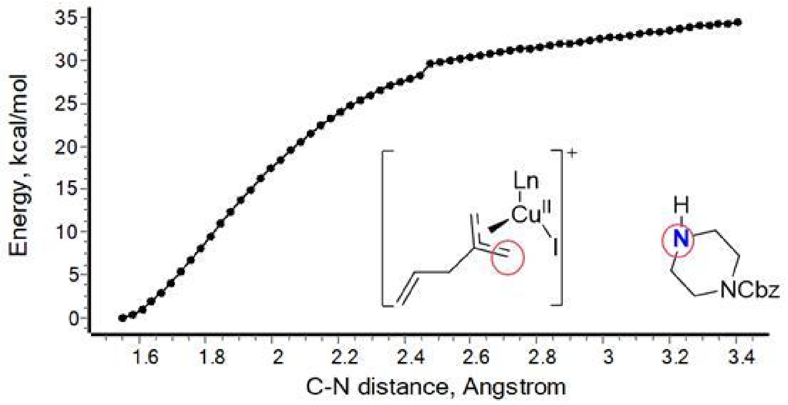

Supplementary Figure 14 Energy profile surface scan with respect to the distance between the C atom of allylic copper(III) and the N atom of the amine. As the computational cost for Def2TZVP basis set is too heavy for scan calculation, The scan calculation was done at the level of 6-31G** C H O N atoms, SDD with *d*-polarization basis set (*Chem. Phys. Lett.* **208**, 237-240 (1993)) for I atoms, SDD with f-polarization (*Chem. Phys. Lett.* **208**, 111-114 (1993)) for Cu atoms. The solvent effect of *N*,*N*-dimethylacetamide by the PCM model was included.

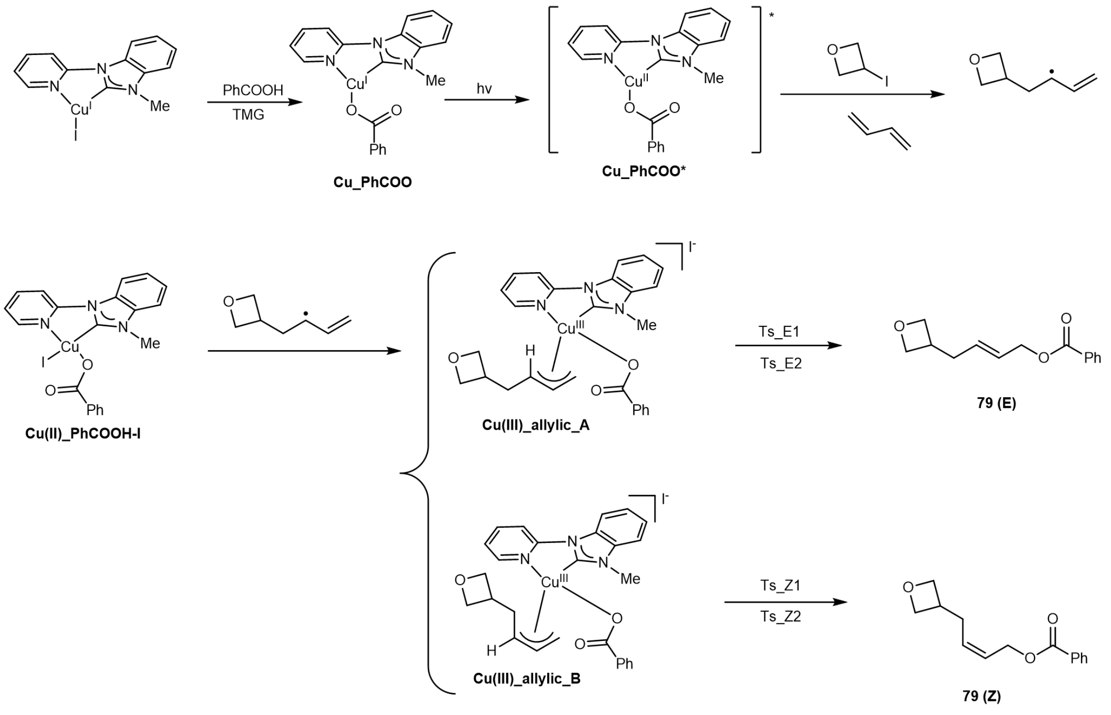

Supplementary Figure 15. Proposed mechanism for the three component reaction with carboxylic acid as the nucleophiles to yield *E*/*Z* isomers

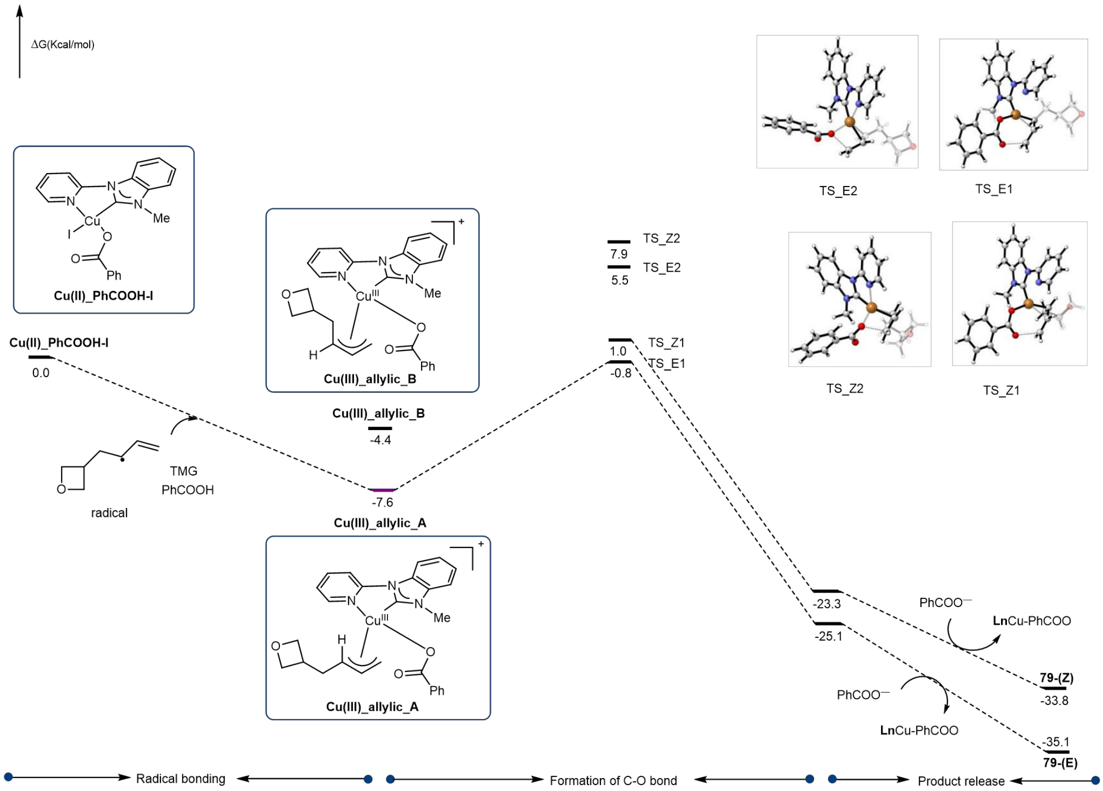

Supplementary Figure 16. Gibbs free energy potential to access *E*/*Z* isomers at the level of B3LYP-D3/Def2-TZVP at 298.15 K. The solvent effects of *N*,*N*-dimethylacetamide by the PCM model was considered. The energies are given in kcal/mol.

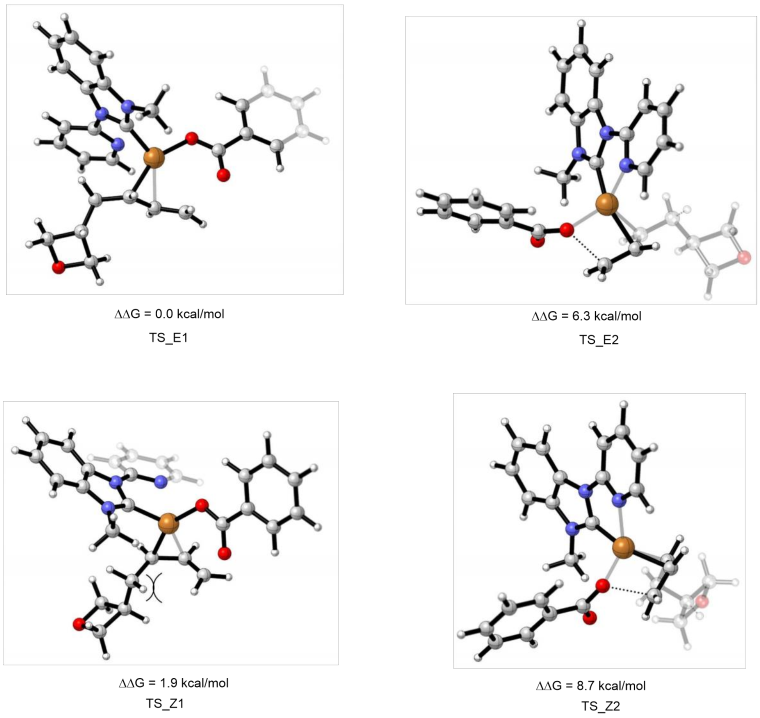

Supplementary Figure 17. The geometry of the different transition state.

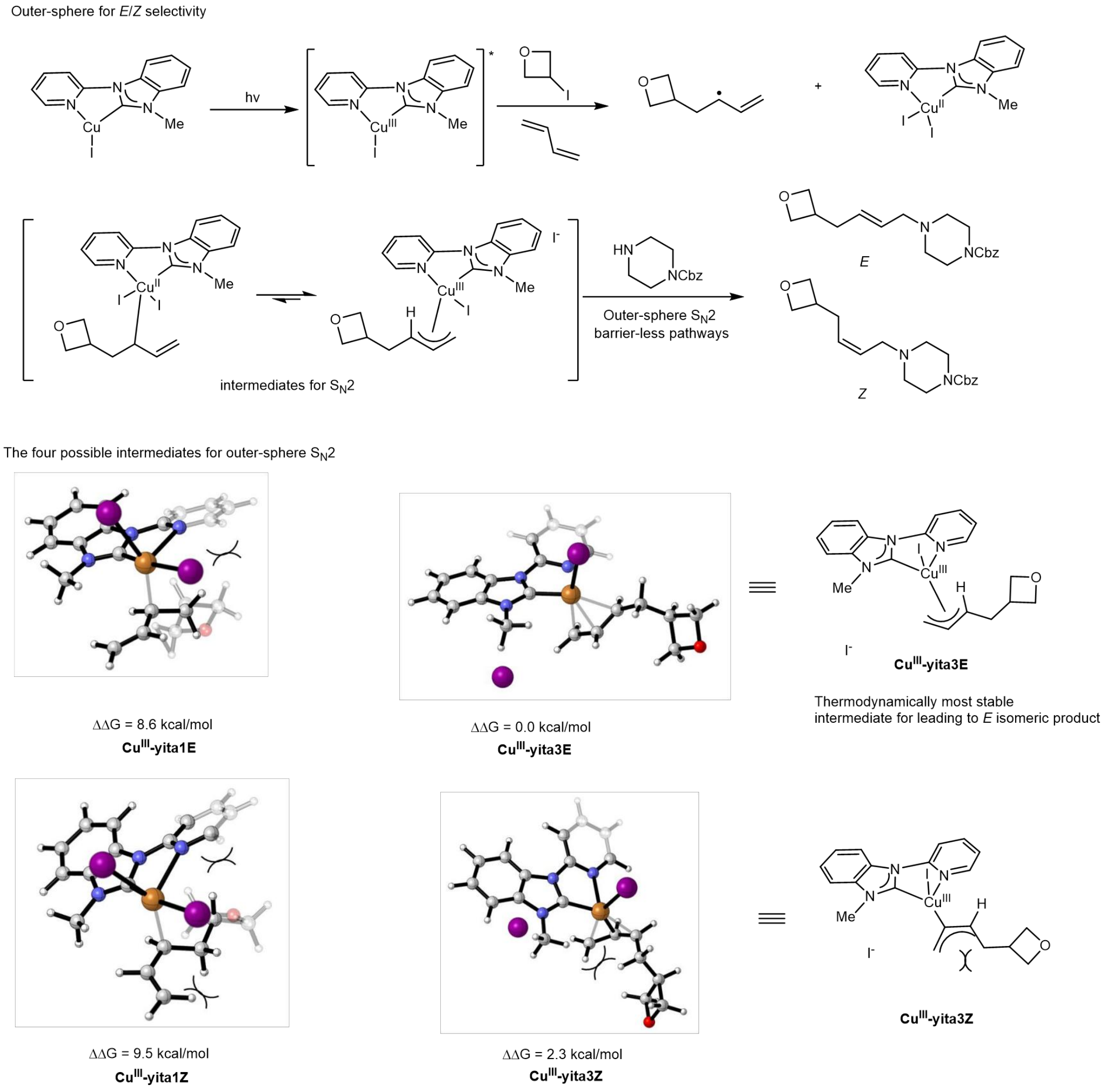

Supplementary Figure 18. The geometry of the allylic Cu^{III} intermediate for outer-sphere S$_N$2 reaction.

6. Copies of NMR Spectra

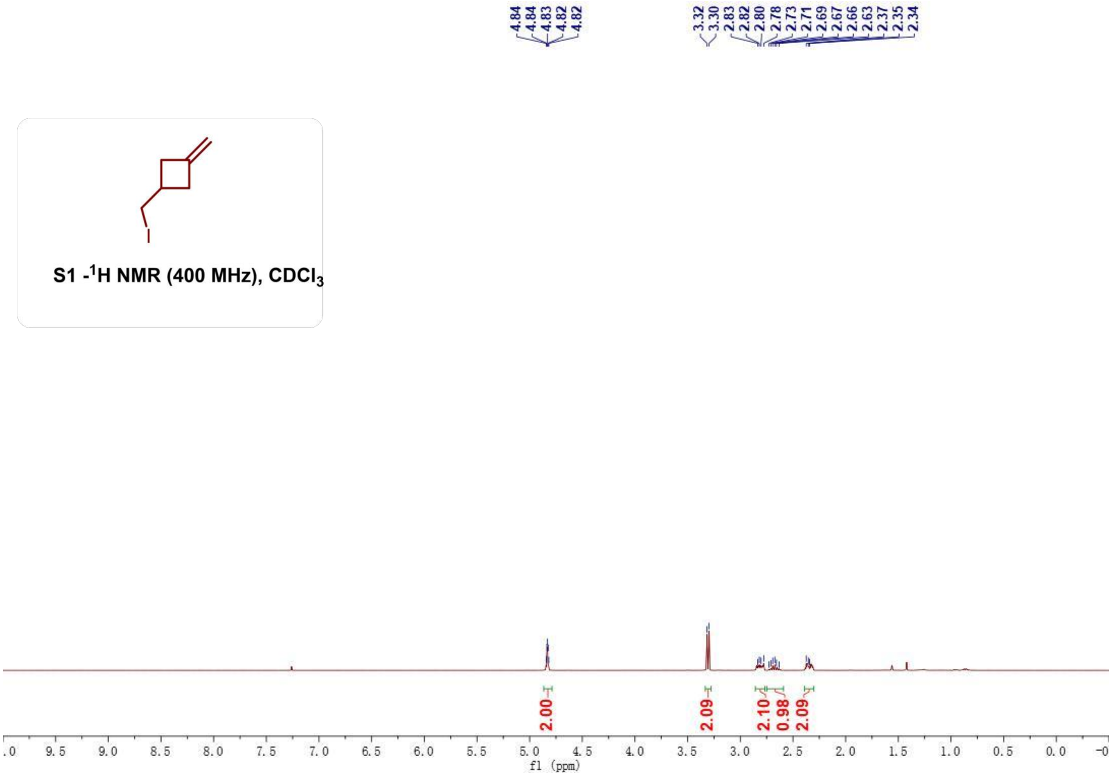

Supplementary Figure 19. ^{1}H NMR (400 MHz, $CDCl_3$) spectrum of compound **S1**

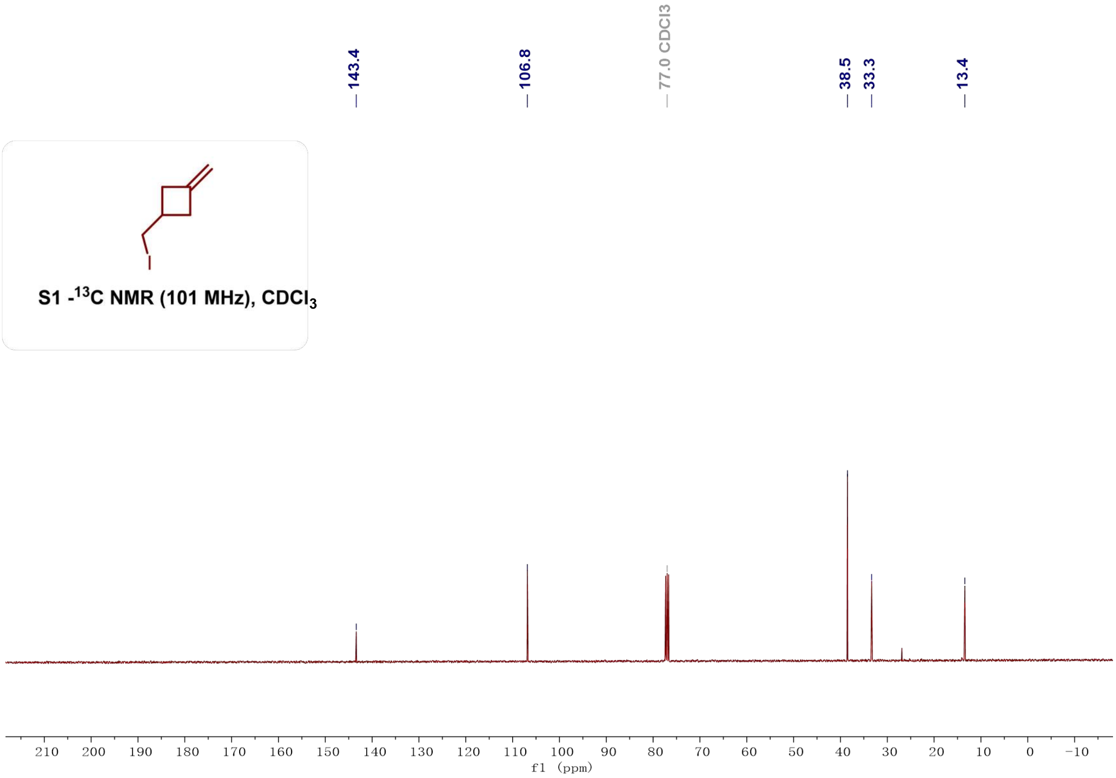

Supplementary Figure 20. ^{13}C NMR (101 MHz, $CDCl_3$) spectrum of compound **S1**

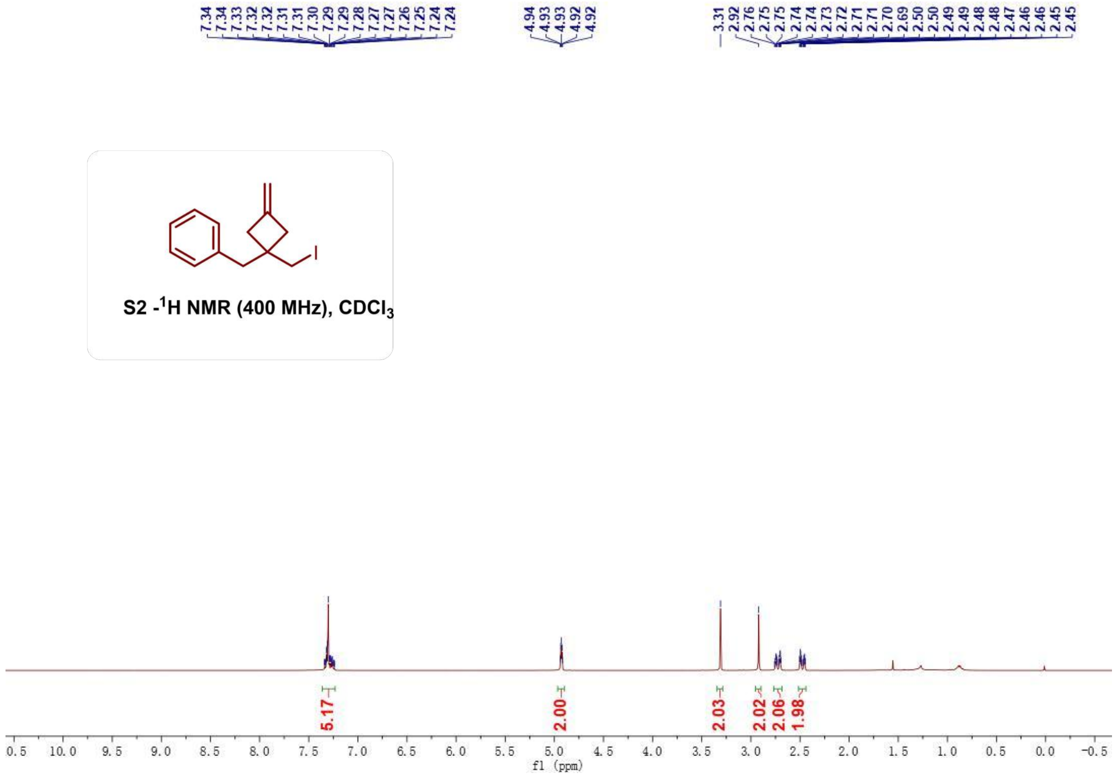

Supplementary Figure 21. ^{1}H NMR (400 MHz, $CDCl_3$) spectrum of compound **S2**

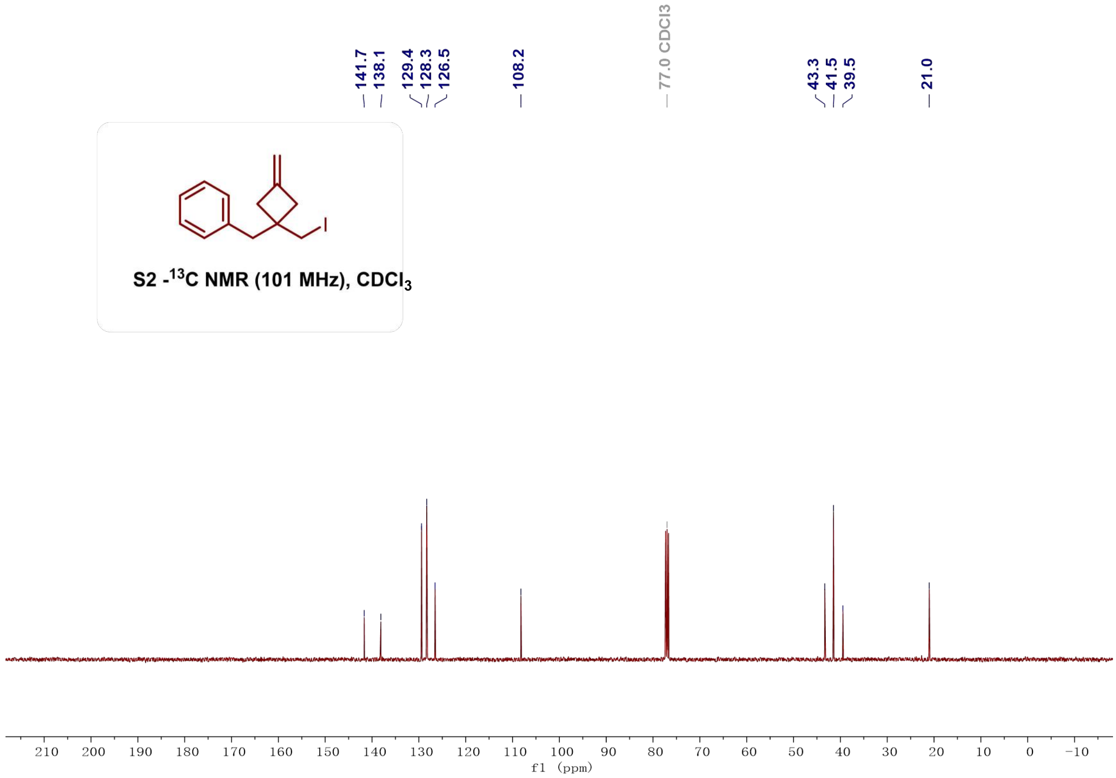

Supplementary Figure 22. ^{13}C NMR (101 MHz, $CDCl_3$) spectrum of compound **S2**

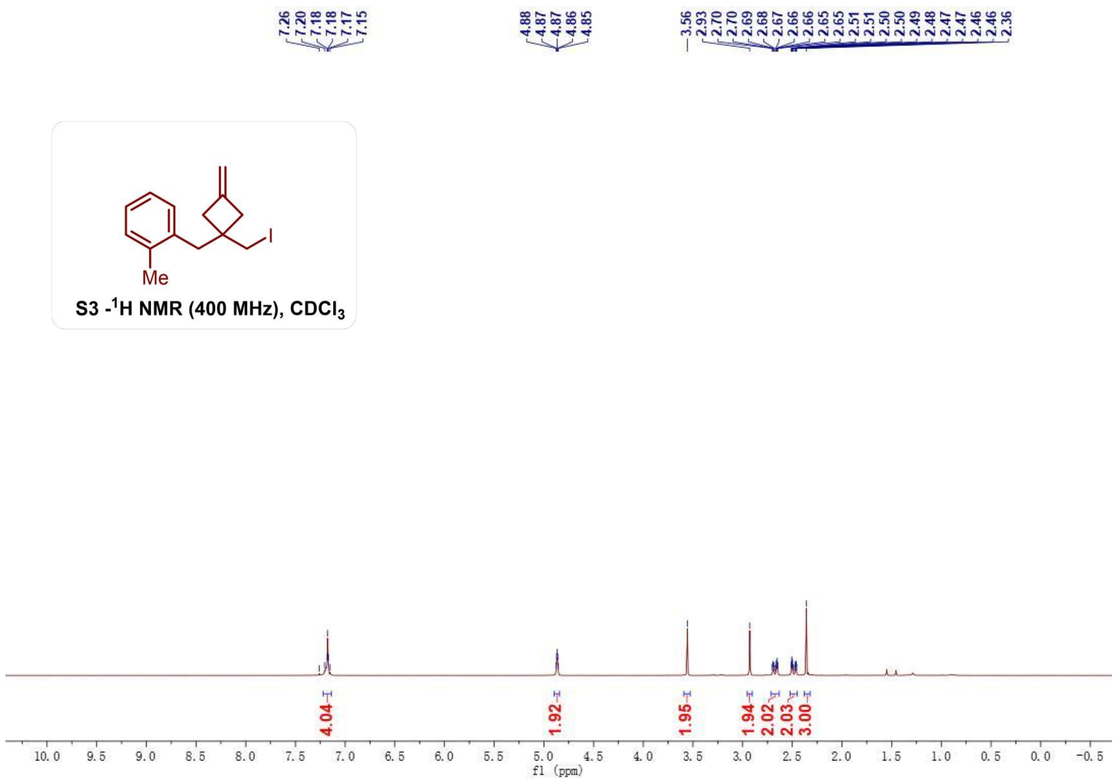

Supplementary Figure 23. ^{1}H NMR (400 MHz, $CDCl_3$) spectrum of compound **S3**

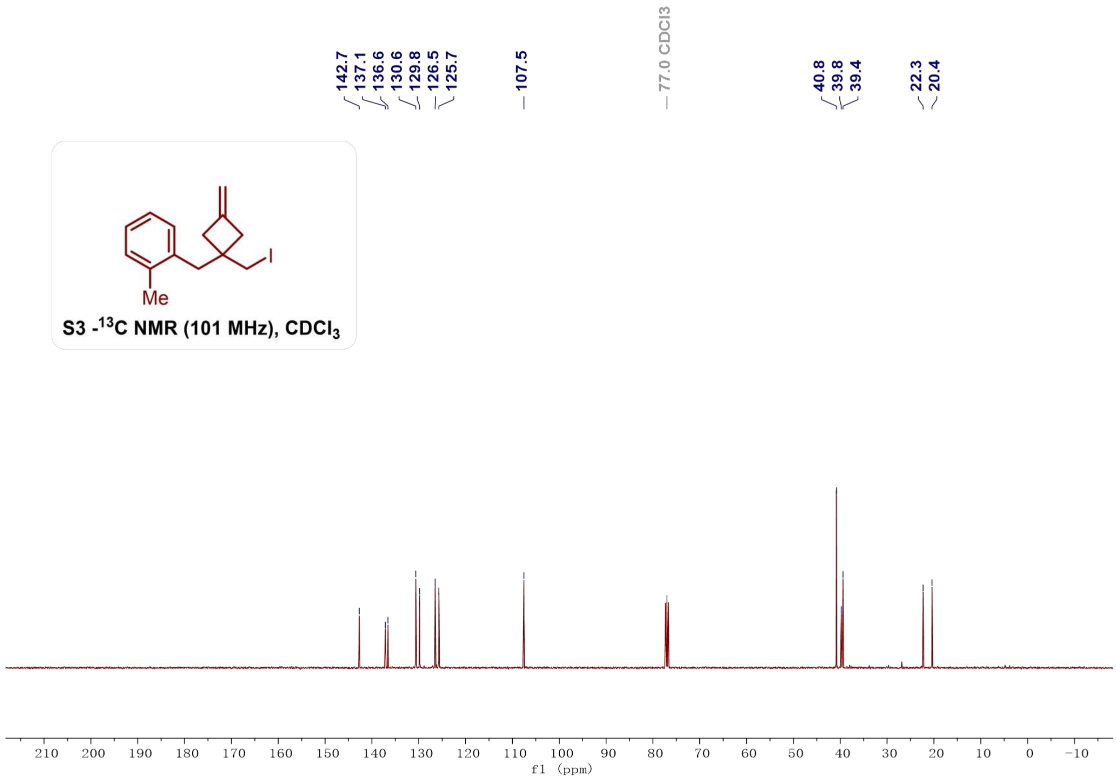

Supplementary Figure 24. ^{13}C NMR (101 MHz, $CDCl_3$) spectrum of compound **S3**

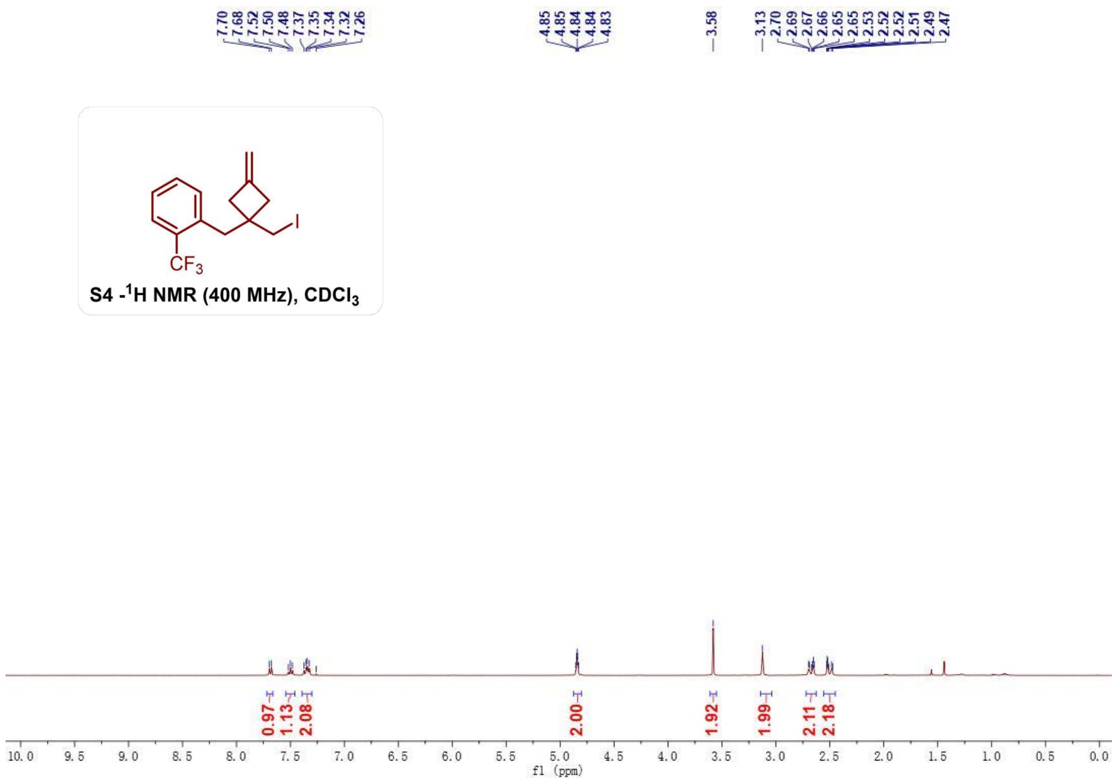

Supplementary Figure 25. ^{1}H NMR (400 MHz, $CDCl_3$) spectrum of compound **S4**

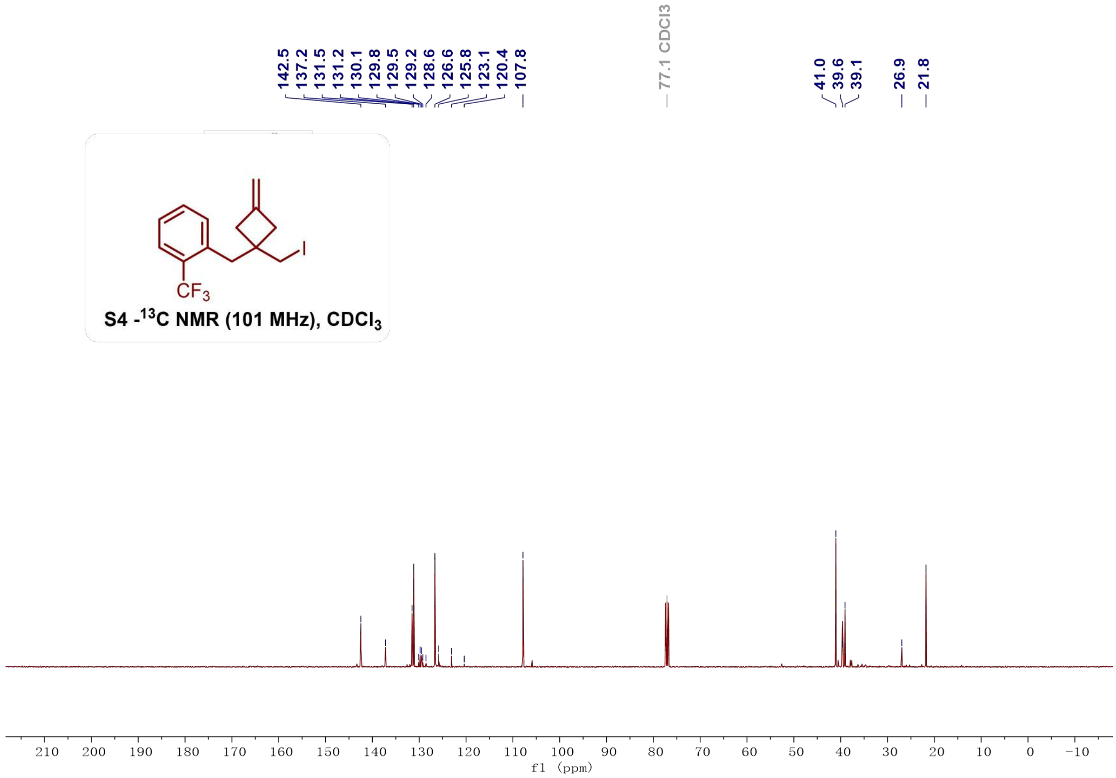

Supplementary Figure 26. ^{13}C NMR (101 MHz, $CDCl_3$) spectrum of compound **S4**

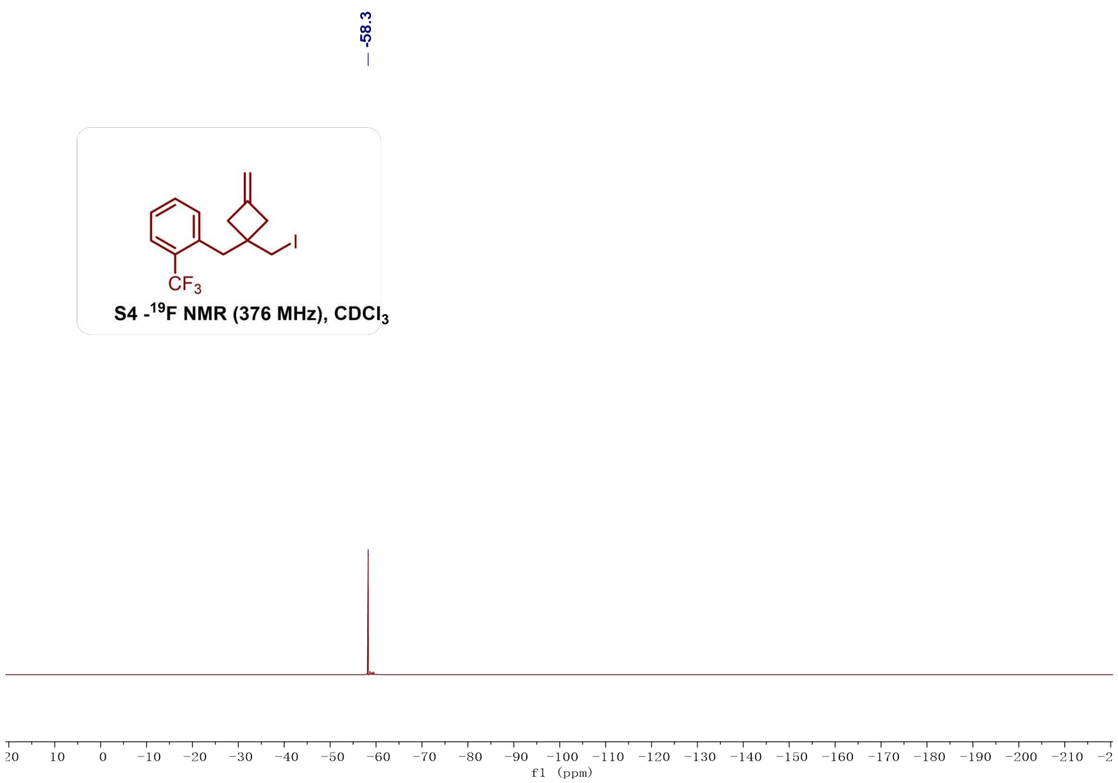

Supplementary Figure 27. ^{19}F NMR (376 MHz, $CDCl_3$) spectrum of compound **S4**

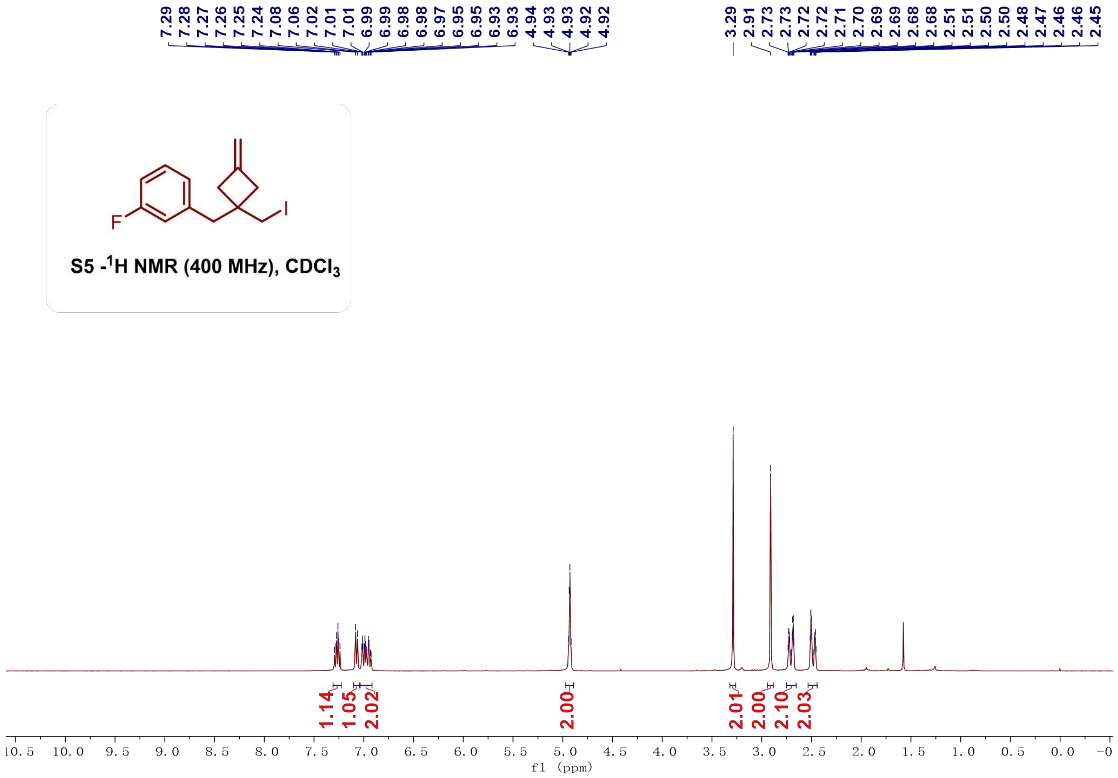

Supplementary Figure 28. ^{1}H NMR (400 MHz, $CDCl_3$) spectrum of compound **S5**

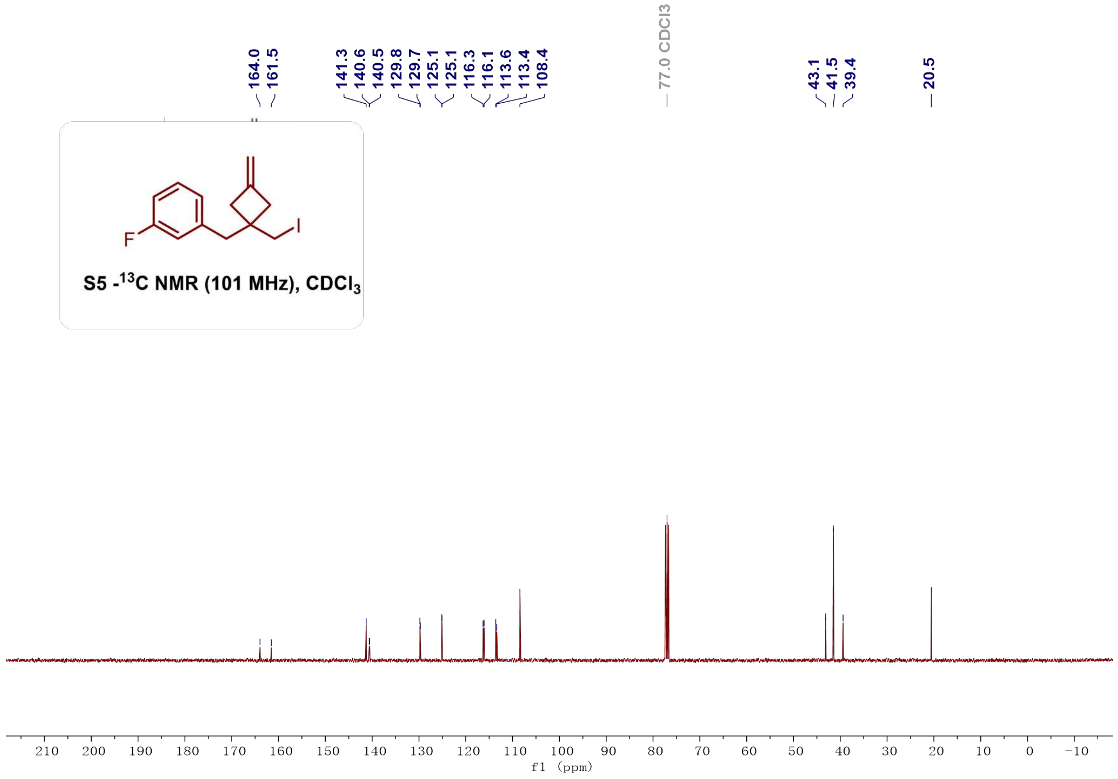

Supplementary Figure 29. ^{13}C NMR (101 MHz, $CDCl_3$) spectrum of compound **S5**

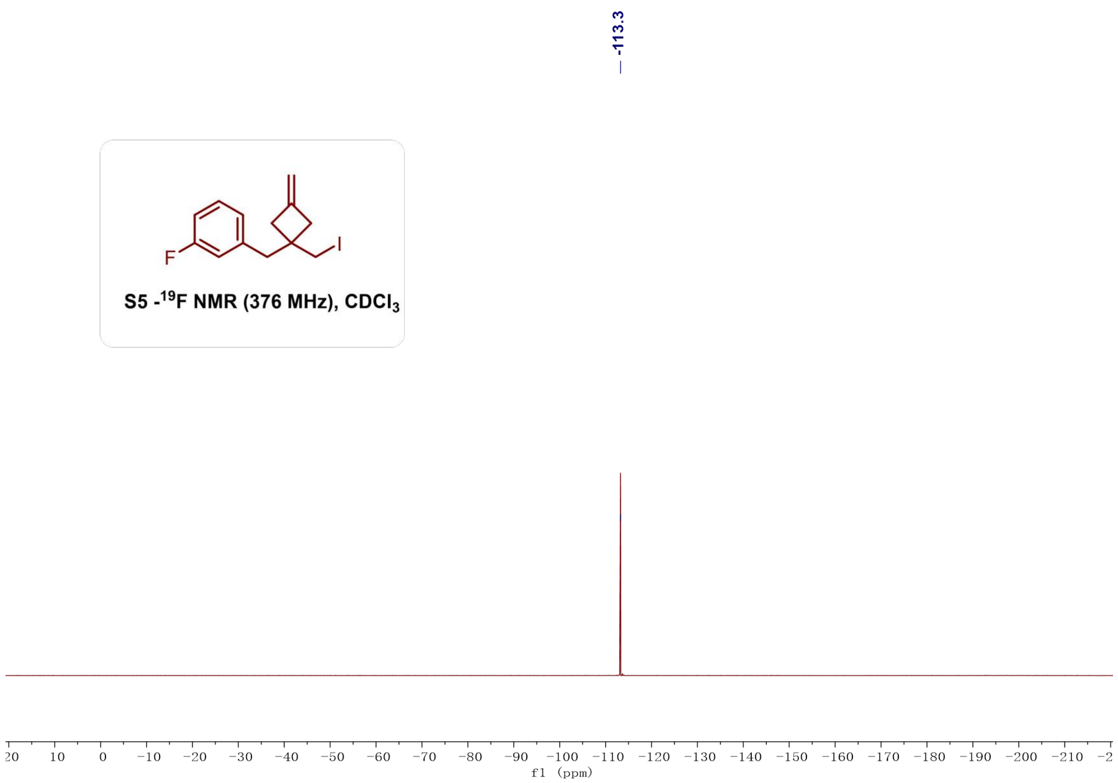

Supplementary Figure 30. ^{19}F NMR (376 MHz, $CDCl_3$) spectrum of compound **S5**

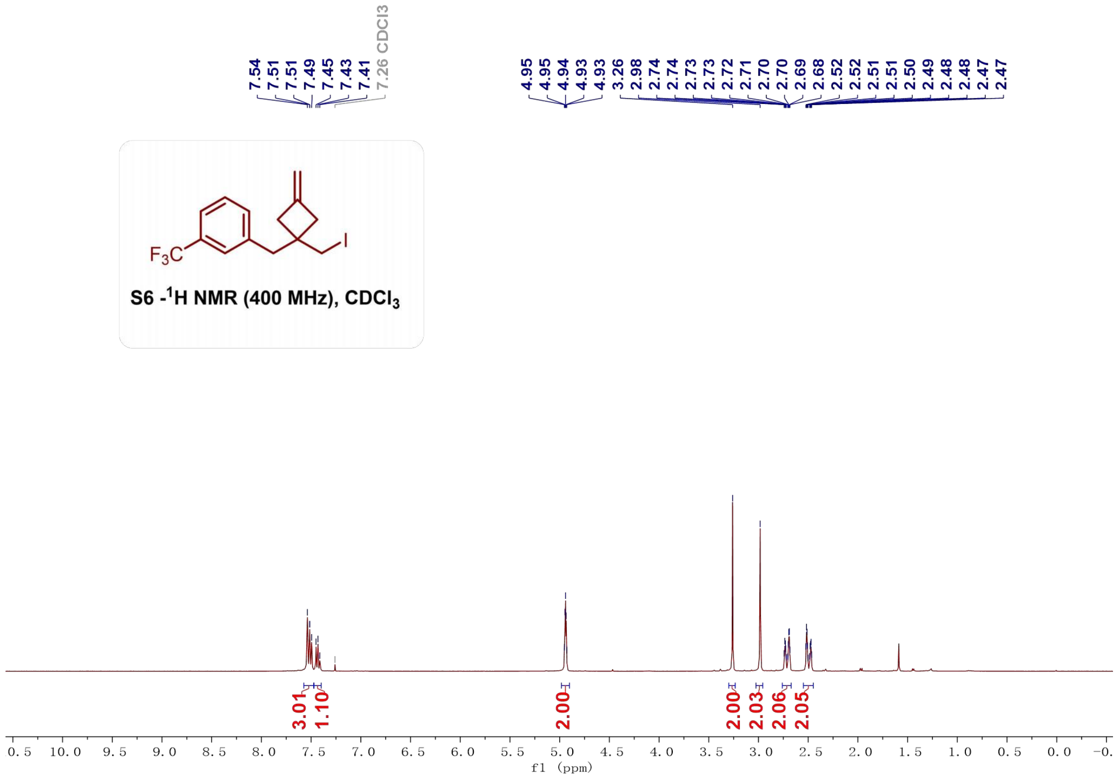

Supplementary Figure 31. ^{1}H NMR (400 MHz, $CDCl_3$) spectrum of compound **S6**

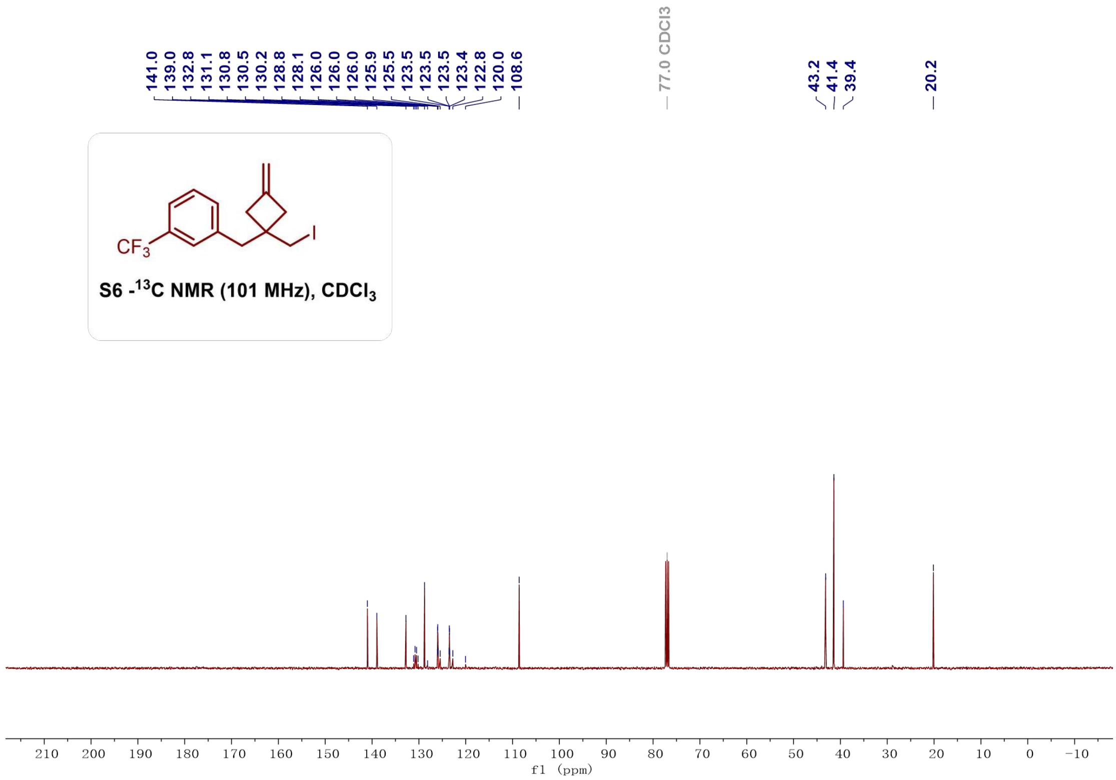

Supplementary Figure 32. ^{13}C NMR (101 MHz, $CDCl_3$) spectrum of compound **S6**

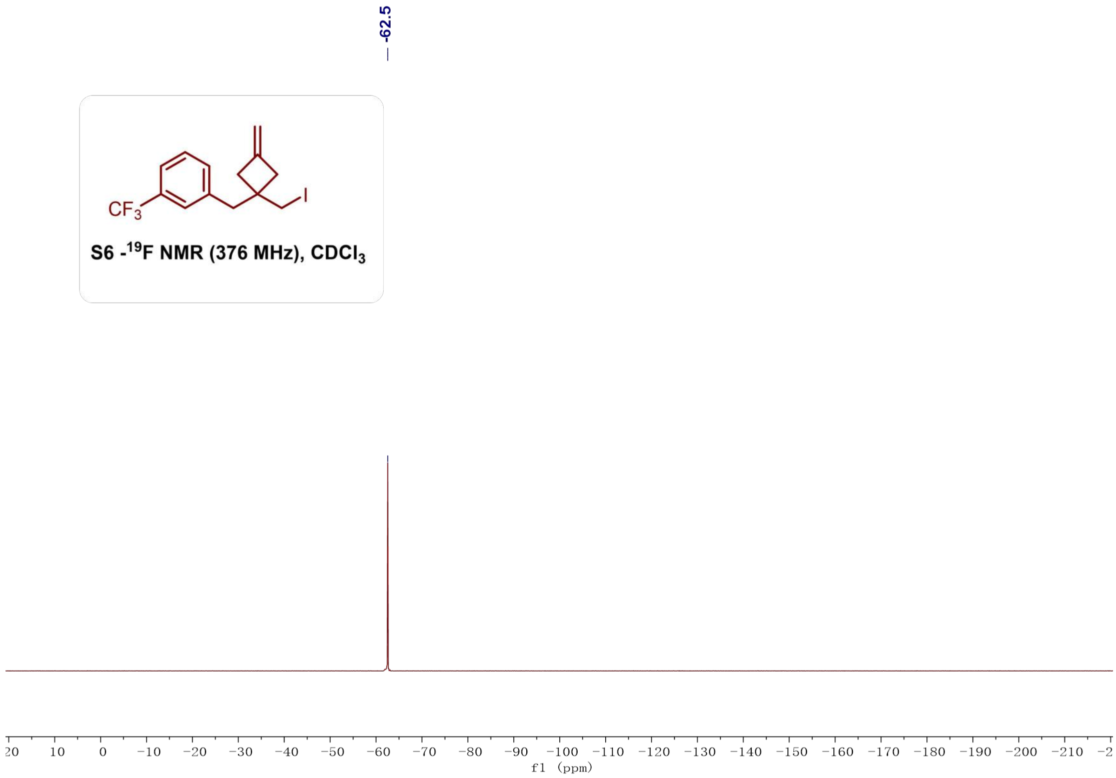

Supplementary Figure 33. ^{19}F NMR (376 MHz, $CDCl_3$) spectrum of compound **S6**

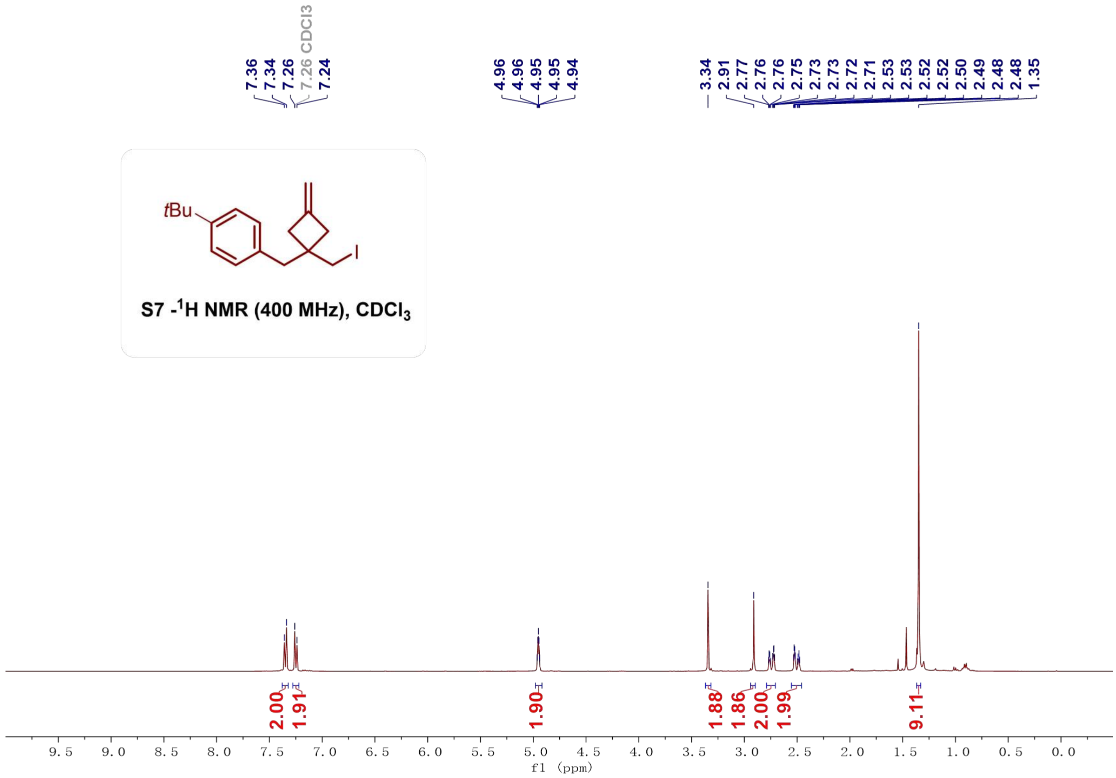

Supplementary Figure 34. ^{1}H NMR (400 MHz, $CDCl_3$) spectrum of compound **S7**

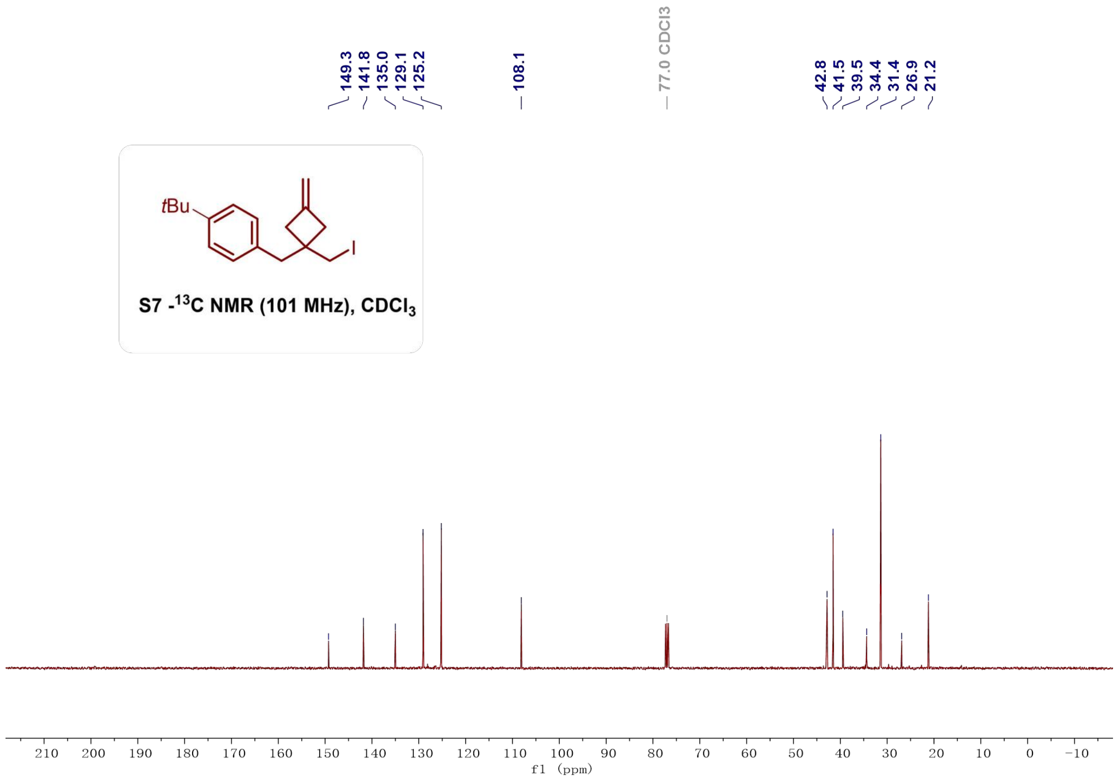

Supplementary Figure 35. ^{13}C NMR (101 MHz, $CDCl_3$) spectrum of compound **S7**

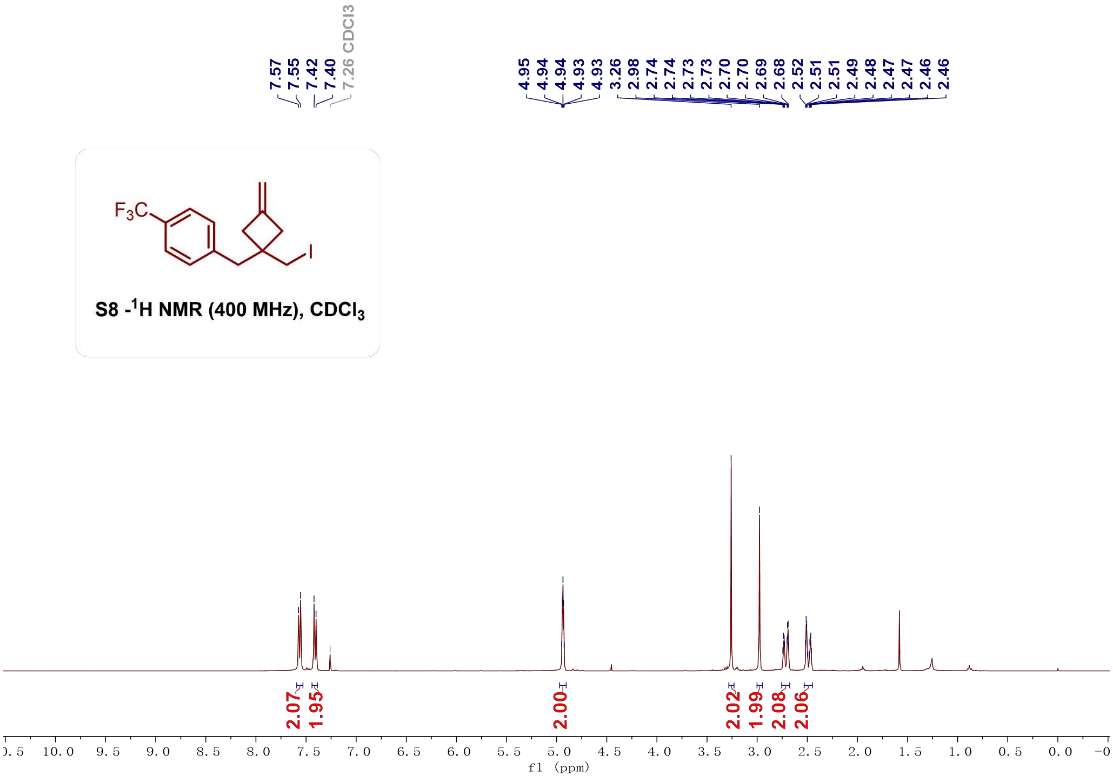

Supplementary Figure 36. ^{1}H NMR (400 MHz, $CDCl_3$) spectrum of compound **S8**

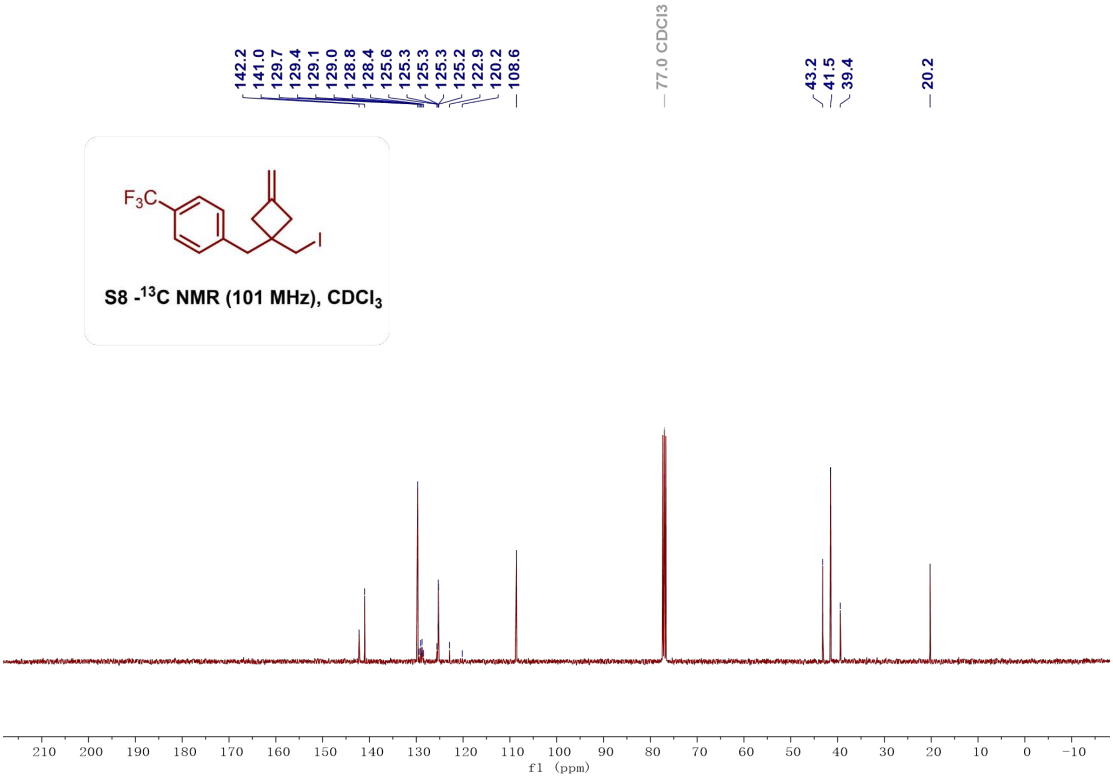

Supplementary Figure 37. ^{13}C NMR (101 MHz, $CDCl_3$) spectrum of compound **S8**

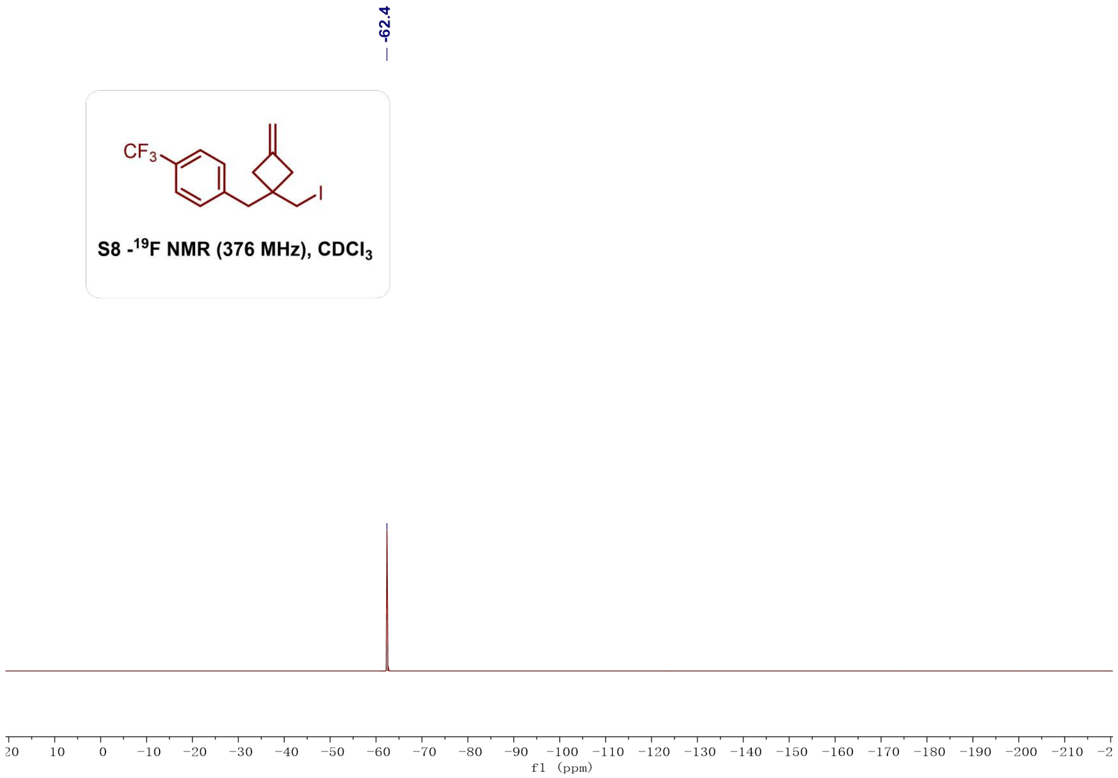

Supplementary Figure 38. ^{19}F NMR (376 MHz, $CDCl_3$) spectrum of compound **S8**

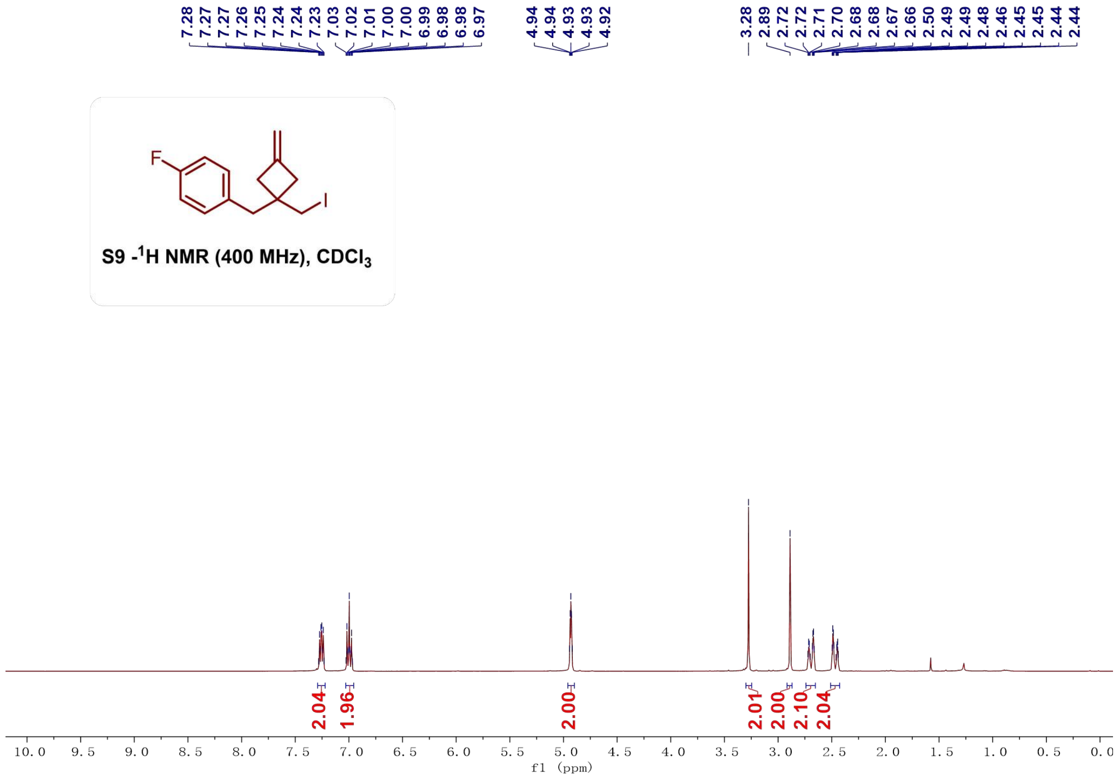

Supplementary Figure 39. ^{1}H NMR (400 MHz, $CDCl_3$) spectrum of compound **S9**

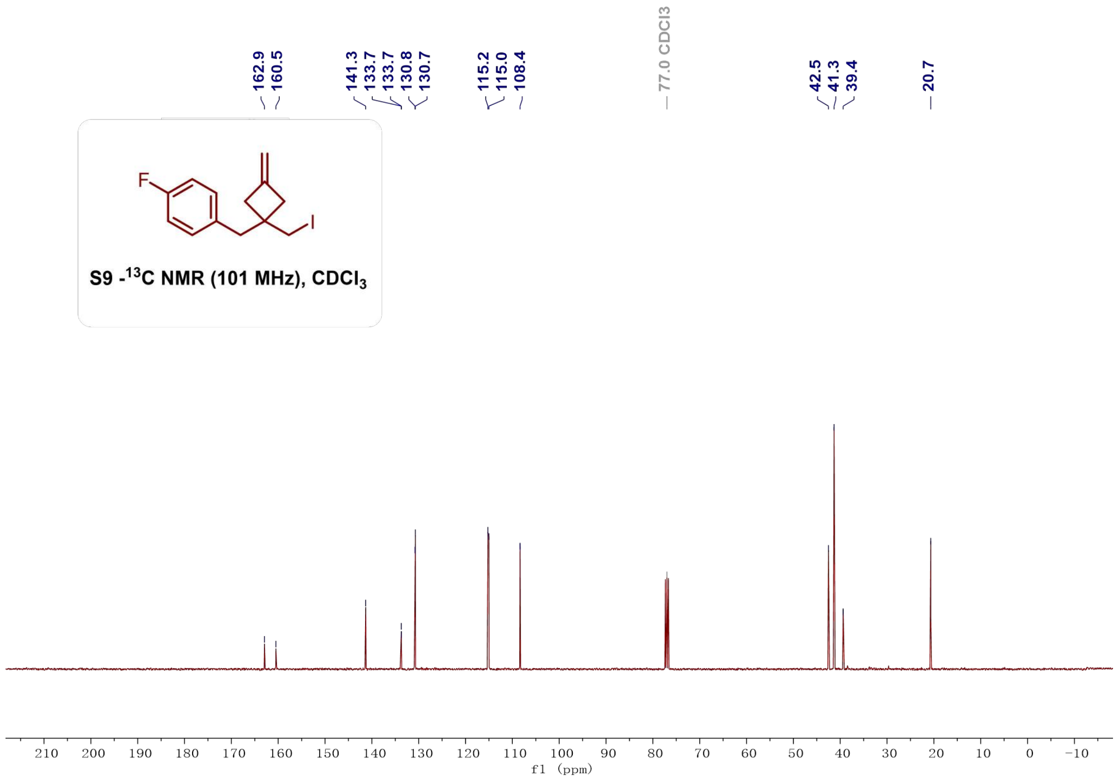

Supplementary Figure 40. ^{13}C NMR (101 MHz, $CDCl_3$) spectrum of compound **S9**

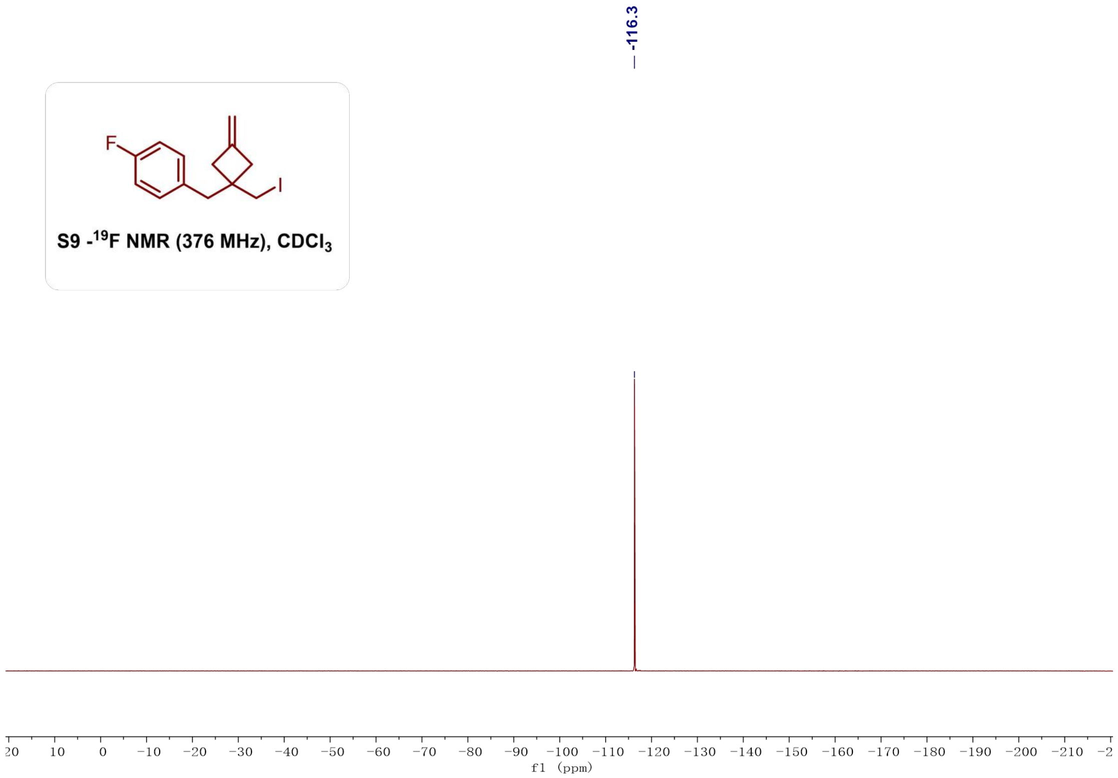

Supplementary Figure 41. ^{19}F NMR (376 MHz, $CDCl_3$) spectrum of compound **S9**

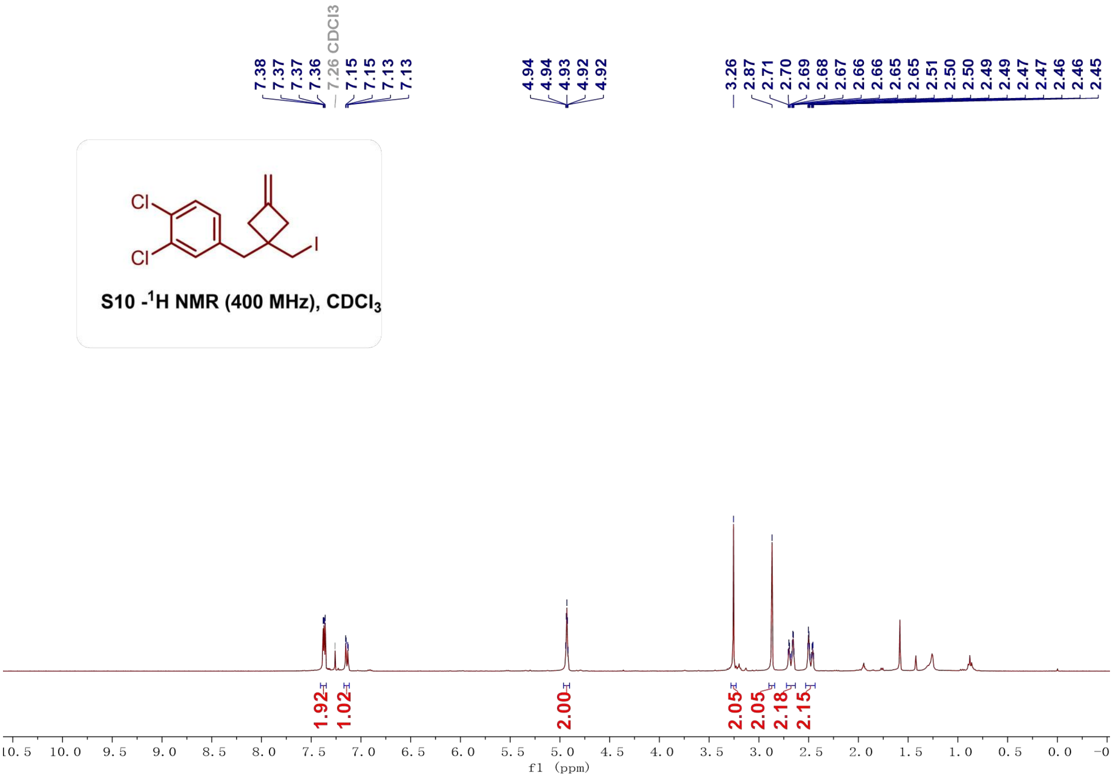

Supplementary Figure 42. ^{1}H NMR (400 MHz, $CDCl_3$) spectrum of compound **S10**

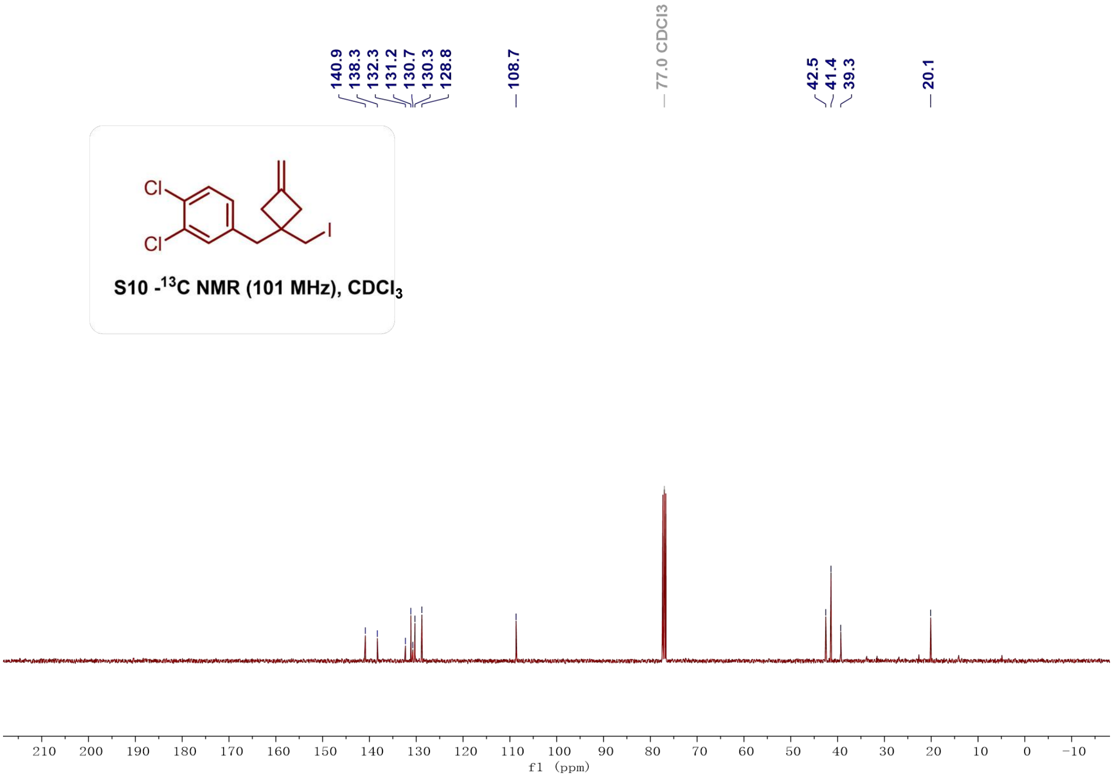

Supplementary Figure 43. ^{13}C NMR (101 MHz, $CDCl_3$) spectrum of compound **S10**

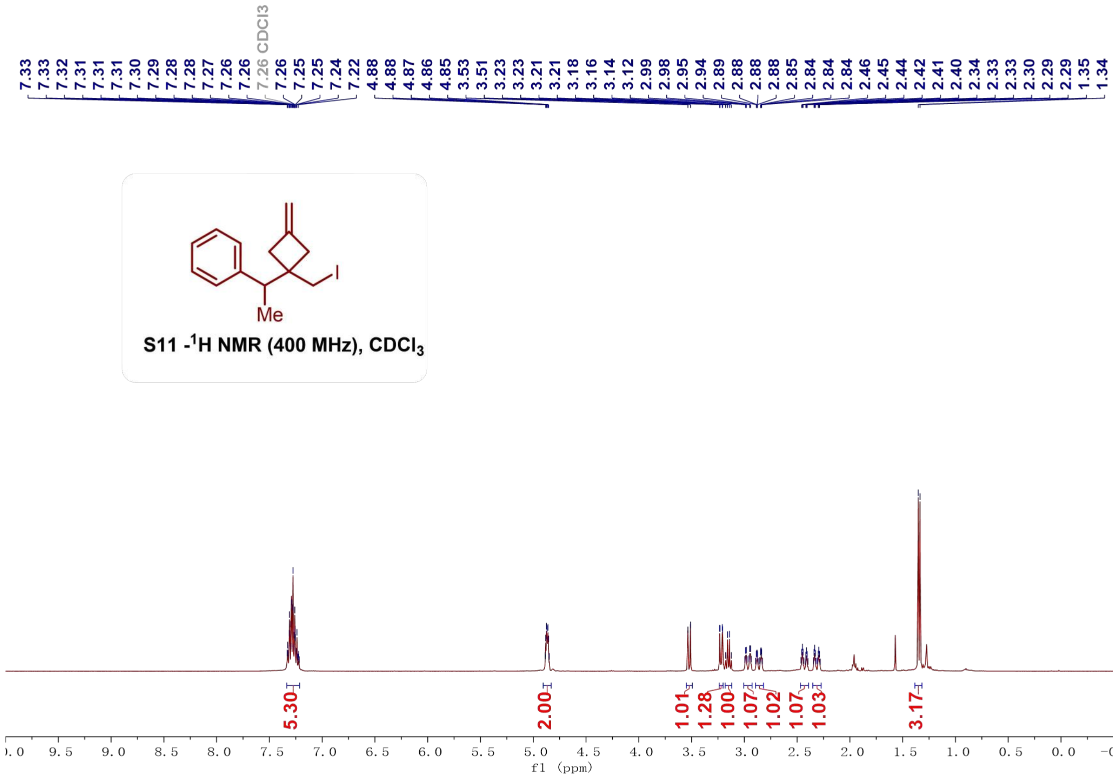

Supplementary Figure 44. ^{1}H NMR (400 MHz, $CDCl_3$) spectrum of compound **S11**

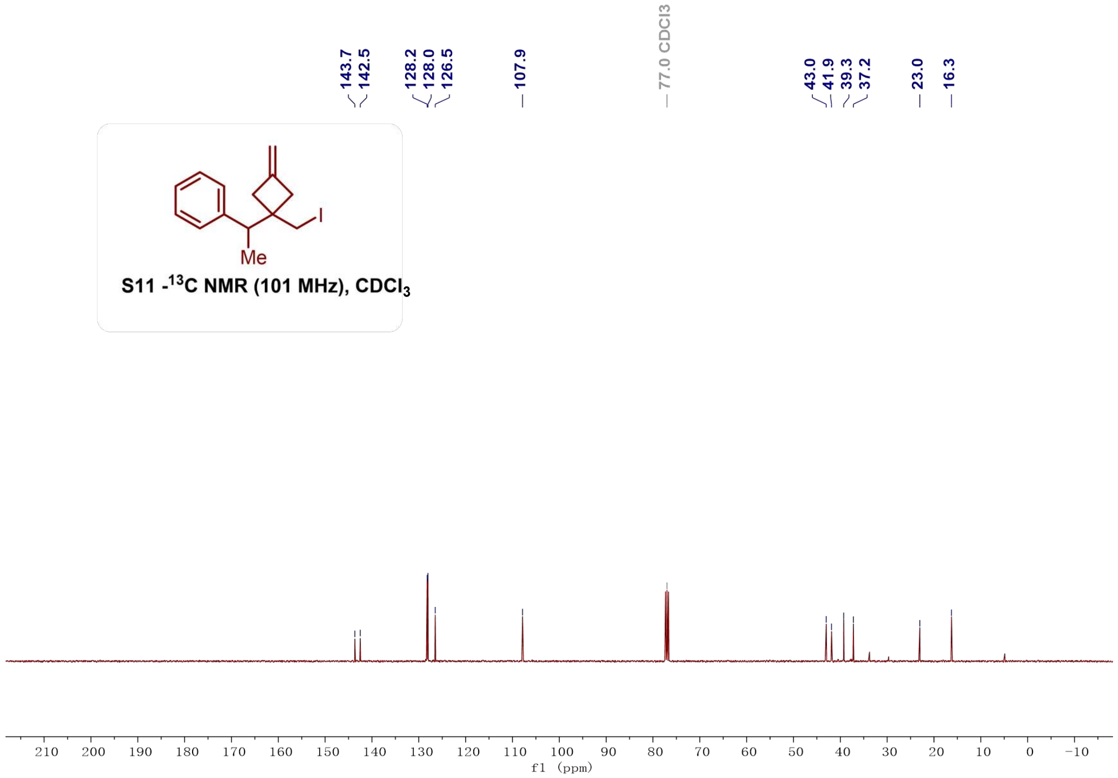

Supplementary Figure 45. ^{13}C NMR (101 MHz, $CDCl_3$) spectrum of compound **S11**

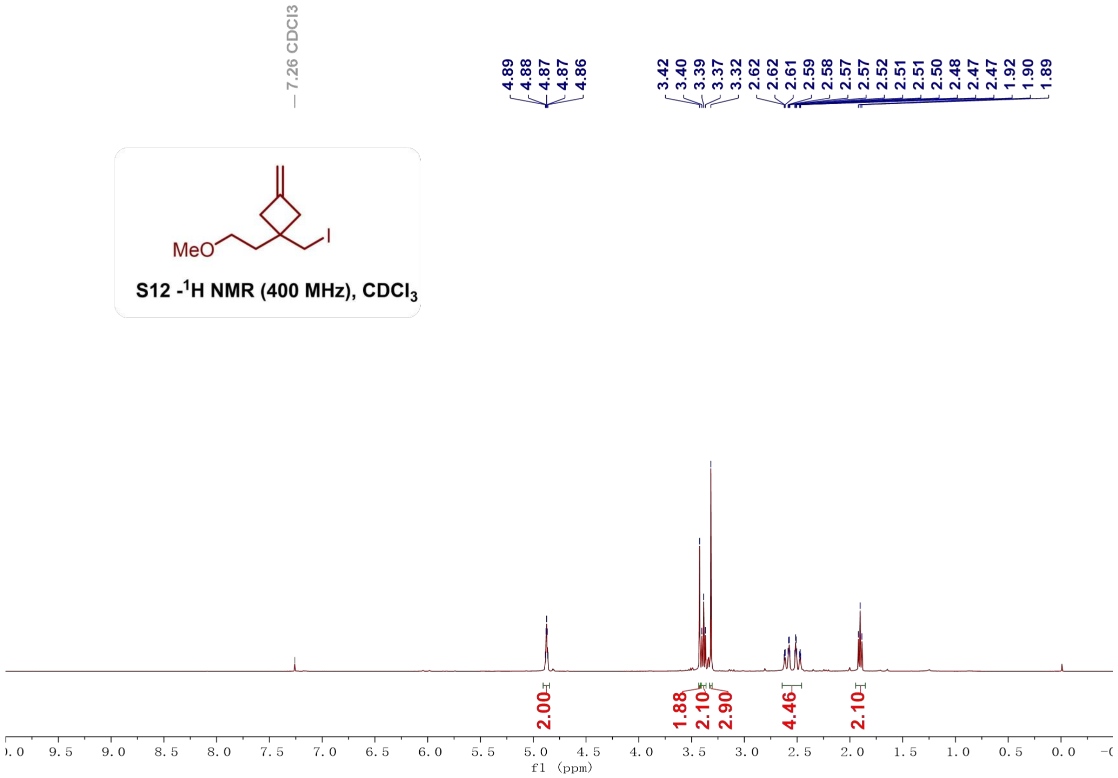

Supplementary Figure 46. ^{1}H NMR (400 MHz, $CDCl_3$) spectrum of compound **S12**

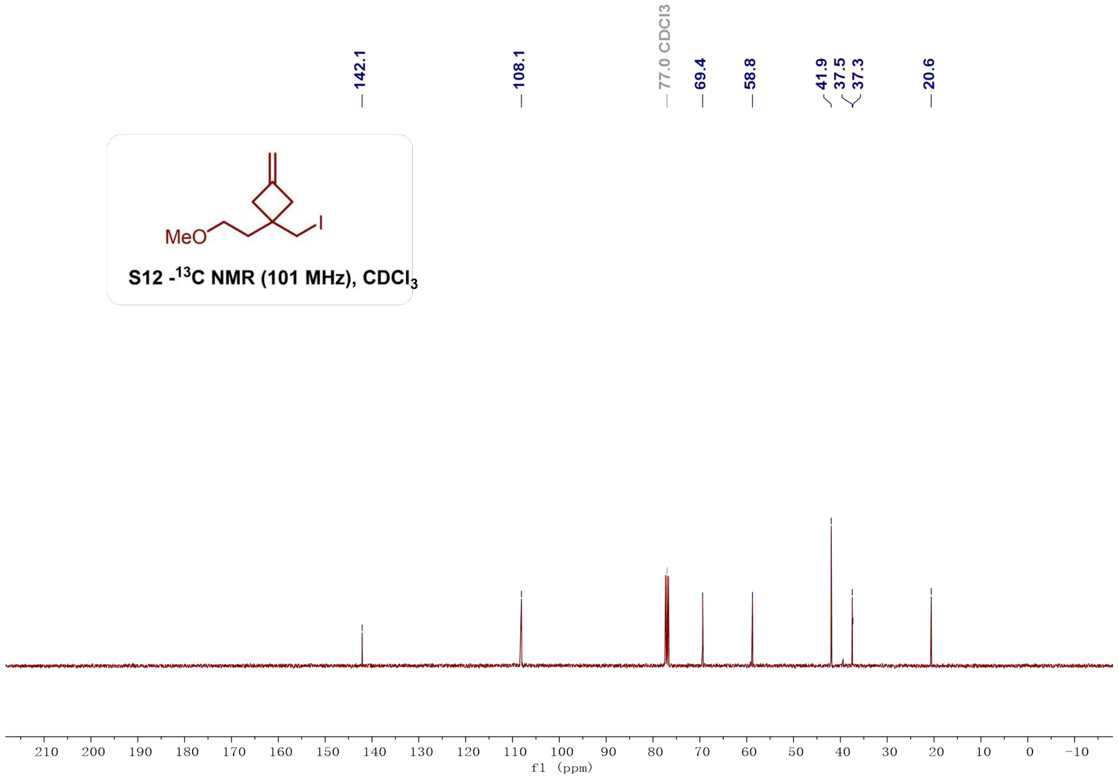

Supplementary Figure 47. ^{13}C NMR (101 MHz, $CDCl_3$) spectrum of compound **S12**

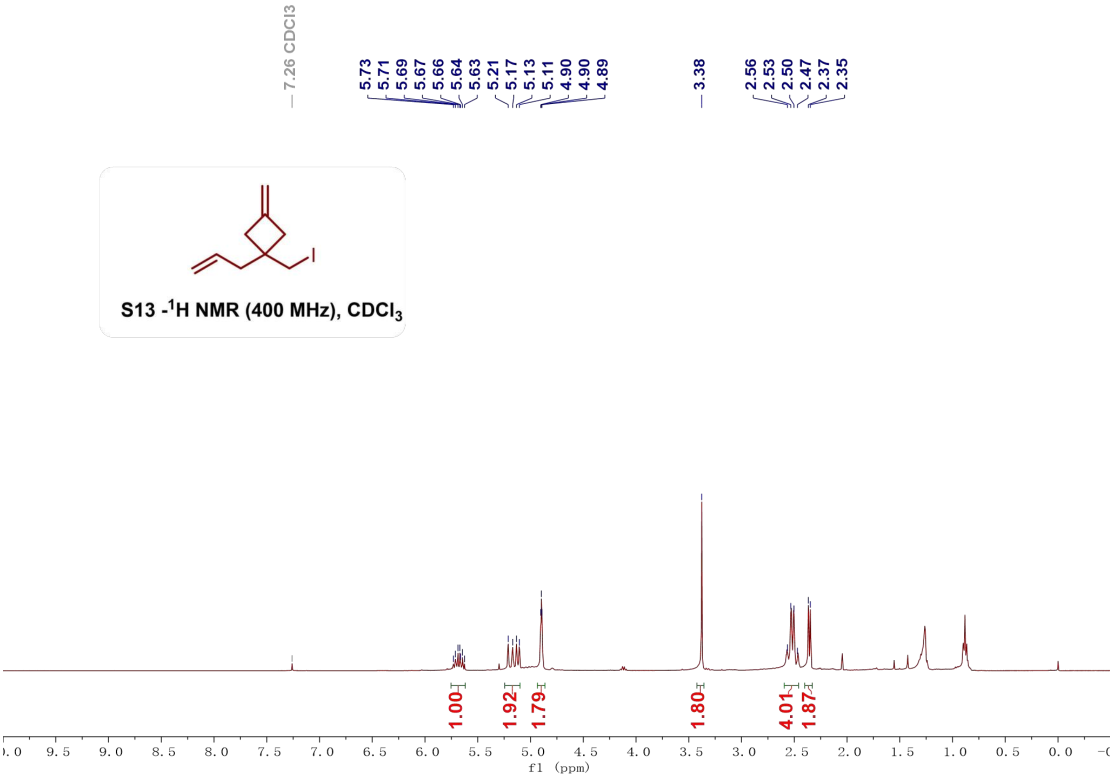

Supplementary Figure 48. ^{1}H NMR (400 MHz, $CDCl_3$) spectrum of compound **S13**

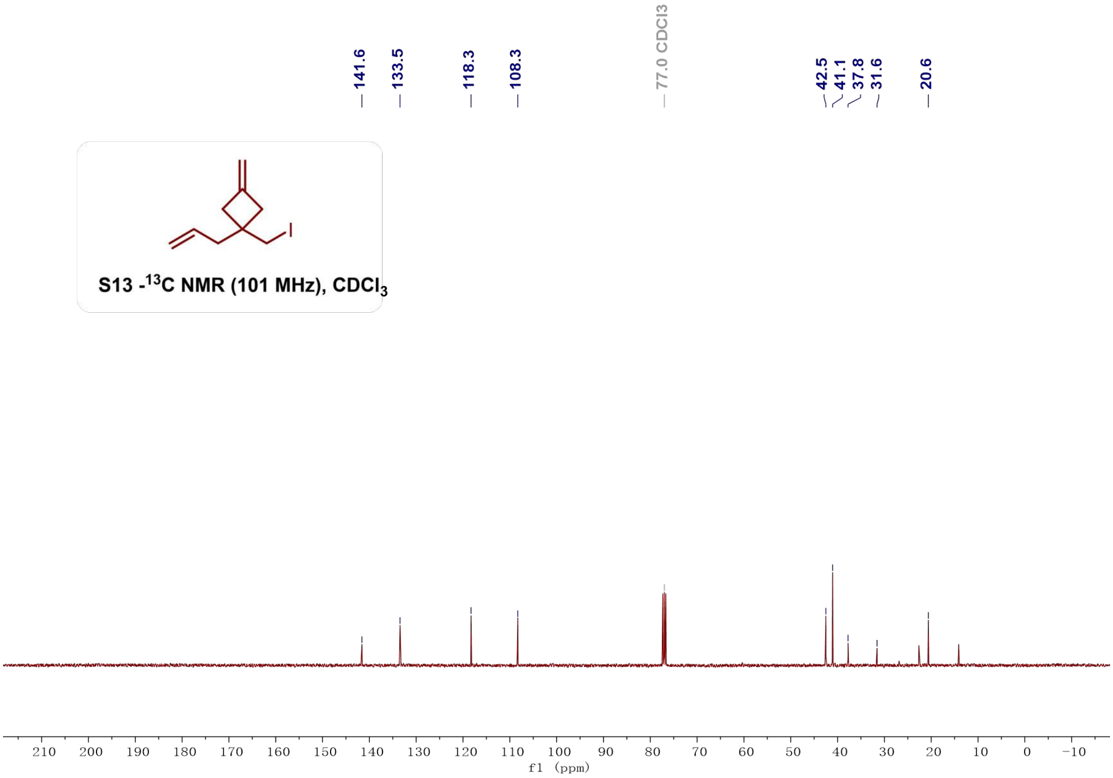

Supplementary Figure 49. ^{13}C NMR (101 MHz, $CDCl_3$) spectrum of compound **S13**

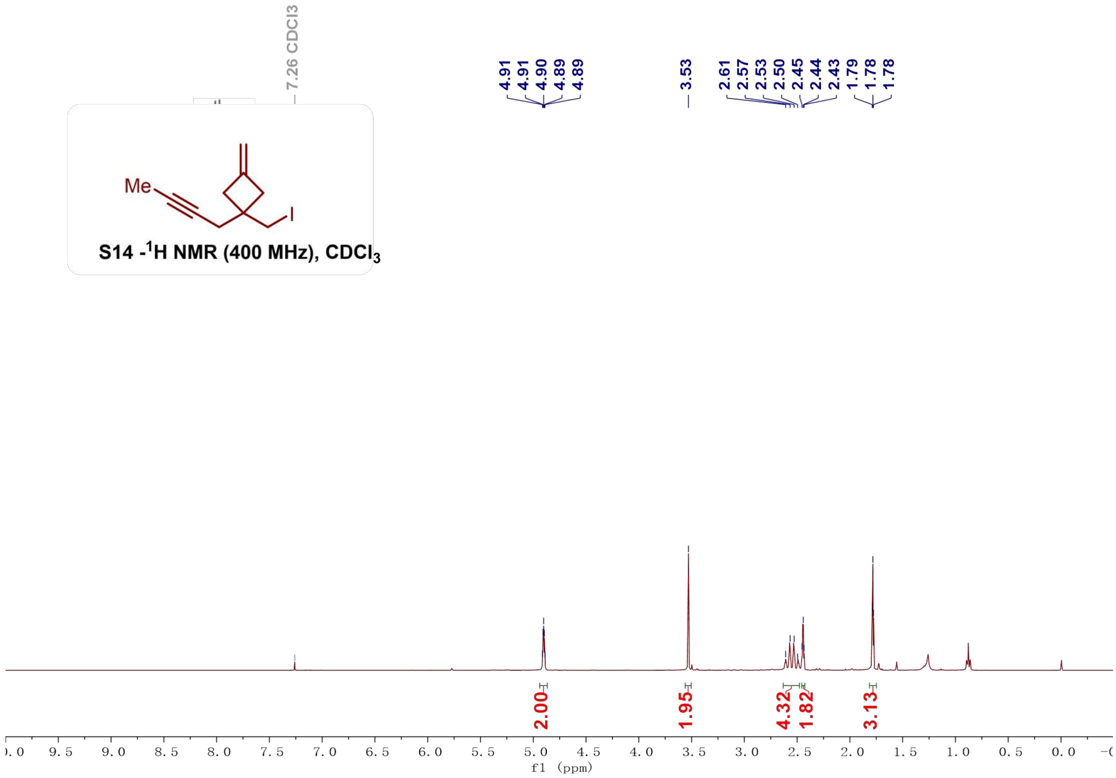

Supplementary Figure 50. ^{1}H NMR (400 MHz, $CDCl_3$) spectrum of compound **S14**

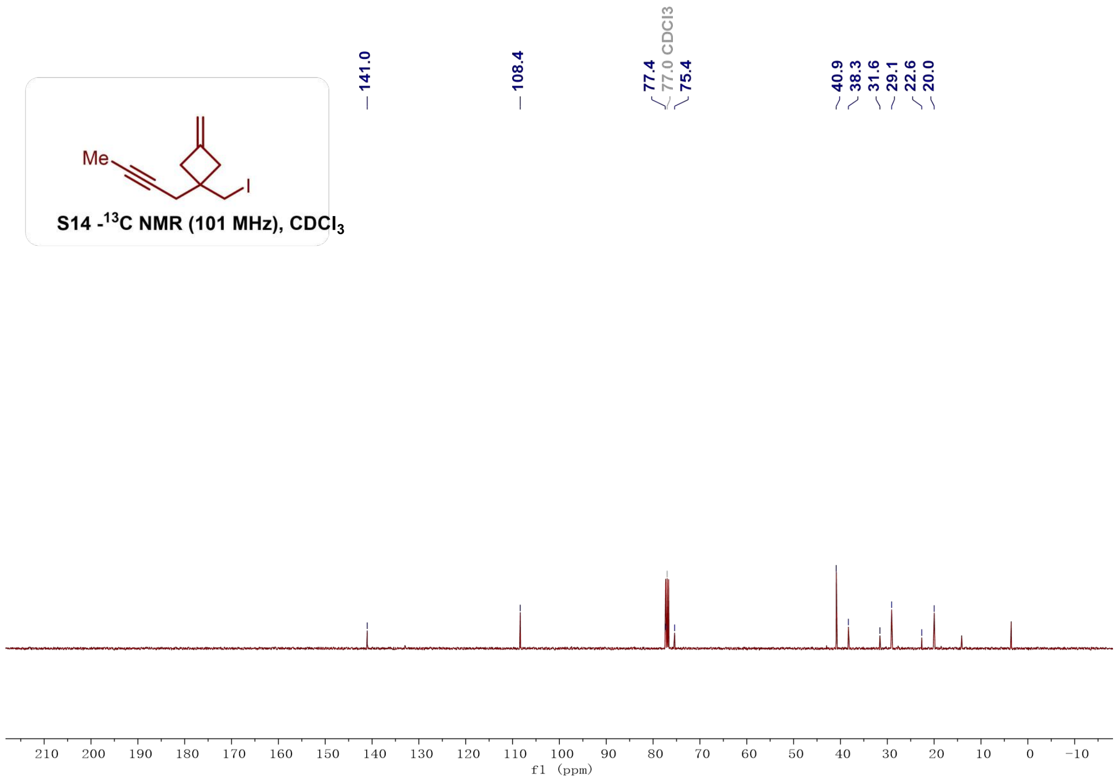

Supplementary Figure 51. ^{13}C NMR (101 MHz, $CDCl_3$) spectrum of compound **S14**

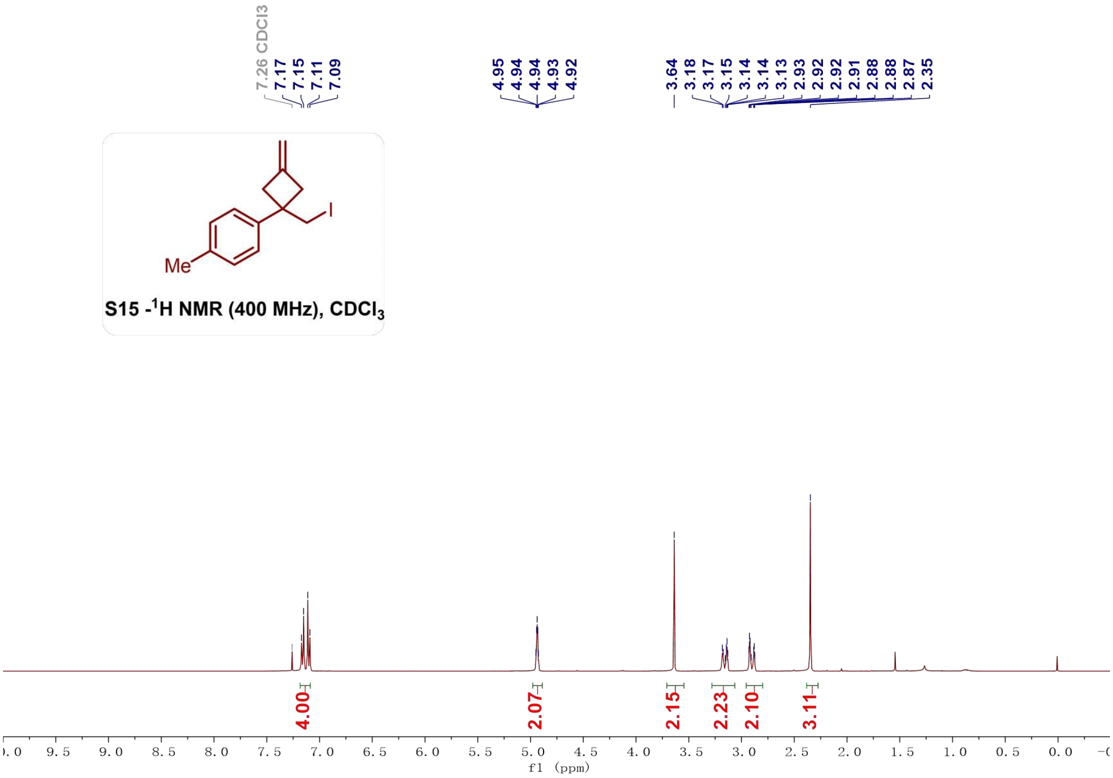

Supplementary Figure 52. ^{1}H NMR (400 MHz, $CDCl_3$) spectrum of compound **S15**

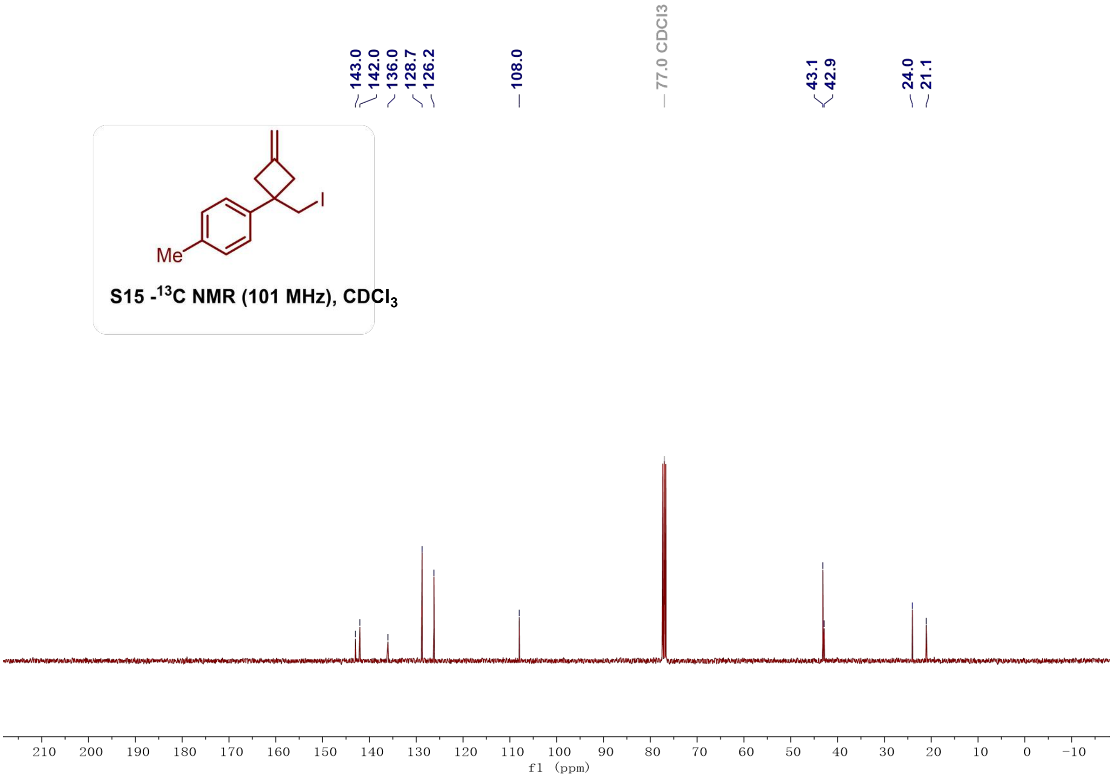

Supplementary Figure 53. ^{13}C NMR (101 MHz, $CDCl_3$) spectrum of compound **S15**

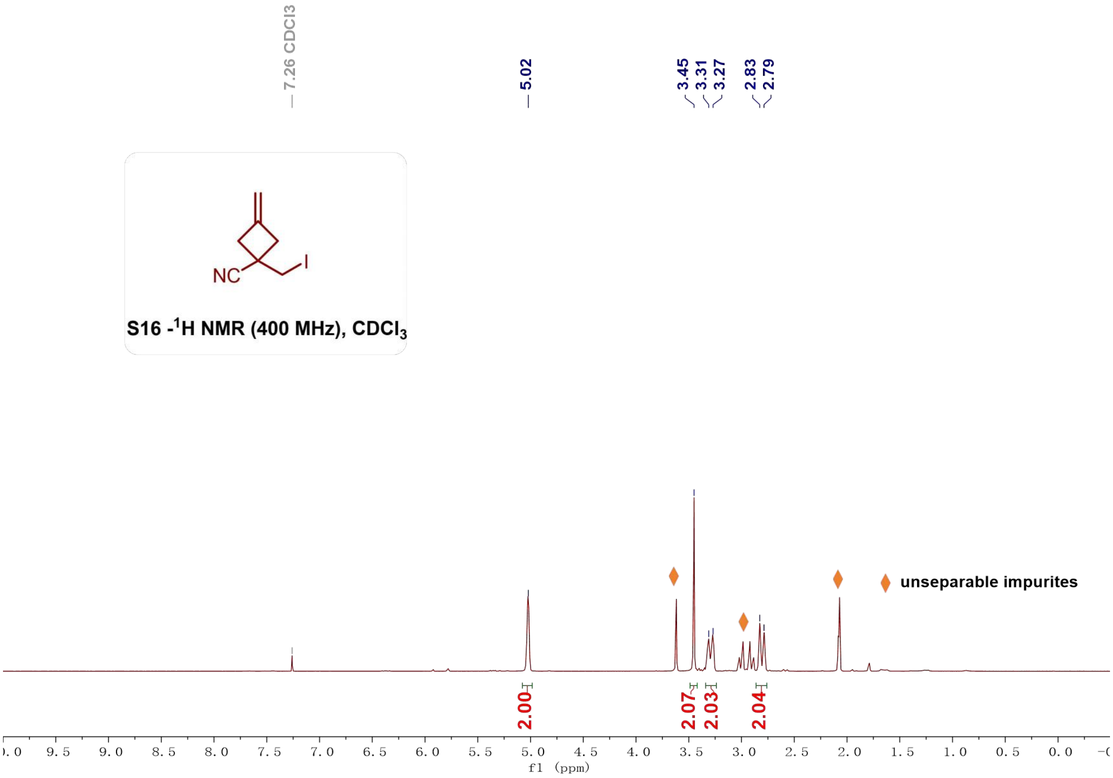

Supplementary Figure 54. ^{1}H NMR (400 MHz, $CDCl_3$) spectrum of compound **S16**

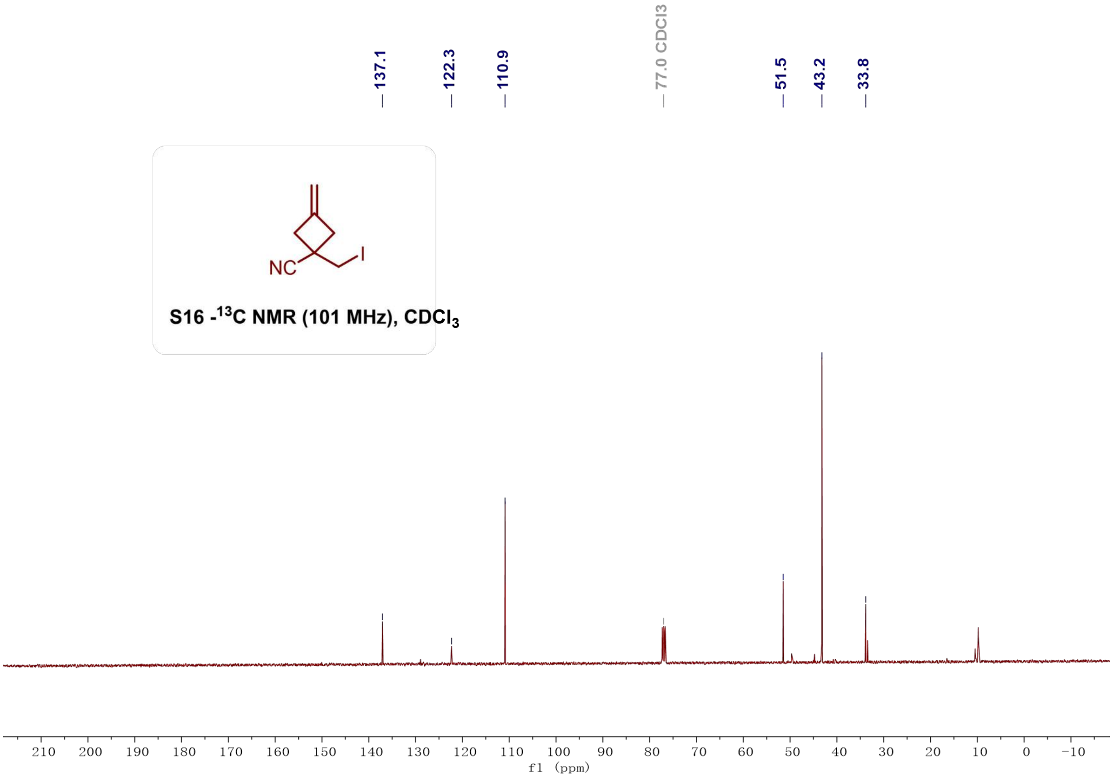

Supplementary Figure 55. ^{13}C NMR (101 MHz, $CDCl_3$) spectrum of compound **S16**

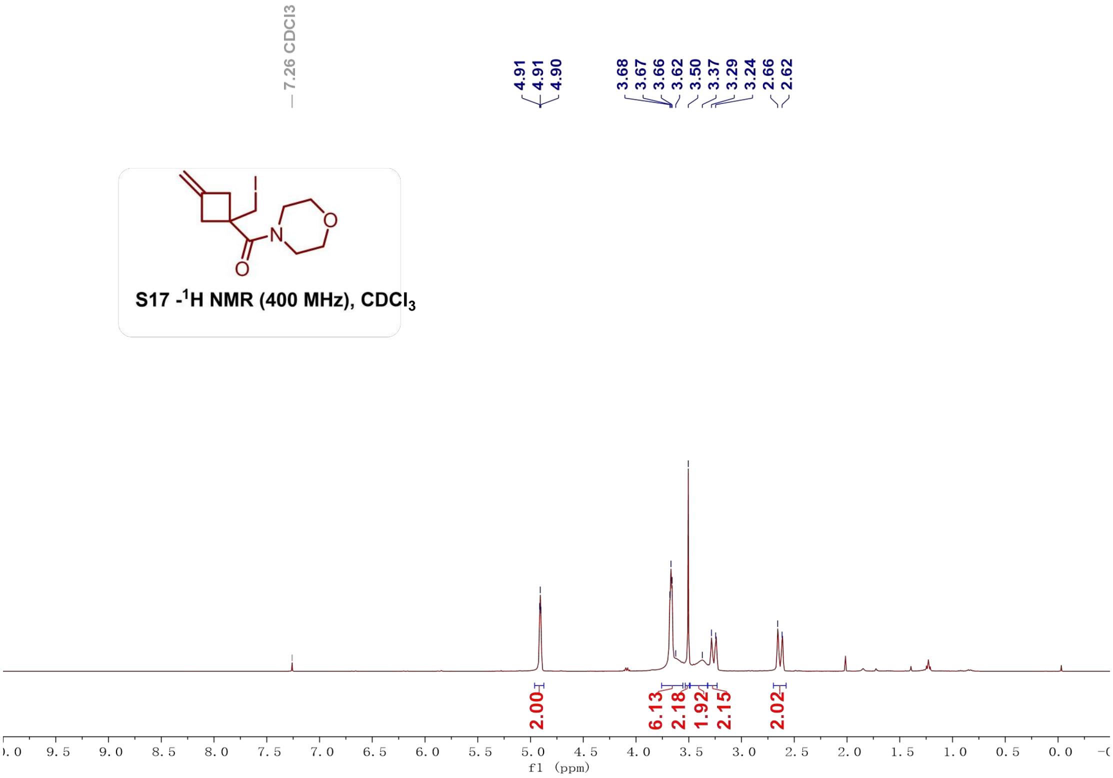

Supplementary Figure 56. ^{1}H NMR (400 MHz, $CDCl_3$) spectrum of compound **S17**

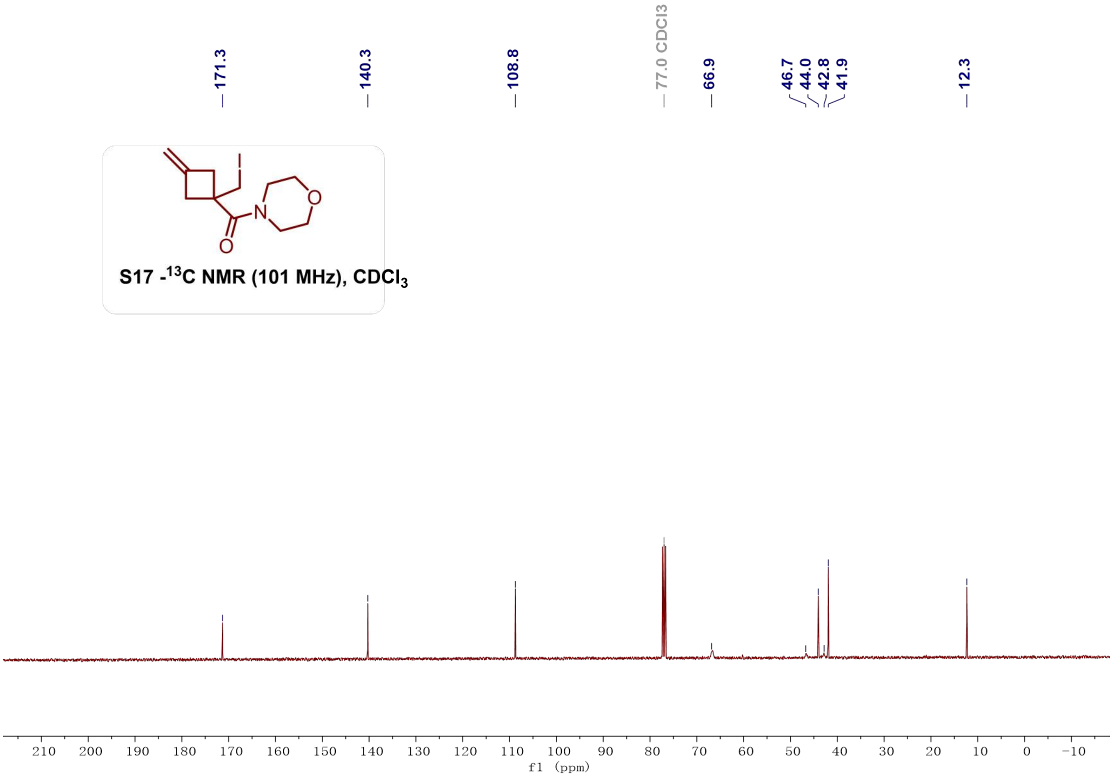

Supplementary Figure 57. ^{13}C NMR (101 MHz, $CDCl_3$) spectrum of compound **S17**

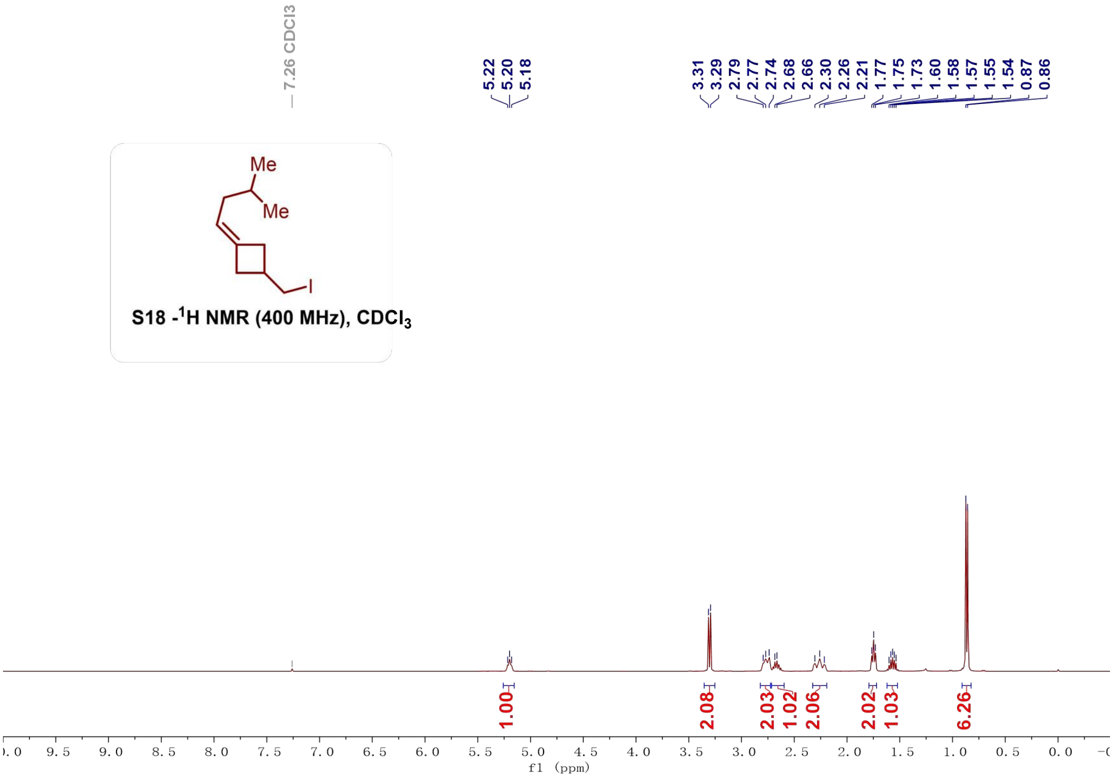

Supplementary Figure 58. ^{1}H NMR (400 MHz, $CDCl_3$) spectrum of compound **S18**

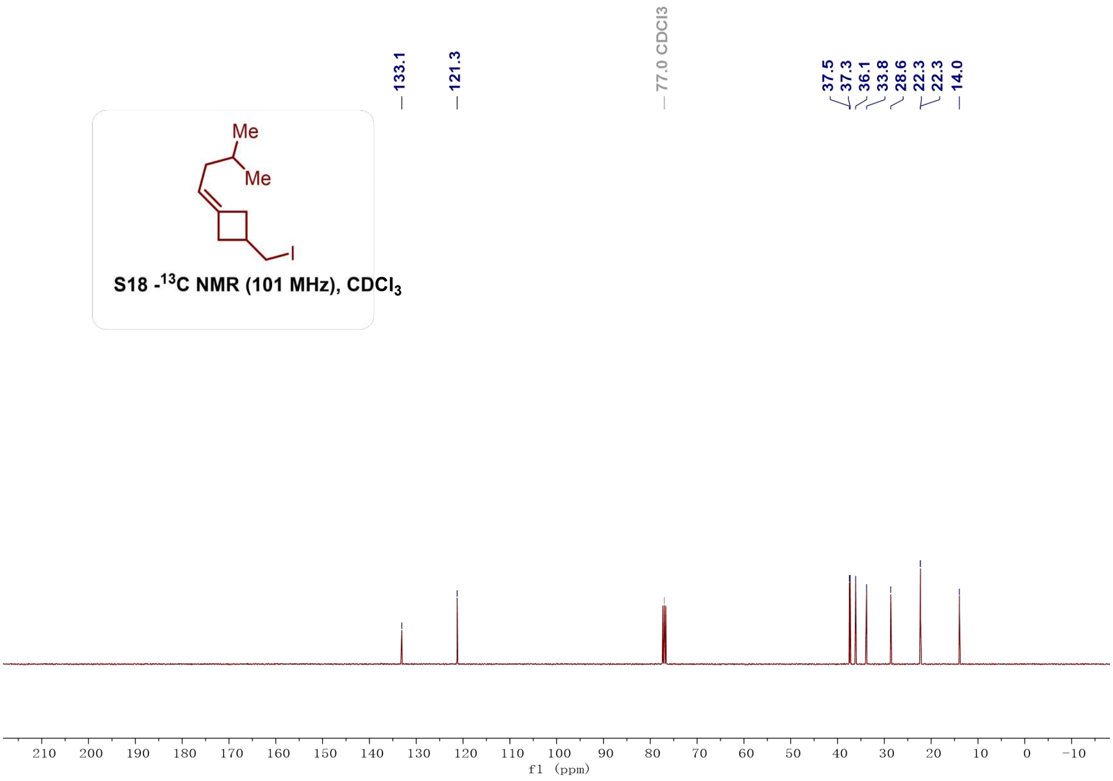

Supplementary Figure 59. ^{13}C NMR (101 MHz, $CDCl_3$) spectrum of compound **S18**

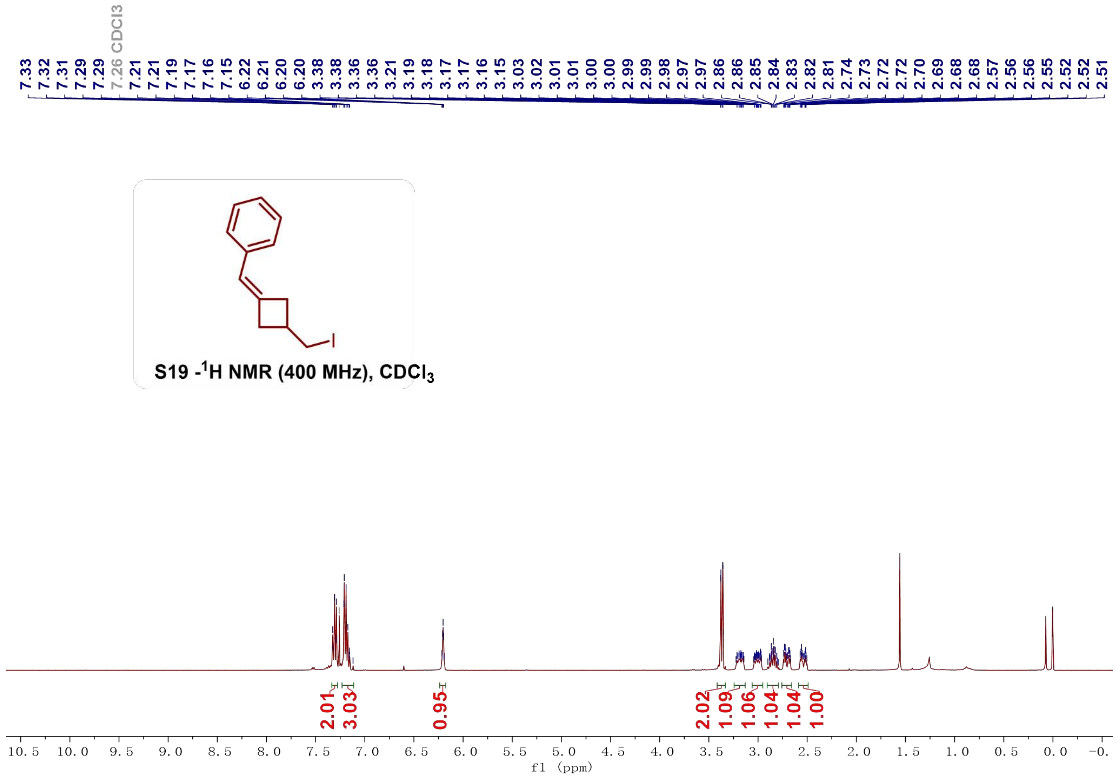

Supplementary Figure 60. ^{1}H NMR (400 MHz, $CDCl_3$) spectrum of compound **S19**

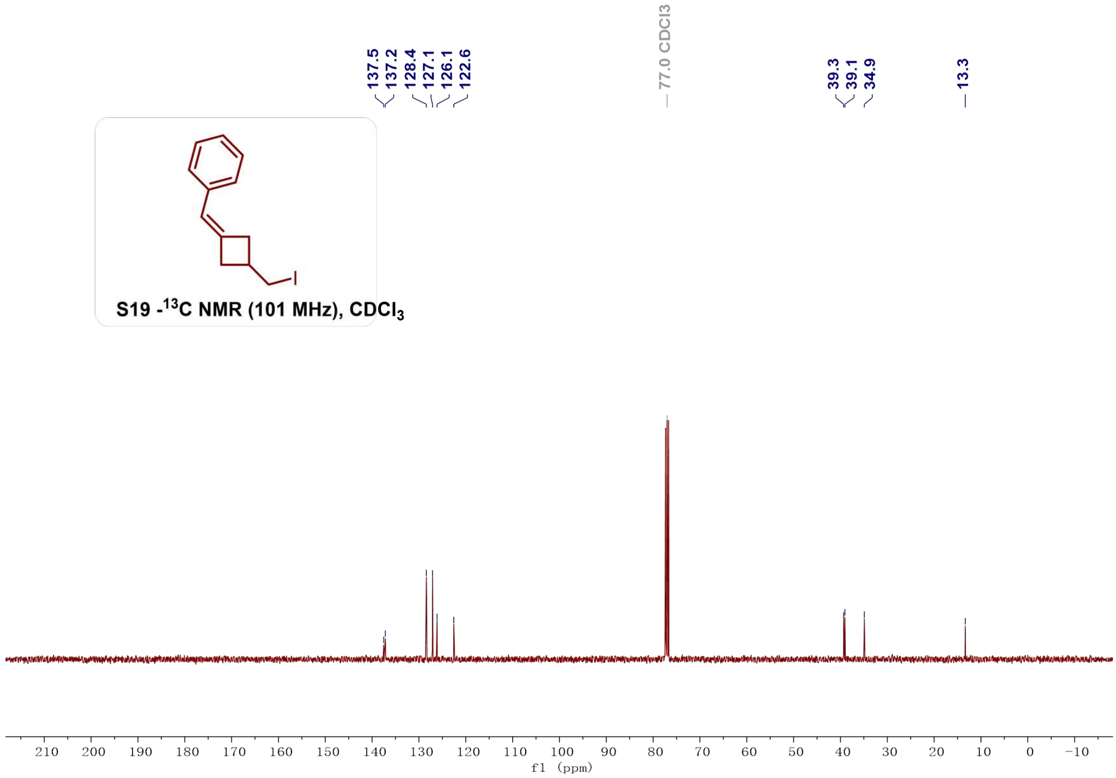

Supplementary Figure 61. ^{13}C NMR (101 MHz, $CDCl_3$) spectrum of compound **S19**

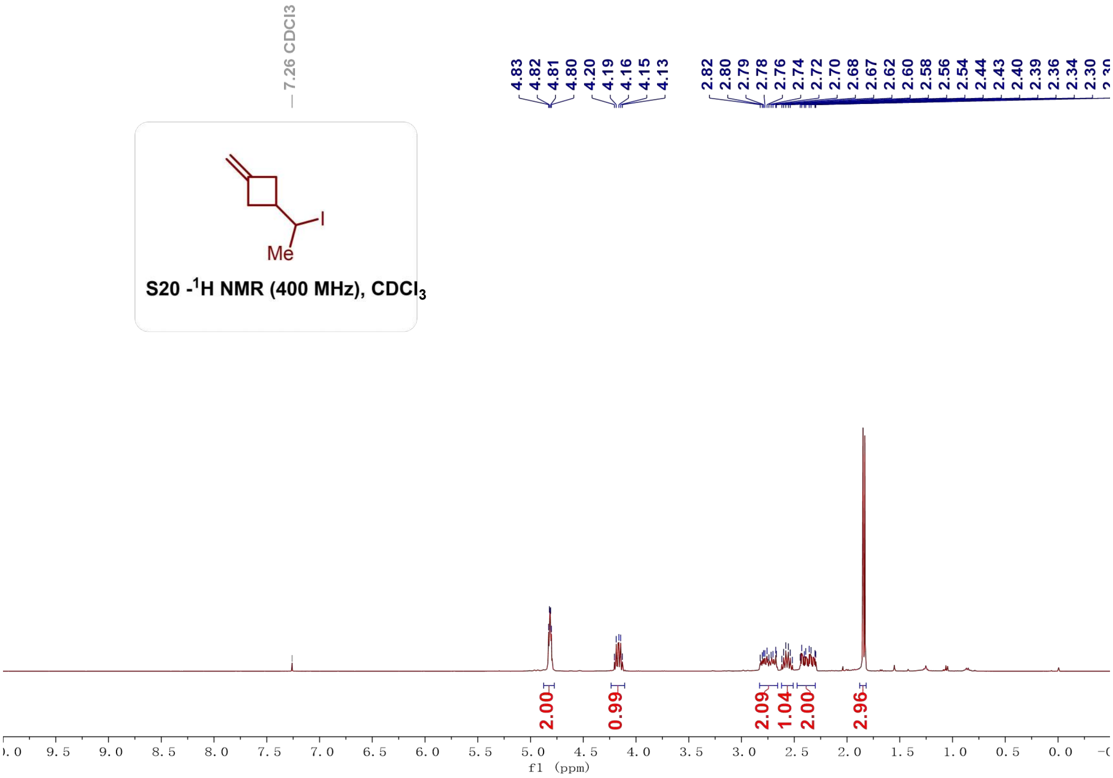

Supplementary Figure 62. ^{1}H NMR (400 MHz, $CDCl_3$) spectrum of compound **S20**

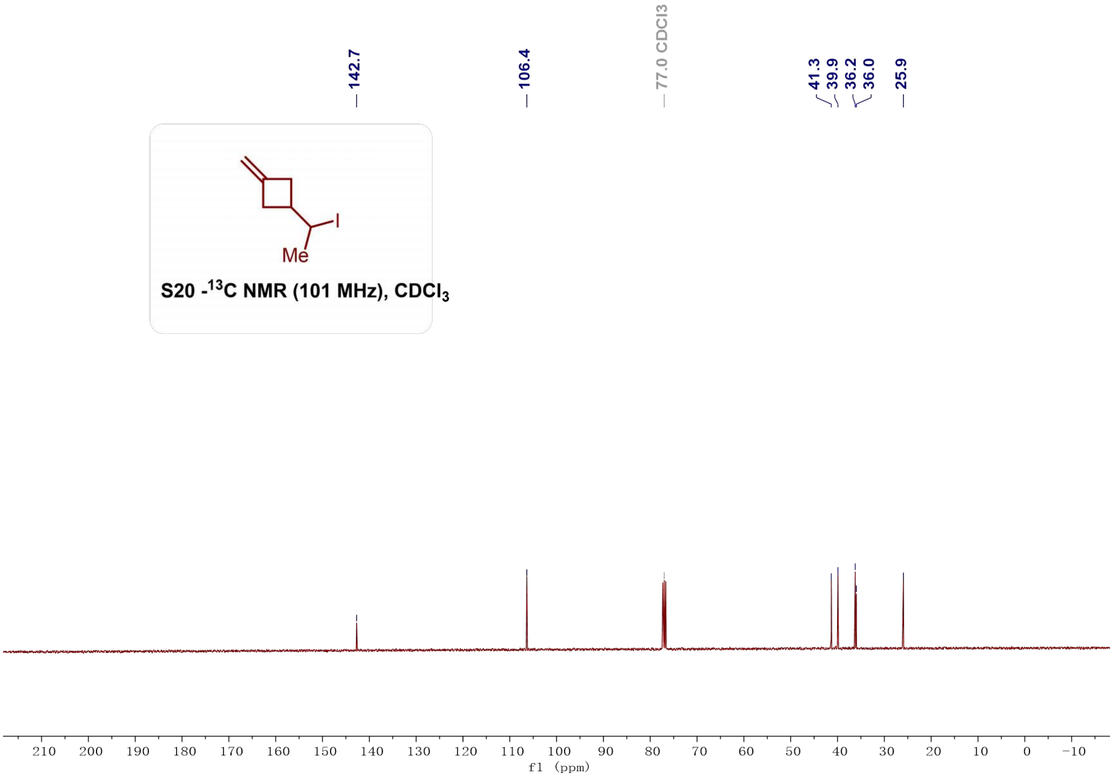

Supplementary Figure 63. ^{13}C NMR (101 MHz, $CDCl_3$) spectrum of compound **S20**

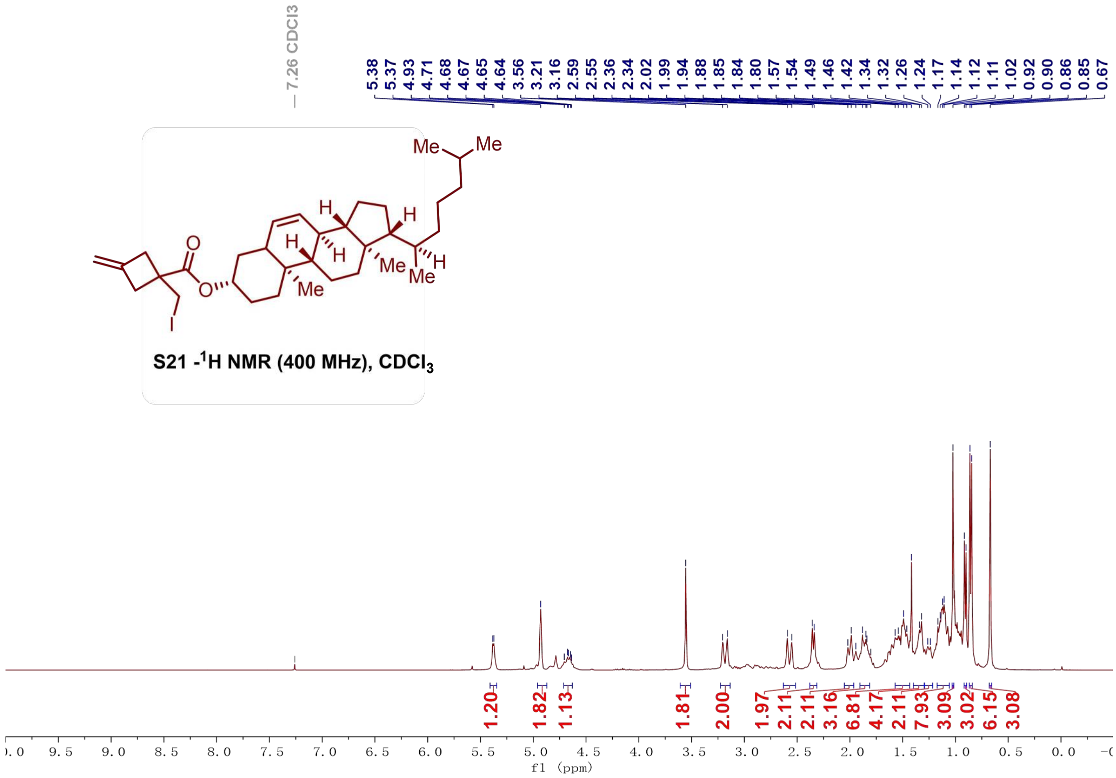

Supplementary Figure 64. ^{1}H NMR (400 MHz, $CDCl_3$) spectrum of compound **S21**

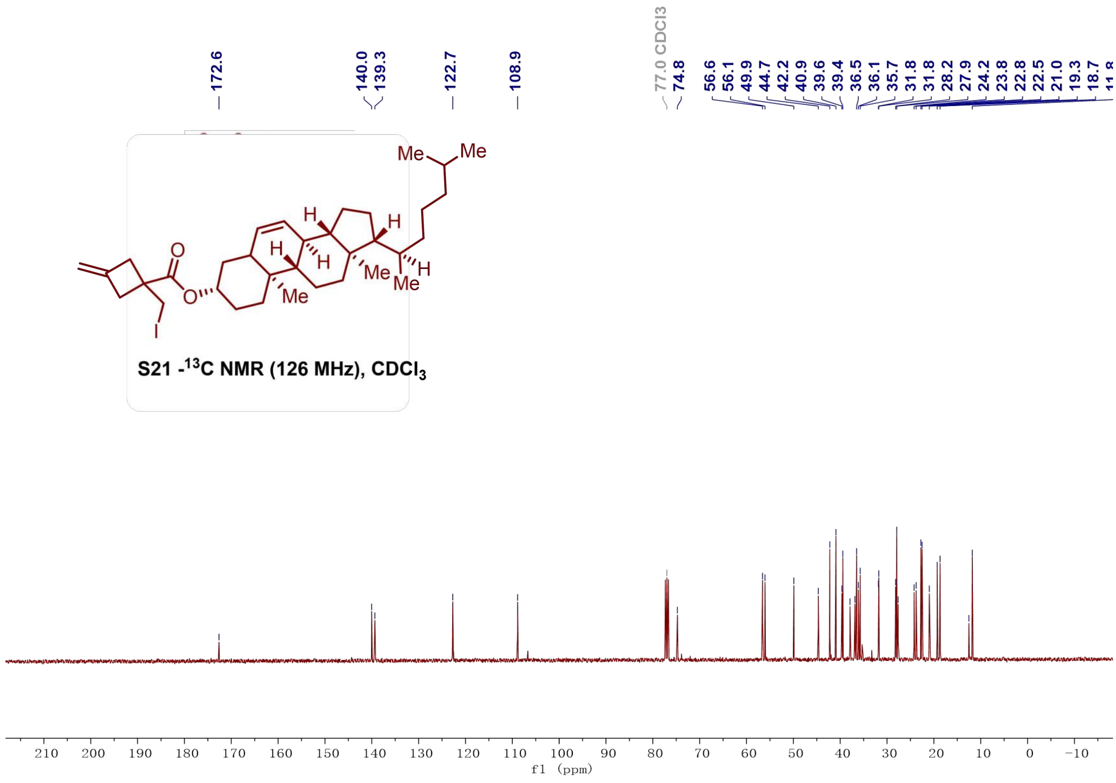

Supplementary Figure 65. ^{13}C NMR (101 MHz, $CDCl_3$) spectrum of compound **S21**

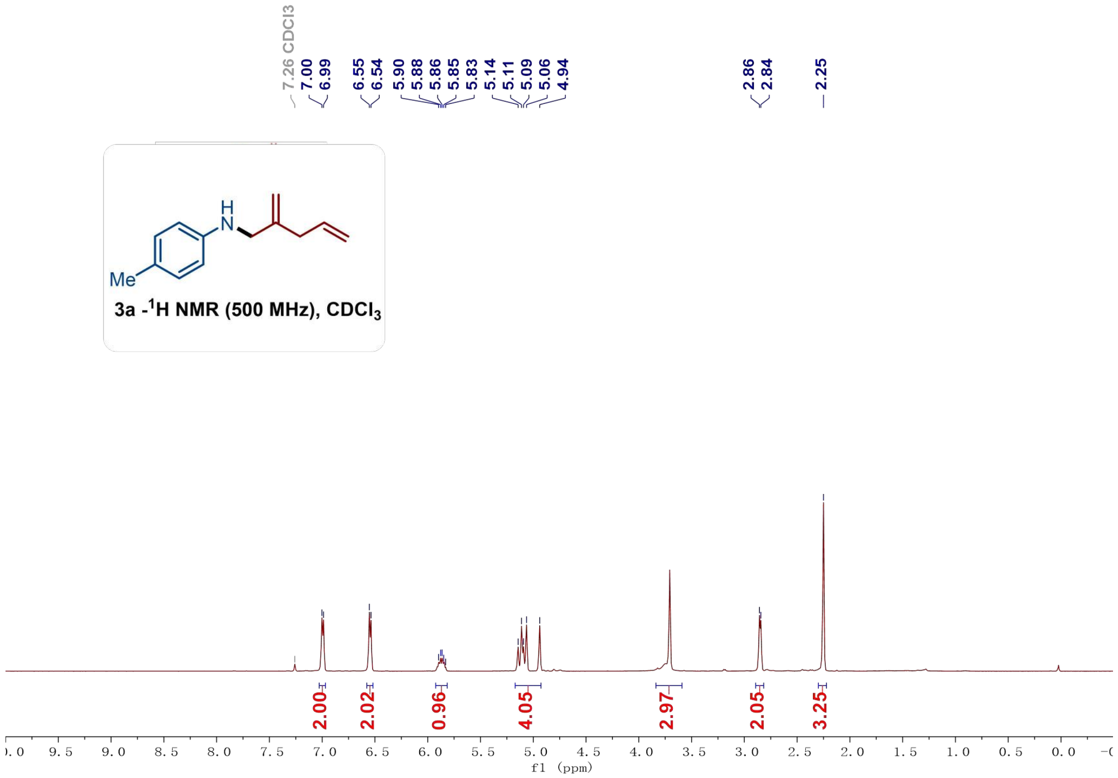

Supplementary Figure 66. ^{1}H NMR (500 MHz, $CDCl_3$) spectrum of compound **3a**

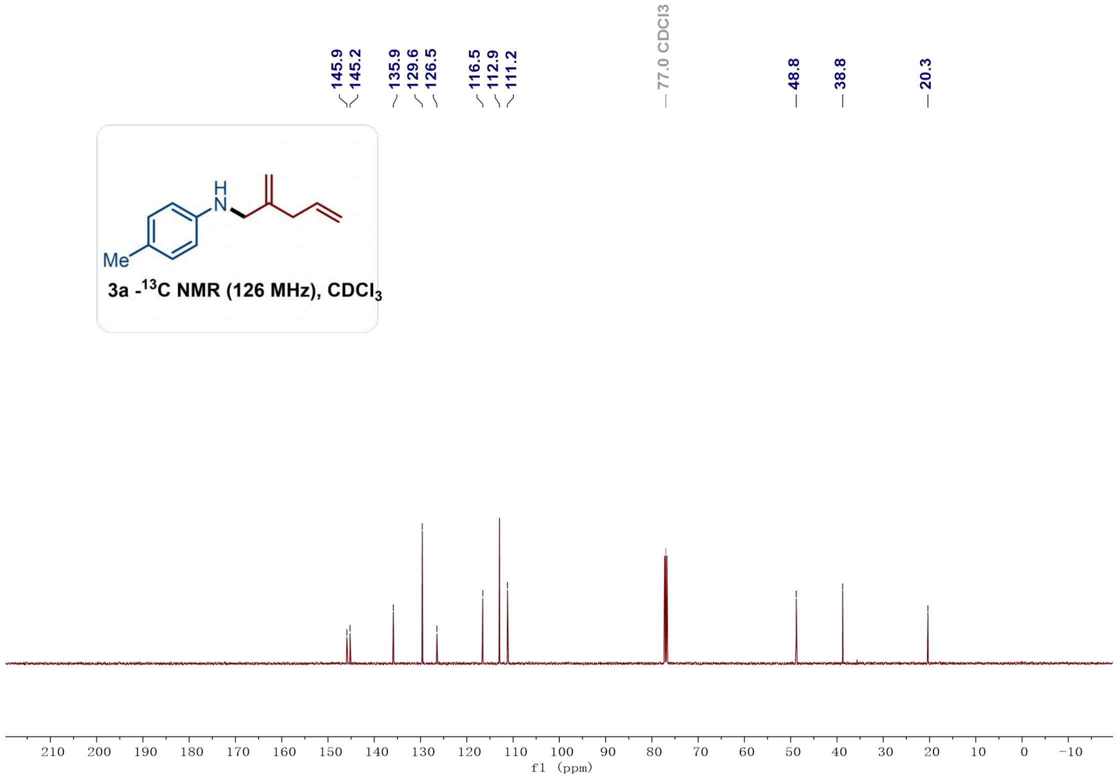

Supplementary Figure 67. ^{13}C NMR (126 MHz, $CDCl_3$) spectrum of compound **3a**

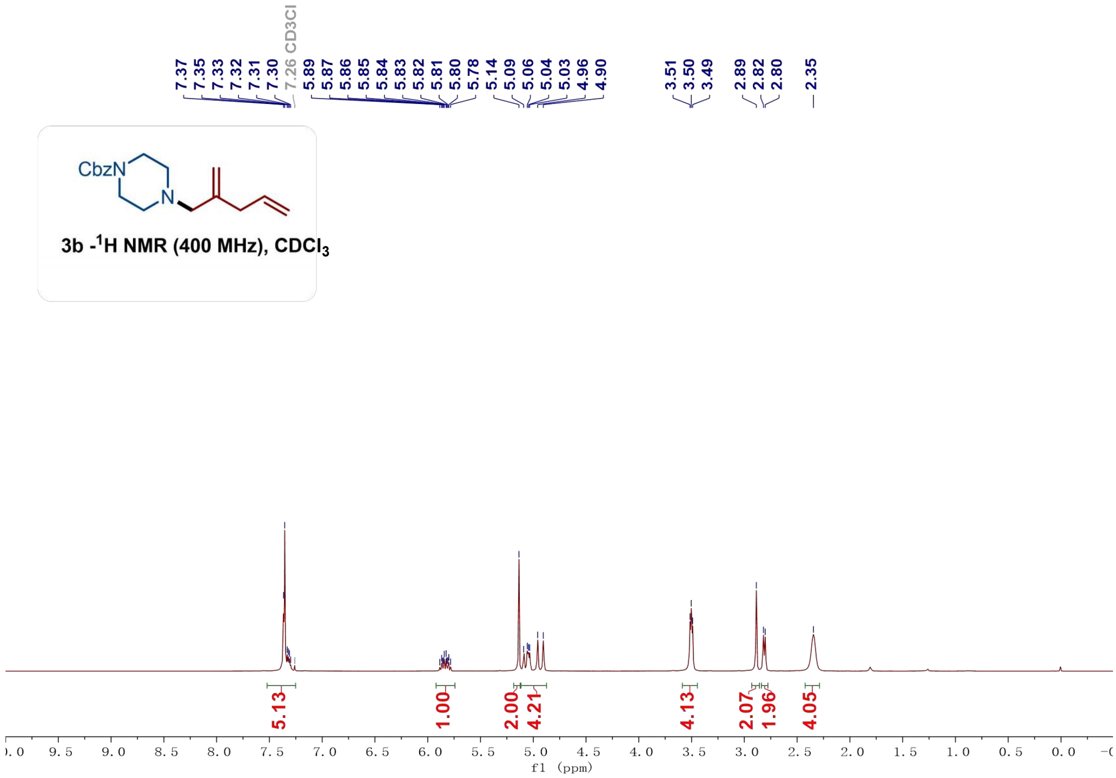

Supplementary Figure 68. ^{1}H NMR (400 MHz, $CDCl_3$) spectrum of compound **3b**

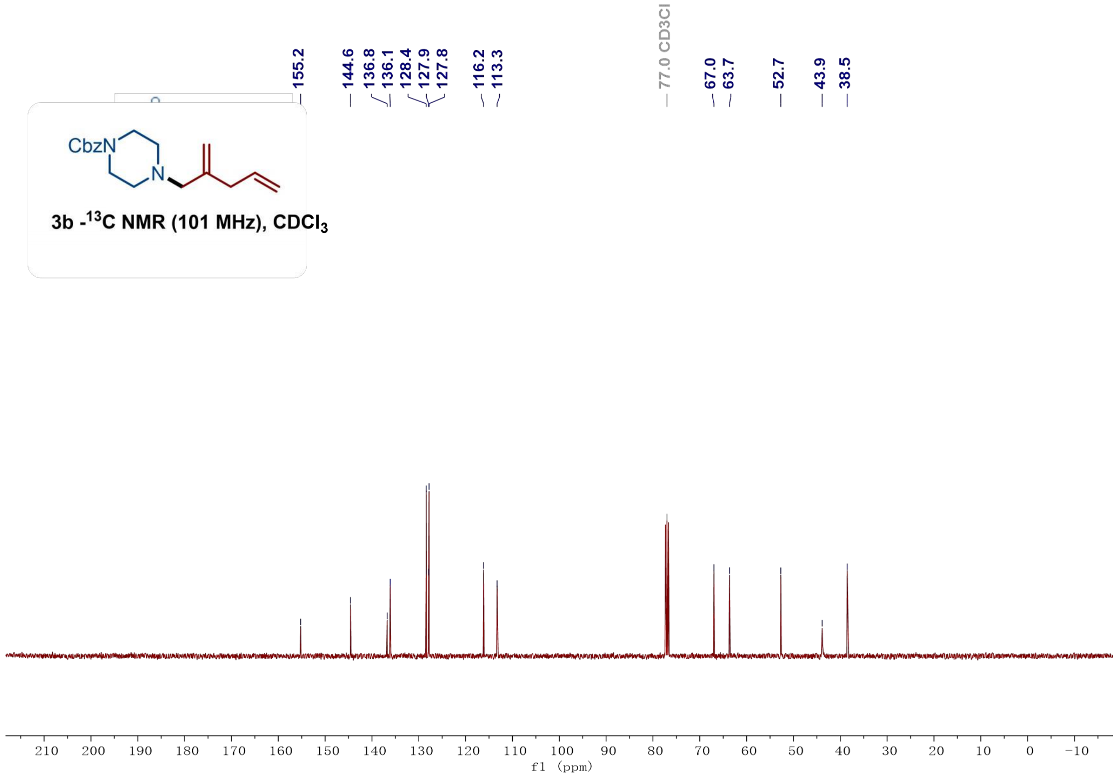

Supplementary Figure 69. ^{13}C NMR (101 MHz, $CDCl_3$) spectrum of compound **3b**

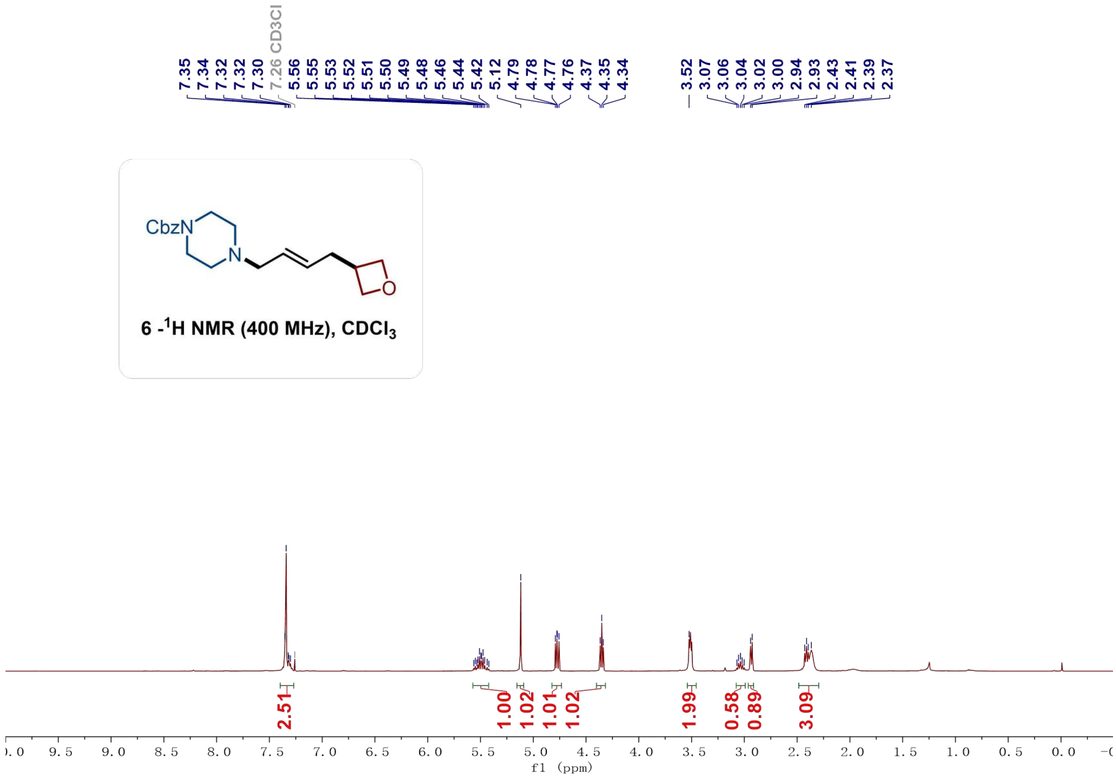

Supplementary Figure 70. ^{1}H NMR (400 MHz, $CDCl_3$) spectrum of compound **6**

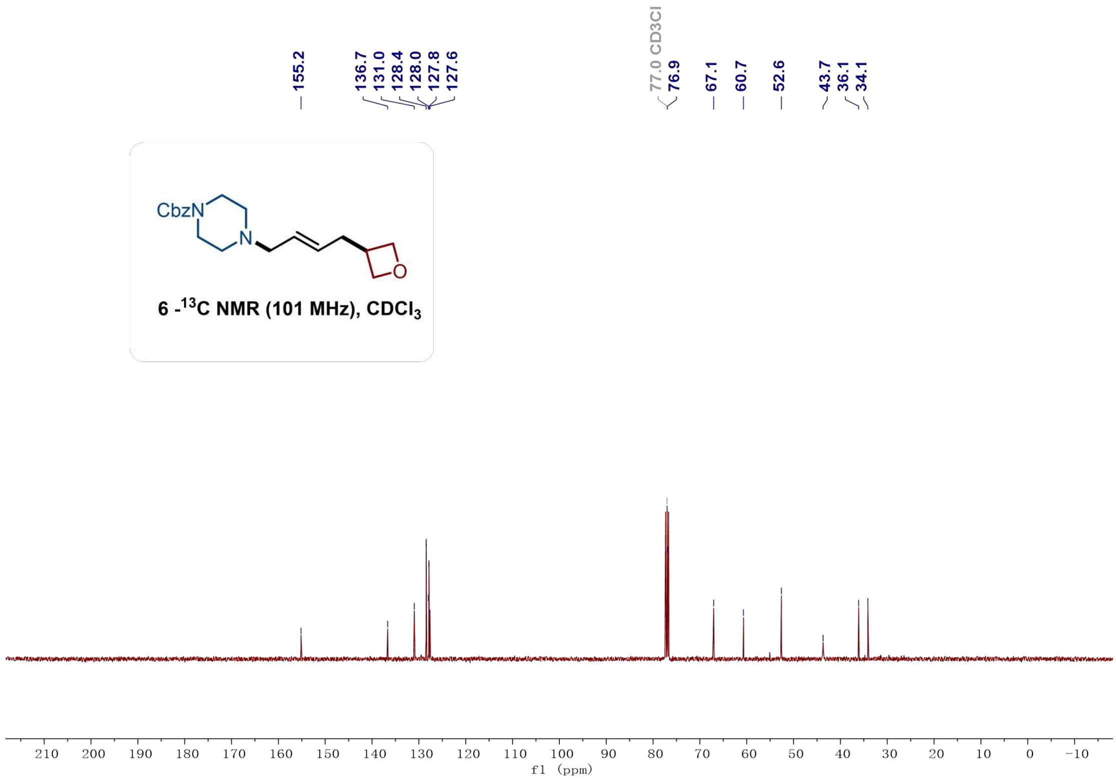

Supplementary Figure 71. ^{13}C NMR (101 MHz, $CDCl_3$) spectrum of compound **6**

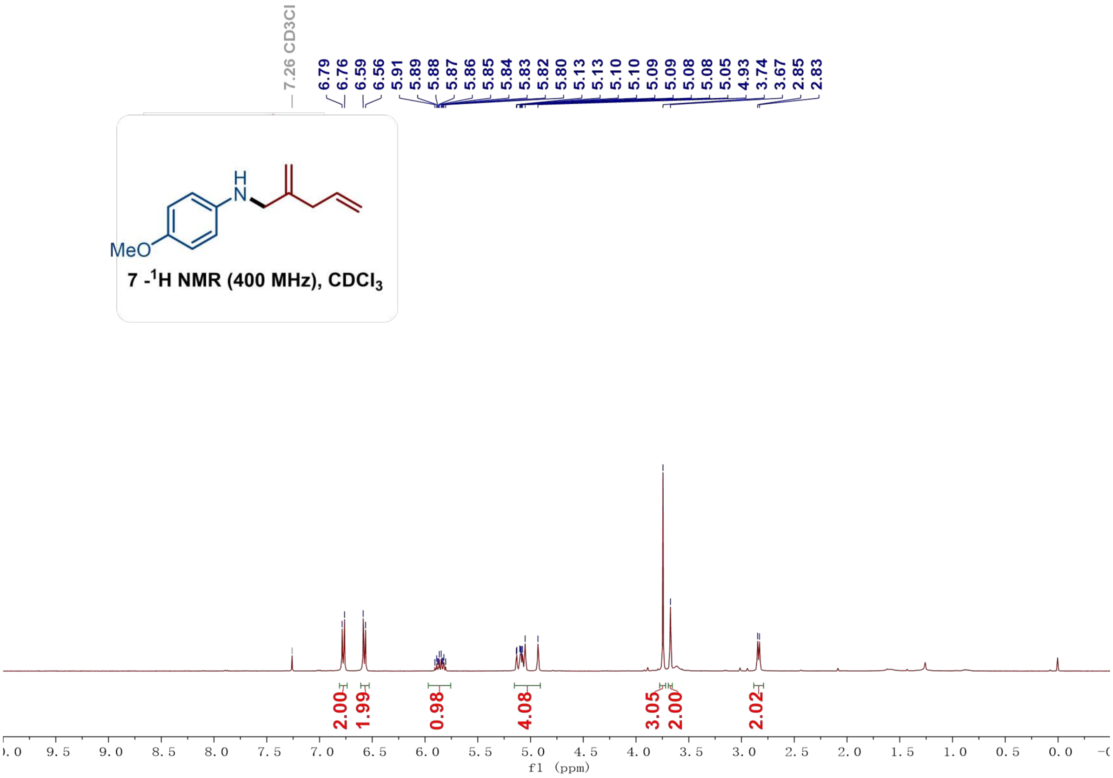

Supplementary Figure 72. ^{1}H NMR (400 MHz, $CDCl_3$) spectrum of compound **7**

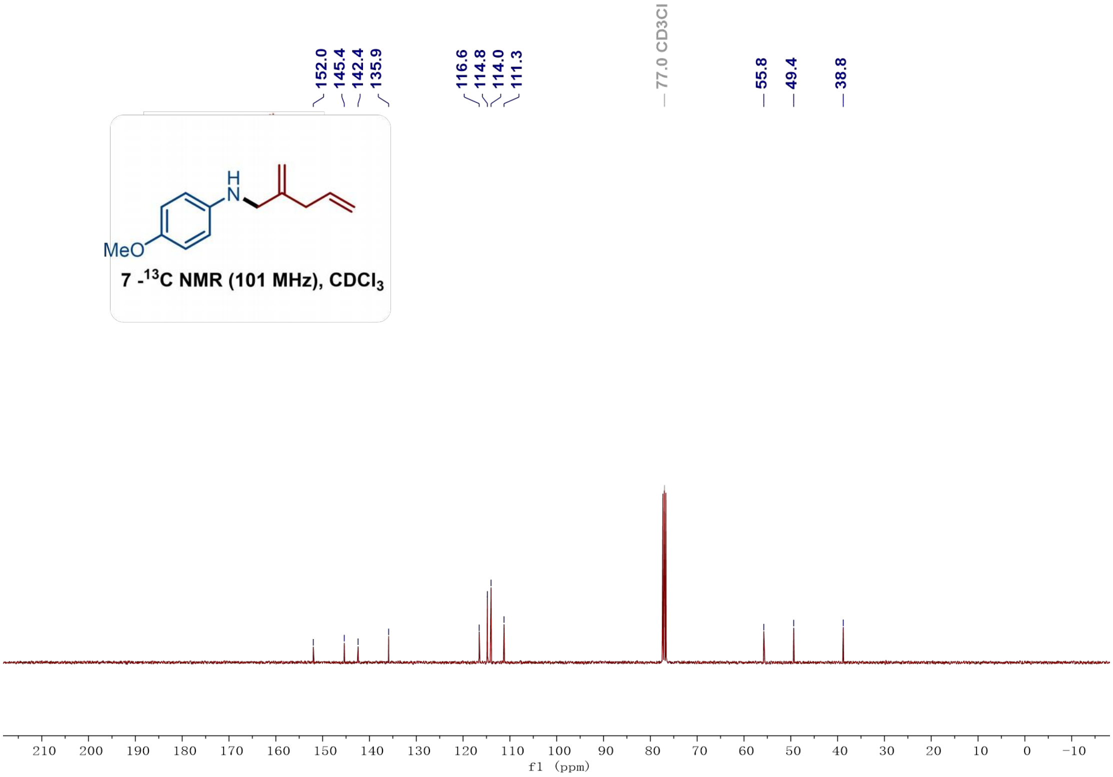

Supplementary Figure 73. ^{13}C NMR (101 MHz, $CDCl_3$) spectrum of compound **7**

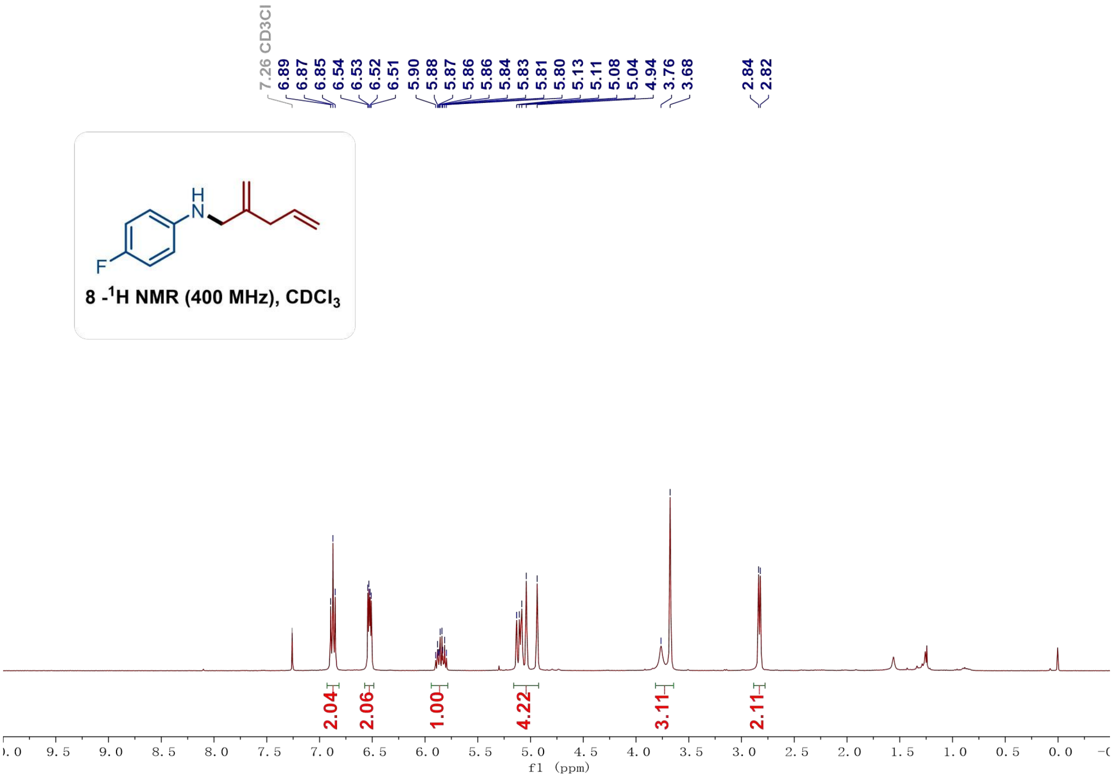

Supplementary Figure 74. ^{1}H NMR (400 MHz, $CDCl_3$) spectrum of compound **8**

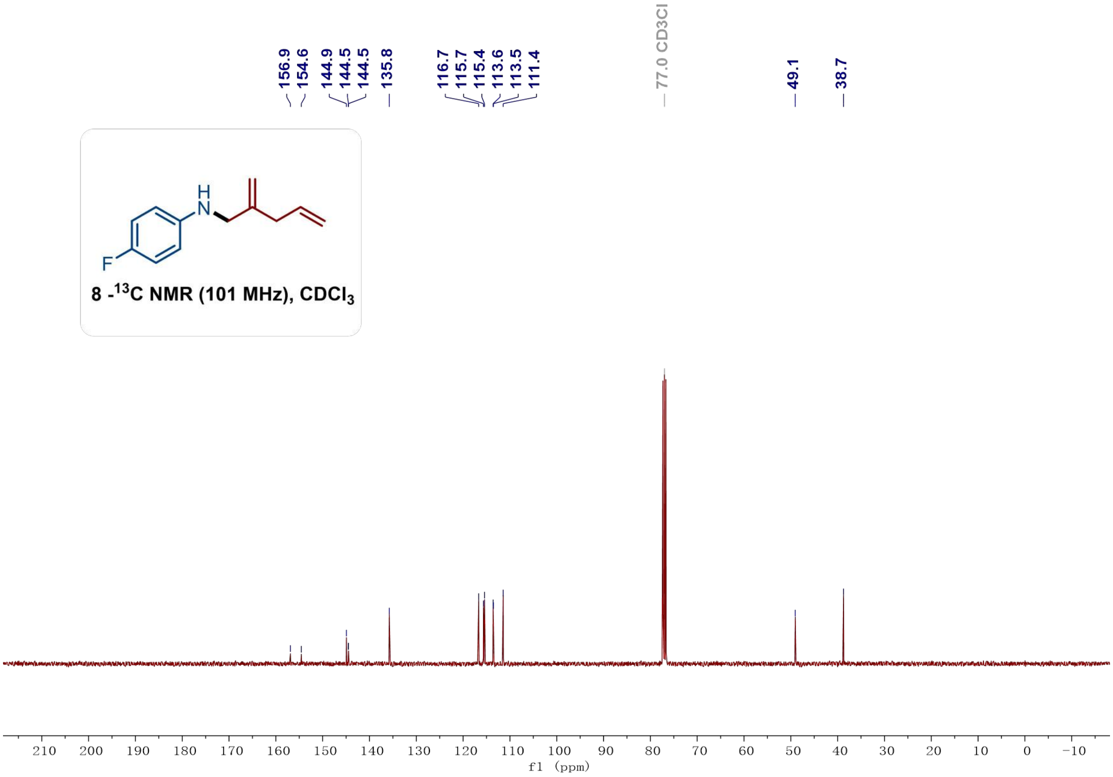

Supplementary Figure 75. ^{13}C NMR (101 MHz, $CDCl_3$) spectrum of compound **8**

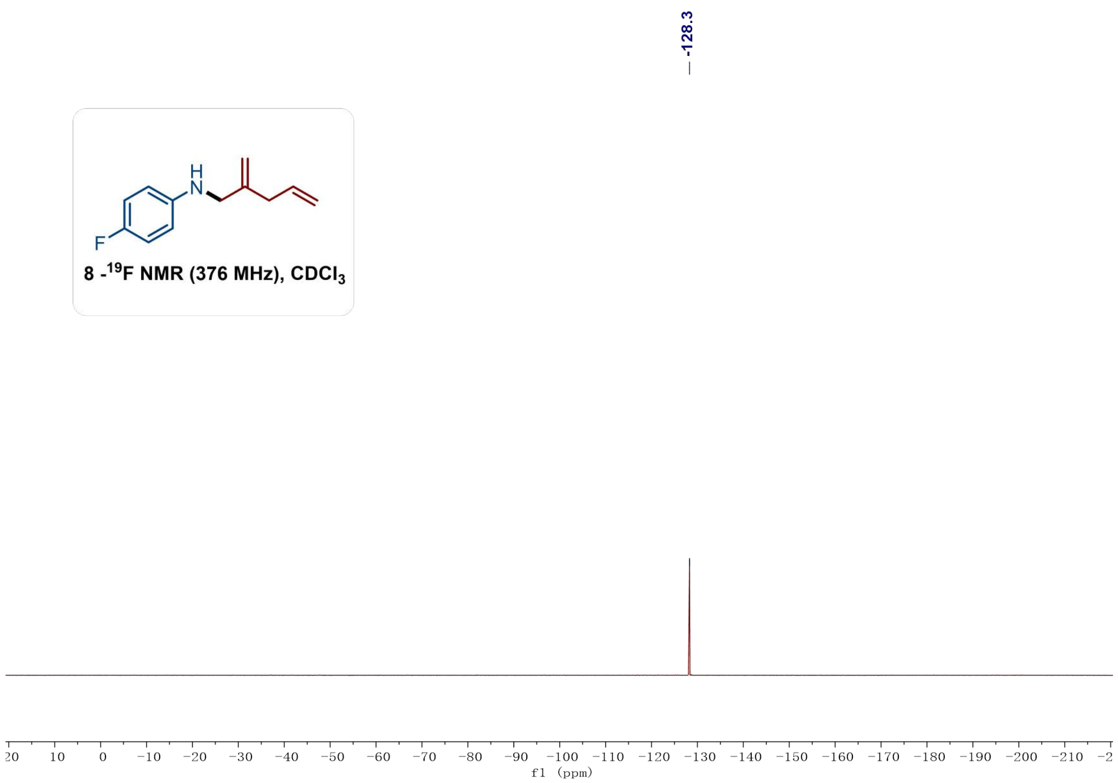

Supplementary Figure 76. ^{19}F NMR (376 MHz, $CDCl_3$) spectrum of compound **8**

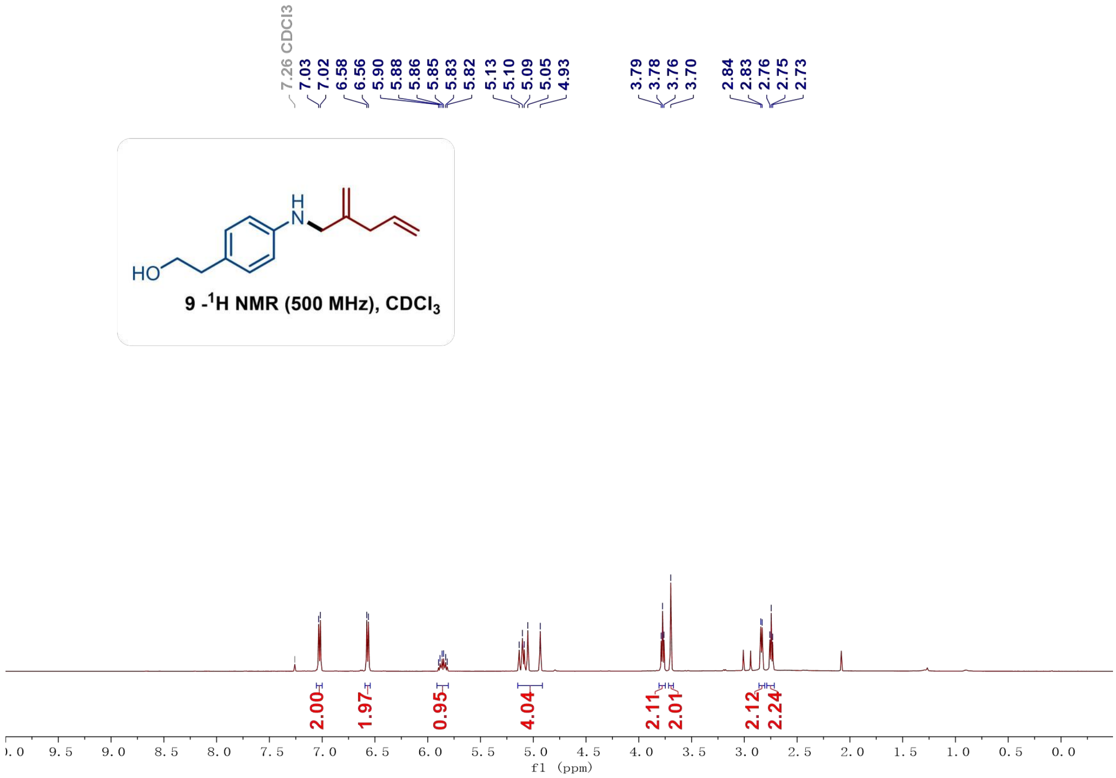

Supplementary Figure 77. ^{1}H NMR (500 MHz, $CDCl_3$) spectrum of compound **9**

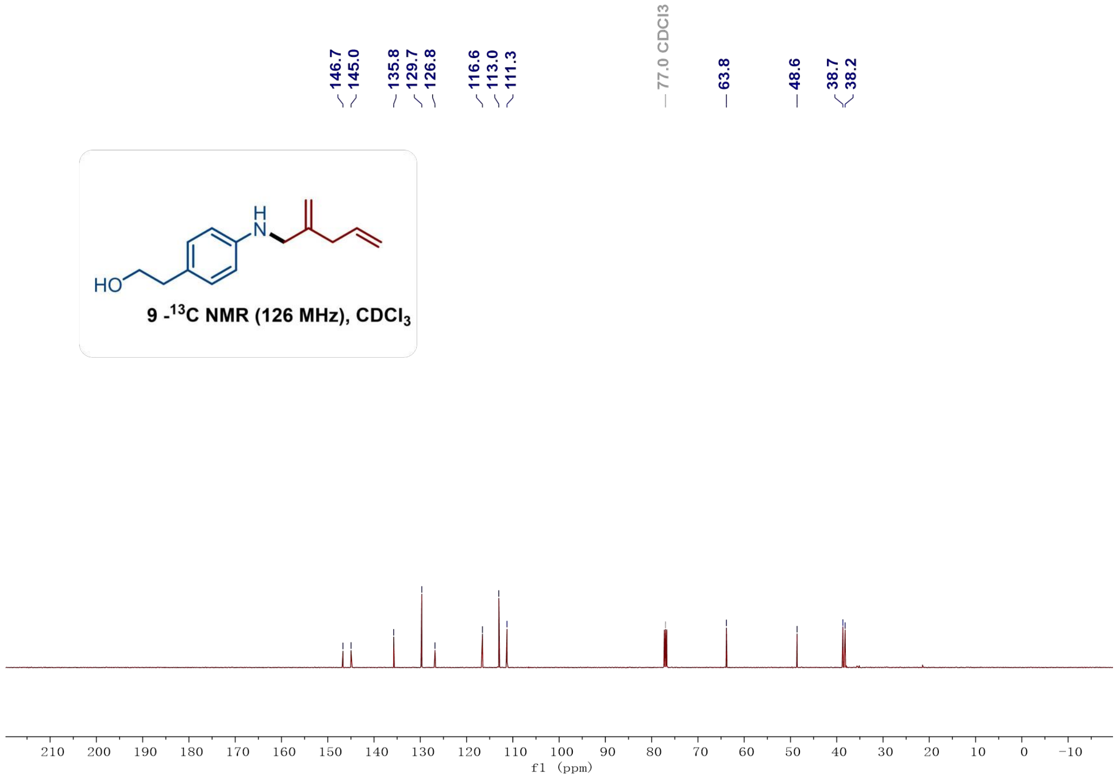

Supplementary Figure 78. ^{13}C NMR (126 MHz, $CDCl_3$) spectrum of compound **9**

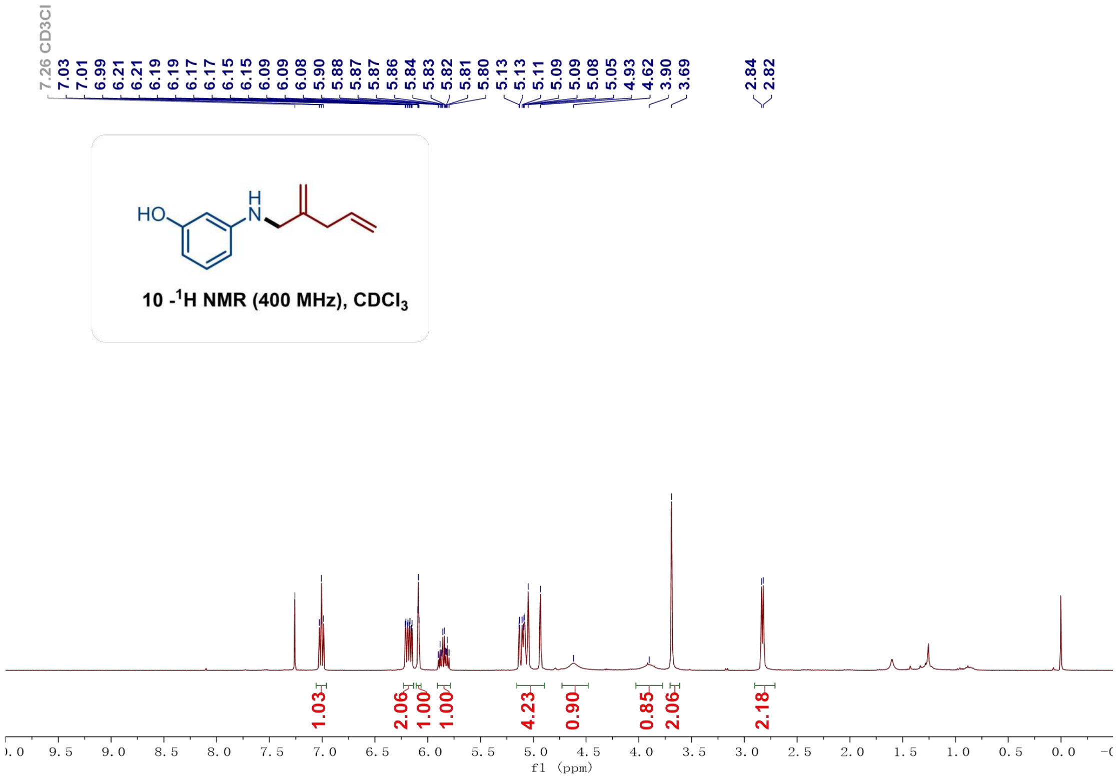

Supplementary Figure 79. ^{1}H NMR (400 MHz, $CDCl_3$) spectrum of compound **10**

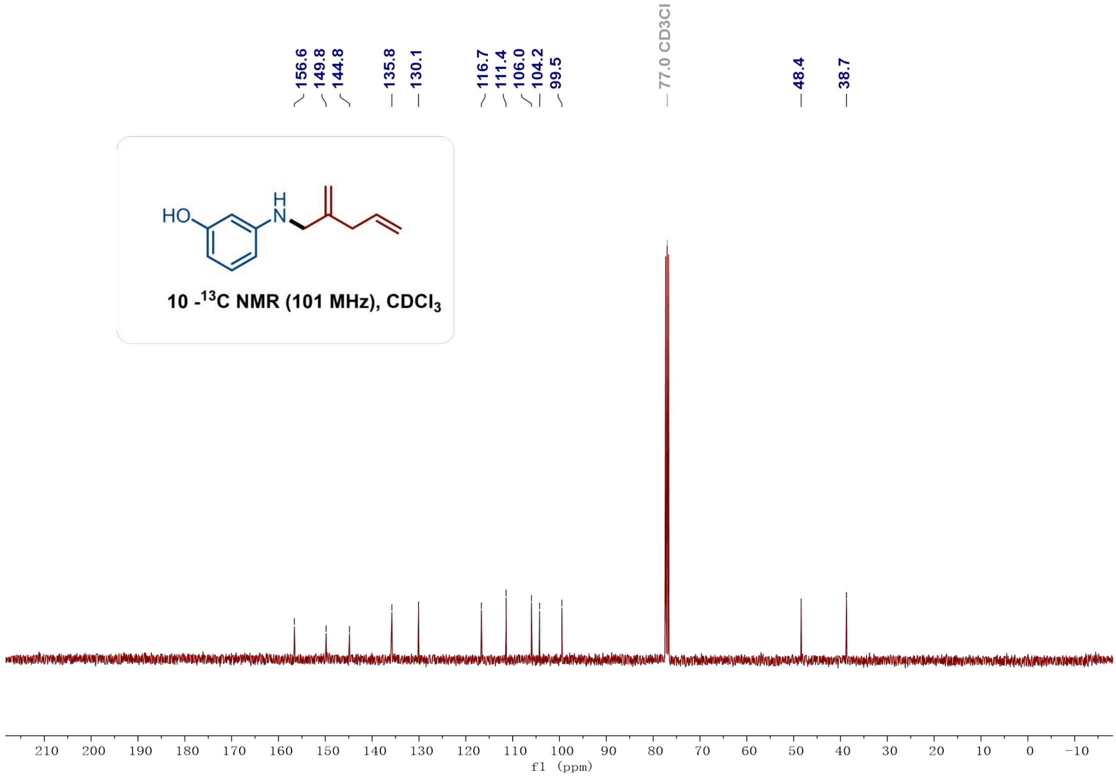

Supplementary Figure 80. ^{13}C NMR (101 MHz, $CDCl_3$) spectrum of compound **10**

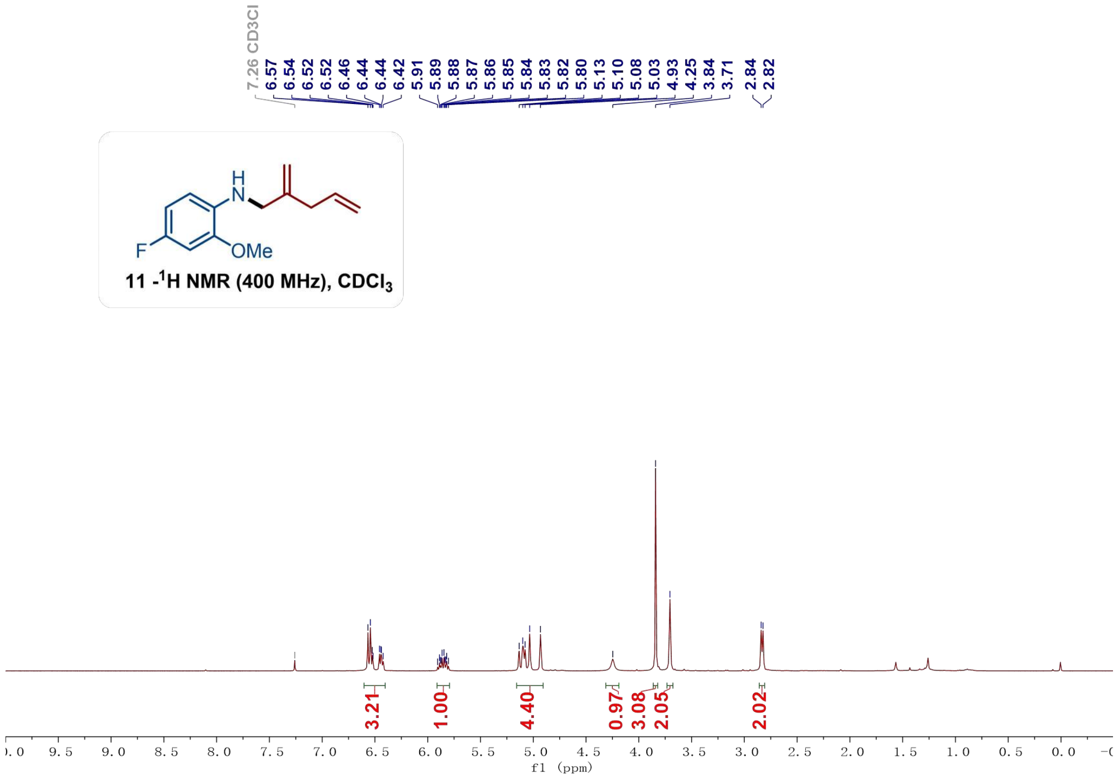

Supplementary Figure 81. ^{1}H NMR (400 MHz, $CDCl_3$) spectrum of compound **11**

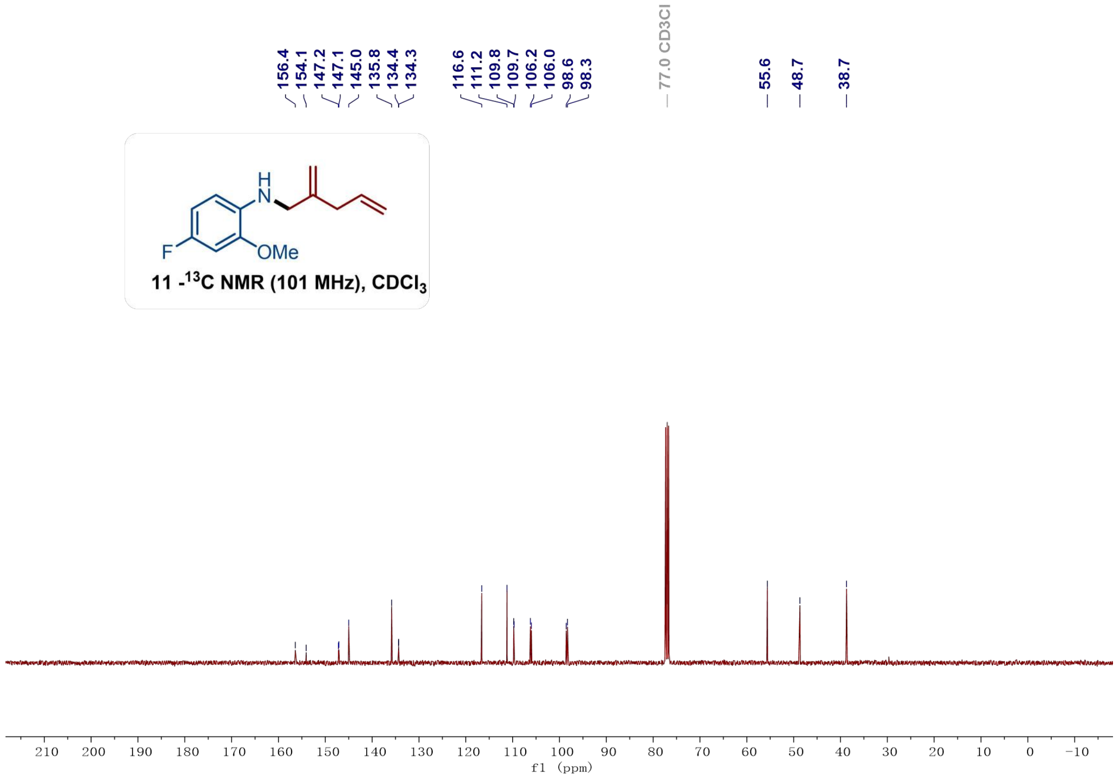

Supplementary Figure 82. ^{13}C NMR (101 MHz, $CDCl_3$) spectrum of compound **11**

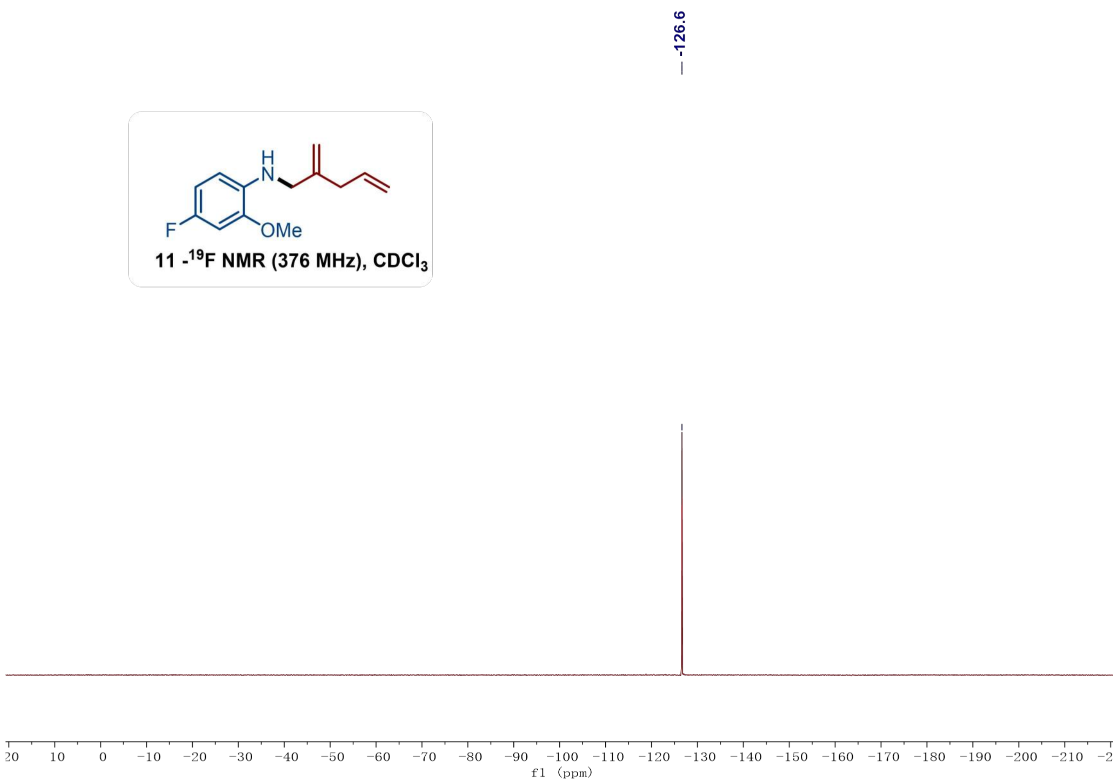

Supplementary Figure 83. ^{19}F NMR (376 MHz, $CDCl_3$) spectrum of compound **11**

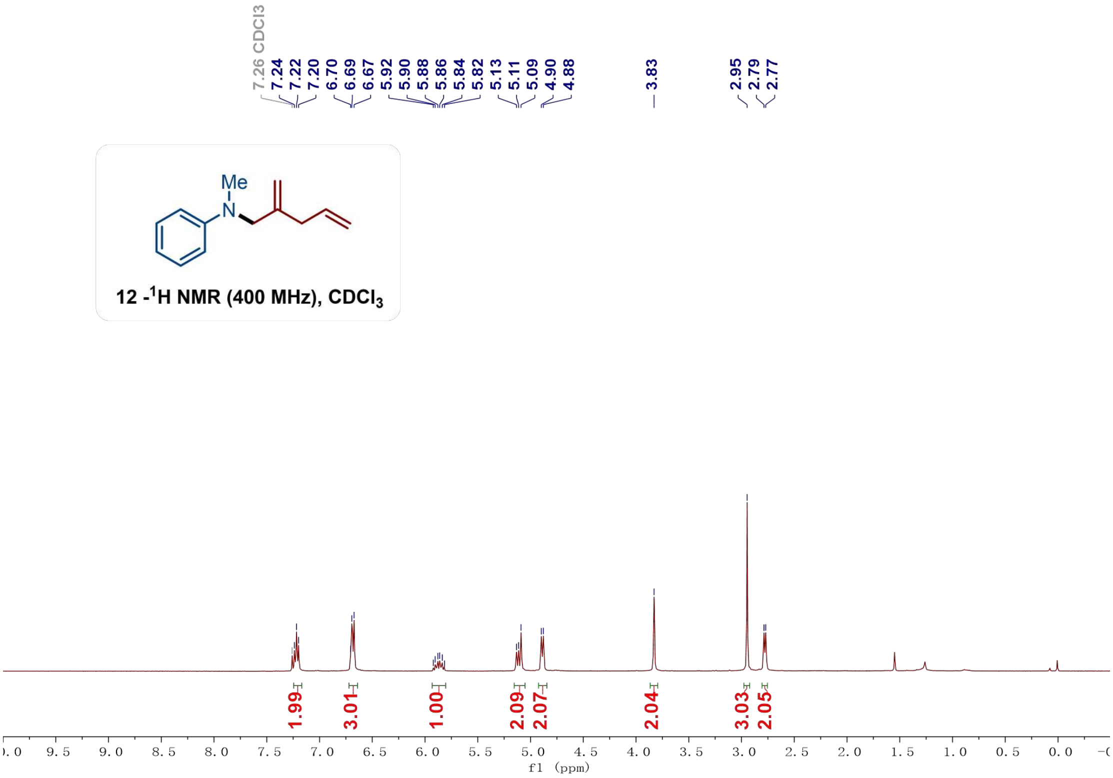

Supplementary Figure 84. ^{1}H NMR (400 MHz, $CDCl_3$) spectrum of compound **12**

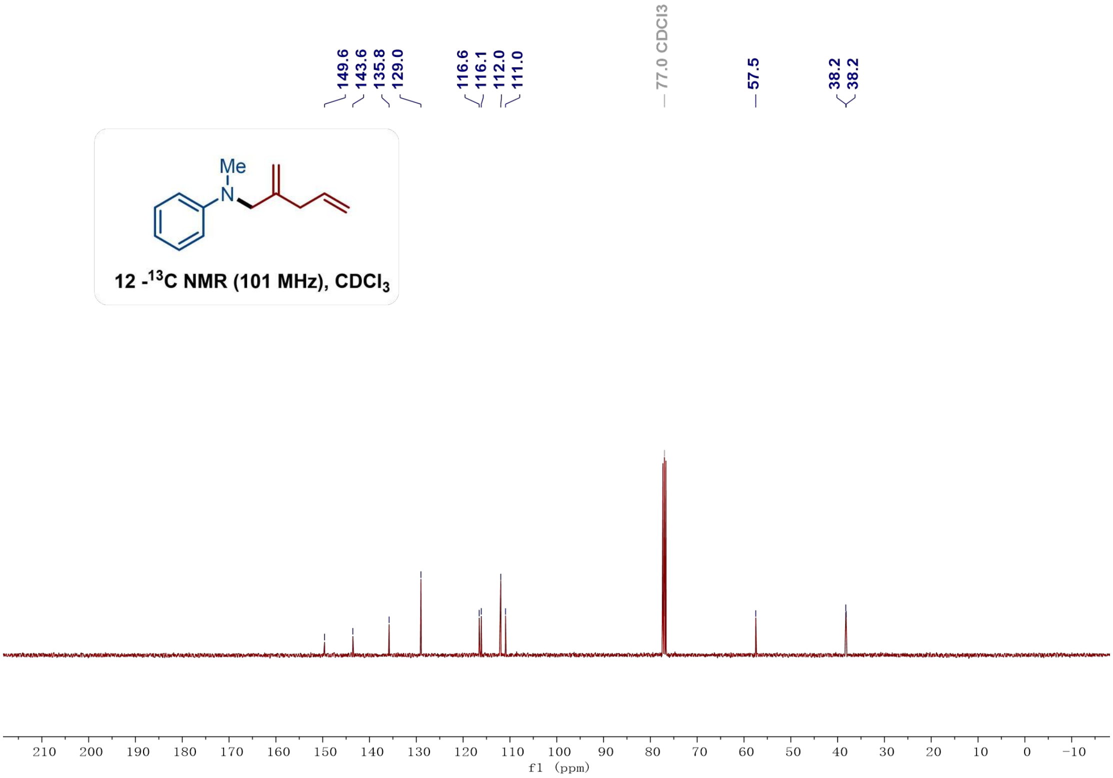

Supplementary Figure 85. ^{13}C NMR (101 MHz, $CDCl_3$) spectrum of compound **12**

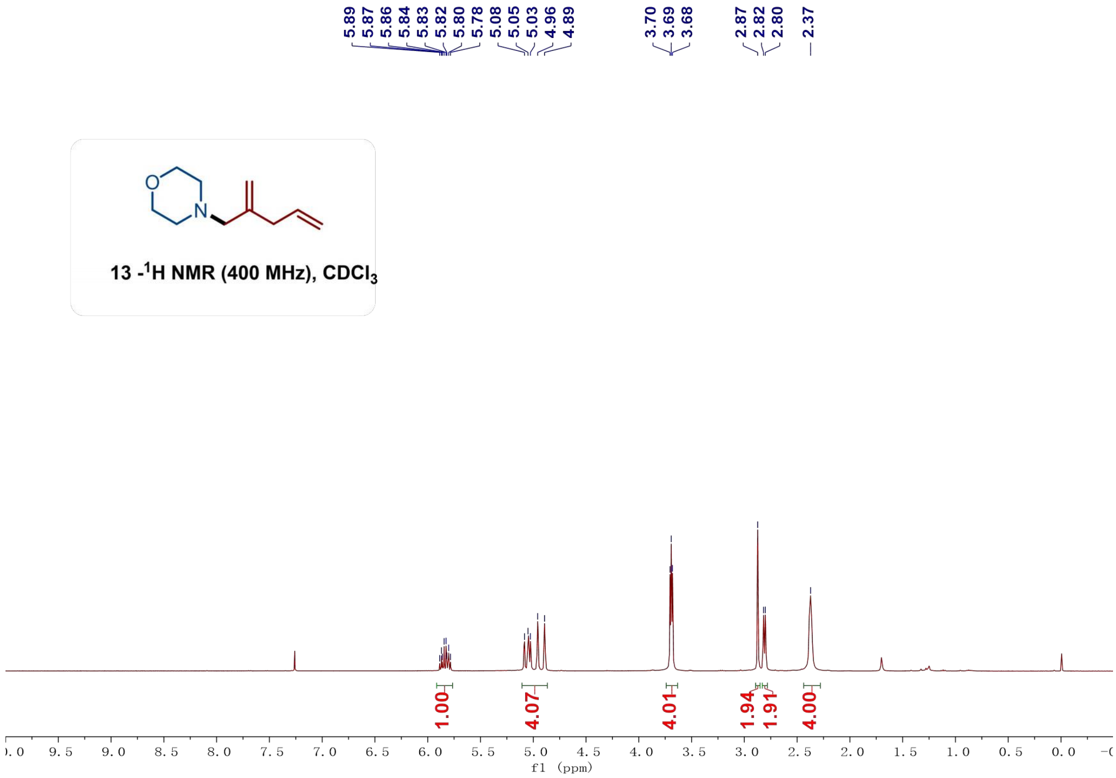

Supplementary Figure 86. ^{1}H NMR (400 MHz, $CDCl_3$) spectrum of compound **13**

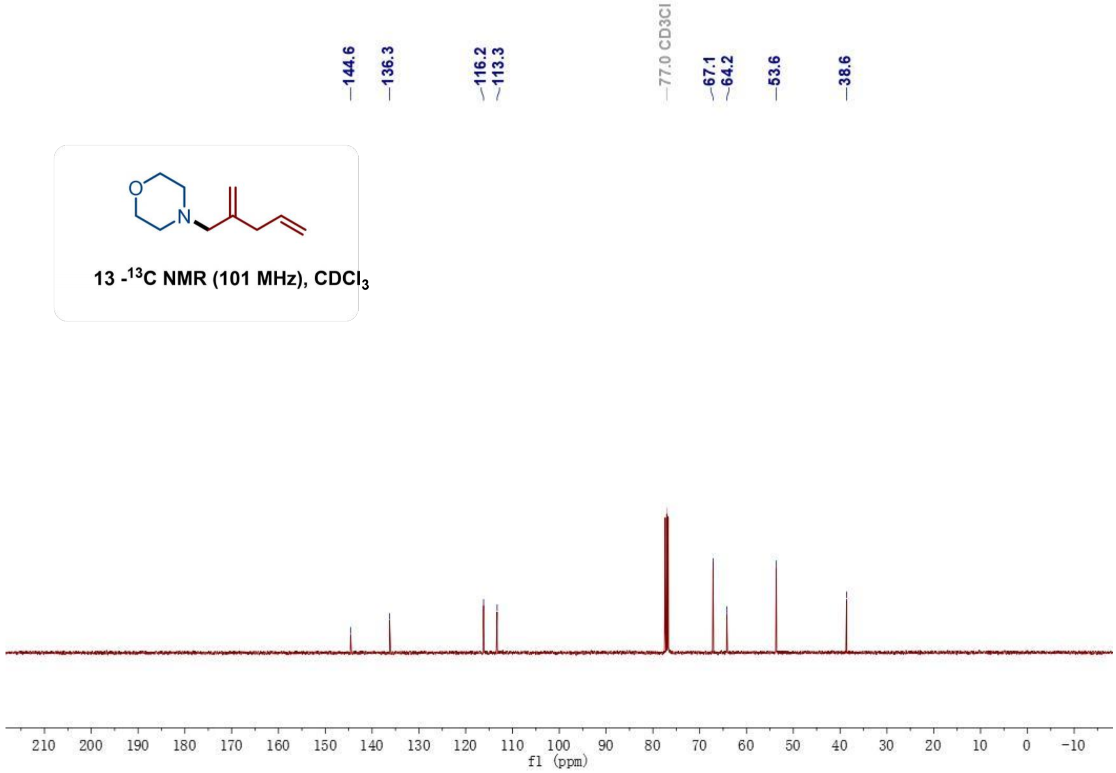

Supplementary Figure 87. ^{13}C NMR (101 MHz, $CDCl_3$) spectrum of compound **13**

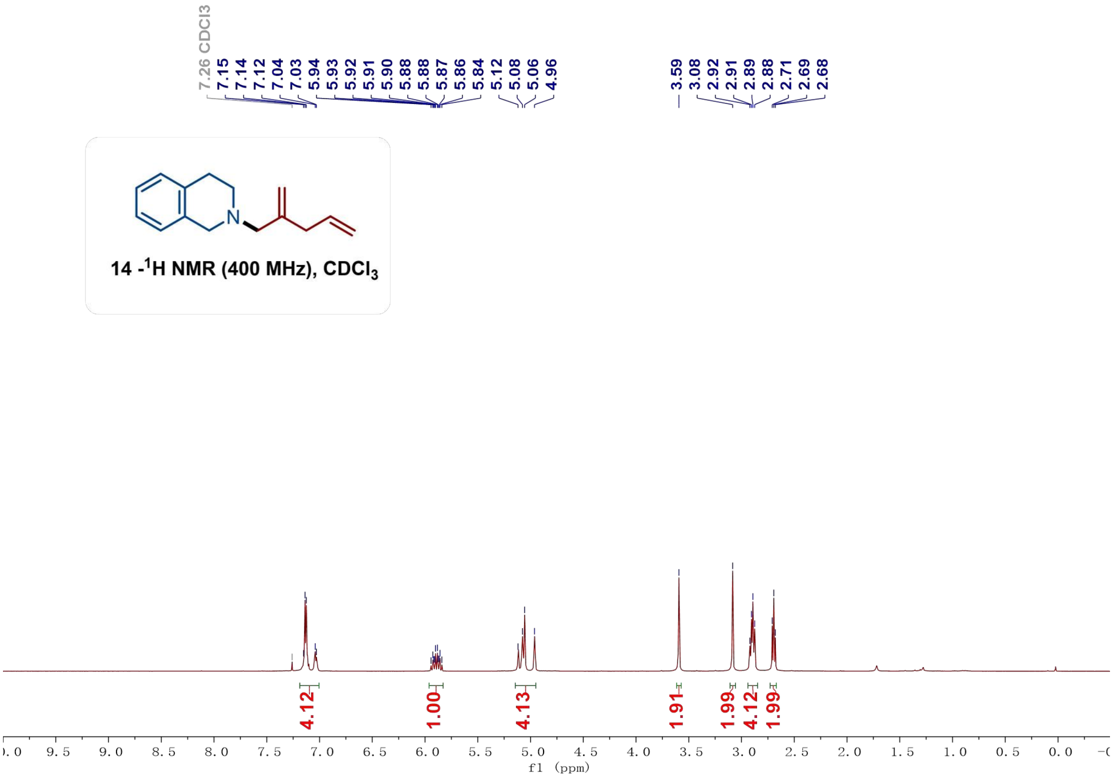

Supplementary Figure 88. ^{1}H NMR (400 MHz, $CDCl_3$) spectrum of compound **14**

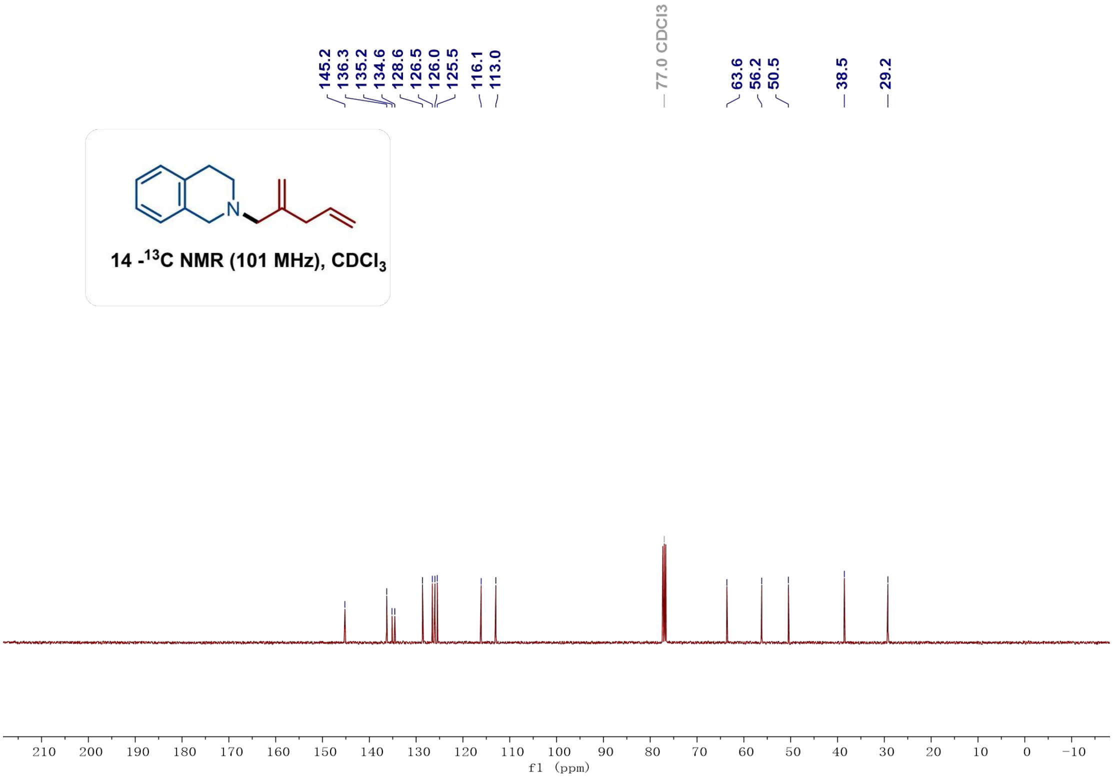

Supplementary Figure 89. ^{13}C NMR (101 MHz, $CDCl_3$) spectrum of compound **14**

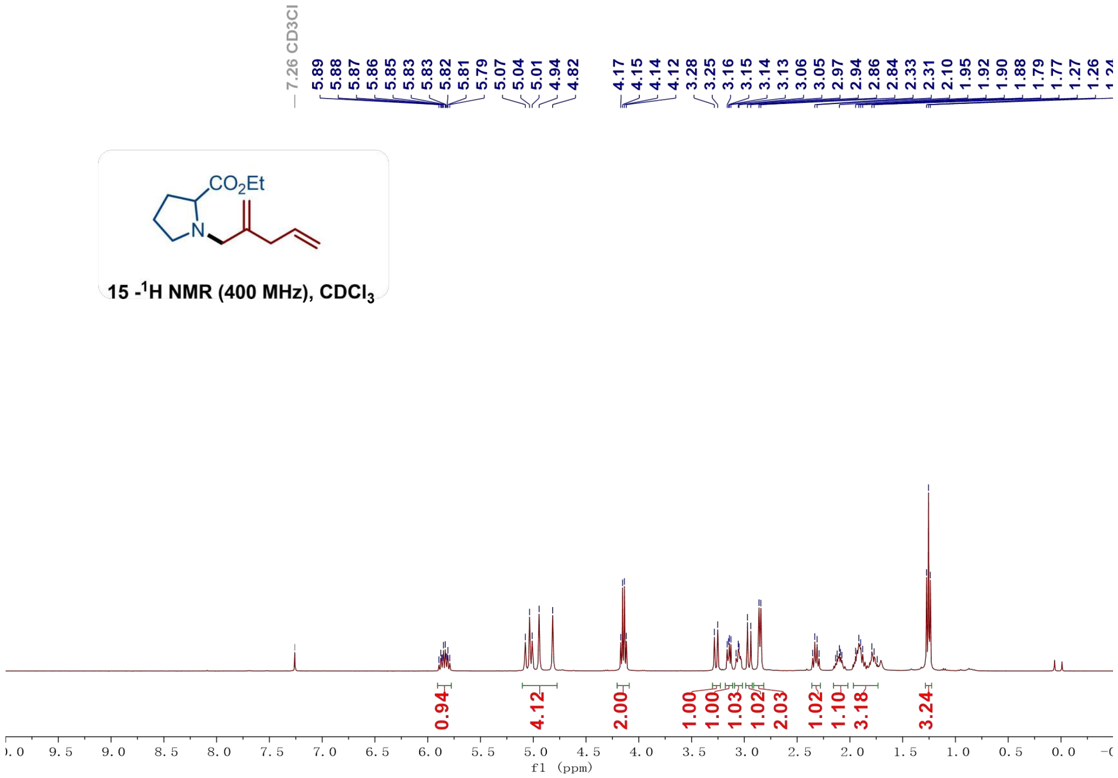

Supplementary Figure 90. ^{1}H NMR (400 MHz, $CDCl_3$) spectrum of compound **15**

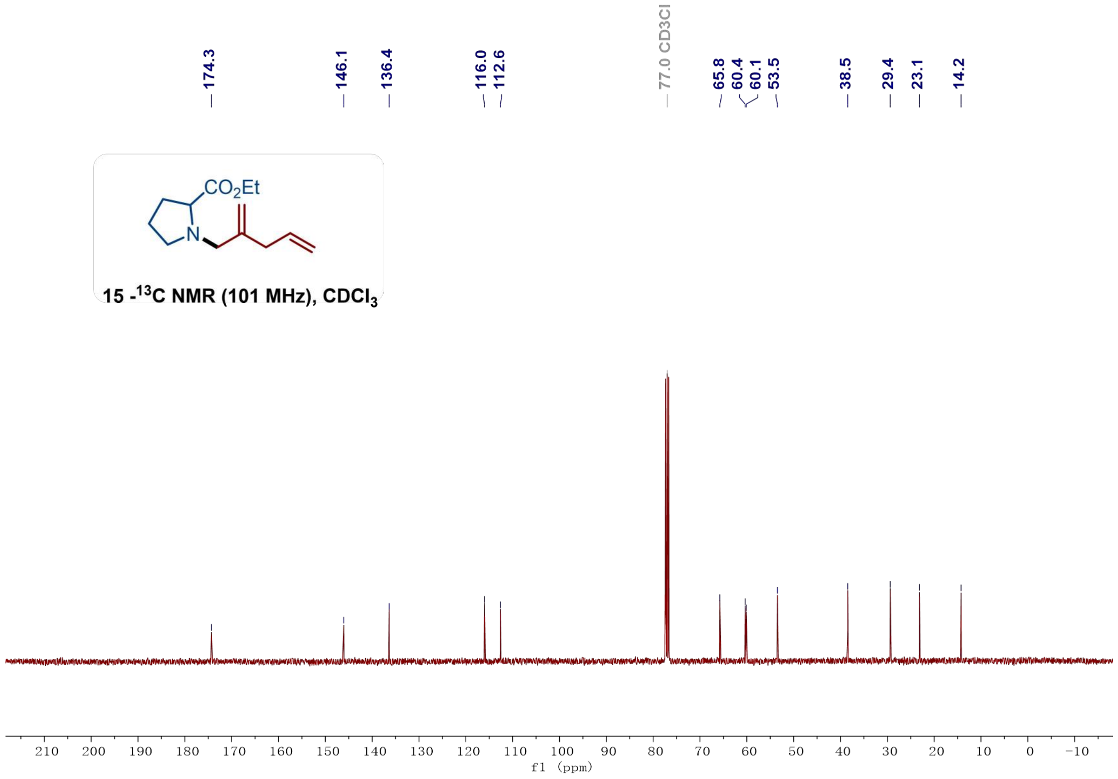

Supplementary Figure 91. ^{13}C NMR (101 MHz, $CDCl_3$) spectrum of compound **15**

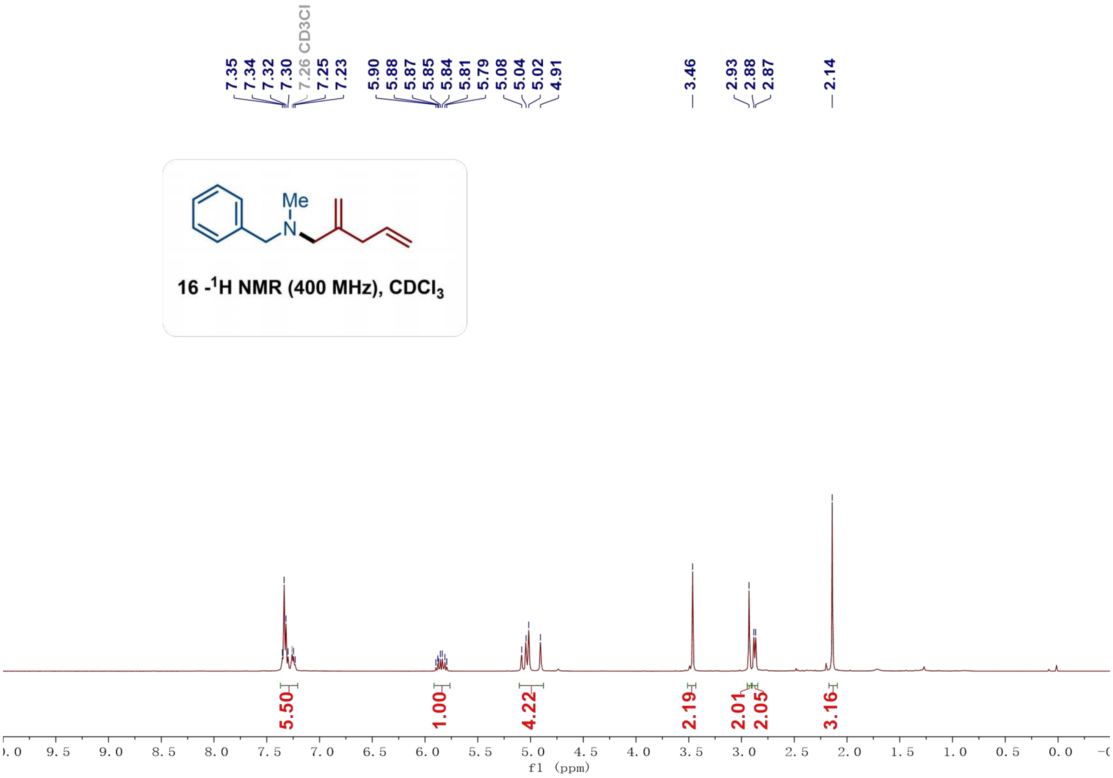

Supplementary Figure 92. ^{1}H NMR (400 MHz, $CDCl_3$) spectrum of compound **16**

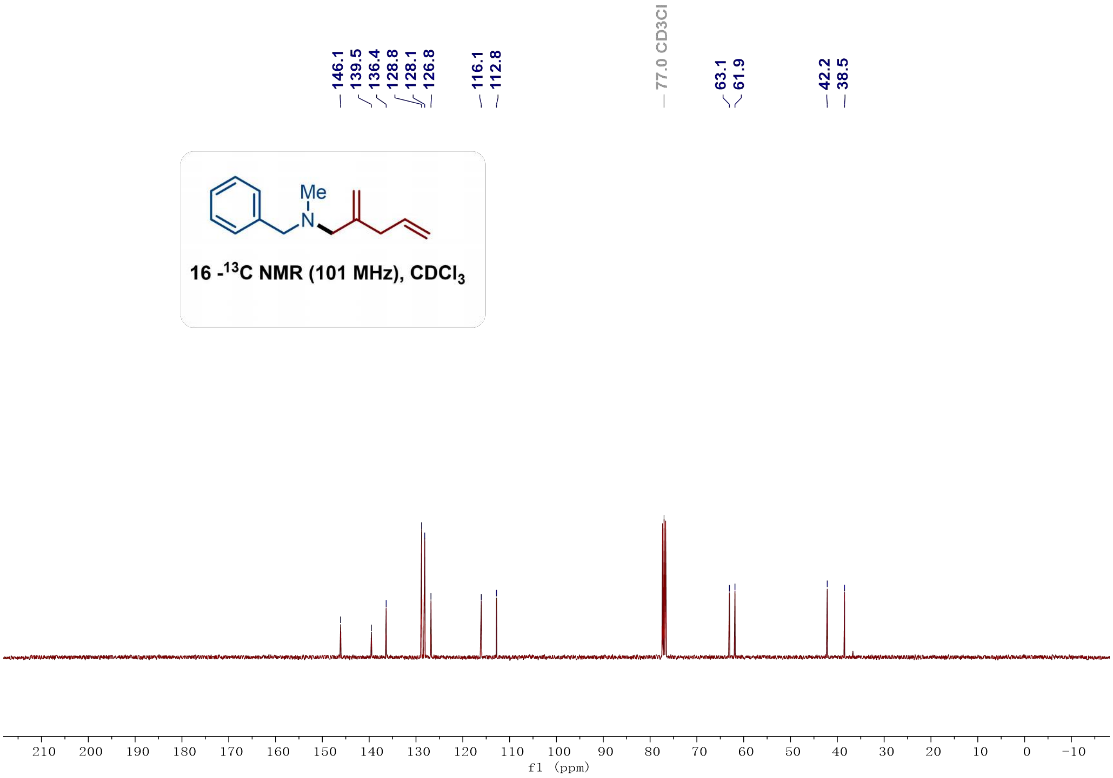

Supplementary Figure 93. ^{13}C NMR (101 MHz, $CDCl_3$) spectrum of compound **16**

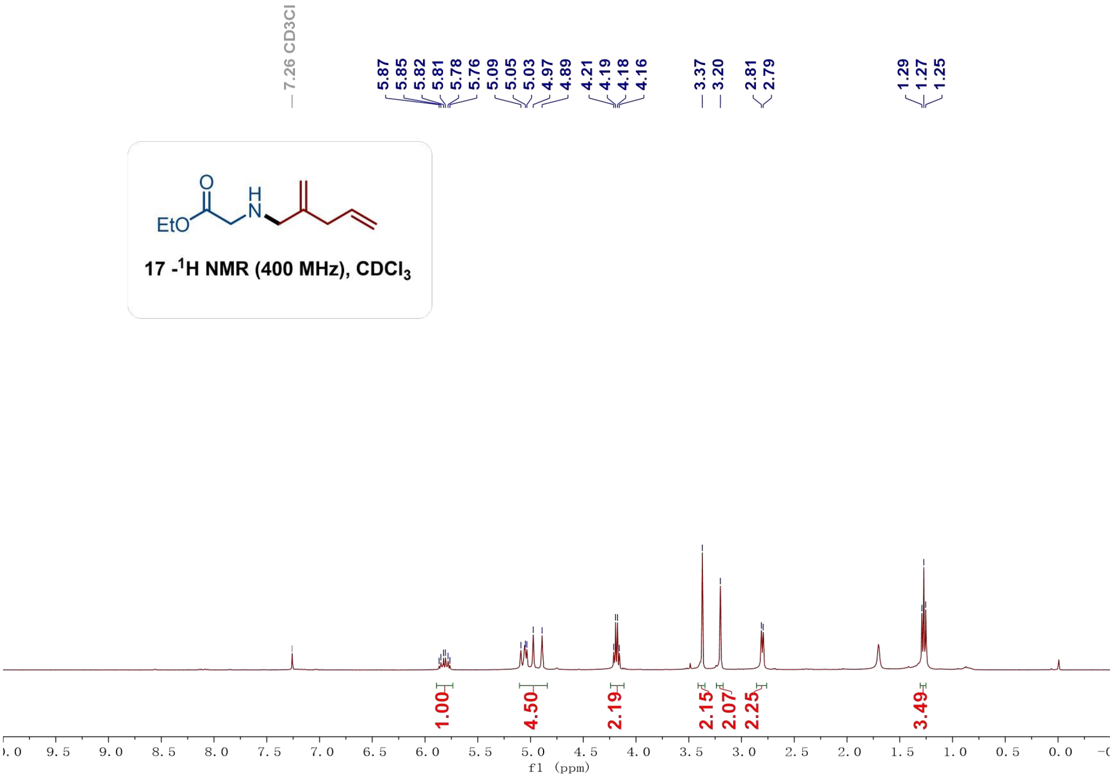

Supplementary Figure 94. ^{1}H NMR (400 MHz, $CDCl_3$) spectrum of compound **17**

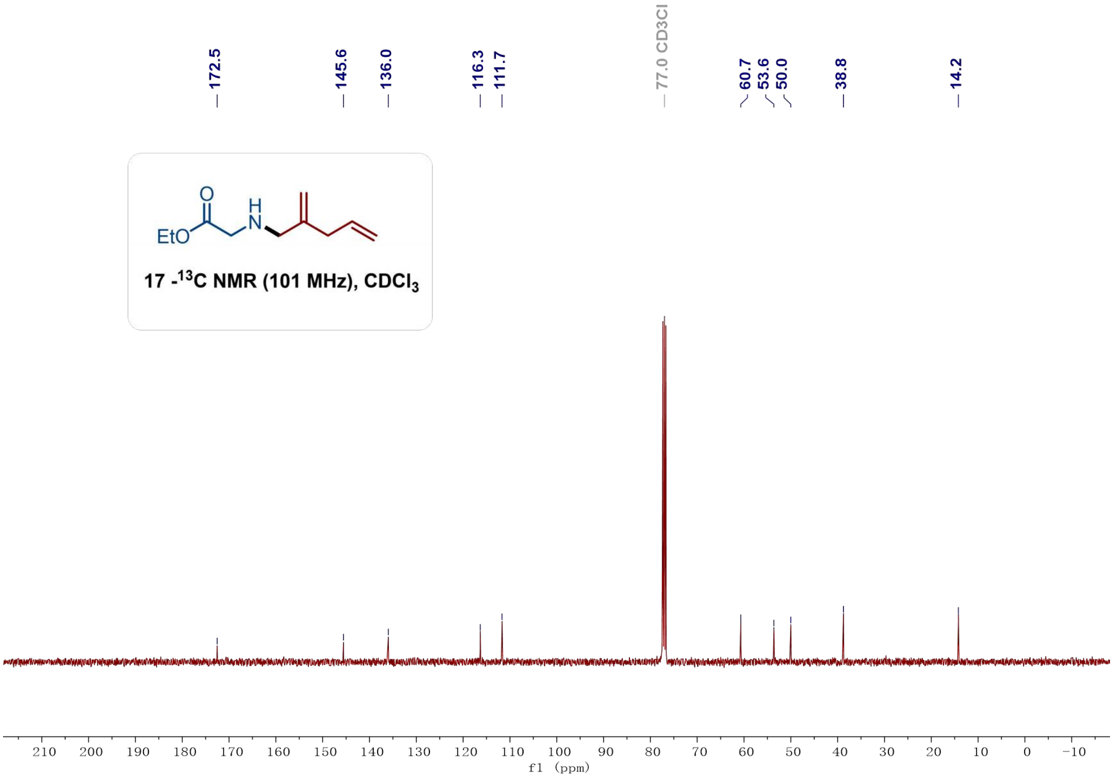

Supplementary Figure 95. ^{13}C NMR (101 MHz, $CDCl_3$) spectrum of compound **17**

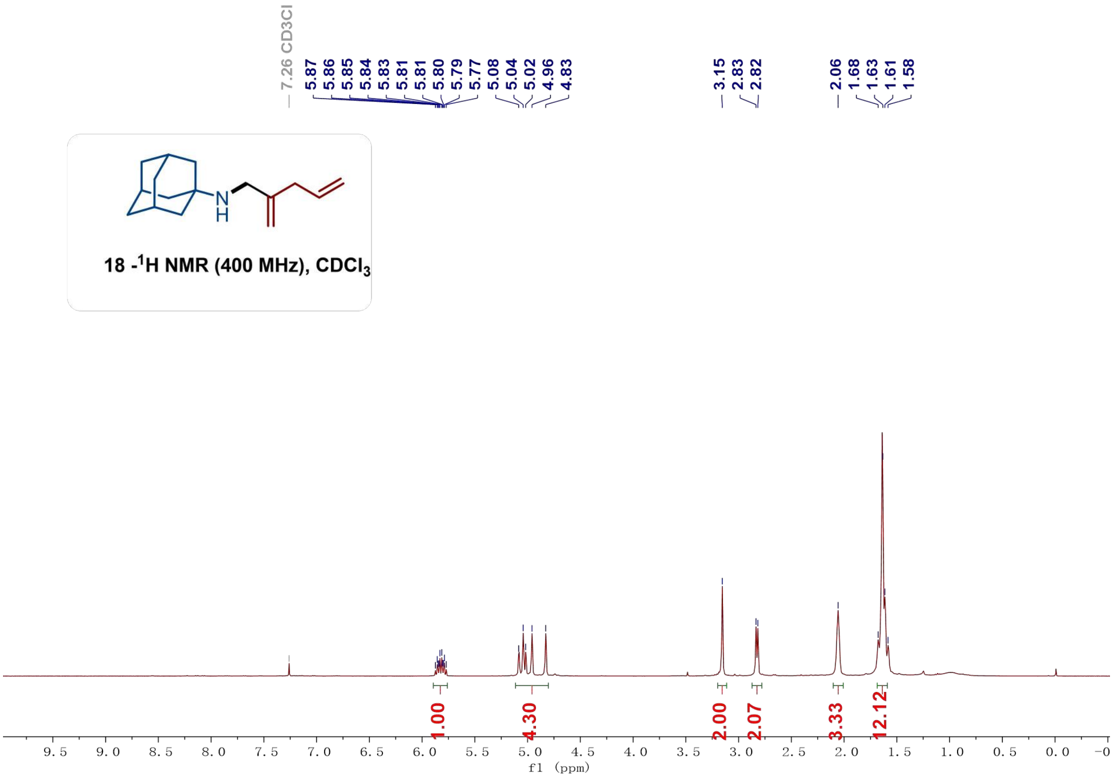

Supplementary Figure 96. ^{1}H NMR (400 MHz, $CDCl_3$) spectrum of compound **18**

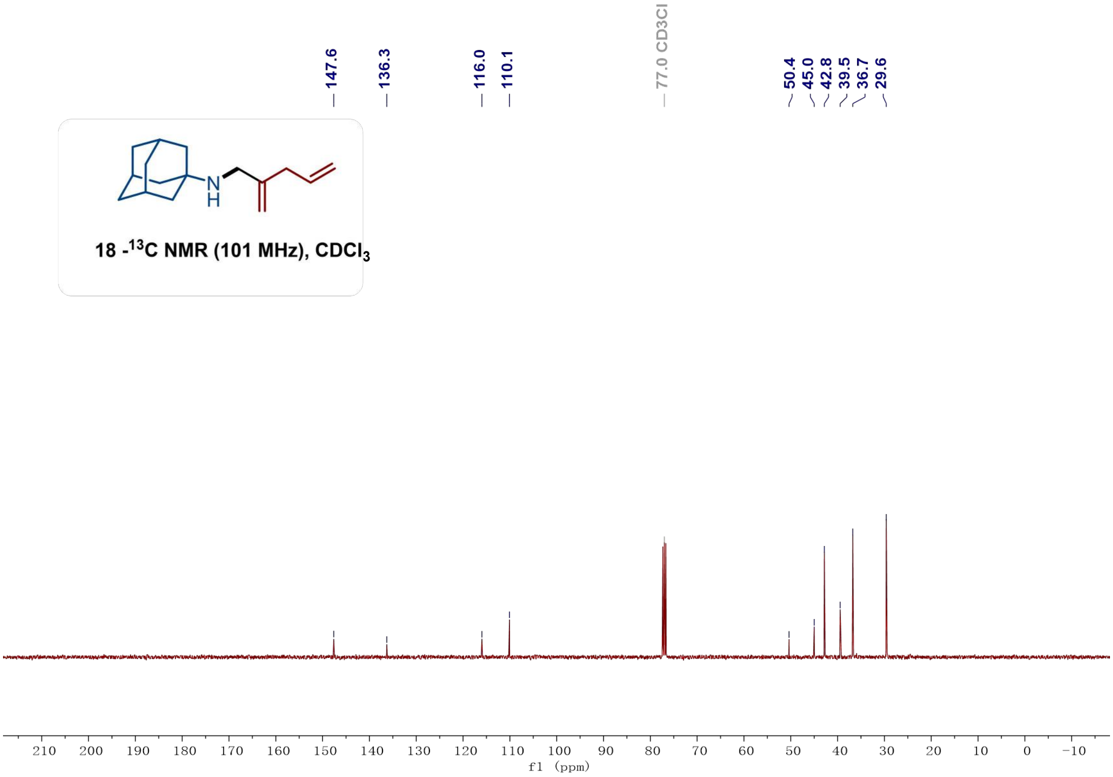

Supplementary Figure 97. ^{13}C NMR (101 MHz, $CDCl_3$) spectrum of compound **18**

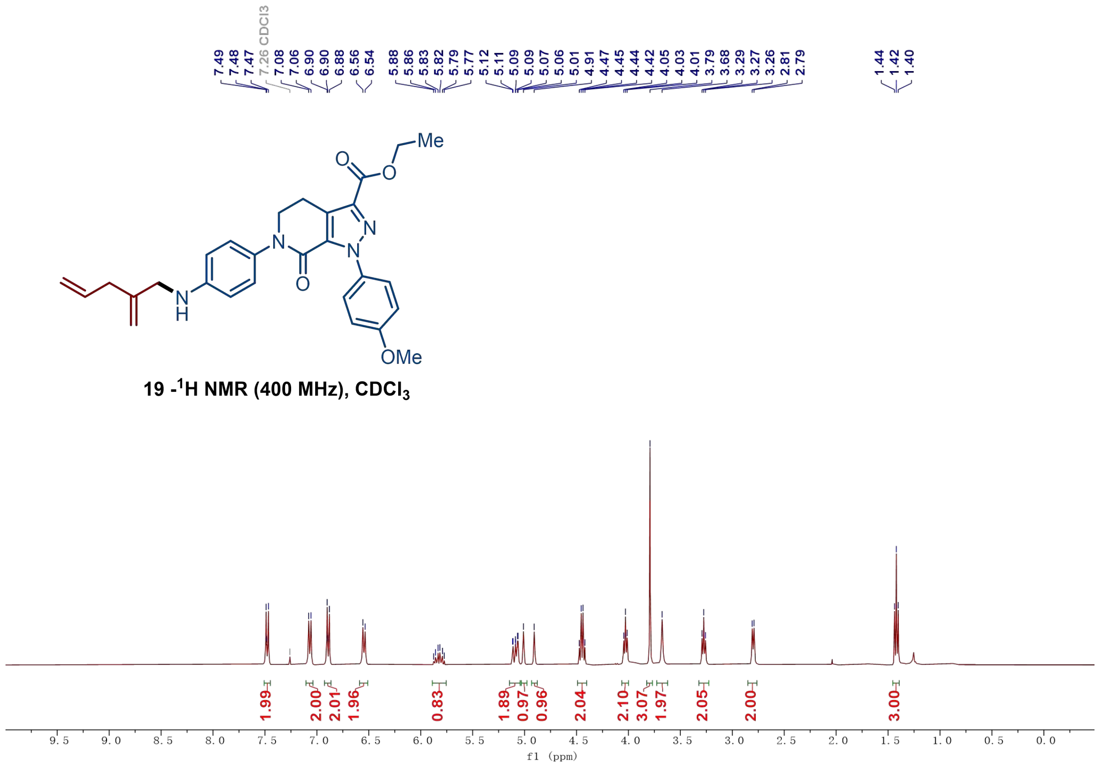

Supplementary Figure 98. ^{1}H NMR (400 MHz, $CDCl_3$) spectrum of compound **19**

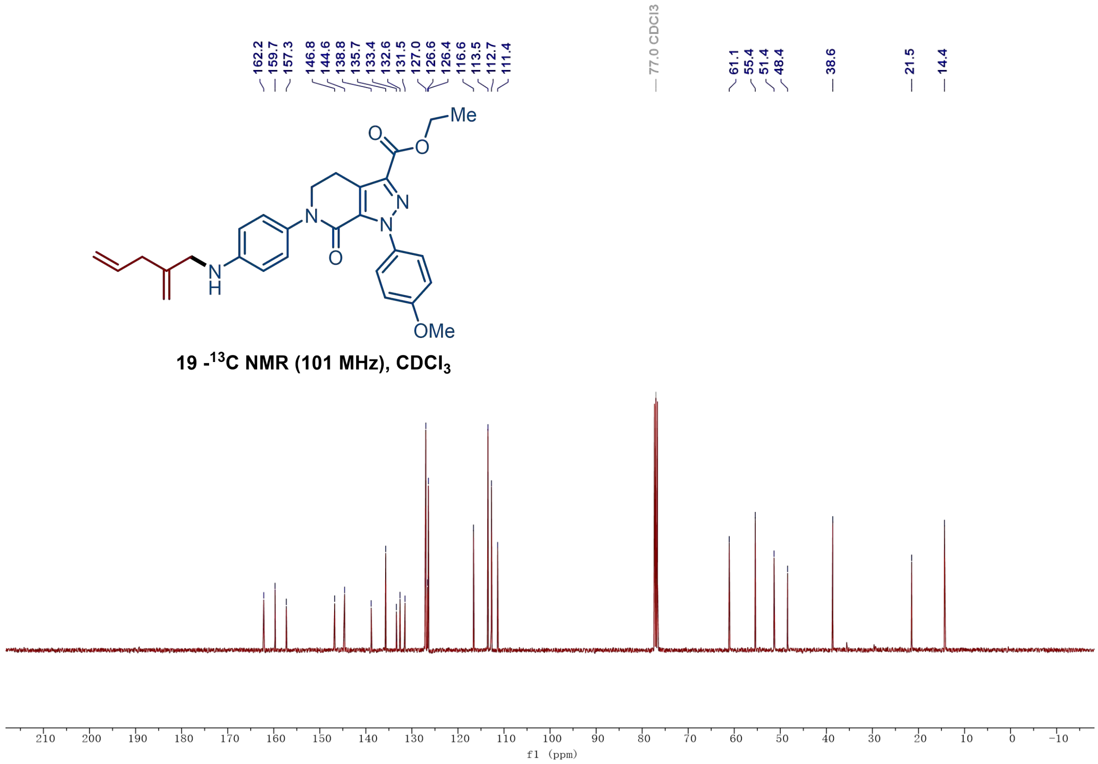

Supplementary Figure 99. ^{13}C NMR (101 MHz, $CDCl_3$) spectrum of compound **19**

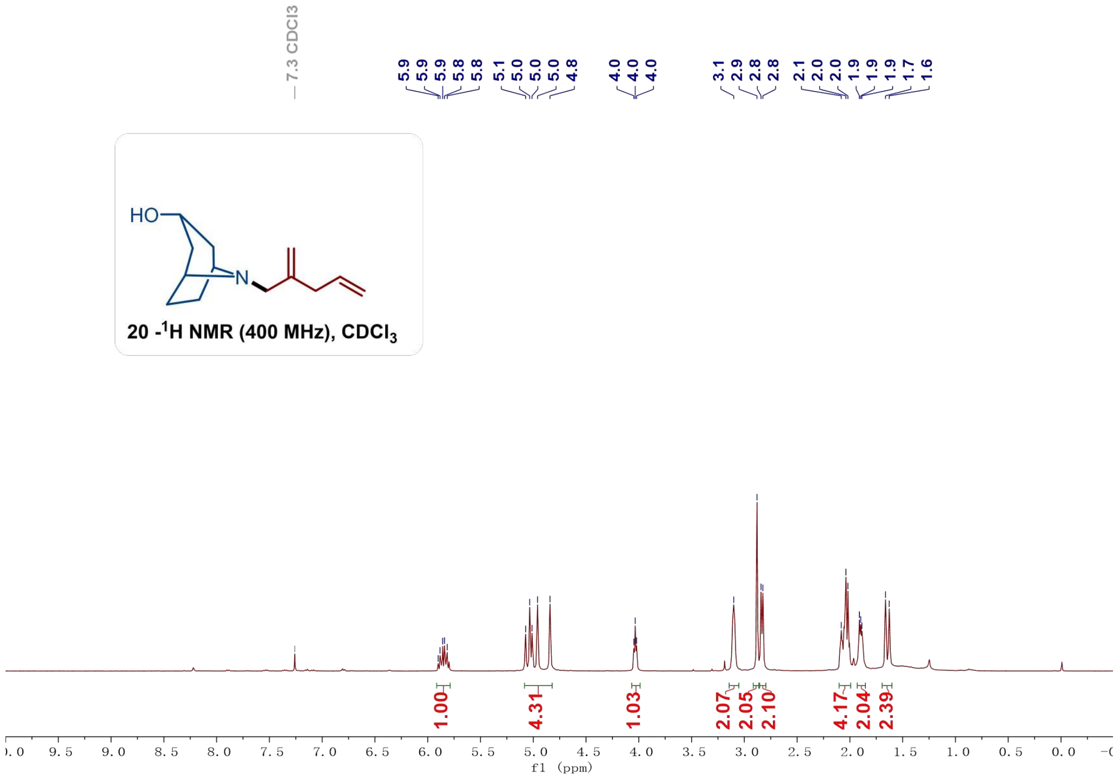

Supplementary Figure 100. ^{1}H NMR (400 MHz, $CDCl_3$) spectrum of compound **20**

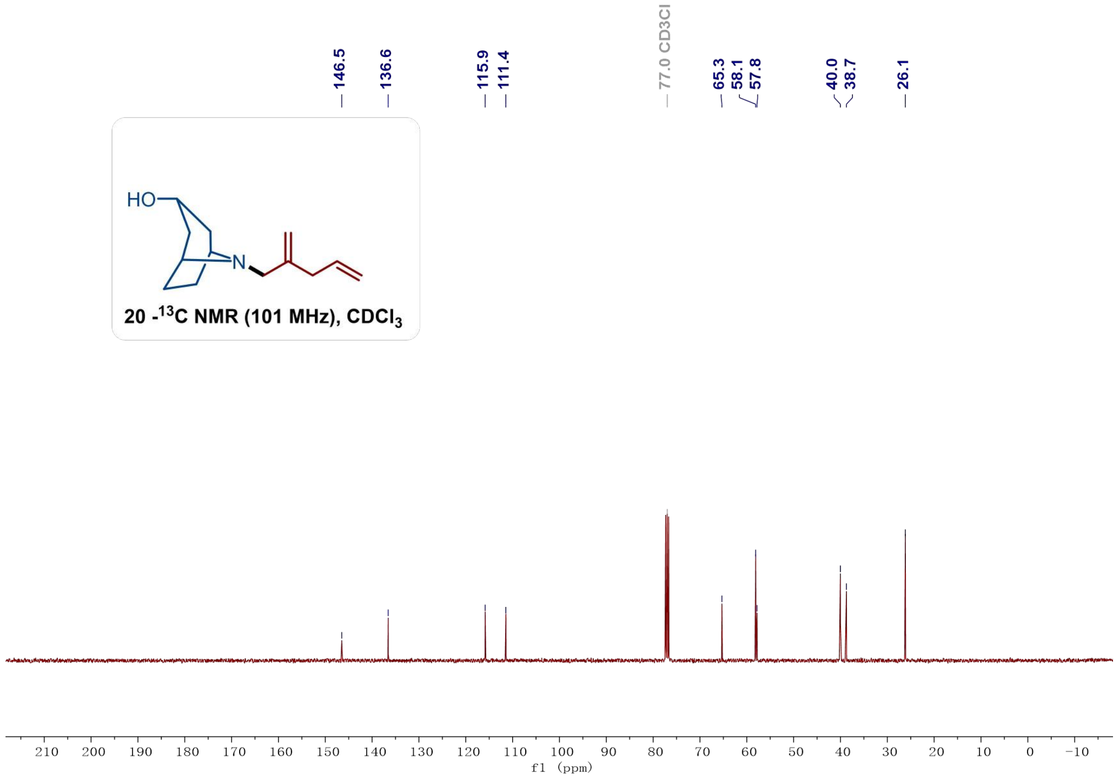

Supplementary Figure 101. ^{13}C NMR (101 MHz, $CDCl_3$) spectrum of compound **20**

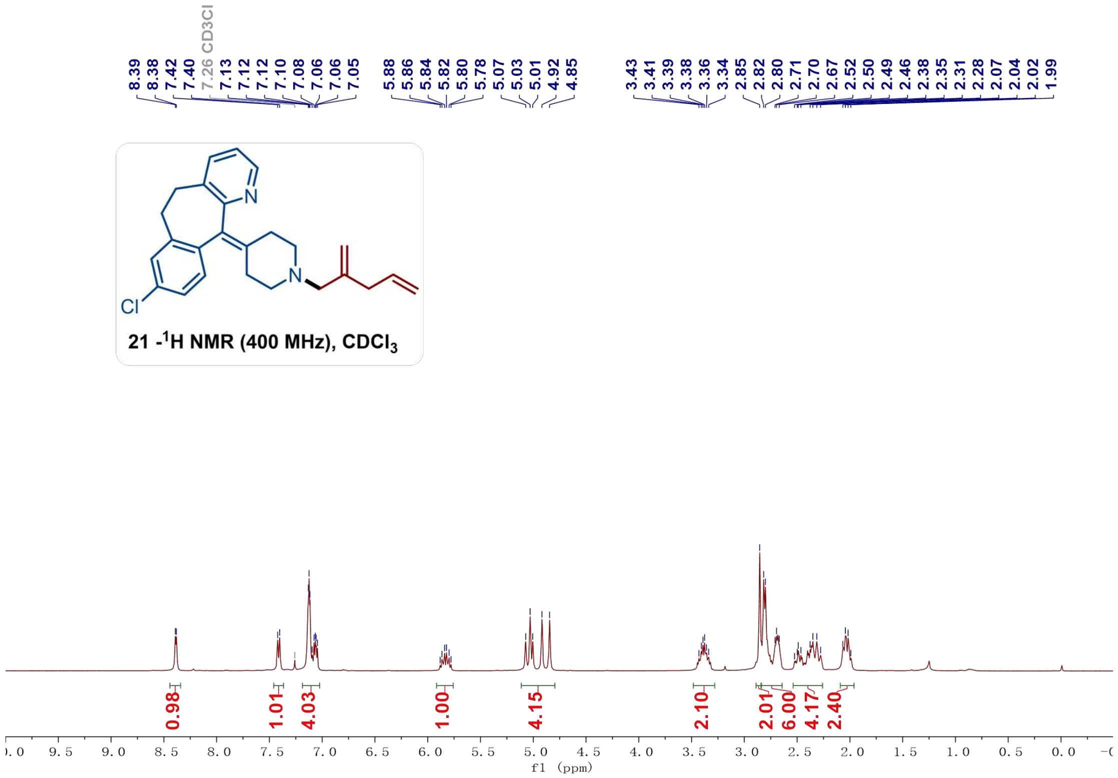

Supplementary Figure 102. ^{1}H NMR (400 MHz, $CDCl_3$) spectrum of compound **21**

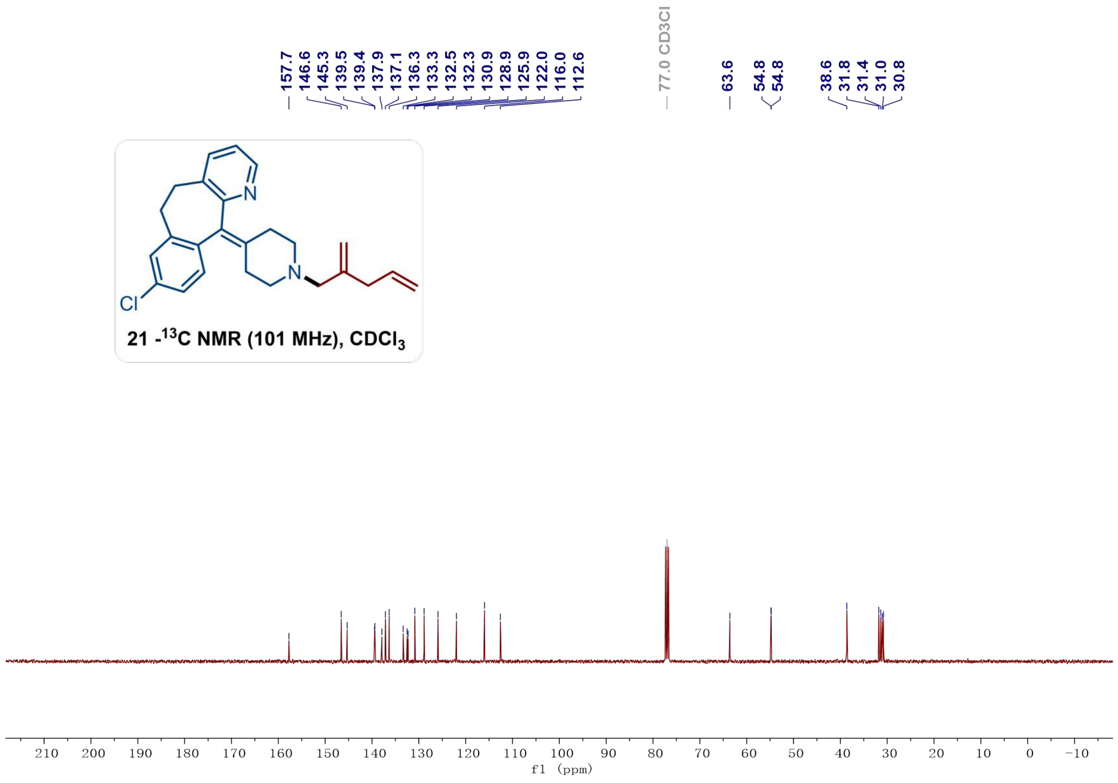

Supplementary Figure 103. ^{13}C NMR (101 MHz, $CDCl_3$) spectrum of compound **21**

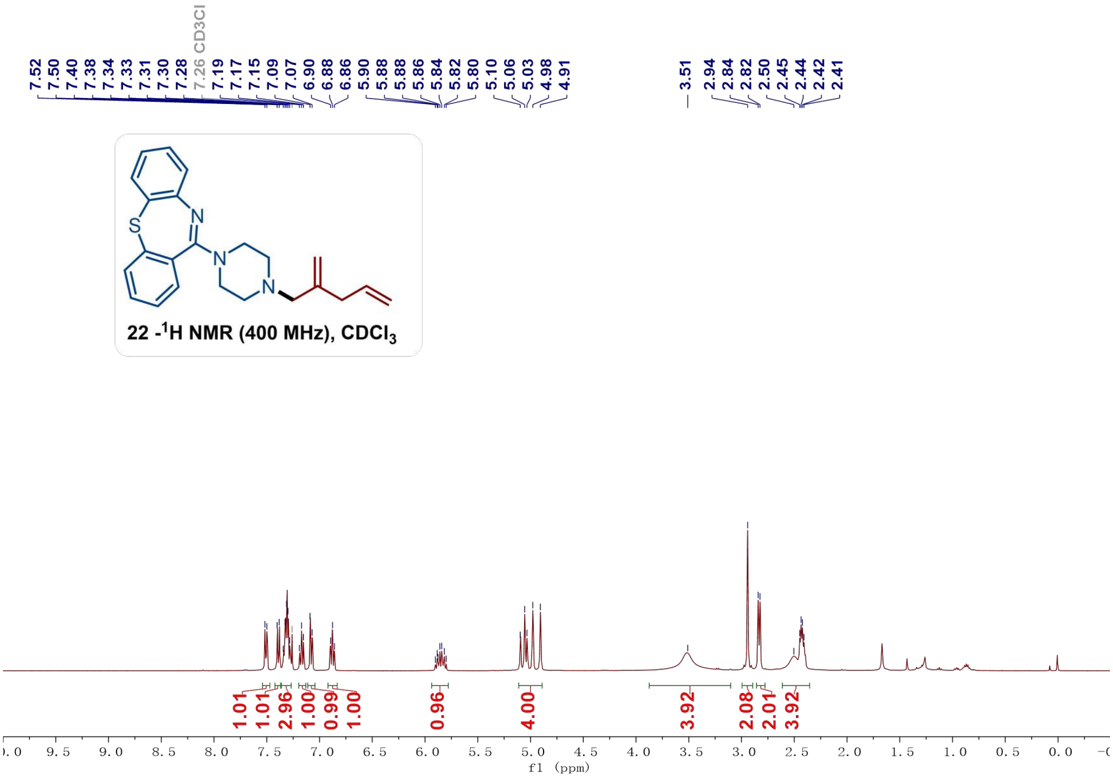

Supplementary Figure 104. ^{1}H NMR (400 MHz, $CDCl_3$) spectrum of compound **22**

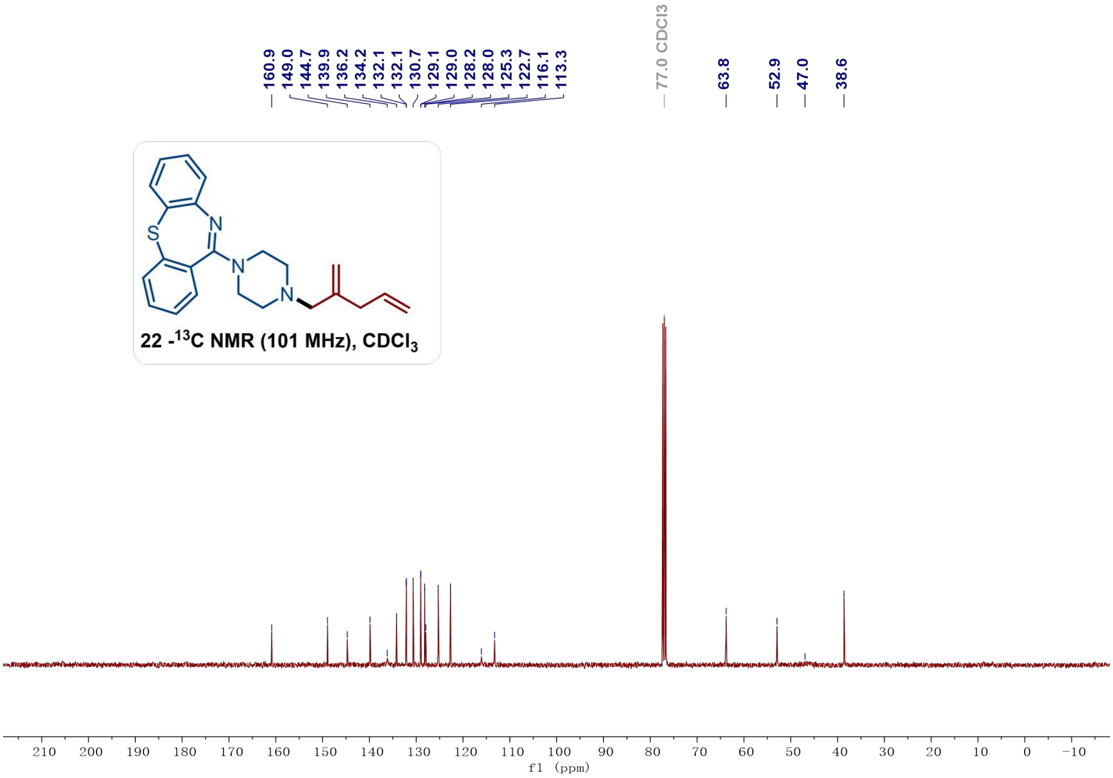

Supplementary Figure 105. ^{13}C NMR (101 MHz, $CDCl_3$) spectrum of compound **22**

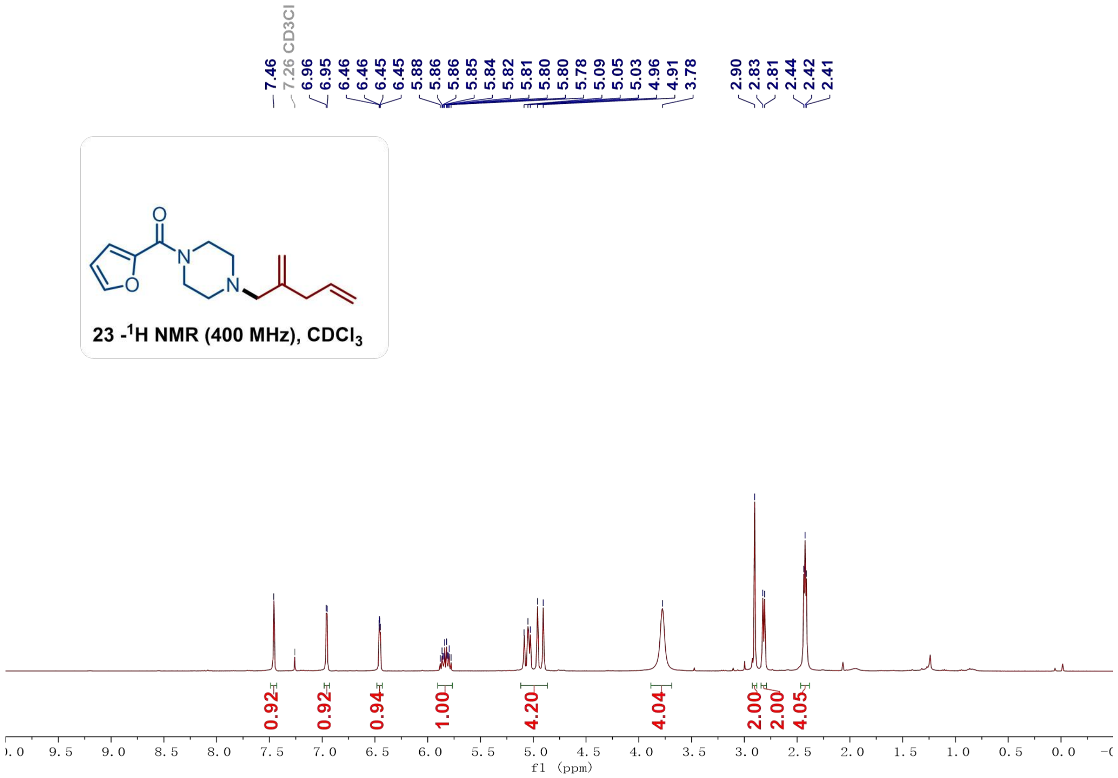

Supplementary Figure 106. ^{1}H NMR (400 MHz, $CDCl_3$) spectrum of compound **23**

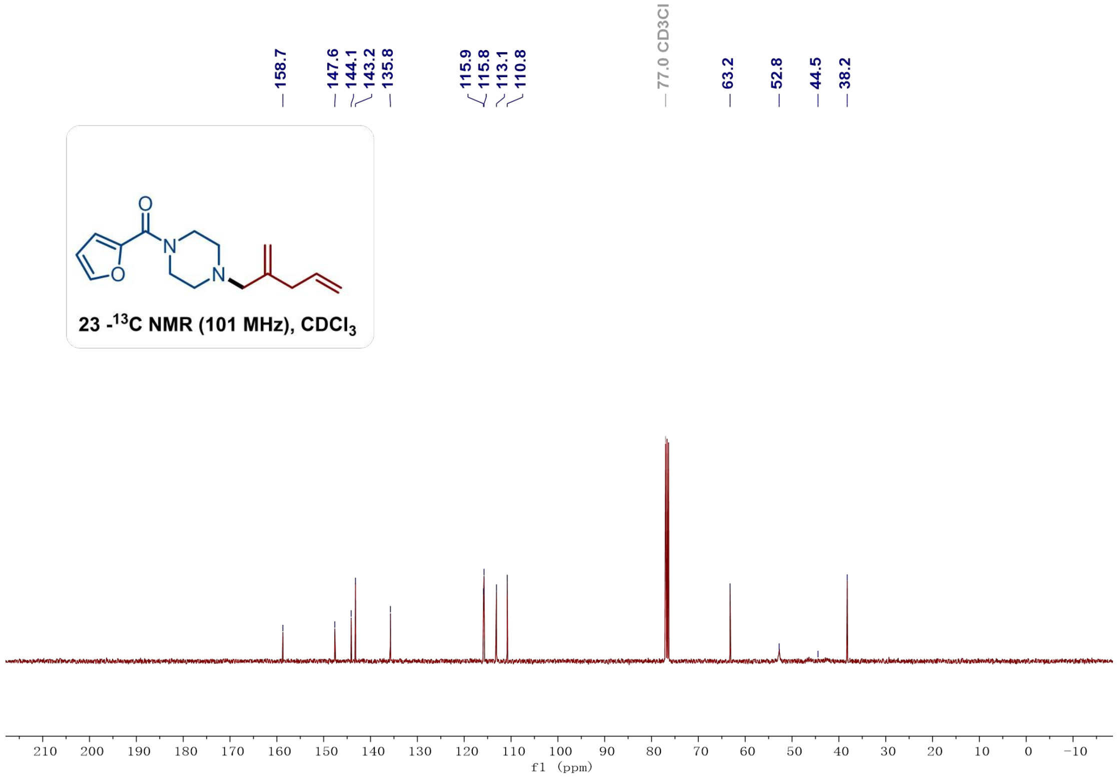

Supplementary Figure 107. ^{13}C NMR (101 MHz, $CDCl_3$) spectrum of compound **23**

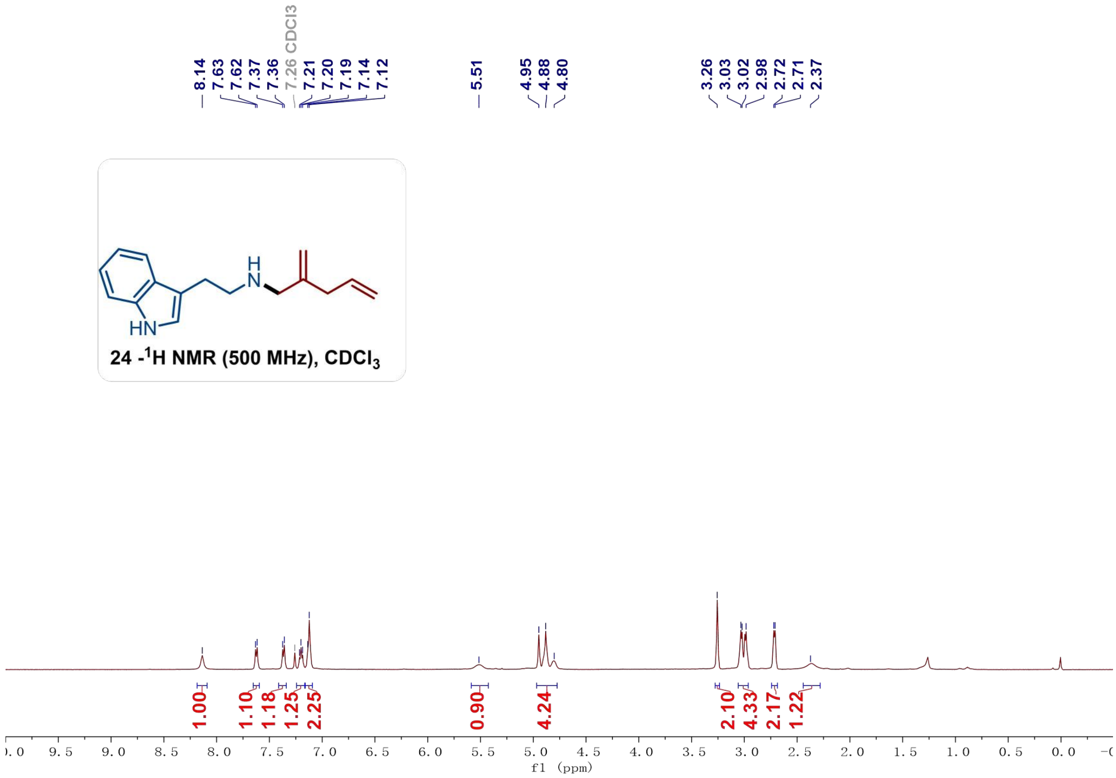

Supplementary Figure 108. ^{1}H NMR (500 MHz, $CDCl_3$) spectrum of compound **24**

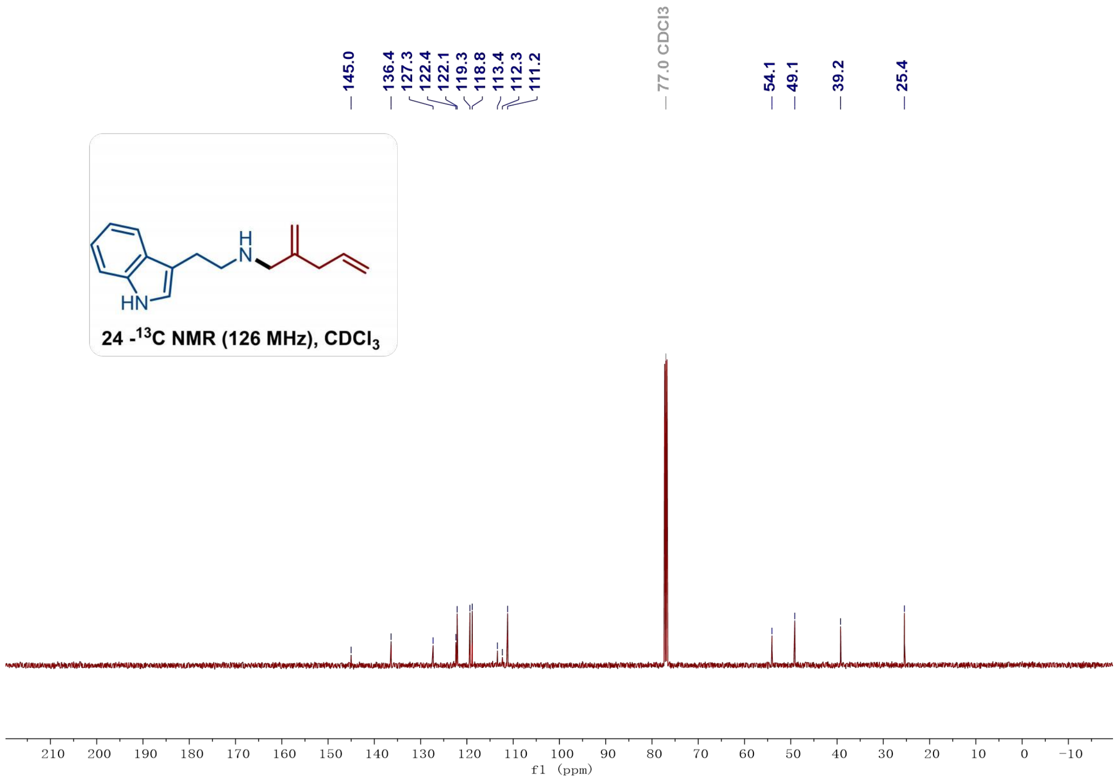

Supplementary Figure 109. ^{13}C NMR (126 MHz, $CDCl_3$) spectrum of compound **24**

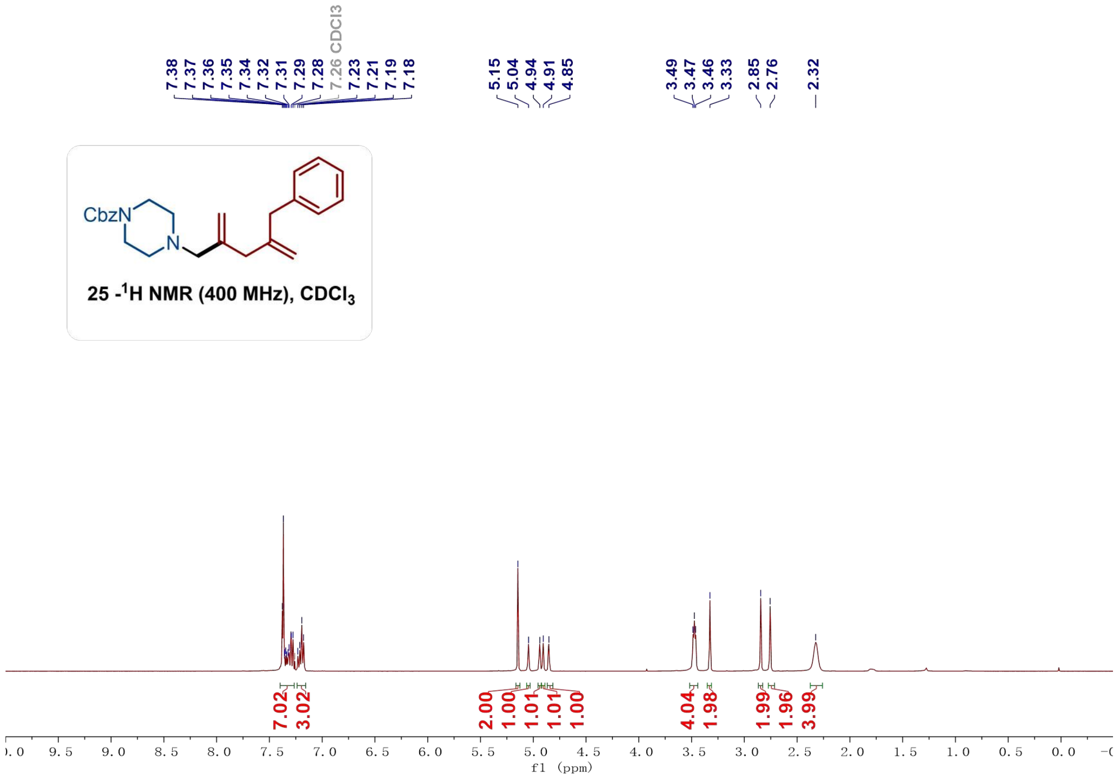

Supplementary Figure 110. ^{1}H NMR (400 MHz, $CDCl_3$) spectrum of compound **25**

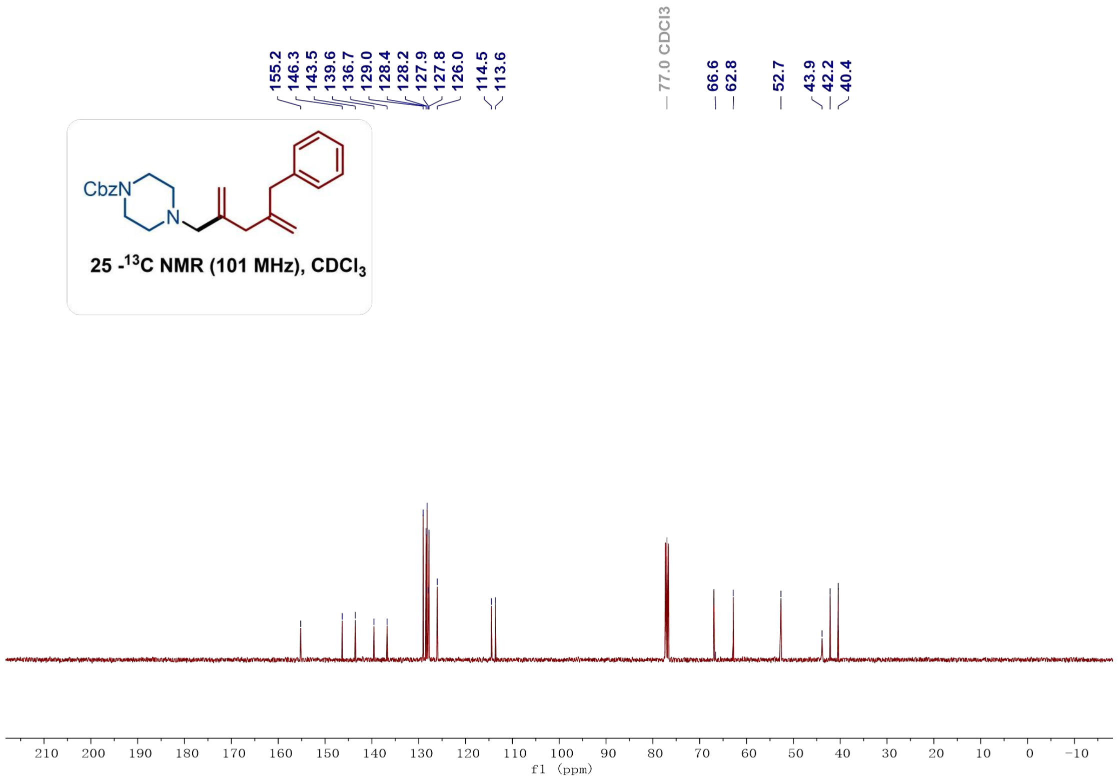

Supplementary Figure 111. ^{13}C NMR (101 MHz, $CDCl_3$) spectrum of compound **25**

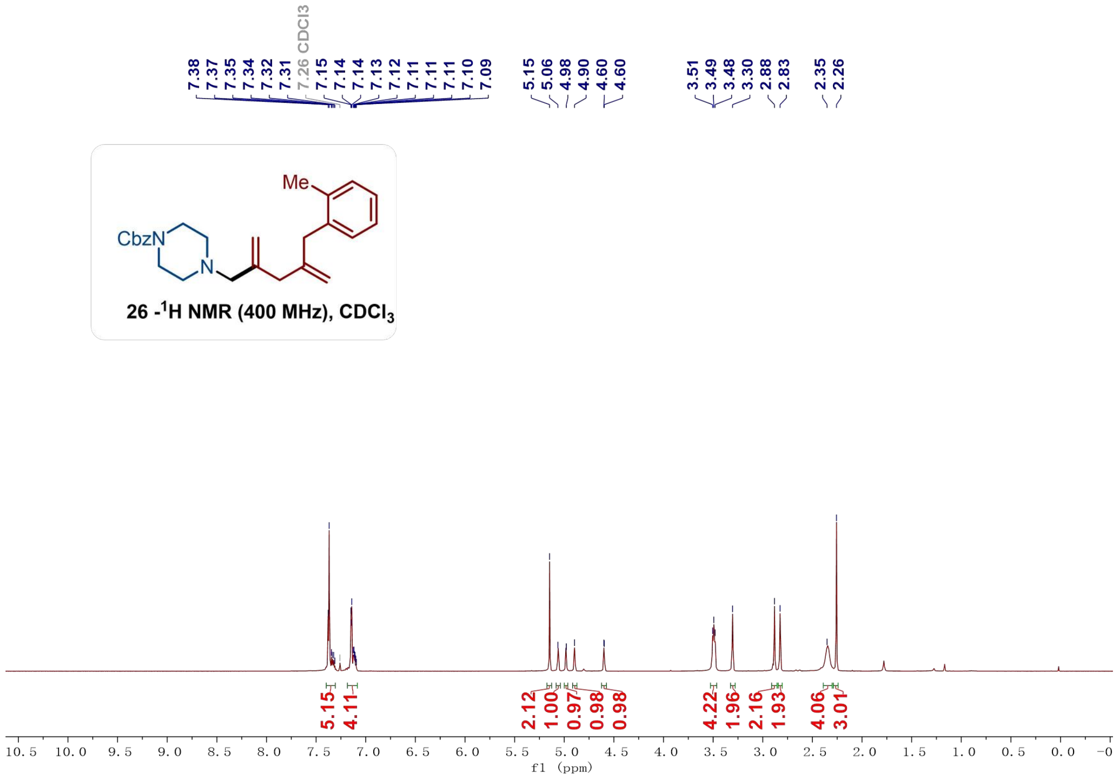

Supplementary Figure 112. ^{1}H NMR (400 MHz, $CDCl_3$) spectrum of compound **26**

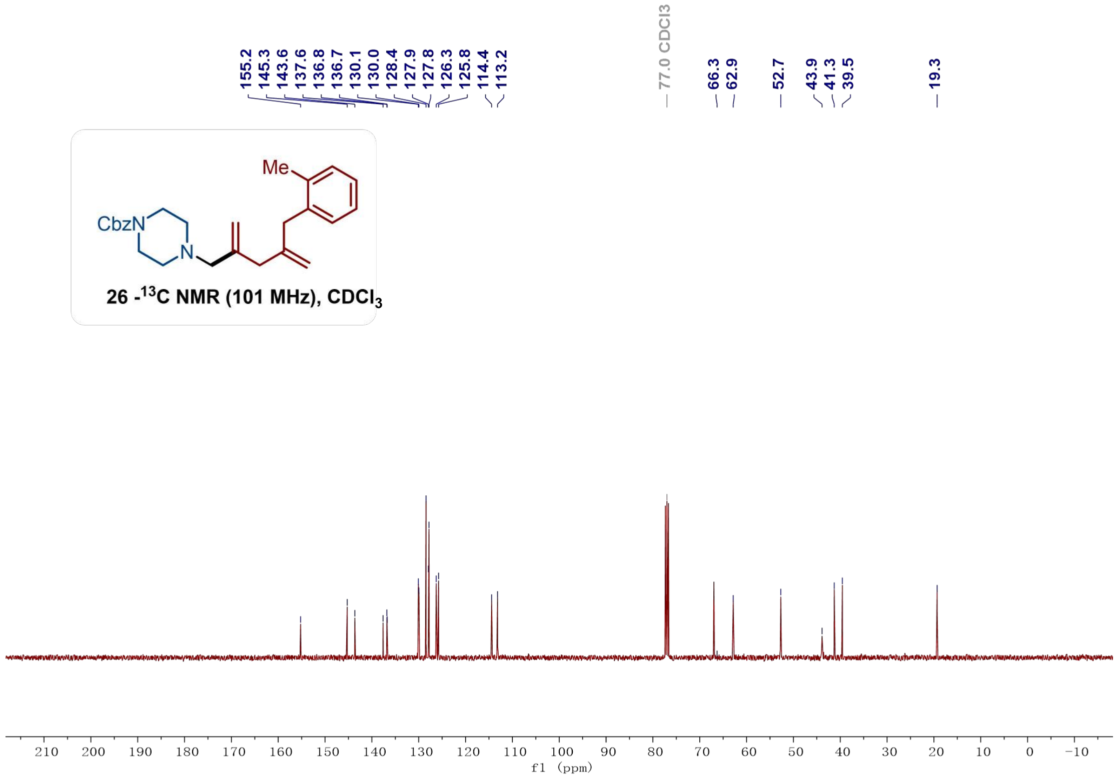

Supplementary Figure 113. ^{13}C NMR (101 MHz, $CDCl_3$) spectrum of compound **26**

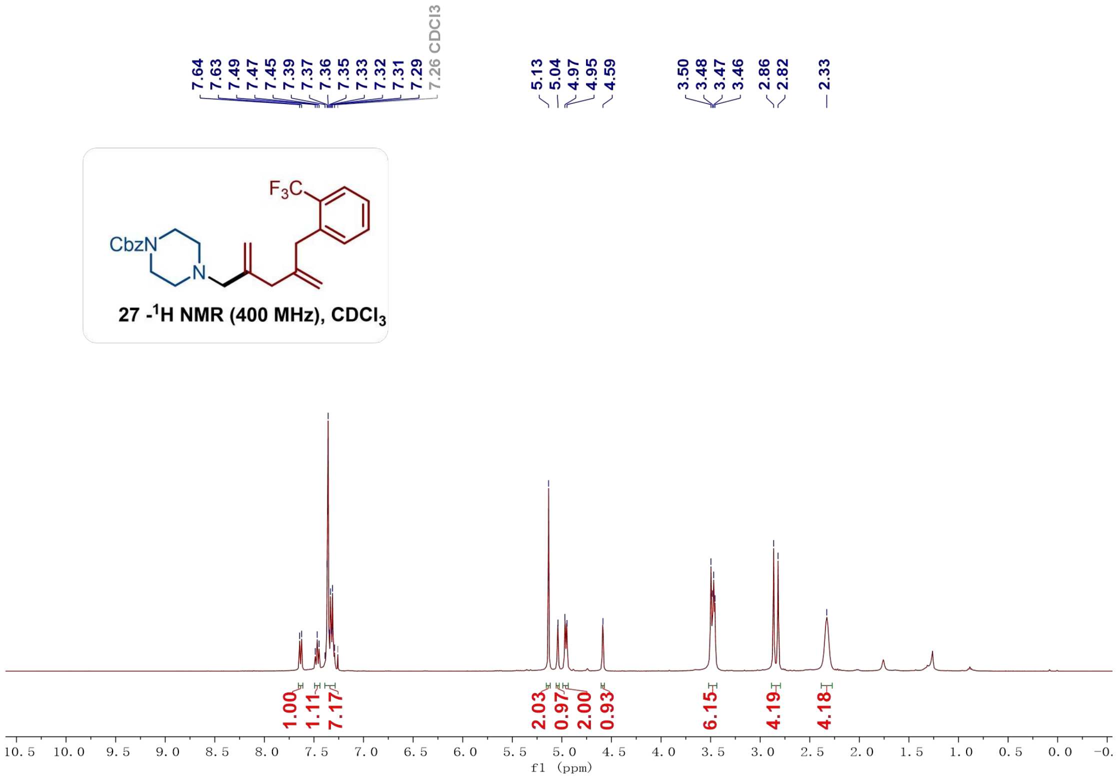

Supplementary Figure 114. ^{1}H NMR (400 MHz, $CDCl_3$) spectrum of compound **27**

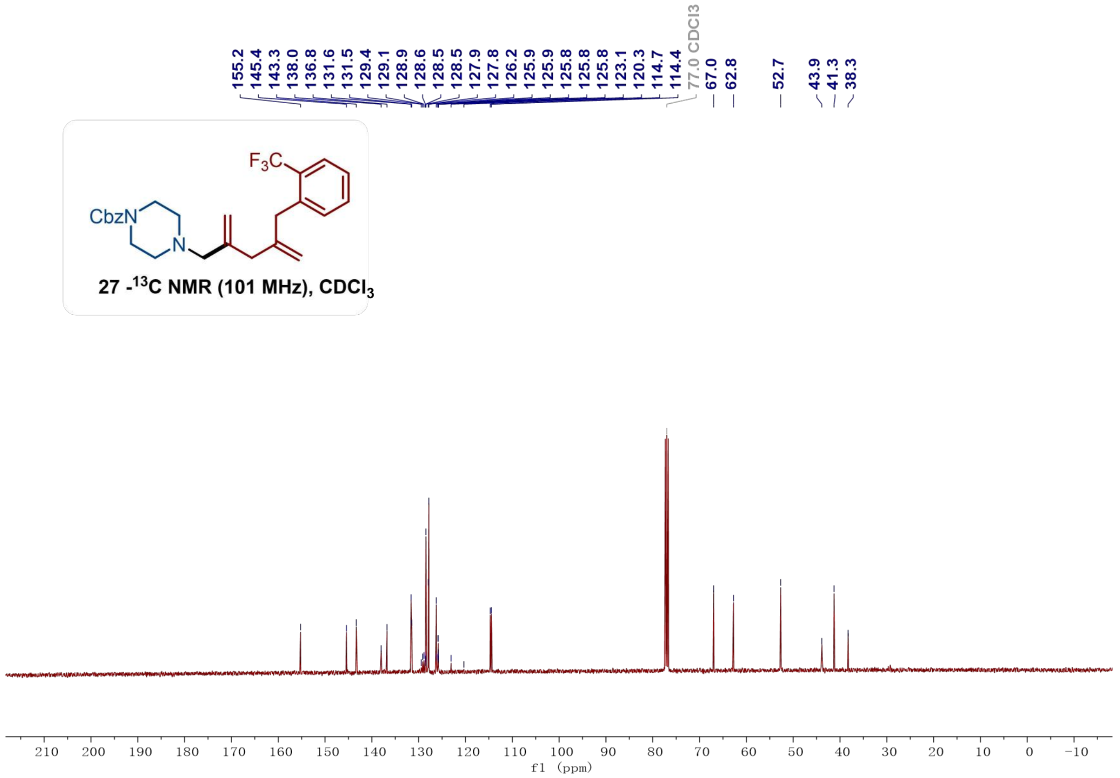

Supplementary Figure 115. ^{13}C NMR (101 MHz, $CDCl_3$) spectrum of compound **27**

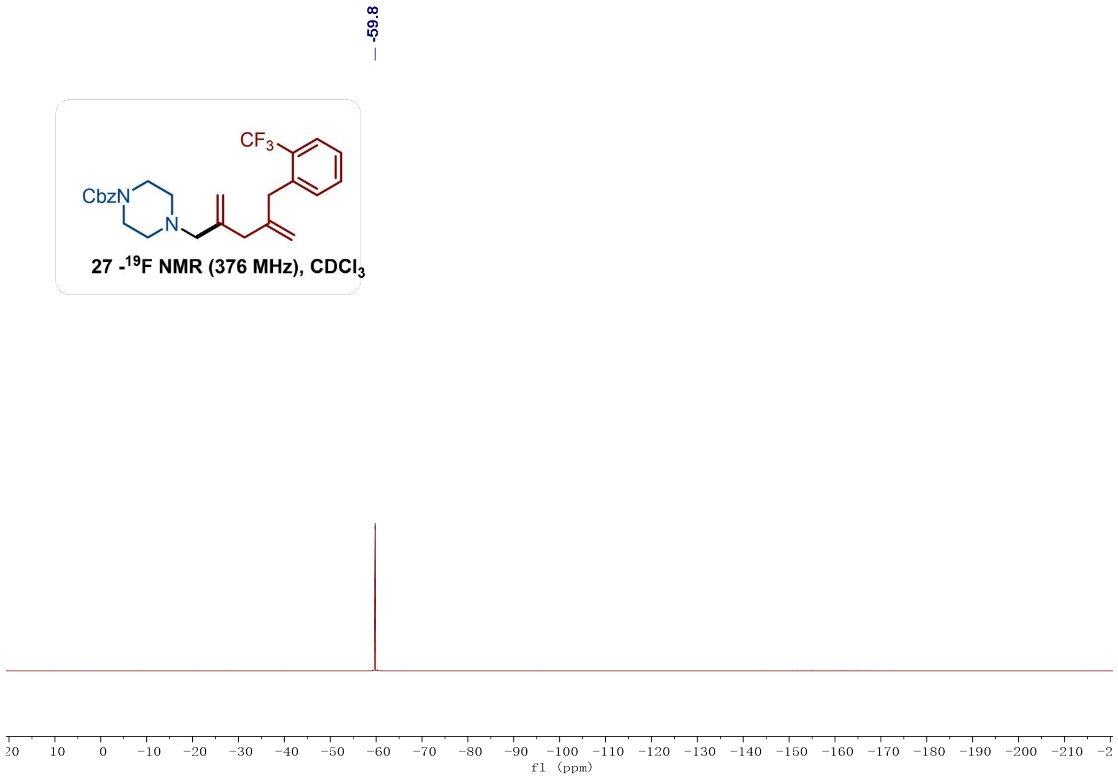

Supplementary Figure 116. ^{19}F NMR (376 MHz, $CDCl_3$) spectrum of compound **27**

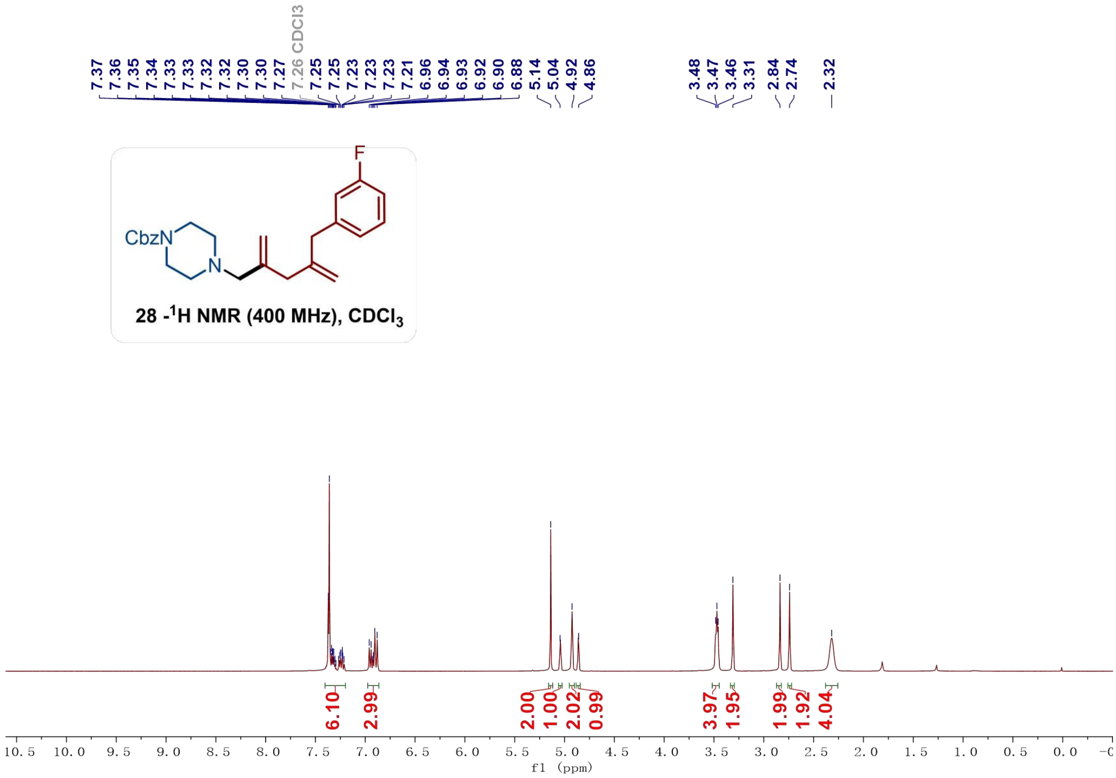

Supplementary Figure 117. ^{1}H NMR (400 MHz, $CDCl_3$) spectrum of compound **28**

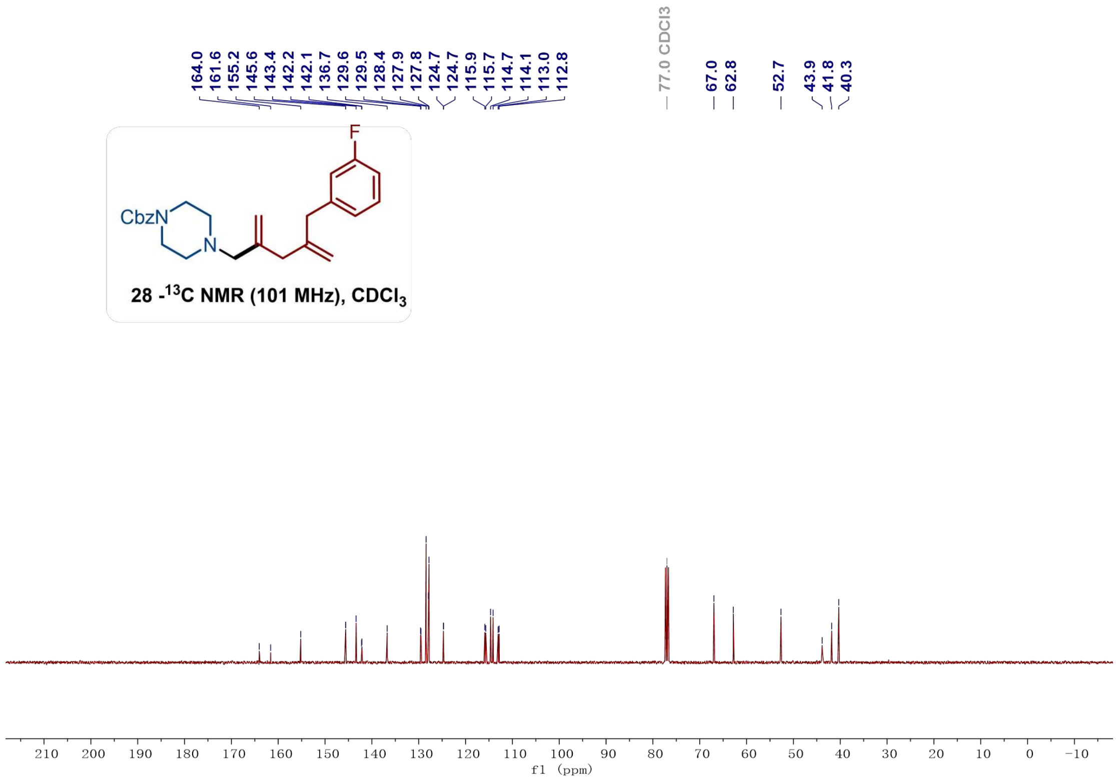

Supplementary Figure 118. ^{13}C NMR (101 MHz, $CDCl_3$) spectrum of compound **28**

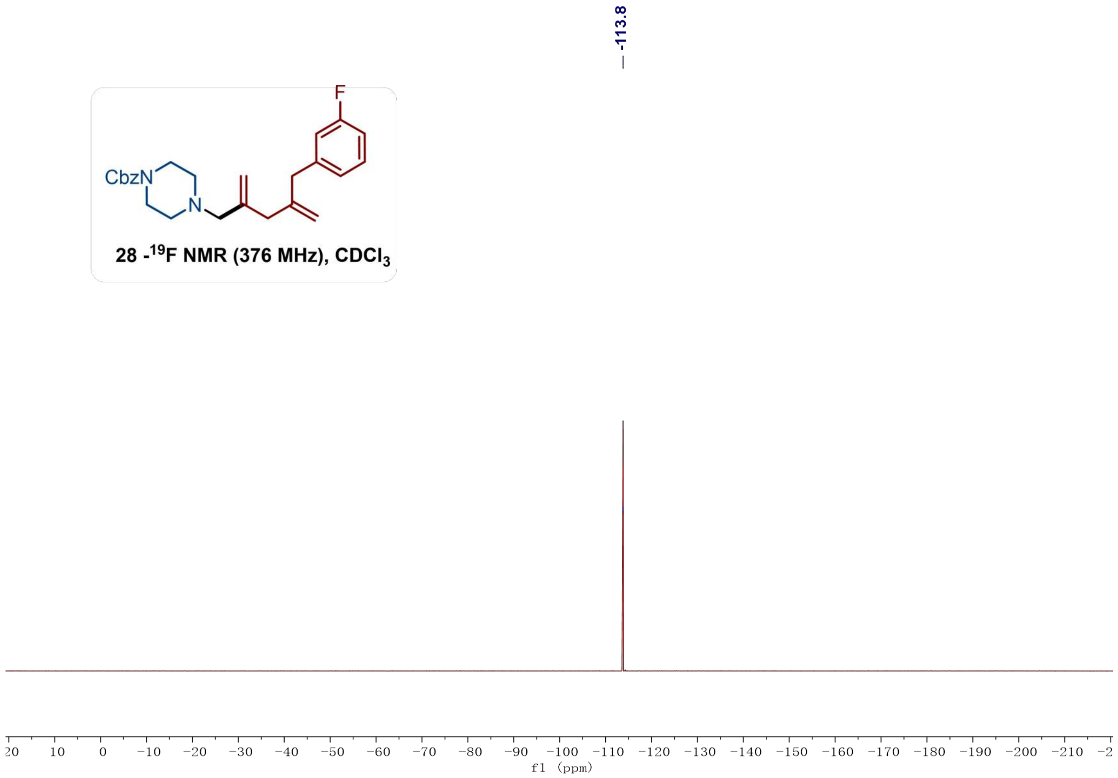

Supplementary Figure 119. ^{19}F NMR (376 MHz, $CDCl_3$) spectrum of compound **28**

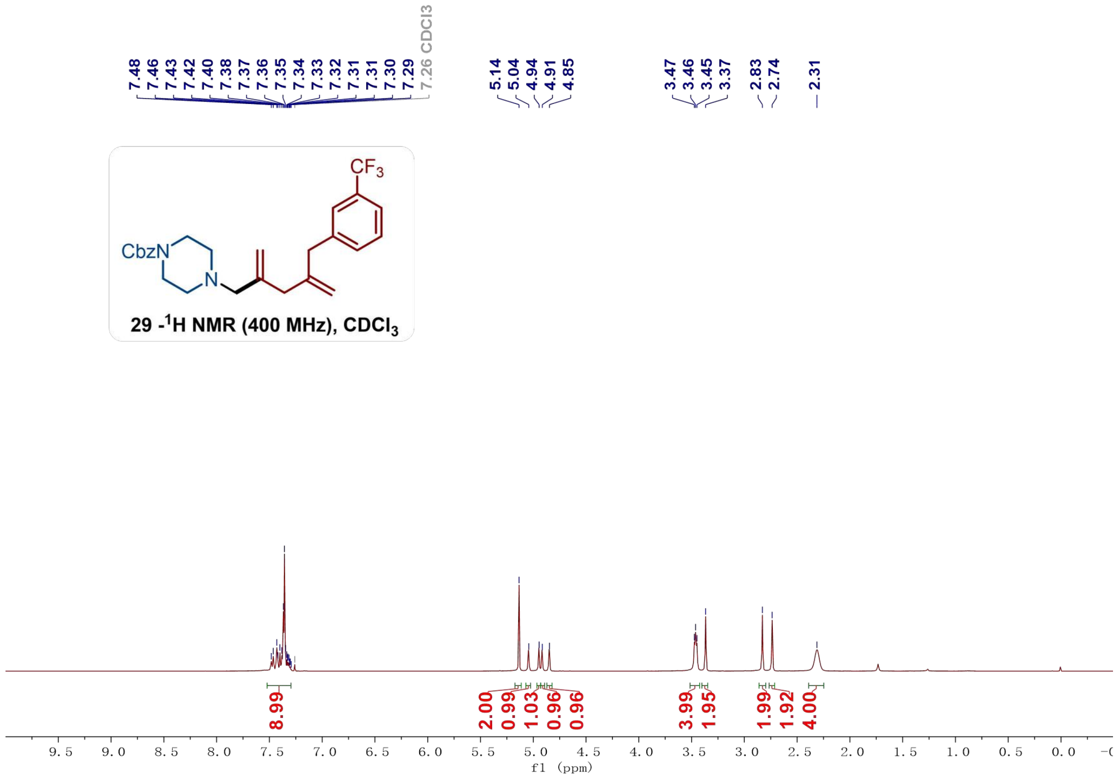

Supplementary Figure 120. ^{1}H NMR (400 MHz, $CDCl_3$) spectrum of compound **29**

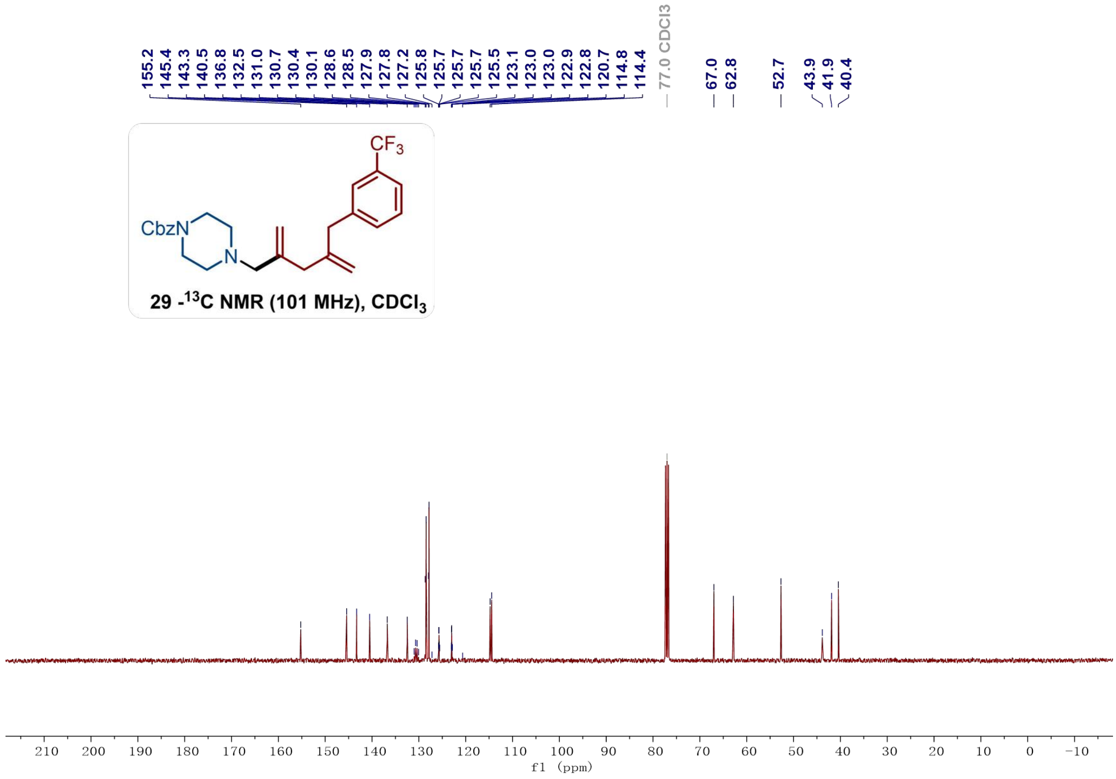

Supplementary Figure 121. ^{13}C NMR (101 MHz, $CDCl_3$) spectrum of compound **29**

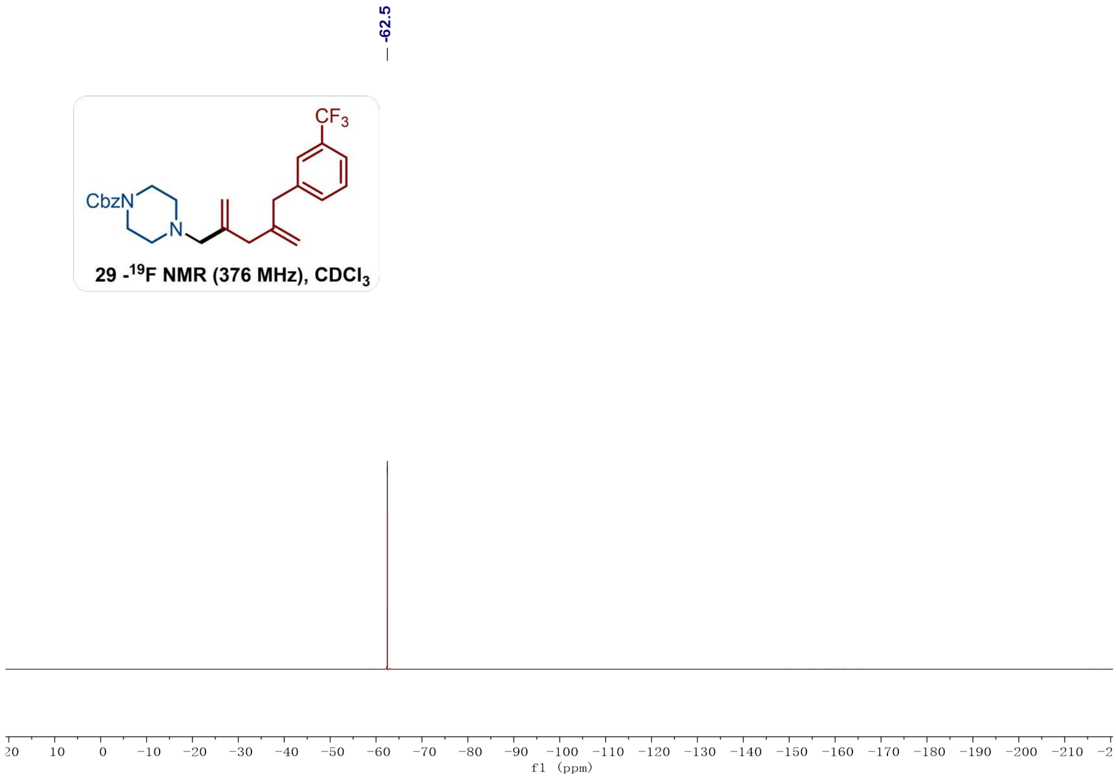

Supplementary Figure 122. ^{19}F NMR (376 MHz, $CDCl_3$) spectrum of compound **29**

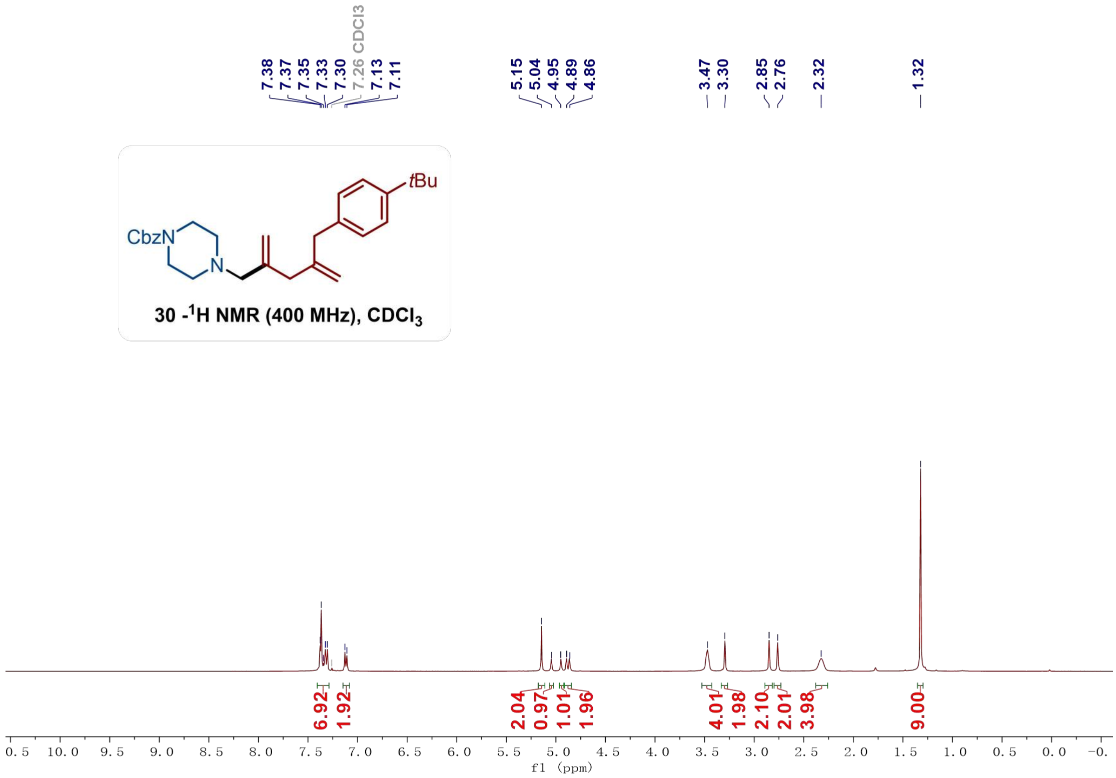

Supplementary Figure 123. ^{1}H NMR (400 MHz, $CDCl_3$) spectrum of compound **30**

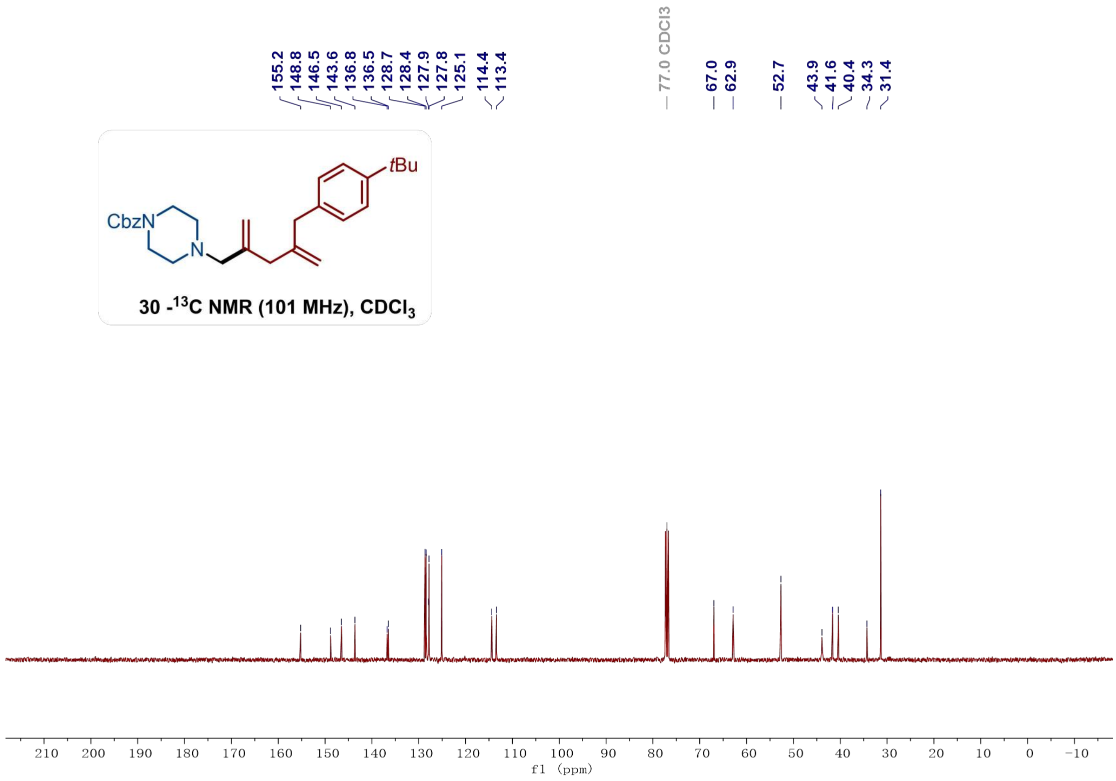

Supplementary Figure 124. ^{13}C NMR (101 MHz, $CDCl_3$) spectrum of compound **30**

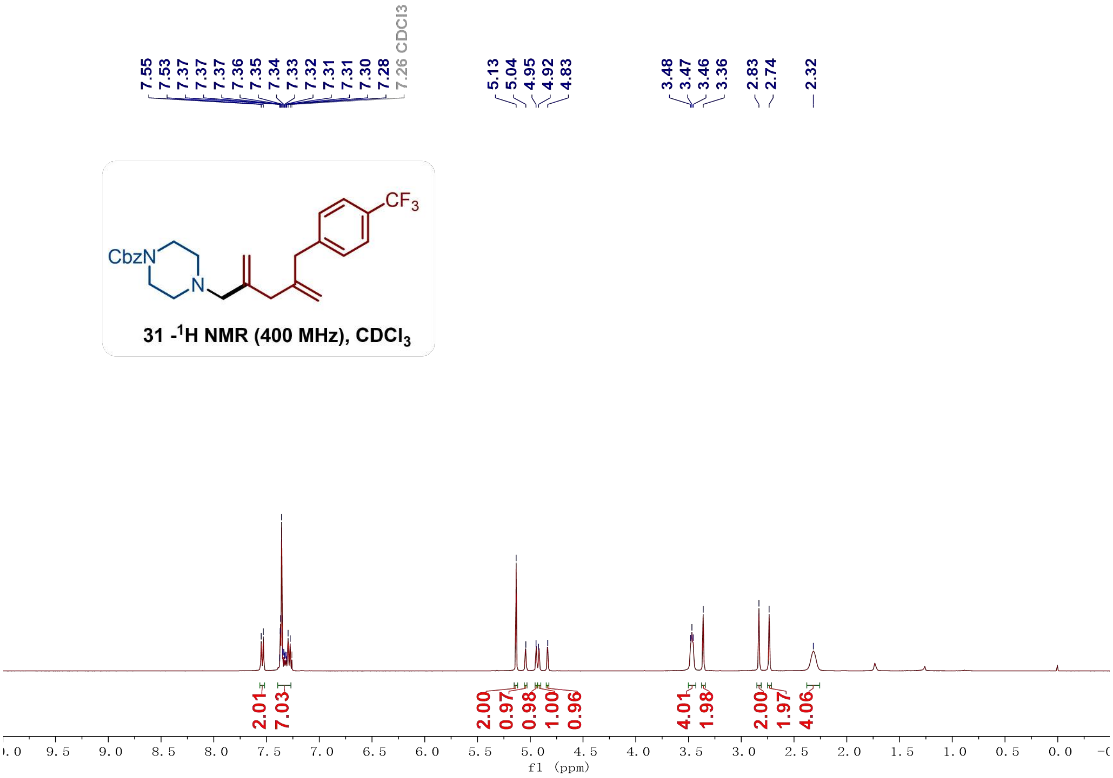

Supplementary Figure 125. ^{1}H NMR (400 MHz, $CDCl_3$) spectrum of compound **31**

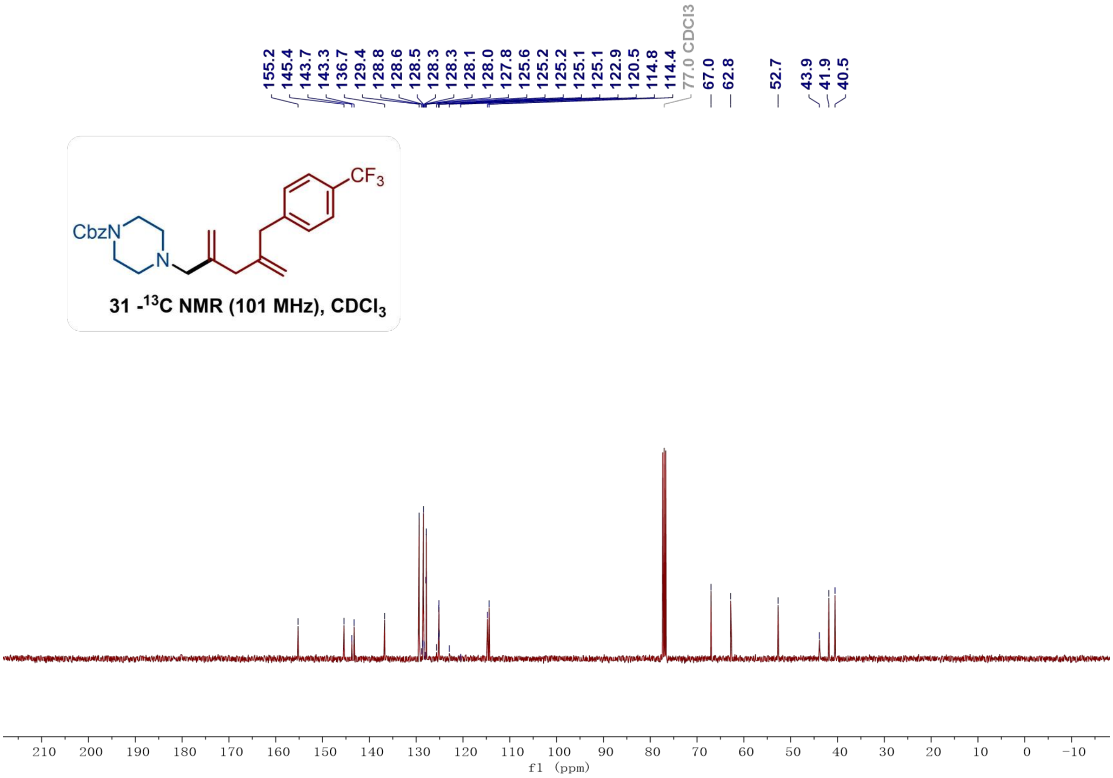

Supplementary Figure 126. ^{13}C NMR (101 MHz, $CDCl_3$) spectrum of compound **31**

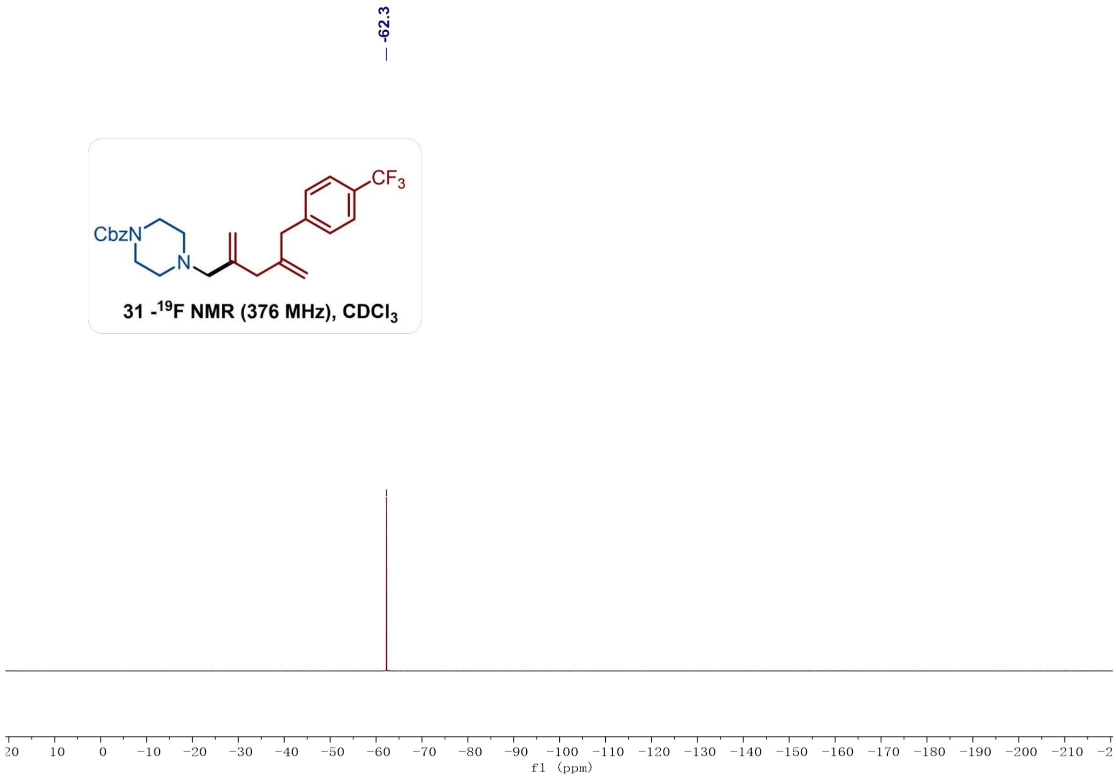

Supplementary Figure 127. ^{19}F NMR (376 MHz, $CDCl_3$) spectrum of compound **31**

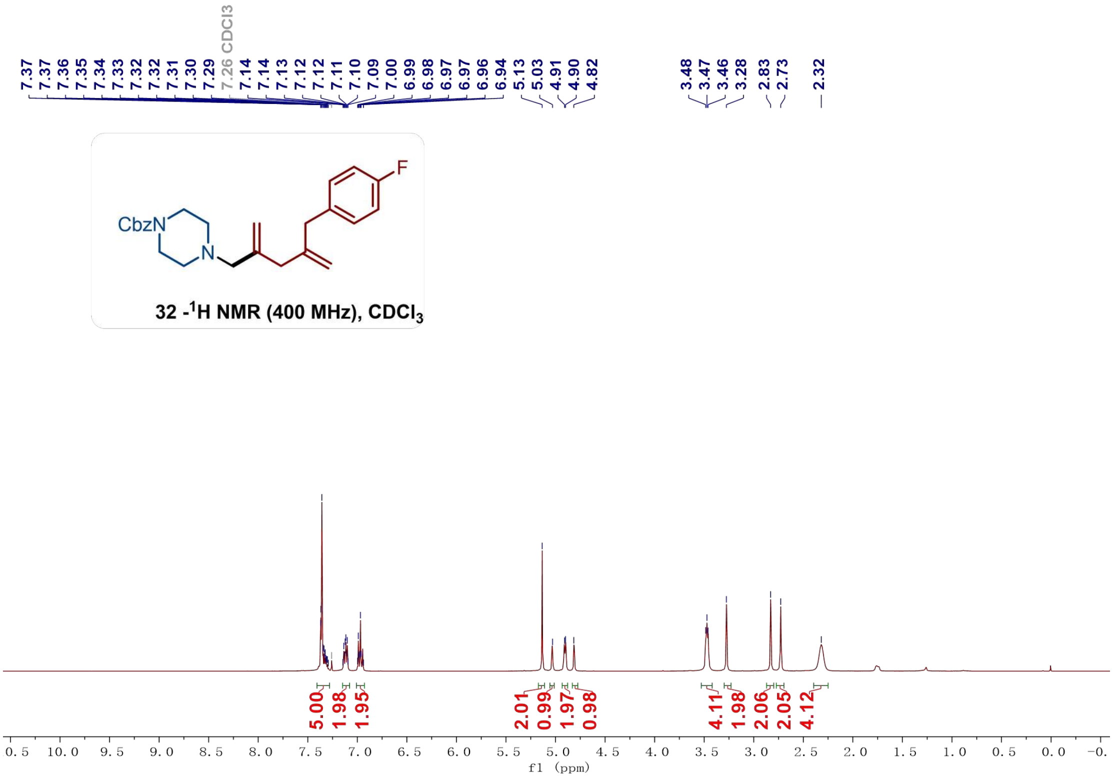

Supplementary Figure 128. ^{1}H NMR (400 MHz, $CDCl_3$) spectrum of compound **32**

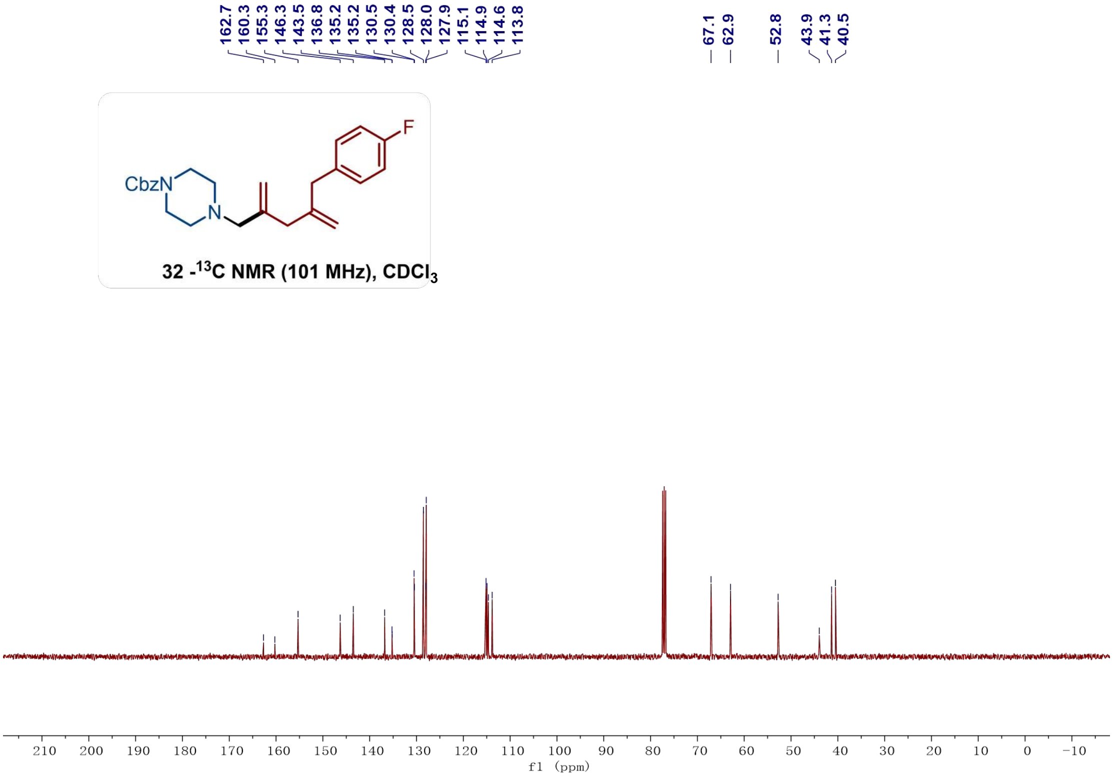

Supplementary Figure 129. ^{13}C NMR (101 MHz, $CDCl_3$) spectrum of compound **32**

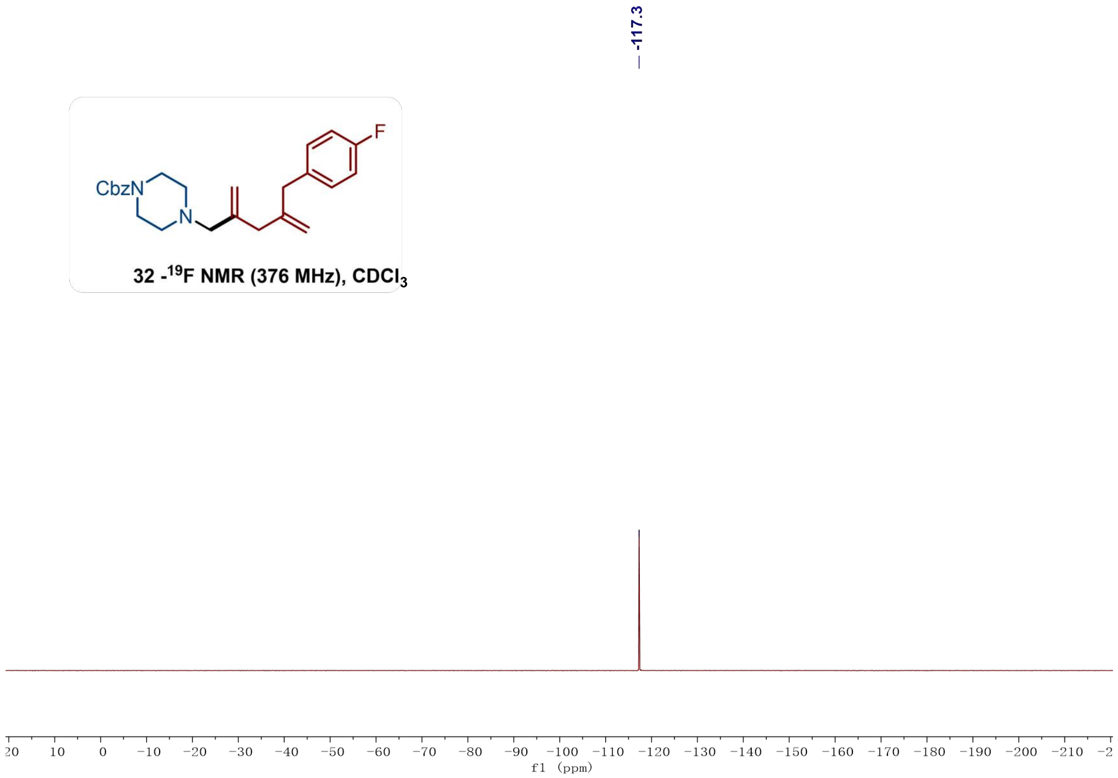

Supplementary Figure 130. ^{19}F NMR (376 MHz, $CDCl_3$) spectrum of compound **32**

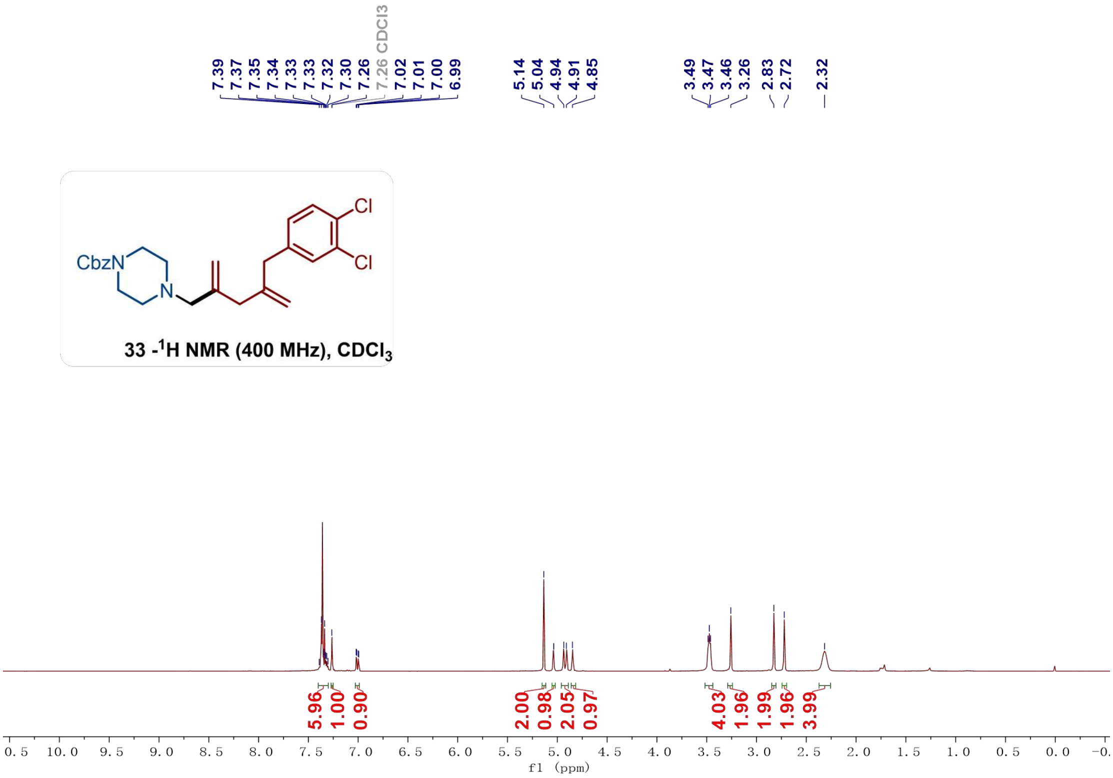

Supplementary Figure 131. ^{1}H NMR (400 MHz, $CDCl_3$) spectrum of compound **33**

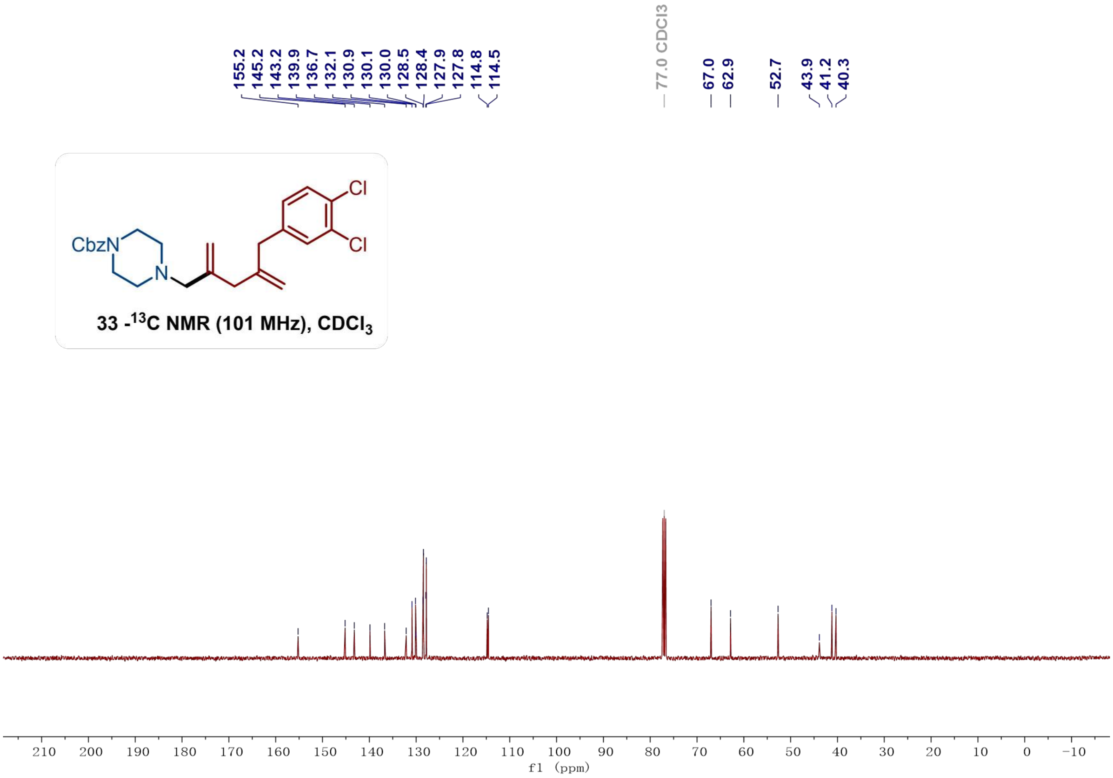

Supplementary Figure 132. ^{13}C NMR (101 MHz, $CDCl_3$) spectrum of compound **33**

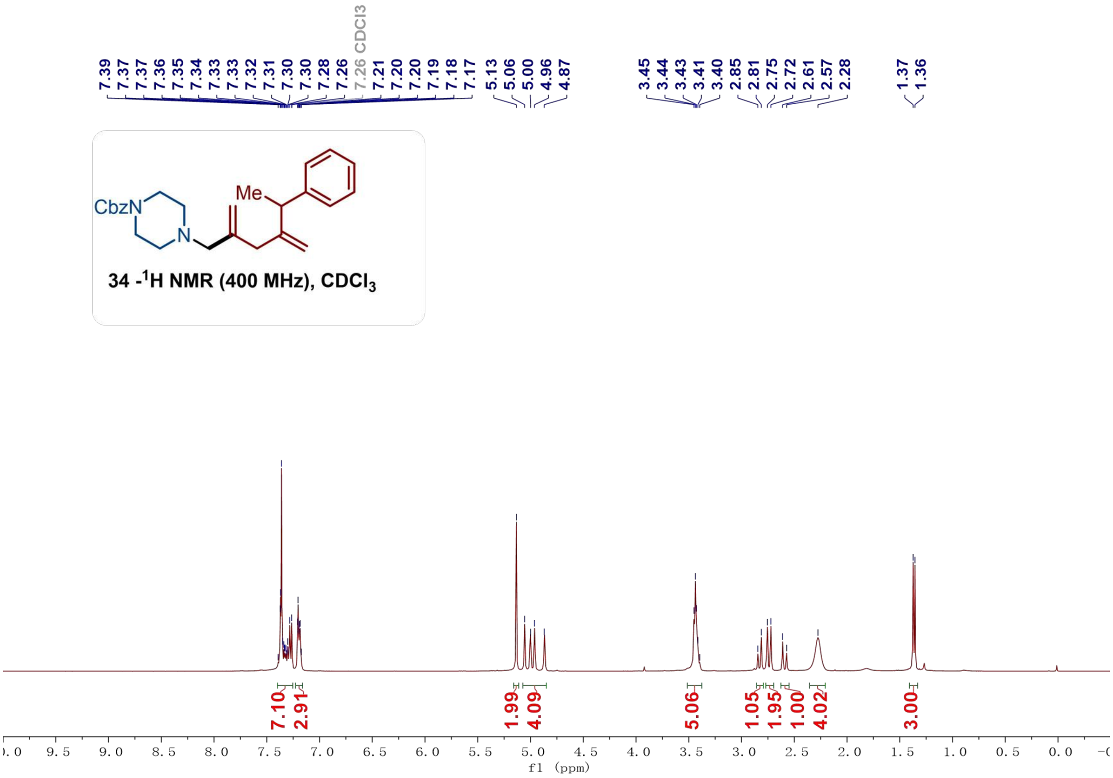

Supplementary Figure 133. ^{1}H NMR (400 MHz, $CDCl_3$) spectrum of compound **34**

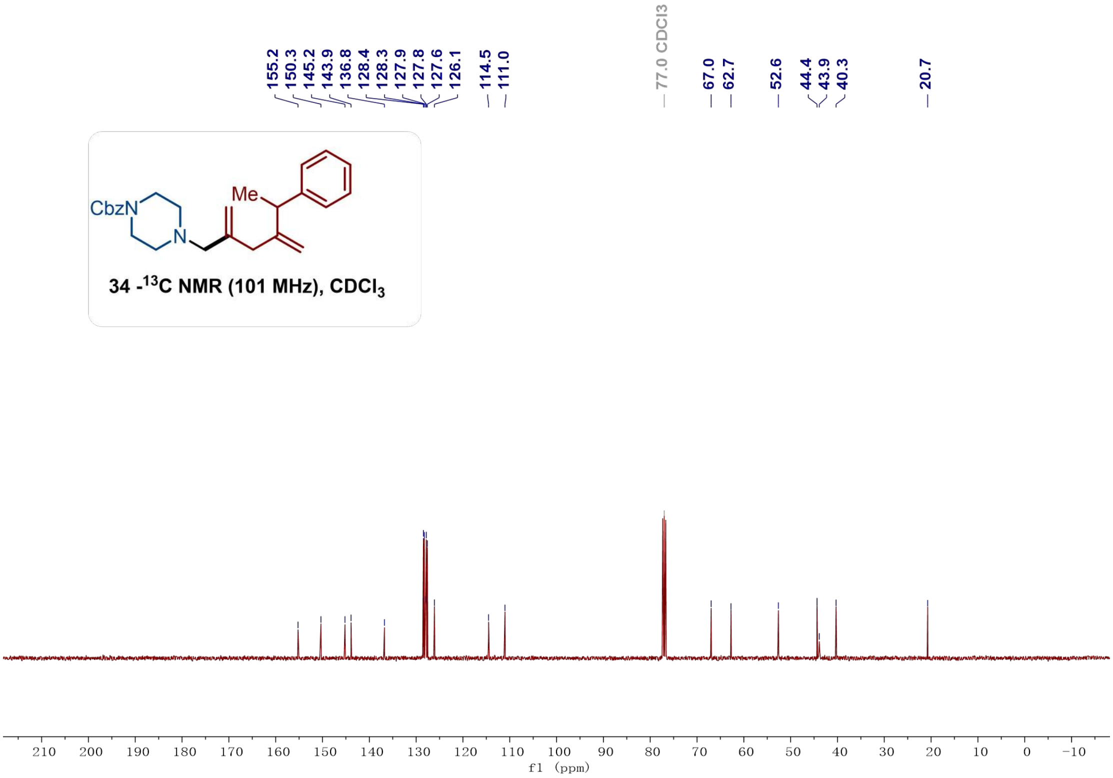

Supplementary Figure 134. ^{13}C NMR (101 MHz, $CDCl_3$) spectrum of compound **34**

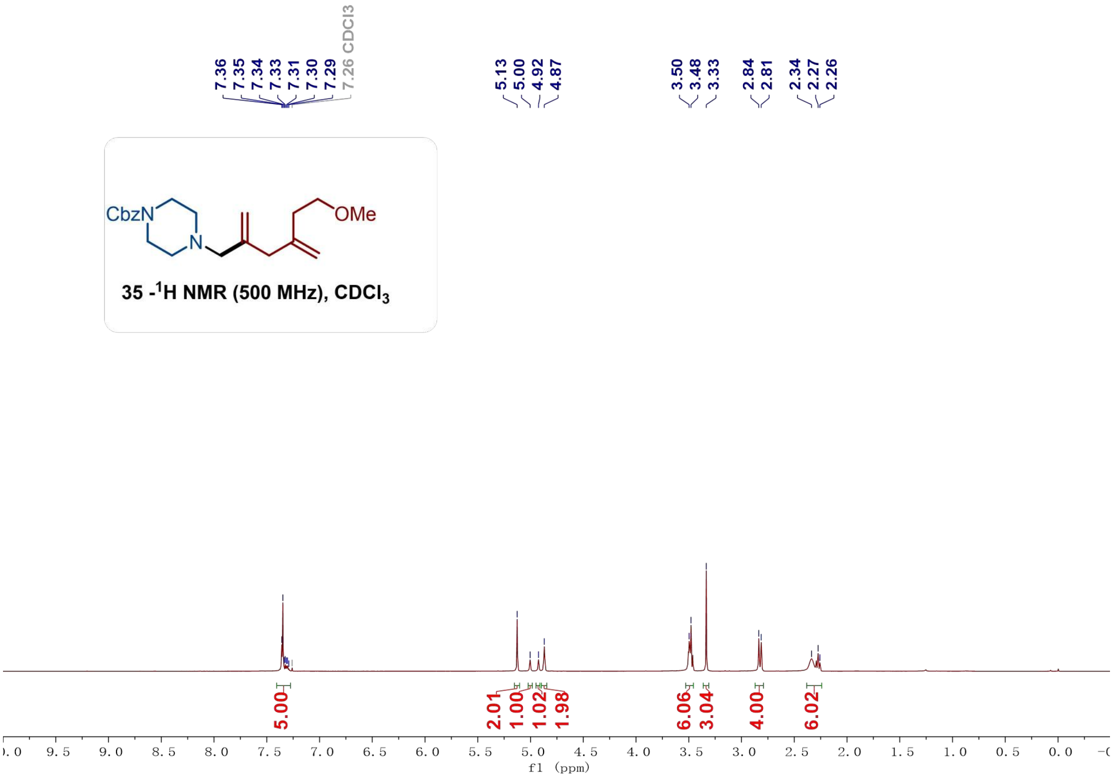

Supplementary Figure 135. ^{1}H NMR (500 MHz, $CDCl_3$) spectrum of compound **35**

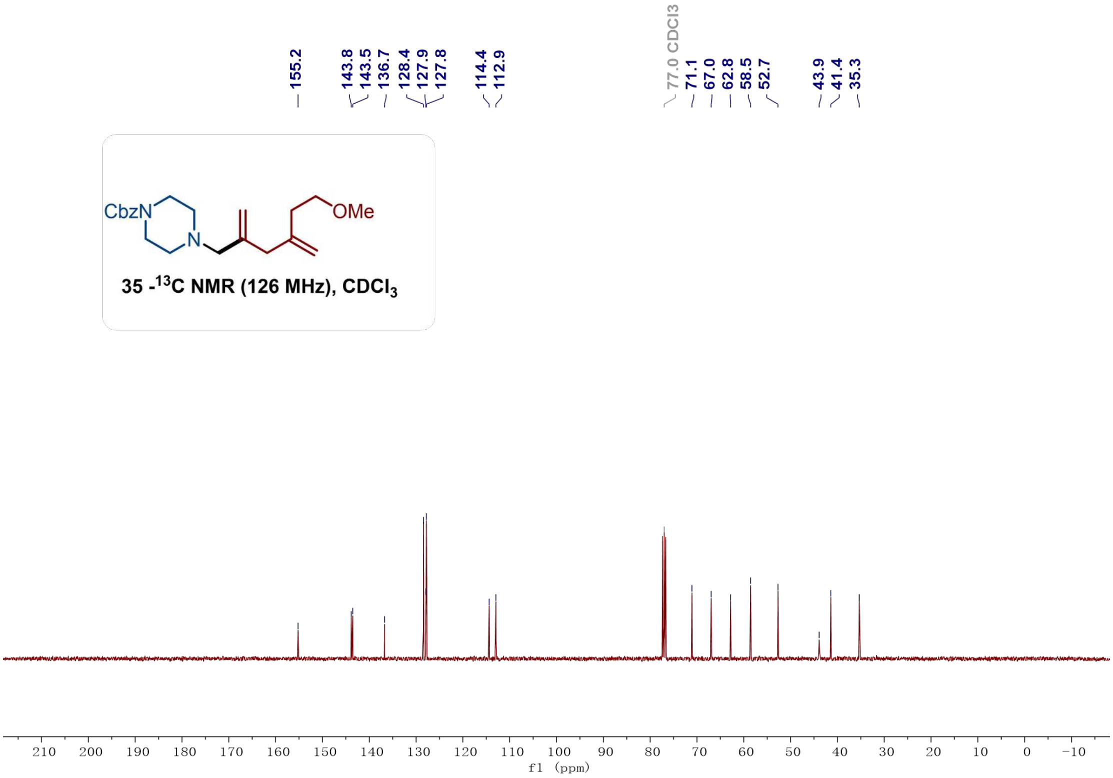

Supplementary Figure 136. ^{13}C NMR (126 MHz, $CDCl_3$) spectrum of compound **35**

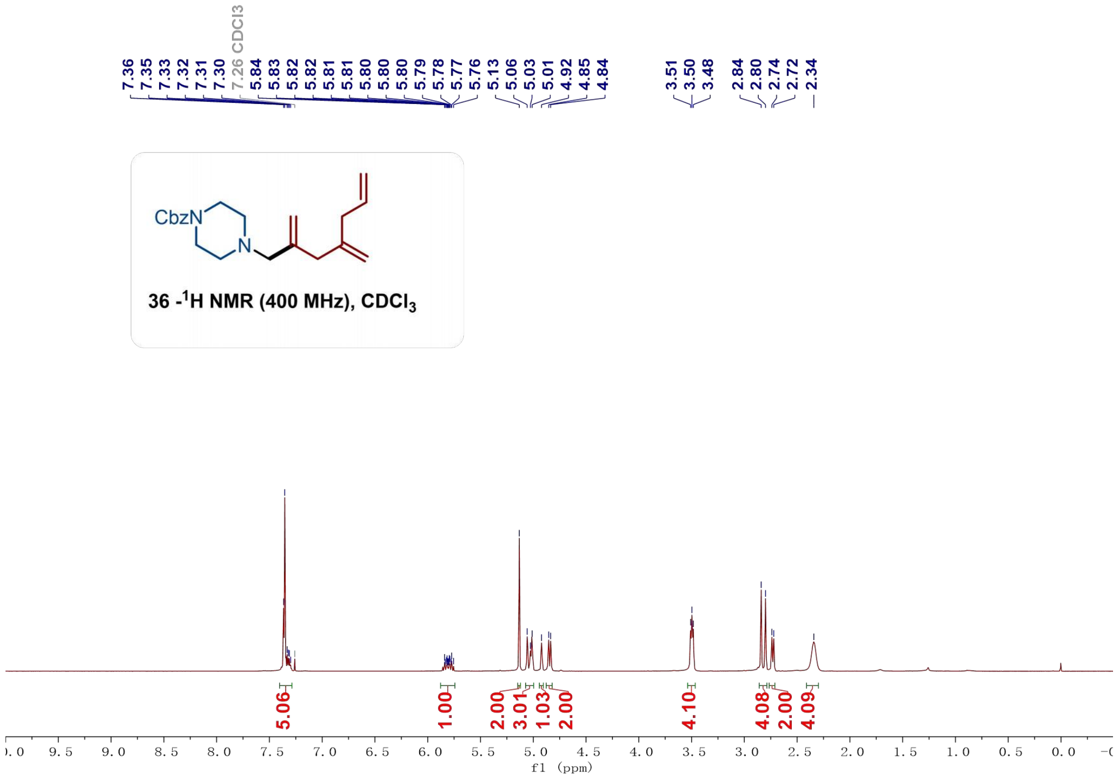

Supplementary Figure 137. ^{1}H NMR (400 MHz, $CDCl_3$) spectrum of compound **36**

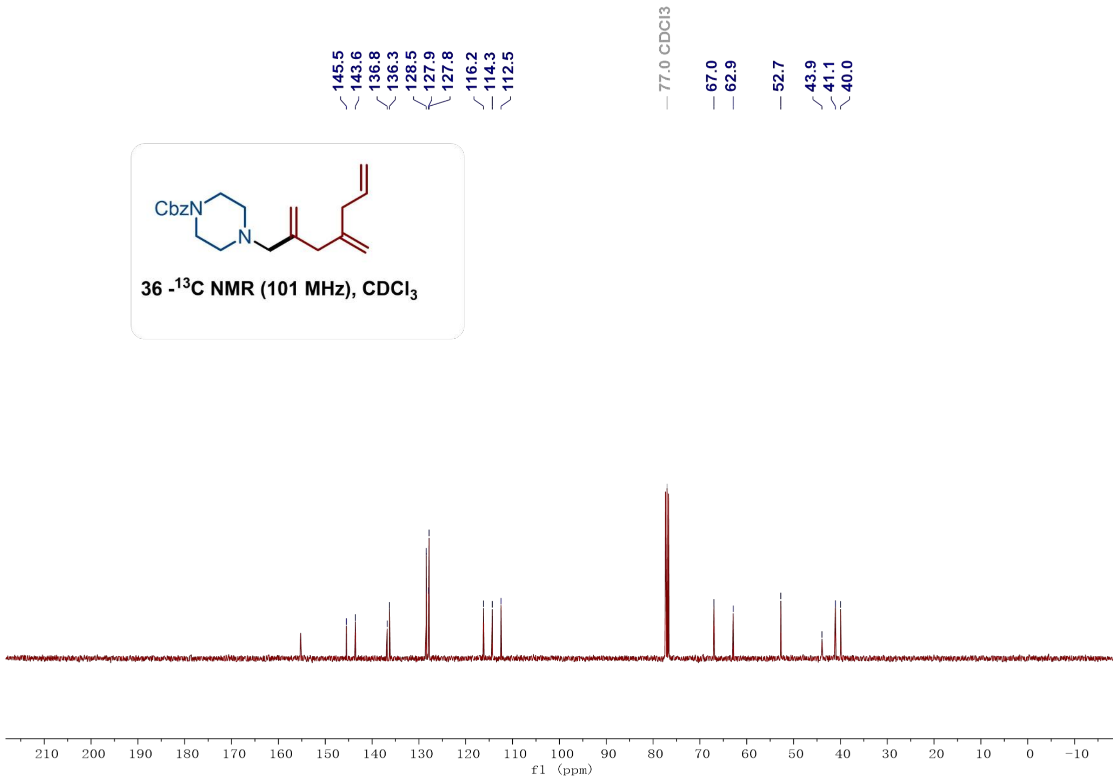

Supplementary Figure 138. ^{13}C NMR (101 MHz, $CDCl_3$) spectrum of compound **36**

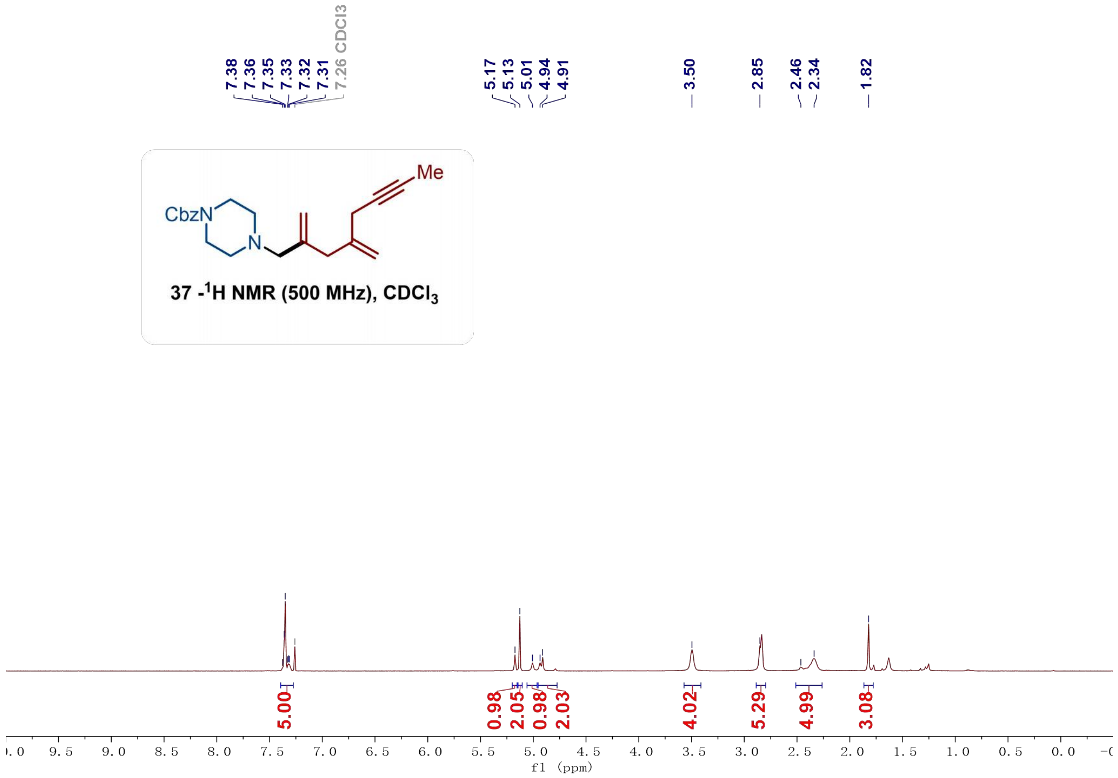

Supplementary Figure 139. ^{1}H NMR (500 MHz, $CDCl_3$) spectrum of compound **37**

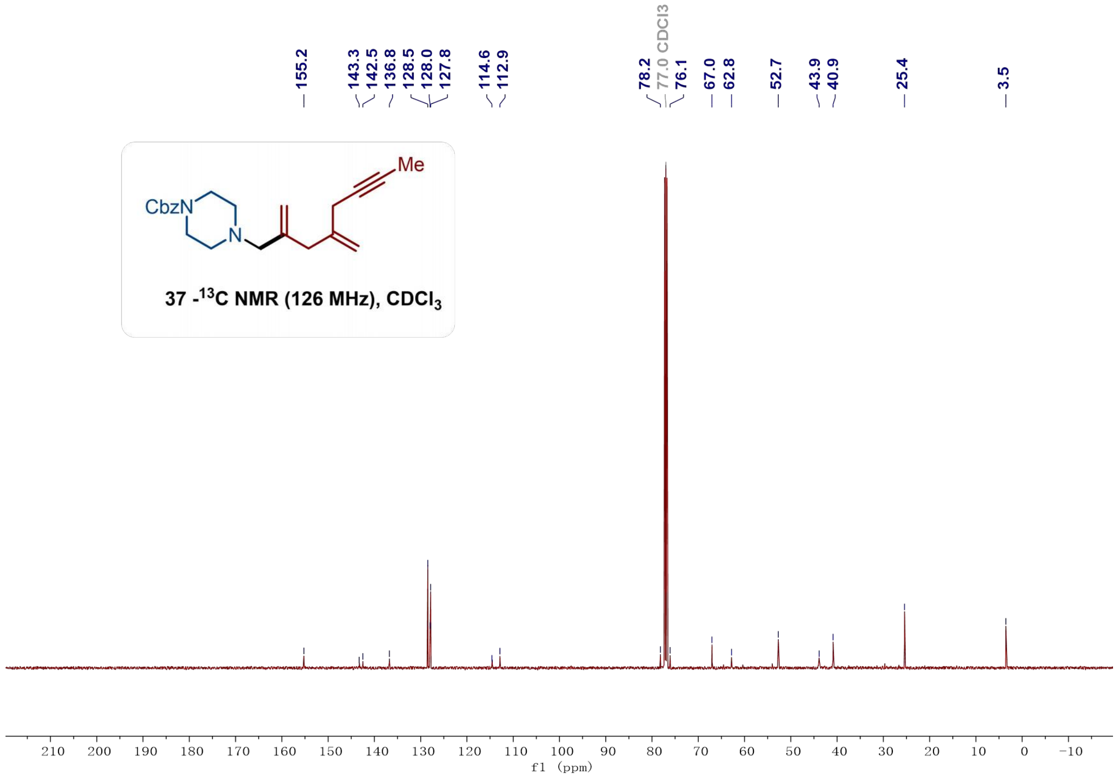

Supplementary Figure 140. ^{13}C NMR (126 MHz, $CDCl_3$) spectrum of compound **37**

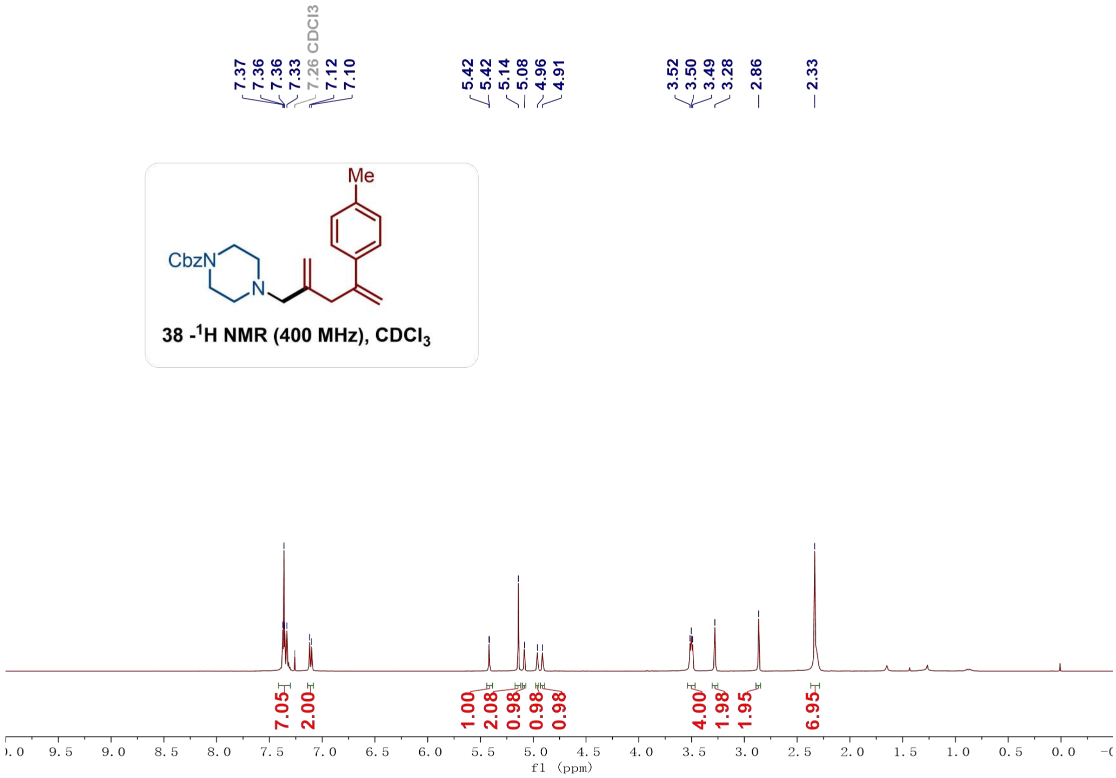

Supplementary Figure 141. ^{1}H NMR (400 MHz, $CDCl_3$) spectrum of compound **38**

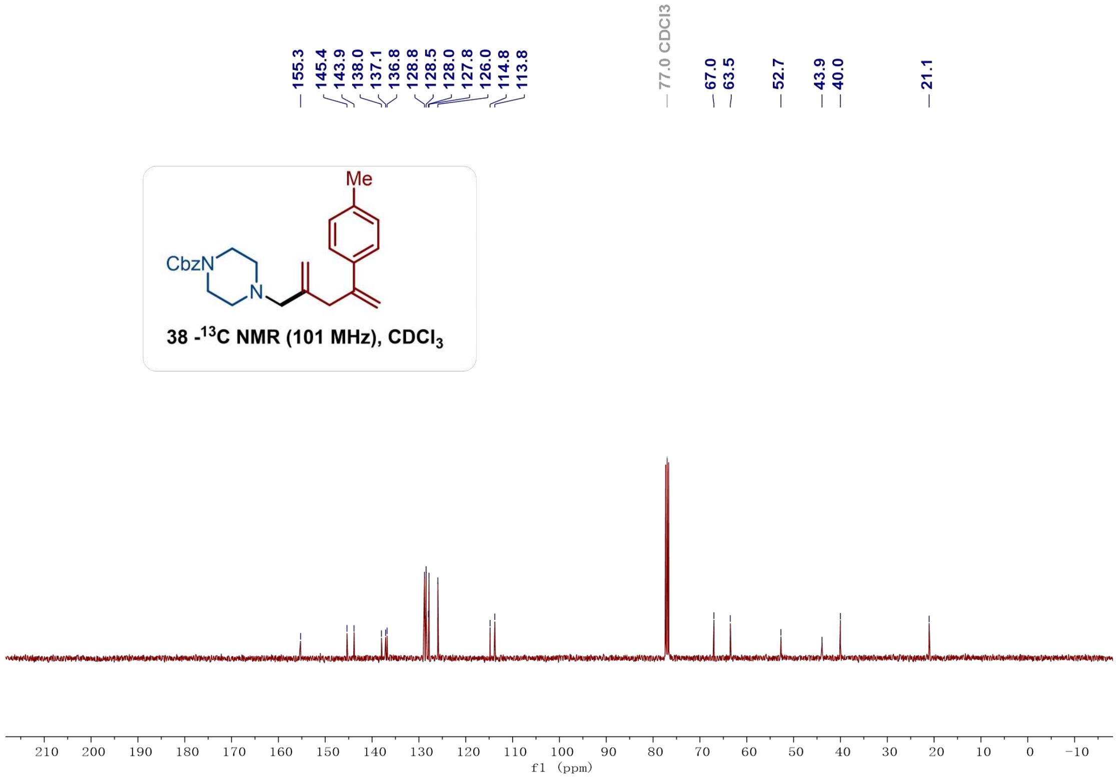

Supplementary Figure 142. ^{13}C NMR (101 MHz, $CDCl_3$) spectrum of compound **38**

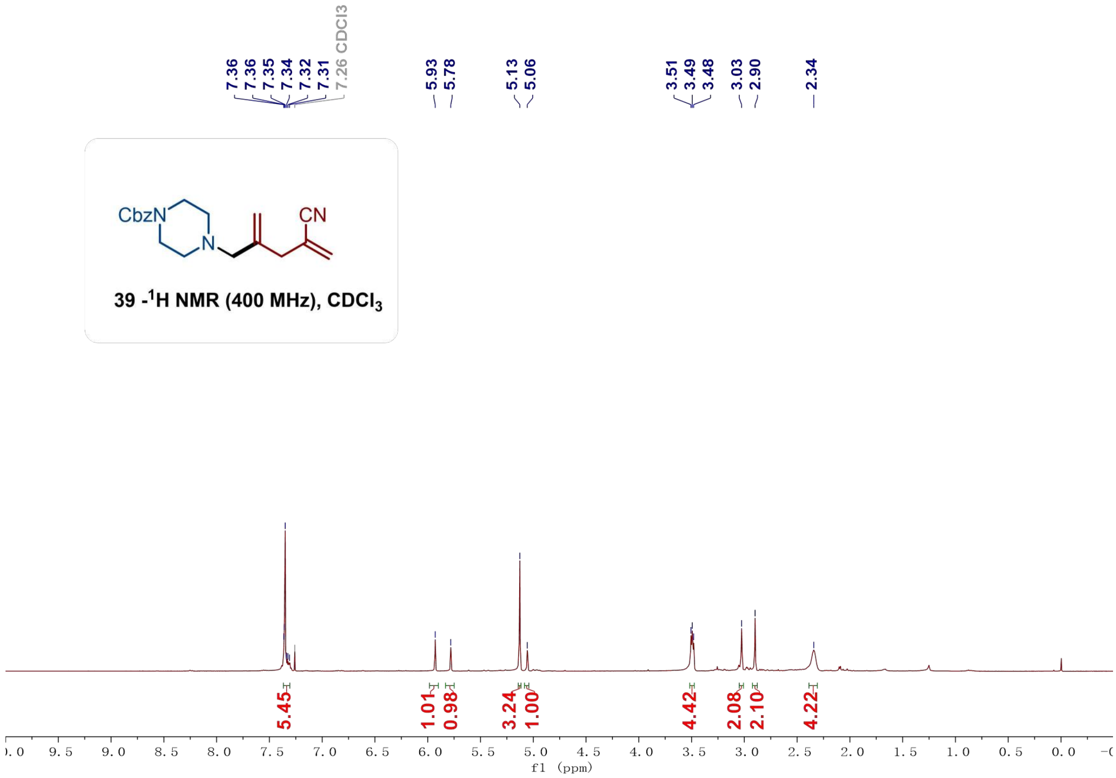

Supplementary Figure 143. ^{1}H NMR (400 MHz, $CDCl_3$) spectrum of compound **39**

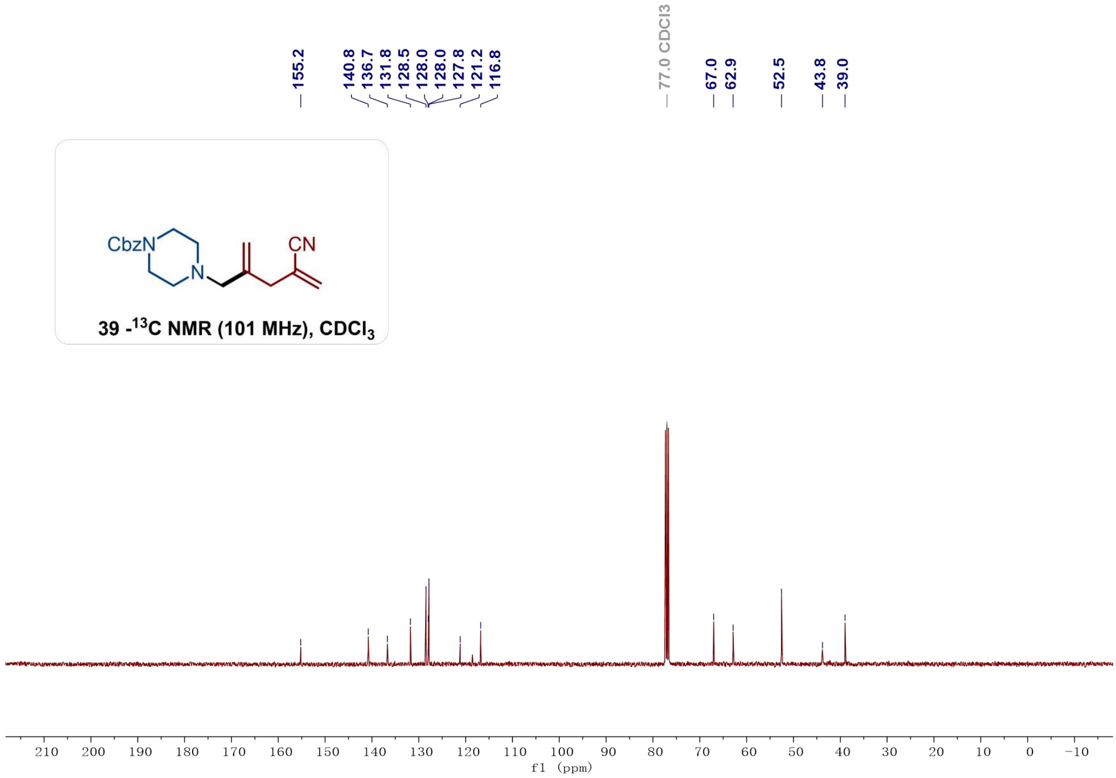

Supplementary Figure 144. ^{13}C NMR (101 MHz, $CDCl_3$) spectrum of compound **39**

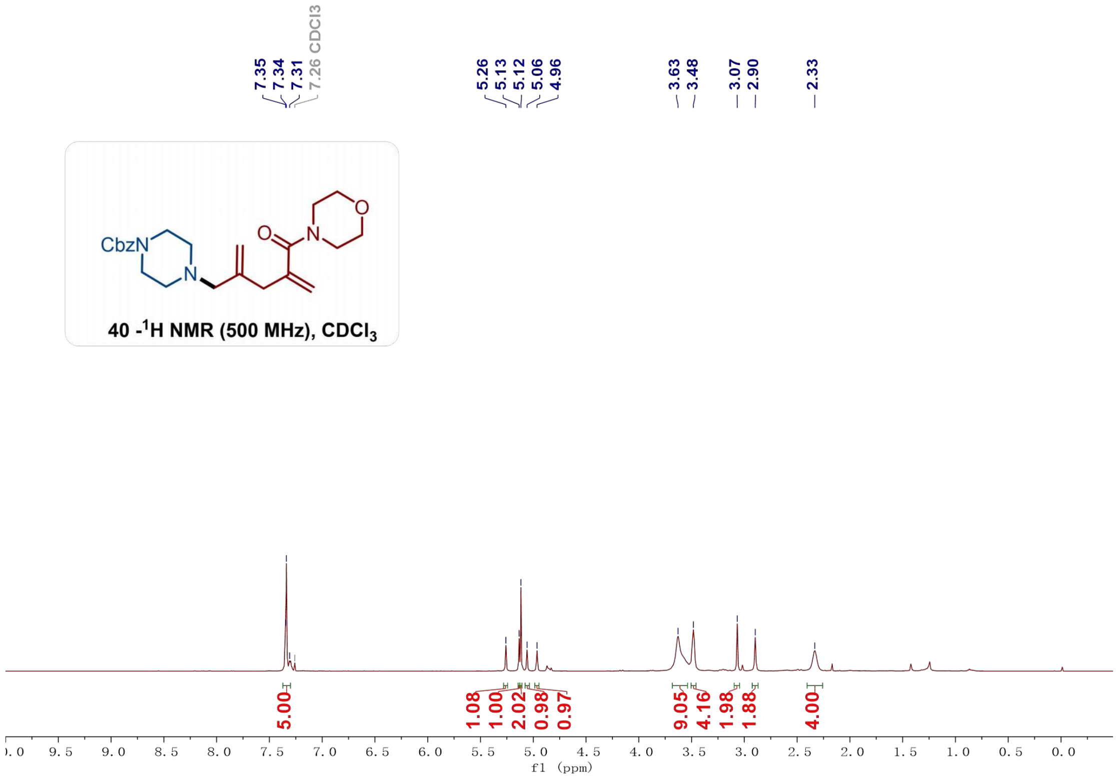

Supplementary Figure 145. ^{1}H NMR (500 MHz, $CDCl_3$) spectrum of compound **40**

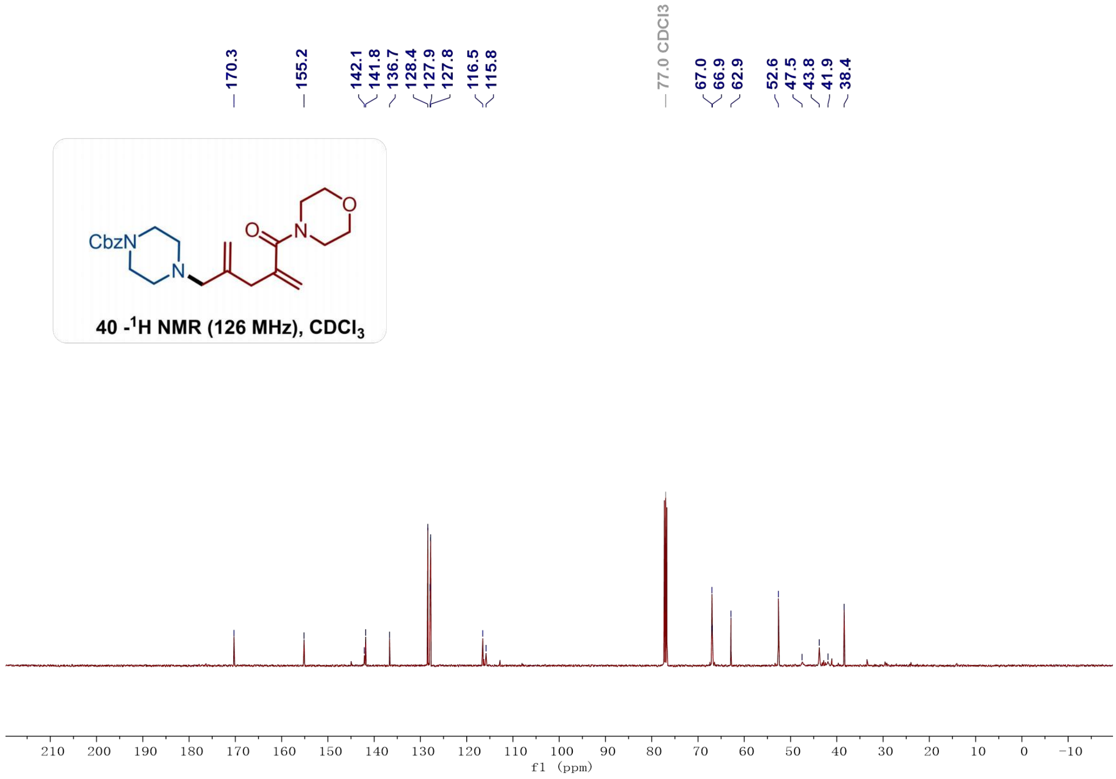

Supplementary Figure 146. ^{13}C NMR (126 MHz, $CDCl_3$) spectrum of compound **40**

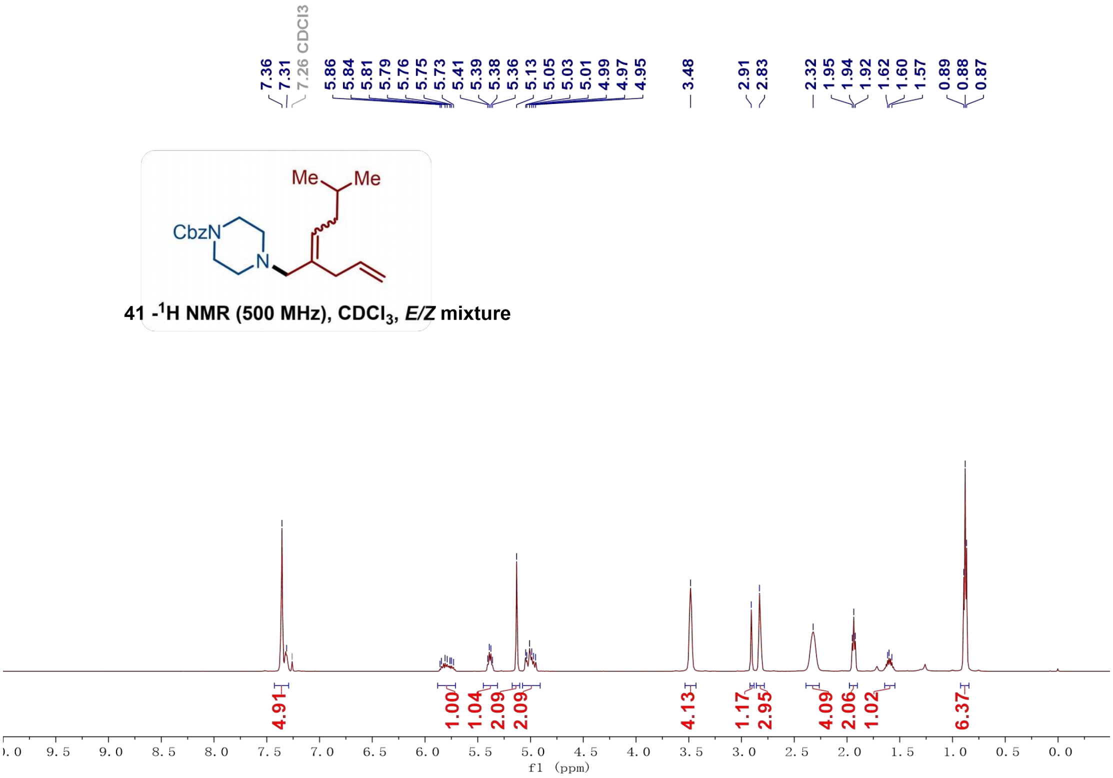

Supplementary Figure 147. ^{1}H NMR (500 MHz, $CDCl_3$) spectrum of compound **41**

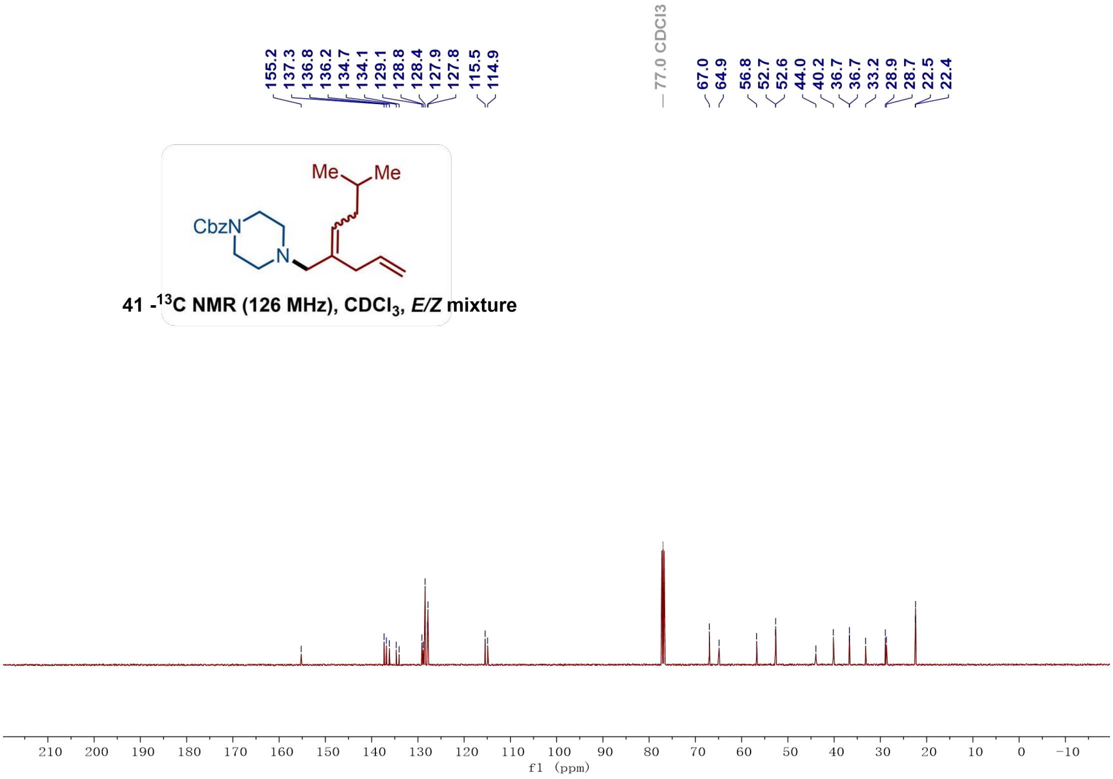

Supplementary Figure 148. ^{13}C NMR (126 MHz, $CDCl_3$) spectrum of compound **41**

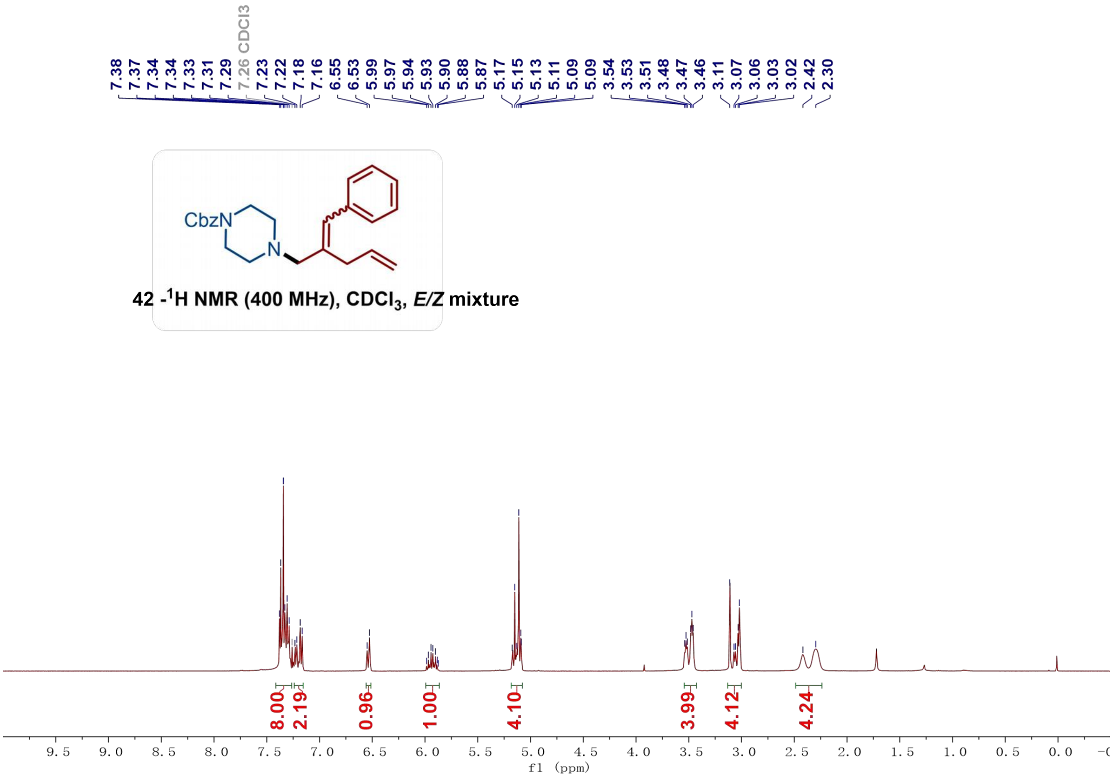

Supplementary Figure 149. ^{1}H NMR (400 MHz, $CDCl_3$) spectrum of compound **42**

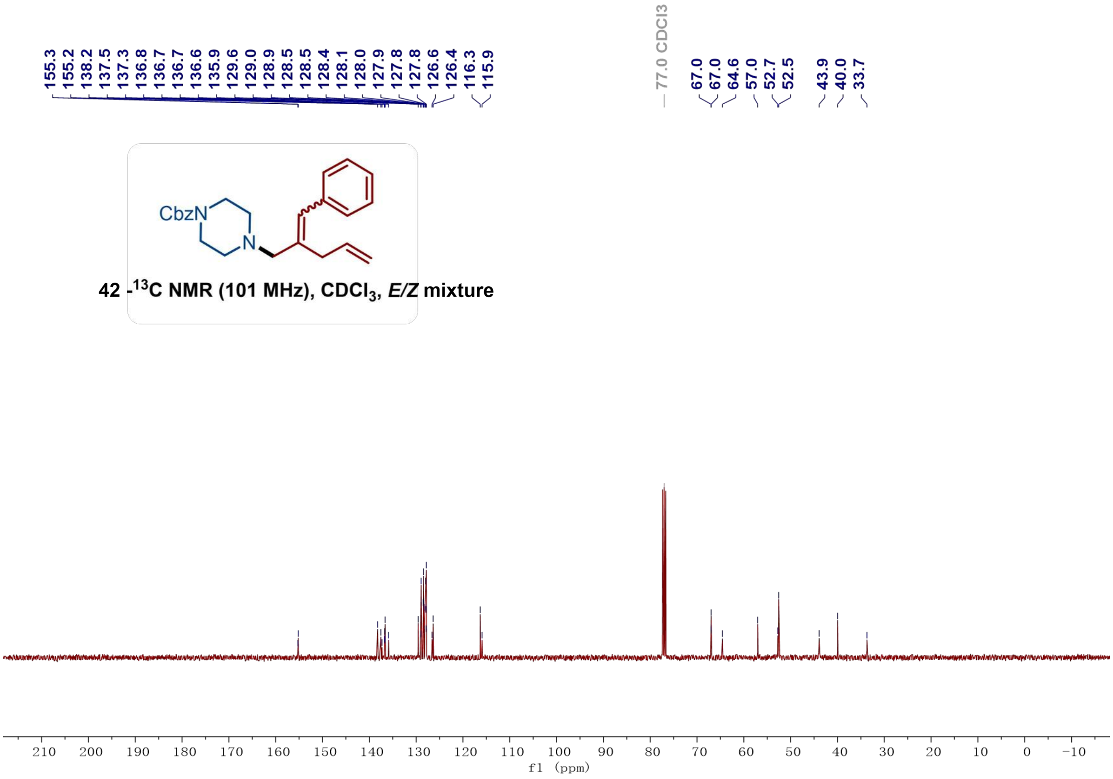

Supplementary Figure 150. ^{13}C NMR (101 MHz, $CDCl_3$) spectrum of compound **42**

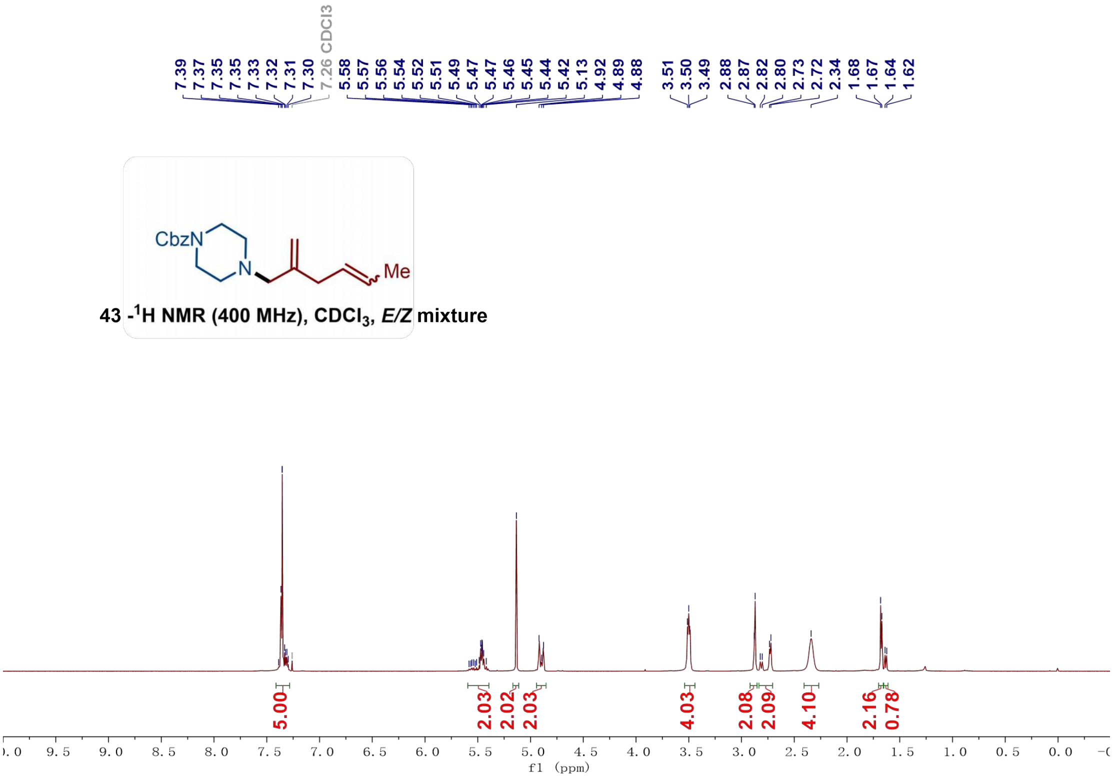

Supplementary Figure 151. ^{1}H NMR (400 MHz, $CDCl_3$) spectrum of compound **43**

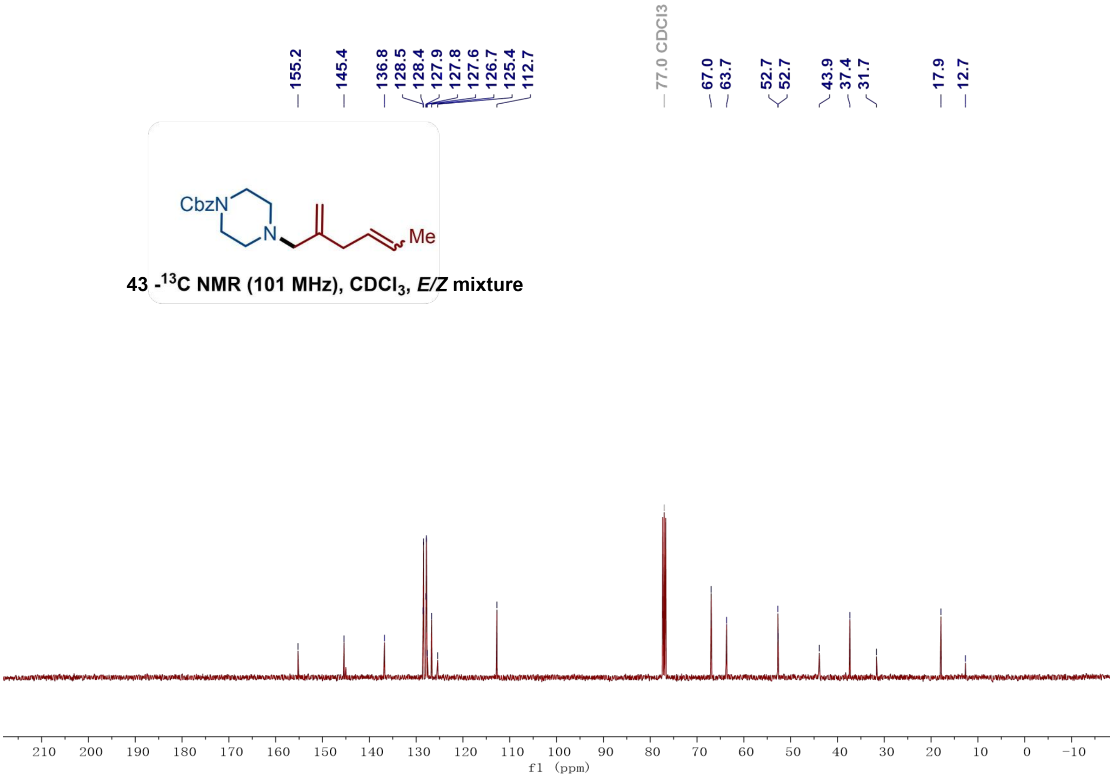

Supplementary Figure 152. ^{13}C NMR (101 MHz, $CDCl_3$) spectrum of compound **43**

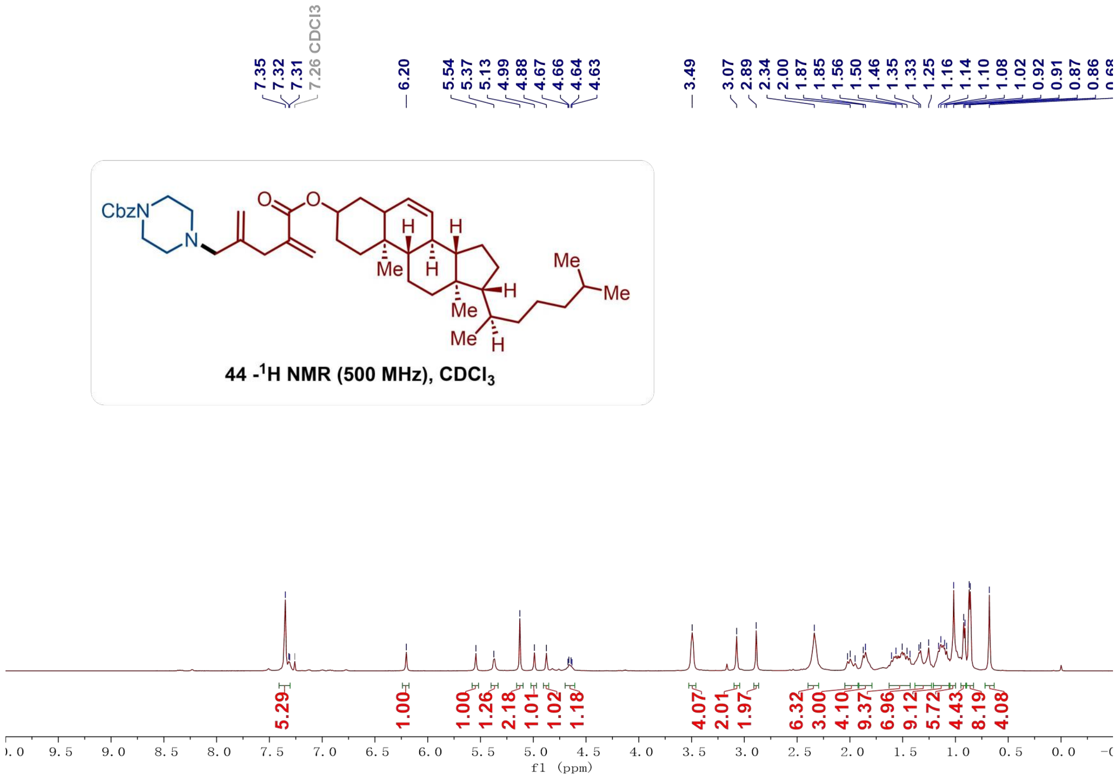

Supplementary Figure 153. ^{1}H NMR (400 MHz, $CDCl_3$) spectrum of compound **44**

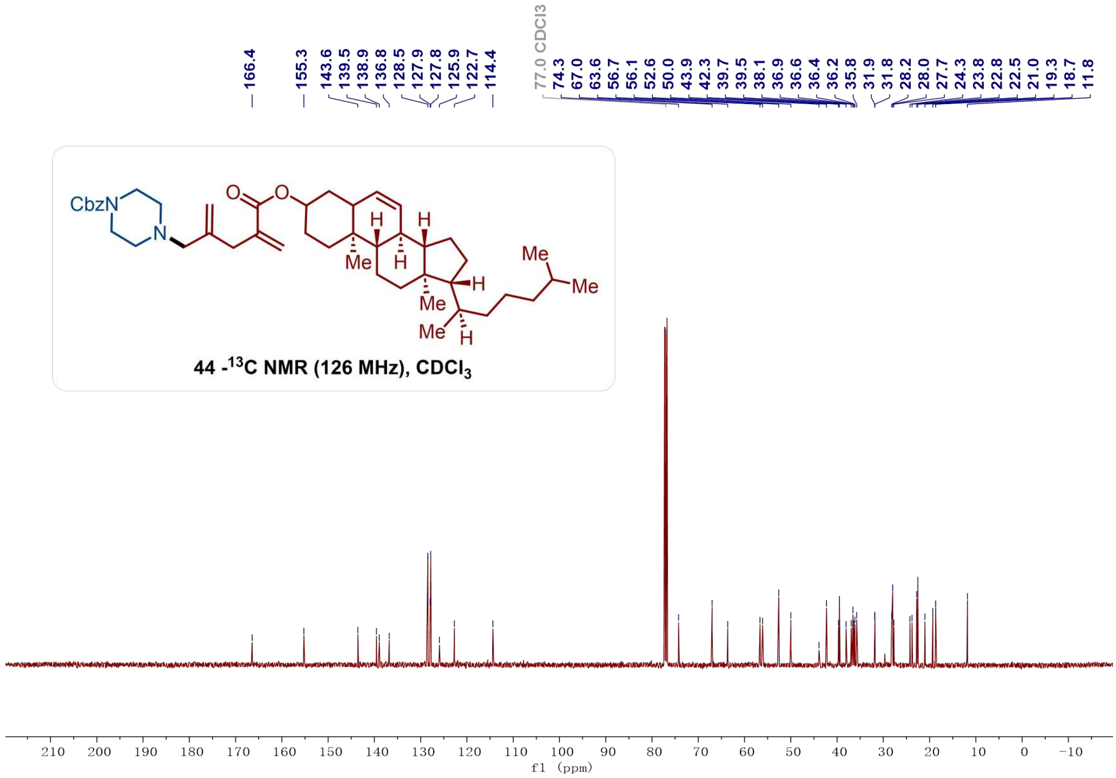

Supplementary Figure 154. ^{13}C NMR (126 MHz, $CDCl_3$) spectrum of compound **44**

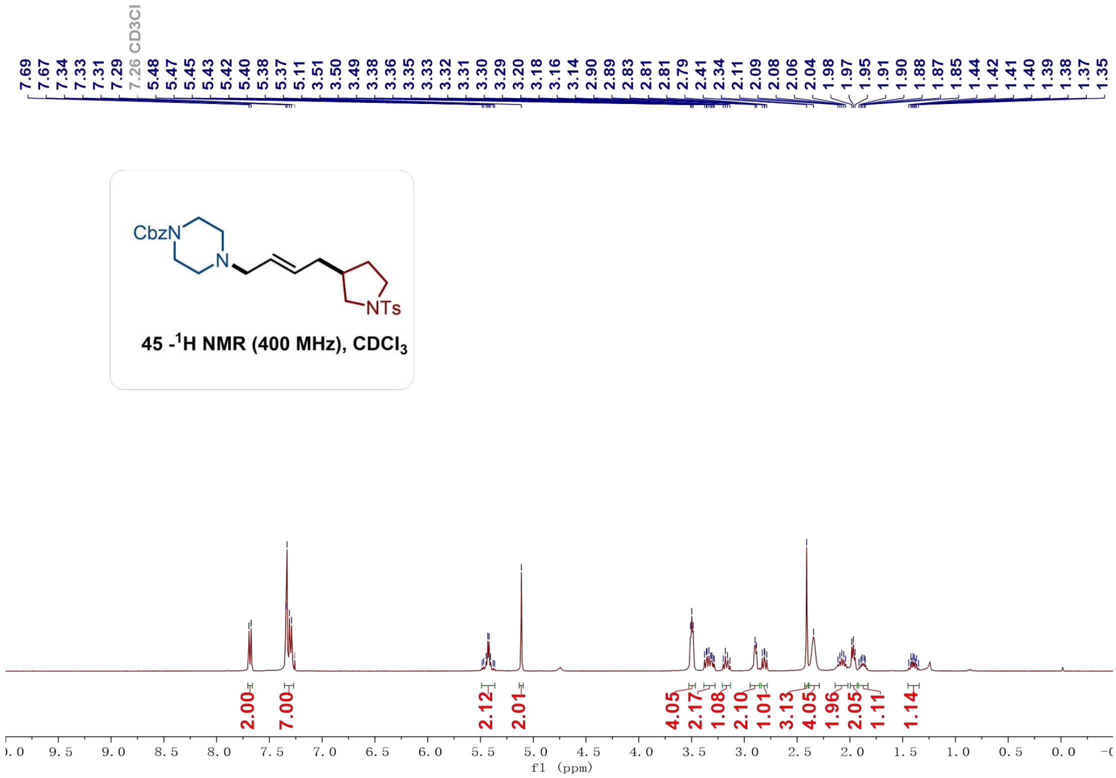

Supplementary Figure 155. ^{1}H NMR (400 MHz, $CDCl_3$) spectrum of compound **45**

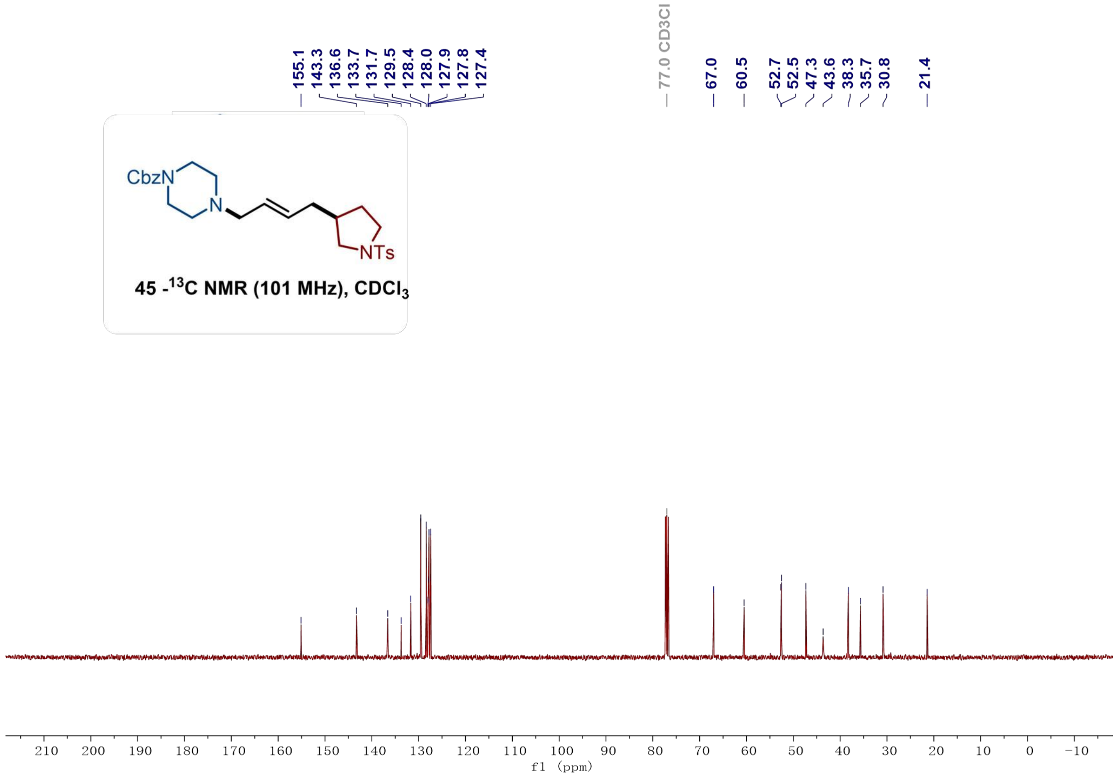

Supplementary Figure 156. ^{13}C NMR (101 MHz, $CDCl_3$) spectrum of compound **45**

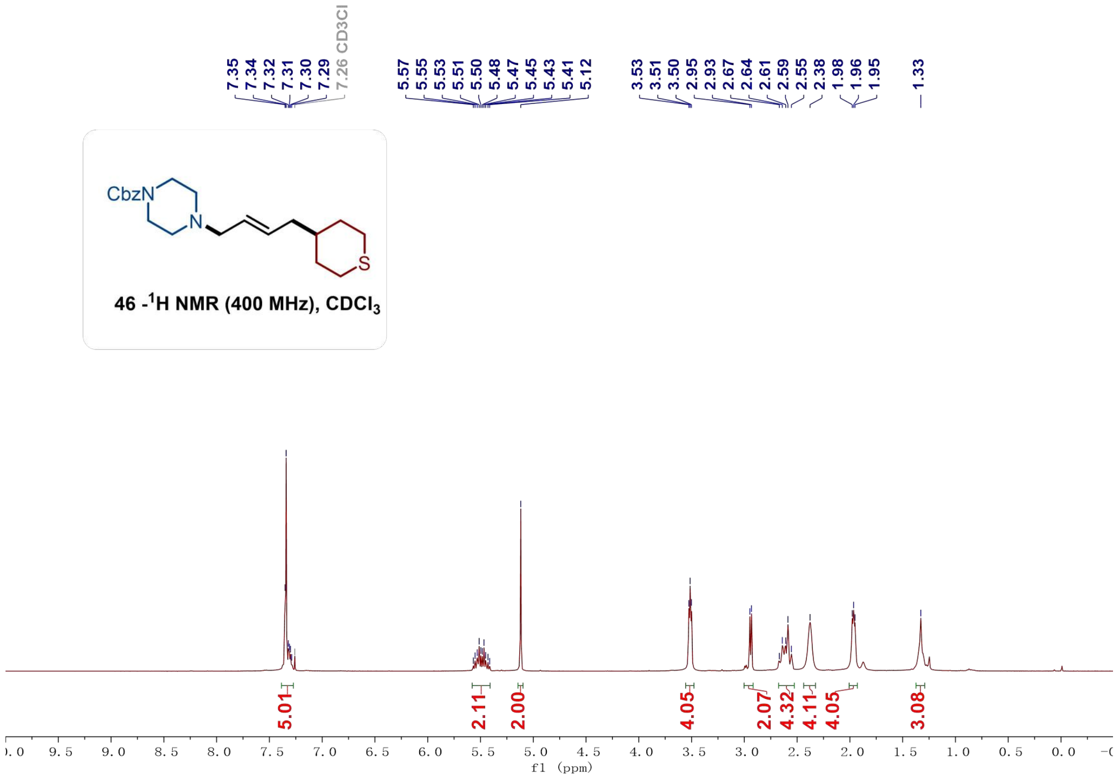

Supplementary Figure 157. ^{1}H NMR (400 MHz, $CDCl_3$) spectrum of compound **46**

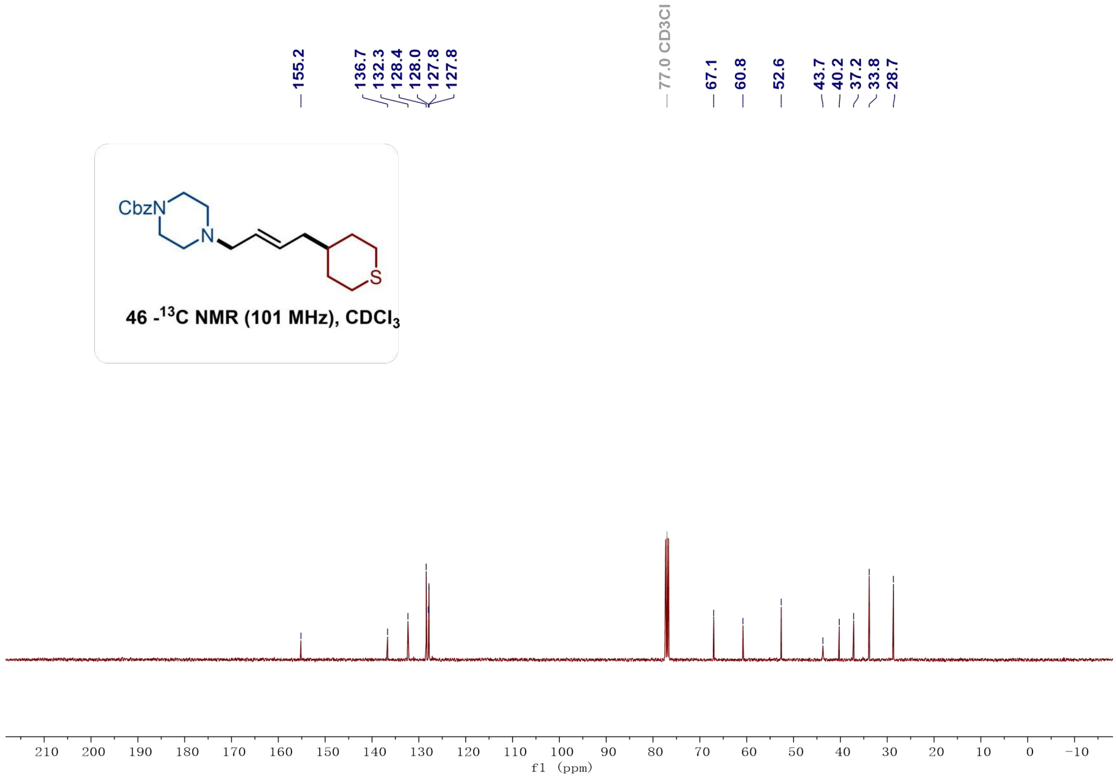

Supplementary Figure 158. ^{13}C NMR (101 MHz, $CDCl_3$) spectrum of compound **46**

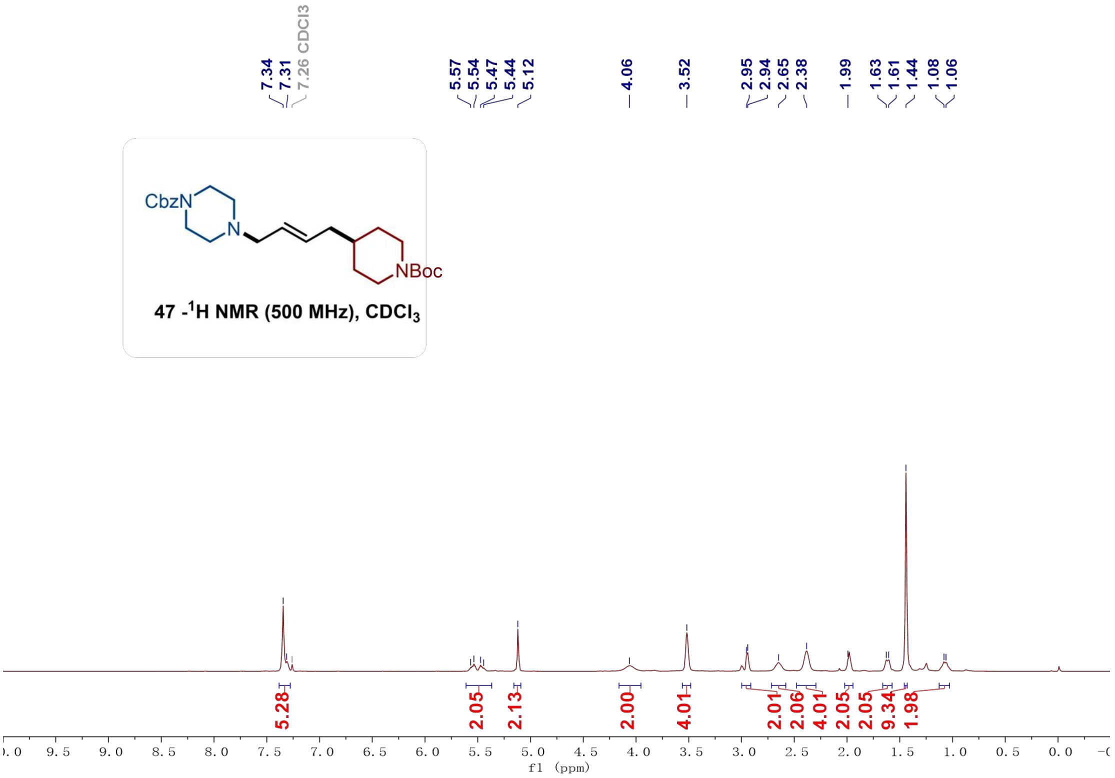

Supplementary Figure 159. ^{1}H NMR (500 MHz, $CDCl_3$) spectrum of compound **47**

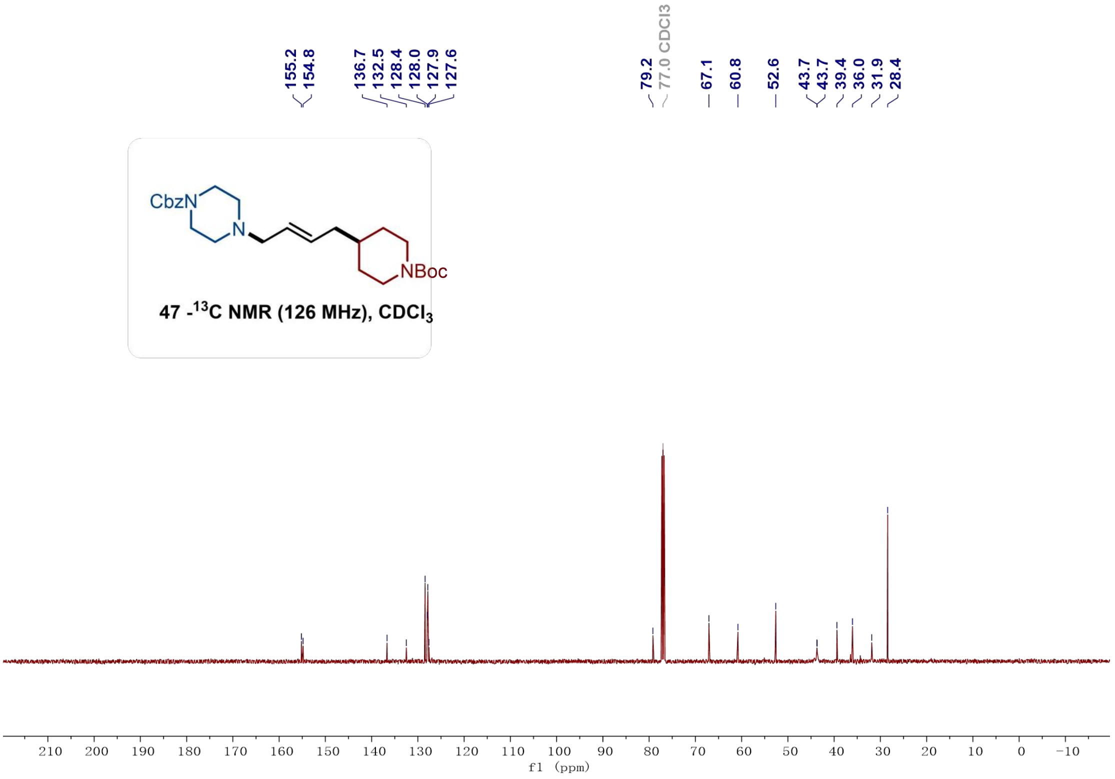

Supplementary Figure 160. ^{13}C NMR (126 MHz, $CDCl_3$) spectrum of compound **47**

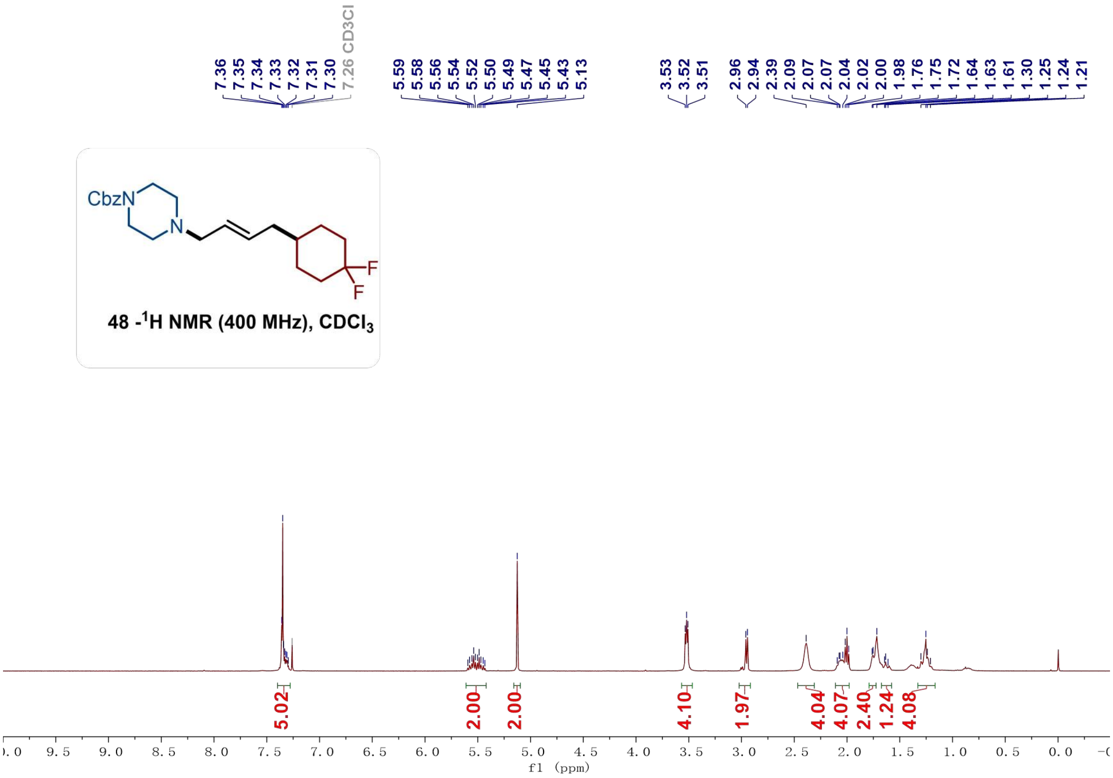

Supplementary Figure 161. ^{1}H NMR (400 MHz, $CDCl_3$) spectrum of compound **48**

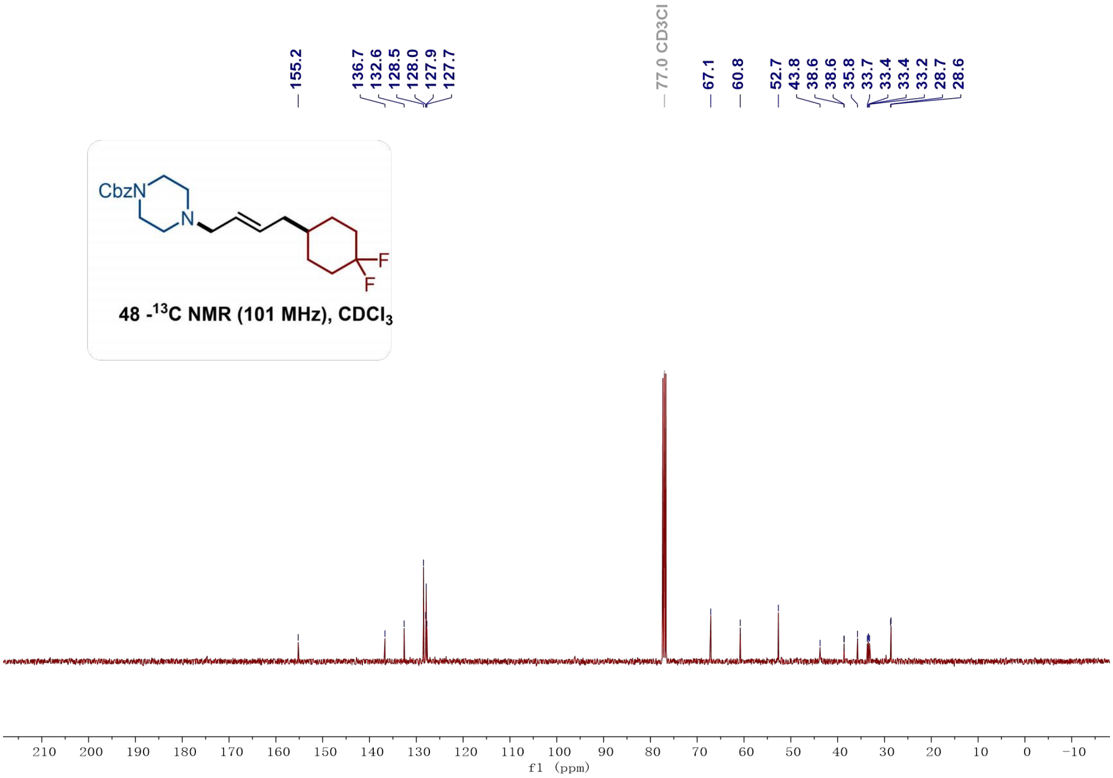

Supplementary Figure 162. ^{13}C NMR (101 MHz, $CDCl_3$) spectrum of compound **48**

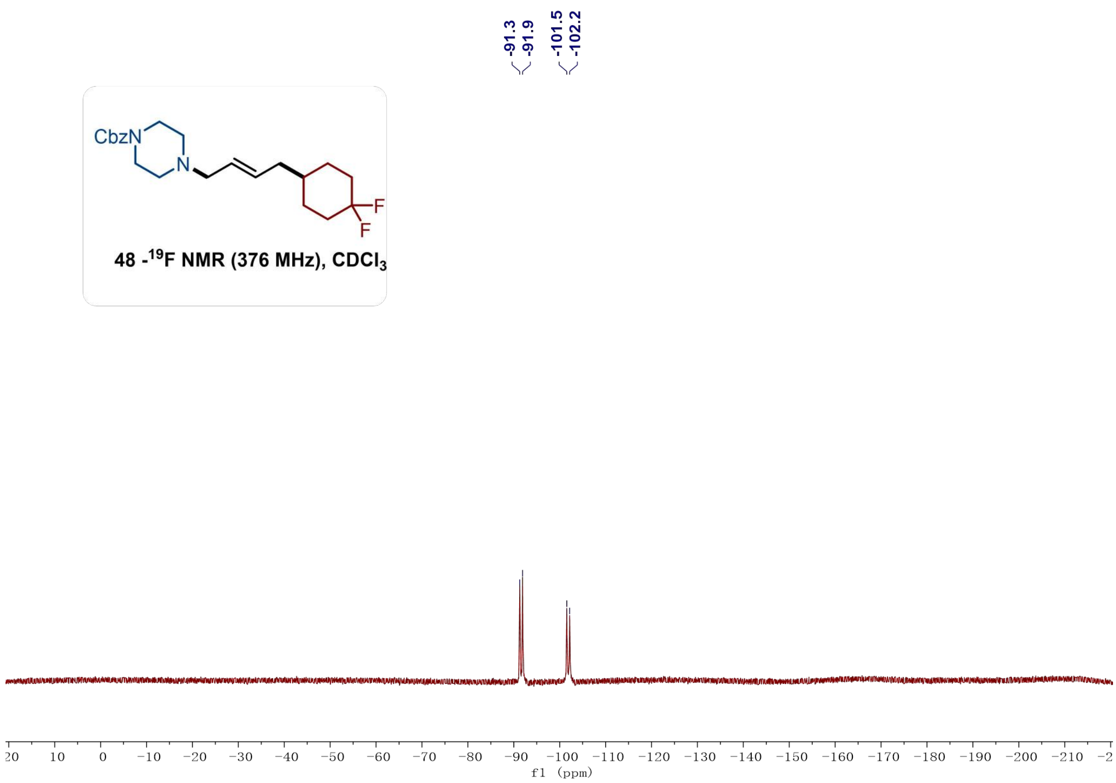

Supplementary Figure 163. ^{19}F NMR (376 MHz, $CDCl_3$) spectrum of compound **48**

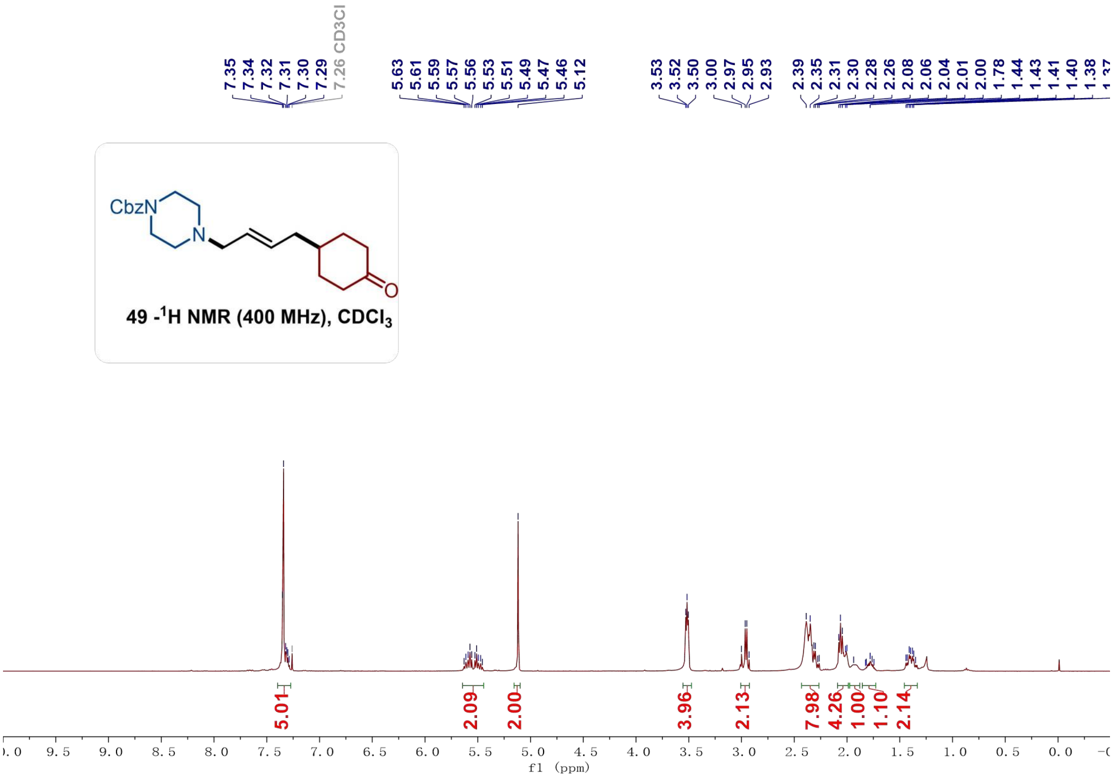

Supplementary Figure 164. ^{1}H NMR (400 MHz, $CDCl_3$) spectrum of compound **49**

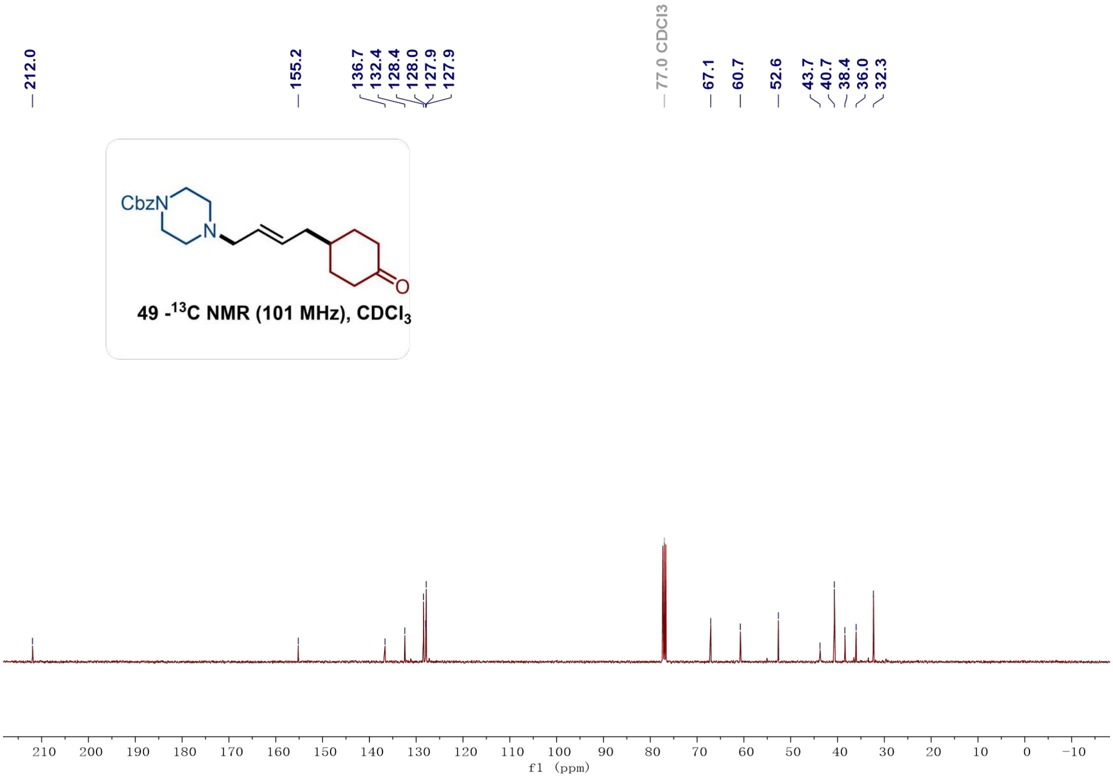

Supplementary Figure 165. ^{13}C NMR (101 MHz, $CDCl_3$) spectrum of compound **49**

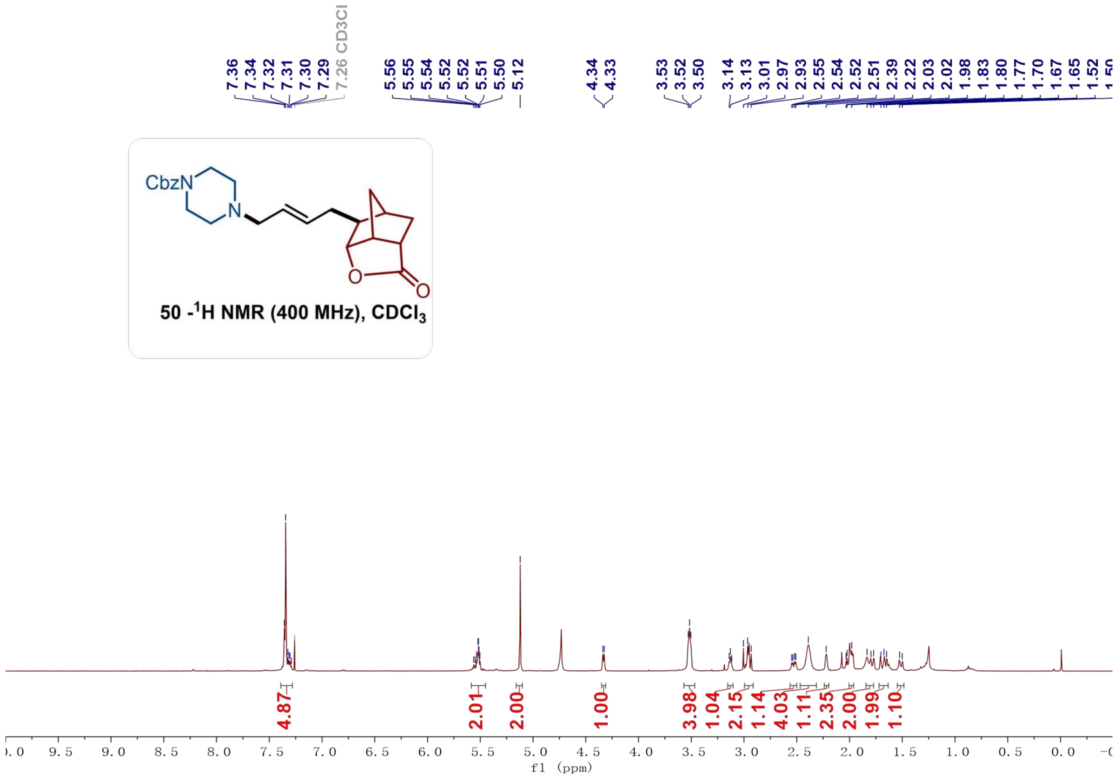

Supplementary Figure 166. ^{1}H NMR (400 MHz, $CDCl_3$) spectrum of compound **50**

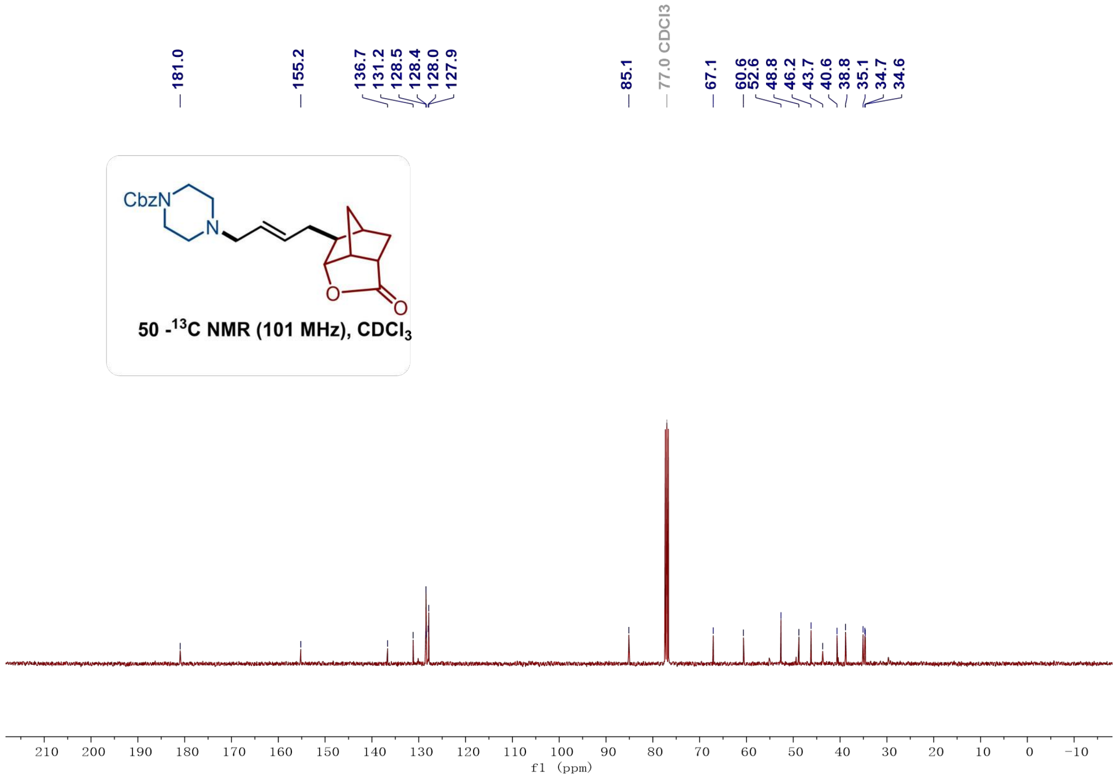

Supplementary Figure 167. ^{13}C NMR (101 MHz, $CDCl_3$) spectrum of compound **50**

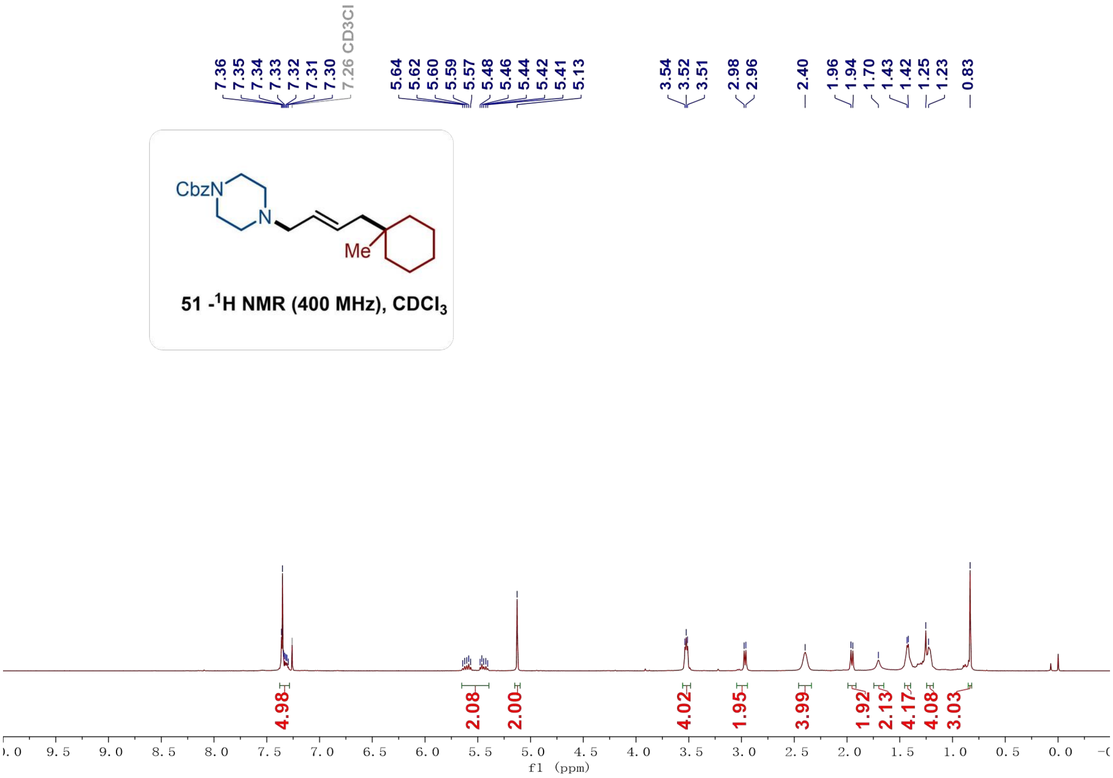

Supplementary Figure 168. ^{1}H NMR (400 MHz, $CDCl_3$) spectrum of compound **51**

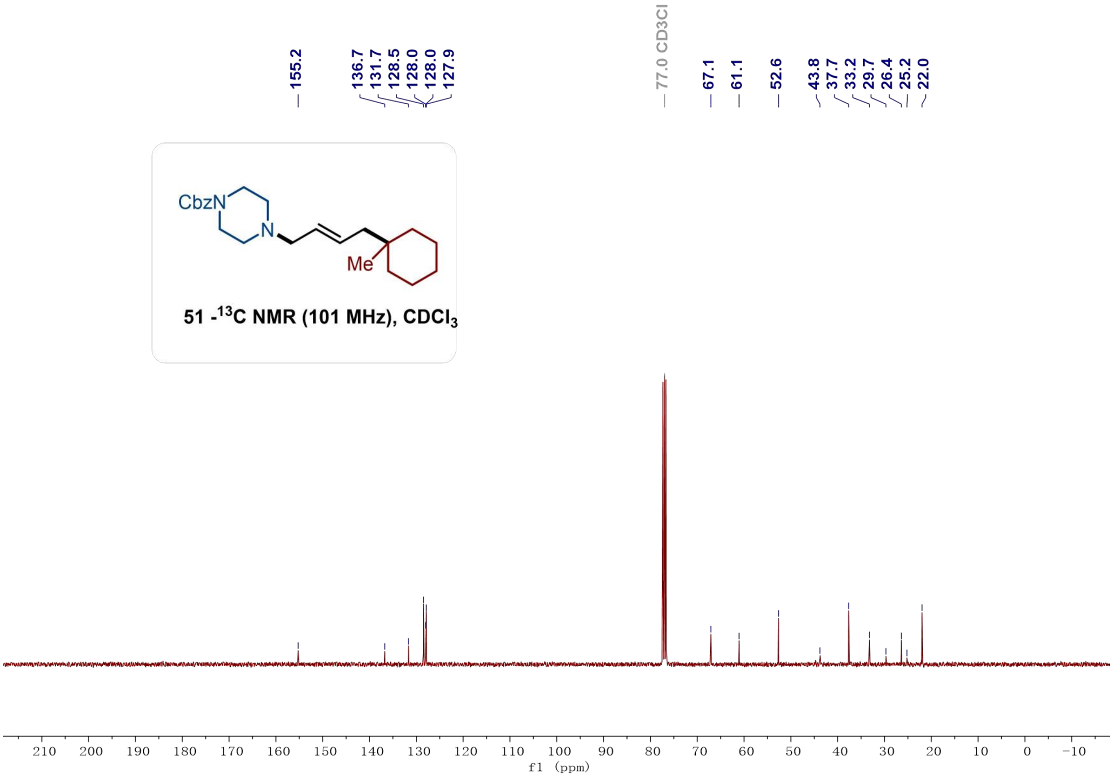

Supplementary Figure 169. ^{13}C NMR (101 MHz, $CDCl_3$) spectrum of compound **51**

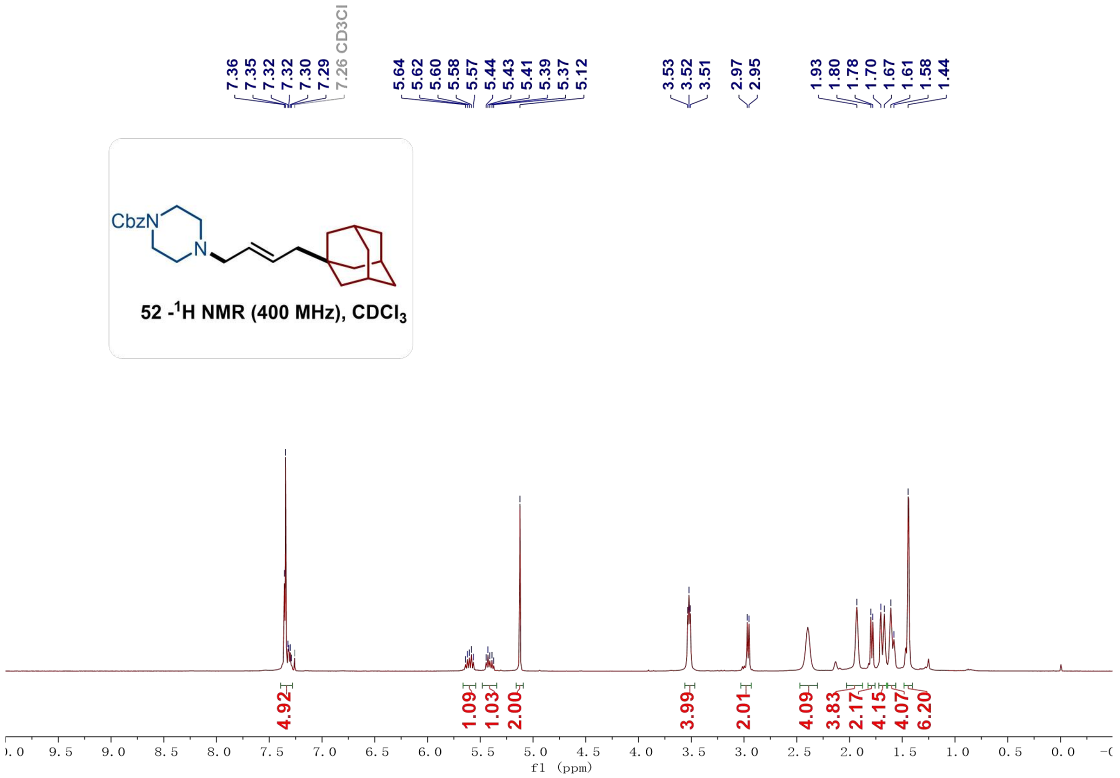

Supplementary Figure 170. ^{1}H NMR (400 MHz, $CDCl_3$) spectrum of compound **52**

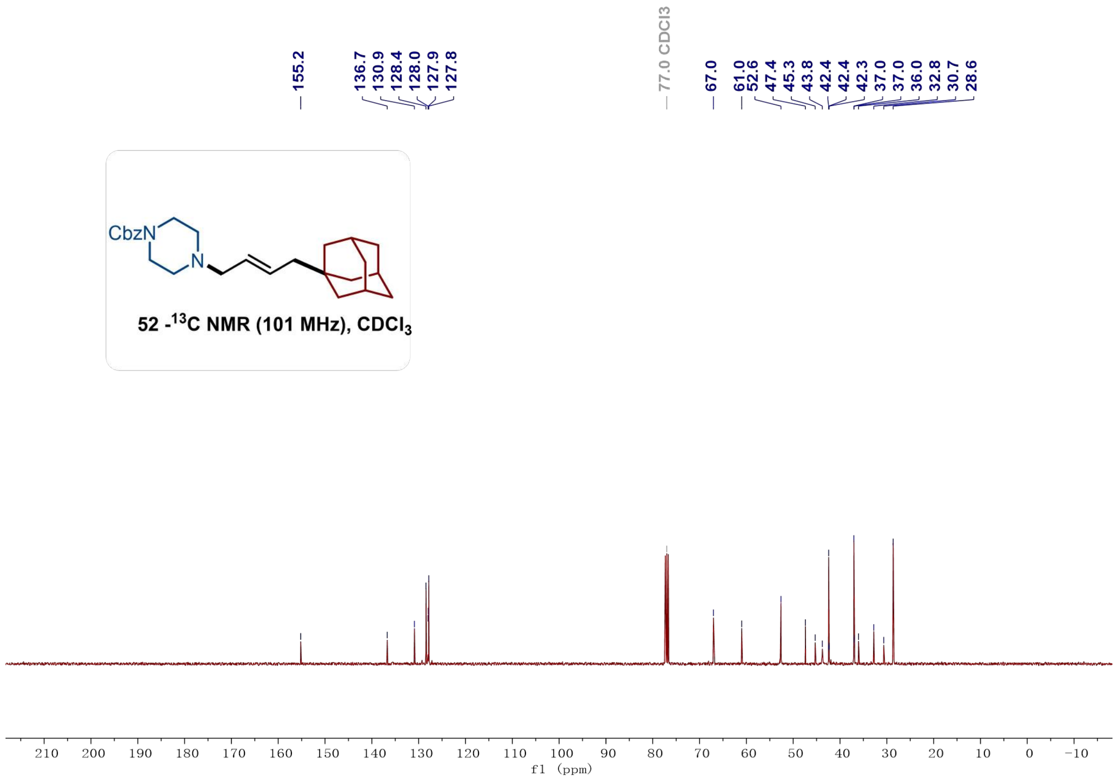

Supplementary Figure 171. ^{13}C NMR (101 MHz, $CDCl_3$) spectrum of compound **52**

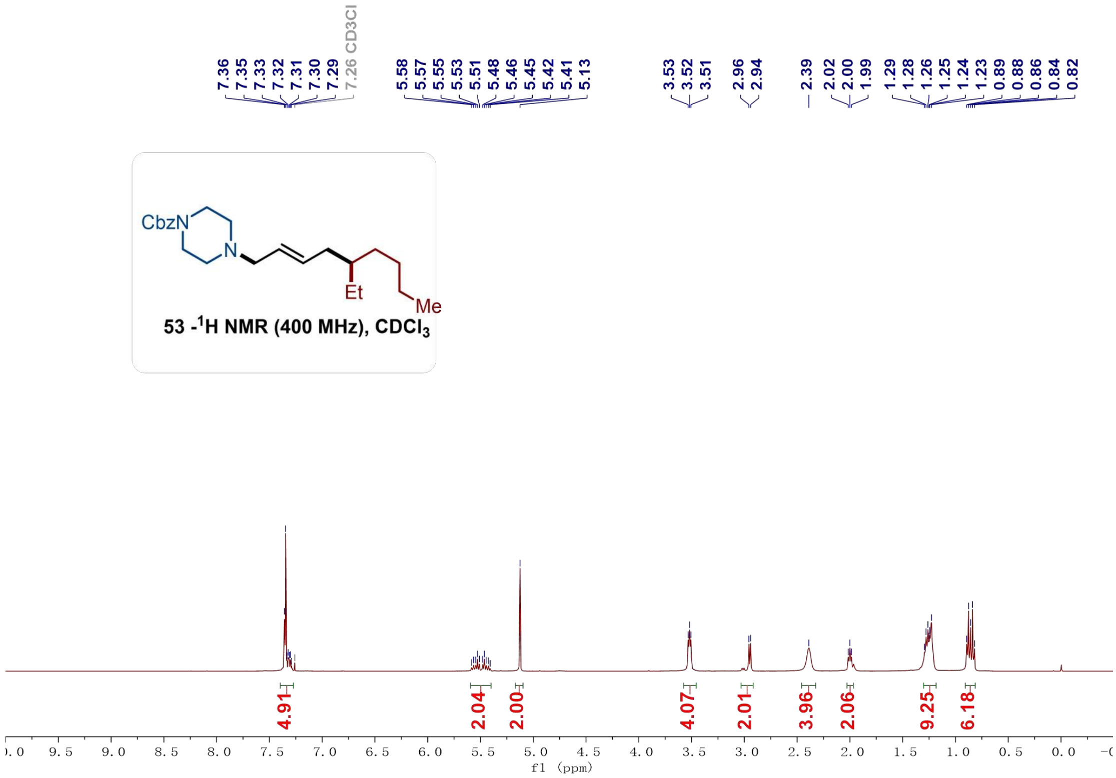

Supplementary Figure 172. ^{1}H NMR (400 MHz, $CDCl_3$) spectrum of compound **53**

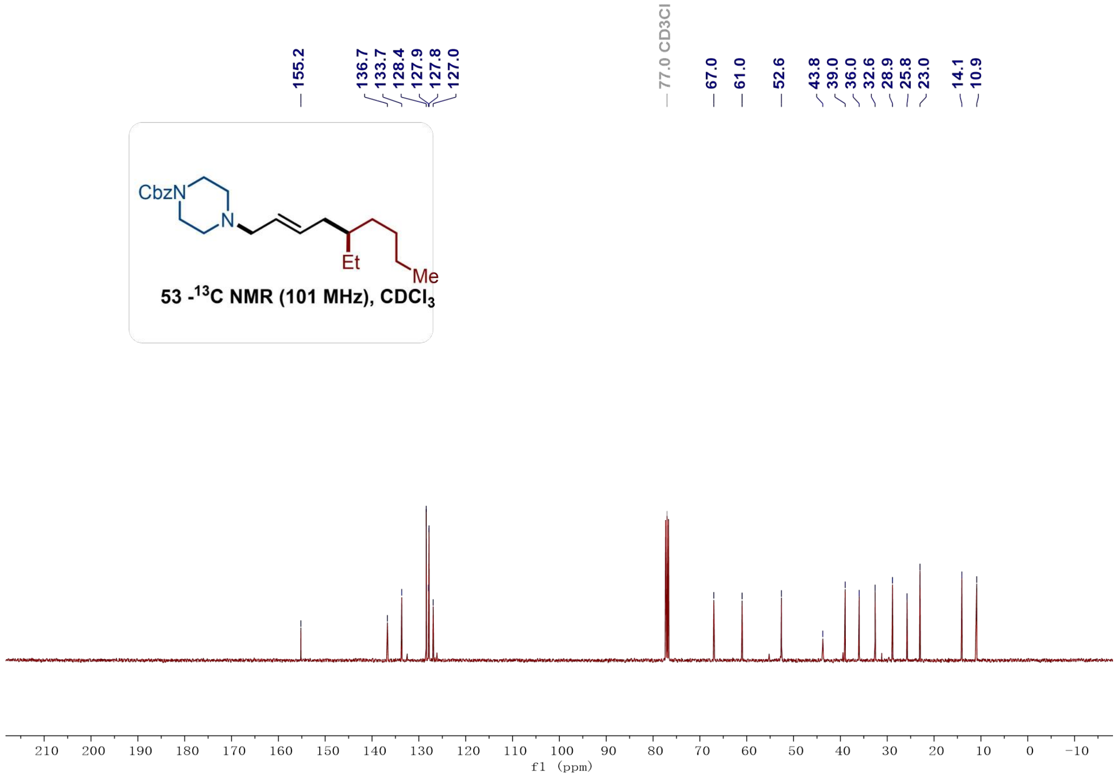

Supplementary Figure 173. ^{13}C NMR (101 MHz, $CDCl_3$) spectrum of compound **53**

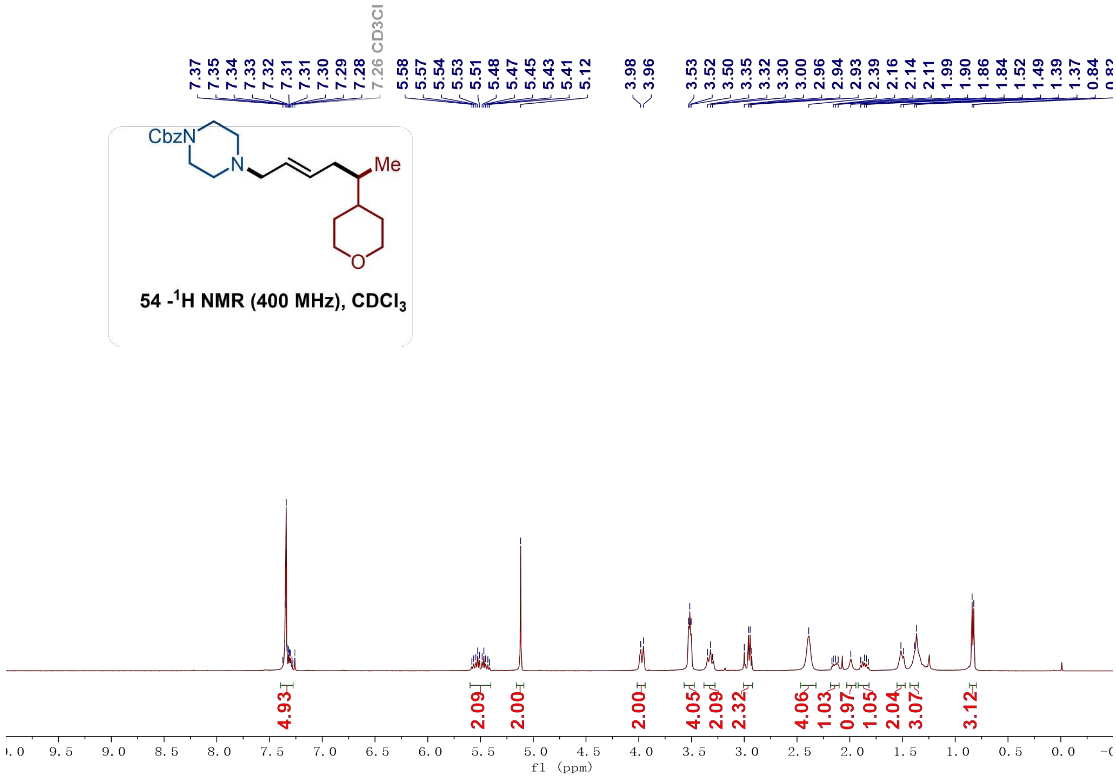

Supplementary Figure 174. ^{1}H NMR (400 MHz, $CDCl_3$) spectrum of compound **54**

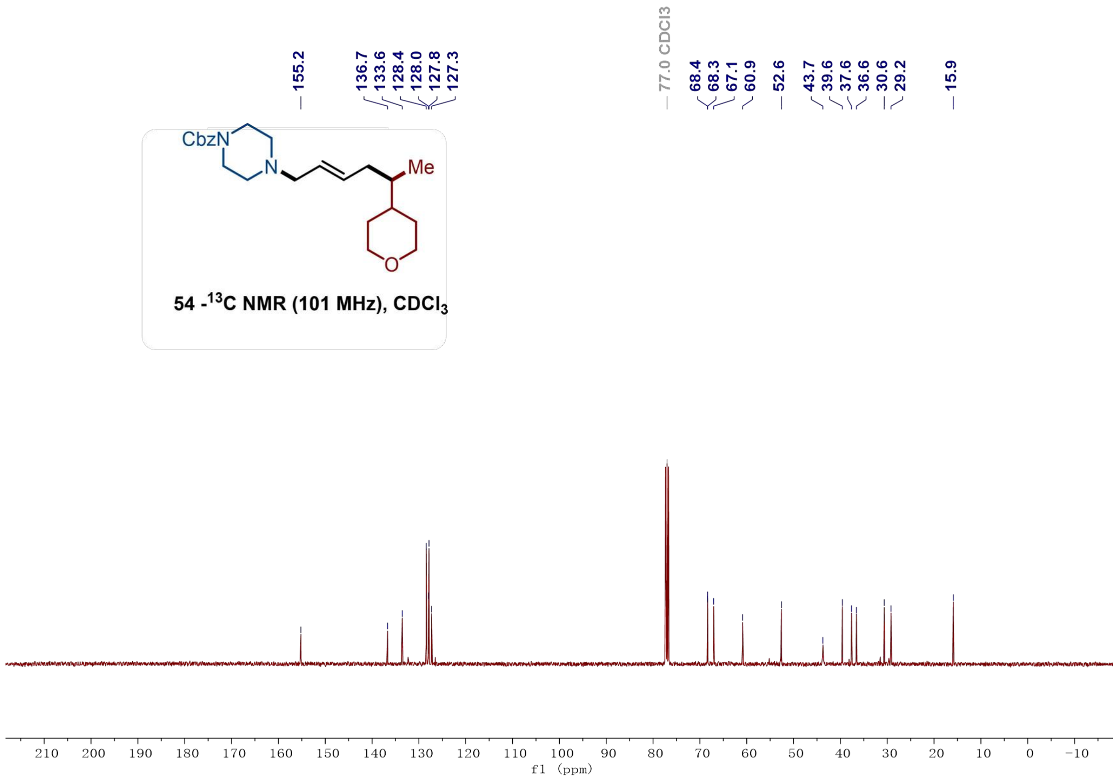

Supplementary Figure 175. ^{13}C NMR (101 MHz, $CDCl_3$) spectrum of compound **54**

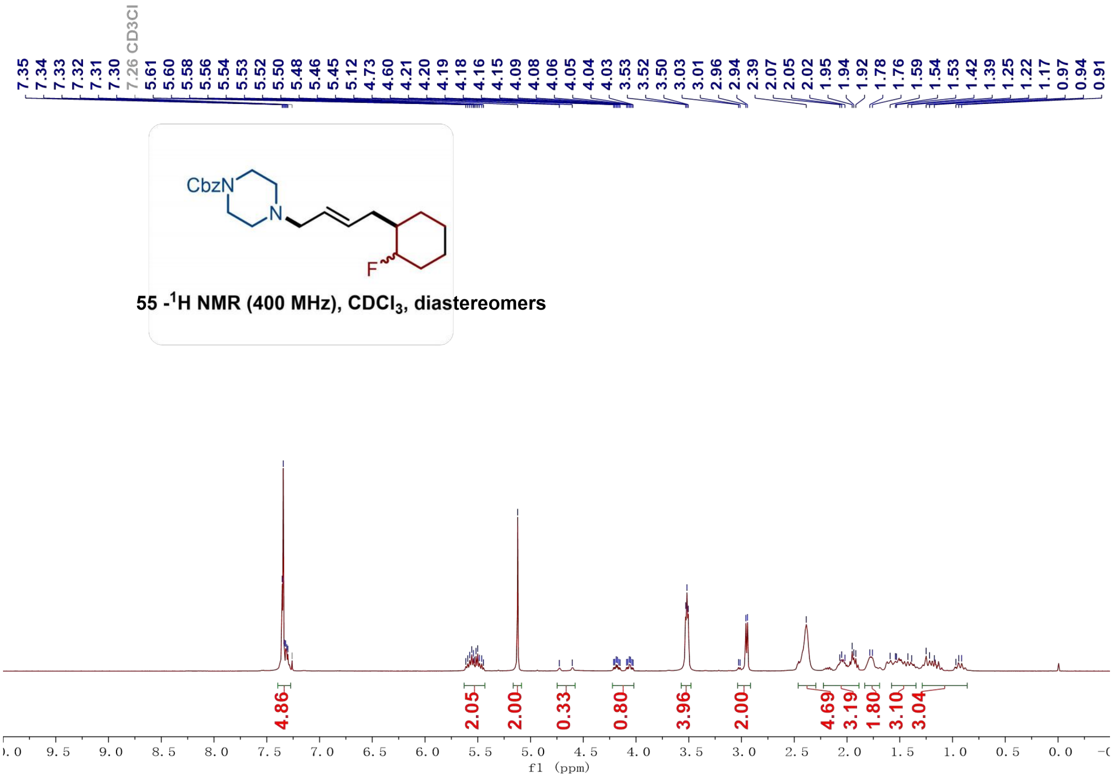

Supplementary Figure 176. ^{1}H NMR (400 MHz, $CDCl_3$) spectrum of compound **55**

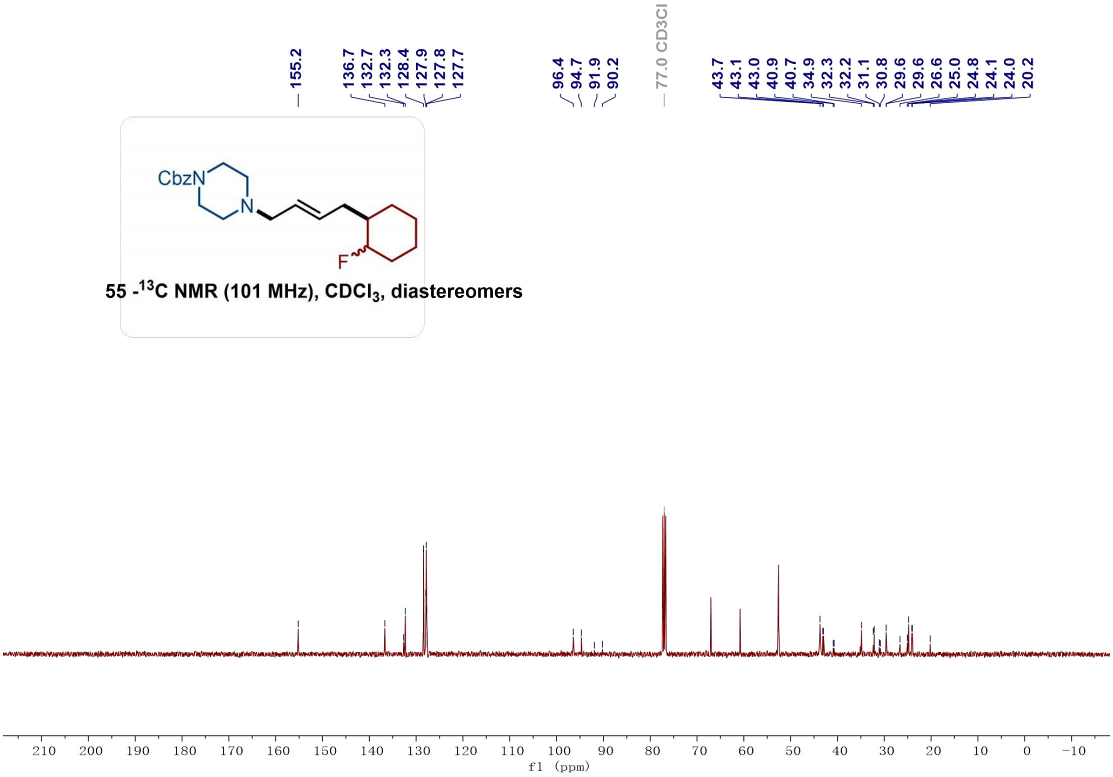

Supplementary Figure 177. ^{13}C NMR (101 MHz, $CDCl_3$) spectrum of compound **55**

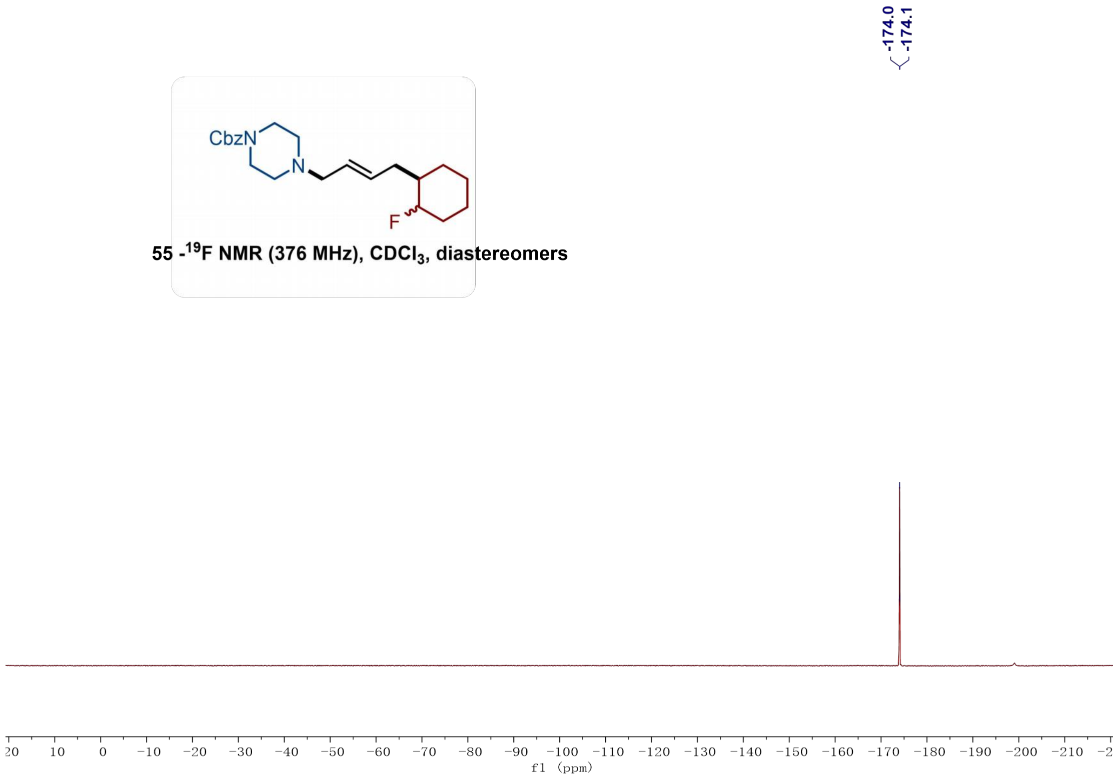

Supplementary Figure 178. ^{19}F NMR (376 MHz, $CDCl_3$) spectrum of compound **55**

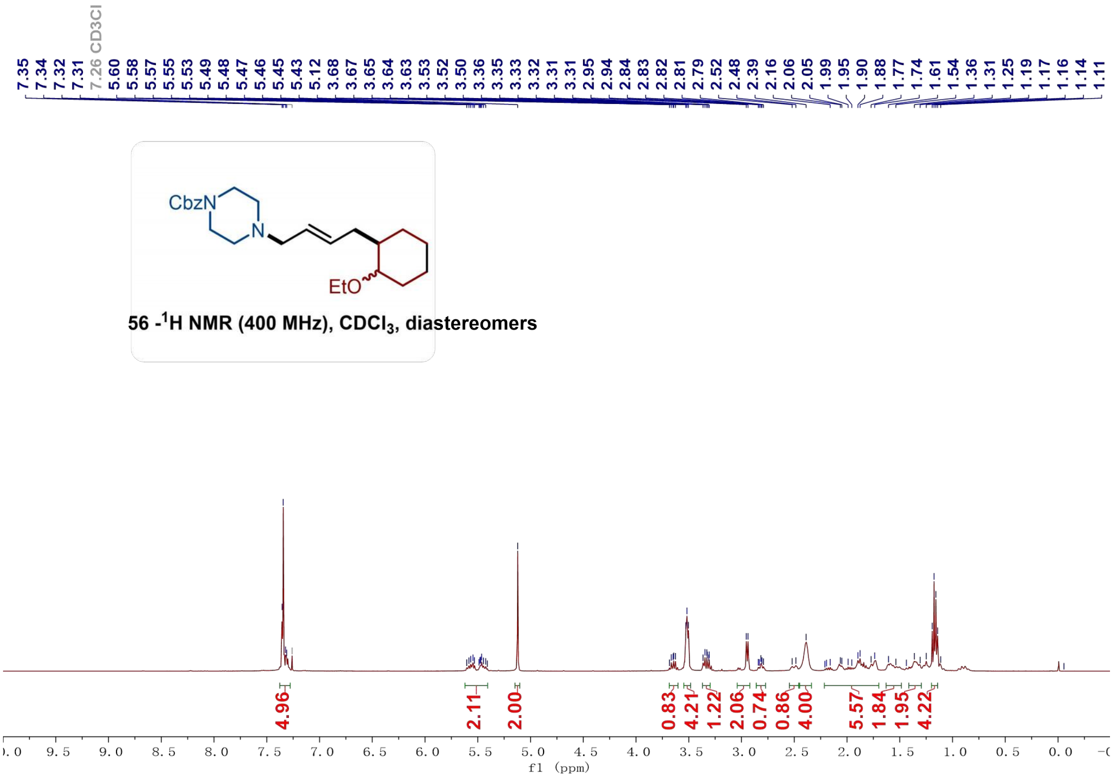

Supplementary Figure 179. ^{1}H NMR (400 MHz, $CDCl_3$) spectrum of compound **56**

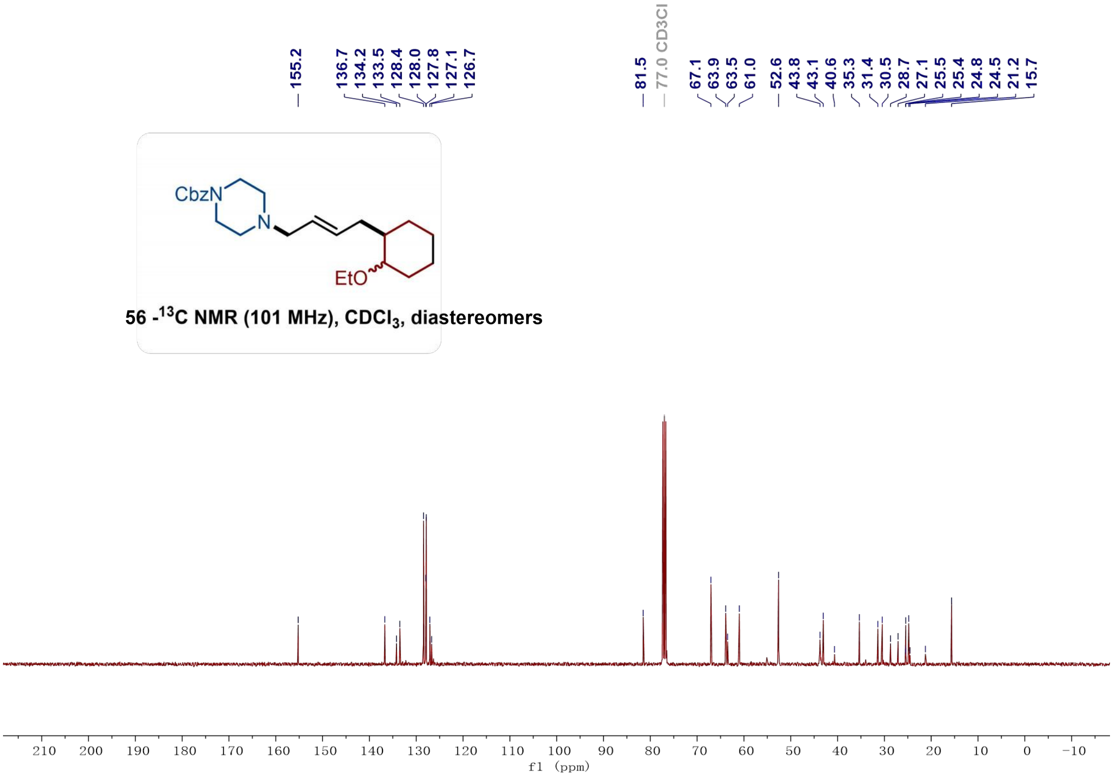

Supplementary Figure 180. ^{13}C NMR (101 MHz, $CDCl_3$) spectrum of compound **56**

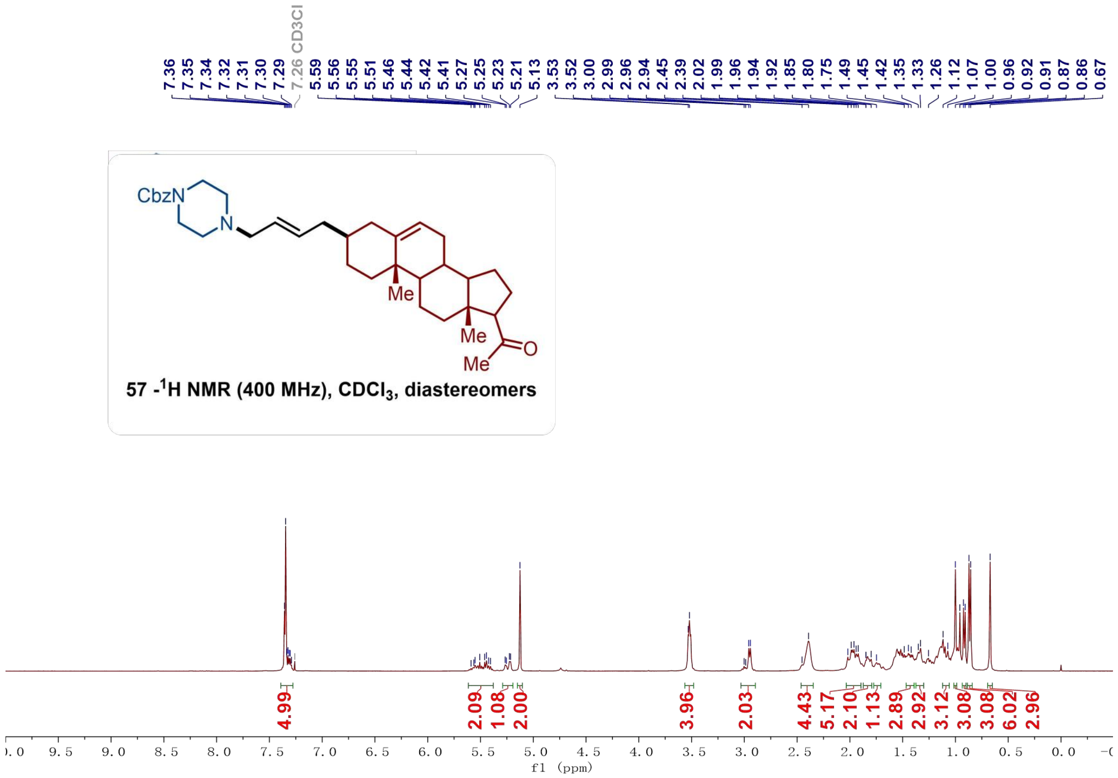

Supplementary Figure 181. ^{1}H NMR (400 MHz, $CDCl_3$) spectrum of compound **57**

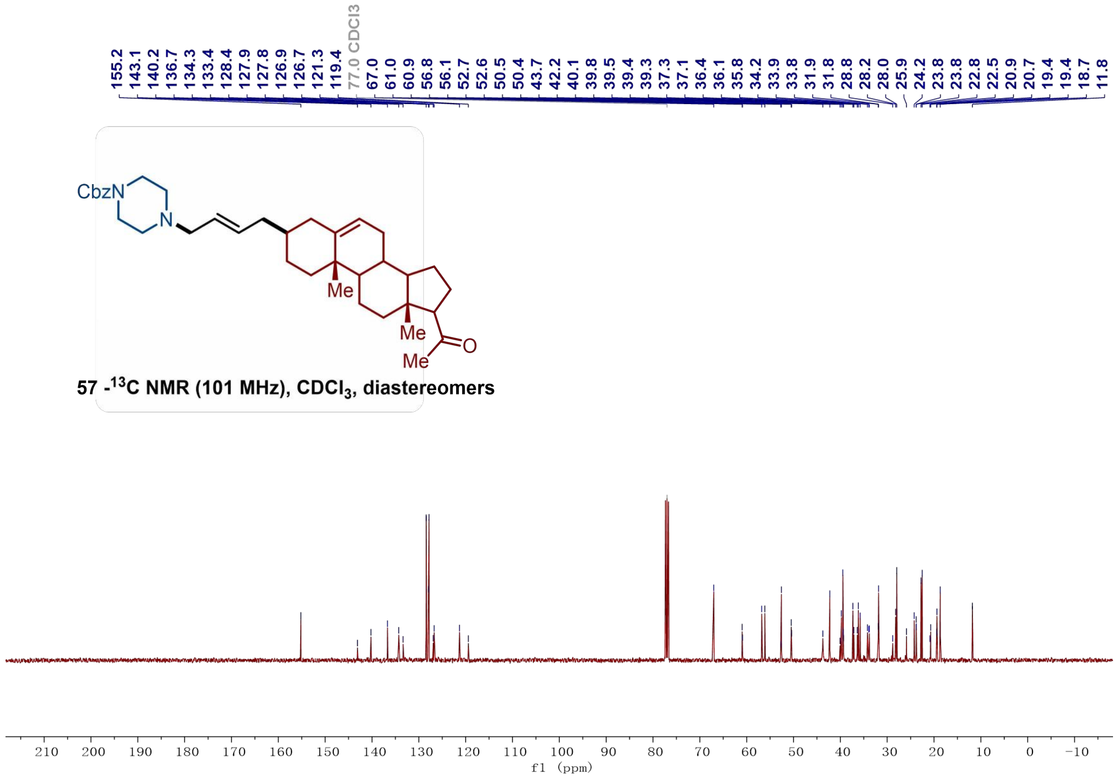

Supplementary Figure 182. ^{13}C NMR (101 MHz, $CDCl_3$) spectrum of compound **57**

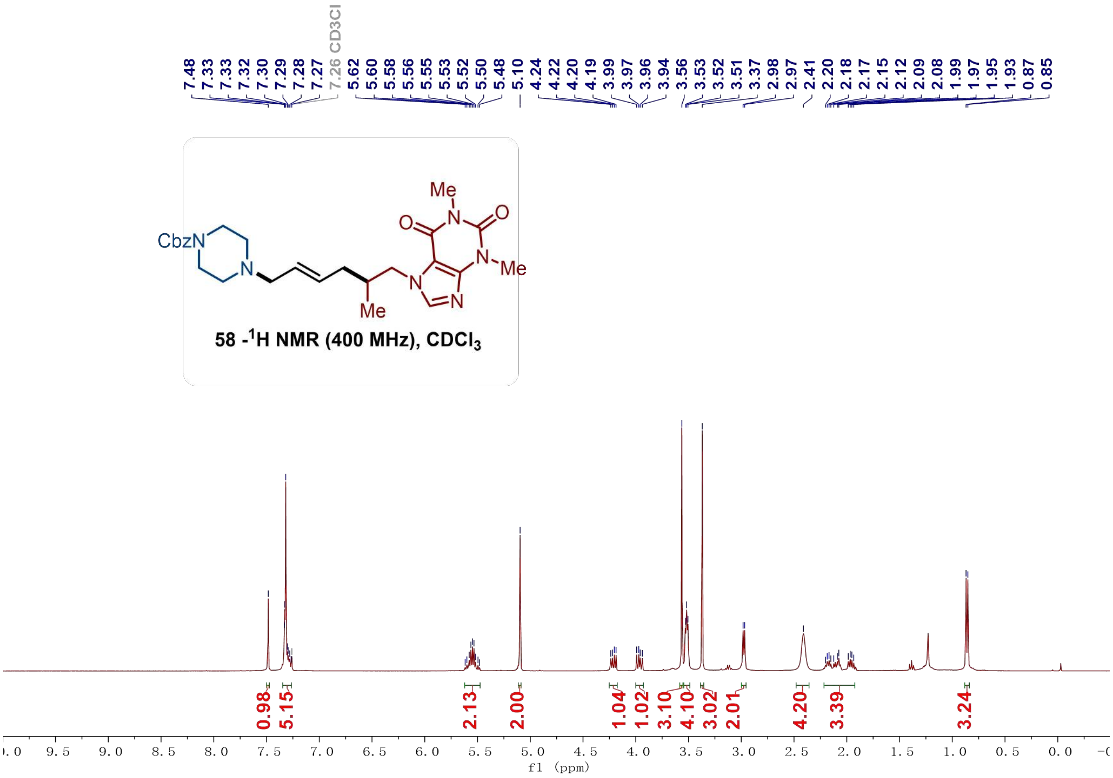

Supplementary Figure 183. ^{1}H NMR (400 MHz, $CDCl_3$) spectrum of compound **58**

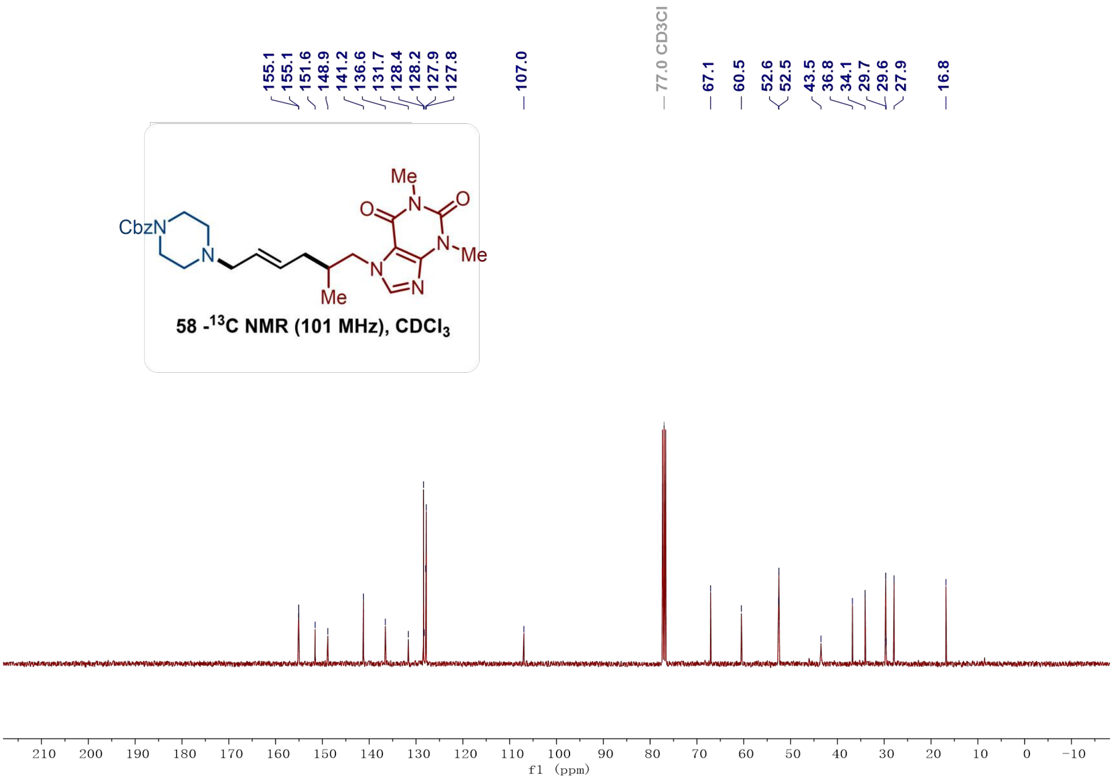

Supplementary Figure 184. ^{13}C NMR (101 MHz, $CDCl_3$) spectrum of compound **58**

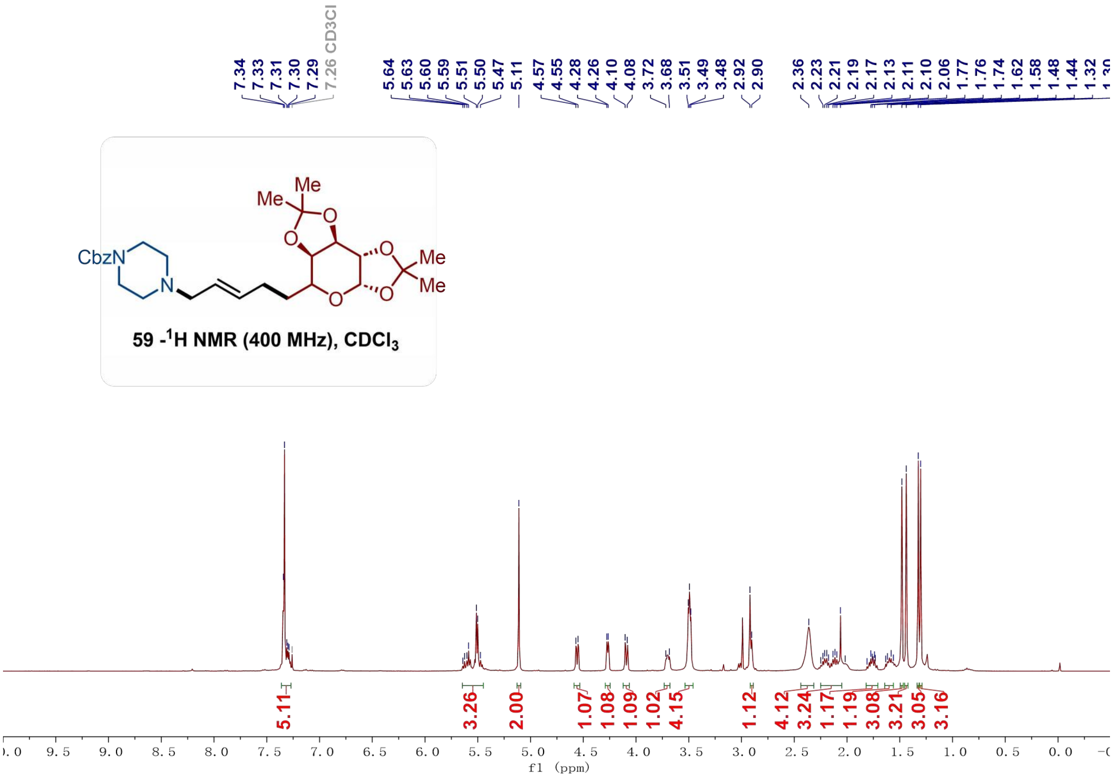

Supplementary Figure 185. ^{1}H NMR (400 MHz, $CDCl_3$) spectrum of compound **59**

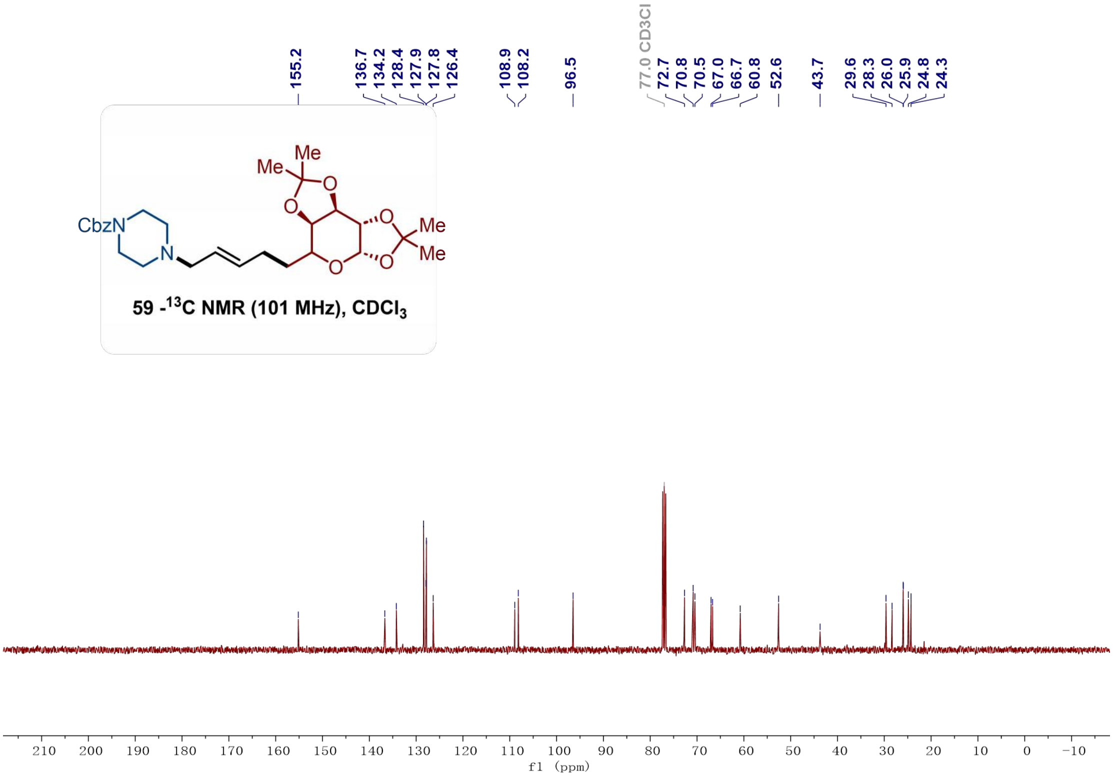

Supplementary Figure 186. ^{13}C NMR (101 MHz, $CDCl_3$) spectrum of compound **59**

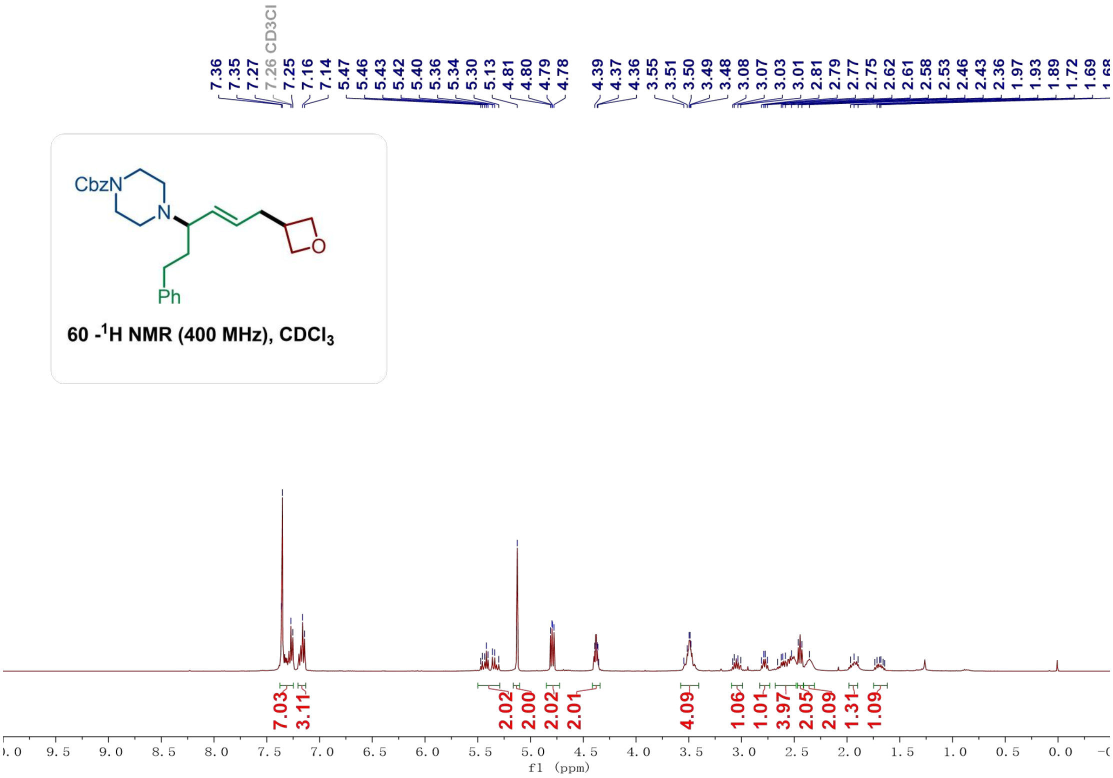

Supplementary Figure 187. ^{1}H NMR (400 MHz, $CDCl_3$) spectrum of compound **60**

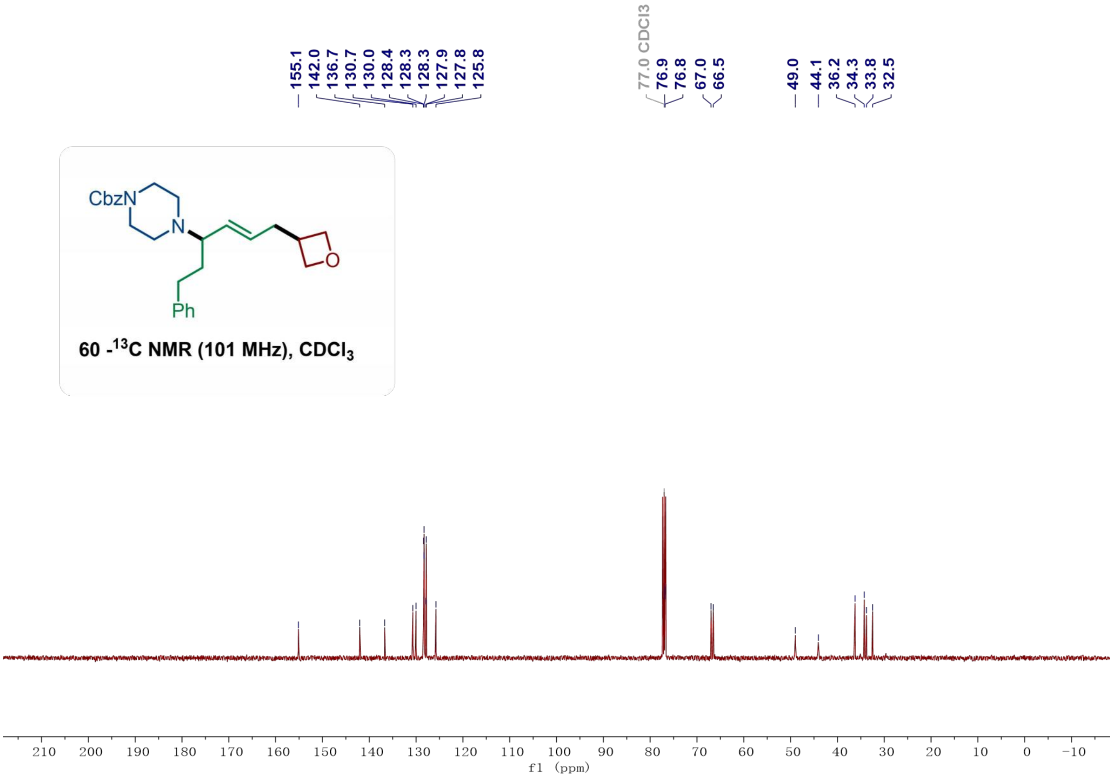

Supplementary Figure 188. ^{13}C NMR (101 MHz, $CDCl_3$) spectrum of compound **60**

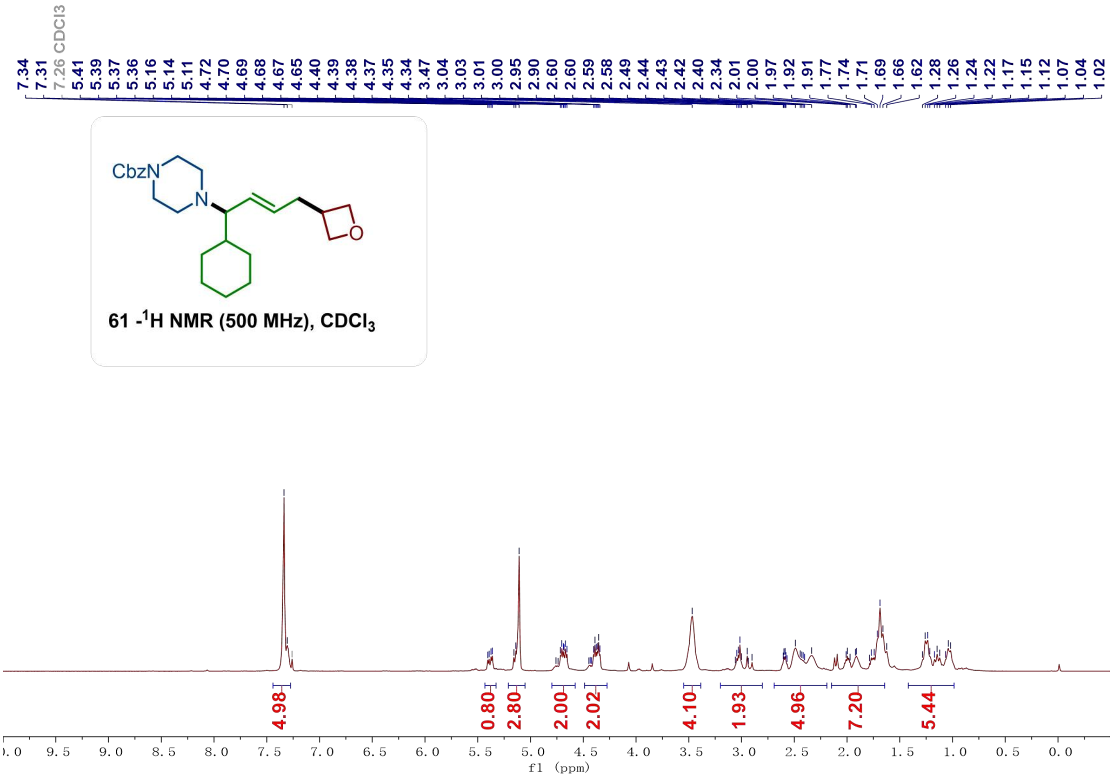

Supplementary Figure 189. ^{1}H NMR (500 MHz, $CDCl_3$) spectrum of compound **61**

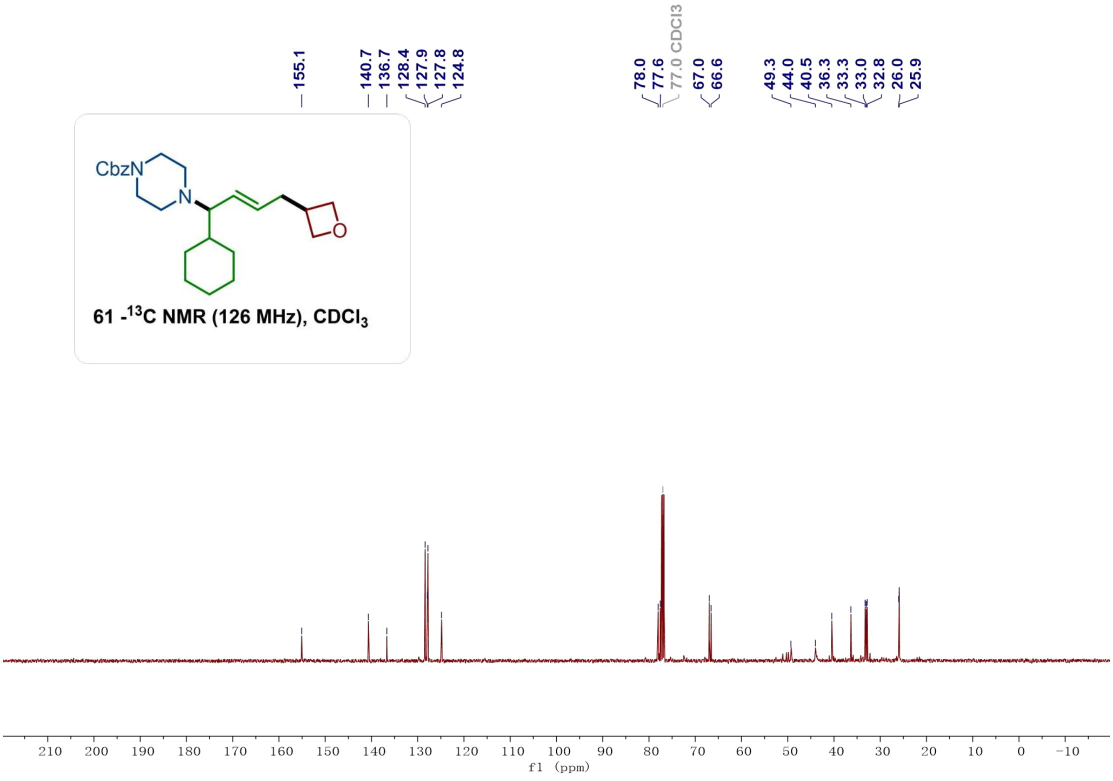

Supplementary Figure 190. ^{13}C NMR (126 MHz, $CDCl_3$) spectrum of compound **61**

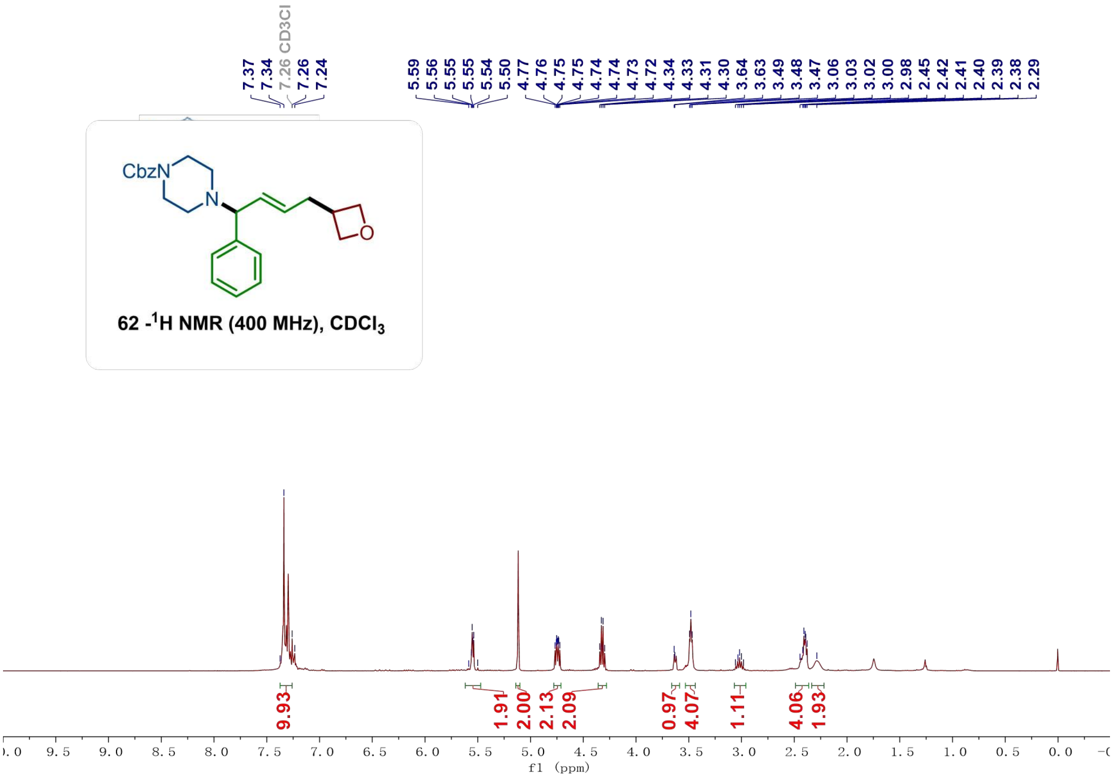

Supplementary Figure 191. ^{1}H NMR (400 MHz, $CDCl_3$) spectrum of compound **62**

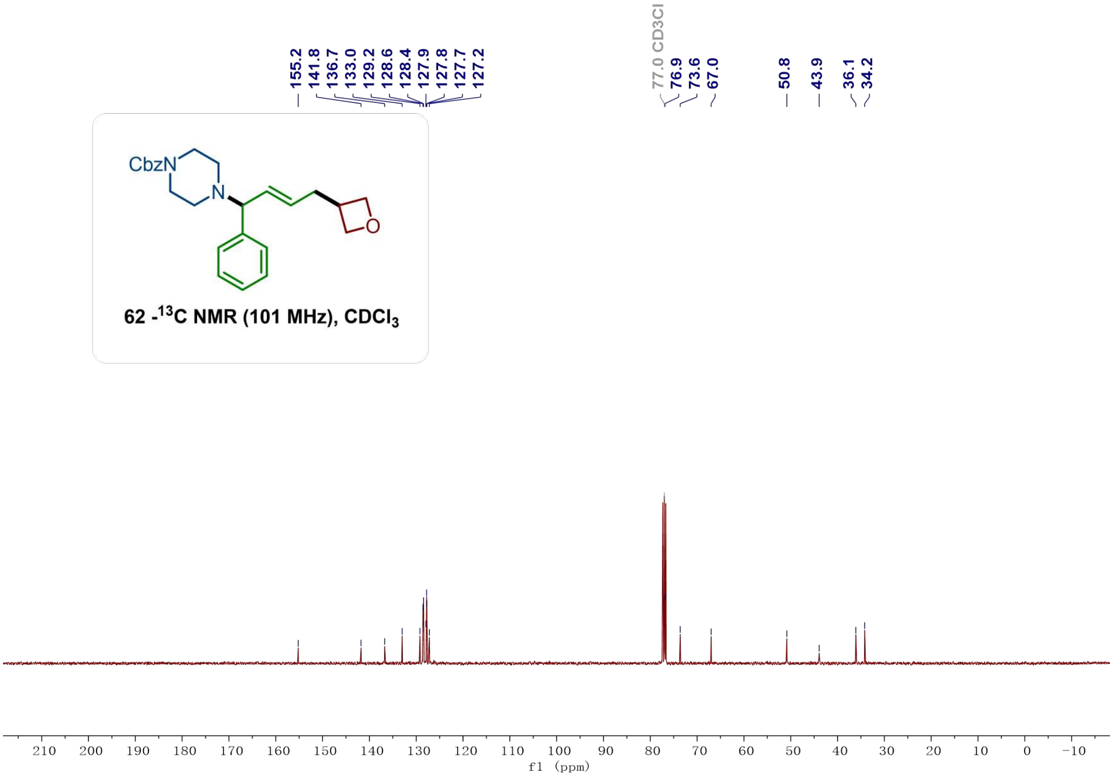

Supplementary Figure 192. ^{13}C NMR (101 MHz, $CDCl_3$) spectrum of compound **62**

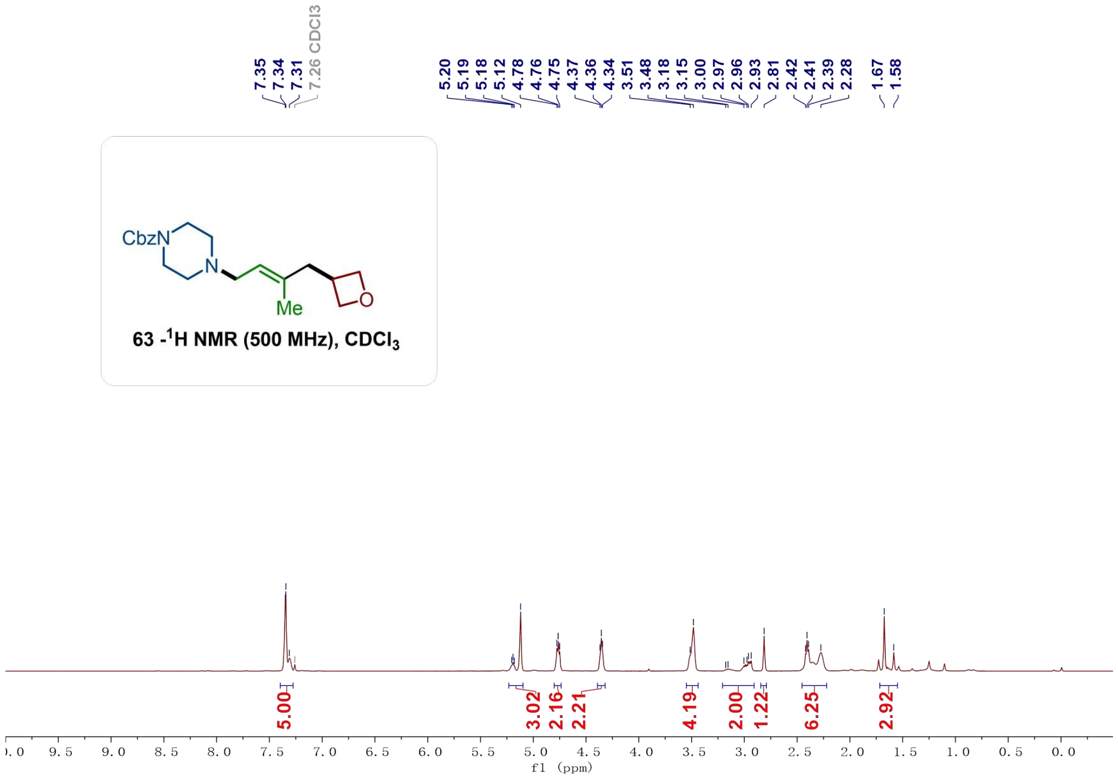

Supplementary Figure 193. ^{1}H NMR (500 MHz, $CDCl_3$) spectrum of compound **63**

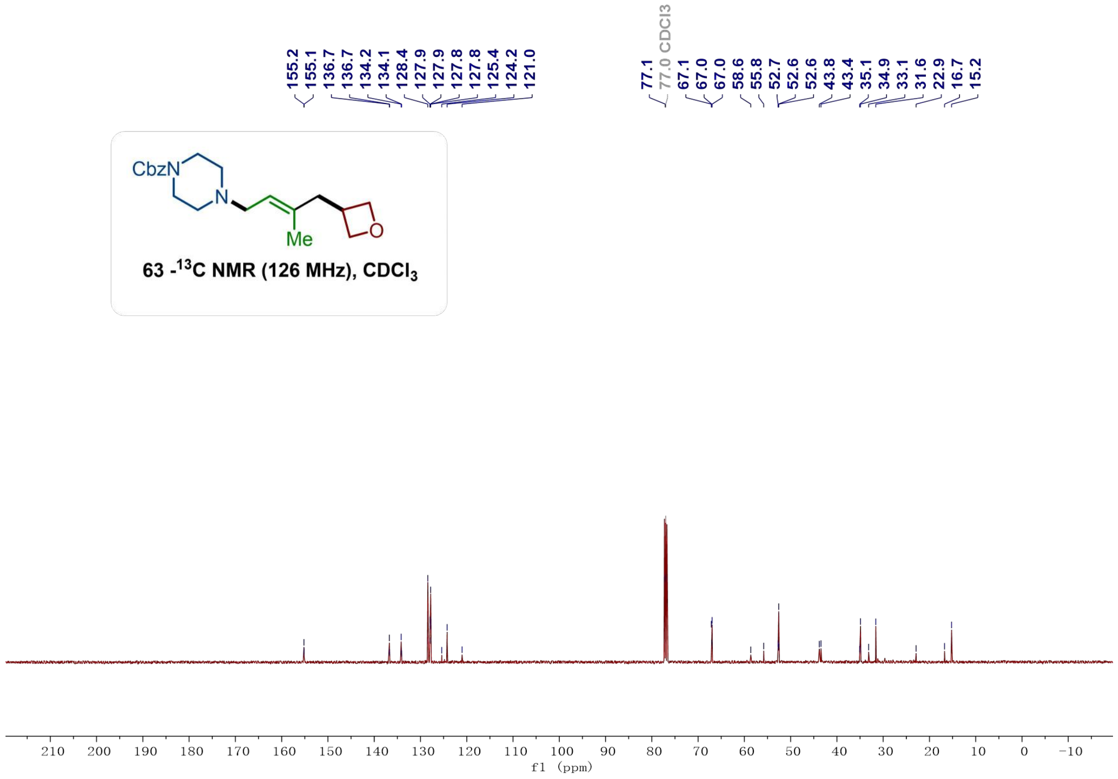

Supplementary Figure 194. ^{13}C NMR (126 MHz, $CDCl_3$) spectrum of compound **63**

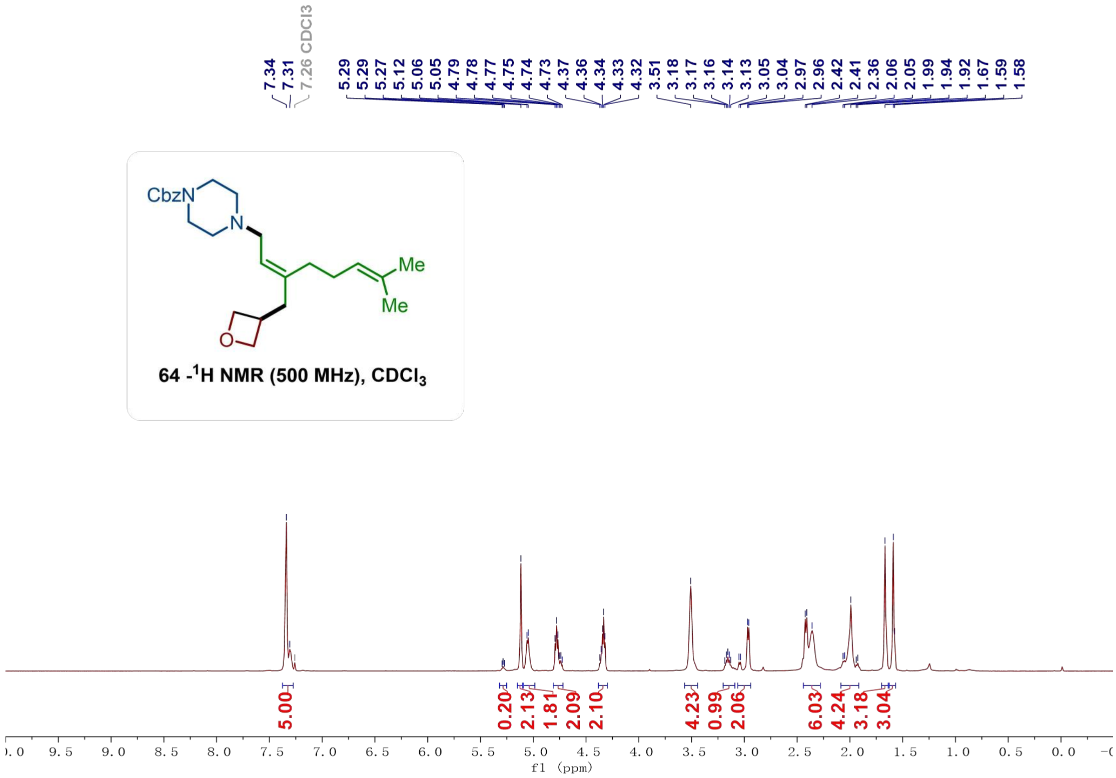

Supplementary Figure 195. ^{1}H NMR (500 MHz, $CDCl_3$) spectrum of compound **64**

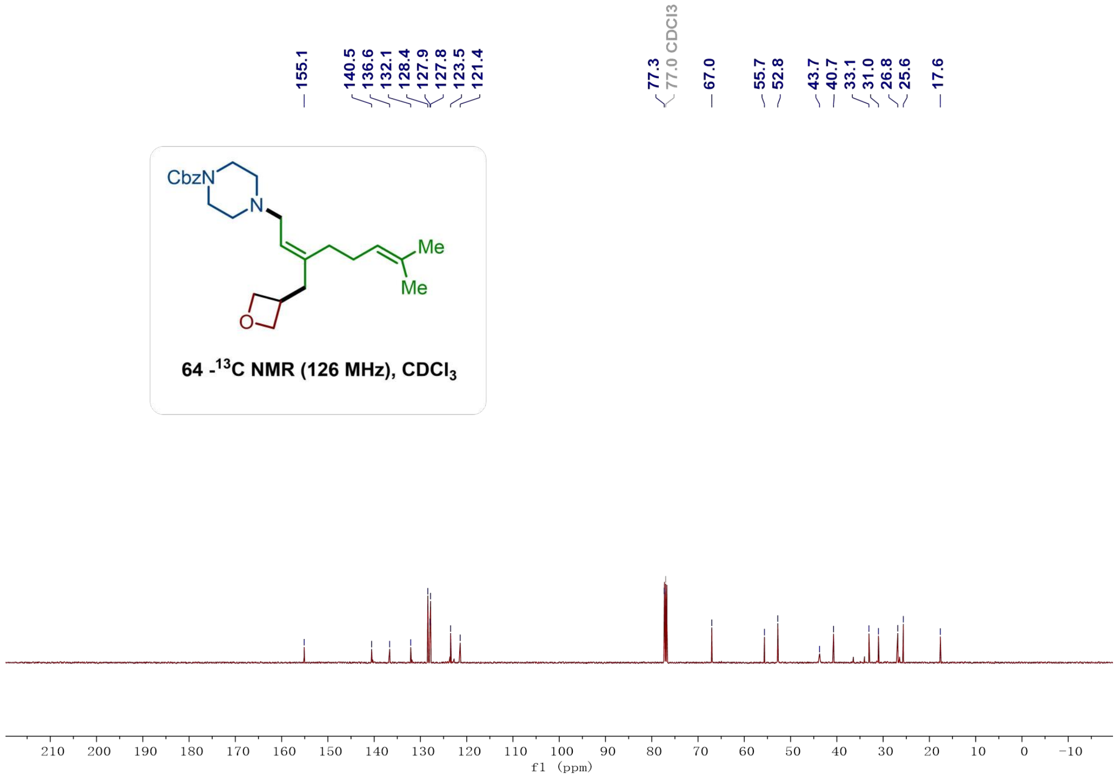

Supplementary Figure 196. ^{13}C NMR (126 MHz, $CDCl_3$) spectrum of compound **64**

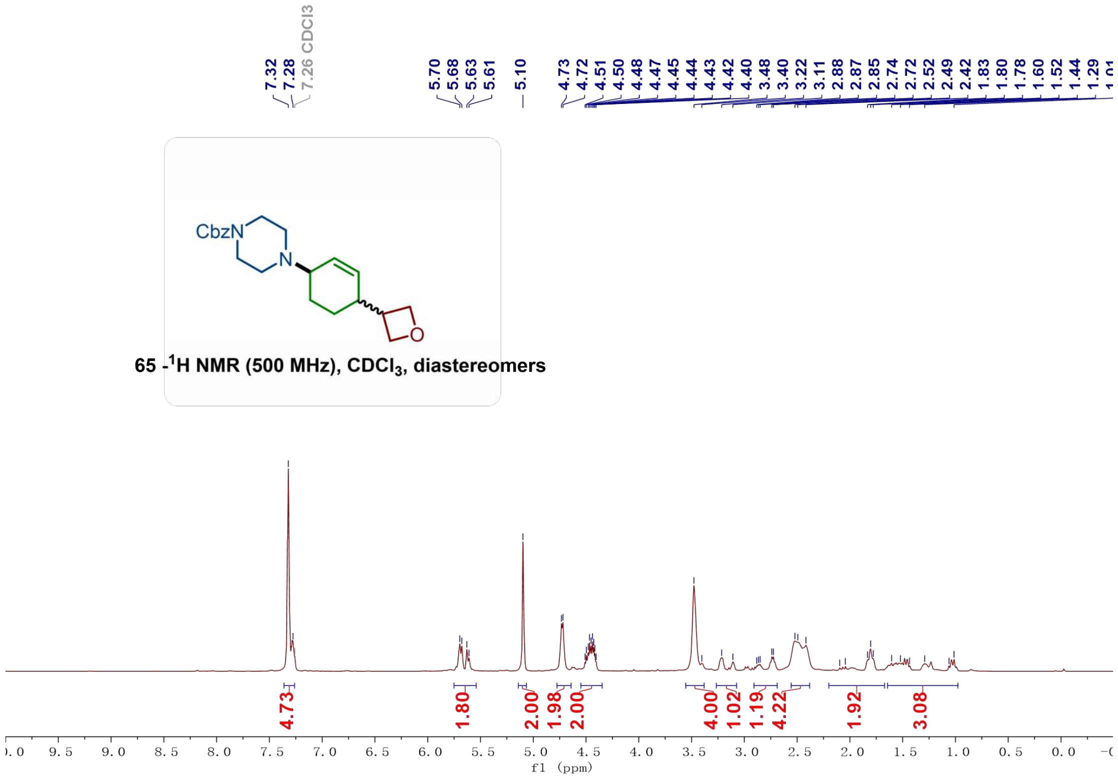

Supplementary Figure 197. ^{1}H NMR (500 MHz, $CDCl_3$) spectrum of compound **65**

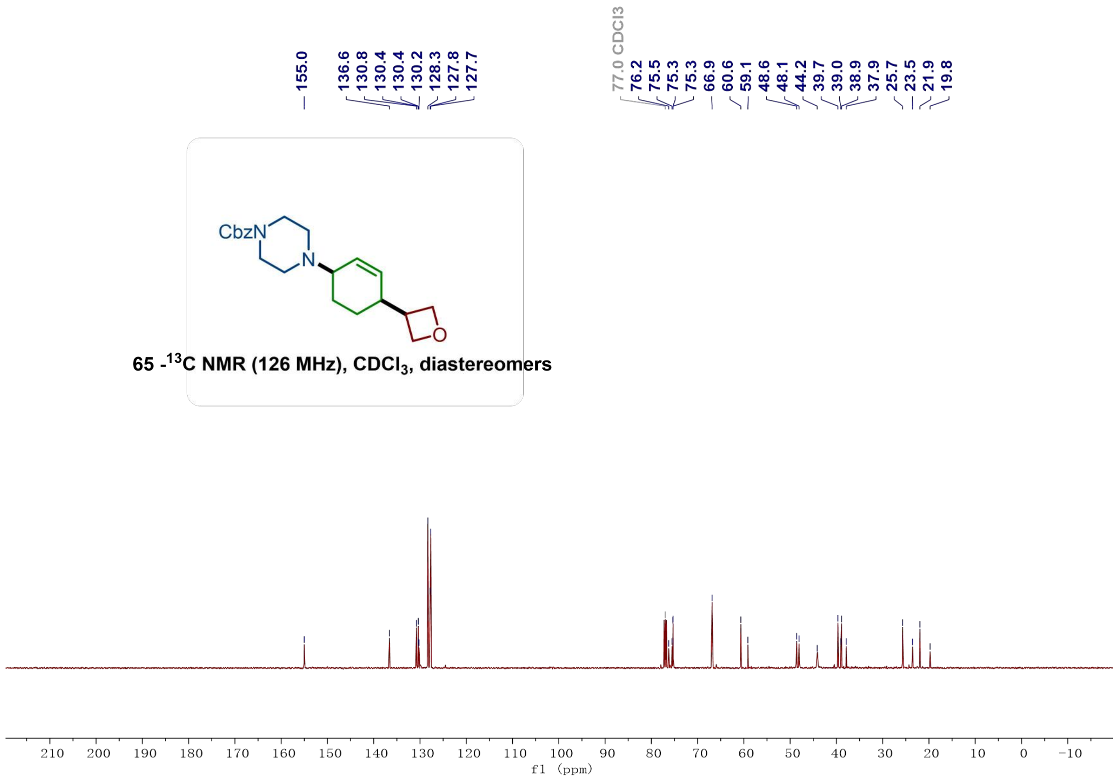

Supplementary Figure 198. ^{13}C NMR (126 MHz, $CDCl_3$) spectrum of compound **65**

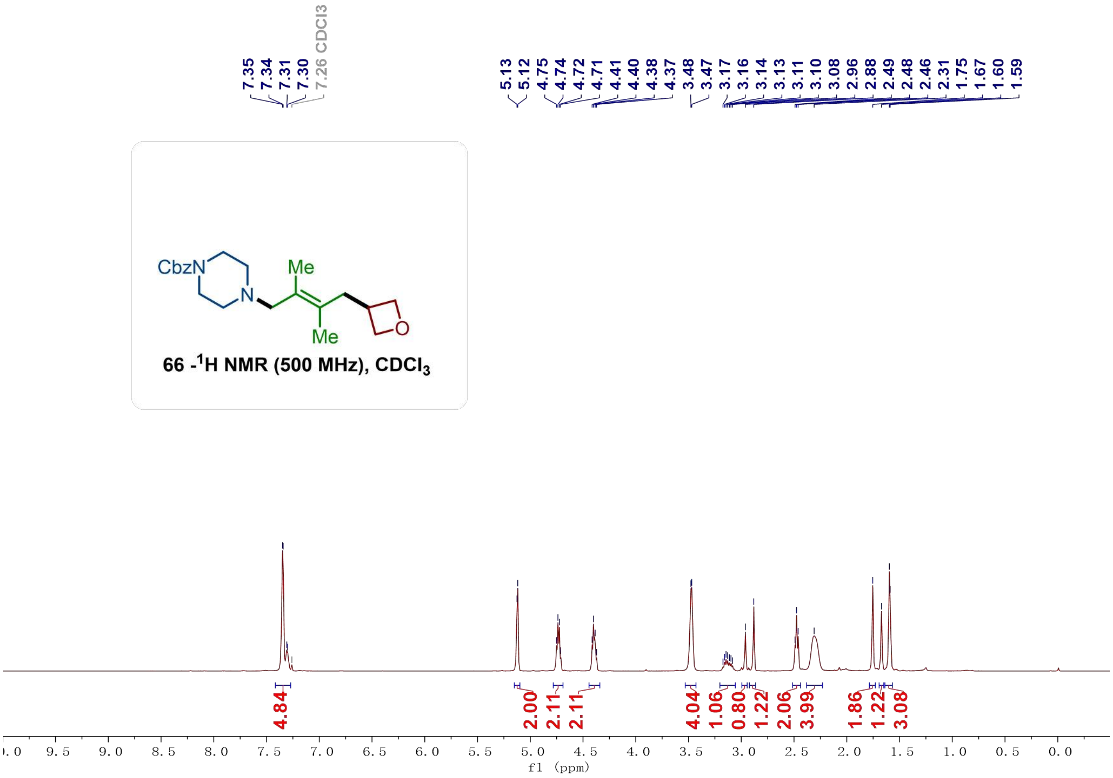

Supplementary Figure 199. ^{1}H NMR (400 MHz, $CDCl_3$) spectrum of compound **66**

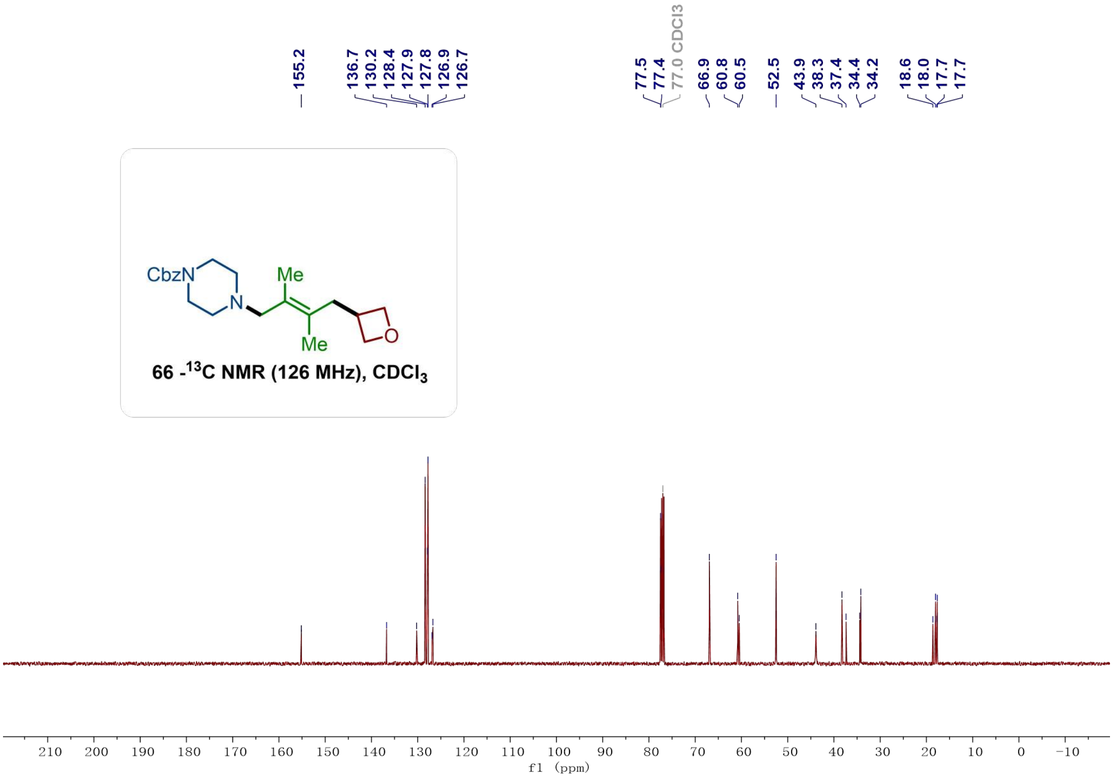

Supplementary Figure 200. ^{13}C NMR (126 MHz, $CDCl_3$) spectrum of compound **66**

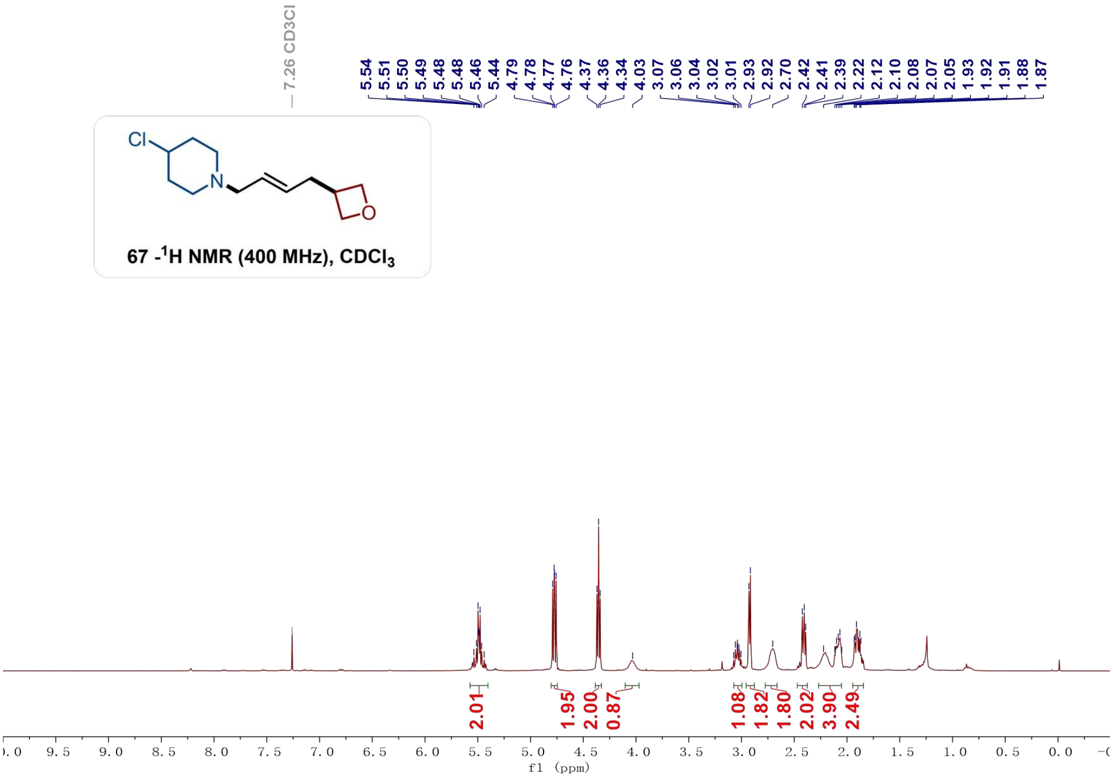

Supplementary Figure 201. ^{1}H NMR (400 MHz, $CDCl_3$) spectrum of compound **67**

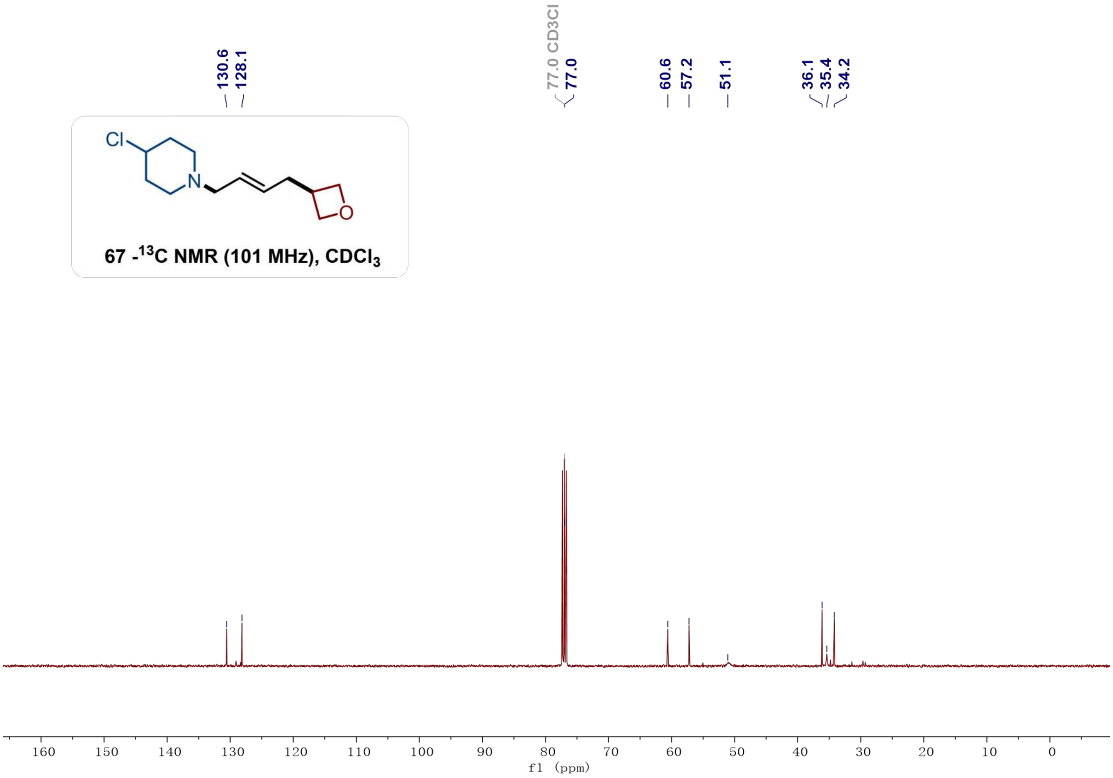

Supplementary Figure 202. ^{13}C NMR (101 MHz, $CDCl_3$) spectrum of compound **67**

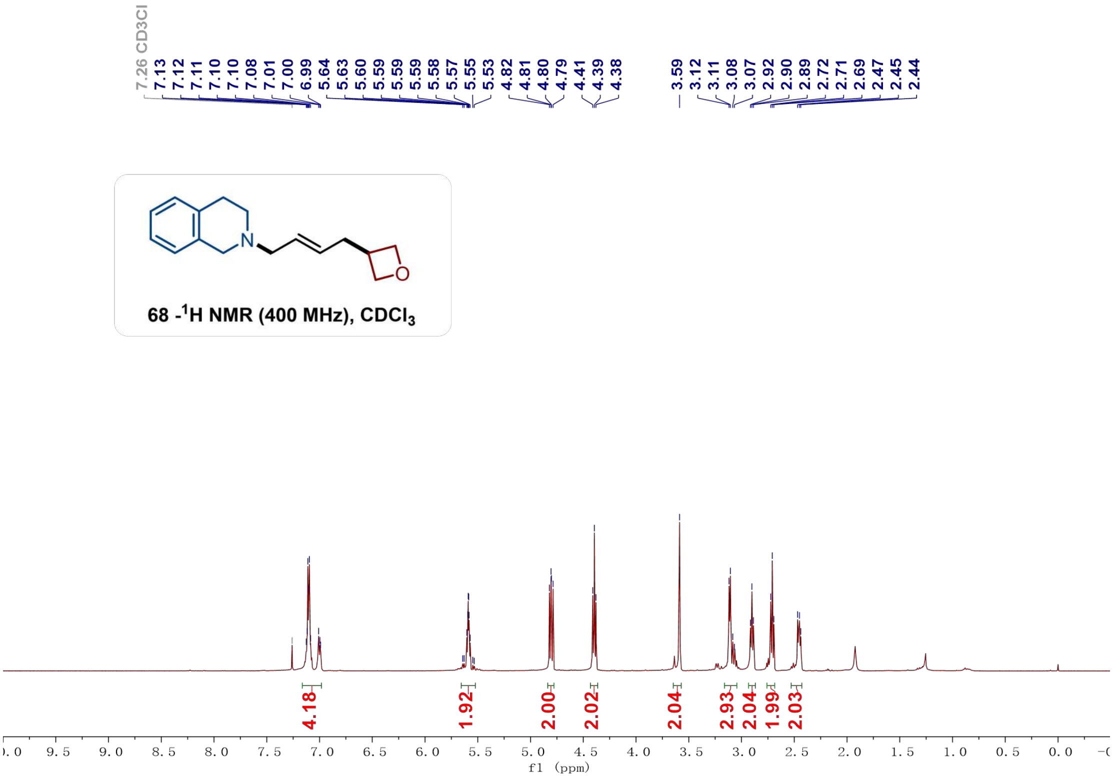

Supplementary Figure 203. ^{1}H NMR (400 MHz, $CDCl_3$) spectrum of compound **68**

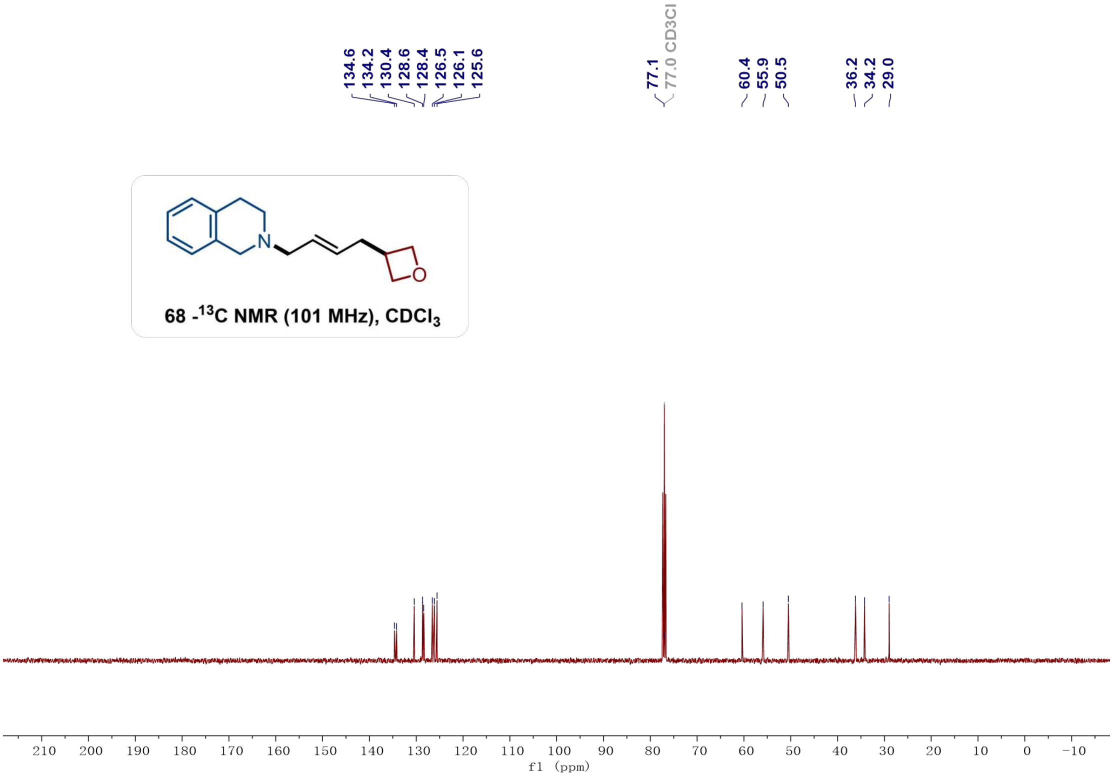

Supplementary Figure 204. ^{13}C NMR (101 MHz, $CDCl_3$) spectrum of compound **68**

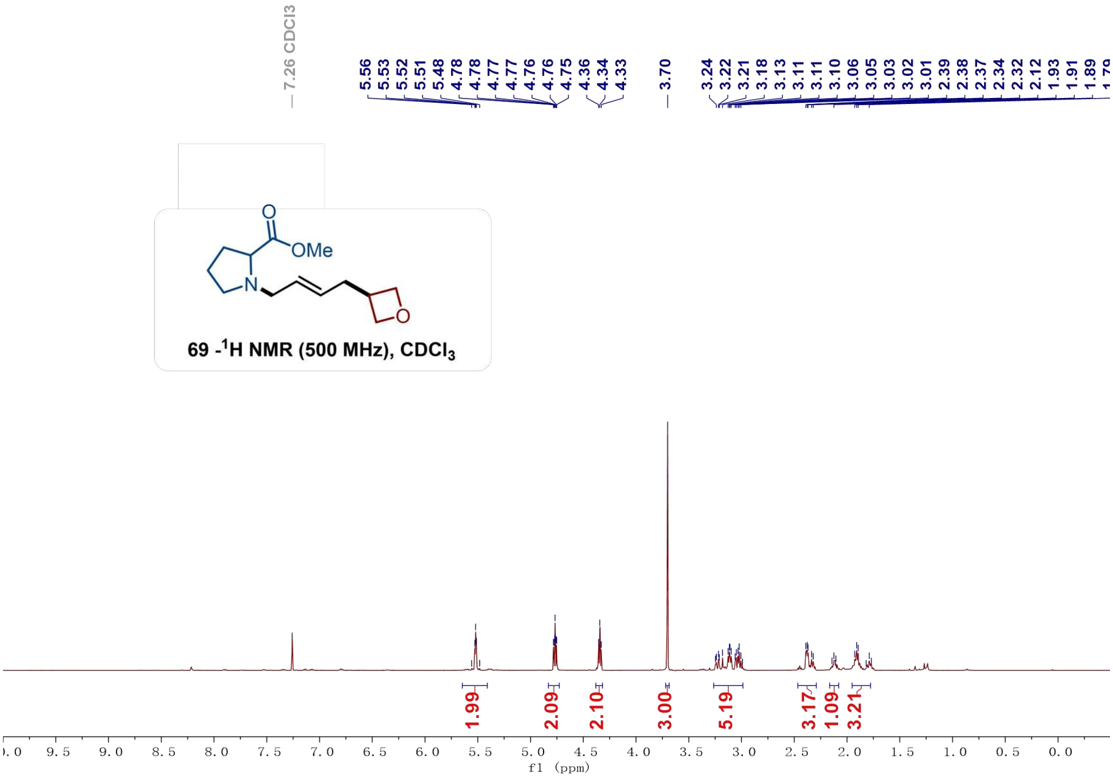

Supplementary Figure 205. ^{1}H NMR (500 MHz, $CDCl_3$) spectrum of compound **69**

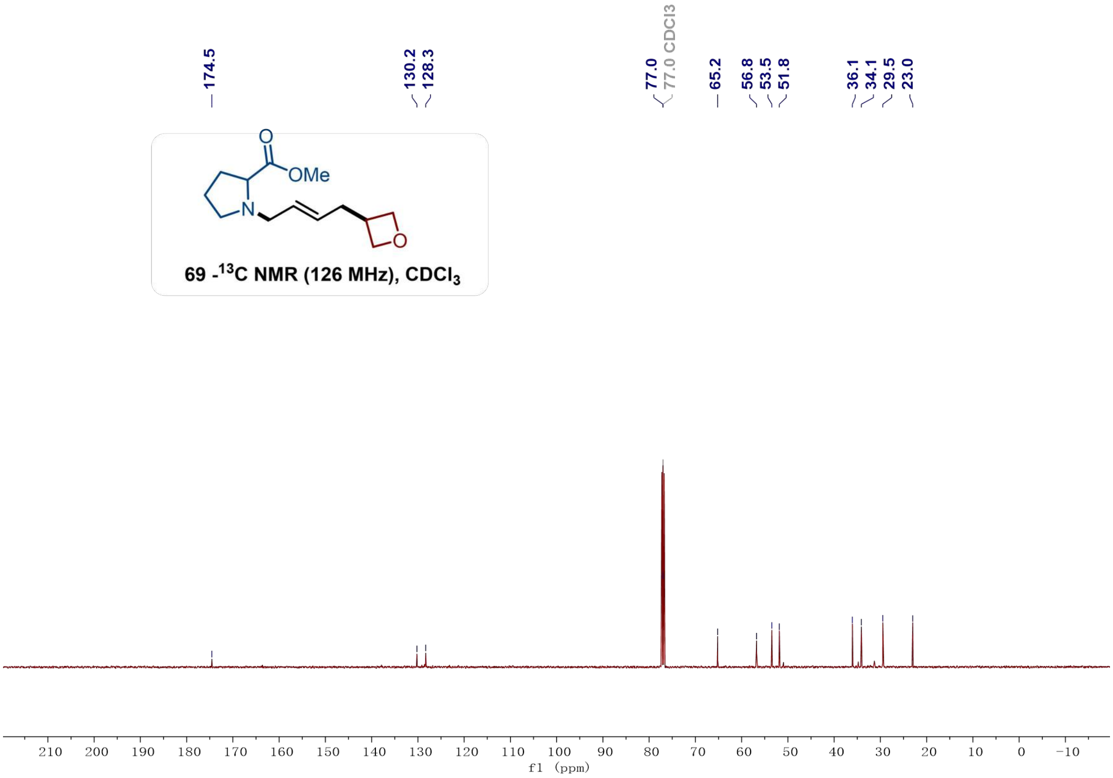

Supplementary Figure 206. ^{13}C NMR (126 MHz, $CDCl_3$) spectrum of compound **69**

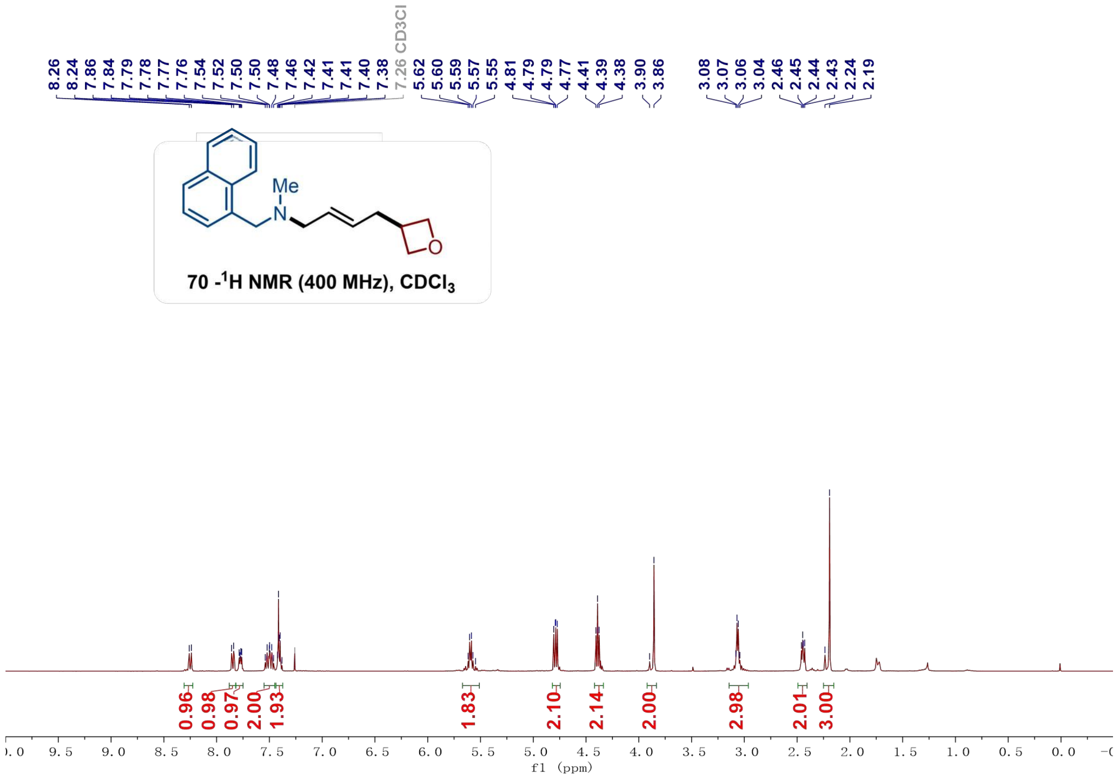

Supplementary Figure 207. ^{1}H NMR (400 MHz, $CDCl_3$) spectrum of compound **70**

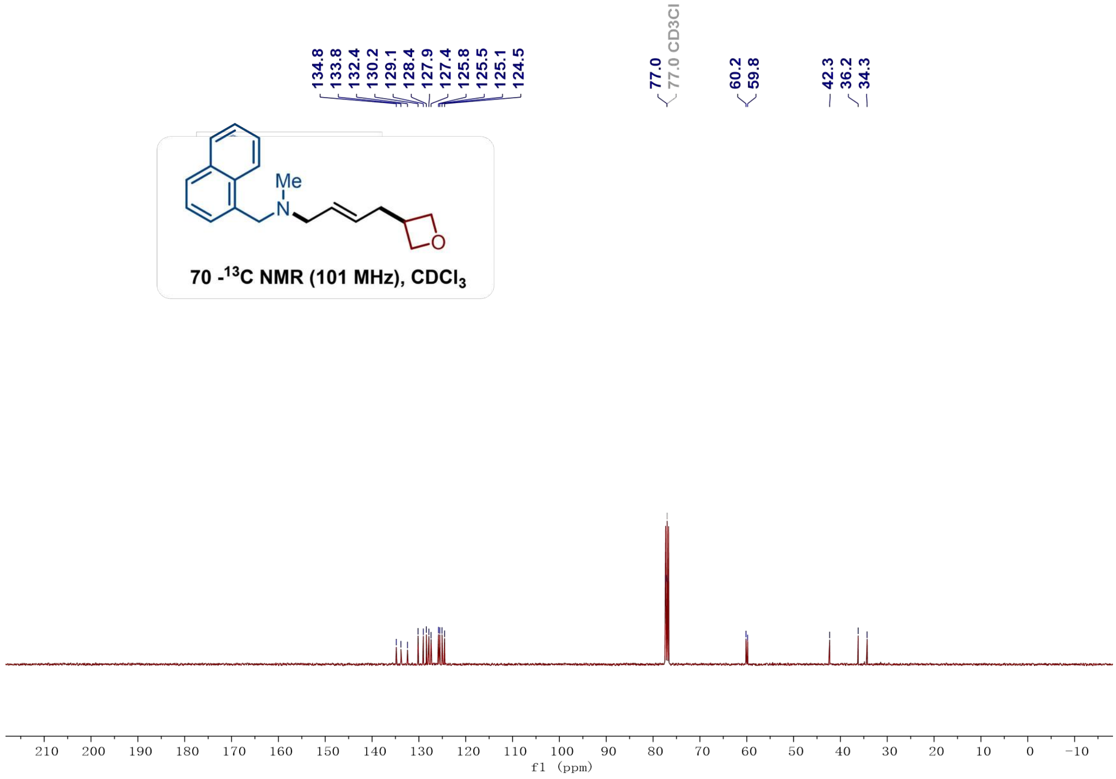

Supplementary Figure 208. ^{13}C NMR (101 MHz, $CDCl_3$) spectrum of compound **70**

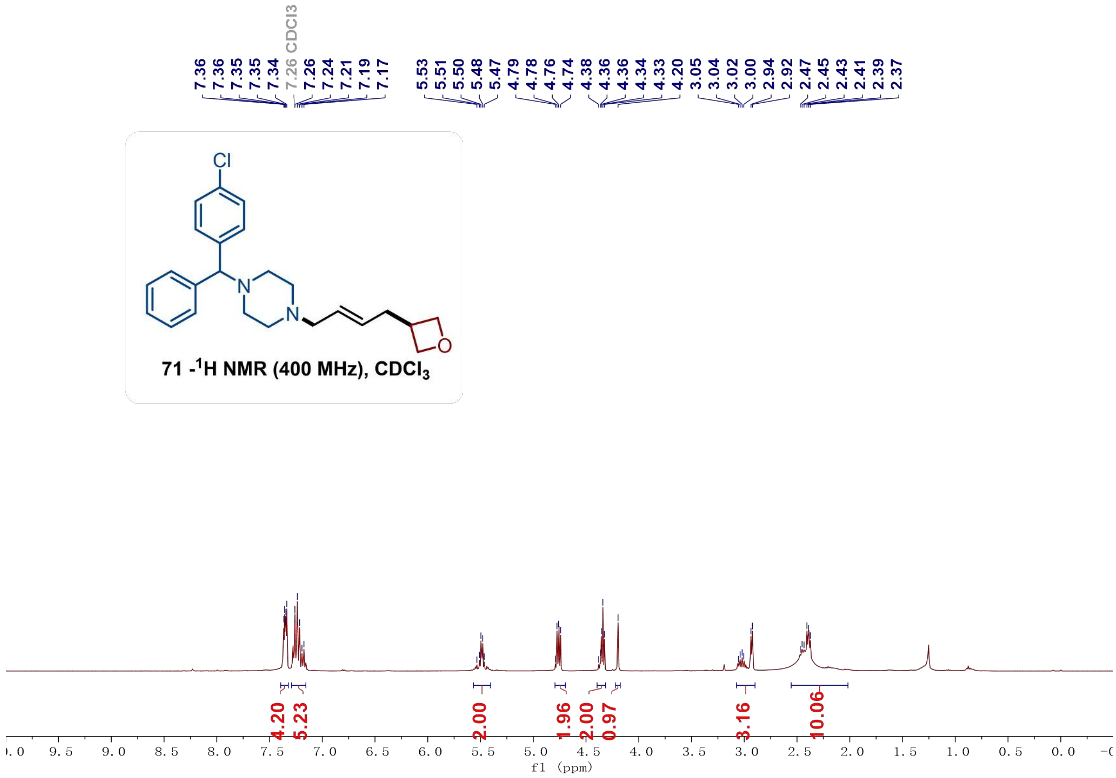

Supplementary Figure 209. ^{1}H NMR (400 MHz, $CDCl_3$) spectrum of compound **71**

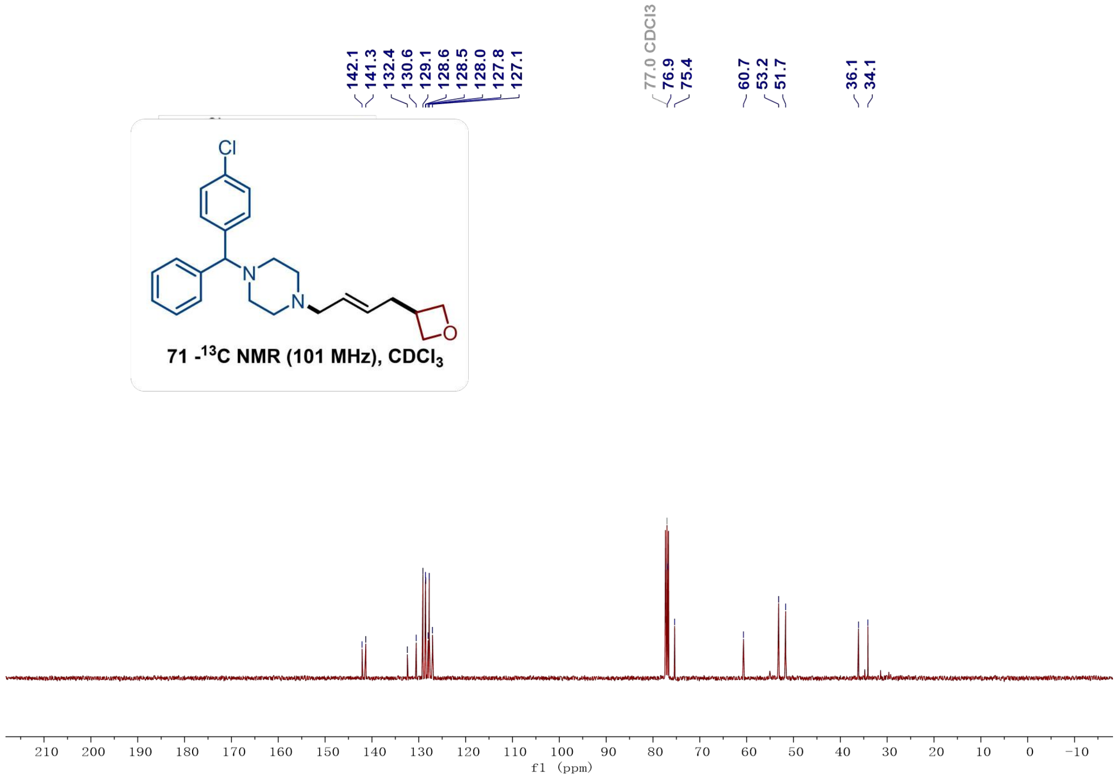

Supplementary Figure 210. ^{13}C NMR (101 MHz, $CDCl_3$) spectrum of compound **71**

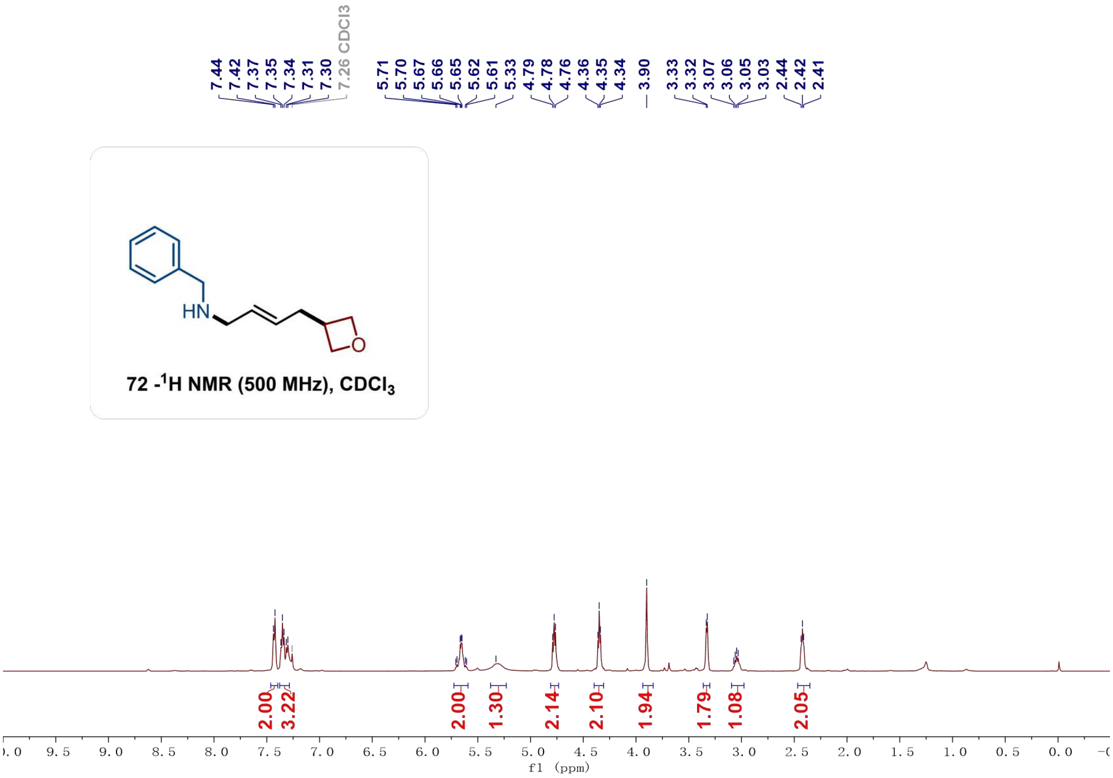

Supplementary Figure 211. ^{1}H NMR (500 MHz, $CDCl_3$) spectrum of compound **72**

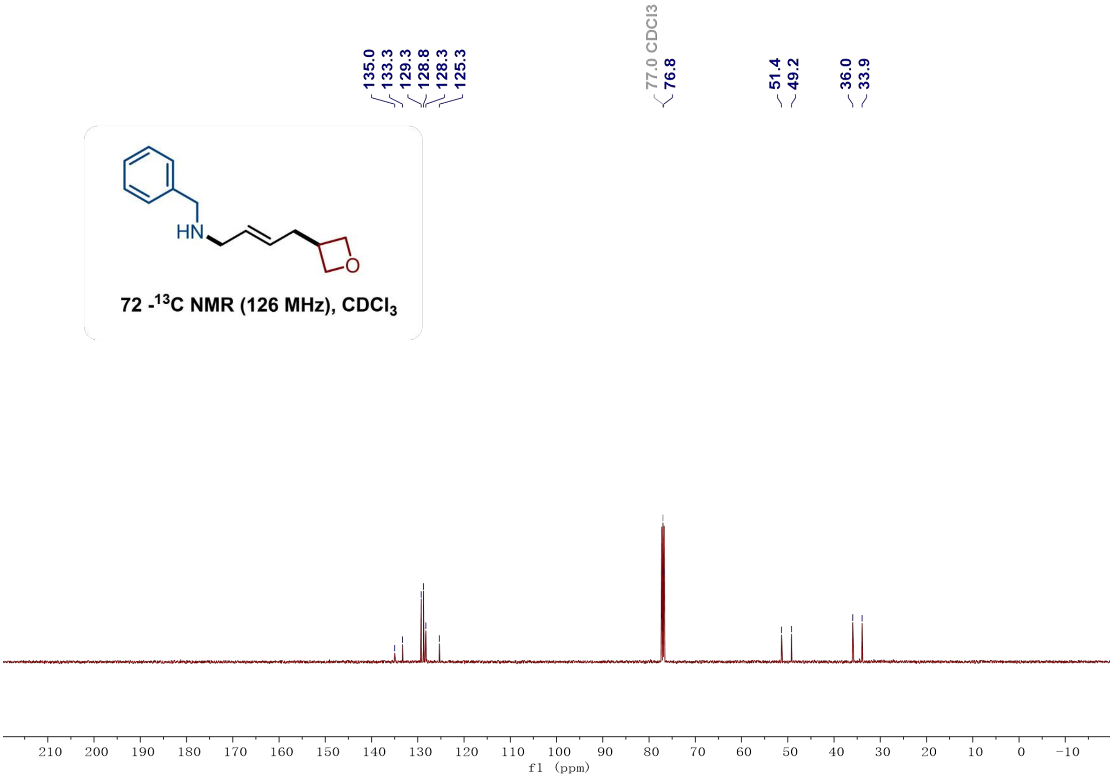

Supplementary Figure 212. ^{13}C NMR (126 MHz, $CDCl_3$) spectrum of compound **72**

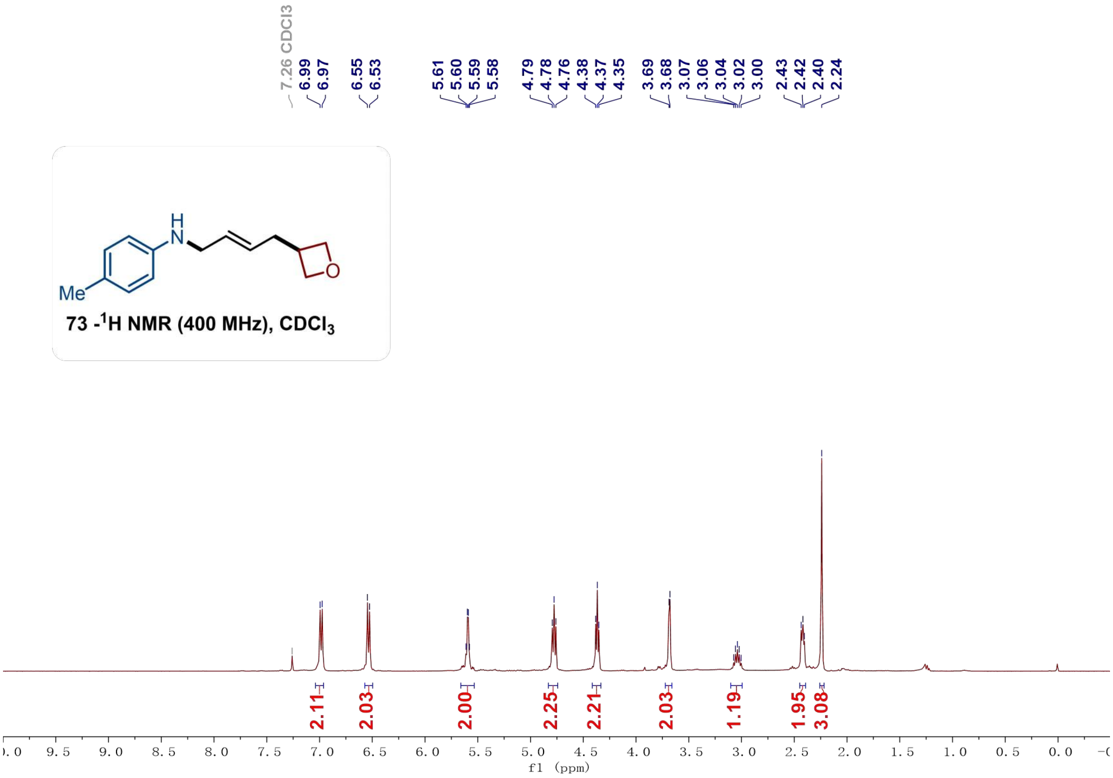

Supplementary Figure 213. ^{1}H NMR (400 MHz, $CDCl_3$) spectrum of compound **73**

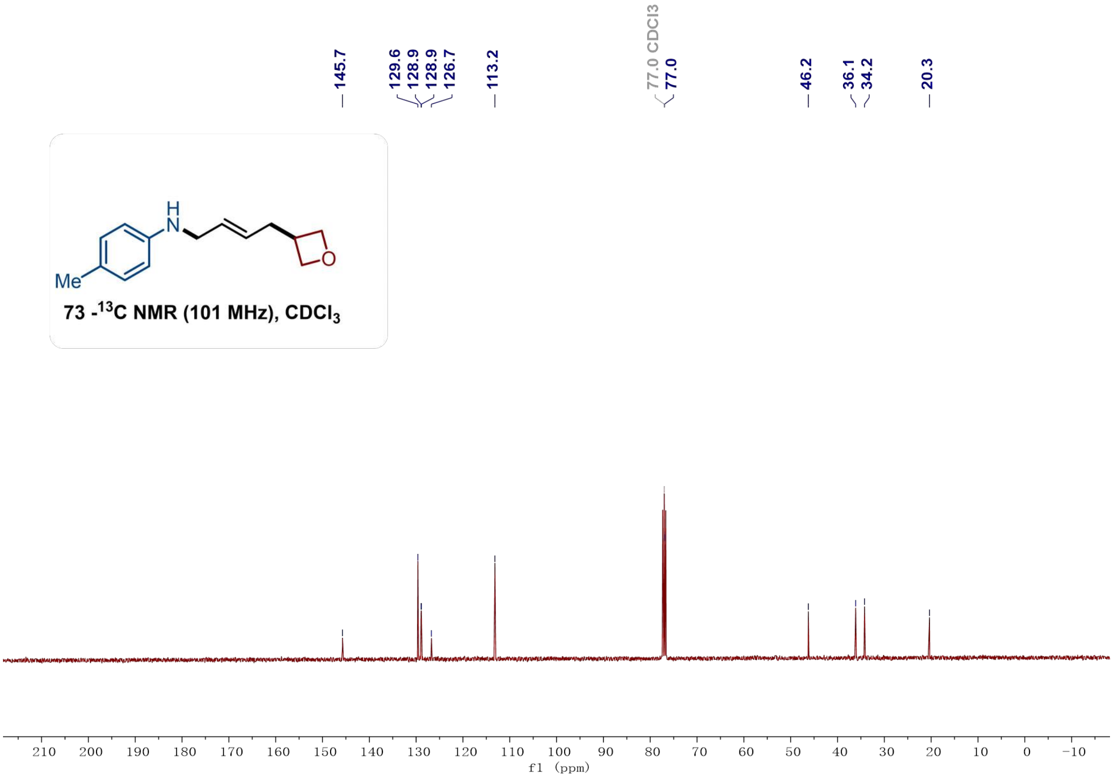

Supplementary Figure 214. ^{13}C NMR (101 MHz, $CDCl_3$) spectrum of compound **73**

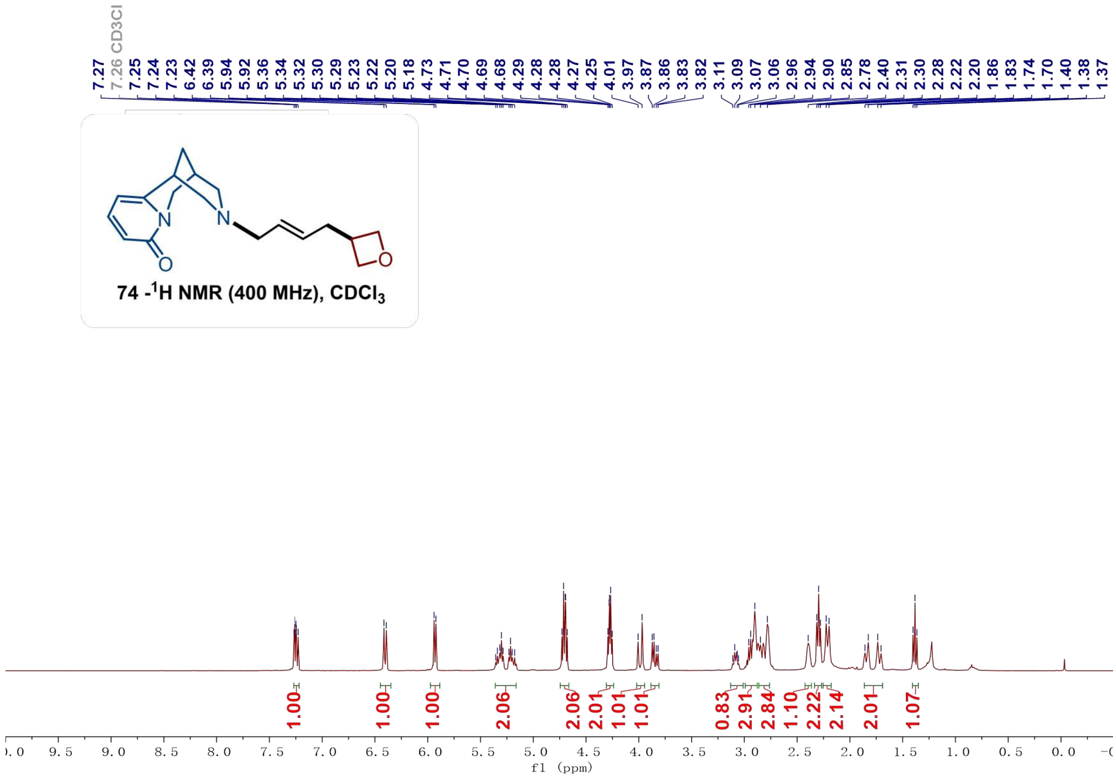

Supplementary Figure 215. ^{1}H NMR (400 MHz, $CDCl_3$) spectrum of compound **74**

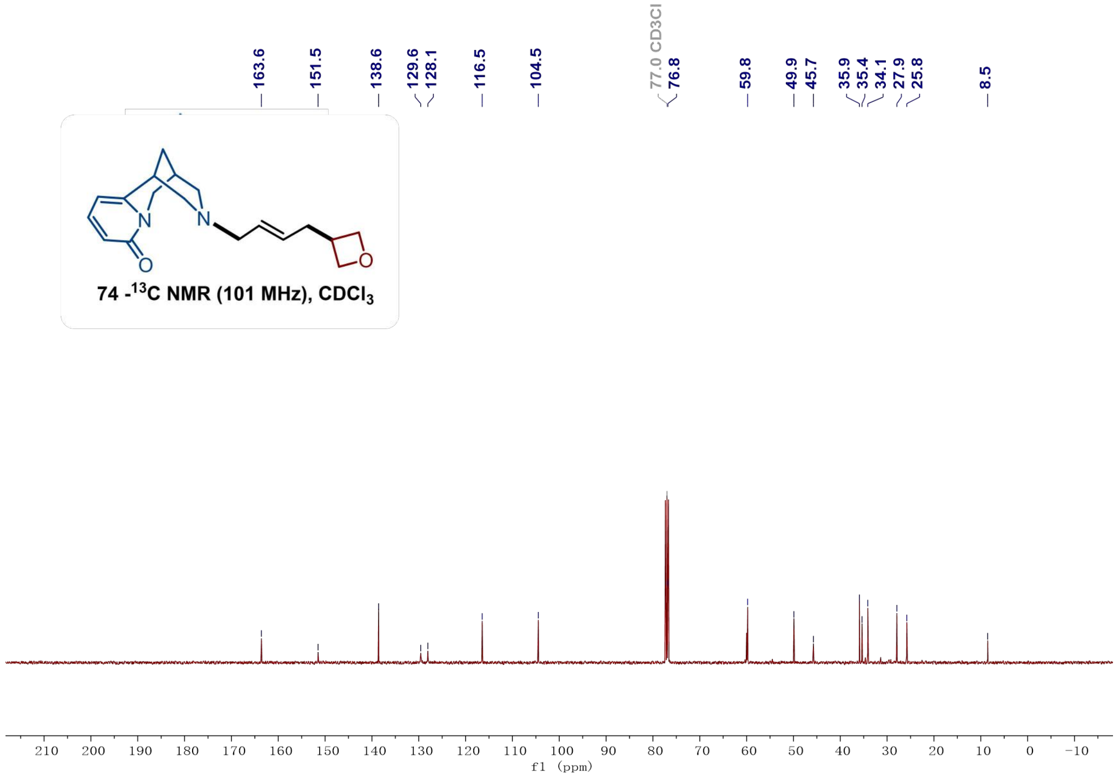

Supplementary Figure 216. ^{13}C NMR (101 MHz, $CDCl_3$) spectrum of compound **74**

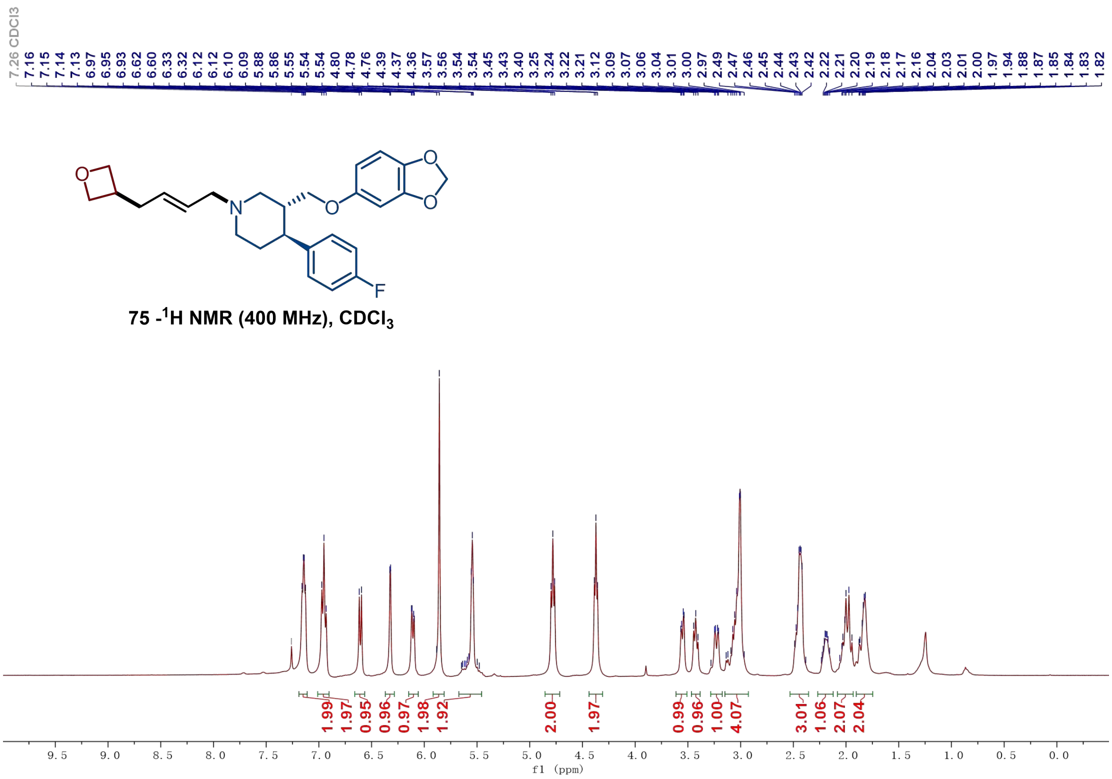

Supplementary Figure 217. ^{1}H NMR (400 MHz, $CDCl_3$) spectrum of compound **75**

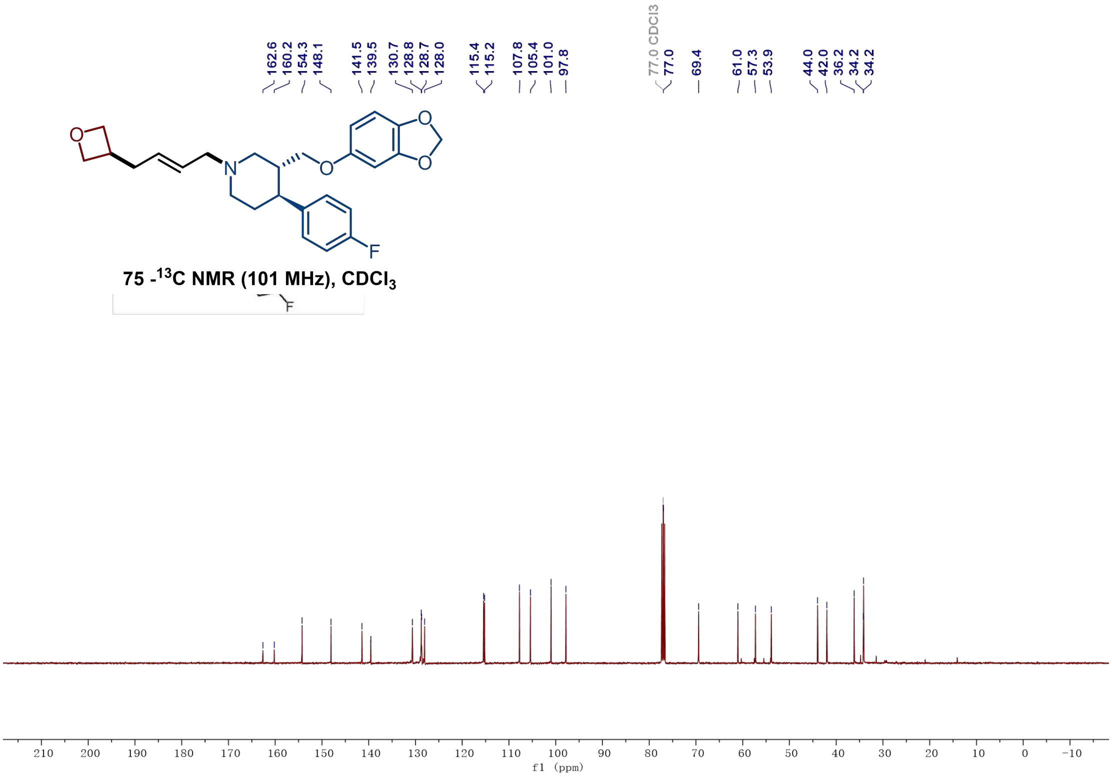

Supplementary Figure 218. ^{13}C NMR (101 MHz, $CDCl_3$) spectrum of compound **75**

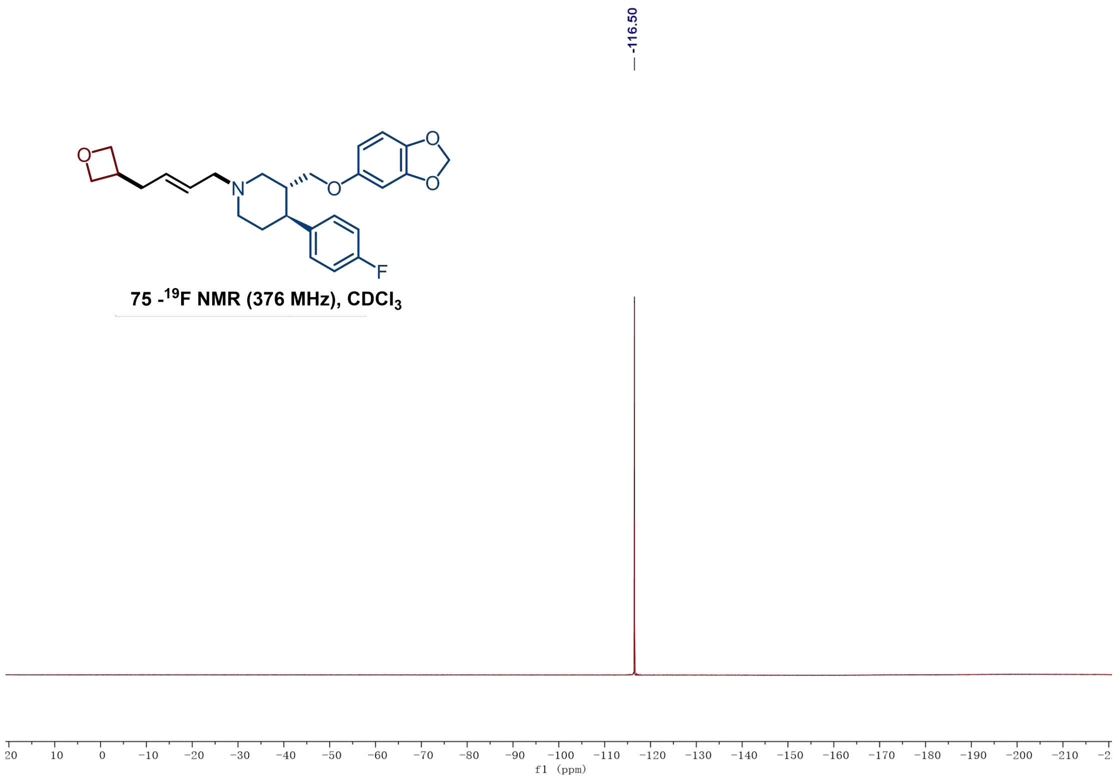

Supplementary Figure 219. ^{19}F NMR (376 MHz, $CDCl_3$) spectrum of compound **75**

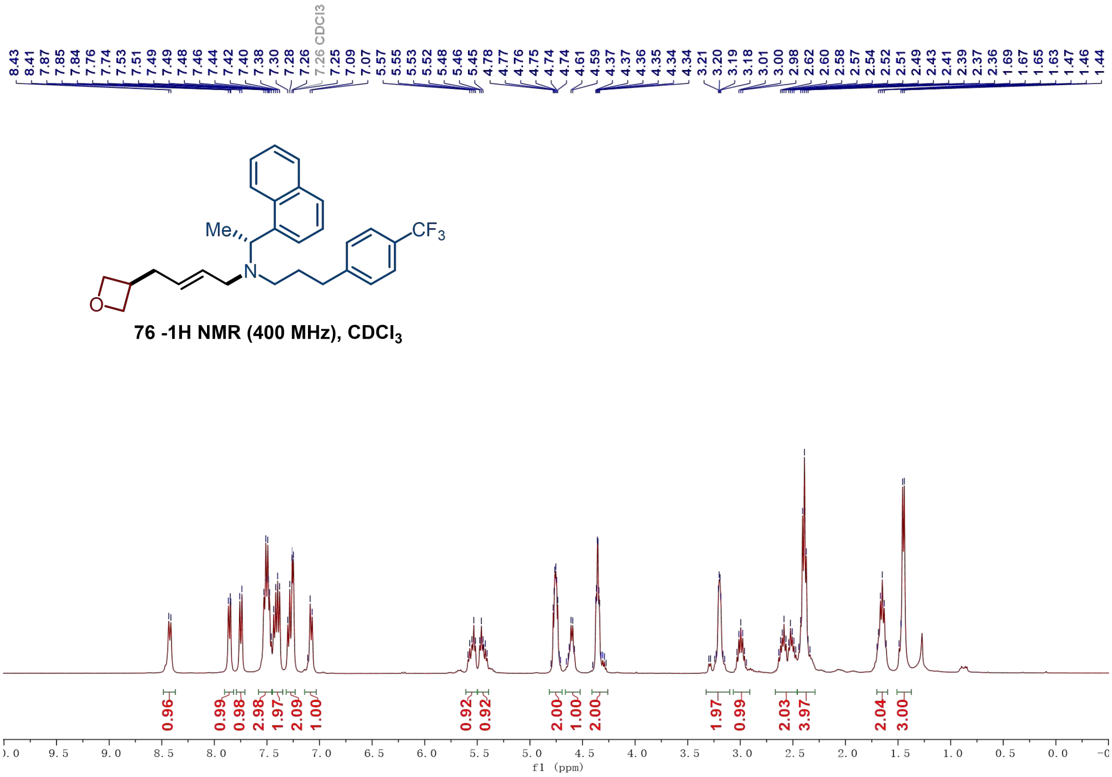

Supplementary Figure 220. ^{1}H NMR (400 MHz, $CDCl_3$) spectrum of compound **76**

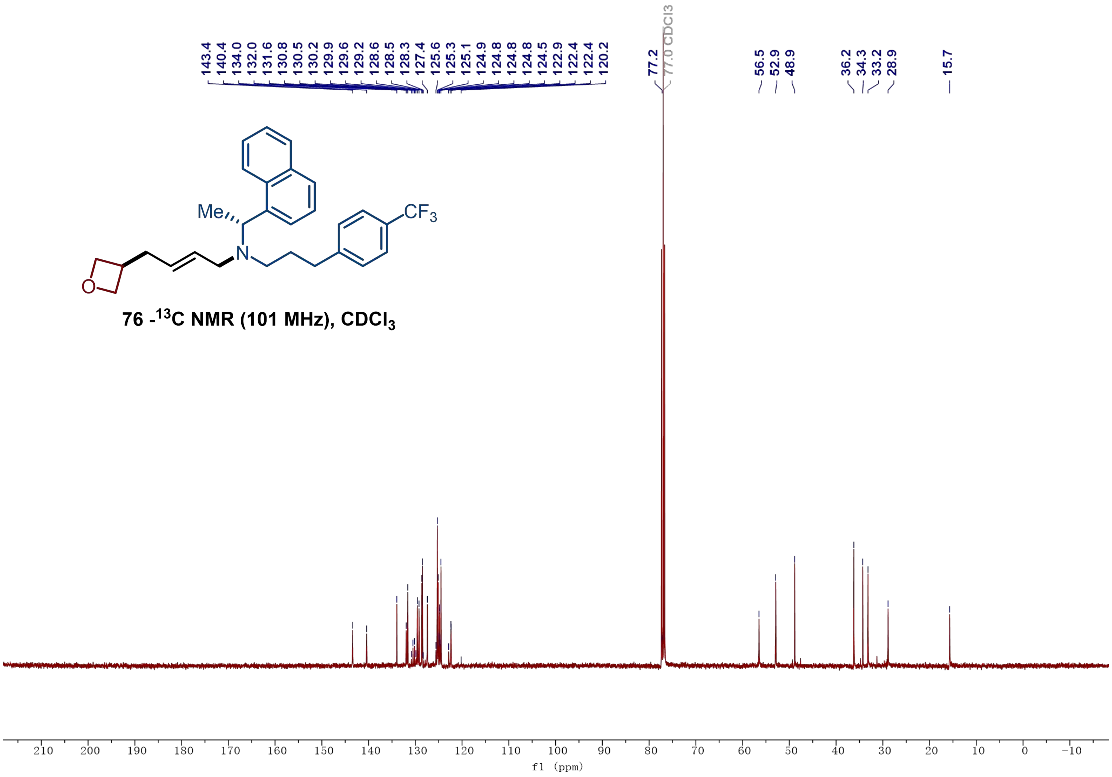

Supplementary Figure 221. ^{13}C NMR (101 MHz, $CDCl_3$) spectrum of compound **76**

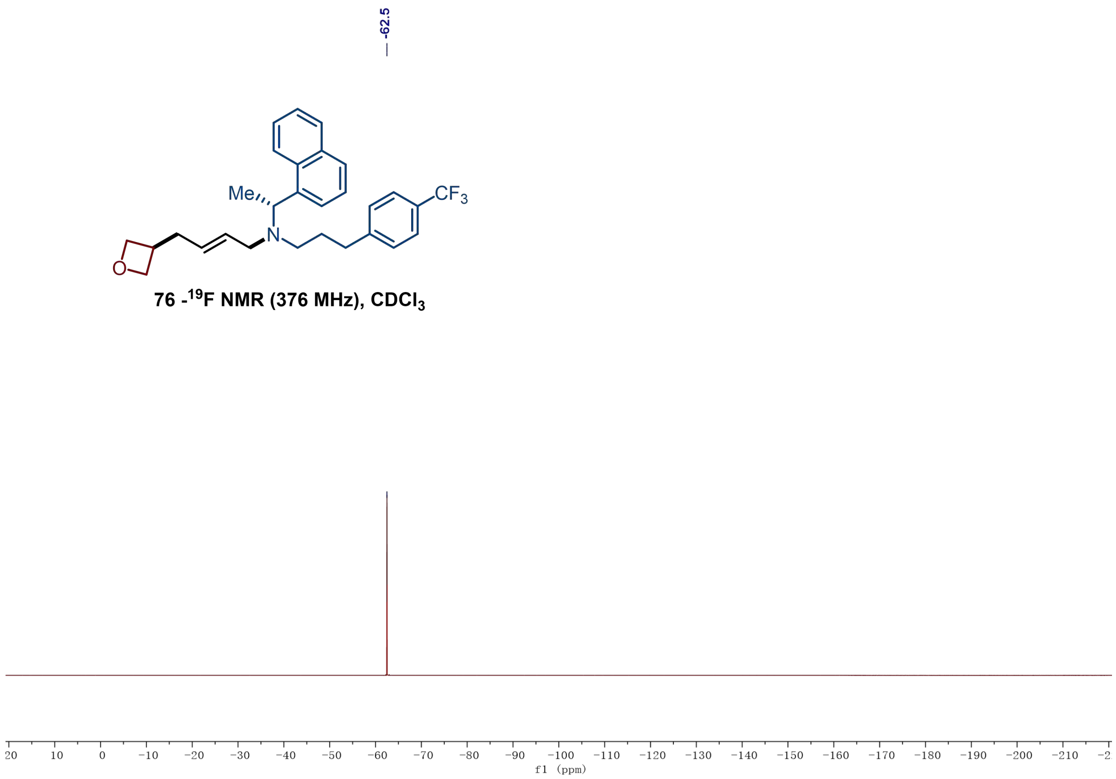

Supplementary Figure 222. ^{19}F NMR (376 MHz, $CDCl_3$) spectrum of compound **76**

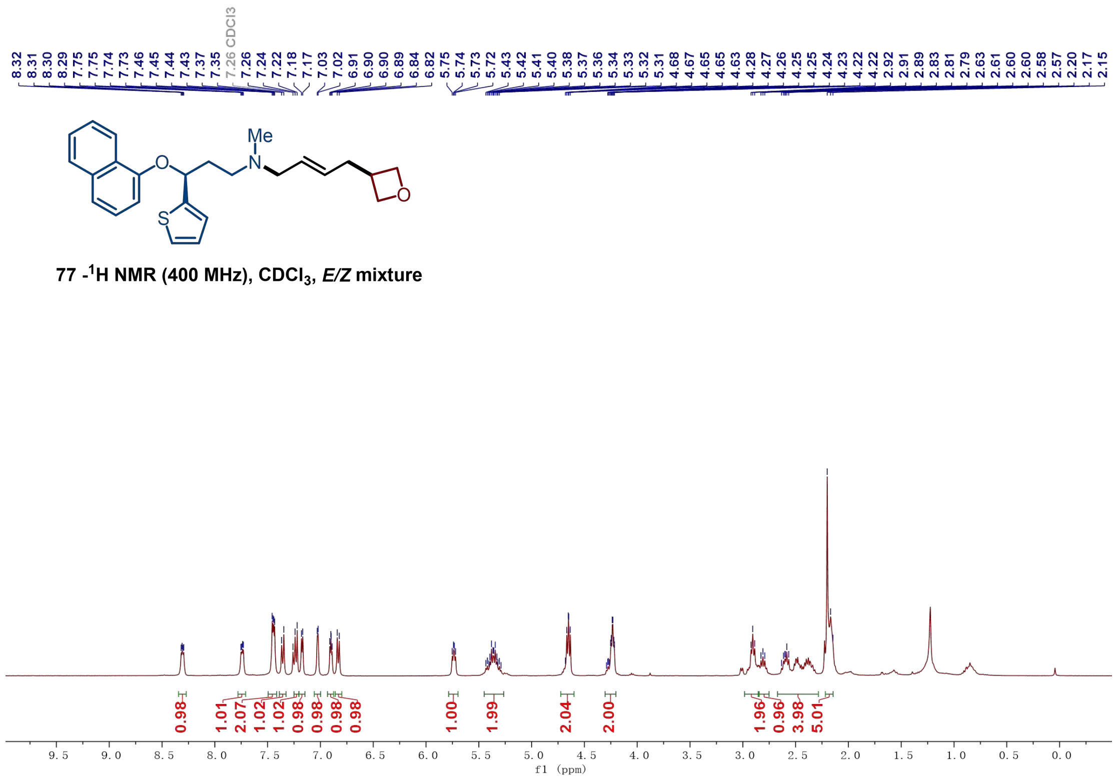

Supplementary Figure 223. ^{1}H NMR (400 MHz, $CDCl_3$) spectrum of compound **77**

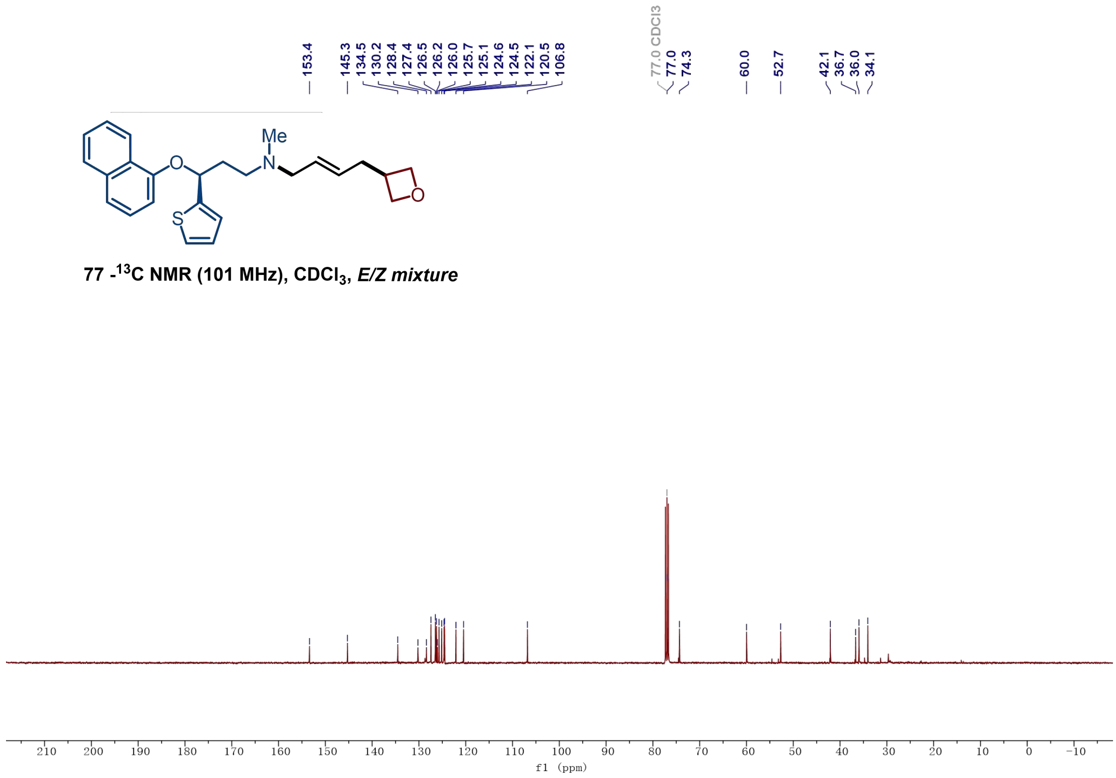

Supplementary Figure 224. ^{13}C NMR (101 MHz, $CDCl_3$) spectrum of compound **77**

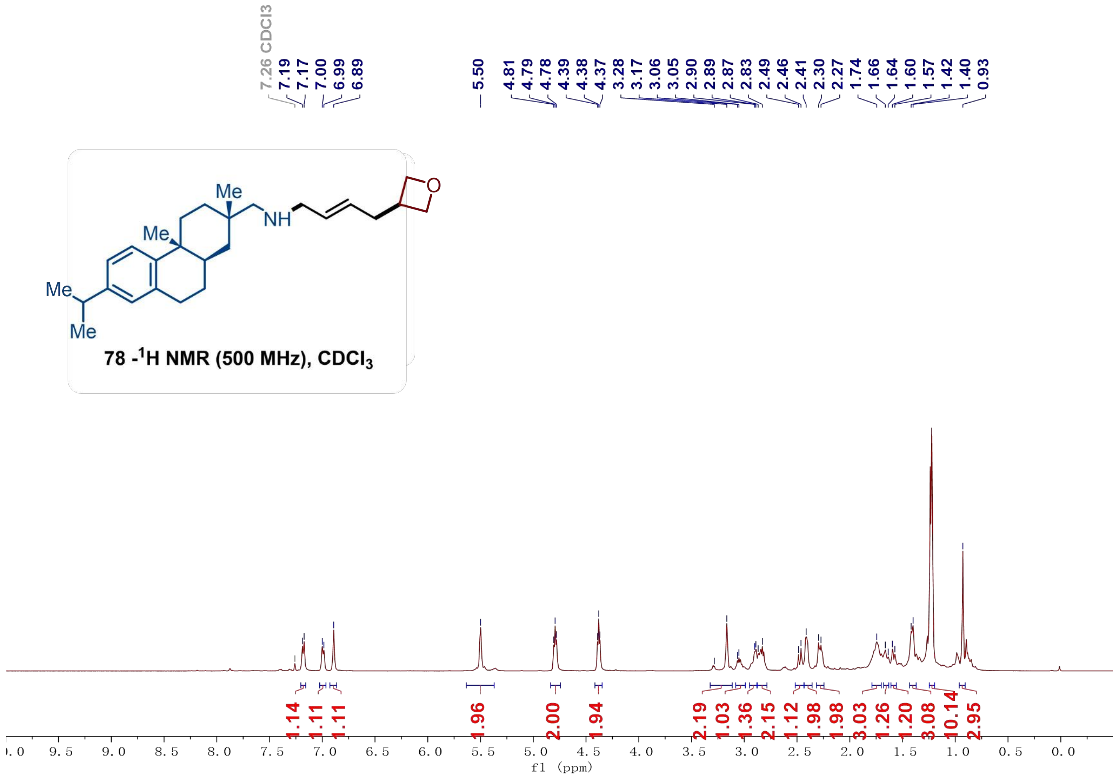

Supplementary Figure 225. ^{1}H NMR (500 MHz, $CDCl_3$) spectrum of compound **78**

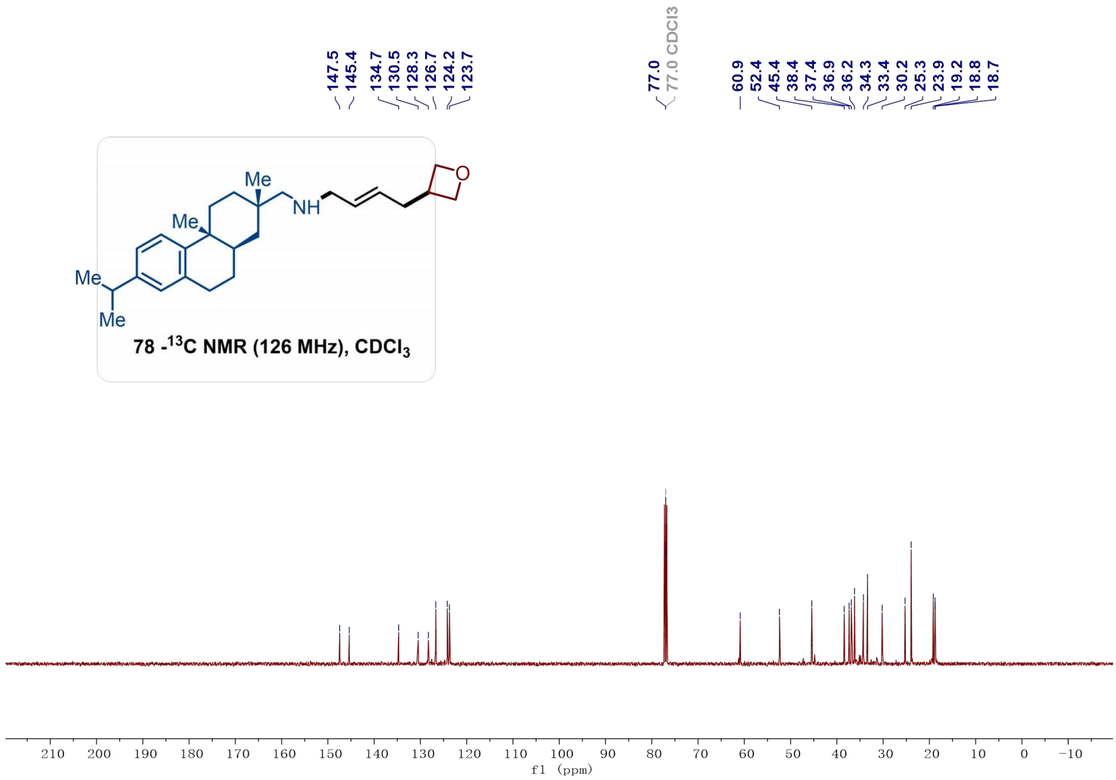

Supplementary Figure 226. ^{13}C NMR (126 MHz, $CDCl_3$) spectrum of compound **78**

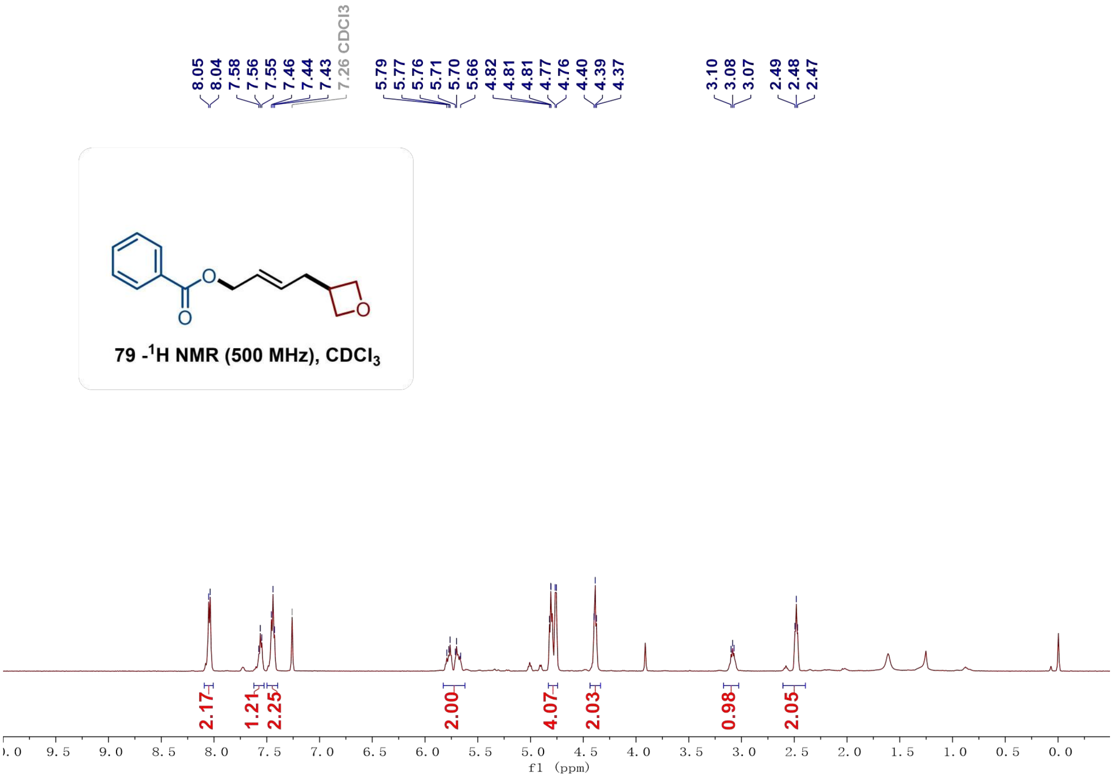

Supplementary Figure 227. ^{1}H NMR (500 MHz, $CDCl_3$) spectrum of compound **79**

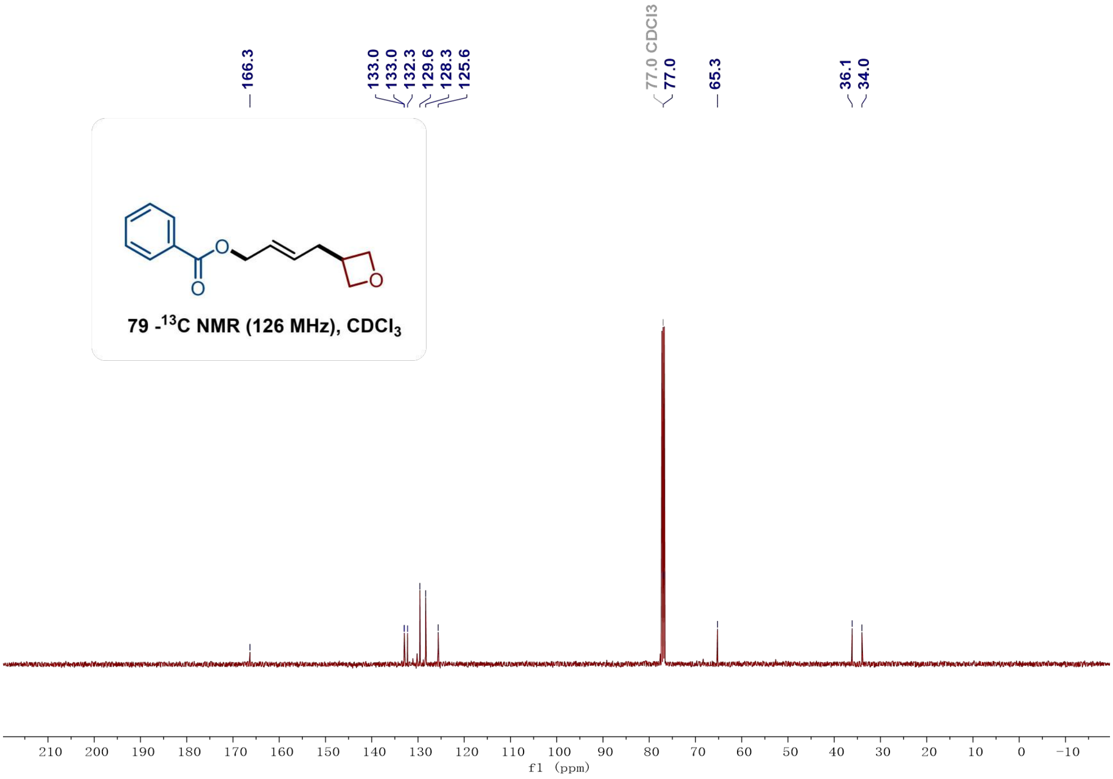

Supplementary Figure 228. ^{13}C NMR (126 MHz, $CDCl_3$) spectrum of compound **79**

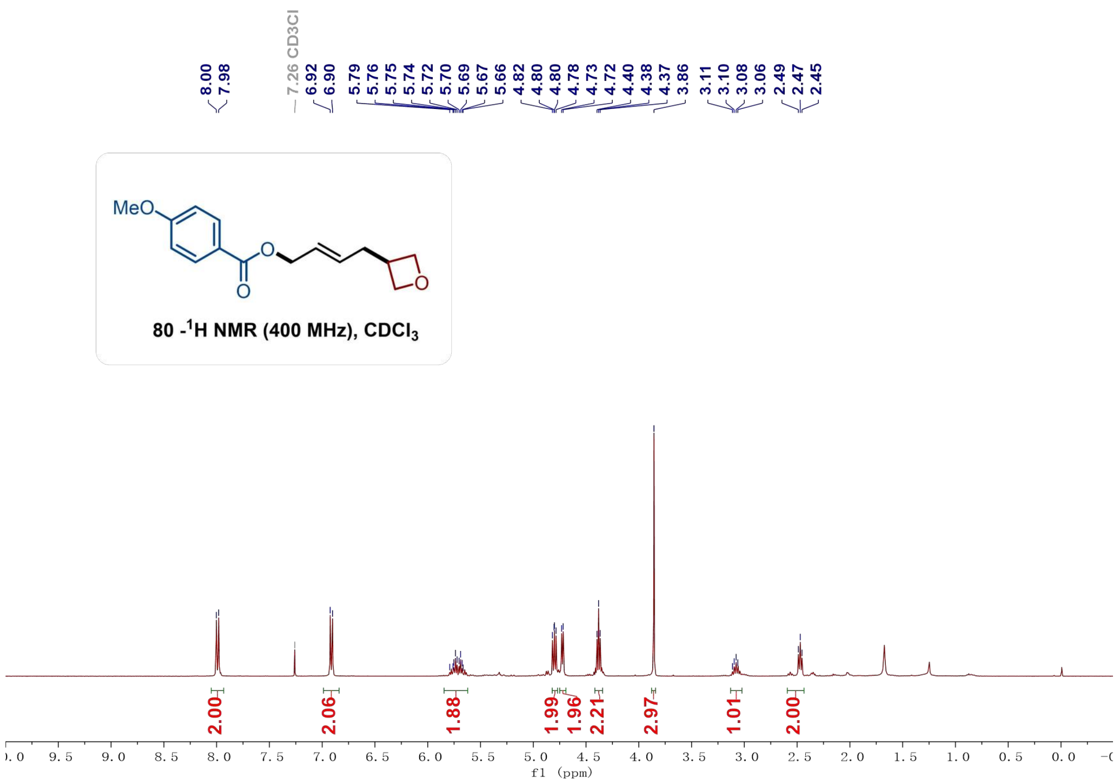

Supplementary Figure 229. ^{1}H NMR (400 MHz, $CDCl_3$) spectrum of compound **80**

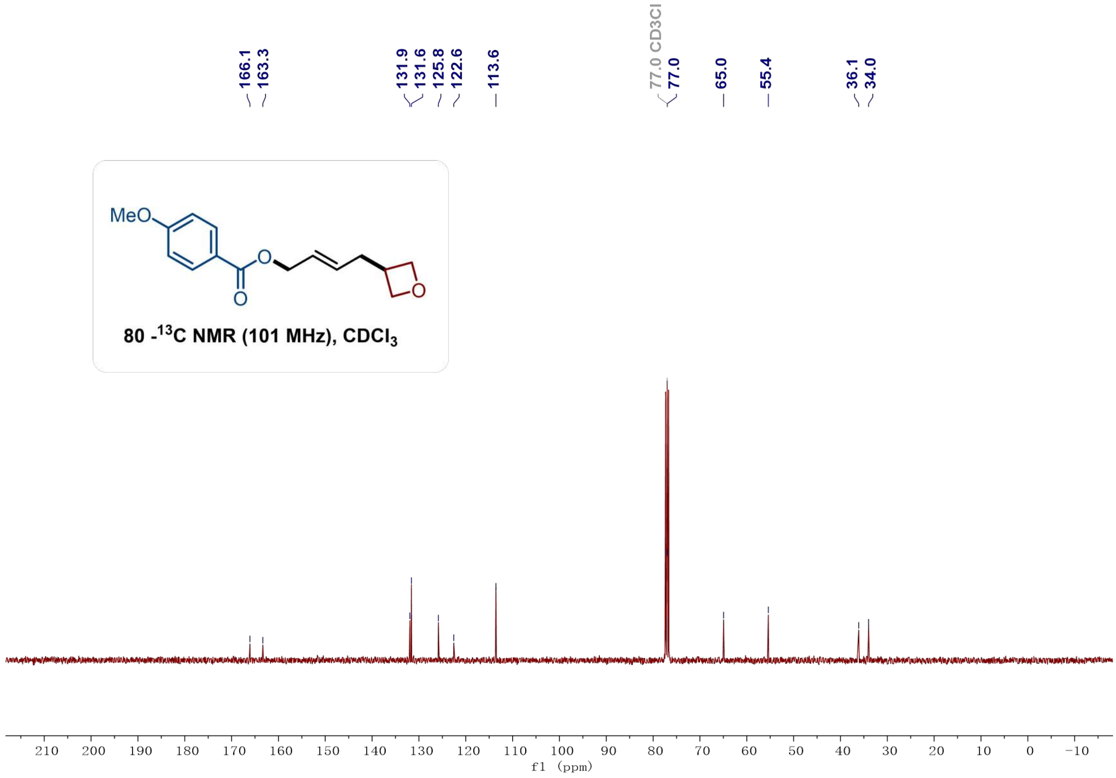

Supplementary Figure 230. ^{13}C NMR (101 MHz, $CDCl_3$) spectrum of compound **80**

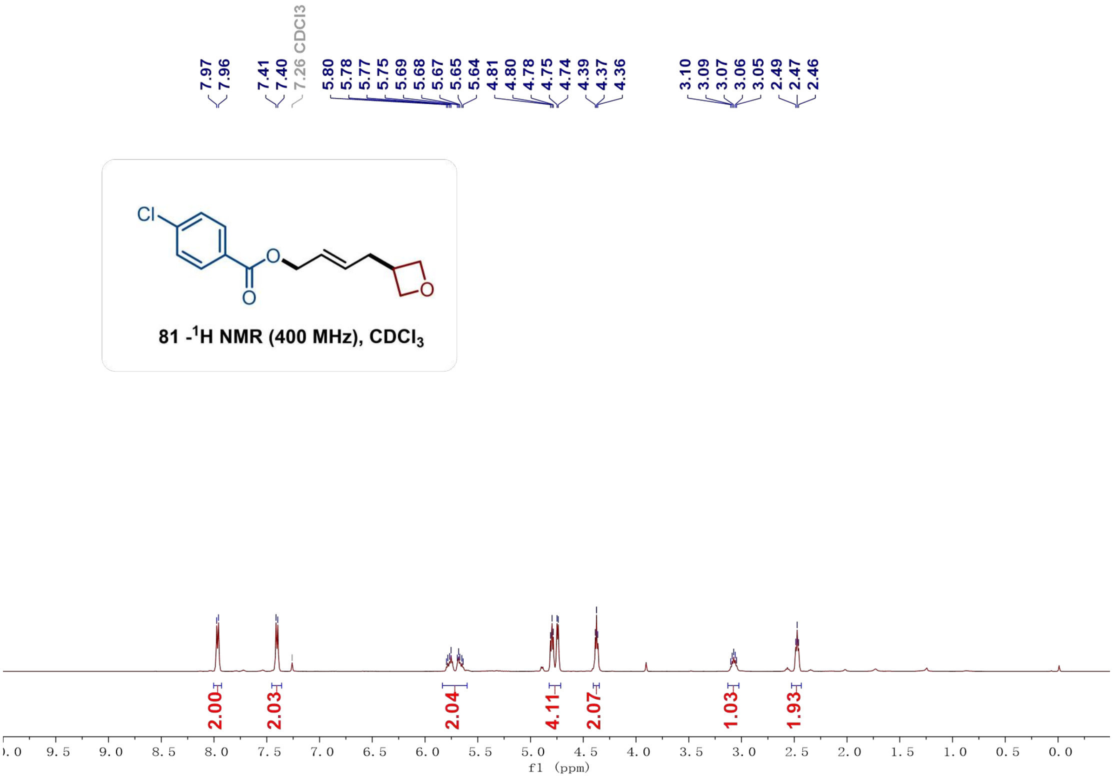

Supplementary Figure 231. ^{1}H NMR (400 MHz, $CDCl_3$) spectrum of compound **81**

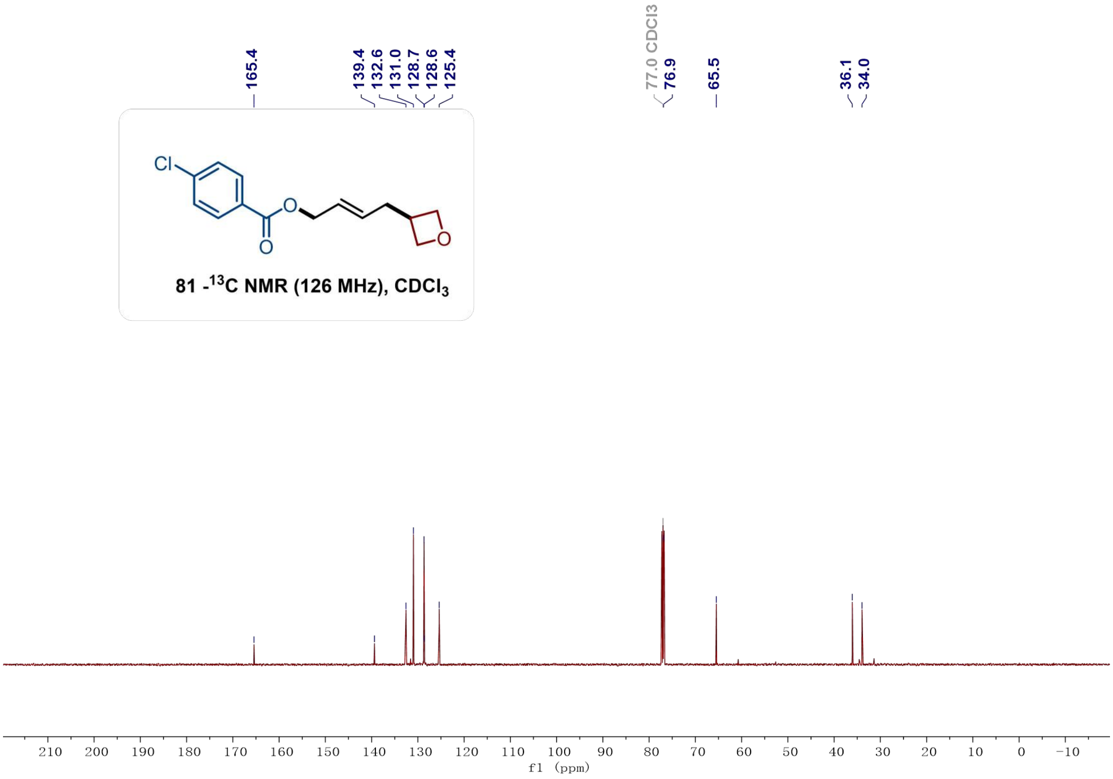

Supplementary Figure 232. ^{13}C NMR (101 MHz, $CDCl_3$) spectrum of compound **81**

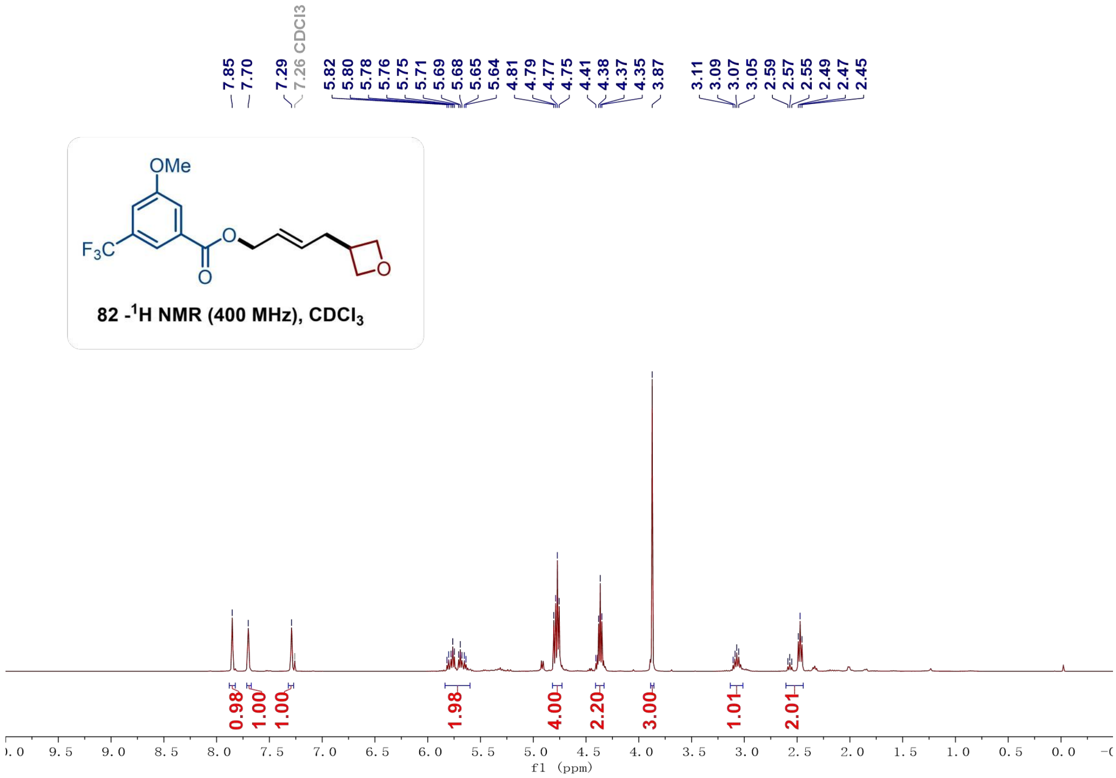

Supplementary Figure 233. ^{1}H NMR (400 MHz, $CDCl_3$) spectrum of compound **82**

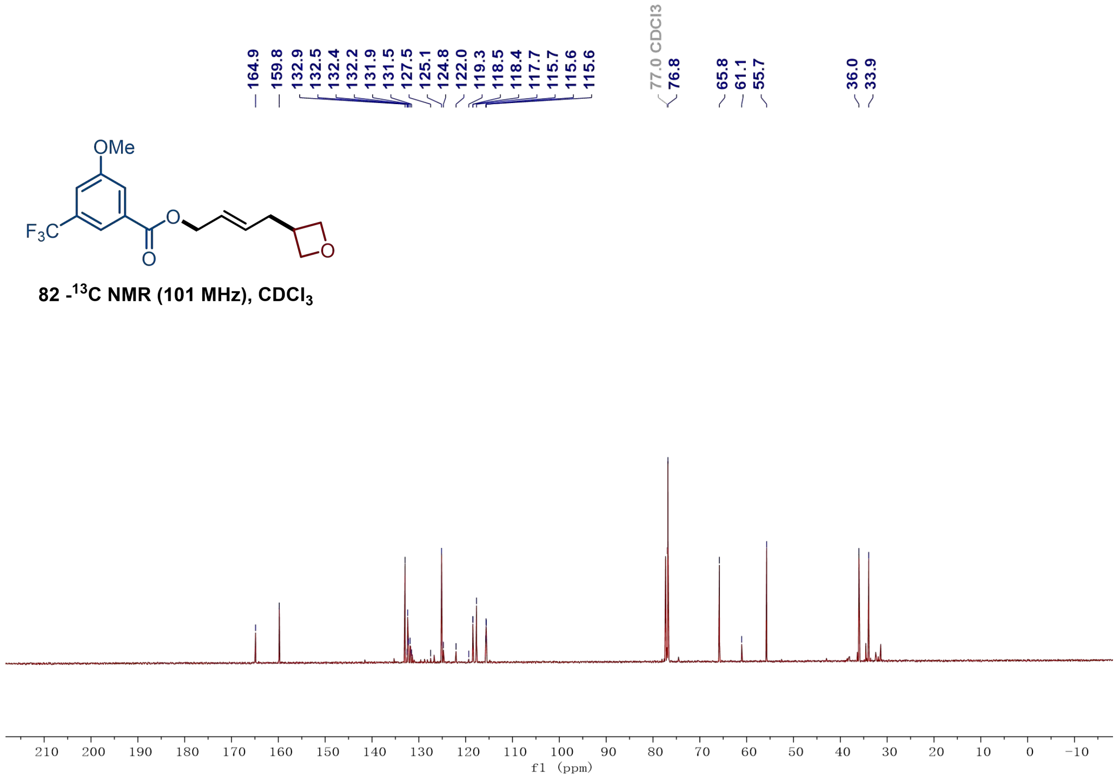

Supplementary Figure 234. ^{13}C NMR (101 MHz, $CDCl_3$) spectrum of compound **82**

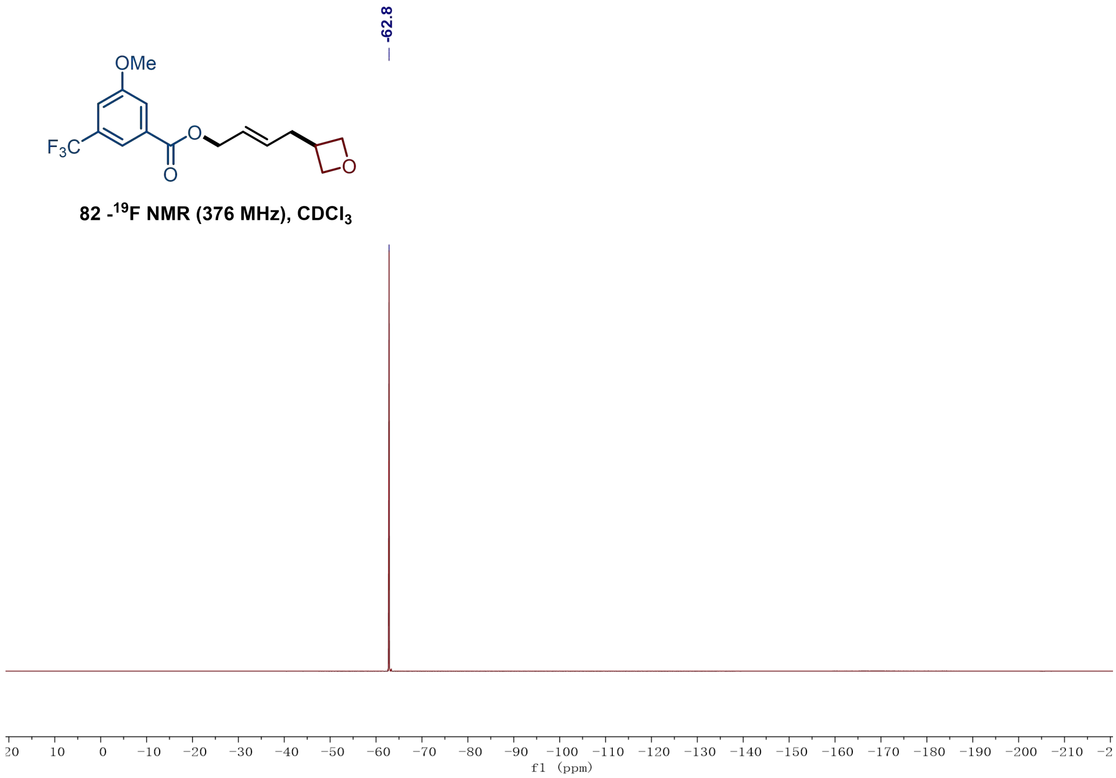

Supplementary Figure 235. ^{19}F NMR (376 MHz, $CDCl_3$) spectrum of compound **82**

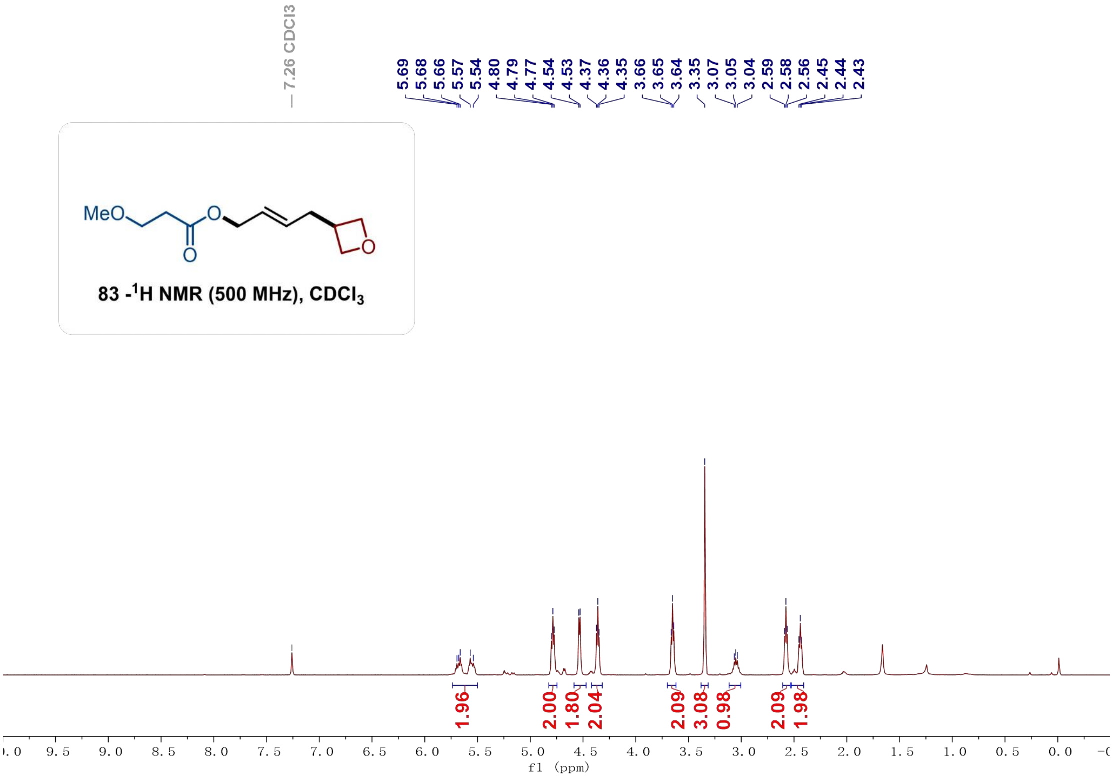

Supplementary Figure 236. ^{1}H NMR (500 MHz, $CDCl_3$) spectrum of compound **83**

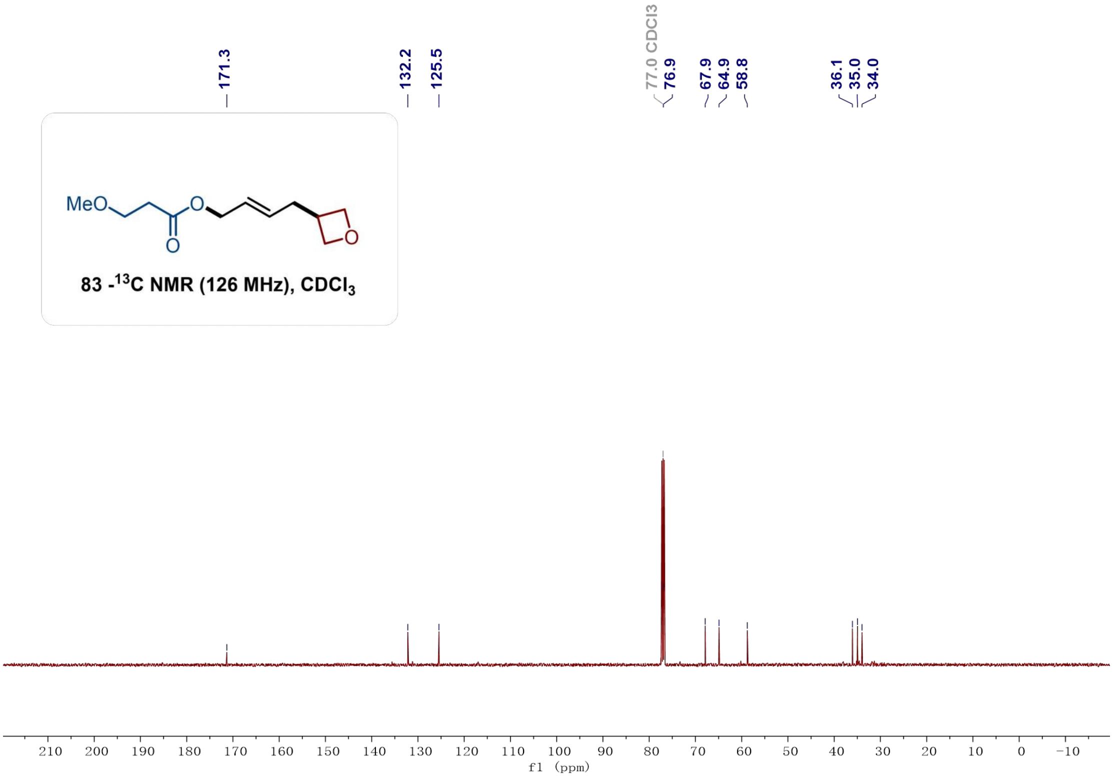

Supplementary Figure 237. ^{13}C NMR (126 MHz, $CDCl_3$) spectrum of compound **83**

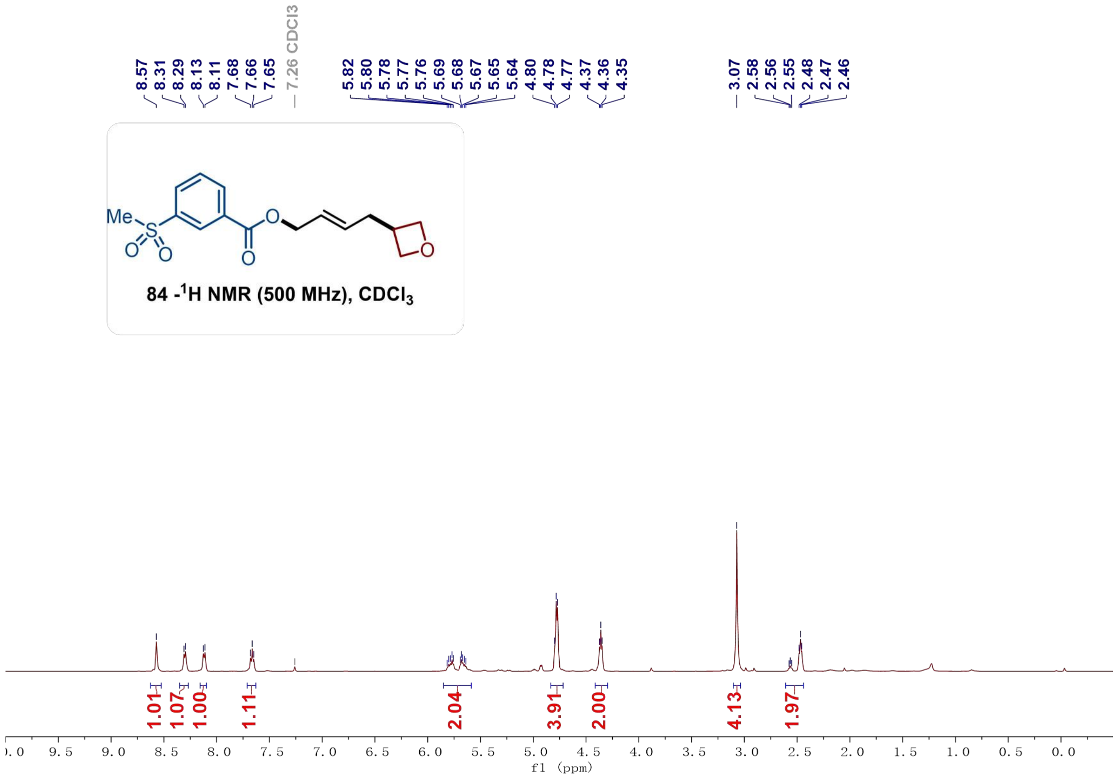

Supplementary Figure 238. ^{1}H NMR (500 MHz, $CDCl_3$) spectrum of compound **84**

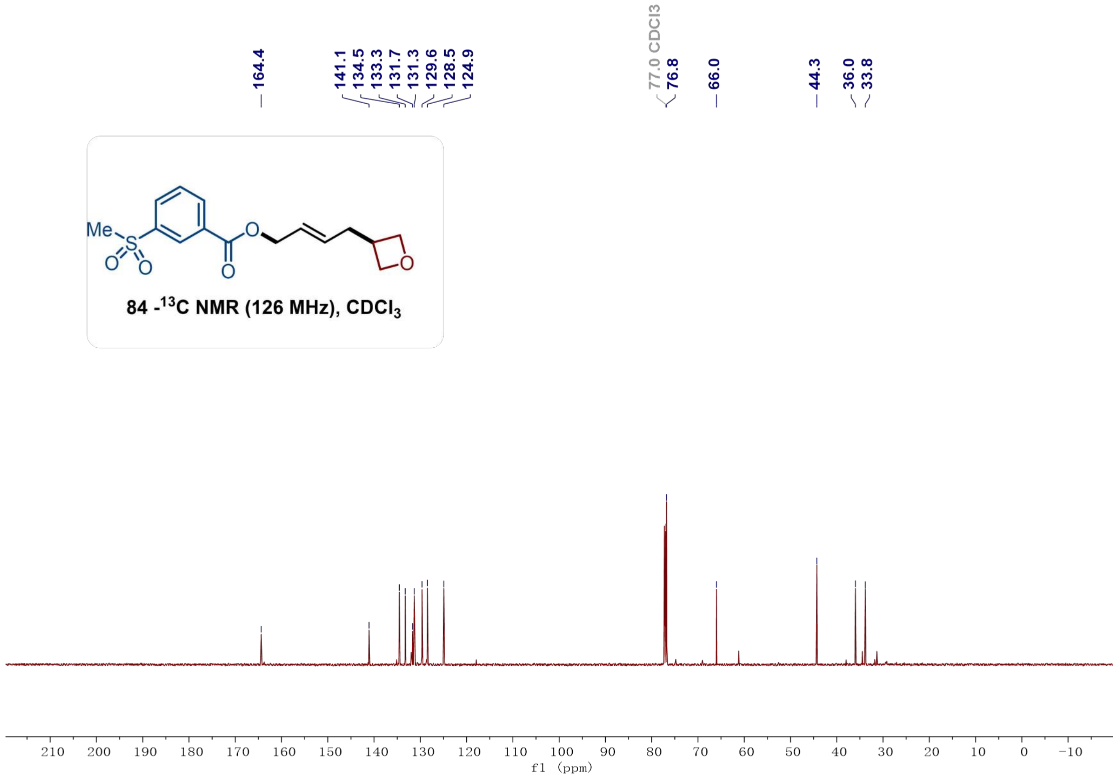

Supplementary Figure 239. ^{13}C NMR (126, MHz, $CDCl_3$) spectrum of compound **84**

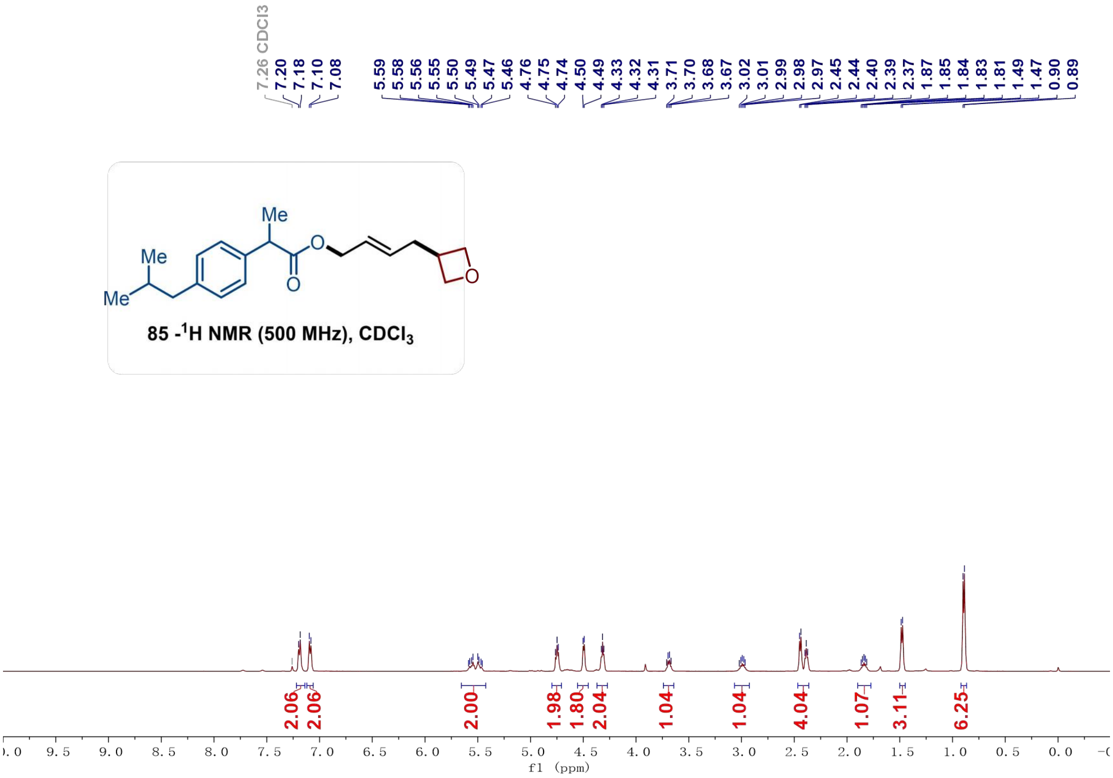

Supplementary Figure 240. ^{1}H NMR (500 MHz, $CDCl_3$) spectrum of compound **85**

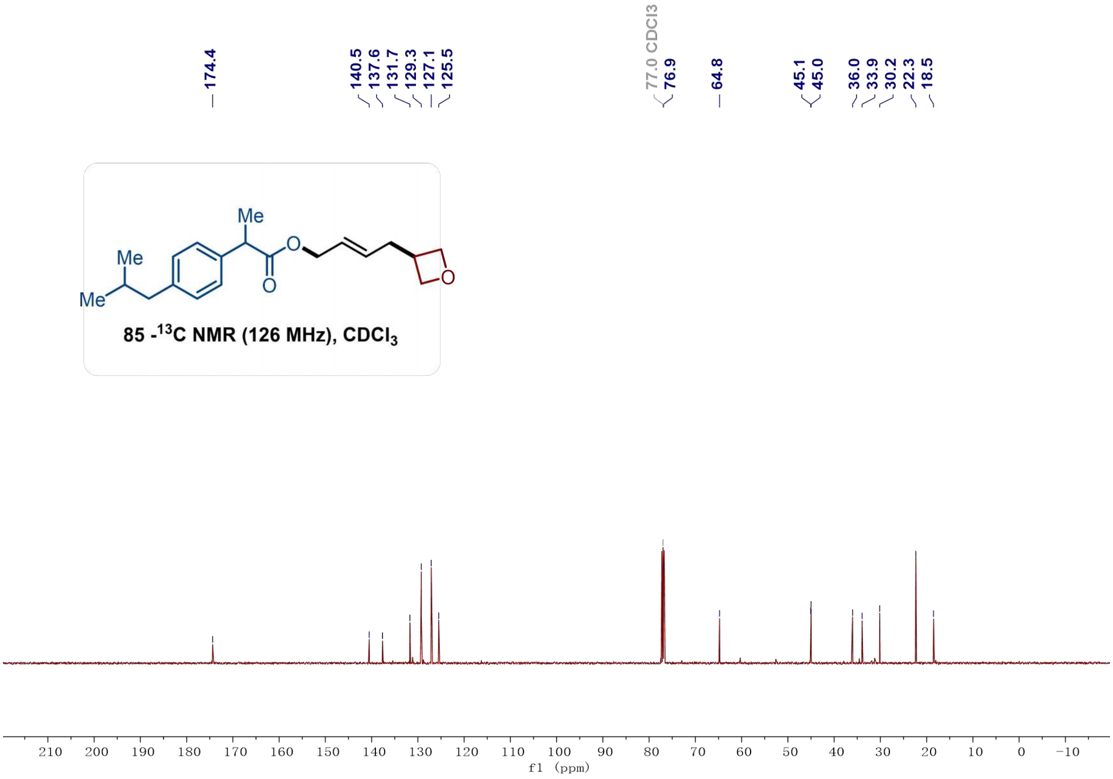

Supplementary Figure 241. ^{13}C NMR (126 MHz, $CDCl_3$) spectrum of compound **85**

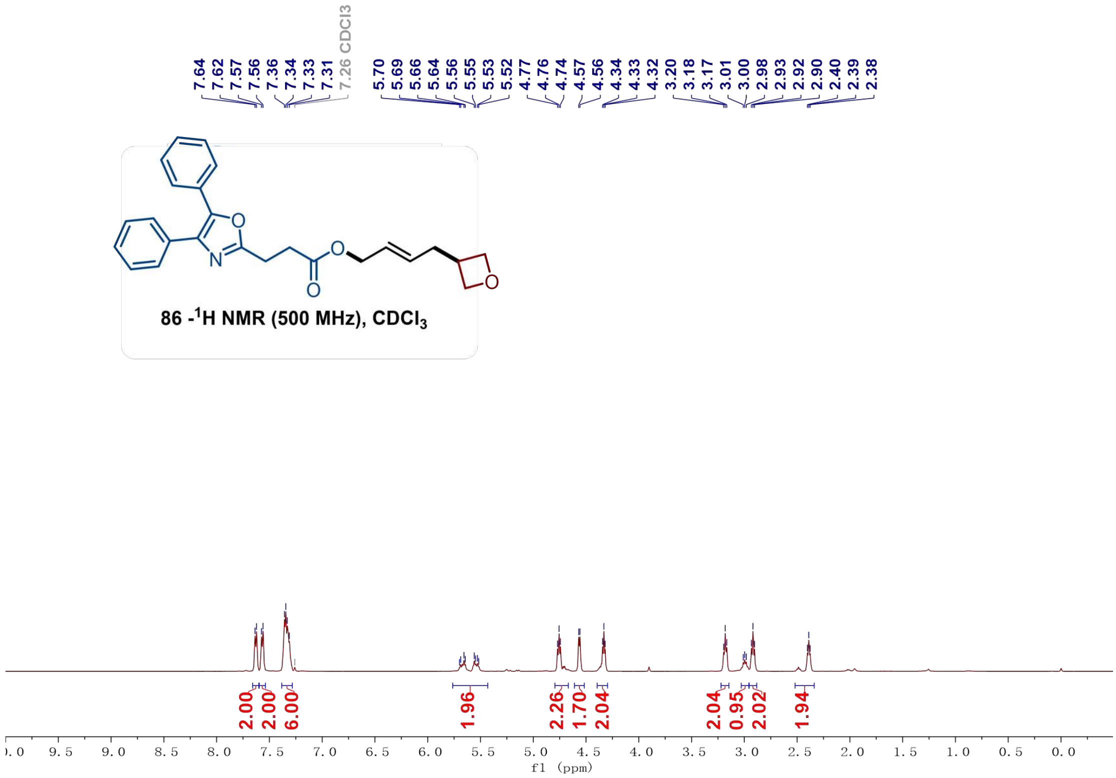

Supplementary Figure 242. ^{1}H NMR (500 MHz, $CDCl_3$) spectrum of compound **86**

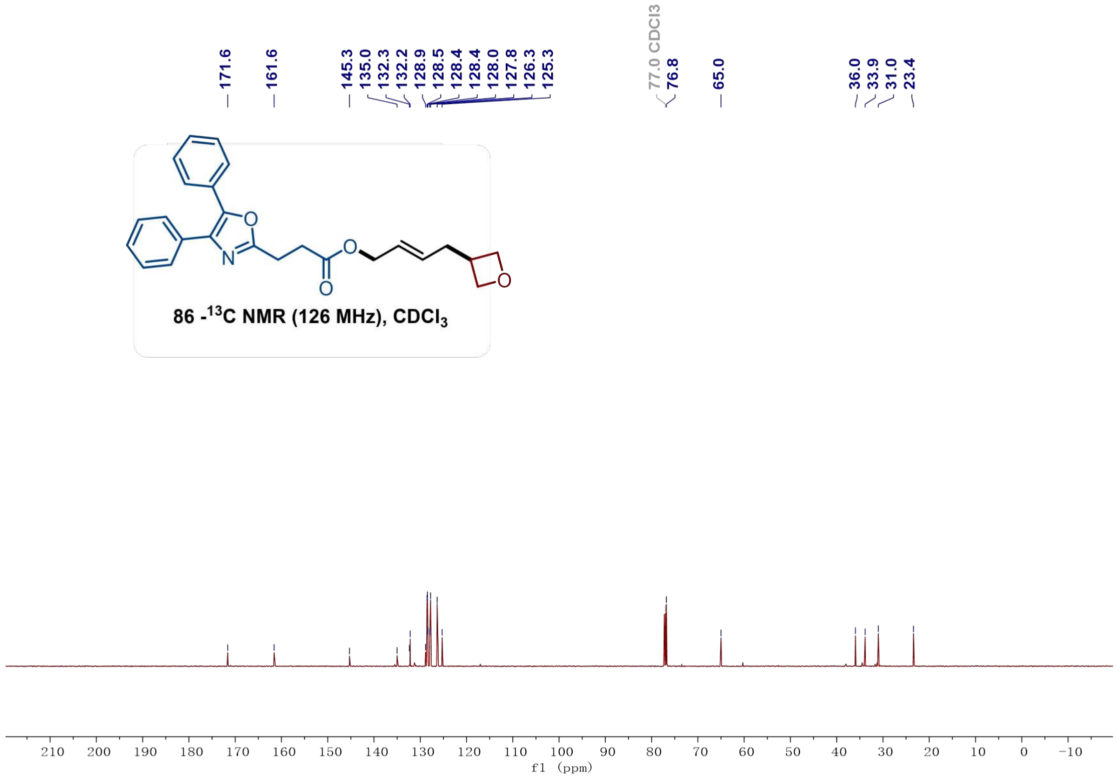

Supplementary Figure 243. ^{13}C NMR (126 MHz, $CDCl_3$) spectrum of compound **86**

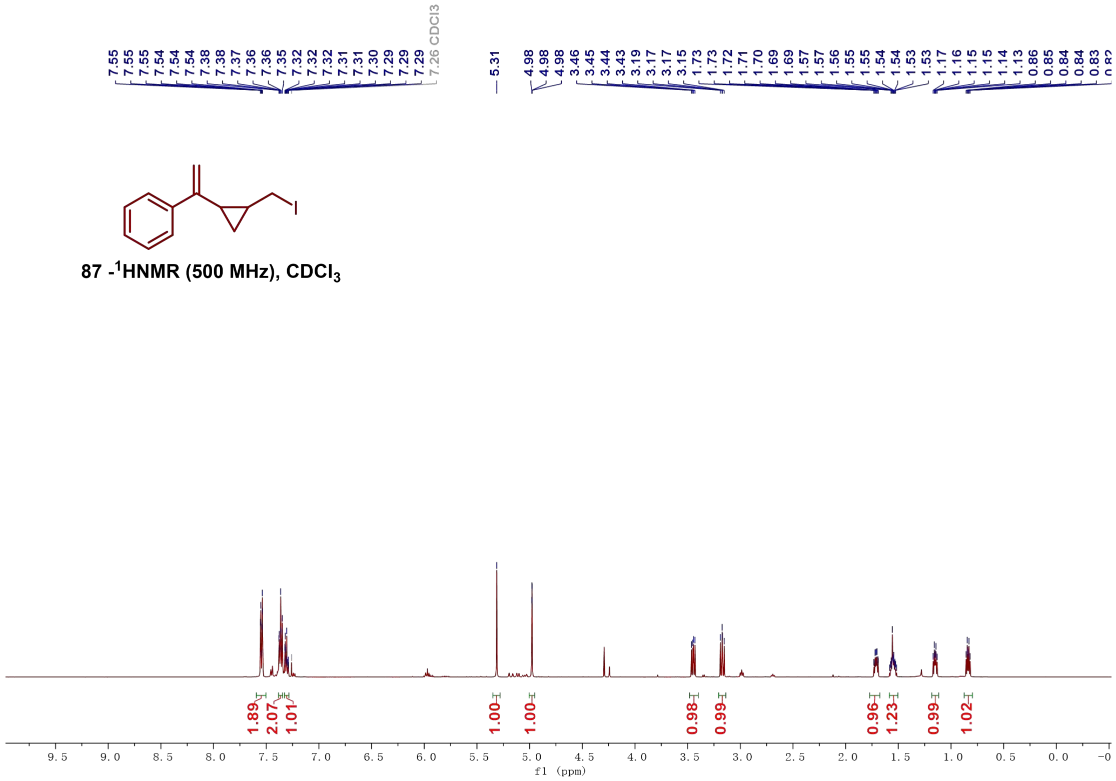

Supplementary Figure 244. ^{1}H NMR (500 MHz, $CDCl_3$) spectrum of compound **87**

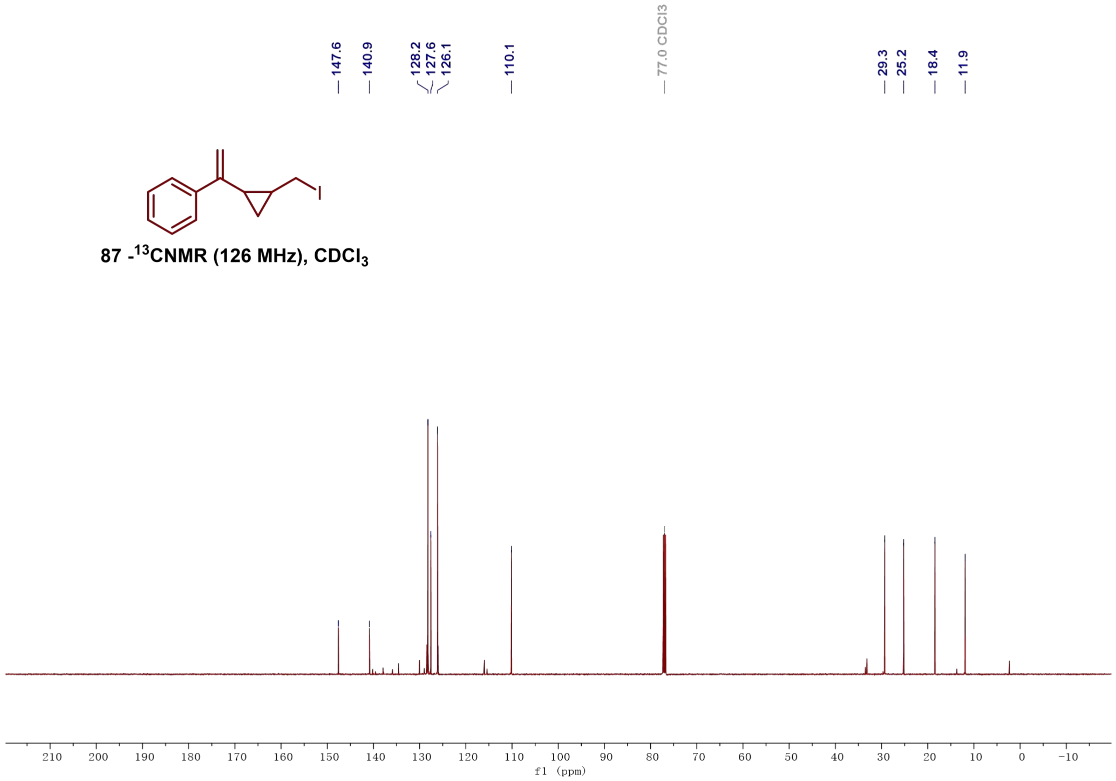

Supplementary Figure 245. ^{13}C NMR (126 MHz, $CDCl_3$) spectrum of compound **87**

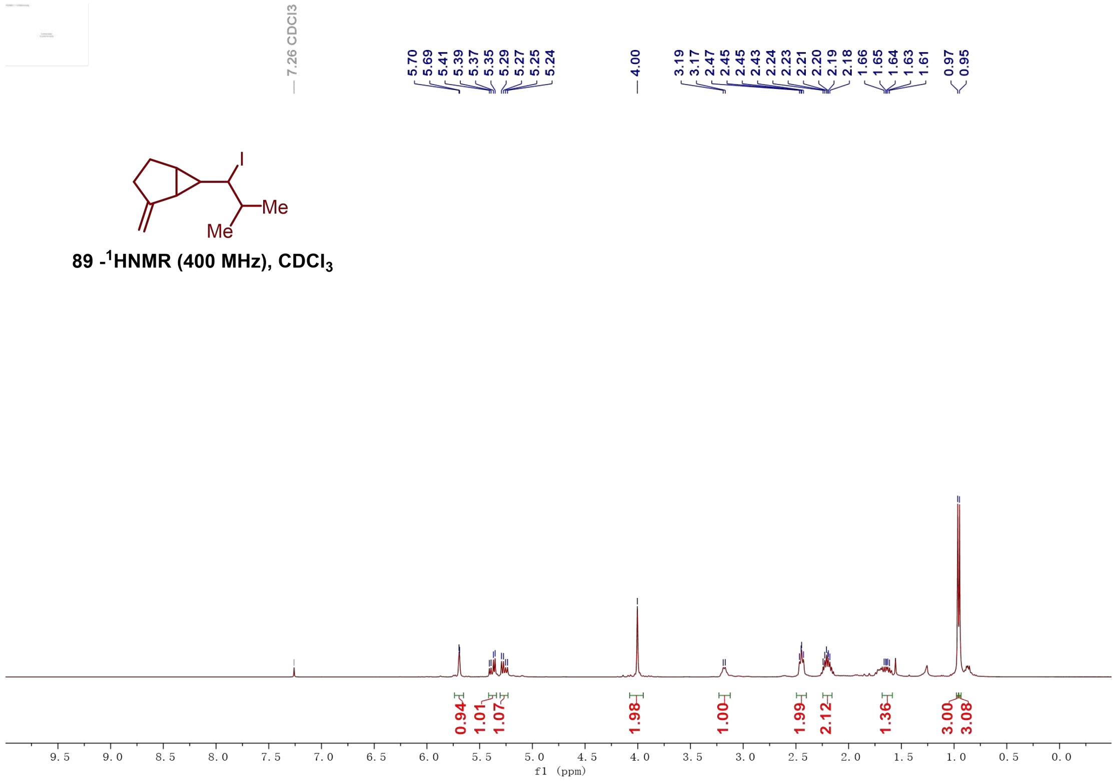

Supplementary Figure 246. ^{1}H NMR (400 MHz, $CDCl_3$) spectrum of compound **89**

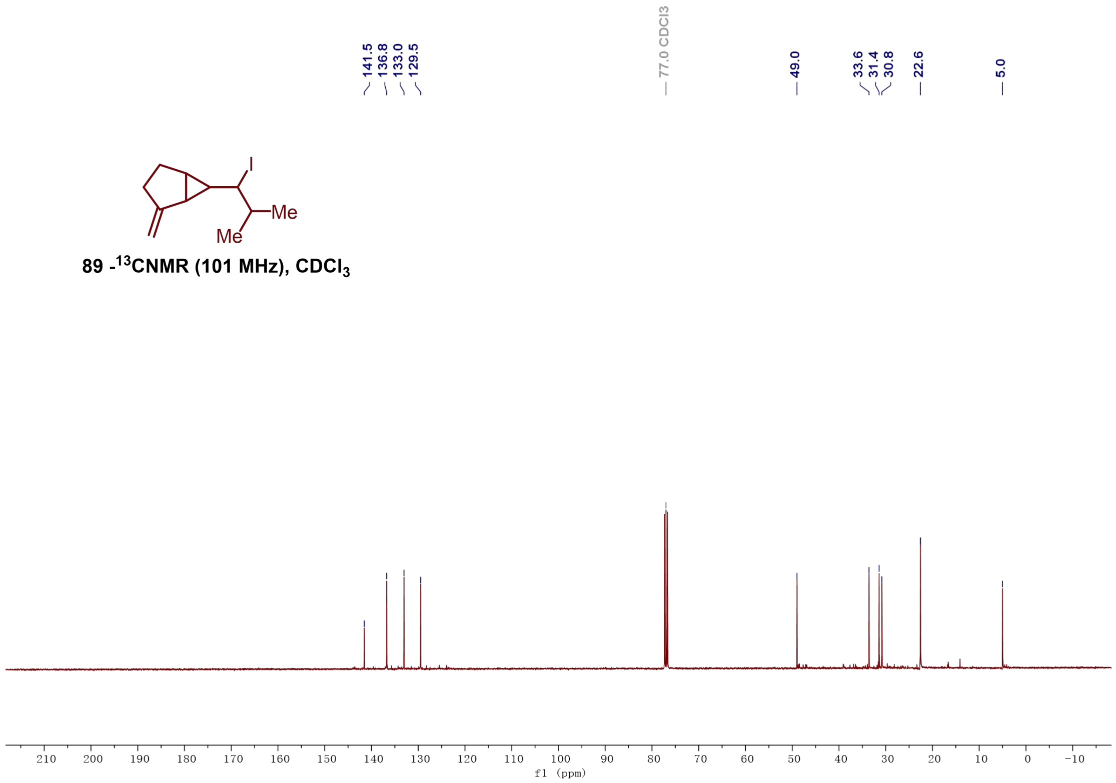

Supplementary Figure 247. ^{13}C NMR (101 MHz, $CDCl_3$) spectrum of compound **89**

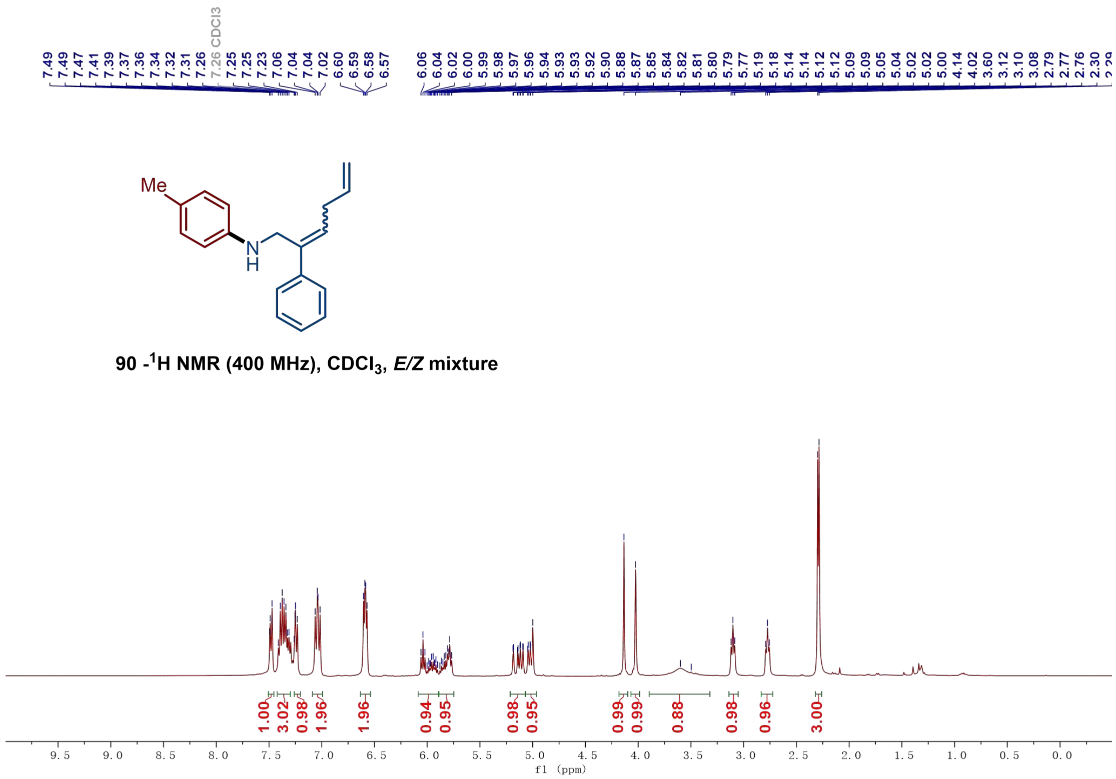

Supplementary Figure 248. ^{1}H NMR (400 MHz, $CDCl_3$) spectrum of compound **90**

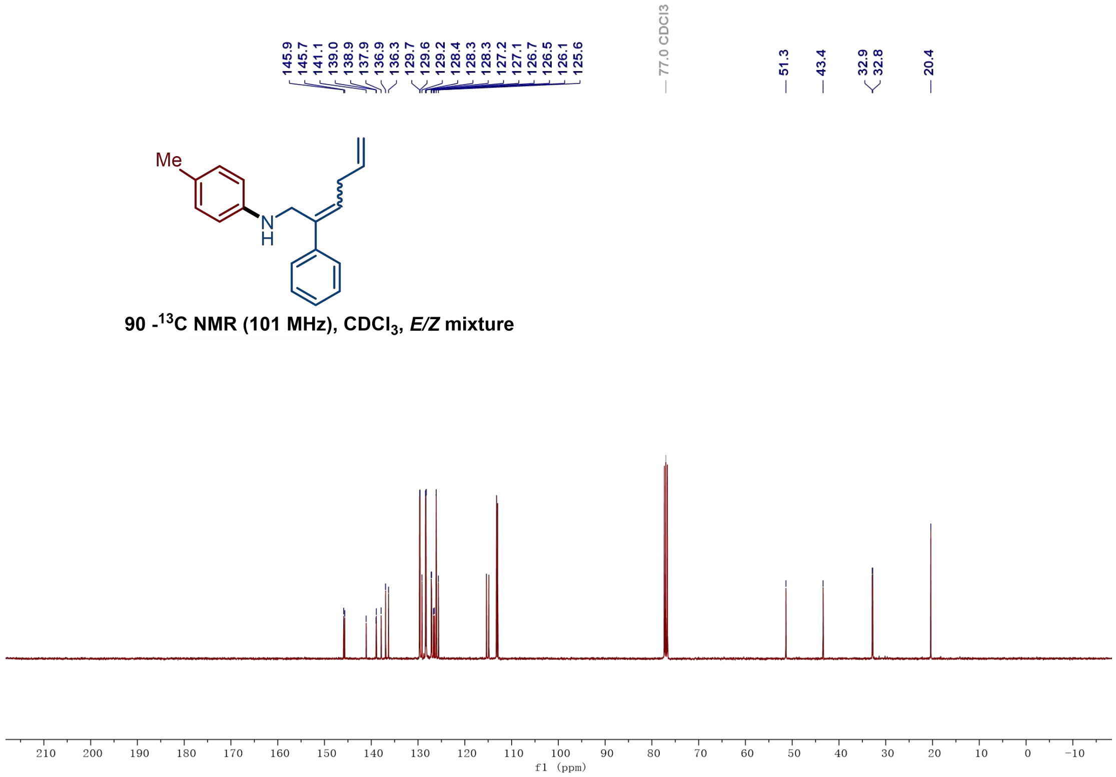

Supplementary Figure 249. ^{13}C NMR (101 MHz, $CDCl_3$) spectrum of compound **90**

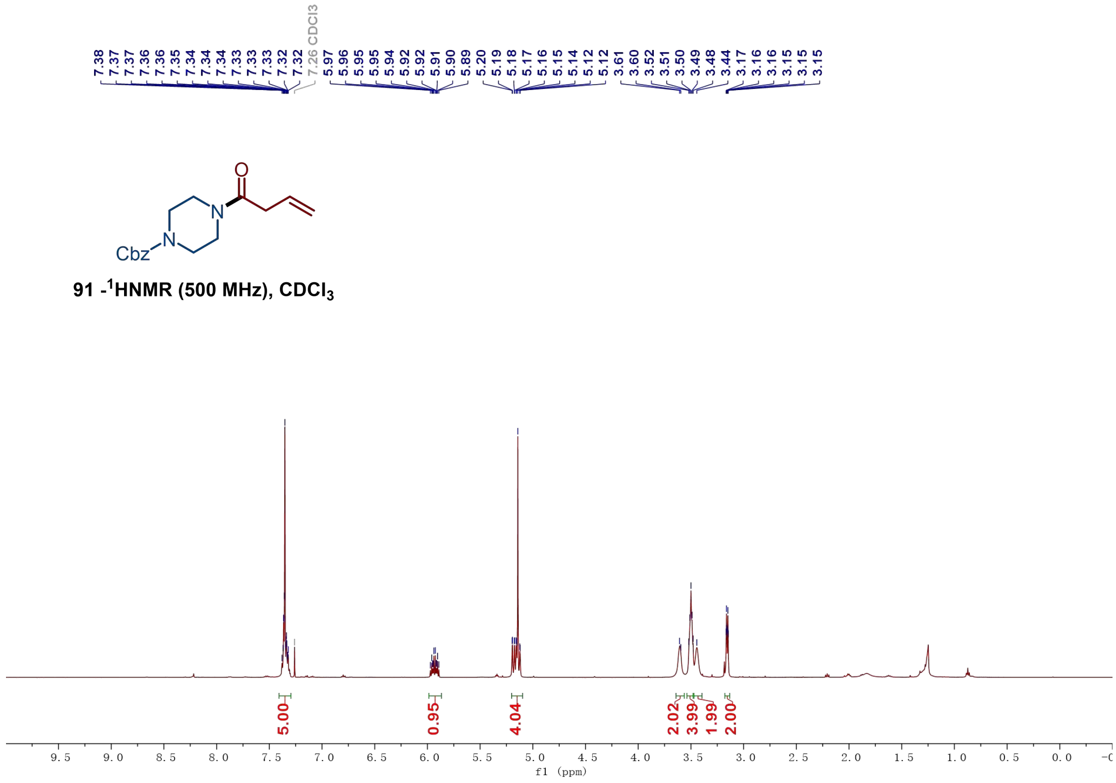

Supplementary Figure 250. ^{1}H NMR (500 MHz, $CDCl_3$) spectrum of compound **91**

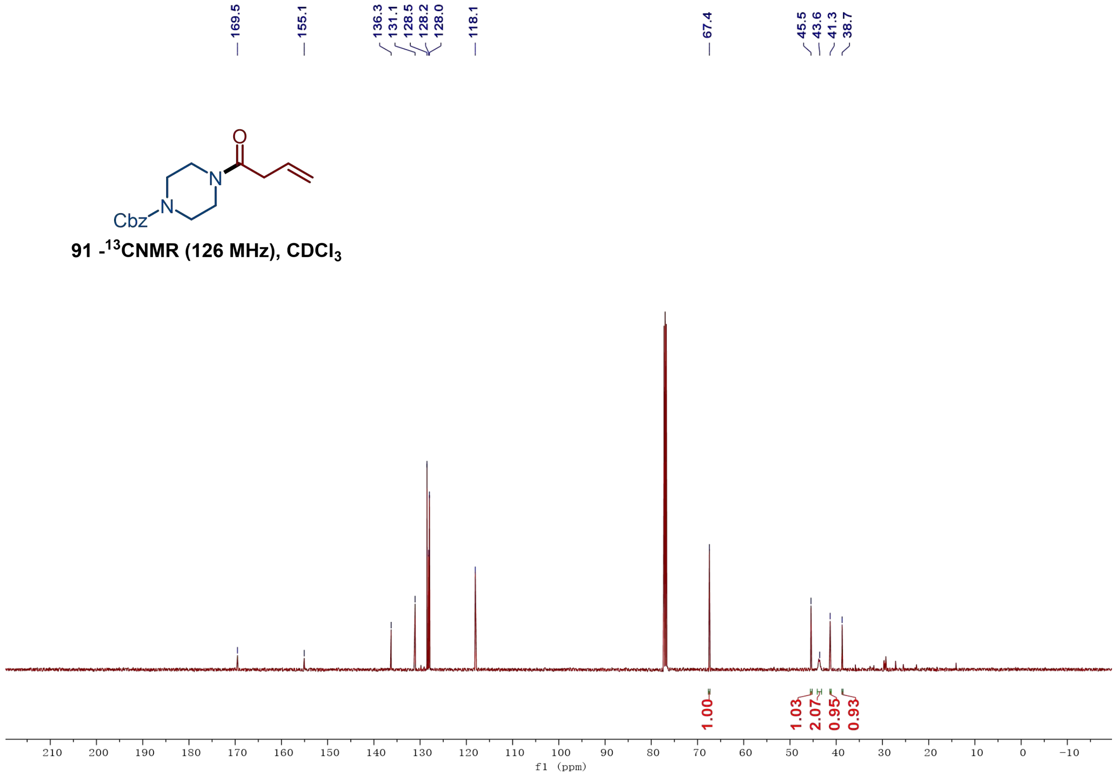

Supplementary Figure 251. ^{13}C NMR (126 MHz, $CDCl_3$) spectrum of compound **91**

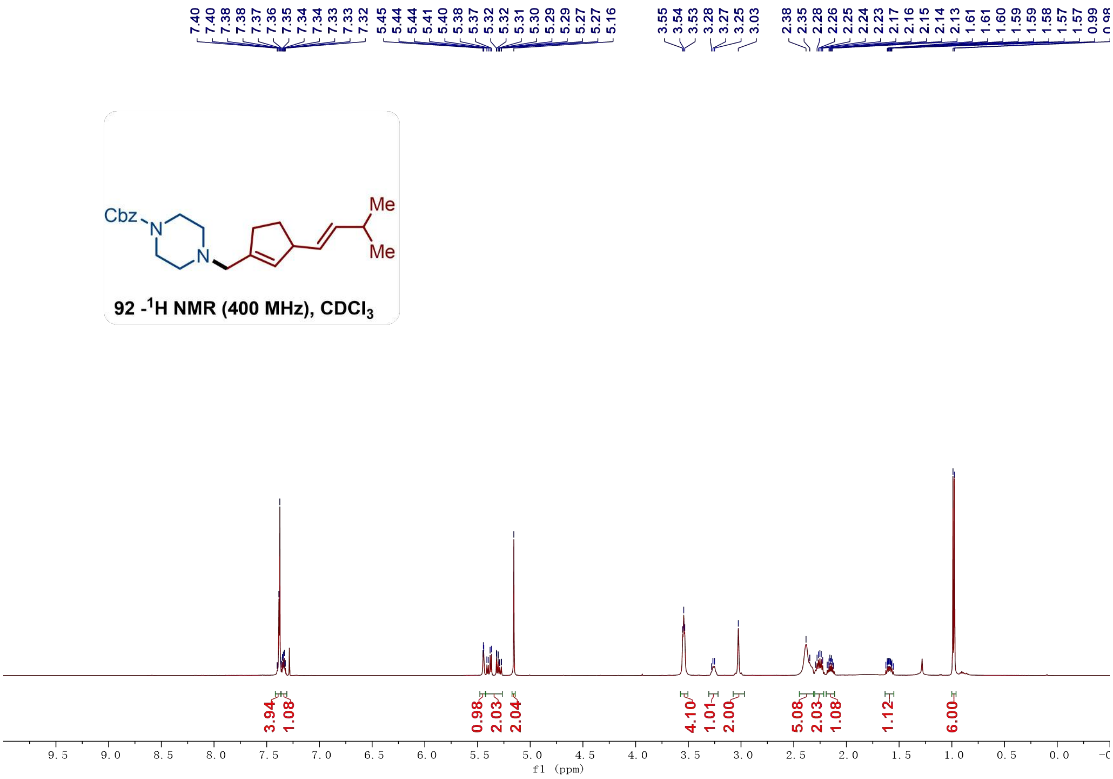

Supplementary Figure 252. ^{1}H NMR (400 MHz, $CDCl_3$) spectrum of compound **92**

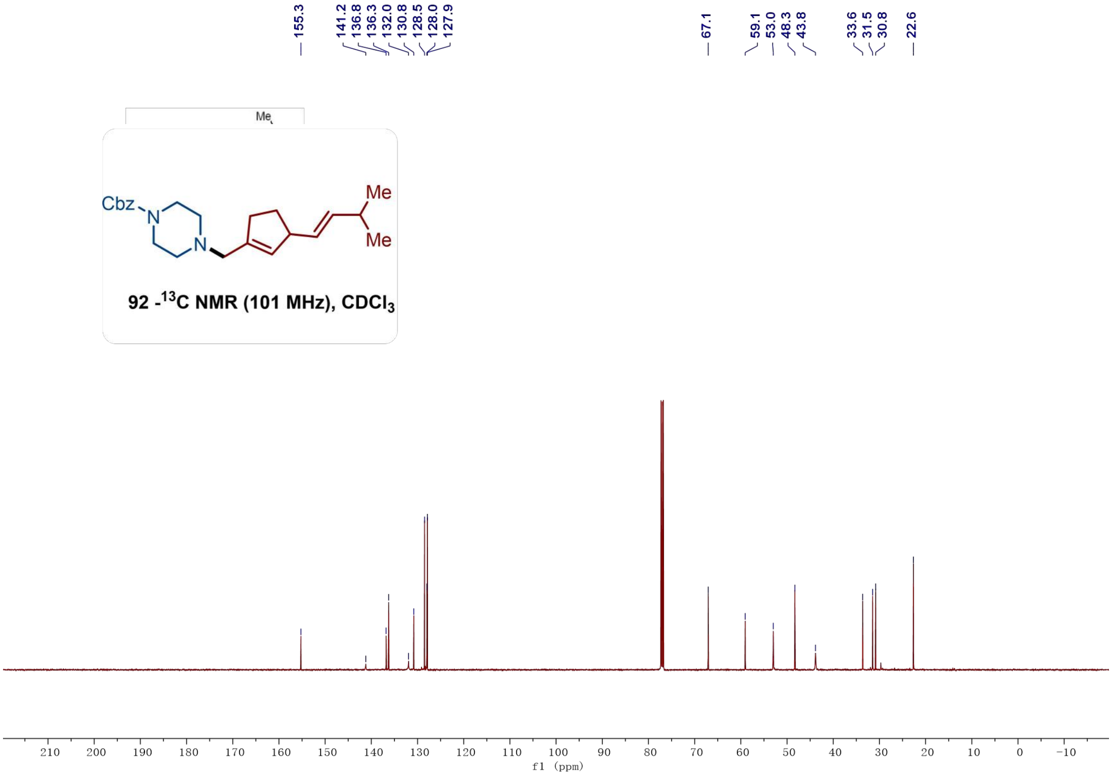

Supplementary Figure 253. ^{13}C NMR (101 MHz, $CDCl_3$) spectrum of compound **92**

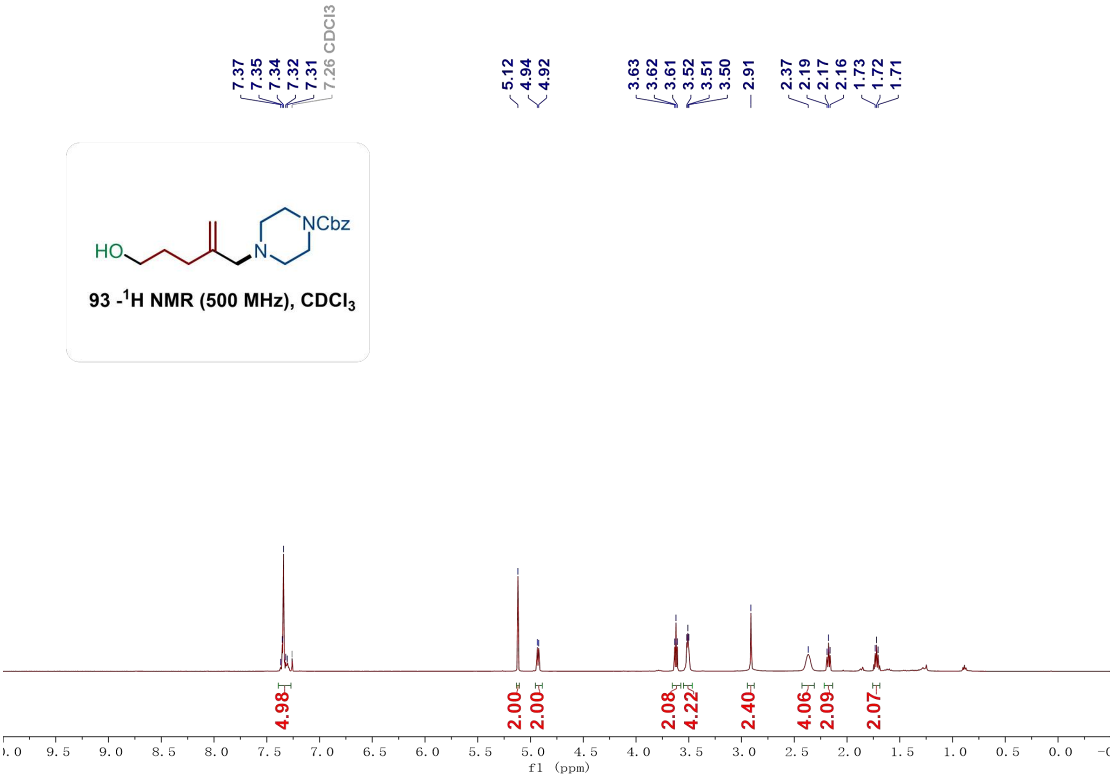

Supplementary Figure 254. ^{1}H NMR (500 MHz, $CDCl_3$) spectrum of compound **93**

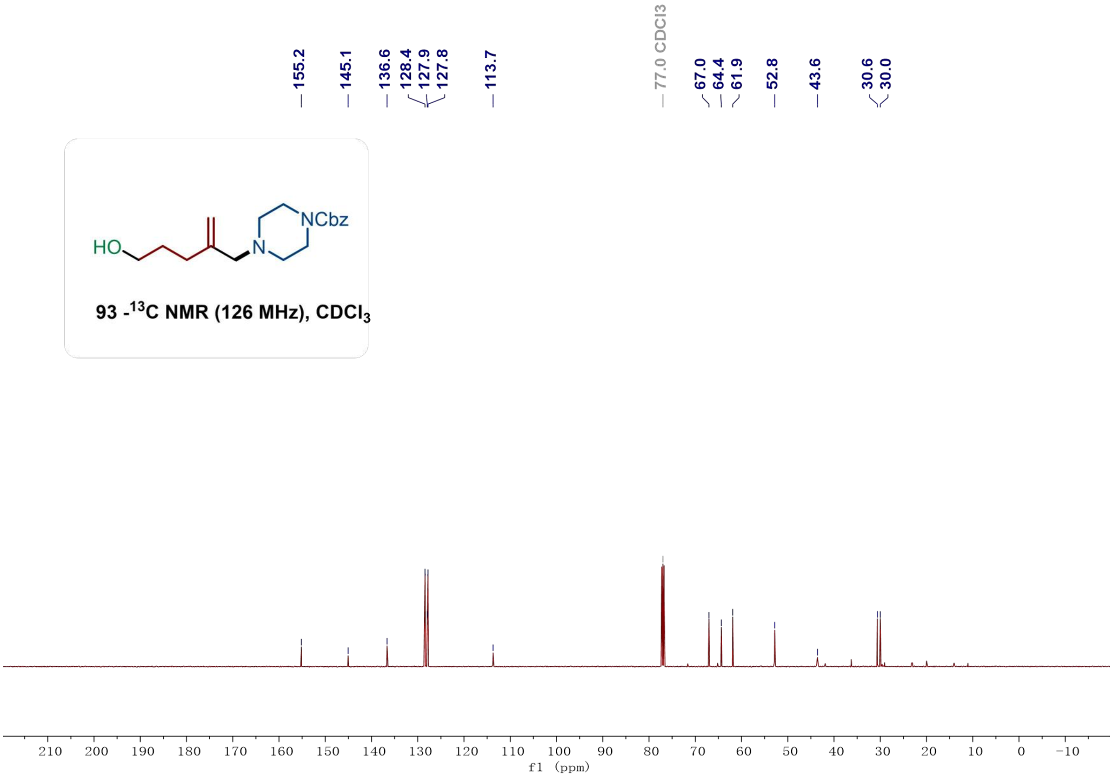

Supplementary Figure 255. ^{13}C NMR (126 MHz, $CDCl_3$) spectrum of compound **93**

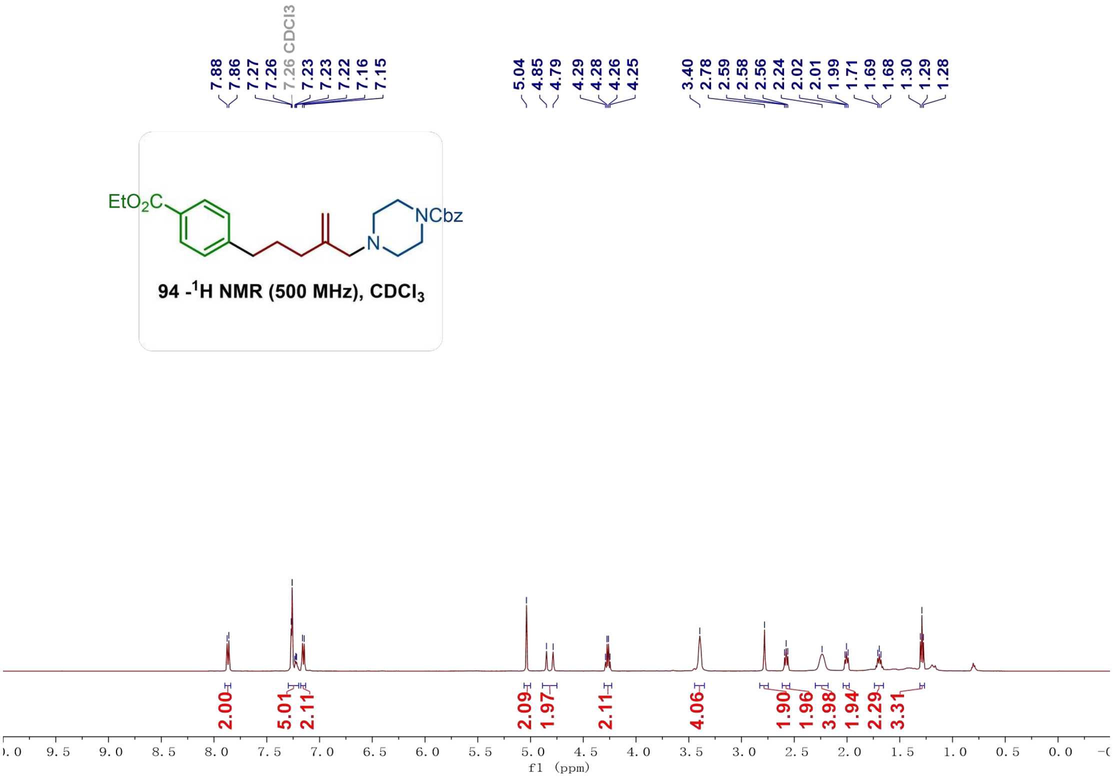

Supplementary Figure 256. ^{1}H NMR (500 MHz, $CDCl_3$) spectrum of compound **94**

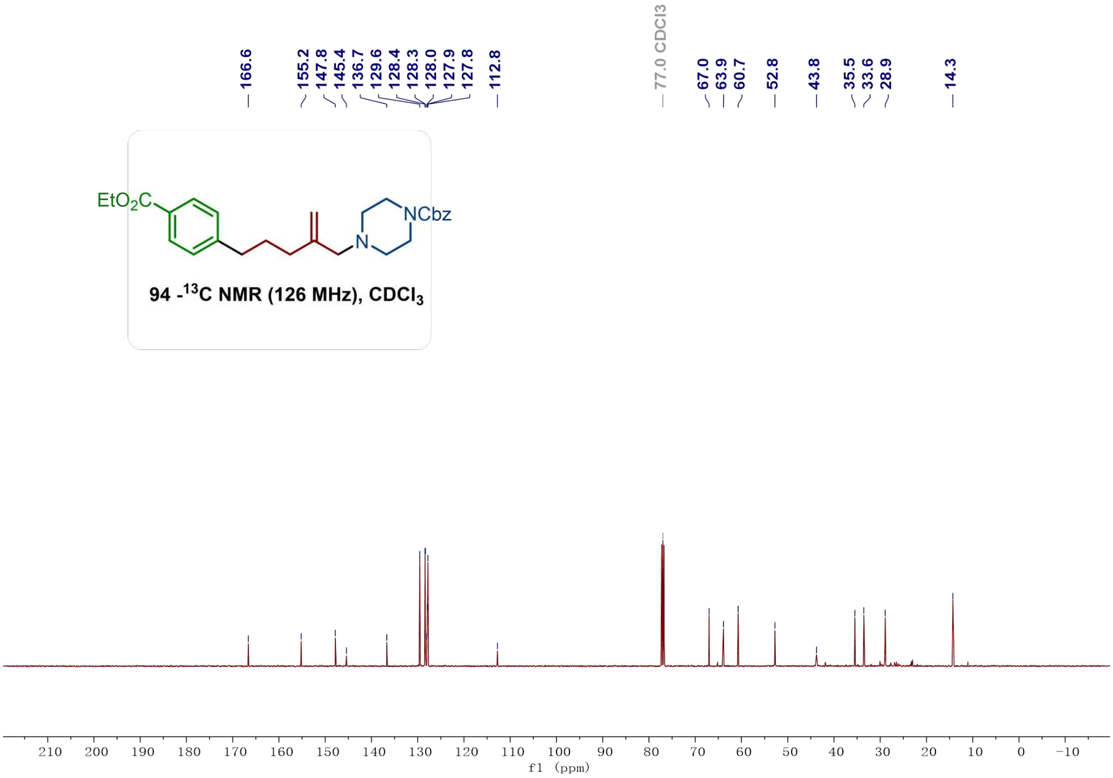

Supplementary Figure 257. ^{13}C NMR (126 MHz, $CDCl_3$) spectrum of compound **94**

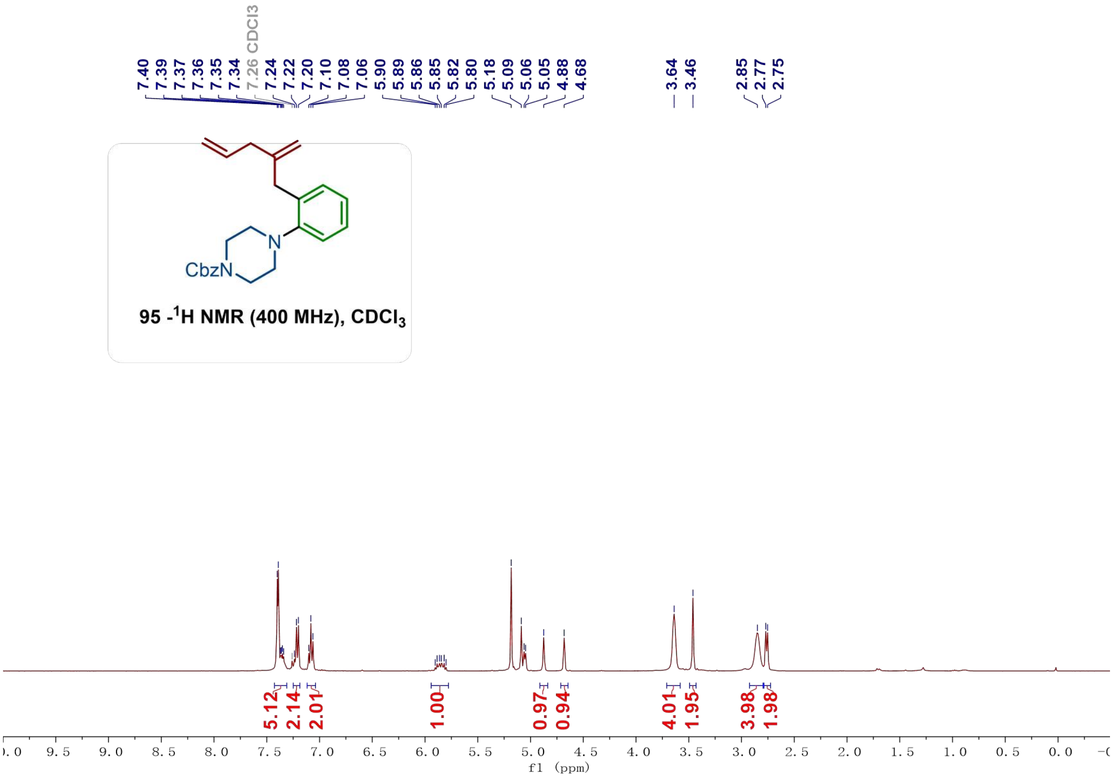

Supplementary Figure 258. ^{1}H NMR (400 MHz, $CDCl_3$) spectrum of compound **95**

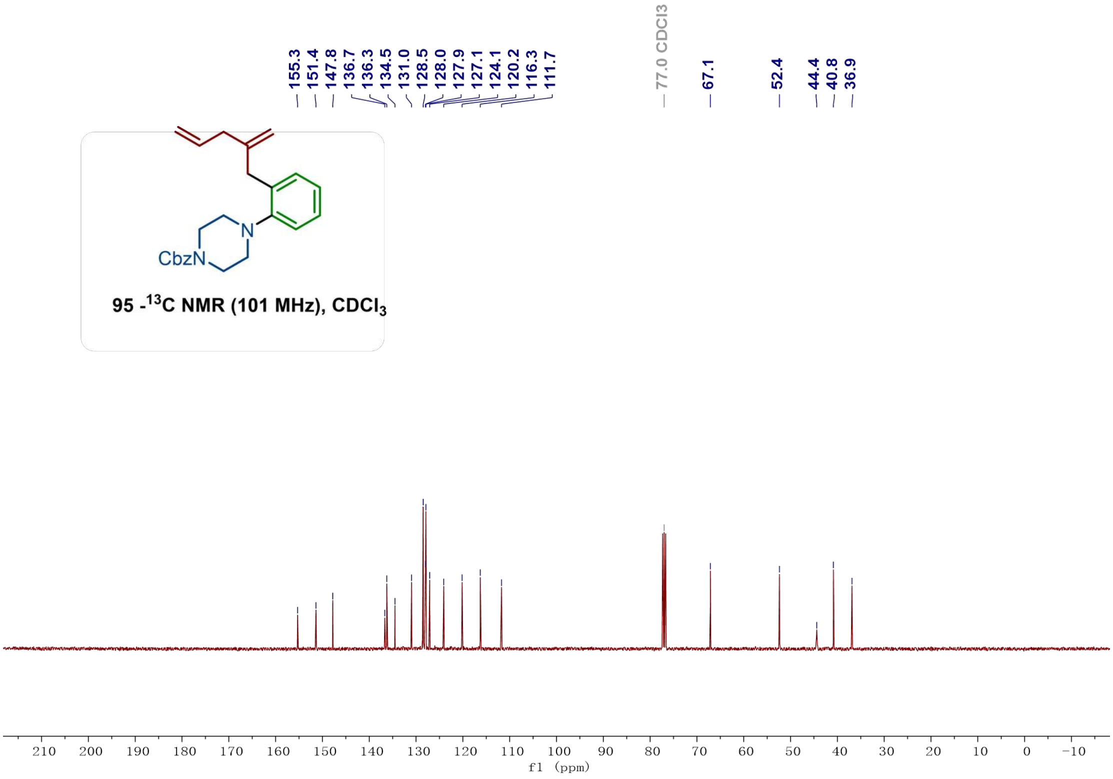

Supplementary Figure 259. ^{13}C NMR (101 MHz, $CDCl_3$) spectrum of compound **95**

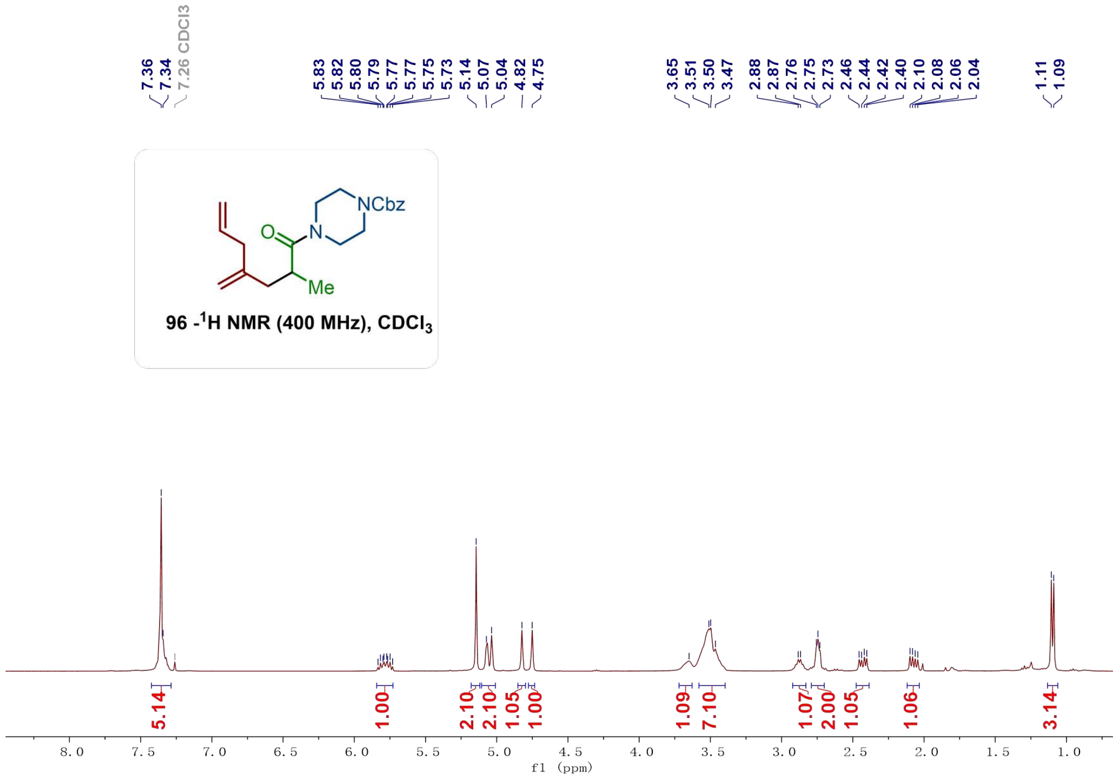

Supplementary Figure 260. ^{1}H NMR (500 MHz, $CDCl_3$) spectrum of compound **96**

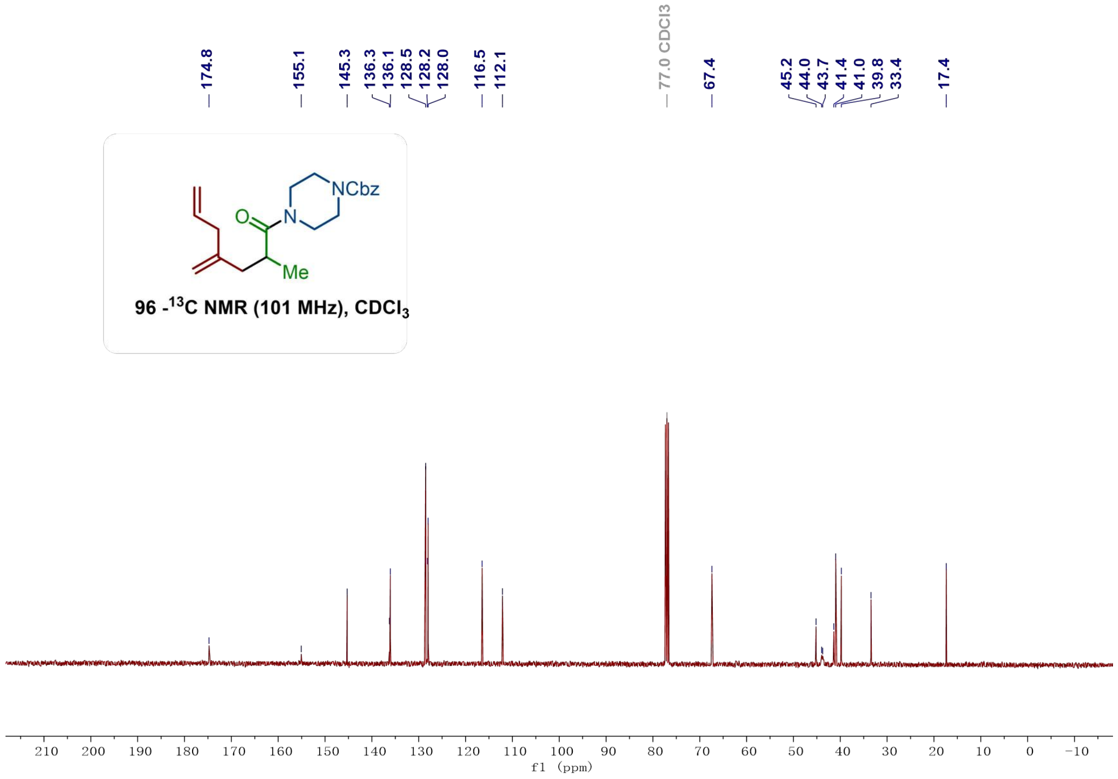

Supplementary Figure 261. ^{13}C NMR (101 MHz, $CDCl_3$) spectrum of compound **96**

Supplementary Reference

1. Evans, R.D., Magee, J.W. & Schauble, J.H. Halocyclization of Unsaturated Alcohols and Carboxylic Acids Using Bis(sym-collidine)iodine(I) Perchlorate. *Synthesis*. **11**, 862-868 (1988).
2. Mailig, M., Hazra, A., Armstrong, M. K. & Lalic, G. Catalytic Anti-Markovnikov Hydroallylation of Terminal and Functionalized Internal Alkynes: Synthesis of Skipped Dienes and Trisubstituted Alkenes. *J. Am. Chem. Soc*. **139**, 6969-6977 (2017).
3. Cant, A. A., Bertrand, G. H. V., Henderson, J. L., Roberts, L. & Greaney, M. F. The Benzyne Aza-Claisen Reaction. *Angew. Chem. Int. Ed*. **48**, 5199-5202 (2009).
4. Dong, V. M. & MacMillan, D. W. C. Design of a New Cascade Reaction for the Construction of Complex Acyclic Architecture: The Tandem Acyl-Claisen Rearrangement. *J. Am. Chem. Soc.* **123**, 2448-2449 (2001).
5. Gaussian 16, Revision C.01, Frisch, M. J., Trucks, G. W., Schlegel, H. B., Scuseria, G. E., Robb, M. A., Cheeseman, J. R., Scalmani, G., Barone, V., Petersson, G. A., Nakatsuji, H., Li, X., Caricato, M., Marenich, A. V., Bloino, J., Janesko, B. G., Gomperts, R., Mennucci, B., Hratchian, H. P., Ortiz, J. V., Izmaylov, A. F., Sonnenberg, J. L., Williams-Young, D., Ding, F., Lipparini, F., Egidi, F., Goings, J., Peng, B., Petrone, A., Henderson, T., Ranasinghe, D., Zakrzewski, V. G., Gao, J., Rega, N., Zheng, G., Liang, W., Hada, M., Ehara, M., Toyota, K., Fukuda, R., Hasegawa, J., Ishida, M., Nakajima, T., Honda, Y., Kitao, O., Nakai, H., Vreven, T., Throssell, K., Montgomery, J. A., Jr., Peralta, J. E., Ogliaro, F., Bearpark, M. J., Heyd, J. J., Brothers, E. N., Kudin, K. N., Staroverov, V. N., Keith, T. A., Kobayashi, R., Normand, J., Raghavachari, K., Rendell, A. P., Burant, J. C., Iyengar, S. S., Tomasi, J., Cossi, M., Millam, J. M., Klene, M., Adamo, C., Cammi, R., Ochterski, J. W., Martin, R. L., Morokuma, K., Farkas, O., Foresman, J. B., Fox, D. J. Gaussian, Inc., Wallingford CT, 2016.
6. Maeda, S., Ohno, K., Morokuma, K. Systematic exploration of the mechanism of chemical reactions: the global reaction route mapping (GRRM) strategy using the ADDF and AFIR methods. *Phys. Chem. Chem. Phys.***15**, 3683-3701 (2013).
7. Grimme, S., Antony, J., Ehrlich, S., Krieg, H. A consistent and accurate ab initio parametrization of density functional dispersion correction (DFT-D) for the 94 elements H-Pu *J. Chem. Phys.* **132**, 154104 (2010).
