# Supplementary material for: Light-driven radical copper-catalyzed allylic amination via allylic copper intermediates
Source: Nat Commun. 2025 Nov 26;16:10425. doi: 10.1038/s41467-025-64951-5 (PMC12657895; doi:10.1038/s41467-025-64951-5)
Supplement: Supplementary file 2 — Description of Additional Supplementary Files [file 41467_2025_64951_MOESM2_ESM.pdf]

Description of Additional Supplementary Files

File Name: Supplementary Data 1
Description: Cartesian coordinates and energy of structures showed in the Figure 8 and Supplementary Figure 13.

File Name: Supplementary Data 2
Description: Cartesian coordinates and energy of structures showed in the Supplementary Figure 16&17.

File Name: Supplementary Data 3
Description: Cartesian coordinates and energy of structures showed in the Supplementary Figure 18.
